# Genome-scale approaches for discovering novel nonconventional splicing substrates of the Ire1 nuclease

**DOI:** 10.1186/gb-2004-6-1-r3

**Published:** 2004-12-22

**Authors:** Maho Niwa, Christopher K Patil, Joe DeRisi, Peter Walter

**Affiliations:** 1 Howard Hughes Medical Institute and Department of Biochemistry and Biophysics, University of California at San Francisco, San Francisco, CA 94143-2200, USA; 2 Current address: Division of Biology, Section of Molecular Biology, University of California at San Diego, La Jolla, CA 92093-0366, USA; 3 Current address: Lawrence Berkeley National Laboratory, Life Sciences Division, 1 Cyclotron Road, Berkeley, CA 94720, USA

## Abstract

**Background:**

The unfolded protein response (UPR) allows intracellular feedback regulation that adjusts the protein-folding capacity of the endoplasmic reticulum (ER) according to need. The signal from the ER lumen is transmitted by the ER-transmembrane kinase Ire1, which upon activation displays a site-specific endoribonuclease activity. Endonucleolytic cleavage of the intron from the *HAC1 *mRNA (encoding a UPR-specific transcription factor) is the first step in a nonconventional mRNA splicing pathway; the released exons are then joined by tRNA ligase. Because only the spliced mRNA is translated, splicing is the key regulatory step of the UPR.

**Results:**

We developed methods to search for additional mRNA substrates of Ire1p in three independent lines of genome-wide analysis. These methods exploited the well characterized enzymology and genetics of the UPR and the yeast genome sequence in conjunction with microarray-based detection. Each method successfully identified *HAC1 *mRNA as a substrate according to three criteria: *HAC1 *mRNA is selectively cleaved *in vitro *by Ire1; the *HAC1 *mRNA sequence contains two predicted Ire1 cleavage sites; and *HAC1 *mRNA is selectively degraded in tRNA ligase mutant cells.

**Conclusion:**

Within the limits of detection, no other mRNA satisfies any of these criteria, suggesting that a unique nonconventional mRNA-processing mechanism has evolved solely for carrying out signal transduction between the ER and the nucleus. The approach described here, which combines biochemical and genetic 'fractionation' of mRNA with a novel application of cDNA microarrays, is generally applicable to the study of pathways in which RNA metabolism and alternative splicing have a regulatory role.

## Abstract 2

Three different genome-scale screens indicate that the *HAC1 *mRNA is the only substrate for the Ire1 nuclease in yeast.

## Background

The unfolded protein response (UPR) regulates the protein-folding and secretory capacity of eukaryotic cells by monitoring conditions within the endoplasmic reticulum (ER) and regulating a downstream gene-expression program (reviewed in [1-3]). In yeast, about 5% of the genome is under the transcriptional control of the UPR [4,5]. Induction of this vast set of genes is thought to lead to a restructuring of the secretory pathway to allow an increased protein flux to the cell surface and enable the cell to tolerate protein-folding stress. Hence, the UPR adjusts the secretory capacity of cells by feedback regulation.

The identification and characterization of the UPR signaling components revealed a unique mechanism of signal transduction whose salient features are conserved among all eukaryotes. The UPR is initiated when the amino-terminal portion of the serine/threonine ER-transmembrane kinase Ire1 detects unfolded proteins within the ER lumen [6,7]. Accumulation of unfolded proteins sequesters chaperones and thereby allows Ire1 molecules to oligomerize in the plane of the ER membrane. Oligomerization, in turn, results in *trans*-autophosphorylation of the cytosolic kinase domain, providing the means by which the signal is transmitted across the ER membrane.

Activated Ire1 acts as a site-specific endoribonuclease, cleaving the mRNA encoding the transcription activator Hac1 at two discrete positions, and removing a 252-nucleotide nonclassical intron [8,9]. A second enzyme, tRNA ligase (Rlg1), then joins the severed exons to produce a spliced version of *HAC1 *mRNA, termed *HAC1*^*i *^mRNA (*i *for UPR-induced) [10]. The *HAC1 *mRNA splicing reaction mediated by Ire1 and Rlg1 is spliceosome-independent, and utilizes chemistry that closely resembles pre-tRNA splicing [9,11]. As in pre-tRNA processing, 5' and 3' splice-site cleavage of *HAC1 *mRNA occur independently. In contrast, spliceosome-mediated splicing is a series of two transesterification reactions that have to be strictly ordered: 3' splice-site cleavage cannot occur before 5' splice-site cleavage. Intriguingly, this Ire1-mediated splicing reaction happens on polyribosome-bound *HAC1 *mRNA in the cytosol [12,13].

In the unspliced *HAC1 *mRNA, the intron forms a long-range base-pairing interaction with the 5' untranslated region (UTR) that is responsible for preventing its translation [12,13]. Splicing abolishes translational inhibition, allowing production of the Hac1 transcription factor and induction of UPR target genes. Hence, removal of the intron provides a key regulatory step in the signaling pathway.

The amino-acid sequence of the nuclease region of Ire1 reveals significant similarities to that of RNase L, a mammalian endoribonuclease that is activated by interferon during viral infection [14,15]. RNase L functions to eliminate infected cells by nonspecific degradation of cellular RNA. Mutagenesis analysis maps the endoribonuclease activity of both proteins to homologous carboxy-terminal domains [16,17]. The nuclease domains in both proteins are preceded by kinase domains, and only the oligomerized forms of each protein appear to be active nucleases. Despite these similarities, the endoribonuclease activity of Ire1 is specific for the *HAC1 *mRNA, whereas the nuclease activity of RNase L shows no sequence specificity. Previously, to demonstrate the sequence specificity of Ire1, we devised an *in vitro *assay which utilized a purified, recombinant Ire1 protein [9]. This protein, here referred to as Ire1*, is composed of a linker region (bridging between the membrane anchor of full-length Ire1 and the cytosolic kinase domain), the kinase domain itself, and the RNase domain. Ire1* cleaves *HAC1 *mRNA faithfully at both 5' and 3' exon-intron junctions and thus recapitulates the substrate specificity of full-length Ire1 *in vivo*. Other RNAs, including actin mRNA and poly(U) RNA, which has been demonstrated to be an RNase L substrate [18], are not cleaved by Ire1* [9,16].

In metazoans, multiple parallel pathways originate from the ER and contribute to the UPR. Two *bona fide *Ire1 orthologs IRE1α and IRE1β [19,20] are present in higher eukaryotes. In addition, a Hac1 ortholog (XBP1) is activated by a similar nonconventional splicing step that changes the protein's carboxy-terminal sequence by introducing a frameshift [21-23]. Another transmembrane kinase, PERK, shares structural similarity to Ire1 in its ER-luminal unfolded-protein-sensing domain and is also activated upon accumulation of unfolded proteins [24,25]. Activated PERK phosphorylates the translation initiation factor eIF2α, thereby inactivating it. This branch of the pathway leads to a global repression of translation, which is thought to lessen the secretory burden on the ER. In cells with reduced eIF2α activity, mRNAs containing small open reading frames (ORFs) in their 5' UTRs become preferentially translated. One such mRNA codes for the transcription factor ATF4, which collaborates with XBP1 to induce UPR target genes [26]. A third branch of the metazoan UPR activates the transcription factor ATF6, which is initially synthesized as an ER transmembrane protein [27,28]. Accumulation of unfolded proteins allows ATF6 to leave the ER and move to the Golgi compartment, where it encounters proteases that release its cytosolic portion as a soluble protein [27,29-32], which participates along with XBP1 and ATF4 in executing the transcriptional program of the UPR [33].

To date, *HAC1 *mRNA is the only known RNA substrate for yeast Ire1, prompting the question of whether other substrate RNAs exist or whether Ire1 and *HAC1 *mRNA function as a matched enzyme-substrate pair that interact exclusively with each other. Here we describe three independent genome-scale methods that address this question. Each approach successfully identifies *HAC1 *mRNA as a substrate of Ire1; none of the approaches identifies any other mRNA as a qualified candidate. Two of these approaches represent novel applications of cDNA array technology and could be adapted to the study of other signal transduction pathways regulated by RNA processing.

## Results and discussion

**Figure 1:**
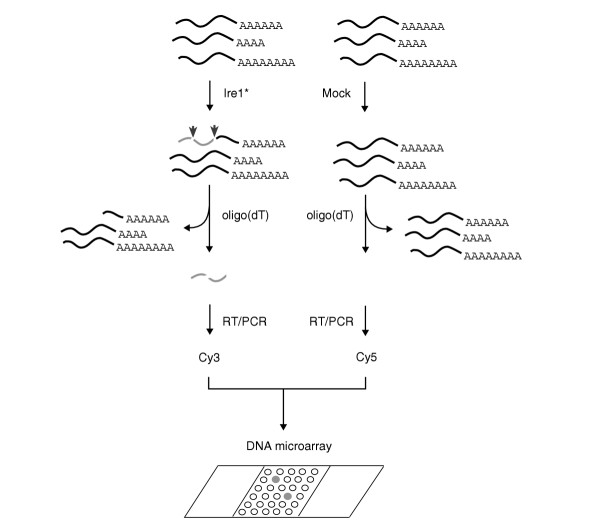
Schematic of screen for RNA substrates of Ire1p endoribonuclease using an *in vitro *nuclease reaction. Recombinant Ire1* expressed and purified from *E. coli *is incubated with poly(A)^+ ^RNA isolated from wild-type *S. cerevisiae *to cleave endogenous *HAC1 *mRNA and other potential RNA substrates of Ire1p. Cleaved RNA (lacking the poly(A) tail) is separated from uncleaved RNA as the unbound, poly(A)^- ^fraction from an oligo(dT) column and used to prepare fluorescent probe by reverse transcription followed by PCR amplification in the presence of Cy3-dTTP. A second control probe, using poly(A)^- ^RNA from mock nuclease reactions (identical reactions except for the lack of Ire1*) is prepared similarly, except that PCR amplification was carried out in the presence of Cy5-dTTP. Equal amounts of these probes are mixed and used to probe the yeast DNA microarray. Because RNA fragments generated by Ire1* cleavage are represented only in the Cy3 probe, microarray spots hybridizing to cleaved fragments should appear green, whereas microarray spots hybridizing to molecules common to both probes should appear yellow upon superimposition of green (Cy3) and red (Cy5) channels.

We first devised a molecular screen to identify mRNAs cleaved by the Ire1 nuclease (Figure 1). In brief, we isolated a total poly(A)^+ ^RNA fraction from cells and subjected it to *in vitro *cleavage reactions in the presence or absence of Ire1*. Fragments that lost their poly(A)^+ ^tails due to cleavage by Ire1* were re-isolated, reverse transcribed, fluorescently labeled and hybridized to genomic cDNA microarrays to identify Ire1 substrates.

For these experiments, we expressed and purified a recombinant Ire1* tagged with glutathione-*S*-transferase (GST). The GST moiety was removed during the purification by protease cleavage. Upon incubation of Ire1* with *in vitro *transcribed *HAC1 *mRNA, we observed efficient and accurate cleavage at both splice junctions as previously described [9,11].

**Figure 2:**
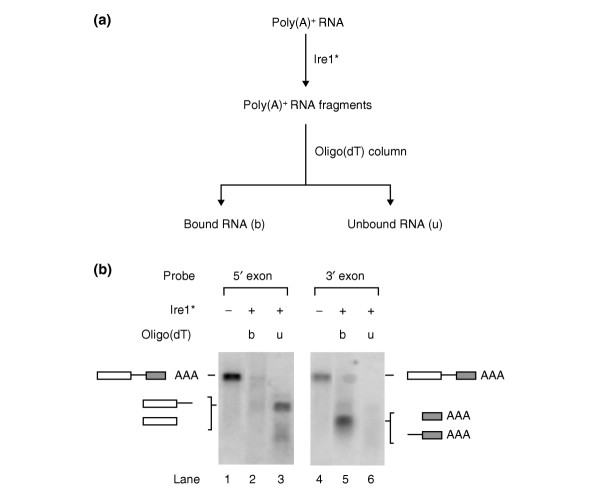
Efficient cleavage of *HAC1 *mRNA by Ire1* in the presence of cellular mRNA. **(a) **Schematic diagram of the Ire1*-cleaved (poly(A)^-^) and uncleaved (poly(A)^+^) RNA fractions separated after an *in vitro *nuclease reaction on yeast poly(A)^+ ^RNA. **(b) **Northern blot of the RNA fractions indicated in (a) probed with a PCR fragment encompassing either the 5' exon of *HAC1 *(lanes 1-3) or the 3' exon (lanes 4-6). Lanes 1 and 4, yeast poly(A)^+ ^RNA before Ire1* cleavage; lanes 2 and 5, bound uncleaved RNA fraction (b, poly(A)^+^); lanes 3 and 6, unbound cleaved RNA fraction (u, poly(A)^-^). Positions of uncleaved and cleaved *HAC1 *mRNA are indicated. Note that the 5' exon and 5' exon plus intron, and the 3' exon and 3' exon plus intron RNA species, respectively, co-migrate on these agarose gels.

We optimized reaction conditions such that *HAC1 *mRNA contained in a total poly(A)^+ ^RNA fraction from yeast would be efficiently cleaved, even in the presence of significant excess of other mRNAs. We incubated total poly(A)^+ ^RNA with Ire1* and then fractionated the products using oligo(dT) cellulose (Figure 2). Input RNA and the bound and unbound fractions of the cleavage reaction were analyzed by Northern blotting using a *HAC1*-specific probe covering the 5' exon (Figure 2, lanes 1-3). Note that *HAC1 *mRNA present in the input fraction (Figure 2, lane 1) was efficiently converted to a faster migrating species corresponding to the released 5' exon and intron, which, lacking poly(A) tails, were recovered exclusively in the unbound fraction after oligo(dT) chromatography (Figure 2, lane 3).

Conversely, analyzing the same RNA fractions with a *HAC1 *probe directed to the 3' exon, the resulting 3' exon (still containing its poly(A) tail) was recovered exclusively in the oligo(dT)-bound fraction (Figure 2, lane 5). No significant levels of uncleaved *HAC1 *mRNA were detectable after Ire1* cleavage (Figure 2, lanes 2, 3, 5 and 6).

**Figure 3:**
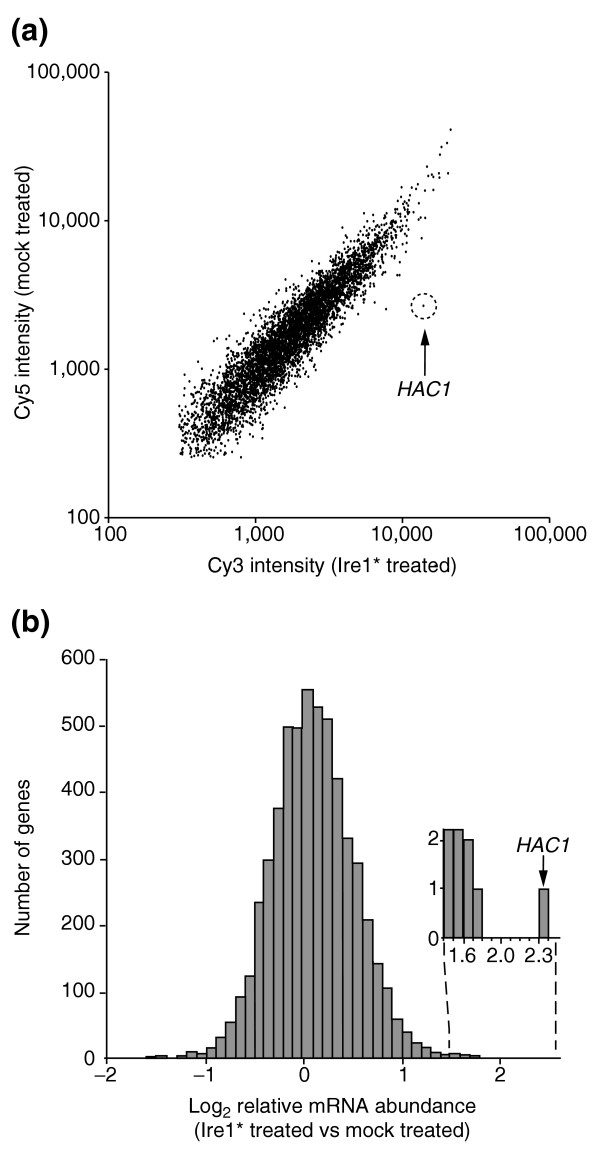
*HAC1 *is a unique RNA cleaved by Ire1*. **(a) **Scatter plot of Cy3 and Cy5 signal intensities following hybridization to a yeast ORF microarray with both Cy3- and Cy5-labeled probes, prepared from the cleaved RNA fragments generated from either Ire1* or mock treatment, respectively, as described in Figure 1. Each point on the plot represents a single yeast ORF. Points below the diagonal represent ORFs that hybridize predominantly to RNAs represented in the Cy3 probe. The position of the spot displaying the brightest Cy3 signals corresponds to *HAC1*. **(b) **Histogram representation of the log_2 _Cy3/Cy5 ratio. Inset, *HAC1 *is the only gene with a log_2 _Cy3/Cy5 ratio near 2.3. All data displayed in Figure 3 are provided in Additional data file 1.

Using these reaction conditions, we isolated Ire1*-cleaved RNA fragments from total poly(A)^+ ^RNA, which were recovered in the unbound fraction after oligo(dT) chromatography. We prepared a mock-treated reference sample using reaction conditions which were identical except that Ire1* was omitted. We reverse-transcribed both samples and fluorescently labeled the resulting cDNA with Cy3 (Ire1*-treated; green) or Cy5 (mock-treated; red), respectively. The two probes were then simultaneously hybridized to yeast microarrays. We expected that contaminating poly(A)^- ^RNA or uncleaved poly(A)^+ ^RNA would be equally represented in the oligo(dT) unbound fractions of both Ire1*-treated and mock-treated reactions, thus leading to equal representation of both fluorescent probes in the mixture. Indeed, scatter plots generated by quantitating the fluorescence levels of both Cy3 and Cy5 (Figure 3a) showed that most spots appeared on a tight diagonal, having hybridized to roughly equal amounts of both the Cy3 and Cy5 probes.

We expected those mRNAs that are specifically cleaved by Ire1* to be enriched in the oligo(dT) unbound fraction of the Ire1*-treated reaction compared to the unbound fraction of the mock-treated reaction, resulting in a higher Cy3/Cy5 fluorescence ratio in the microarray hybridization. Depletion of particular mRNAs by Ire1* digestion thus provides an enzymological tool to fractionate substrate from nonsubstrate mRNAs. We therefore plotted a histogram of the log_2 _Cy3/Cy5 ratio for all mRNAs (Figure 3b, and see Additional data file 1). The histogram approximates a tight normal distribution (mean = 0.06; σ = 0.41) with only one significant outlier: At a log_2 _Cy3/C5 ratio of 2.3, *HAC1 *mRNA falls 5.6σ from the mean of the distribution, clearly identifying this mRNA as an Ire1* substrate. Indeed, under these conditions, *HAC1 *mRNA is the only substrate for cleavage by Ire1* represented in the poly(A)^+ ^RNA fraction. As the Ire1 cleavage sites on other mRNAs could, in principle, be located within the 5' or 3' UTRs, we also hybridized the same probe to genomic microarrays containing yeast intergenic regions in addition to the ORFs. Our results were similar to those observed using ORF-only microarrays, with *HAC1 *being the only significant outlier (data not shown).

**Figure 4:**
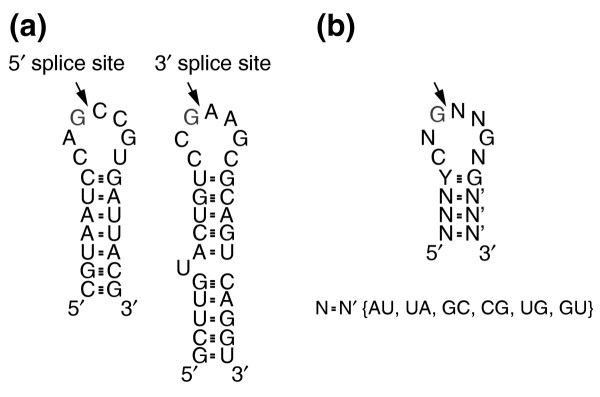
*HAC1 *stem-loop motif used for the genome wide computational consensus search. **(a) **Predicted secondary structure and sequence flanking both 5' and 3' Ire1p cleavage sites in *HAC1 *mRNA. Cleavage occurs after a G residue located at the third position of the seven-nucleotide loop. **(b) **Parameters used for the computer search. A four base-pair stem with a seven-nucleotide loop with C at the first position, and G both at third and sixth positions, are determined experimentally as described previously [14]. Possible base-pairs (N = N') in the stem include AU, UA, GC, CG, UG, GU. The complete output of the computational screen is listed in Additional data file 2.

To exclude the possibility that other potential RNA substrates might be hiding in the scatter of the data, our second approach combined the microarray analysis with a bioinformatics approach to search the genome for potential Ire1 cleavage sites. We had previously defined a consensus stem-loop motif by comparing the two splice sites of *HAC1 *mRNA and mutational analysis (Figure 4a) [11]. Both cleavages occur between the third and fourth nucleotide of a predicted seven-nucleotide loop bounded by a stem; the first nucleotide of both loops is a C; the third and sixth nucleotide of both loops is a G; and the first nucleotide of the 3' leg of the stem is in both cases a G. The primary and secondary sequence information in this consensus is illustrated in Figure 4b. *In vitro*, Ire1* can cleave short RNA substrates containing only the 5' or 3' stem-loop sequences [11]. Mutational studies at nucleotide resolution demonstrate that each of the shared primary and secondary sequence elements is essential for efficient cleavage.

We computationally searched the yeast genome for the presence of sequences fitting the consensus stem-loop motif. In this analysis, we searched ORFs, as well as 1,000 nucleotides upstream and downstream to include potential stem-loop motifs in 5' and 3' UTRs. The search yielded a total of 52 hits (Additional data file 2). In the list of genes identified, only a single gene, *HAC1*, contains two predicted stem-loop structures, consistent with the notion that *HAC1 *mRNA is the only Ire1-dependent splicing substrate in yeast cells.

For the remaining 52 predicted stem-loop structures, the possibility remained open that Ire1 would cleave some substrates at only a single site, perhaps to downregulate particular mRNAs. However, in the light of the data obtained from the microarray analysis described in Figure 3 we consider this possibility unlikely. We would have expected to see the 5' fragments resulting from such cleavage events in the oligo(dT) unbound fraction of Ire1*-treated poly(A)^- ^mRNA, resulting in high outlying Cy3/Cy5 ratios; however, none of the genes predicted to bear single *HAC1 *splice consensus sequences fell more than 3σ from the mean of the distribution (that is, none fell outside a 99% confidence interval). Because previous *in vitro *analyses have shown that stem-loop structures matching the consensus are sufficient for cleavage, we consider it most likely that the predicted stem-loop structures are either not included in the transcripts or do not fold as predicted in the context of the full mRNA sequences.

In a third genome-scale approach, we exploited the phenotype of a mutant in tRNA ligase that is defective in UPR induction [10]. Characterization of this mutant, *rlg1-100*, previously showed that Ire1-dependent cleavage of *HAC1 *mRNA occurs normally in the absence of ligation, but that the cleavage products are rapidly degraded. We assume that any other mRNA following this pathway should suffer the same fate, and that we could identify substrates of Ire1 by looking for mRNAs whose steady-state levels drop upon UPR induction in the *rlg1-100 *mutant, but not in a wild-type cell. The Ire1-dependent selective disappearance of *HAC1 *mRNA in *rlg1-100 *cells thus provides us with a tool to assess the spectrum of mRNAs that utilize the Ire1-mediated splicing pathway.

**Figure 5:**
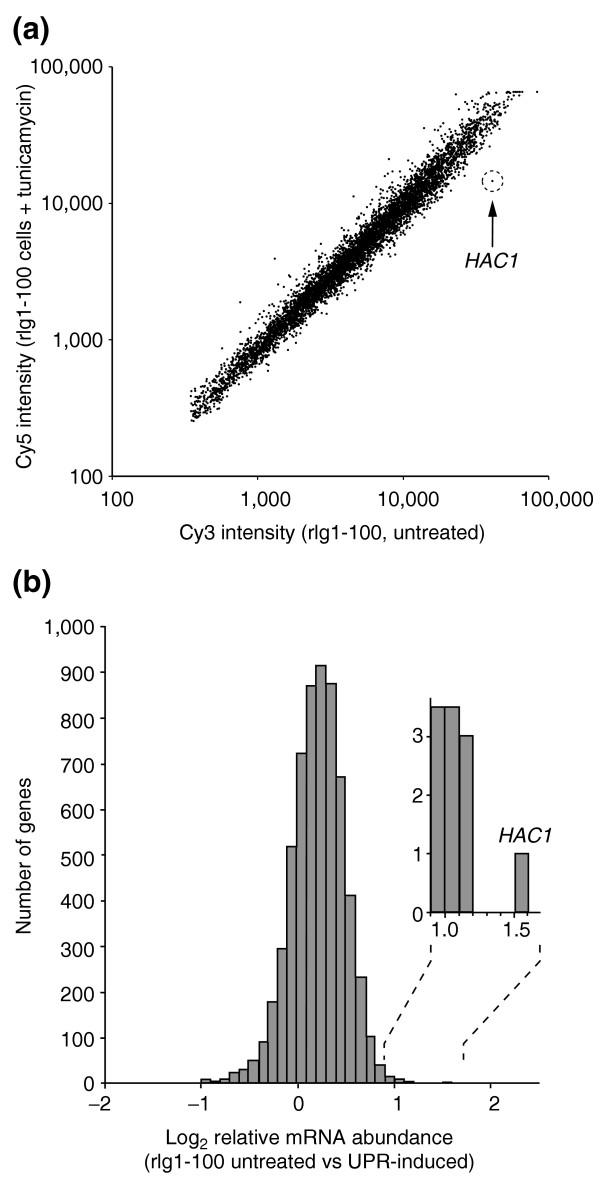
*HAC1 *is the only transcript decreasing in *rlg1-100 *cells following tunicamycin treatment. **(a) **Scatter plot of Cy3 and Cy5 signal intensities following hybridization to a yeast ORF microarray with both Cy3- and Cy5-labeled probes, prepared from RNA isolated from *rlg1-100 *cells either untreated or treated with tunicamycin for 40 min, respectively. Points below the diagonal represent ORFs that hybridize predominantly to RNAs represented in the Cy3 probe, and which are therefore present at reduced levels upon tunicamycin treatment. The position of the spot displaying the brightest Cy3 signals (corresponding to the *HAC1*) is shown. **(b) **Histogram presentation of microarray data measuring log_2 _of the relative mRNA abundance between tunicamycin treated (40 min) and untreated *rlg1-100 *cells. Levels of most mRNAs were not altered, and the only mRNA significantly changed upon tunicamycin treatment is *HAC1*. Inset, *HAC1 *is the only gene with a log_2 _fold-change in abundance near 1.5. All data displayed in Figure 5 are provided in Additional data file 3.

We treated *rlg1-100 *cells with either tunicamycin (to induce the UPR by inhibition of *N*-linked glycosylation in the ER) or with no drug as a control, isolated total mRNA, reverse transcribed and fluorescently labeled the cDNA with Cy5 (for the tunicamycin-treated sample) and Cy3 (for the untreated sample) before simultaneous hybridization to genomic microarrays. mRNAs which are depleted during tunicamycin treatment should have Cy3/Cy5 ratios greater than 1 (log_2 _Cy3/Cy5 ratios greater than 0). As shown by the data in Figure 5, the steady-state levels of most mRNAs remain unchanged upon tunicamycin treatment (Figure 5a, and Additional data file 3). As before, a histogram of the log_2 _Cy3/Cy5 ratios followed a tight quasi-normal distribution (mean = 0.11, σ = 0.27) with a single outlier. *HAC1*, at a log_2 _Cy3/5 ratio of 1.5, *HAC1 *falls 5σ from the mean, and is thus successfully identified as a splicing target of the UPR. Once again, no other mRNA clearly satisfied the criteria for identification as an Ire1 substrate. The depletion of *HAC1 *mRNA upon tunicamycin treatment was specific to *rlg1-100 *cells. A similar analysis of wild-type cells showed the expected induction of UPR target genes and no depletion of *HAC1 *mRNA relative to an untreated control (data not shown). Thus, *HAC1 *mRNA again stands out as the unique substrate for Ire1-dependent cleavage in yeast.

## Conclusions

The experiments presented in this paper represent three genome-scale approaches for identifying mRNA substrates of the Ire1-dependent mRNA splicing pathway in yeast. Each approach successfully and selectively identifies the *HAC1 *mRNA as a target of Ire1 nuclease. *In vitro *cleavage of mRNAs by Ire1*, followed by microarray detection of the fractionated mRNAs, identifies *HAC1 *mRNA as significantly enriched in the population of mRNAs specifically cleaved by Ire1. A computational search for the experimentally determined Ire1 consensus cleavage sites identifies *HAC1 *as the unique gene containing two such sequences. Finally, *in vivo *induction of splicing in a cell containing wild-type tRNA ligase or the mutant *rlg1-100*, and subsequent microarray detection of 'genetically fractionated' mRNAs, identifies *HAC1 *as selectively depleted in the absence of the wild-type ligase. No other mRNA met any of these criteria for identification as an Ire1 substrate. We consider it reasonable to conclude that among the set of robustly expressed genes, *HAC1 *mRNA is the lone substrate of Ire1.

In principle, there are many reasons why each of the approaches presented here could have missed identifying an Ire1 substrate other than *HAC1 *mRNA. For example, a poorly expressed substrate would exhibit a low signal-to-noise ratio in the microarray readout of the *in vitro *cleavage assay, or a substrate cleaved close to the 5' end would still hybridize to cDNA arrays with an efficiency comparable to that of the uncleaved mRNA, and hence could have escaped detection. In our computational screen, we might have missed cleavage sites that are significantly divergent from the experimentally defined consensus. Finally, because tRNA ligase would not take part in a cleavage-only reaction on a single-site substrate, we would also not expect our third method to lead to a relative reduction of the abundance of such mRNAs in *rlg1-100 *mutant cells as compared to the wild-type (though we would have expected such substrates to be identified in the *in vitro *Ire1* cleavage experiment). Thus rigorously, we cannot conclude that *HAC1 *mRNA is the only Ire1 substrate. However, because the potential caveats are noncongruent and because *HAC1 *stands out unambiguously in each of the three methods applied, we consider it highly unlikely that additional substrates exist.

Previous studies in metazoans have suggested that other RNAs are degraded when Ire1 is activated [17,34]. When overexpressed, wild-type IRE1α mRNA accumulated at levels that were greatly reduced compared to those in which an RNase-dead mutant was overexpressed, raising the possibility that Ire1 might downregulate its own mRNA by degradation. Similarly, overexpression of IRE1β appeared to cause fragmentation of 28S rRNA. In both cases, however, no Ire1 cleavage consensus stem-loop structures were found. It therefore remains questionable at this time whether these degradation events are directly due to cleavage by Ire1 itself or to indirect secondary effects.

According to the current evidence, therefore, Ire1 has a single identified target in yeast and thus functions solely for the purpose of post-transcriptional regulation of *HAC1*. In contrast, other signaling pathways in the cell are commonly branched, using one enzyme to activate multiple different substrates. The utilization of common principles of protein modification, such as phosphorylation or ubiquitination, readily allows cross-talk among different signaling pathways that use common components. One phosphatase, for example, can dephosphorylate substrates that have been phosphorylated by several different kinases. In contrast, communication between the ER and nucleus through Ire1 appears to be a very private affair. Ire1-dependent splicing offers unique mechanistic advantages, such as the ability to quickly complete the synthesis of partially made Hac1 transcription factor by releasing the translational arrest of stalled polyribosomes. Given that the ER is a topologically distinct compartment of the cell with metabolic considerations quite distinct from those of the cytosol, a communication route that is wired differently and therefore more insulated from other informational 'chatter' in the cell may be particularly beneficial in the secure passage of information from the ER to the nucleus.

The linear connectivity (activation of Ire1 → production of Hac1) of the two key players of the yeast UPR is supported by genetic analysis. Both *Δire1 *and *Δhac1 *mutant cells display indistinguishable growth phenotypes and highly correlated gene-expression profiles [4]. In higher eukaryotes, signaling through the UPR is more complex, with multiple ER-proximal components each activating distinct downstream targets. Metazoan Ire1 exists as two isoforms (Ire1α and Ire1β) [20,35]; in addition, the transmembrane kinase PERK is activated and the membrane-tethered transcription factors ATF6α and ATF6β are released when unfolded proteins accumulate in the ER [27,36]. The diversity of these signaling components varies widely between different tissues, and the relative contribution of each of the parallel pathways to the induction of the downstream transcriptional program is currently the subject of intense study. Given this increased complexity, it is possible that there are other metazoan Ire1 splicing substrates in addition to the *HAC1 *ortholog XBP1, and the methods developed here may prove useful in searching for such putative, additional Ire1 substrates in metazoans.

## Materials and methods

### RNA isolation

RNA samples were prepared as described previously [6]. Briefly, yeast cells were grown to mid-log phase in selective media. *rlg1-100 *cells were treated with 1 μg/ml tunicamycin (Calbiochem) for 40 min in the experiments described in Figure 5. Total RNA was isolated in SDS-high salt buffer by hot phenol extraction. For the microarray experiments described in Figures 3 and 5, poly(A)^+ ^RNA was isolated from total RNA using the PolyA^+ ^Tract mRNA isolation system (Promega) according to the manufacturer's instructions. Poly(A)^+ ^RNA used for *in vitro *Ire1* nuclease reactions was prepared by two rounds of purification using the PolyA^+ ^Tract system.

### Northern blot analysis

Total cellular RNA and RNA recovered from *in vitro *nuclease reactions was analyzed by Northern blot hybridization as described previously [8] by separation on 1.5% agarose gels containing 6.7% formaldehyde. Hybridization probes were generated by random labeling of PCR fragments using [^32^P]α-dCTP according to the manufacturer's instructions (Amersham). All probes prepared from DNA fragments were generated by PCR amplification of yeast genomic DNA.

### *In vitro *Ire1* nuclease reaction

*In vitro *nuclease reactions were performed by incubating yeast poly(A)^+ ^RNA with recombinant Ire1* (expressed and purified from *Escherichia coli*) as described previously [9,11]. Following phenol/chloroform extraction, the reaction mixture was fractionated on oligo(dT) magnetic beads using the PolyA^+ ^Tract mRNA Isolation System (Promega) according to the manufacturer's instructions. Control nuclease reactions were performed and processed identically, except that Ire1* was omitted from the reaction mixture.

### Microarray hybridization of amino-allyl coupled cDNA probe

General protocols for microarray hybridization and for preparation of probe were as described previously [37]. A sample of oligo(dT) unbound RNA was reverse transcribed with StrataScript (Stratagene) using random primer A (5' GGTTCCCAGTCACGATCNNNNNNNNN 3', where N is any nucleotide) followed by addition of Sequenase to synthesize the second strand. The resulting double-stranded cDNA above was amplified in the polymerase chain reaction (PCR) (20 cycles) using primer B (5' GGTTCCCAGTCACGATC 3') complimentary to the specific sequence portion of primer A. This extra PCR amplification step was added because of the low abundance of Ire1*-cleaved RNA fragments. After ethanol precipitation, one fourth of these PCR reactions was used to re-PCR in the presence of fluorescently labeled nucleotides (either Cy3 or Cy5 dTTP). The resulting Cy3- and Cy5-labeled probes were combined. Unreacted fluorescent dye was quenched. Probes were cleaned up in a QIA-quick PCR purification spin column and hybridized at 50°C for 24 h to glass slide microarrays containing the entire yeast genome. Synthesis of the cDNA for poly(A)^+ ^RNA (2 μg) used for the experiment in Figure 5 was carried out by reverse transcription in the presence of aminoallyl-dUTP at 42°C for 2 h. cDNA prepared from untreated cells was coupled with Cy3, and cDNA prepared from tunicamycin-treated cells was labeled with Cy5.

### Data analysis

Hybridized microarrays were scanned with a GenePix 4000A microarray scanner (Axon Instruments). GenePix Pro was used to analyze and to display the data. All data points with absolute fluorescence intensity less than 250 in either channel were discarded. Cy3 and Cy5 fluorescence intensities were normalized against one another to adjust for differences in labeling efficiency and scanner gain. Quantitative analysis was performed in Microsoft Excel.

### Stem-loop consensus search

Pattern searching was performed using the public-domain software scan_for_matches [38]. Sequence file input was a database of all yeast ORFs ± 1,000 nucleotides obtained from the *Saccharomyces *Genome Database [39]. Pattern file input was

r1 = {au,ua,gc,cg,ug,gu}

RNA base-pairing rules were used; allowed base-pairs are the fields of argument r1.

p1 = 4..4 YCNGNNGNG ~p1

These parameters instructed the program to use RNA base-pairing rules to find a sequence of four nucleotides (one arm of stem), followed by a sequence matching YCNGNNGNG (where Y is pyrimidine; the seven-nucleotide loop flanked the conserved closing nucleotide pair), followed by the reverse complement of the four nucleotides at the beginning (the second arm of the stem). The output of the computational screen is listed in Additional data file 2.

## Additional data files

The following additional data are available with the online version of this paper. Additional data file 1 contains supplementary Table 1 which lists all the data displayed in Figure 3; Additional data file 2 contains supplementary Table 2, which lists the complete output of the computational screen; Additional data file 3 contains supplementary Table 3 which lists all data displayed in Figure 5. Additional data file 4 is a Word file containing the captions and keys to the tables.

## Supplementary Material

Additional data file 1 Supplementary Table 1 which lists all the data displayed in Figure 3 Click here for additional data file

Additional data file 2 Supplementary Table 2, which lists the complete output of the computational screen Click here for additional data file

Additional data file 3 Supplementary Table 3 which lists all data displayed in Figure 5 Click here for additional data file

Additional data file 4 The captions and keys to the supplementary tables Click here for additional data file
