# Supplementary material for: Genome-scale approaches for discovering novel nonconventional splicing substrates of the Ire1 nuclease
Source: Genome Biol. 2004 Dec 22;6(1):R3. doi: 10.1186/gb-2004-6-1-r3 (PMC549064; doi:10.1186/gb-2004-6-1-r3)
Supplement: Additional data file 1 — Supplementary Table 1 which lists all the data displayed in Figure 3 [file gb-2004-6-1-r3-s1.pdf]

Niwa et al.

Supplementary Table 1

ID	Name	F532=X	F635=Y	(X/Y)	log2(X/Y)	(/Y)-mean(log2(X/Y))	Nsigma
YFL031W		13891	2670	5.20	2.38	2.32	5.61
YJR112W	NNF1	1116	342	3.26	1.71	1.65	3.99
YER150W	SPI1	798	254	3.14	1.65	1.60	3.85
YPR186C	PZF1	7752	2528	3.07	1.62	1.56	3.77
YNL304W	YPT11	1581	549	2.88	1.53	1.47	3.55
YGL208W	SIP2	1125	399	2.82	1.50	1.44	3.48
YLR298C	YHC1	1109	394	2.82	1.49	1.44	3.47
YJR107W		964	350	2.75	1.46	1.41	3.39
YPR005C	HAL1	1122	421	2.67	1.41	1.36	3.28
YOR122C	PFY1	2574	986	2.61	1.38	1.33	3.21
YHL028W	WSC4	2290	889	2.58	1.36	1.31	3.16
YKL007W	CAP1	918	364	2.52	1.33	1.28	3.09
YJR162C		1911	760	2.51	1.33	1.27	3.08
YPL067C		694	276	2.51	1.33	1.27	3.08
YOR312C	RPL20B	1080	436	2.48	1.31	1.25	3.02
YHL018W		1325	538	2.46	1.30	1.24	3.00
YGR292W	MAL12	2503	1039	2.41	1.27	1.21	2.93
YNL130C	CPT1	757	316	2.39	1.26	1.20	2.91
YJL144W		1399	585	2.39	1.26	1.20	2.90
YHR150W		1206	505	2.39	1.26	1.20	2.90
YFL045C	SEC53	2536	1070	2.37	1.24	1.19	2.87
YPR006C	ICL2	1173	498	2.36	1.24	1.18	2.85
YKR038C		2172	927	2.34	1.23	1.17	2.83
YOR078W		1856	795	2.33	1.22	1.17	2.82
YMR041C		628	271	2.32	1.21	1.16	2.79
YOR091W		6474	2802	2.31	1.21	1.15	2.78
YPL224C	MMT2	614	266	2.31	1.21	1.15	2.78
YMR296C	LCB1	2210	959	2.30	1.20	1.15	2.77
YPL068C		615	267	2.30	1.20	1.15	2.77
YOL053W		2021	888	2.28	1.19	1.13	2.73

YJR146W		622	274	2.27	1.18	1.13	2.72
YJR063W	RPA12	953	420	2.27	1.18	1.13	2.72
YLR254C		1000	441	2.27	1.18	1.13	2.72
YDL244W	THI13	1062	470	2.26	1.18	1.12	2.70
YHL014C	YLF2	787	349	2.26	1.17	1.12	2.70
YJR060W	CBF1	751	336	2.23	1.16	1.10	2.67
YJL111W	CCT7	9550	4295	2.22	1.15	1.10	2.65
YKR068C	BET3	1353	612	2.21	1.14	1.09	2.63
YMR236W	TAF17	937	425	2.21	1.14	1.08	2.62
YOR319W	HSH49	740	336	2.20	1.14	1.08	2.61
YLR313C	SPH1	1162	530	2.19	1.13	1.08	2.60
YNL230C	ELA1	1074	494	2.17	1.12	1.06	2.57
YDL023C		994	458	2.17	1.12	1.06	2.56
YML021C	UNG1	1928	891	2.16	1.11	1.06	2.55
YLL002W	KIM2	964	446	2.16	1.11	1.06	2.55
YJL060W		1625	752	2.16	1.11	1.06	2.55
YLR029C	RPL15A	9235	4292	2.15	1.11	1.05	2.53
YAL049C		1964	915	2.15	1.10	1.05	2.53
YDR284C	DPP1	4995	2338	2.14	1.10	1.04	2.51
YLL065W	GIN11	1470	689	2.13	1.09	1.04	2.50
YFL063W		2410	1136	2.12	1.08	1.03	2.48
YKL225W		1265	597	2.12	1.08	1.03	2.48
YHR081W		2797	1321	2.12	1.08	1.03	2.48
YOR076C		915	433	2.11	1.08	1.02	2.47
YIL136W	OM45	777	368	2.11	1.08	1.02	2.47
YER175C		1139	541	2.11	1.07	1.02	2.46
YNL338W		3733	1775	2.10	1.07	1.02	2.45
YER159C	BUR6	2119	1008	2.10	1.07	1.02	2.45
YBR032W		792	379	2.09	1.06	1.01	2.43
YHR158C	KEL1	1097	528	2.08	1.05	1.00	2.41
YOR236W	DFR1	1863	897	2.08	1.05	1.00	2.41
YDL230W	PTP1	1154	556	2.08	1.05	1.00	2.41
YOR106W	VAM3	1517	732	2.07	1.05	1.00	2.40
YJR094W-A	RPL43B	1510	729	2.07	1.05	0.99	2.40
YNL074C	MLF3	1193	576	2.07	1.05	0.99	2.40
YER116C		1557	753	2.07	1.05	0.99	2.40

YHL002W		1561	755	2.07	1.05	0.99	2.39
YNL240C	NAR1	581	282	2.06	1.04	0.99	2.38
YDL070W	BDF2	2436	1186	2.05	1.04	0.98	2.37
YJL194W	CDC6	621	304	2.04	1.03	0.97	2.35
YLR301W		2345	1148	2.04	1.03	0.97	2.35
YJL108C		553	271	2.04	1.03	0.97	2.35
YLR041W		736	363	2.03	1.02	0.96	2.33
YIL154C	IMP2'	517	255	2.03	1.02	0.96	2.33
YLR231C		1642	810	2.03	1.02	0.96	2.33
YDR051C		1992	984	2.02	1.02	0.96	2.32
YER056C-A	RPL34A	946	468	2.02	1.01	0.96	2.32
YNL100W		1917	949	2.02	1.01	0.96	2.31
YBL080C	PET112	1688	836	2.02	1.01	0.96	2.31
YIL172C		1093	542	2.02	1.01	0.96	2.31
YOR298C-A	YOR298C-A	1189	590	2.02	1.01	0.95	2.31
YKR026C	GCN3	823	409	2.01	1.01	0.95	2.30
YHL016C	DUR3	1920	954	2.01	1.01	0.95	2.30
YMR240C	CUS1	3308	1644	2.01	1.01	0.95	2.30
YHL031C	GOS1	1343	668	2.01	1.01	0.95	2.30
YHR013C	ARD1	1980	990	2.00	1.00	0.94	2.28
YOL052C	SPE2	3027	1514	2.00	1.00	0.94	2.28
YDR274C		880	441	2.00	1.00	0.94	2.27
YGR294W		821	412	1.99	0.99	0.94	2.27
YPL111W	CAR1	1464	737	1.99	0.99	0.93	2.26
YLR126C		654	331	1.98	0.98	0.93	2.24
YLR353W	BUD8	809	411	1.97	0.98	0.92	2.22
YNL241C	ZWF1	1770	902	1.96	0.97	0.92	2.21
YFR023W	PES4	918	468	1.96	0.97	0.92	2.21
YOR052C		1003	513	1.95	0.97	0.91	2.20
YLR374C		1304	668	1.95	0.97	0.91	2.20
YML008C	ERG6	3941	2020	1.95	0.96	0.91	2.19
YDL091C		815	418	1.95	0.96	0.91	2.19
YNL057W		866	445	1.95	0.96	0.90	2.18
YML064C	TEM1	816	421	1.94	0.95	0.90	2.17
YHL017W		2758	1424	1.94	0.95	0.90	2.17
YPR078C		5448	2813	1.94	0.95	0.90	2.17

YDR075W	PPH3	3237	1673	1.93	0.95	0.90	2.16
YAR031W		928	480	1.93	0.95	0.89	2.16
YGR286C	BIO2	1920	994	1.93	0.95	0.89	2.16
YJR044C		1675	867	1.93	0.95	0.89	2.16
YBL109W		3844	1993	1.93	0.95	0.89	2.15
YLR317W		739	384	1.92	0.94	0.89	2.14
YOL038W	PRE6	2809	1463	1.92	0.94	0.89	2.14
YMRWdelta16	YMRWdelta16	495	258	1.92	0.94	0.88	2.14
YNL159C		1359	708	1.92	0.94	0.88	2.14
YBR061C		1089	569	1.91	0.94	0.88	2.12
YKR100C		1539	806	1.91	0.93	0.88	2.12
YDL218W		1487	779	1.91	0.93	0.88	2.12
YER100W	UBC6	2018	1059	1.91	0.93	0.87	2.11
YLR427W		1484	779	1.91	0.93	0.87	2.11
YMR271C	URA10	2614	1372	1.91	0.93	0.87	2.11
YMR203W	TOM40	1061	557	1.90	0.93	0.87	2.11
YLR358C		2109	1110	1.90	0.93	0.87	2.10
YNL142W	MEP2	961	506	1.90	0.93	0.87	2.10
YNL236W	SIN4	1101	580	1.90	0.92	0.87	2.10
YMR042W	ARG80	1276	673	1.90	0.92	0.87	2.09
YHR142W	CHS7	1131	597	1.89	0.92	0.87	2.09
YGL160W		2881	1522	1.89	0.92	0.86	2.09
YPL229W		1546	818	1.89	0.92	0.86	2.08
YEL016C		1483	785	1.89	0.92	0.86	2.08
YER161C	SPT2	5518	2937	1.88	0.91	0.85	2.06
YDR044W	HEM13	712	379	1.88	0.91	0.85	2.06
YDR020C		591	315	1.88	0.91	0.85	2.06
YNL063W		2764	1474	1.88	0.91	0.85	2.06
YFR028C	CDC14	2686	1433	1.87	0.91	0.85	2.05
YMR010W		1690	902	1.87	0.91	0.85	2.05
YIL108W		1700	911	1.87	0.90	0.84	2.04
YJL097W		1566	841	1.86	0.90	0.84	2.03
YER024W		1223	657	1.86	0.90	0.84	2.03
YHL032C	GUT1	1394	750	1.86	0.89	0.84	2.02
YGL158W	RCK1	526	283	1.86	0.89	0.84	2.02
YHR175W	CTR2	832	448	1.86	0.89	0.84	2.02

YER177W	BMH1	4066	2195	1.85	0.89	0.83	2.01
YML019W	OST6	1527	826	1.85	0.89	0.83	2.00
YKR105C		584	316	1.85	0.89	0.83	2.00
YCR056W		2262	1225	1.85	0.88	0.83	2.00
YPL254W	HFI1	1355	734	1.85	0.88	0.83	2.00
YHL033C	RPL8A	5422	2944	1.84	0.88	0.82	1.99
YHR012W	VPS29	3153	1712	1.84	0.88	0.82	1.99
YKL154W	SRP102	984	535	1.84	0.88	0.82	1.99
YOL065C	INP54	983	535	1.84	0.88	0.82	1.98
YGR287C		786	428	1.84	0.88	0.82	1.98
YEL062W	NPR2	1179	643	1.83	0.88	0.82	1.98
YLR264W	RPS28B	9596	5238	1.83	0.87	0.82	1.97
YJL145W		1254	685	1.83	0.87	0.82	1.97
YLR125W		762	416	1.83	0.87	0.82	1.97
YGR280C		2301	1260	1.83	0.87	0.81	1.96
YLR297W		1022	561	1.82	0.87	0.81	1.95
YKR012C		1488	818	1.82	0.86	0.81	1.95
YOR377W	ATF1	2278	1253	1.82	0.86	0.81	1.95
YKL124W	SSH4	932	513	1.82	0.86	0.81	1.95
YLR369W	SSQ1	539	297	1.81	0.86	0.80	1.94
YHL046C		710	392	1.81	0.86	0.80	1.93
YOL062C	APM4	2919	1613	1.81	0.86	0.80	1.93
YMR208W	ERG12	2707	1497	1.81	0.85	0.80	1.93
YNL053W	MSG5	484	268	1.81	0.85	0.80	1.93
YMR255W	GFD1	728	403	1.81	0.85	0.80	1.92
YPL112C		1379	765	1.80	0.85	0.79	1.92
YPR196W		1135	630	1.80	0.85	0.79	1.91
YOL069W	NUF2	1127	626	1.80	0.85	0.79	1.91
YDL234C	GYP7	1699	944	1.80	0.85	0.79	1.91
YJL172W	CPS1	1782	991	1.80	0.85	0.79	1.91
YCLX09W		1521	846	1.80	0.85	0.79	1.91
YCR022C		1529	851	1.80	0.85	0.79	1.91
YIL152W		487	271	1.80	0.84	0.79	1.90
YOL031C		1877	1046	1.79	0.84	0.79	1.90
YLR090W	XDJ1	2338	1307	1.79	0.84	0.78	1.89
YNL085W	MKT1	3434	1920	1.79	0.84	0.78	1.89

YIL124W	AYR1	785	439	1.79	0.84	0.78	1.89
YJL208C	NUC1	2603	1458	1.79	0.84	0.78	1.88
YJL113W		1081	606	1.78	0.84	0.78	1.88
YNL131W	TOM22	1570	881	1.78	0.83	0.78	1.88
YOL059W	GPD2	1945	1091	1.78	0.83	0.78	1.88
YIL135C		2000	1124	1.78	0.83	0.78	1.87
YLR429W	CRN1	4573	2572	1.78	0.83	0.77	1.87
YER130C		1311	738	1.78	0.83	0.77	1.87
YOR047C	STD1	1965	1106	1.78	0.83	0.77	1.87
YER145C	FTR1	3349	1886	1.78	0.83	0.77	1.87
YGL157W		1683	948	1.78	0.83	0.77	1.86
YIL089W		708	399	1.78	0.83	0.77	1.86
YMR085W		13515	7614	1.78	0.83	0.77	1.86
YBR249C	ARO4	6411	3619	1.77	0.83	0.77	1.86
YNL288W		1339	756	1.77	0.82	0.77	1.86
YDR353W	TRR1	3917	2214	1.77	0.82	0.77	1.85
YHR160C	PEX18	1608	909	1.77	0.82	0.77	1.85
YJL140W	RPB4	914	517	1.77	0.82	0.77	1.85
YPL244C		1402	793	1.77	0.82	0.77	1.85
YDR290W		684	387	1.77	0.82	0.77	1.85
YDR415C		958	542	1.77	0.82	0.77	1.85
YGR278W		1489	844	1.76	0.82	0.76	1.84
YBR016W		1606	910	1.76	0.82	0.76	1.84
YFR039C		1962	1114	1.76	0.82	0.76	1.84
YPL245W		1629	925	1.76	0.82	0.76	1.84
YKR070W		822	468	1.76	0.81	0.76	1.83
YGL192W	IME4	1267	721	1.76	0.81	0.76	1.83
YLR257W		2628	1496	1.76	0.81	0.76	1.83
YER162C	RAD4	2976	1696	1.75	0.81	0.76	1.82
YOR389W		1590	906	1.75	0.81	0.76	1.82
YNL073W	MSK1	500	285	1.75	0.81	0.76	1.82
YMR070W	MOT3	2806	1599	1.75	0.81	0.75	1.82
YKR043C		3228	1840	1.75	0.81	0.75	1.82
YGL044C	RNA15	2377	1355	1.75	0.81	0.75	1.82
YDR036C		1068	610	1.75	0.81	0.75	1.82
YDR414C	ERD1	2061	1178	1.75	0.81	0.75	1.81

YMR053C	STB2	1166	668	1.75	0.80	0.75	1.81
YPR187W	RPO26	935	536	1.74	0.80	0.75	1.80
YBL006C		4504	2583	1.74	0.80	0.75	1.80
YPR081C		3360	1927	1.74	0.80	0.75	1.80
YBR067C	TIP1	2033	1166	1.74	0.80	0.75	1.80
YKR091W	SRL3	2372	1362	1.74	0.80	0.74	1.80
YOL033W	MSE1	644	370	1.74	0.80	0.74	1.80
YDR496C		3019	1735	1.74	0.80	0.74	1.79
YEL058W	PCM1	2613	1503	1.74	0.80	0.74	1.79
YCR024C		1058	609	1.74	0.80	0.74	1.79
YFR055W		1859	1070	1.74	0.80	0.74	1.79
YLR253W		1942	1119	1.74	0.80	0.74	1.79
YNR015W	SMM1	1543	889	1.74	0.80	0.74	1.78
YPL117C	IDI1	932	538	1.73	0.79	0.74	1.78
YMR224C	MRE11	2265	1308	1.73	0.79	0.74	1.78
YKL002W		734	424	1.73	0.79	0.74	1.78
YPL212C	PUS1	1504	869	1.73	0.79	0.74	1.78
YIL104C		2063	1193	1.73	0.79	0.73	1.77
YLR440C		989	572	1.73	0.79	0.73	1.77
YJL139C	YUR1	1809	1047	1.73	0.79	0.73	1.77
YGL156W	AMS1	846	490	1.73	0.79	0.73	1.77
YOL020W	TAT2	1442	835	1.73	0.79	0.73	1.77
YHR217C		5583	3236	1.73	0.79	0.73	1.76
YDL229W	SSB1	650	377	1.72	0.79	0.73	1.76
YMR023C	MSS1	1070	621	1.72	0.79	0.73	1.76
YOR380W		1067	619	1.72	0.79	0.73	1.76
YNL290W	RFC3	1833	1064	1.72	0.78	0.73	1.76
YGL174W		981	570	1.72	0.78	0.73	1.76
YNL004W	HRB1	4583	2668	1.72	0.78	0.72	1.75
YBR158W	ICS4	10572	6155	1.72	0.78	0.72	1.75
YOR267C		2694	1569	1.72	0.78	0.72	1.75
YOL086C	ADH1	5435	3166	1.72	0.78	0.72	1.75
YFL047W		3039	1775	1.71	0.78	0.72	1.74
YCR009C	RVS161	1191	696	1.71	0.78	0.72	1.74
YBR003W	COQ1	1954	1143	1.71	0.77	0.72	1.73
YNL134C		2342	1370	1.71	0.77	0.72	1.73

YNL326C		1288	754	1.71	0.77	0.72	1.73
YOR187W	TUF1	2187	1280	1.71	0.77	0.72	1.73
YER147C		2069	1212	1.71	0.77	0.72	1.73
YDL185W	TFP1	7648	4481	1.71	0.77	0.72	1.73
YOR303W	CPA1	1188	696	1.71	0.77	0.72	1.73
YLR247C		3777	2214	1.71	0.77	0.71	1.73
YOR062C		1802	1057	1.70	0.77	0.71	1.72
YCL067C	HMLALPHA2	874	513	1.70	0.77	0.71	1.72
YOR237W	HES1	1208	710	1.70	0.77	0.71	1.72
YOR096W	RPS7A	1303	766	1.70	0.77	0.71	1.71
YJR077C	MIR1	1833	1079	1.70	0.76	0.71	1.71
YOR313C	SPS4	569	335	1.70	0.76	0.71	1.71
YPR202W		6369	3756	1.70	0.76	0.71	1.70
YLR439W	MRPL4	1080	637	1.70	0.76	0.71	1.70
YMR144W		657	388	1.69	0.76	0.70	1.70
YPR158W		889	525	1.69	0.76	0.70	1.70
YGL236C	MTO1	1322	782	1.69	0.76	0.70	1.69
YBR156C	SLI15	4056	2399	1.69	0.76	0.70	1.69
YKL153W		7294	4322	1.69	0.76	0.70	1.69
YHR156C		1162	689	1.69	0.75	0.70	1.69
YPR138C	MEP3	2229	1322	1.69	0.75	0.70	1.68
YPL011C	TAF47	1267	752	1.68	0.75	0.70	1.68
YOL149W	DCP1	454	270	1.68	0.75	0.69	1.68
YLR461W	PAU4	789	470	1.68	0.75	0.69	1.67
YER092W		438	261	1.68	0.75	0.69	1.67
YOL040C	RPS15	5227	3113	1.68	0.75	0.69	1.67
YOL030W		6034	3594	1.68	0.75	0.69	1.67
YKL056C		1931	1151	1.68	0.75	0.69	1.67
YOR048C	RAT1	1210	721	1.68	0.75	0.69	1.67
YPR149W	NCE102	2471	1474	1.68	0.75	0.69	1.66
YPL037C	EGD1	1836	1095	1.68	0.75	0.69	1.66
YFL041W	FET5	3766	2247	1.68	0.74	0.69	1.66
YKR040C		1428	852	1.68	0.74	0.69	1.66
YOR108W		848	506	1.68	0.74	0.69	1.66
YDR351W	SBE2	2142	1279	1.67	0.74	0.69	1.66
YDL245C	HXT15	486	290	1.67	0.74	0.69	1.66

YJR058C	APS2	954	570	1.67	0.74	0.69	1.66
YEL074W		5613	3358	1.67	0.74	0.69	1.65
YDR301W	CFT1	677	405	1.67	0.74	0.68	1.65
YIL107C	PFK26	792	474	1.67	0.74	0.68	1.65
YDL133W		754	452	1.67	0.74	0.68	1.65
YIL155C	GUT2	569	341	1.67	0.74	0.68	1.65
YKR076W	ECM4	555	333	1.67	0.74	0.68	1.64
YJL112W		2207	1326	1.66	0.74	0.68	1.64
YFR024C		1729	1039	1.66	0.73	0.68	1.64
YNL102W	POL1	668	402	1.66	0.73	0.68	1.64
YDL107W	MSS2	740	445	1.66	0.73	0.68	1.64
YPL259C	APM1	1218	733	1.66	0.73	0.68	1.63
YDL047W	SIT4	2170	1306	1.66	0.73	0.68	1.63
YDR363W-A	YDR363W-A	2130	1282	1.66	0.73	0.68	1.63
YPL277C		1809	1089	1.66	0.73	0.68	1.63
YMR235C	RNA1	1141	687	1.66	0.73	0.68	1.63
YOL064C	MET22	1173	707	1.66	0.73	0.67	1.63
YBR188C	NTC20	1058	638	1.66	0.73	0.67	1.63
YGL161C		2238	1349	1.66	0.73	0.67	1.63
YKL140W	TGL1	2153	1298	1.66	0.73	0.67	1.63
YPL023C	MET12	2527	1524	1.66	0.73	0.67	1.63
YMR282C	AEP2	1940	1170	1.66	0.73	0.67	1.63
YOR301W	RAX1	1540	929	1.66	0.73	0.67	1.63
YCR052W	RSC6	7419	4480	1.66	0.73	0.67	1.62
YPL249C-A	YPL249C-A	1164	703	1.66	0.73	0.67	1.62
YPR060C	ARO7	827	500	1.65	0.73	0.67	1.62
YKL152C	GPM1	1880	1137	1.65	0.73	0.67	1.62
YPL055C		1099	665	1.65	0.73	0.67	1.62
YPL233W		751	455	1.65	0.72	0.67	1.61
YMR015C	ERG5	1555	943	1.65	0.72	0.67	1.61
YGL172W	NUP49	2121	1287	1.65	0.72	0.66	1.61
YCR039C	MATALPHA2	992	602	1.65	0.72	0.66	1.60
YOR113W	AZF1	608	369	1.65	0.72	0.66	1.60
YLR299W	ECM38	1483	901	1.65	0.72	0.66	1.60
YPL069C	BTS1	966	587	1.65	0.72	0.66	1.60
YLR293C	GSP1	1768	1074	1.65	0.72	0.66	1.60

YNL214W	PEX17	953	579	1.65	0.72	0.66	1.60
YDR363W	ESC2	1334	811	1.65	0.72	0.66	1.60
YLR077W		1661	1010	1.64	0.72	0.66	1.60
YPR181C	SEC23	4320	2629	1.64	0.72	0.66	1.60
YAL035C-A		1206	734	1.64	0.72	0.66	1.59
YER126C		1511	920	1.64	0.72	0.66	1.59
YER102W	RPS8B	2383	1451	1.64	0.72	0.66	1.59
YKL168C	KKQ8	576	351	1.64	0.72	0.66	1.59
YHL034C	SBP1	2763	1683	1.64	0.72	0.66	1.59
YOR304C-A		466	284	1.64	0.71	0.66	1.59
YEL026W	SNU13	1773	1080	1.64	0.71	0.66	1.59
YNL118C	DCP2	2956	1802	1.64	0.71	0.66	1.59
YOR238W		1200	732	1.64	0.71	0.66	1.59
YJR121W	ATP2	1988	1213	1.64	0.71	0.66	1.59
YCLX05C		1015	620	1.64	0.71	0.65	1.58
YDR419W	RAD30	1310	801	1.64	0.71	0.65	1.58
YOL021C	DIS3	2693	1647	1.64	0.71	0.65	1.58
YGL189C	RPS26A	2250	1376	1.63	0.71	0.65	1.58
YOR252W		1593	975	1.63	0.71	0.65	1.58
YML123C	PHO84	5386	3296	1.63	0.71	0.65	1.58
YCR007C		590	361	1.63	0.71	0.65	1.57
YGR283C		1540	943	1.63	0.71	0.65	1.57
YHR136C	SPL2	3005	1844	1.63	0.70	0.65	1.57
YKR025W	RPC37	1127	692	1.63	0.70	0.65	1.56
YDR046C	BAP3	637	391	1.63	0.70	0.65	1.56
YOR166C		1067	655	1.63	0.70	0.65	1.56
YDR298C	ATP5	1260	774	1.63	0.70	0.65	1.56
YOR348C	PUT4	805	495	1.63	0.70	0.65	1.56
YOL050C		1580	972	1.63	0.70	0.64	1.56
YPL027W		645	397	1.63	0.70	0.64	1.56
YMR204C		2763	1702	1.62	0.70	0.64	1.55
YJR001W		2454	1512	1.62	0.70	0.64	1.55
YFL055W	AGP3	1369	844	1.62	0.70	0.64	1.55
YFR037C	RSC8	3309	2041	1.62	0.70	0.64	1.55
YBR204C		1144	707	1.62	0.69	0.64	1.54
YER062C	HOR2	862	533	1.62	0.69	0.64	1.54

YKL023W		1118	691	1.62	0.69	0.64	1.54
YKL059C		1027	635	1.62	0.69	0.64	1.54
YEL047C		2569	1590	1.62	0.69	0.64	1.54
YCR023C		1753	1085	1.62	0.69	0.64	1.54
YDL071C		1459	903	1.62	0.69	0.64	1.54
YEL030W	ECM10	754	467	1.62	0.69	0.64	1.54
YDR032C		1093	677	1.62	0.69	0.64	1.53
YGR295C	COS6	4733	2932	1.61	0.69	0.63	1.53
YKL013C	ARC19	3205	1987	1.61	0.69	0.63	1.53
YGR108W	CLB1	2935	1820	1.61	0.69	0.63	1.53
YDR054C	CDC34	1475	915	1.61	0.69	0.63	1.53
YDL022W	GPD1	1550	962	1.61	0.69	0.63	1.53
YLL011W	SOF1	2218	1378	1.61	0.69	0.63	1.52
YKL093W	MBR1	613	381	1.61	0.69	0.63	1.52
YBR233W	PBP2	6054	3765	1.61	0.69	0.63	1.52
YNL322C	KRE1	892	555	1.61	0.69	0.63	1.52
YMR264W	CUE1	746	464	1.61	0.68	0.63	1.52
YMR087W		516	321	1.61	0.68	0.63	1.52
YOR234C	RPL33B	1219	759	1.61	0.68	0.63	1.52
YHR043C	DOG2	2675	1666	1.61	0.68	0.63	1.51
YLL044W		3561	2220	1.60	0.68	0.63	1.51
YFR041C		959	598	1.60	0.68	0.63	1.51
YLR105C	SEN2	1165	727	1.60	0.68	0.62	1.51
YILWTy3-1A	YILWTy3-1A	2806	1751	1.60	0.68	0.62	1.51
YOR016C	ERP4	1102	688	1.60	0.68	0.62	1.51
YPL177C	CUP9	978	611	1.60	0.68	0.62	1.50
YDR432W	NPL3	4240	2649	1.60	0.68	0.62	1.50
YJR072C		1970	1232	1.60	0.68	0.62	1.50
YMR114C		999	625	1.60	0.68	0.62	1.50
YLR270W		1254	785	1.60	0.68	0.62	1.50
YHL048W	COS8	2934	1836	1.60	0.68	0.62	1.50
YOR283W		820	513	1.60	0.68	0.62	1.50
YCR038C	BUD5	647	405	1.60	0.67	0.62	1.49
YDR189W	SLY1	2555	1601	1.60	0.67	0.62	1.49
YPL063W		2304	1444	1.60	0.67	0.62	1.49
YOR224C	RPB8	967	607	1.59	0.67	0.62	1.49

YNL046W		4423	2779	1.59	0.67	0.61	1.48
YNL049C	SFB2	2251	1415	1.59	0.67	0.61	1.48
YPR197C		1162	731	1.59	0.67	0.61	1.48
YHR118C	ORC6	3427	2158	1.59	0.67	0.61	1.48
YJL125C	GCD14	1780	1121	1.59	0.67	0.61	1.48
YBR159W		2519	1587	1.59	0.67	0.61	1.47
YJR059W	PTK2	1819	1146	1.59	0.67	0.61	1.47
YGL240W	DOC1	693	437	1.58	0.66	0.61	1.47
YNL132W		1880	1188	1.58	0.66	0.61	1.46
YEL012W	UBC8	728	460	1.58	0.66	0.61	1.46
YKR057W	RPS21A	1425	901	1.58	0.66	0.61	1.46
YGR277C		1533	970	1.58	0.66	0.60	1.46
YLR033W		8826	5588	1.58	0.66	0.60	1.46
YOL019W		3478	2203	1.58	0.66	0.60	1.45
YPL031C	PHO85	957	606	1.58	0.66	0.60	1.45
YPL064C		834	529	1.58	0.66	0.60	1.45
YDL026W		518	329	1.58	0.66	0.60	1.45
YMR313C		691	439	1.57	0.66	0.60	1.45
YLR094C	GIS3	1222	776	1.57	0.65	0.60	1.45
YMR121C	RPL15B	2896	1840	1.57	0.65	0.60	1.45
YPR142C		967	615	1.57	0.65	0.60	1.44
YLR157C-A	TyA	1126	716	1.57	0.65	0.60	1.44
YPR154W		533	339	1.57	0.65	0.60	1.44
YML080W		1435	913	1.57	0.65	0.60	1.44
YOL042W		1801	1146	1.57	0.65	0.60	1.44
YER149C	PEA2	1371	873	1.57	0.65	0.59	1.44
YPL095C		614	391	1.57	0.65	0.59	1.44
YIL170W	HXT12	810	516	1.57	0.65	0.59	1.44
YPR022C		3618	2305	1.57	0.65	0.59	1.44
YIL110W		832	530	1.57	0.65	0.59	1.43
YEL011W	GLC3	1946	1241	1.57	0.65	0.59	1.43
YAL025C	MAK16	4158	2652	1.57	0.65	0.59	1.43
YOL147C	PEX11	676	431	1.57	0.65	0.59	1.43
YFL048C	EMP47	2395	1528	1.57	0.65	0.59	1.43
YLR263W	RED1	2207	1409	1.57	0.65	0.59	1.43
YKR101W	SIR1	563	360	1.56	0.65	0.59	1.42

YNL160W	YGP1	446	285	1.56	0.64	0.59	1.42
YER144C	UBP5	1498	959	1.56	0.64	0.59	1.42
YPR148C		2275	1458	1.56	0.64	0.59	1.41
YBR236C	ABD1	1579	1012	1.56	0.64	0.59	1.41
YIL118W	RHO3	4912	3149	1.56	0.64	0.59	1.41
YGR091W	PRP31	877	562	1.56	0.64	0.59	1.41
YDR205W		651	418	1.56	0.64	0.58	1.41
YJL190C	RPS22A	1734	1113	1.56	0.64	0.58	1.41
YGR087C	PDC6	1145	736	1.56	0.64	0.58	1.41
YNL082W	PMS1	1446	929	1.56	0.64	0.58	1.41
YER113C		3594	2310	1.56	0.64	0.58	1.40
YLR367W	RPS22B	1296	833	1.56	0.64	0.58	1.40
YOL079W		1395	897	1.56	0.64	0.58	1.40
YPR086W	SUA7	2039	1312	1.55	0.64	0.58	1.40
YDR412W		2163	1392	1.55	0.64	0.58	1.40
YGL184C		719	463	1.55	0.64	0.58	1.40
YKR086W	PRP16	604	389	1.55	0.64	0.58	1.40
YOR268C		407	262	1.55	0.63	0.58	1.40
YHR010W	RPL27A	1595	1027	1.55	0.63	0.58	1.40
YGL224C		1276	822	1.55	0.63	0.58	1.40
YLR426W		1790	1153	1.55	0.63	0.58	1.40
YJR032W	CPR7	1327	855	1.55	0.63	0.58	1.40
YGL175C	SAE2	1498	965	1.55	0.63	0.58	1.40
YLR359W	ADE13	1517	978	1.55	0.63	0.58	1.39
YGL176C		564	364	1.55	0.63	0.58	1.39
YJR094C	IME1	2669	1722	1.55	0.63	0.58	1.39
YPR180W	AOS1	970	626	1.55	0.63	0.58	1.39
YGL209W	MIG2	2060	1330	1.55	0.63	0.57	1.39
YOR176W	HEM15	1385	895	1.55	0.63	0.57	1.39
YPL049C	DIG1	2980	1929	1.54	0.63	0.57	1.38
YKR042W	UTH1	1976	1280	1.54	0.63	0.57	1.38
YNL133C		2780	1801	1.54	0.63	0.57	1.38
YPL193W	RSA1	804	521	1.54	0.63	0.57	1.38
YNL113W	RPC19	1130	733	1.54	0.62	0.57	1.37
YPL048W	CAM1	1658	1076	1.54	0.62	0.57	1.37
YPL274W	SAM3	3078	1998	1.54	0.62	0.57	1.37

YOL022C		1848	1200	1.54	0.62	0.57	1.37
YOR192C		1050	682	1.54	0.62	0.57	1.37
YNR035C	ARC35	2196	1427	1.54	0.62	0.57	1.37
YJL178C		1385	900	1.54	0.62	0.57	1.37
YAL048C		8851	5754	1.54	0.62	0.57	1.36
YMR038C	LYS7	2349	1528	1.54	0.62	0.56	1.36
YOR383C		2008	1306	1.54	0.62	0.56	1.36
YIL088C		1412	919	1.54	0.62	0.56	1.36
YHL013C		1018	663	1.54	0.62	0.56	1.36
YBR290W	BSD2	1053	686	1.54	0.62	0.56	1.36
YLR061W	RPL22A	1916	1248	1.53	0.62	0.56	1.36
YBR005W		832	542	1.53	0.62	0.56	1.36
YBR109C	CMD1	1184	772	1.53	0.62	0.56	1.36
YHL015W	RPS20	2930	1911	1.53	0.62	0.56	1.35
YDR416W	SYF1	929	606	1.53	0.62	0.56	1.35
YGR169C		2211	1443	1.53	0.62	0.56	1.35
YNL001W	DOM34	4734	3091	1.53	0.62	0.56	1.35
YOR288C	MPD1	1893	1236	1.53	0.61	0.56	1.35
YGL226W		865	565	1.53	0.61	0.56	1.35
YMR297W	PRC1	2414	1578	1.53	0.61	0.56	1.35
YDL086W		1471	962	1.53	0.61	0.56	1.34
YDR108W	GSG1	483	316	1.53	0.61	0.56	1.34
YDL233W		1131	740	1.53	0.61	0.56	1.34
YOL098C		6513	4264	1.53	0.61	0.56	1.34
YKR088C		964	631	1.53	0.61	0.56	1.34
YOR314W		655	429	1.53	0.61	0.55	1.34
YGL237C	HAP2	450	295	1.53	0.61	0.55	1.34
YDR339C		597	391	1.53	0.61	0.55	1.34
YGL030W	RPL30	797	522	1.53	0.61	0.55	1.34
YHR093W	AHT1	1521	997	1.53	0.61	0.55	1.34
YNL047C		840	551	1.53	0.61	0.55	1.33
YDL054C		2264	1485	1.52	0.61	0.55	1.33
YIL103W		700	459	1.52	0.61	0.55	1.33
YJL109C		2508	1645	1.52	0.61	0.55	1.33
YER118C	SSU81	3441	2258	1.52	0.61	0.55	1.33
YAR002W		2571	1687	1.52	0.61	0.55	1.33

YER099C	PRS2	1876	1231	1.52	0.61	0.55	1.33
YDR002W	YRB1	844	554	1.52	0.61	0.55	1.33
YPL129W	ANC1	827	543	1.52	0.61	0.55	1.33
YFR038W		2240	1471	1.52	0.61	0.55	1.33
YDL059C	RAD59	769	505	1.52	0.61	0.55	1.33
YMR243C	ZRC1	3415	2244	1.52	0.61	0.55	1.33
YPR002W	PDH1	972	639	1.52	0.61	0.55	1.33
YLL061W	MMP1	1678	1103	1.52	0.61	0.55	1.33
YLR407W		812	534	1.52	0.61	0.55	1.33
YBR034C	HMT1	2933	1929	1.52	0.60	0.55	1.32
YAL051W	YAF1	1899	1249	1.52	0.60	0.55	1.32
YCL068C		926	610	1.52	0.60	0.55	1.32
YJL093C	TOK1	1949	1284	1.52	0.60	0.55	1.32
YCL025C	AGP1	2799	1845	1.52	0.60	0.55	1.32
YCR073W-A	YCR073W-A	869	573	1.52	0.60	0.55	1.32
YBR123C	TFC1	1044	688	1.52	0.60	0.55	1.32
YNL050C		5543	3654	1.52	0.60	0.55	1.32
YLR393W	ATP10	426	281	1.52	0.60	0.54	1.32
YER134C		775	511	1.52	0.60	0.54	1.32
YCLX07W		2146	1415	1.52	0.60	0.54	1.31
YAL008W	FUN14	1160	765	1.52	0.60	0.54	1.31
YOR080W		777	513	1.52	0.60	0.54	1.31
YDL199C		3060	2020	1.51	0.60	0.54	1.31
YPL246C		671	443	1.51	0.60	0.54	1.31
YLR325C	RPL38	1975	1306	1.51	0.60	0.54	1.31
YOL051W	GAL11	5465	3615	1.51	0.60	0.54	1.30
YLR073C		611	405	1.51	0.59	0.54	1.30
YOR156C	NFI1	1966	1303	1.51	0.59	0.54	1.30
YNL051W		1552	1029	1.51	0.59	0.54	1.30
YIL105C		2767	1835	1.51	0.59	0.54	1.30
YEL015W		2251	1493	1.51	0.59	0.54	1.29
YAL038W	CDC19	10678	7083	1.51	0.59	0.54	1.29
YCR040W	MATALPHA1	806	535	1.51	0.59	0.54	1.29
YJL210W	PEX2	702	466	1.51	0.59	0.54	1.29
YPL079W	RPL21B	1405	933	1.51	0.59	0.53	1.29
YNL115C		2735	1818	1.50	0.59	0.53	1.29

YEL045C		943	627	1.50	0.59	0.53	1.29
YDR347W	MRP1	573	381	1.50	0.59	0.53	1.28
YHR030C	SLT2	1227	816	1.50	0.59	0.53	1.28
YPL015C	HST2	592	394	1.50	0.59	0.53	1.28
YDL041W		4757	3167	1.50	0.59	0.53	1.28
YDR006C	SOK1	475	316	1.50	0.59	0.53	1.28
YAL069W		3754	2499	1.50	0.59	0.53	1.28
YPR019W	CDC54	3699	2463	1.50	0.59	0.53	1.28
YIL123W	SIM1	2194	1462	1.50	0.59	0.53	1.28
YOL066C	RIB2	3359	2239	1.50	0.59	0.53	1.28
YGR167W	CLC1	2256	1504	1.50	0.58	0.53	1.28
YPL003W	ULA1	459	306	1.50	0.58	0.53	1.28
YLR285W		1276	851	1.50	0.58	0.53	1.28
YDL119C		2715	1811	1.50	0.58	0.53	1.27
YOR128C	ADE2	800	534	1.50	0.58	0.53	1.27
YDR064W	RPS13	1383	923	1.50	0.58	0.53	1.27
YDR163W		644	430	1.50	0.58	0.53	1.27
YOL018C	TLG2	1258	840	1.50	0.58	0.53	1.27
YDR540C		614	410	1.50	0.58	0.53	1.27
YJL101C	GSH1	2674	1786	1.50	0.58	0.53	1.27
YNL017C		900	601	1.50	0.58	0.53	1.27
YPL081W	RPS9A	2034	1359	1.50	0.58	0.53	1.27
YOR075W	UFE1	1269	848	1.50	0.58	0.53	1.27
YHR041C	SRB2	3611	2414	1.50	0.58	0.52	1.27
YIL157C		720	482	1.49	0.58	0.52	1.26
YNL027W	CRZ1	1269	850	1.49	0.58	0.52	1.26
YHR192W		481	322	1.49	0.58	0.52	1.26
YER148W	SPT15	861	577	1.49	0.58	0.52	1.26
YKL218C	SRY1	412	276	1.49	0.58	0.52	1.26
YOL095C	HMI1	1700	1140	1.49	0.58	0.52	1.26
YDR188W	CCT6	2240	1503	1.49	0.58	0.52	1.25
YNL061W	NOP2	3672	2465	1.49	0.58	0.52	1.25
YEL072W		1578	1059	1.49	0.58	0.52	1.25
YMR267W	PPA2	1326	890	1.49	0.58	0.52	1.25
YAL039C	CYC3	1354	909	1.49	0.57	0.52	1.25
YKL001C	MET14	1012	680	1.49	0.57	0.52	1.25

YKL186C	MTR2	604	406	1.49	0.57	0.52	1.25
YER163C		949	638	1.49	0.57	0.52	1.25
YJL166W	QCR8	1173	789	1.49	0.57	0.52	1.25
YHR109W		4738	3187	1.49	0.57	0.52	1.25
YOR144C	EFD1	1483	998	1.49	0.57	0.52	1.25
YMR301C	ATM1	2971	2001	1.48	0.57	0.51	1.24
YPR023C		1701	1146	1.48	0.57	0.51	1.24
YCR041W		1340	903	1.48	0.57	0.51	1.24
YOR066W		657	443	1.48	0.57	0.51	1.24
YCR024C-A	PMP1	2862	1929	1.48	0.57	0.51	1.24
YNL079C	TPM1	2525	1702	1.48	0.57	0.51	1.24
YOR331C		992	669	1.48	0.57	0.51	1.24
YOR239W		2520	1700	1.48	0.57	0.51	1.24
YAL020C	ATS1	1257	848	1.48	0.57	0.51	1.24
YOR361C	PRT1	5083	3430	1.48	0.57	0.51	1.23
YIL141W		1285	867	1.48	0.57	0.51	1.23
YBR033W		1471	993	1.48	0.57	0.51	1.23
YPL281C	ERR2	2249	1518	1.48	0.57	0.51	1.23
YPL065W	VPS28	505	341	1.48	0.57	0.51	1.23
YNL248C	RPA49	1563	1056	1.48	0.57	0.51	1.23
YMR037C	MSN2	2136	1443	1.48	0.57	0.51	1.23
YJR154W		952	643	1.48	0.57	0.51	1.23
YGR086C		3238	2188	1.48	0.57	0.51	1.23
YPR003C		1487	1005	1.48	0.57	0.51	1.23
YPL270W	MDL2	2441	1650	1.48	0.57	0.51	1.23
YLR074C		881	596	1.48	0.56	0.51	1.23
YDR255C		1281	867	1.48	0.56	0.51	1.22
YPR132W	RPS23B	3343	2263	1.48	0.56	0.51	1.22
YPL228W	CET1	1142	773	1.48	0.56	0.51	1.22
YDR174W	HMO1	2077	1406	1.48	0.56	0.51	1.22
YIR008C	PRI1	1237	838	1.48	0.56	0.51	1.22
YIL119C	RPI1	384	260	1.48	0.56	0.51	1.22
YGL221C	NIF3	620	420	1.48	0.56	0.51	1.22
YOR256C		2502	1695	1.48	0.56	0.51	1.22
YJR031C	GEA1	1303	883	1.48	0.56	0.51	1.22
YPR157W		689	467	1.48	0.56	0.50	1.22

YPL235W	RVB2	3979	2697	1.48	0.56	0.50	1.22
YOR385W		1041	706	1.47	0.56	0.50	1.22
YGL210W	YPT32	1030	699	1.47	0.56	0.50	1.22
YPL066W		4332	2940	1.47	0.56	0.50	1.22
YOR371C		871	591	1.47	0.56	0.50	1.21
YNL337W		2850	1935	1.47	0.56	0.50	1.21
YCR068W		7096	4817	1.47	0.56	0.50	1.21
YPR063C		2078	1411	1.47	0.56	0.50	1.21
YMR280C	CAT8	1418	963	1.47	0.56	0.50	1.21
YGL012W	ERG4	4714	3207	1.47	0.56	0.50	1.21
YDR394W	RPT3	3429	2334	1.47	0.56	0.50	1.20
YHR125W		2795	1903	1.47	0.55	0.50	1.20
YGL219C		3306	2251	1.47	0.55	0.50	1.20
YDR373W	FRQ1	1126	767	1.47	0.55	0.50	1.20
YNL278W		2192	1493	1.47	0.55	0.50	1.20
YPR122W	AXL1	2683	1828	1.47	0.55	0.50	1.20
YJR085C		851	580	1.47	0.55	0.50	1.20
YER139C		1020	695	1.47	0.55	0.50	1.20
YJL012C		5064	3452	1.47	0.55	0.50	1.20
YOL016C	CMK2	978	667	1.47	0.55	0.50	1.20
YNR047W		2304	1571	1.47	0.55	0.50	1.20
YPL017C		911	621	1.47	0.55	0.50	1.20
YLR280C		800	546	1.47	0.55	0.50	1.20
YHR135C	YCK1	1135	774	1.47	0.55	0.50	1.20
YLL012W		2111	1440	1.47	0.55	0.50	1.20
YPL078C	ATP4	2264	1545	1.47	0.55	0.50	1.20
YMR308C	PSE1	8453	5769	1.47	0.55	0.50	1.20
YDR063W		1669	1139	1.46	0.55	0.49	1.19
YNL080C		664	453	1.46	0.55	0.49	1.19
YOL045W		2787	1903	1.46	0.55	0.49	1.19
YGR138C		1436	981	1.46	0.55	0.49	1.19
YBL082C	RHK1	1319	901	1.46	0.55	0.49	1.19
YNL114C		562	384	1.46	0.55	0.49	1.19
YKL103C	LAP4	1561	1067	1.46	0.55	0.49	1.19
YHR058C	MED6	1256	859	1.46	0.55	0.49	1.19
YBR217W	APG12	3074	2105	1.46	0.55	0.49	1.18

YBL068W	PRS4	2281	1562	1.46	0.55	0.49	1.18
YGR102C		581	398	1.46	0.55	0.49	1.18
YBR157C	ICS2	851	583	1.46	0.55	0.49	1.18
YPL101W		1086	744	1.46	0.55	0.49	1.18
YCR092C	MSH3	1555	1065	1.46	0.55	0.49	1.18
YGR235C		1406	963	1.46	0.55	0.49	1.18
YKL104C	GFA1	938	643	1.46	0.55	0.49	1.18
YNL067W	RPL9B	1977	1356	1.46	0.54	0.49	1.18
YFL037W	TUB2	4733	3246	1.46	0.54	0.49	1.18
YEL076C-A		3719	2551	1.46	0.54	0.49	1.18
YMR084W		544	373	1.46	0.54	0.49	1.18
YHR009C		3209	2203	1.46	0.54	0.49	1.17
YLR040C		2480	1703	1.46	0.54	0.49	1.17
YLR177W		821	564	1.46	0.54	0.49	1.17
YDR130C		1457	1001	1.46	0.54	0.49	1.17
YMR256C	COX7	1219	838	1.46	0.54	0.48	1.17
YMR206W		2302	1582	1.46	0.54	0.48	1.17
YKR047W		989	680	1.45	0.54	0.48	1.17
YOR249C	APC5	1394	958	1.45	0.54	0.48	1.17
YHR001W		1373	944	1.45	0.54	0.48	1.17
YJR124C		1418	975	1.45	0.54	0.48	1.17
YPL276W		398	274	1.45	0.54	0.48	1.17
YPL239W	YAR1	1326	912	1.45	0.54	0.48	1.17
YPL053C	KTR6	1442	992	1.45	0.54	0.48	1.17
YOL099C		994	684	1.45	0.54	0.48	1.17
YLR443W	ECM7	1016	699	1.45	0.54	0.48	1.17
YPL249C		1838	1265	1.45	0.54	0.48	1.17
YHL035C		1623	1117	1.45	0.54	0.48	1.17
YHR164C	DNA2	3373	2324	1.45	0.54	0.48	1.16
YCR013C		4041	2784	1.45	0.54	0.48	1.16
YLR046C		2061	1420	1.45	0.54	0.48	1.16
YGL218W		2792	1924	1.45	0.54	0.48	1.16
YBR129C	OPY1	760	524	1.45	0.54	0.48	1.16
YOR065W	CYT1	1494	1030	1.45	0.54	0.48	1.16
YHR085W		6307	4348	1.45	0.54	0.48	1.16
YDR398W		7058	4867	1.45	0.54	0.48	1.16

YPR076W		1417	977	1.45	0.54	0.48	1.16
YOR049C		1038	716	1.45	0.54	0.48	1.16
YKL041W	VPS24	1694	1169	1.45	0.54	0.48	1.16
YDR397C	NCB2	523	361	1.45	0.54	0.48	1.16
YDR168W	CDC37	6273	4330	1.45	0.53	0.48	1.16
YLR314C	CDC3	3389	2340	1.45	0.53	0.48	1.16
YHR163W	SOL3	3671	2535	1.45	0.53	0.48	1.15
YLR147C	SMD3	2720	1878	1.45	0.53	0.48	1.15
YBR011C	IPP1	6407	4428	1.45	0.53	0.48	1.15
YHR089C	GAR1	4968	3434	1.45	0.53	0.48	1.15
YMR298W		3430	2372	1.45	0.53	0.48	1.15
YMR146C	TIF34	4763	3295	1.45	0.53	0.48	1.15
YER087W		611	423	1.45	0.53	0.48	1.15
YDL075W	RPL31A	1158	801	1.45	0.53	0.48	1.15
YLR343W		530	367	1.45	0.53	0.48	1.15
YGR117C		1016	703	1.45	0.53	0.48	1.15
YPL258C	THI21	1024	709	1.44	0.53	0.47	1.15
YNL106C	INP52	1796	1243	1.44	0.53	0.47	1.15
YDR385W	EFT2	10038	6949	1.44	0.53	0.47	1.15
YOR060C		1722	1193	1.44	0.53	0.47	1.14
YNR012W	URK1	2302	1595	1.44	0.53	0.47	1.14
YLR121C	YPS3	747	518	1.44	0.53	0.47	1.14
YMR226C		4922	3413	1.44	0.53	0.47	1.14
YLR042C		627	435	1.44	0.53	0.47	1.14
YLR289W	GUF1	1625	1127	1.44	0.53	0.47	1.14
YLR286C	CTS1	6038	4190	1.44	0.53	0.47	1.14
YNL070W	TOM7	1882	1306	1.44	0.53	0.47	1.14
YBR141C		539	374	1.44	0.53	0.47	1.14
YIL139C	REV7	471	327	1.44	0.53	0.47	1.14
YJL124C	LSM1	1246	865	1.44	0.53	0.47	1.14
YNL256W	FOL1	2866	1991	1.44	0.53	0.47	1.13
YOR138C		1039	722	1.44	0.52	0.47	1.13
YER114C	BOI2	2224	1547	1.44	0.52	0.47	1.13
YGR159C	NSR1	4854	3377	1.44	0.52	0.47	1.13
YHR121W		1000	696	1.44	0.52	0.47	1.13
YPL128C	TBF1	1317	917	1.44	0.52	0.47	1.13

YPR152C		1298	904	1.44	0.52	0.47	1.12
YIL050W	PCL7	3045	2121	1.44	0.52	0.47	1.12
YLR300W	EXG1	2980	2076	1.44	0.52	0.47	1.12
YGR106C		1534	1069	1.44	0.52	0.47	1.12
YAL030W	SNC1	5887	4103	1.43	0.52	0.46	1.12
YJL156C	SSY5	691	482	1.43	0.52	0.46	1.12
YNR003C	RPC34	668	466	1.43	0.52	0.46	1.12
YBL050W	SEC17	840	586	1.43	0.52	0.46	1.12
YEL076W-C		4271	2979	1.43	0.52	0.46	1.12
YOR308C	SNU66	2888	2015	1.43	0.52	0.46	1.12
YMR239C	RNT1	367	256	1.43	0.52	0.46	1.12
YEL027W	CUP5	2933	2047	1.43	0.52	0.46	1.12
YFL021W	GAT1	2952	2061	1.43	0.52	0.46	1.12
YDL215C	GDH2	2037	1423	1.43	0.52	0.46	1.11
YER040W	GLN3	2148	1501	1.43	0.52	0.46	1.11
YER030W		1182	826	1.43	0.52	0.46	1.11
YLR161W		3565	2493	1.43	0.52	0.46	1.11
YNL188W	KAR1	2776	1942	1.43	0.52	0.46	1.11
YOR335C	ALA1	7057	4937	1.43	0.52	0.46	1.11
YBR009C	HHF1	863	604	1.43	0.52	0.46	1.11
YFR029W	PTR3	2095	1466	1.43	0.51	0.46	1.11
YLR193C		484	339	1.43	0.51	0.46	1.11
YBR079C	RPG1	7396	5179	1.43	0.51	0.46	1.11
YBR078W	ECM33	3416	2392	1.43	0.51	0.46	1.11
YLL045C	RPL8B	6499	4552	1.43	0.51	0.46	1.10
YMR067C		599	420	1.43	0.51	0.46	1.10
YKR072C	SIS2	2824	1979	1.43	0.51	0.46	1.10
YHR025W	THR1	1832	1284	1.43	0.51	0.46	1.10
YPL198W	RPL7B	4374	3067	1.43	0.51	0.46	1.10
YPL097W	MSY1	1854	1300	1.43	0.51	0.46	1.10
YPL094C	SEC62	2545	1785	1.43	0.51	0.46	1.10
YHR016C	YSC84	1092	766	1.43	0.51	0.46	1.10
YLR134W	PDC5	4426	3104	1.43	0.51	0.46	1.10
YLR108C		2644	1855	1.43	0.51	0.46	1.10
YOR201C	PET56	1361	955	1.43	0.51	0.45	1.10
YMR234W	RNH1	2896	2033	1.42	0.51	0.45	1.10

YHL001W	RPL14B	1235	867	1.42	0.51	0.45	1.10
YIL121W		929	652	1.42	0.51	0.45	1.10
YMR257C	PET111	3669	2576	1.42	0.51	0.45	1.10
YAL021C	CCR4	2711	1904	1.42	0.51	0.45	1.10
YGL129C		2858	2007	1.42	0.51	0.45	1.10
YDR433W		3613	2538	1.42	0.51	0.45	1.10
YIL122W		1912	1343	1.42	0.51	0.45	1.10
YKL117W	SBA1	4366	3068	1.42	0.51	0.45	1.09
YGR214W	RPS0A	4012	2819	1.42	0.51	0.45	1.09
YPR165W	RHO1	1551	1090	1.42	0.51	0.45	1.09
YCL036W		4525	3180	1.42	0.51	0.45	1.09
YHR011W		750	527	1.42	0.51	0.45	1.09
YPL256C	CLN2	1780	1252	1.42	0.51	0.45	1.09
YPR026W	ATH1	1587	1117	1.42	0.51	0.45	1.09
YMR052C-A		524	369	1.42	0.51	0.45	1.09
YOL060C	AMI3	3909	2751	1.42	0.51	0.45	1.09
YGR090W		1844	1298	1.42	0.51	0.45	1.09
YBR060C	ORC2	2500	1761	1.42	0.51	0.45	1.09
YPL227C	ALG5	576	406	1.42	0.51	0.45	1.09
YOR311C		1655	1166	1.42	0.51	0.45	1.09
YCL062W		750	528	1.42	0.51	0.45	1.09
YGL166W	CUP2	585	412	1.42	0.51	0.45	1.08
YDR429C	TIF35	2525	1779	1.42	0.50	0.45	1.08
YPR108W	RPN7	4863	3428	1.42	0.50	0.45	1.08
YNL178W	RPS3	496	350	1.42	0.50	0.45	1.08
YLR206W	ENT2	3343	2357	1.42	0.50	0.45	1.08
YLR190W		2119	1494	1.42	0.50	0.45	1.08
YPR016C	TIF6	2048	1444	1.42	0.50	0.45	1.08
YMR027W	HRT2	2246	1585	1.42	0.50	0.45	1.08
YOL129W		567	400	1.42	0.50	0.45	1.08
YFL016C	MDJ1	2452	1731	1.42	0.50	0.45	1.08
YBL098W		561	396	1.42	0.50	0.45	1.08
YIL106W	MOB1	1315	929	1.42	0.50	0.45	1.08
YLR054C		2112	1492	1.42	0.50	0.45	1.07
YIL140W	SRO4	1988	1405	1.42	0.50	0.44	1.07
YLR045C	STU2	2044	1445	1.41	0.50	0.44	1.07

YOR090C	PTC5	2802	1981	1.41	0.50	0.44	1.07
YGL223C		1283	908	1.41	0.50	0.44	1.07
YLR238W		1733	1226	1.41	0.50	0.44	1.07
YLR335W	NUP2	958	678	1.41	0.50	0.44	1.07
YPL260W		1843	1305	1.41	0.50	0.44	1.07
YKL057C	NUP120	408	289	1.41	0.50	0.44	1.07
YGR150C		1190	843	1.41	0.50	0.44	1.07
YGL242C		1263	895	1.41	0.50	0.44	1.06
YDL083C	RPS16B	2677	1898	1.41	0.50	0.44	1.06
YOR065W	CYT1	1395	989	1.41	0.50	0.44	1.06
YKL100C		2812	1994	1.41	0.50	0.44	1.06
YGR279C	SCW4	4236	3004	1.41	0.50	0.44	1.06
YGR296W	YRF1-3	1642	1165	1.41	0.50	0.44	1.06
YNL239W	LAP3	4721	3350	1.41	0.49	0.44	1.06
YCR028C	FEN2	1878	1333	1.41	0.49	0.44	1.06
YDR384C		690	490	1.41	0.49	0.44	1.06
YOL036W		2063	1465	1.41	0.49	0.44	1.06
YPR185W	APG13	3054	2169	1.41	0.49	0.44	1.06
YDL176W		1751	1244	1.41	0.49	0.44	1.06
YPL084W	BRO1	1200	853	1.41	0.49	0.44	1.05
YPR008W		1796	1277	1.41	0.49	0.44	1.05
YJL141C	YAK1	1700	1209	1.41	0.49	0.44	1.05
YCR043C		857	610	1.41	0.49	0.43	1.05
YOR208W	PTP2	1150	819	1.40	0.49	0.43	1.05
YHR141C	RPL42B	1081	770	1.40	0.49	0.43	1.05
YGL103W	RPL28	6290	4480	1.40	0.49	0.43	1.05
YDR014W		608	433	1.40	0.49	0.43	1.05
YGL013C	PDR1	1472	1049	1.40	0.49	0.43	1.05
YDR094W		919	655	1.40	0.49	0.43	1.04
YNL021W	HDA1	2893	2062	1.40	0.49	0.43	1.04
YAL055W		398	284	1.40	0.49	0.43	1.04
YNL090W	RHO2	1109	791	1.40	0.49	0.43	1.04
YHR055C	CUP1-2	757	540	1.40	0.49	0.43	1.04
YLR351C	NIT3	828	591	1.40	0.49	0.43	1.04
YGL225W	GOG5	1247	890	1.40	0.49	0.43	1.04
YPL255W	BBP1	769	549	1.40	0.49	0.43	1.04

YPR190C	RPC82	2056	1469	1.40	0.49	0.43	1.04
YBL091C	MAP2	2016	1441	1.40	0.48	0.43	1.03
YCRWdelta9	YCRWdelta9	631	451	1.40	0.48	0.43	1.03
YDR380W		1127	806	1.40	0.48	0.43	1.03
YFL049W		1164	832	1.40	0.48	0.43	1.03
YPR083W		1110	794	1.40	0.48	0.43	1.03
YPR198W	SGE1	831	594	1.40	0.48	0.43	1.03
YGR293C		777	556	1.40	0.48	0.43	1.03
YMR220W	ERG8	492	352	1.40	0.48	0.43	1.03
YGL110C		976	699	1.40	0.48	0.43	1.03
YMR116C	ASC1	2182	1563	1.40	0.48	0.43	1.03
YJR097W		1653	1184	1.40	0.48	0.43	1.03
YLR326W		777	557	1.40	0.48	0.42	1.03
YGR154C		1006	721	1.40	0.48	0.42	1.03
YOR067C	ALG8	1482	1062	1.40	0.48	0.42	1.03
YMR310C		2196	1574	1.40	0.48	0.42	1.03
YJR073C	OPI3	1780	1276	1.39	0.48	0.42	1.02
YNL086W		2516	1804	1.39	0.48	0.42	1.02
YNL112W	DBP2	4056	2909	1.39	0.48	0.42	1.02
YOR171C	LCB4	1764	1266	1.39	0.48	0.42	1.02
YKR087C		656	471	1.39	0.48	0.42	1.02
YNL255C	GIS2	4209	3022	1.39	0.48	0.42	1.02
YDR300C	PRO1	1422	1021	1.39	0.48	0.42	1.02
YOR347C	PYK2	895	643	1.39	0.48	0.42	1.02
YDR118W	APC4	1856	1334	1.39	0.48	0.42	1.02
YBR076W	ECM8	878	631	1.39	0.48	0.42	1.01
YAL003W	EFB1	5198	3737	1.39	0.48	0.42	1.01
YFL018C	LPD1	2022	1454	1.39	0.48	0.42	1.01
YOL100W	PKH2	5784	4161	1.39	0.48	0.42	1.01
YNL127W		2121	1527	1.39	0.47	0.42	1.01
YLL010C		2010	1447	1.39	0.47	0.42	1.01
YFL035C		671	483	1.39	0.47	0.42	1.01
YBL005W	PDR3	2307	1661	1.39	0.47	0.42	1.01
YEL043W		2404	1731	1.39	0.47	0.42	1.01
YER117W	RPL23B	2577	1856	1.39	0.47	0.42	1.01
YNL258C		2615	1884	1.39	0.47	0.42	1.01

YCL065W		466	336	1.39	0.47	0.42	1.01
YPR178W	PRP4	1675	1207	1.39	0.47	0.42	1.01
YFR031C-A	RPL2A	5257	3790	1.39	0.47	0.42	1.00
YNL110C		1327	957	1.39	0.47	0.42	1.00
YDR413C		2532	1826	1.39	0.47	0.42	1.00
YGR118W	RPS23A	1963	1416	1.39	0.47	0.41	1.00
YOL034W		1717	1239	1.39	0.47	0.41	1.00
YGR155W	CYS4	4874	3517	1.39	0.47	0.41	1.00
YNL303W		1535	1108	1.39	0.47	0.41	1.00
YMR134W		951	686	1.39	0.47	0.41	1.00
YIR011C	STS1	1107	799	1.39	0.47	0.41	1.00
YLR093C	NYV1	687	496	1.38	0.47	0.41	1.00
YDL014W	NOP1	4868	3517	1.38	0.47	0.41	1.00
YDR050C	TPI1	3568	2579	1.38	0.47	0.41	1.00
YJR002W	MPP10	737	533	1.38	0.47	0.41	1.00
YPL096W		884	639	1.38	0.47	0.41	0.99
YLR237W	THI7	1302	941	1.38	0.47	0.41	0.99
YCR061W		2285	1652	1.38	0.47	0.41	0.99
YKL128C	PMU1	1606	1161	1.38	0.47	0.41	0.99
YER165W	PAB1	3066	2217	1.38	0.47	0.41	0.99
YLR110C		2169	1569	1.38	0.47	0.41	0.99
YLR150W	STM1	7312	5292	1.38	0.47	0.41	0.99
YPR010C	RPA135	3364	2435	1.38	0.47	0.41	0.99
YOR154W		1101	797	1.38	0.47	0.41	0.99
YIL109C	SEC24	5498	3983	1.38	0.47	0.41	0.99
YHL039W		1916	1388	1.38	0.46	0.41	0.99
YNR048W		2590	1877	1.38	0.46	0.41	0.99
YAL009W	SPO7	406	294	1.38	0.46	0.41	0.99
YGR170W	PSD2	2166	1571	1.38	0.46	0.41	0.98
YMR302C	PRP12	1859	1348	1.38	0.46	0.41	0.98
YFR051C	RET2	2934	2129	1.38	0.46	0.41	0.98
YOR373W	NUD1	2270	1648	1.38	0.46	0.41	0.98
YLR391W		843	612	1.38	0.46	0.41	0.98
YKR027W		2601	1890	1.38	0.46	0.40	0.98
YLR227C		4417	3210	1.38	0.46	0.40	0.98
YBLWdelta1	YBLWdelta1	1222	888	1.38	0.46	0.40	0.98

YER132C	PMD1	3859	2805	1.38	0.46	0.40	0.98
YDR226W	ADK1	2910	2116	1.38	0.46	0.40	0.97
YJL013C	MAD3	3509	2553	1.37	0.46	0.40	0.97
YCR037C	PHO87	1435	1044	1.37	0.46	0.40	0.97
YPL091W	GLR1	1681	1223	1.37	0.46	0.40	0.97
YLR337C	VRP1	591	430	1.37	0.46	0.40	0.97
YOR069W	VPS5	1493	1087	1.37	0.46	0.40	0.97
YBR035C	PDX3	1354	986	1.37	0.46	0.40	0.97
YBR228W		9099	6628	1.37	0.46	0.40	0.97
YOL097C	WRS1	1242	905	1.37	0.46	0.40	0.97
YNL033W		1399	1019	1.37	0.46	0.40	0.97
YBL003C	HTA2	2675	1949	1.37	0.46	0.40	0.97
YOR259C	RPT4	1914	1395	1.37	0.46	0.40	0.97
YOR302W		484	353	1.37	0.46	0.40	0.97
YPR036W	VMA13	1974	1439	1.37	0.46	0.40	0.97
YJL105W		1129	823	1.37	0.46	0.40	0.96
YOR198C	BFR1	5644	4116	1.37	0.46	0.40	0.96
YOR299W	BUD7	498	363	1.37	0.46	0.40	0.96
YGR010W		1655	1208	1.37	0.45	0.40	0.96
YOR043W	WHI2	2348	1714	1.37	0.45	0.40	0.96
YHR207C		501	366	1.37	0.45	0.40	0.96
YJR148W	BAT2	1969	1438	1.37	0.45	0.40	0.96
YNL032W	SIW14	1854	1354	1.37	0.45	0.40	0.96
YLR170C	APS1	14282	10432	1.37	0.45	0.40	0.96
YNL126W	SPC98	923	674	1.37	0.45	0.40	0.96
YDR035W	ARO3	1900	1390	1.37	0.45	0.39	0.95
YBR168W		4257	3116	1.37	0.45	0.39	0.95
YHR072W	ERG7	1952	1429	1.37	0.45	0.39	0.95
YGR119C	NUP57	3958	2898	1.37	0.45	0.39	0.95
YER164W	CHD1	2763	2024	1.37	0.45	0.39	0.95
YLR211C		3102	2273	1.36	0.45	0.39	0.95
YGR133W	PEX4	980	718	1.36	0.45	0.39	0.95
YPL238C		3094	2268	1.36	0.45	0.39	0.95
YGL009C	LEU1	5123	3758	1.36	0.45	0.39	0.94
YCR036W	RBK1	954	700	1.36	0.45	0.39	0.94
YMR091C	NPL6	2355	1728	1.36	0.45	0.39	0.94

YAL034W-A	MTW1	2964	2175	1.36	0.45	0.39	0.94
YMR191W		2985	2191	1.36	0.45	0.39	0.94
YMR196W		1538	1129	1.36	0.45	0.39	0.94
YJR080C		1322	972	1.36	0.44	0.39	0.94
YLR417W	VPS36	696	512	1.36	0.44	0.39	0.94
YHR139C	SPS100	2322	1708	1.36	0.44	0.39	0.94
YIL092W		465	342	1.36	0.44	0.39	0.93
YPL225W		776	571	1.36	0.44	0.39	0.93
YLR268W	SEC22	987	726	1.36	0.44	0.39	0.93
YKL108W	SLD2	466	343	1.36	0.44	0.39	0.93
YGR282C	BGL2	2690	1980	1.36	0.44	0.39	0.93
YMR004W	MVP1	2009	1479	1.36	0.44	0.39	0.93
YGR250C		2435	1793	1.36	0.44	0.39	0.93
YOR160W	MTR10	884	651	1.36	0.44	0.39	0.93
YDR117C		2591	1909	1.36	0.44	0.38	0.93
YLR192C	HCR1	1977	1457	1.36	0.44	0.38	0.93
YEL031W	SPF1	697	514	1.36	0.44	0.38	0.93
YJL069C		1782	1314	1.36	0.44	0.38	0.93
YML054C	CYB2	2190	1615	1.36	0.44	0.38	0.93
YDR012W	RPL4B	3017	2225	1.36	0.44	0.38	0.93
YKL120W	OAC1	565	417	1.36	0.44	0.38	0.93
YMR145C		1838	1356	1.36	0.44	0.38	0.92
YPR102C	RPL11A	1717	1267	1.36	0.44	0.38	0.92
YPL154C	PEP4	4552	3359	1.36	0.44	0.38	0.92
YIL125W	KGD1	4087	3016	1.35	0.44	0.38	0.92
YIL090W		2552	1884	1.35	0.44	0.38	0.92
YDR426C		1653	1220	1.35	0.44	0.38	0.92
YILWTy3-1C	YILWTy3-1C	1962	1448	1.35	0.44	0.38	0.92
YMR011W	HXT2	3159	2332	1.35	0.44	0.38	0.92
YGR160W		5452	4025	1.35	0.44	0.38	0.92
YMR268C	PRP24	1600	1181	1.35	0.44	0.38	0.92
YBR122C	MRPL36	6013	4440	1.35	0.44	0.38	0.92
YDR529C	QCR7	1602	1183	1.35	0.44	0.38	0.92
YGR175C	ERG1	2463	1819	1.35	0.44	0.38	0.92
YGR085C	RPL11B	4466	3299	1.35	0.44	0.38	0.92
YLL001W	DNM1	999	738	1.35	0.44	0.38	0.92

YLR409C		2350	1737	1.35	0.44	0.38	0.92
YJL193W		1176	869	1.35	0.44	0.38	0.92
YER131W	RPS26B	5759	4257	1.35	0.44	0.38	0.92
YKR053C	YSR3	602	445	1.35	0.44	0.38	0.92
YLR370C	ARC18	1369	1013	1.35	0.43	0.38	0.91
YMR295C		2462	1821	1.35	0.43	0.38	0.91
YAL004W		10681	7905	1.35	0.43	0.38	0.91
YCR014C	POL4	1577	1167	1.35	0.43	0.38	0.91
YLR425W	TUS1	984	729	1.35	0.43	0.38	0.91
YJL075C		975	722	1.35	0.43	0.38	0.91
YCLX04W		717	531	1.35	0.43	0.38	0.91
YNL273W	TOF1	837	620	1.35	0.43	0.38	0.91
YFR053C	HXK1	1399	1037	1.35	0.43	0.38	0.91
YGR171C	MSM1	972	721	1.35	0.43	0.38	0.91
YOR355W	GDS1	4175	3100	1.35	0.43	0.37	0.90
YJL209W	CBP1	1780	1322	1.35	0.43	0.37	0.90
YDR221W		1012	752	1.35	0.43	0.37	0.90
YBR029C	CDS1	1061	788	1.35	0.43	0.37	0.90
YJR130C		763	567	1.35	0.43	0.37	0.90
YDR185C		4085	3037	1.35	0.43	0.37	0.90
YNL204C	SPS18	982	730	1.35	0.43	0.37	0.90
YGR267C	FOL2	1322	983	1.35	0.43	0.37	0.90
YBR161W		655	487	1.35	0.43	0.37	0.90
YKL206C		866	644	1.34	0.43	0.37	0.90
YPR079W		2539	1889	1.34	0.43	0.37	0.89
YDR225W	HTA1	4548	3384	1.34	0.43	0.37	0.89
YDR128W		1309	974	1.34	0.43	0.37	0.89
YBR283C	SSH1	4703	3500	1.34	0.43	0.37	0.89
YLR236C		392	292	1.34	0.43	0.37	0.89
YPL202C		5046	3757	1.34	0.43	0.37	0.89
YER180C	ISC10	481	358	1.34	0.43	0.37	0.89
YKR056W	RNC1	1639	1221	1.34	0.43	0.37	0.89
YGR177C	ATF2	2655	1979	1.34	0.42	0.37	0.89
YPR171W		934	696	1.34	0.42	0.37	0.89
YOR167C	RPS28A	5156	3844	1.34	0.42	0.37	0.89
YKL144C	RPC25	1510	1126	1.34	0.42	0.37	0.89

YDL238C		1250	932	1.34	0.42	0.37	0.89
YGR135W	PRE9	1562	1165	1.34	0.42	0.37	0.89
YJR095W	SFC1	775	578	1.34	0.42	0.37	0.89
YFR052W	RPN12	3542	2642	1.34	0.42	0.37	0.89
YHL007C	STE20	3824	2853	1.34	0.42	0.37	0.88
YPR114W		1718	1282	1.34	0.42	0.37	0.88
YBR205W	KTR3	4768	3558	1.34	0.42	0.37	0.88
YDR152W		2445	1825	1.34	0.42	0.37	0.88
YGL212W	VAM7	633	473	1.34	0.42	0.36	0.88
YDL150W	RPC53	2648	1979	1.34	0.42	0.36	0.88
YOR115C	TRS33	510	381	1.34	0.42	0.36	0.88
YKL039W	PTM1	1136	849	1.34	0.42	0.36	0.88
YIL019W		2285	1709	1.34	0.42	0.36	0.88
YEL075C		6277	4697	1.34	0.42	0.36	0.87
YGL075C	MPS2	1201	899	1.34	0.42	0.36	0.87
YLR250W	SSP120	750	561	1.34	0.42	0.36	0.87
YHLCsigma1	YHLCsigma1	492	368	1.34	0.42	0.36	0.87
YER026C	CHO1	3814	2856	1.34	0.42	0.36	0.87
YPL180W		1207	904	1.34	0.42	0.36	0.87
YNL163C		794	595	1.33	0.42	0.36	0.87
YER154W	OXA1	1267	949	1.33	0.42	0.36	0.87
YEL042W	GDA1	1024	768	1.33	0.42	0.36	0.87
YBR220C		1906	1429	1.33	0.42	0.36	0.87
YKL025C	PAN3	1382	1036	1.33	0.42	0.36	0.87
YHL029C		1775	1332	1.33	0.41	0.36	0.86
YOL082W		1049	787	1.33	0.41	0.36	0.86
YML035C-A		1102	827	1.33	0.41	0.36	0.86
YKL194C	MST1	360	270	1.33	0.41	0.36	0.86
YNL196C	SLZ1	1344	1010	1.33	0.41	0.36	0.86
YOR102W		605	455	1.33	0.41	0.36	0.86
YOR321W	PMT3	1145	861	1.33	0.41	0.36	0.86
YBL092W	RPL32	4342	3264	1.33	0.41	0.36	0.86
YGR195W	SKI6	1704	1281	1.33	0.41	0.36	0.86
YHR144C	DCD1	746	561	1.33	0.41	0.35	0.86
YNR063W		448	337	1.33	0.41	0.35	0.86
YKR023W		1460	1099	1.33	0.41	0.35	0.85

YML032C	RAD52	6473	4872	1.33	0.41	0.35	0.85
YHR040W		1384	1042	1.33	0.41	0.35	0.85
YOR140W	SFL1	1357	1022	1.33	0.41	0.35	0.85
YOR315W		1317	992	1.33	0.41	0.35	0.85
YJR016C	ILV3	2905	2188	1.33	0.41	0.35	0.85
YGL111W		2712	2043	1.33	0.41	0.35	0.85
YHR066W	SSF1	1089	820	1.33	0.41	0.35	0.85
YOR173W		1725	1300	1.33	0.41	0.35	0.85
YPL036W	PMA2	746	562	1.33	0.41	0.35	0.85
YOL076W	MDM20	2694	2030	1.33	0.41	0.35	0.85
YLR167W	RPS31	2124	1601	1.33	0.41	0.35	0.85
YDR062W	LCB2	5479	4131	1.33	0.41	0.35	0.85
YNL034W		3090	2330	1.33	0.41	0.35	0.85
YNR045W	PET494	557	420	1.33	0.41	0.35	0.85
YKL047W		1020	769	1.33	0.41	0.35	0.85
YFL035C-B		1905	1437	1.33	0.41	0.35	0.85
YFL061W		435	328	1.33	0.41	0.35	0.85
YIL083C		1039	784	1.33	0.41	0.35	0.85
YKR089C		775	585	1.32	0.41	0.35	0.84
YJR105W		3560	2688	1.32	0.41	0.35	0.84
YNR008W	LRO1	4215	3183	1.32	0.41	0.35	0.84
YBR121C	GRS1	6478	4894	1.32	0.40	0.35	0.84
YJR100C		670	506	1.32	0.40	0.35	0.84
YDL069C	CBS1	456	345	1.32	0.40	0.35	0.84
YNL099C		3555	2687	1.32	0.40	0.35	0.84
YHL026C		443	335	1.32	0.40	0.35	0.84
YBR299W	MAL32	7265	5493	1.32	0.40	0.35	0.84
YIR012W	SQT1	2268	1715	1.32	0.40	0.35	0.84
YLR222C		2010	1521	1.32	0.40	0.35	0.84
YPR139C		1911	1446	1.32	0.40	0.35	0.84
YBR063C		1115	844	1.32	0.40	0.35	0.84
YGL155W	CDC43	2671	2022	1.32	0.40	0.35	0.83
YKL006C-A	SFT1	699	529	1.32	0.40	0.35	0.83
YOL026C		1020	772	1.32	0.40	0.35	0.83
YDR359C		2186	1655	1.32	0.40	0.35	0.83
YPL151C	PRP46	1851	1402	1.32	0.40	0.34	0.83

YDR381W	YRA1	1624	1231	1.32	0.40	0.34	0.83
YMR032W	HOF1	1167	885	1.32	0.40	0.34	0.83
YBR111C	YSA1	918	696	1.32	0.40	0.34	0.83
YOR337W	TEA1	2638	2001	1.32	0.40	0.34	0.83
YMR254C		1127	855	1.32	0.40	0.34	0.83
YER133W	GLC7	2405	1824	1.32	0.40	0.34	0.83
YNR041C	COQ2	832	631	1.32	0.40	0.34	0.83
YLR163C	MAS1	2957	2243	1.32	0.40	0.34	0.83
YOR271C		1222	927	1.32	0.40	0.34	0.83
YLR295C	ATP14	1103	837	1.32	0.40	0.34	0.83
YBR143C	SUP45	2819	2139	1.32	0.40	0.34	0.83
YKR059W	TIF1	3836	2911	1.32	0.40	0.34	0.83
YLR415C		538	408	1.32	0.40	0.34	0.83
YOR345C		523	397	1.32	0.40	0.34	0.83
YLR342W	FKS1	2499	1897	1.32	0.40	0.34	0.82
YHL049C		3492	2651	1.32	0.40	0.34	0.82
YGL198W		436	331	1.32	0.40	0.34	0.82
YKL073W	LHS1	602	457	1.32	0.40	0.34	0.82
YNL138W	SRV2	1268	963	1.32	0.40	0.34	0.82
YBR117C	TKL2	1526	1159	1.32	0.40	0.34	0.82
YGR281W	YOR1	2347	1783	1.32	0.40	0.34	0.82
YIL151C		728	553	1.32	0.40	0.34	0.82
YMR324C		2296	1745	1.32	0.40	0.34	0.82
YJL123C		4916	3737	1.32	0.40	0.34	0.82
YHL023C		2791	2122	1.32	0.40	0.34	0.82
YHR119W	SET1	1327	1009	1.32	0.40	0.34	0.82
YBL100C		5208	3961	1.31	0.39	0.34	0.82
YBR261C		3473	2642	1.31	0.39	0.34	0.82
YBR254C	TRS20	8504	6472	1.31	0.39	0.34	0.82
YKL070W		1281	975	1.31	0.39	0.34	0.82
YGR176W		4107	3127	1.31	0.39	0.34	0.81
YDL111C	RRP42	1865	1420	1.31	0.39	0.34	0.81
YLR453C	RIF2	1685	1284	1.31	0.39	0.34	0.81
YDRWdelta30	YDRWdelta30	407	310	1.31	0.39	0.34	0.81
YLR076C		1643	1252	1.31	0.39	0.34	0.81
YDR327W		518	395	1.31	0.39	0.34	0.81

YPL237W	SUI3	4291	3272	1.31	0.39	0.34	0.81
YNL182C		947	722	1.31	0.39	0.34	0.81
YKL151C		821	626	1.31	0.39	0.34	0.81
YPL243W	SRP68	2259	1723	1.31	0.39	0.33	0.81
YLR152C		1012	772	1.31	0.39	0.33	0.81
YHR205W	SCH9	1460	1114	1.31	0.39	0.33	0.81
YPR169W		2200	1679	1.31	0.39	0.33	0.81
YPR184W		1827	1395	1.31	0.39	0.33	0.80
YJR115W		707	540	1.31	0.39	0.33	0.80
YNL030W	HHF2	2057	1571	1.31	0.39	0.33	0.80
YHR216W	PUR5	5551	4239	1.31	0.39	0.33	0.80
YER049W		6556	5007	1.31	0.39	0.33	0.80
YNL166C	BNI5	999	763	1.31	0.39	0.33	0.80
YHR070W		2229	1703	1.31	0.39	0.33	0.80
YOL073C		2721	2079	1.31	0.39	0.33	0.80
YLR234W	TOP3	1751	1338	1.31	0.39	0.33	0.80
YFR009W	GCN20	3005	2297	1.31	0.39	0.33	0.80
YDL039C		1494	1143	1.31	0.39	0.33	0.80
YPR084W		1781	1363	1.31	0.39	0.33	0.80
YNL058C		759	581	1.31	0.39	0.33	0.80
YNL242W		966	740	1.31	0.38	0.33	0.79
YJL096W	MRPL49	433	332	1.31	0.38	0.33	0.79
YPL107W		634	486	1.31	0.38	0.33	0.79
YLR350W		4306	3299	1.31	0.38	0.33	0.79
YLR015W		1913	1466	1.31	0.38	0.33	0.79
YDL104C	QRI7	5640	4322	1.30	0.38	0.33	0.79
YOR136W	IDH2	1646	1261	1.30	0.38	0.33	0.79
YOR111W		1026	786	1.30	0.38	0.33	0.79
YDL160C	DHH1	3482	2669	1.30	0.38	0.33	0.79
YCR100C		1870	1433	1.30	0.38	0.33	0.79
YLR339C		2158	1654	1.30	0.38	0.33	0.79
YOR006C		3120	2392	1.30	0.38	0.33	0.79
YAL040C	CLN3	5638	4323	1.30	0.38	0.33	0.79
YBR080C	SEC18	5001	3836	1.30	0.38	0.33	0.79
YER103W	SSA4	2571	1972	1.30	0.38	0.33	0.79
YOR369C	RPS12	2862	2196	1.30	0.38	0.33	0.79

YNL025C	SSN8	3342	2564	1.30	0.38	0.33	0.79
YHR035W		2080	1596	1.30	0.38	0.33	0.79
YHR176W		769	590	1.30	0.38	0.33	0.79
YKL190W	CNB1	718	551	1.30	0.38	0.33	0.79
YKR084C	HBS1	1321	1014	1.30	0.38	0.33	0.79
YOR327C	SNC2	858	659	1.30	0.38	0.33	0.79
YGR165W		3194	2452	1.30	0.38	0.33	0.79
YHL019C	APM2	1116	857	1.30	0.38	0.33	0.79
YGR007W	MUQ1	1499	1151	1.30	0.38	0.33	0.78
YJR091C	JSN1	898	690	1.30	0.38	0.32	0.78
YGR058W		432	332	1.30	0.38	0.32	0.78
YPL006W	NCR1	4670	3588	1.30	0.38	0.32	0.78
YCR075C	ERS1	1007	774	1.30	0.38	0.32	0.78
YLR373C		1426	1096	1.30	0.38	0.32	0.78
YLR423C		739	568	1.30	0.38	0.32	0.78
YGL002W	ERP6	1271	978	1.30	0.38	0.32	0.78
YCL064C	CHA1	2440	1877	1.30	0.38	0.32	0.78
YHR024C	MAS2	2171	1671	1.30	0.38	0.32	0.78
YBR139W		3019	2324	1.30	0.38	0.32	0.78
YDL146W		1681	1294	1.30	0.38	0.32	0.78
YDR430C		3616	2784	1.30	0.38	0.32	0.78
YGR219W		987	760	1.30	0.38	0.32	0.77
YHL011C	PRS3	2816	2170	1.30	0.38	0.32	0.77
YBL081W		4664	3594	1.30	0.38	0.32	0.77
YDR364C	CDC40	363	280	1.30	0.38	0.32	0.77
YGR246C	BRF1	1562	1204	1.30	0.38	0.32	0.77
YMR086C-A		528	407	1.30	0.38	0.32	0.77
YMR192W		804	620	1.30	0.37	0.32	0.77
YML012W	ERV25	4416	3406	1.30	0.37	0.32	0.77
YOR233W	KIN4	2064	1593	1.30	0.37	0.32	0.77
YOL090W	MSH2	2138	1650	1.30	0.37	0.32	0.77
YKL106W	AAT1	1385	1069	1.30	0.37	0.32	0.77
YPL002C	SNF8	745	575	1.30	0.37	0.32	0.77
YBR196C	PGI1	7694	5941	1.30	0.37	0.32	0.77
YER034W		1700	1313	1.29	0.37	0.32	0.76
YHR130C		2223	1717	1.29	0.37	0.32	0.76

YLR451W	LEU3	2113	1632	1.29	0.37	0.32	0.76
YMR251W		1834	1417	1.29	0.37	0.32	0.76
YKL200C		678	524	1.29	0.37	0.32	0.76
YGL105W	ARC1	2985	2307	1.29	0.37	0.32	0.76
YGR180C	RNR4	6876	5315	1.29	0.37	0.32	0.76
YKL077W		1833	1417	1.29	0.37	0.32	0.76
YDR037W	KRS1	1706	1319	1.29	0.37	0.32	0.76
YNR030W	ECM39	2218	1715	1.29	0.37	0.32	0.76
YLR248W	RCK2	3429	2652	1.29	0.37	0.31	0.76
YFL002C	SPB4	1624	1256	1.29	0.37	0.31	0.76
YJR036C		517	400	1.29	0.37	0.31	0.76
YGL213C	SKI8	2918	2258	1.29	0.37	0.31	0.76
YLR109W	AHP1	866	670	1.29	0.37	0.31	0.76
YMR080C	NAM7	3582	2772	1.29	0.37	0.31	0.76
YCR044C		1524	1180	1.29	0.37	0.31	0.76
YNL301C	RPL18B	3694	2861	1.29	0.37	0.31	0.76
YFL053W	DAK2	1056	818	1.29	0.37	0.31	0.75
YIR034C	LYS1	2412	1869	1.29	0.37	0.31	0.75
YBL066C	SEF1	4047	3136	1.29	0.37	0.31	0.75
YFR035C		3737	2896	1.29	0.37	0.31	0.75
YMR083W	ADH3	1438	1115	1.29	0.37	0.31	0.75
YJL163C		5283	4095	1.29	0.37	0.31	0.75
YGR049W	SCM4	1784	1383	1.29	0.37	0.31	0.75
YOL005C	RPB11	539	418	1.29	0.37	0.31	0.75
YIL034C	CAP2	3277	2543	1.29	0.37	0.31	0.75
YLR347C	KAP95	3039	2359	1.29	0.37	0.31	0.75
YOR095C	RKI1	931	723	1.29	0.37	0.31	0.75
YJR062C	NTA1	1053	818	1.29	0.36	0.31	0.75
YNL272C	SEC2	1744	1354	1.29	0.36	0.31	0.75
YBR031W	RPL4A	2779	2158	1.29	0.36	0.31	0.75
YPR025C	CCL1	894	694	1.29	0.36	0.31	0.75
YNL292W	PUS4	1302	1011	1.29	0.36	0.31	0.74
YKL021C	MAK11	2147	1668	1.29	0.36	0.31	0.74
YDR417C		1190	925	1.29	0.36	0.31	0.74
YOR195W	SLK19	3224	2506	1.29	0.36	0.31	0.74
YOR056C		1449	1127	1.29	0.36	0.31	0.74

YCLX10C		1089	847	1.29	0.36	0.31	0.74
YML068W		463	360	1.28	0.36	0.31	0.74
YJR068W	RFC2	799	622	1.28	0.36	0.31	0.74
YKL207W		2180	1697	1.28	0.36	0.30	0.74
YNL306W		890	693	1.28	0.36	0.30	0.74
YLR242C	ARV1	639	498	1.28	0.36	0.30	0.73
YNL312W	RFA2	535	417	1.28	0.36	0.30	0.73
YNL175C		1343	1047	1.28	0.36	0.30	0.73
YDR143C	SAN1	3145	2452	1.28	0.36	0.30	0.73
YFL065C		1413	1102	1.28	0.36	0.30	0.73
YBR110W	ALG1	3220	2511	1.28	0.36	0.30	0.73
YNL109W		1371	1069	1.28	0.36	0.30	0.73
YGL050W		895	698	1.28	0.36	0.30	0.73
YOR003W	YSP3	1688	1317	1.28	0.36	0.30	0.73
YBR081C	SPT7	2967	2315	1.28	0.36	0.30	0.73
YDL055C	PSA1	4198	3276	1.28	0.36	0.30	0.73
YNR051C		6204	4842	1.28	0.36	0.30	0.73
YHL047C		2160	1686	1.28	0.36	0.30	0.73
YCR016W		4544	3548	1.28	0.36	0.30	0.73
YGLWtau2	YGLWtau2	488	381	1.28	0.36	0.30	0.73
YNL189W	SRP1	844	659	1.28	0.36	0.30	0.73
YHR080C		10383	8114	1.28	0.36	0.30	0.72
YMR202W	ERG2	3221	2517	1.28	0.36	0.30	0.72
YDL030W	PRP9	3123	2441	1.28	0.36	0.30	0.72
YER122C	GLO3	1857	1452	1.28	0.36	0.30	0.72
YOR284W		659	515	1.28	0.36	0.30	0.72
YGL253W	HXK2	3751	2933	1.28	0.35	0.30	0.72
YCR065W	HCM1	7301	5709	1.28	0.35	0.30	0.72
YNL037C	IDH1	1349	1055	1.28	0.35	0.30	0.72
YKR077W		1401	1096	1.28	0.35	0.30	0.72
YPL181W		1501	1175	1.28	0.35	0.30	0.72
YAL017W	FUN31	2858	2238	1.28	0.35	0.30	0.72
YIL153W	RRD1	469	367	1.28	0.35	0.30	0.72
YDL027C		1269	994	1.28	0.35	0.30	0.72
YKL017C	HCS1	924	724	1.28	0.35	0.30	0.71
YNL294C		1531	1200	1.28	0.35	0.29	0.71

YNL203C		2655	2082	1.28	0.35	0.29	0.71
YOL006C	TOP1	1922	1507	1.28	0.35	0.29	0.71
YDL144C		1277	1002	1.27	0.35	0.29	0.71
YGR123C	PPT1	1816	1425	1.27	0.35	0.29	0.71
YKL184W	SPE1	633	497	1.27	0.35	0.29	0.71
YPLCTy4-1B	YPLCTy4-1B	951	746	1.27	0.35	0.29	0.71
YNR053C		3233	2538	1.27	0.35	0.29	0.71
YDL182W	LYS20	2698	2118	1.27	0.35	0.29	0.71
YAR075W		5864	4605	1.27	0.35	0.29	0.71
YLL058W		1360	1068	1.27	0.35	0.29	0.71
YJR140C	HIR3	1511	1187	1.27	0.35	0.29	0.71
YFL066C		6253	4912	1.27	0.35	0.29	0.71
YDR091C	RLI1	2815	2213	1.27	0.35	0.29	0.70
YHL003C	LAG1	1483	1166	1.27	0.35	0.29	0.70
YER123W	YCK3	1662	1307	1.27	0.35	0.29	0.70
YOR270C	VPH1	1867	1469	1.27	0.35	0.29	0.70
YGR101W		1765	1389	1.27	0.35	0.29	0.70
YBR083W	TEC1	2563	2017	1.27	0.35	0.29	0.70
YBR037C	SCO1	467	368	1.27	0.34	0.29	0.70
YPL045W	VPS16	2729	2149	1.27	0.34	0.29	0.70
YAL045C		13302	10475	1.27	0.34	0.29	0.70
YML058C-A		1902	1498	1.27	0.34	0.29	0.70
YPR004C		443	349	1.27	0.34	0.29	0.70
YJR045C	SSC1	2296	1808	1.27	0.34	0.29	0.70
YGR254W	ENO1	7214	5682	1.27	0.34	0.29	0.70
YDR017C	KCS1	741	584	1.27	0.34	0.29	0.69
YER129W	PAK1	2548	2008	1.27	0.34	0.29	0.69
YIL128W	MET18	4883	3851	1.27	0.34	0.29	0.69
YPR080W	TEF1	12903	10176	1.27	0.34	0.29	0.69
YMR021C	MAC1	1212	956	1.27	0.34	0.29	0.69
YLR441C	RPS1A	1907	1505	1.27	0.34	0.29	0.69
YMR110C		1832	1446	1.27	0.34	0.29	0.69
YLL048C	YBT1	2025	1598	1.27	0.34	0.29	0.69
YGR199W	PMT6	1872	1478	1.27	0.34	0.28	0.69
YDR403W	DIT1	441	348	1.27	0.34	0.28	0.69
YDR096W	GIS1	855	675	1.27	0.34	0.28	0.69

YDR350C	TCM10	1636	1292	1.27	0.34	0.28	0.69
YAL013W	DEP1	1058	836	1.27	0.34	0.28	0.69
YDR233C		2339	1848	1.27	0.34	0.28	0.69
YBR118W	TEF2	10985	8678	1.27	0.34	0.28	0.69
YDR382W	RPP2B	2332	1843	1.27	0.34	0.28	0.68
YMR093W		1603	1267	1.27	0.34	0.28	0.68
YNR073C		366	289	1.27	0.34	0.28	0.68
YBR265W	TSC10	1931	1527	1.26	0.34	0.28	0.68
YOL044W	PEX15	599	474	1.26	0.34	0.28	0.68
YPL204W	HRR25	5824	4606	1.26	0.34	0.28	0.68
YOL070C		3159	2499	1.26	0.34	0.28	0.68
YIL156W	UBP7	1552	1228	1.26	0.34	0.28	0.68
YDR411C		662	524	1.26	0.34	0.28	0.68
YDR410C	STE14	2189	1732	1.26	0.34	0.28	0.68
YPL099C		980	775	1.26	0.34	0.28	0.68
YLR435W		1244	984	1.26	0.34	0.28	0.68
YOL120C	RPL18A	4012	3175	1.26	0.34	0.28	0.68
YLR259C	HSP60	3595	2846	1.26	0.34	0.28	0.68
YML041C		2141	1695	1.26	0.34	0.28	0.68
YBR238C		1848	1463	1.26	0.34	0.28	0.68
YPL139C	UME1	1267	1003	1.26	0.34	0.28	0.68
YER178W	PDA1	3631	2877	1.26	0.34	0.28	0.68
YGR272C		2257	1789	1.26	0.34	0.28	0.67
YBR176W	ECM31	1120	888	1.26	0.33	0.28	0.67
YLR274W	CDC46	1030	817	1.26	0.33	0.28	0.67
YML020W		6052	4799	1.26	0.33	0.28	0.67
YPL145C	KES1	2163	1715	1.26	0.33	0.28	0.67
YHL045W		1018	808	1.26	0.33	0.28	0.67
YML118W		929	737	1.26	0.33	0.28	0.67
YPL197C		1890	1500	1.26	0.33	0.28	0.67
YPL133C		683	542	1.26	0.33	0.28	0.67
YPL279C		1090	865	1.26	0.33	0.28	0.67
YMR316C-B		2089	1658	1.26	0.33	0.28	0.67
YMR189W	GCV2	1564	1242	1.26	0.33	0.28	0.67
YDR269C		501	398	1.26	0.33	0.28	0.67
YKL101W	HSL1	3393	2695	1.26	0.33	0.28	0.67

YMR036C	MIH1	540	429	1.26	0.33	0.28	0.67
YJL002C	OST1	2778	2207	1.26	0.33	0.28	0.67
YJL174W	KRE9	2596	2063	1.26	0.33	0.28	0.67
YJL143W	TIM17	1895	1506	1.26	0.33	0.28	0.66
YML113W	DAT1	676	537	1.26	0.33	0.28	0.66
YJR074W	MOG1	1127	896	1.26	0.33	0.28	0.66
YKL145W	RPT1	4381	3482	1.26	0.33	0.28	0.66
YOR063W	RPL3	2809	2233	1.26	0.33	0.28	0.66
YGR151C		940	747	1.26	0.33	0.27	0.66
YMR288W		1589	1263	1.26	0.33	0.27	0.66
YHR060W	VMA22	716	569	1.26	0.33	0.27	0.66
YBR049C	REB1	1988	1581	1.26	0.33	0.27	0.66
YKL213C	DOA1	3336	2654	1.26	0.33	0.27	0.66
YDL147W	RPN5	8431	6709	1.26	0.33	0.27	0.66
YFL034W		2465	1962	1.26	0.33	0.27	0.66
YGL003C	CDH1	2517	2004	1.26	0.33	0.27	0.66
YNR050C	LYS9	1253	998	1.26	0.33	0.27	0.66
YNL274C		511	407	1.26	0.33	0.27	0.66
YGL147C	RPL9A	5851	4661	1.26	0.33	0.27	0.66
YDL117W		2275	1813	1.25	0.33	0.27	0.66
YAR066W		2789	2224	1.25	0.33	0.27	0.65
YEL059C-A	SOM1	565	451	1.25	0.33	0.27	0.65
YCL044C		3114	2484	1.25	0.33	0.27	0.65
YGR257C		1391	1110	1.25	0.33	0.27	0.65
YBL023C	MCM2	1689	1348	1.25	0.33	0.27	0.65
YNL280C	ERG24	1187	947	1.25	0.33	0.27	0.65
YOL130W	ALR1	2091	1669	1.25	0.33	0.27	0.65
YGR004W		4882	3897	1.25	0.33	0.27	0.65
YDL087C	LUC7	1457	1163	1.25	0.32	0.27	0.65
YHR209W		2954	2359	1.25	0.32	0.27	0.65
YKL081W	TEF4	2559	2043	1.25	0.32	0.27	0.65
YFR007W		918	733	1.25	0.32	0.27	0.65
YFR013W		2548	2035	1.25	0.32	0.27	0.65
YPR014C		1384	1106	1.25	0.32	0.27	0.65
YJL055W		1960	1567	1.25	0.32	0.27	0.64
YJR076C	CDC11	988	790	1.25	0.32	0.27	0.64

YLR455W		2770	2216	1.25	0.32	0.27	0.64
YPL057C	SUR1	2583	2066	1.25	0.32	0.27	0.64
YOR204W	DED1	3979	3183	1.25	0.32	0.27	0.64
YKR106W		1509	1207	1.25	0.32	0.27	0.64
YGL011C	SCL1	2287	1830	1.25	0.32	0.27	0.64
YDL053C		1979	1583	1.25	0.32	0.27	0.64
YGR005C	TFG2	975	780	1.25	0.32	0.27	0.64
YHR105W		1053	843	1.25	0.32	0.27	0.64
YIR002C	MPH1	2987	2391	1.25	0.32	0.27	0.64
YLR004C		1070	857	1.25	0.32	0.26	0.64
YHR026W	PPA1	1813	1452	1.25	0.32	0.26	0.64
YFL006W		648	519	1.25	0.32	0.26	0.64
YKL029C	MAE1	3687	2954	1.25	0.32	0.26	0.64
YBR113W		2839	2275	1.25	0.32	0.26	0.64
YCR012W	PGK1	4668	3740	1.25	0.32	0.26	0.64
YALCdelta2	YALCdelta2	1027	823	1.25	0.32	0.26	0.64
YLR060W	FRS1	5158	4136	1.25	0.32	0.26	0.63
YDL248W	COS7	874	701	1.25	0.32	0.26	0.63
YGL222C		541	434	1.25	0.32	0.26	0.63
YGR255C	COQ6	3092	2480	1.25	0.32	0.26	0.63
YML023C		712	571	1.25	0.32	0.26	0.63
YER048C	CAJ1	1468	1177	1.25	0.32	0.26	0.63
YOL055C	THI20	1362	1093	1.25	0.32	0.26	0.63
YKR104W		727	583	1.25	0.32	0.26	0.63
YCL040W	GLK1	2786	2236	1.25	0.32	0.26	0.63
YGL014W		1339	1075	1.25	0.32	0.26	0.63
YGR103W		5890	4728	1.25	0.32	0.26	0.63
YILWTy3-1B	YILWTy3-1B	3332	2677	1.24	0.32	0.26	0.63
YPR035W	GLN1	2714	2180	1.24	0.32	0.26	0.63
YCL014W	BUD3	1736	1395	1.24	0.32	0.26	0.63
YMR198W	CIK1	1036	833	1.24	0.32	0.26	0.63
YIL137C		2395	1925	1.24	0.32	0.26	0.63
YBR149W	ARA1	3654	2938	1.24	0.31	0.26	0.62
YDR068W	DOS2	2986	2401	1.24	0.31	0.26	0.62
YBR266C		4283	3445	1.24	0.31	0.26	0.62
YJR070C		3992	3212	1.24	0.31	0.26	0.62

YDR079W	PET100	2551	2053	1.24	0.31	0.26	0.62
YKL162C		1216	979	1.24	0.31	0.26	0.62
YER120W	SCS2	5690	4582	1.24	0.31	0.26	0.62
YKL010C	UFD4	3932	3167	1.24	0.31	0.26	0.62
YJL222W	VTH2	3417	2753	1.24	0.31	0.26	0.62
YOR340C	RPA43	2533	2041	1.24	0.31	0.26	0.62
YGL238W	CSE1	3157	2544	1.24	0.31	0.26	0.62
YER055C	HIS1	961	775	1.24	0.31	0.25	0.62
YGR111W		982	792	1.24	0.31	0.25	0.61
YJR039W		466	376	1.24	0.31	0.25	0.61
YFL005W	SEC4	2138	1725	1.24	0.31	0.25	0.61
YBL064C		4309	3480	1.24	0.31	0.25	0.61
YPR074C	TKL1	4259	3440	1.24	0.31	0.25	0.61
YOL139C	CDC33	3124	2524	1.24	0.31	0.25	0.61
YLR459W	CDC91	624	504	1.24	0.31	0.25	0.61
YKR073C		513	415	1.24	0.31	0.25	0.61
YDL167C	NRP1	7255	5865	1.24	0.31	0.25	0.61
YLR220W	CCC1	2129	1721	1.24	0.31	0.25	0.61
YGR253C	PUP2	2546	2059	1.24	0.31	0.25	0.60
YBR059C	AKL1	3187	2577	1.24	0.31	0.25	0.60
YBR232C		5257	4252	1.24	0.31	0.25	0.60
YPR107C	YTH1	706	571	1.24	0.31	0.25	0.60
YDR343C	HXT6	1178	953	1.24	0.31	0.25	0.60
YPL261C		532	430	1.24	0.31	0.25	0.60
YNL176C		2837	2295	1.24	0.31	0.25	0.60
YOR179C		777	629	1.24	0.31	0.25	0.60
YGR149W		1985	1606	1.24	0.31	0.25	0.60
YER036C		2410	1950	1.24	0.31	0.25	0.60
YDR076W	RAD55	489	396	1.24	0.30	0.25	0.60
YOR123C	LEO1	857	694	1.24	0.30	0.25	0.60
YBR002C	RER2	3940	3190	1.24	0.30	0.25	0.60
YAL035W	FUN12	4836	3916	1.23	0.30	0.25	0.60
YOL068C	HST1	1614	1307	1.23	0.30	0.25	0.60
YPR136C		2173	1760	1.23	0.30	0.25	0.60
YGL187C	COX4	2936	2378	1.23	0.30	0.25	0.60
YDR033W	MRH1	3125	2531	1.23	0.30	0.25	0.60

YJR015W		1190	964	1.23	0.30	0.25	0.60
YGL010W		2305	1868	1.23	0.30	0.25	0.60
YPLCTy4-1A	YPLCTy4-1A	515	417	1.23	0.30	0.25	0.60
YOR053W		2022	1639	1.23	0.30	0.25	0.60
YCR072C		3084	2500	1.23	0.30	0.25	0.60
YGR121C	MEP1	2631	2133	1.23	0.30	0.25	0.60
YJL149W		990	803	1.23	0.30	0.25	0.60
YPL050C	MNN9	5762	4672	1.23	0.30	0.25	0.60
YNL052W	COX5A	2219	1800	1.23	0.30	0.25	0.59
YER182W		943	765	1.23	0.30	0.25	0.59
YMR119W		1367	1109	1.23	0.30	0.25	0.59
YGL171W	ROK1	2343	1901	1.23	0.30	0.25	0.59
YBL099W	ATP1	1725	1400	1.23	0.30	0.25	0.59
YJR030C		1043	846	1.23	0.30	0.25	0.59
YGL023C	PIB2	3135	2544	1.23	0.30	0.25	0.59
YKR048C	NAP1	2952	2396	1.23	0.30	0.24	0.59
YPL019C		3539	2873	1.23	0.30	0.24	0.59
YDR253C	MET32	427	347	1.23	0.30	0.24	0.59
YPL018W	CTF19	1152	935	1.23	0.30	0.24	0.59
YNR064C		2165	1758	1.23	0.30	0.24	0.59
YBL014C	RRN6	6083	4940	1.23	0.30	0.24	0.59
YPR055W	SEC8	944	767	1.23	0.30	0.24	0.59
YOR317W	FAA1	1322	1074	1.23	0.30	0.24	0.59
YCLX06C		2173	1766	1.23	0.30	0.24	0.59
YNL180C	RHO5	2539	2064	1.23	0.30	0.24	0.59
YGR210C		3269	2658	1.23	0.30	0.24	0.59
YOR032C	HMS1	783	637	1.23	0.30	0.24	0.59
YKL046C		5072	4125	1.23	0.30	0.24	0.58
YFR048W		1910	1553	1.23	0.30	0.24	0.58
YIL093C		637	518	1.23	0.30	0.24	0.58
YGL248W	PDE1	1601	1302	1.23	0.30	0.24	0.58
YDL195W	SEC31	8759	7126	1.23	0.30	0.24	0.58
YBLCsigma1	YBLCsigma1	690	562	1.23	0.30	0.24	0.58
YDL226C	GCS1	2651	2159	1.23	0.30	0.24	0.58
YML109W	ZDS2	1505	1226	1.23	0.30	0.24	0.58
YGR166W	KRE11	800	652	1.23	0.30	0.24	0.58

YLR165C		3009	2452	1.23	0.30	0.24	0.58
YDL179W	PCL9	2298	1873	1.23	0.30	0.24	0.58
YNL065W		1489	1214	1.23	0.29	0.24	0.58
YOR269W	PAC1	1504	1226	1.23	0.29	0.24	0.58
YIL022W	TIM44	3763	3068	1.23	0.29	0.24	0.58
YOLWdelta6	YOLWdelta6	2448	1996	1.23	0.29	0.24	0.58
YOR046C	DBP5	3750	3058	1.23	0.29	0.24	0.58
YLR262C	YPT6	1162	948	1.23	0.29	0.24	0.57
YBL007C	SLA1	2624	2140	1.23	0.29	0.24	0.57
YJL092W	HPR5	680	555	1.23	0.29	0.24	0.57
YDR379W	RGA2	1268	1034	1.23	0.29	0.24	0.57
YDR362C	TFC6	1677	1368	1.23	0.29	0.24	0.57
YNL319W		1096	894	1.23	0.29	0.24	0.57
YPR118W		1297	1058	1.23	0.29	0.24	0.57
YOR250C	CLP1	401	327	1.23	0.29	0.24	0.57
YGR096W		1809	1476	1.23	0.29	0.24	0.57
YBR251W	MRPS5	2850	2326	1.23	0.29	0.24	0.57
YPR160W	GPH1	1569	1281	1.23	0.29	0.24	0.57
YAL067C	SEO1	524	428	1.22	0.29	0.24	0.57
YJL048C		736	601	1.22	0.29	0.24	0.57
YHR028C	DAP2	851	695	1.22	0.29	0.24	0.57
YGR285C	ZUO1	3766	3075	1.22	0.29	0.24	0.57
YIR006C	PAN1	1742	1423	1.22	0.29	0.24	0.57
YER110C	KAP123	3394	2772	1.22	0.29	0.24	0.57
YDL137W	ARF2	4826	3942	1.22	0.29	0.24	0.57
YDR179C		662	541	1.22	0.29	0.24	0.57
YPL090C	RPS6A	5087	4155	1.22	0.29	0.24	0.57
YBR048W	RPS11B	2929	2393	1.22	0.29	0.24	0.57
YKL087C	CYT2	1356	1108	1.22	0.29	0.24	0.57
YJL133W	MRS3	808	660	1.22	0.29	0.24	0.57
YJR042W	NUP85	1616	1321	1.22	0.29	0.24	0.57
YPL025C		700	572	1.22	0.29	0.23	0.57
YBR024W	SCO2	1406	1149	1.22	0.29	0.23	0.57
YHR029C		1656	1354	1.22	0.29	0.23	0.57
YCL052C	PBN1	2020	1651	1.22	0.29	0.23	0.57
YDL043C	PRP11	840	687	1.22	0.29	0.23	0.57

YEL046C	GLY1	1217	995	1.22	0.29	0.23	0.57
YNL150W		1596	1305	1.22	0.29	0.23	0.57
YOL156W	HXT11	1878	1536	1.22	0.29	0.23	0.56
YJR110W		1127	922	1.22	0.29	0.23	0.56
YGL228W	SHE10	2382	1949	1.22	0.29	0.23	0.56
YLL026W	HSP104	5982	4896	1.22	0.29	0.23	0.56
YDL191W	RPL35A	2452	2007	1.22	0.29	0.23	0.56
YLR243W		1297	1062	1.22	0.29	0.23	0.56
YGL098W		1470	1204	1.22	0.29	0.23	0.56
YOR001W	RRP6	2513	2058	1.22	0.29	0.23	0.56
YPR155C	NCA2	889	728	1.22	0.29	0.23	0.56
YOL089C	HAL9	2536	2077	1.22	0.29	0.23	0.56
YPL122C	TFB2	2991	2450	1.22	0.29	0.23	0.56
YMR183C	SSO2	3497	2865	1.22	0.29	0.23	0.56
YKL049C	CSE4	964	790	1.22	0.29	0.23	0.56
YDL201W		3228	2646	1.22	0.29	0.23	0.56
YMR272C	SCS7	1638	1343	1.22	0.29	0.23	0.56
YJL076W	NET1	2787	2285	1.22	0.29	0.23	0.56
YJR046W	TAH11	832	682	1.22	0.29	0.23	0.56
YOL144W	NOP8	1055	865	1.22	0.29	0.23	0.56
YFR024C-A		538	441	1.22	0.29	0.23	0.55
YPR133C		361	296	1.22	0.29	0.23	0.55
YLR014C	PPR1	921	756	1.22	0.29	0.23	0.55
YHR183W	GND1	850	698	1.22	0.28	0.23	0.55
YBL051C		2585	2123	1.22	0.28	0.23	0.55
YIL169C		4969	4081	1.22	0.28	0.23	0.55
YJL213W		2397	1969	1.22	0.28	0.23	0.55
YIR022W	SEC11	666	547	1.22	0.28	0.23	0.55
YGR054W		2226	1828	1.22	0.28	0.23	0.55
YBR221C	PDB1	5874	4826	1.22	0.28	0.23	0.55
YGL035C	MIG1	2457	2019	1.22	0.28	0.23	0.55
YGL031C	RPL24A	4790	3937	1.22	0.28	0.23	0.55
YIL091C		2537	2085	1.22	0.28	0.23	0.55
YCL038C		2143	1762	1.22	0.28	0.23	0.55
YMR266W		1923	1581	1.22	0.28	0.23	0.55
YNL282W	POP3	1432	1178	1.22	0.28	0.23	0.55

YDR389W	SAC7	3789	3116	1.22	0.28	0.23	0.55
YLR309C	IMH1	3521	2896	1.22	0.28	0.23	0.55
YIL097W		598	492	1.22	0.28	0.23	0.55
YPL029W	SUV3	4393	3614	1.22	0.28	0.23	0.54
YGL078C	DBP3	1277	1051	1.22	0.28	0.23	0.54
YNL168C		2473	2035	1.22	0.28	0.22	0.54
YDR423C	CAD1	1080	889	1.21	0.28	0.22	0.54
YAL043C	PTA1	1454	1197	1.21	0.28	0.22	0.54
YDR028C	REG1	946	779	1.21	0.28	0.22	0.54
YGL062W	PYC1	476	392	1.21	0.28	0.22	0.54
YCR042C	TSM1	2142	1765	1.21	0.28	0.22	0.54
YNL270C	ALP1	1113	917	1.21	0.28	0.22	0.54
YGR238C	KEL2	3159	2604	1.21	0.28	0.22	0.54
YDL127W	PCL2	3162	2606	1.21	0.28	0.22	0.54
YPL100W		653	538	1.21	0.28	0.22	0.54
YGR234W	YHB1	5412	4462	1.21	0.28	0.22	0.54
YMR214W	SCJ1	7908	6520	1.21	0.28	0.22	0.54
YCL008C	STP22	3637	3000	1.21	0.28	0.22	0.54
YNL116W		1831	1510	1.21	0.28	0.22	0.54
YKL180W	RPL17A	3836	3164	1.21	0.28	0.22	0.54
YER107C	GLE2	1022	843	1.21	0.28	0.22	0.54
YMR245W		1154	952	1.21	0.28	0.22	0.53
YHR039C		1323	1092	1.21	0.28	0.22	0.53
YGL091C	NBP35	2728	2251	1.21	0.28	0.22	0.53
YNL314W	DAL82	1400	1155	1.21	0.28	0.22	0.53
YLR154C		2845	2349	1.21	0.28	0.22	0.53
YEL025C		3861	3188	1.21	0.28	0.22	0.53
YBR203W		1040	859	1.21	0.28	0.22	0.53
YGL068W		860	710	1.21	0.28	0.22	0.53
YLR057W		1317	1088	1.21	0.28	0.22	0.53
YMR321C		793	655	1.21	0.28	0.22	0.53
YNL211C		687	567	1.21	0.28	0.22	0.53
YOL102C	TPT1	489	404	1.21	0.28	0.22	0.53
YLR011W		1956	1616	1.21	0.28	0.22	0.53
YNL078W		1231	1018	1.21	0.27	0.22	0.53
YDR365C		5088	4208	1.21	0.27	0.22	0.53

YOR248W		1629	1347	1.21	0.27	0.22	0.53
YIR044C		1623	1342	1.21	0.27	0.22	0.53
YKR075C		789	653	1.21	0.27	0.22	0.53
YLR135W		3645	3015	1.21	0.27	0.22	0.53
YOR205C		2173	1798	1.21	0.27	0.22	0.53
YLR186W		1346	1114	1.21	0.27	0.22	0.52
YPL061W	ALD6	627	519	1.21	0.27	0.22	0.52
YGL159W		1187	982	1.21	0.27	0.22	0.52
YBL097W	BRN1	2213	1832	1.21	0.27	0.22	0.52
YML015C	TAF40	977	809	1.21	0.27	0.22	0.52
YJR104C	SOD1	1440	1192	1.21	0.27	0.22	0.52
YFL064C		3789	3137	1.21	0.27	0.22	0.52
YOL096C	COQ3	407	337	1.21	0.27	0.22	0.52
YBR171W	SEC66	697	578	1.21	0.27	0.21	0.52
YDR387C		1363	1130	1.21	0.27	0.21	0.52
YOR214C		656	544	1.21	0.27	0.21	0.52
YER022W	SRB4	1274	1056	1.21	0.27	0.21	0.52
YNL221C	POP1	1587	1316	1.21	0.27	0.21	0.52
YDR395W	SXM1	2527	2095	1.21	0.27	0.21	0.52
YBL035C	POL12	1856	1541	1.20	0.27	0.21	0.51
YMR262W		2603	2162	1.20	0.27	0.21	0.51
YJL129C	TRK1	1780	1479	1.20	0.27	0.21	0.51
YDR110W	FOB1	1367	1136	1.20	0.27	0.21	0.51
YNL111C	CYB5	4830	4015	1.20	0.27	0.21	0.51
YFR044C		3149	2618	1.20	0.27	0.21	0.51
YML025C		1073	892	1.20	0.27	0.21	0.51
YLR249W	YEF3	5534	4602	1.20	0.27	0.21	0.51
YFL008W	SMC1	2938	2443	1.20	0.27	0.21	0.51
YOR131C		516	429	1.20	0.27	0.21	0.51
YIL011W		3940	3277	1.20	0.27	0.21	0.51
YDR279W		1367	1137	1.20	0.27	0.21	0.51
YNL254C		1837	1528	1.20	0.27	0.21	0.51
YPL226W		5065	4214	1.20	0.27	0.21	0.51
YGL190C	CDC55	2883	2399	1.20	0.27	0.21	0.50
YEL014C		1084	902	1.20	0.26	0.21	0.50
YDR478W	SNM1	1816	1512	1.20	0.26	0.21	0.50

YMR060C	TOM37	4980	4146	1.20	0.26	0.21	0.50
YOR360C	PDE2	1428	1189	1.20	0.26	0.21	0.50
YNL013C		4389	3656	1.20	0.26	0.21	0.50
YLR398C	SKI2	4722	3934	1.20	0.26	0.21	0.50
YKL085W	MDH1	4974	4145	1.20	0.26	0.21	0.50
YKL096W	CWP1	3751	3126	1.20	0.26	0.21	0.50
YLR304C	ACO1	4544	3789	1.20	0.26	0.21	0.50
YLR333C	RPS25B	1230	1026	1.20	0.26	0.21	0.50
YGL042C		2677	2233	1.20	0.26	0.21	0.50
YMR244C-A		2360	1969	1.20	0.26	0.21	0.50
YEL032W	MCM3	2230	1861	1.20	0.26	0.21	0.50
YJR144W	MGM101	1168	975	1.20	0.26	0.21	0.50
YJR040W	GEF1	2418	2018	1.20	0.26	0.20	0.49
YPR191W	QCR2	3867	3228	1.20	0.26	0.20	0.49
YPR113W	PIS1	2498	2085	1.20	0.26	0.20	0.49
YNL244C	SUI1	448	374	1.20	0.26	0.20	0.49
YOR349W	CIN1	780	651	1.20	0.26	0.20	0.49
YLR144C	ACF2	667	557	1.20	0.26	0.20	0.49
YFL028C	CAF16	1666	1391	1.20	0.26	0.20	0.49
YNL048W	ALG11	664	554	1.20	0.26	0.20	0.49
YJR007W	SUI2	1831	1529	1.20	0.26	0.20	0.49
YDL011C		1182	987	1.20	0.26	0.20	0.49
YGL076C	RPL7A	2578	2153	1.20	0.26	0.20	0.49
YER142C	MAG1	1068	892	1.20	0.26	0.20	0.49
YCR104W	PAU3	641	535	1.20	0.26	0.20	0.49
YOL061W	PRS5	2583	2158	1.20	0.26	0.20	0.49
YOL140W	ARG8	1940	1621	1.20	0.26	0.20	0.49
YNL062C	GCD10	1923	1607	1.20	0.26	0.20	0.49
YIL143C	SSL2	2504	2093	1.20	0.26	0.20	0.49
YOL017W		791	661	1.20	0.26	0.20	0.49
YOR242C	SSP2	690	577	1.20	0.26	0.20	0.49
YHR083W		3536	2956	1.20	0.26	0.20	0.49
YLR070C		1859	1554	1.20	0.26	0.20	0.49
YEL076C		6060	5067	1.20	0.26	0.20	0.49
YLR399C	BDF1	3421	2860	1.20	0.26	0.20	0.49
YBR108W		5514	4611	1.20	0.26	0.20	0.49

YML037C		1451	1213	1.20	0.26	0.20	0.49
YLR139C	SLS1	1901	1590	1.20	0.26	0.20	0.49
YCL059C	KRR1	5720	4785	1.20	0.26	0.20	0.49
YDL134C	PPH21	1400	1171	1.20	0.26	0.20	0.49
YCL011C	GBP2	4536	3795	1.20	0.26	0.20	0.49
YDR213W		3511	2938	1.20	0.26	0.20	0.49
YOR381W	FRE3	1081	905	1.19	0.26	0.20	0.48
YNL307C	MCK1	5219	4368	1.19	0.26	0.20	0.48
YOR372C	NDD1	2875	2406	1.19	0.26	0.20	0.48
YOR133W	EFT1	5537	4635	1.19	0.26	0.20	0.48
YBR181C	RPS6B	6134	5135	1.19	0.26	0.20	0.48
YEL020C		3270	2739	1.19	0.26	0.20	0.48
YOR112W		1028	861	1.19	0.26	0.20	0.48
YPR069C	SPE3	850	712	1.19	0.26	0.20	0.48
YOL136C	PFK27	1924	1612	1.19	0.26	0.20	0.48
YGR048W	UFD1	1589	1331	1.19	0.26	0.20	0.48
YPR164W	KIM3	2839	2379	1.19	0.26	0.20	0.48
YGL028C	SCW11	2881	2414	1.19	0.25	0.20	0.48
YDR026C		2755	2309	1.19	0.25	0.20	0.48
YGR055W	MUP1	1620	1358	1.19	0.25	0.20	0.48
YMR314W	PRE5	5485	4598	1.19	0.25	0.20	0.48
YLR214W	FRE1	5184	4346	1.19	0.25	0.20	0.48
YJR078W		318	267	1.19	0.25	0.20	0.48
YLR321C	SFH1	1297	1088	1.19	0.25	0.20	0.48
YDL247W		2504	2101	1.19	0.25	0.20	0.48
YHR106W	TRR2	2803	2352	1.19	0.25	0.20	0.48
YKL038W	RGT1	1202	1009	1.19	0.25	0.20	0.48
YKL097W-A		2401	2015	1.19	0.25	0.20	0.48
YDR352W		1707	1433	1.19	0.25	0.20	0.47
YHR008C	SOD2	2147	1802	1.19	0.25	0.20	0.47
YGL182C		1257	1055	1.19	0.25	0.20	0.47
YKR044W		1847	1550	1.19	0.25	0.20	0.47
YBL067C	UBP13	3869	3248	1.19	0.25	0.20	0.47
YLR023C		1299	1091	1.19	0.25	0.20	0.47
YLR382C	NAM2	1078	905	1.19	0.25	0.20	0.47
YLL051C	FRE6	2542	2135	1.19	0.25	0.20	0.47

YAL058C-A		1894	1591	1.19	0.25	0.20	0.47
YDR272W	GLO2	553	465	1.19	0.25	0.19	0.47
YOR231W	MKK1	2195	1845	1.19	0.25	0.19	0.47
YLR241W		1349	1134	1.19	0.25	0.19	0.47
YMR320W		2697	2267	1.19	0.25	0.19	0.47
YMR270C	RRN9	805	677	1.19	0.25	0.19	0.47
YCR094W	CDC50	3870	3254	1.19	0.25	0.19	0.47
YAL037W		2199	1849	1.19	0.25	0.19	0.47
YJL185C		1730	1455	1.19	0.25	0.19	0.47
YDR323C	PEP7	1762	1482	1.19	0.25	0.19	0.47
YJL061W	NUP82	1756	1477	1.19	0.25	0.19	0.47
YDL198C	YHM1	2011	1692	1.19	0.25	0.19	0.47
YOR007C	SGT2	1745	1468	1.19	0.25	0.19	0.47
YOR157C	PUP1	1486	1250	1.19	0.25	0.19	0.47
YNL014W		2195	1847	1.19	0.25	0.19	0.47
YER170W	ADK2	644	542	1.19	0.25	0.19	0.47
YGL095C	VPS45	2709	2280	1.19	0.25	0.19	0.46
YOL128C		438	369	1.19	0.25	0.19	0.46
YBL032W		4590	3865	1.19	0.25	0.19	0.46
YPR033C	HTS1	2356	1984	1.19	0.25	0.19	0.46
YHL043W	ECM34	860	724	1.19	0.25	0.19	0.46
YBR192W	RIM2	1585	1335	1.19	0.25	0.19	0.46
YOR320C		1635	1377	1.19	0.25	0.19	0.46
YJL073W	JEM1	2141	1804	1.19	0.25	0.19	0.46
YJR122W	CAF17	587	495	1.19	0.25	0.19	0.46
YER007C-A		1286	1084	1.19	0.25	0.19	0.46
YDR467C		1285	1083	1.19	0.25	0.19	0.46
YIL176C		1803	1520	1.19	0.25	0.19	0.46
YGL137W	SEC27	5452	4597	1.19	0.25	0.19	0.46
YDL131W	LYS21	2652	2236	1.19	0.25	0.19	0.46
YHR188C		1842	1553	1.19	0.25	0.19	0.46
YDR399W	HPT1	745	628	1.19	0.25	0.19	0.46
YGR216C	GPI1	3319	2799	1.19	0.25	0.19	0.46
YER119C		724	611	1.19	0.24	0.19	0.46
YMR097C		780	658	1.19	0.24	0.19	0.46
YKR021W		1110	937	1.18	0.24	0.19	0.46

YHR074W		719	607	1.18	0.24	0.19	0.46
YOR290C	SNF2	440	371	1.18	0.24	0.19	0.46
YNL036W	NCE103	1636	1381	1.18	0.24	0.19	0.45
YGR033C		2055	1735	1.18	0.24	0.19	0.45
YLR027C	AAT2	3564	3009	1.18	0.24	0.19	0.45
YCL018W	LEU2	1277	1079	1.18	0.24	0.19	0.45
YLR021W		1374	1161	1.18	0.24	0.19	0.45
YDR132C		1309	1106	1.18	0.24	0.19	0.45
YDL064W	UBC9	2583	2183	1.18	0.24	0.19	0.45
YBL112C		5081	4295	1.18	0.24	0.19	0.45
YPR106W	ISR1	664	561	1.18	0.24	0.19	0.45
YNL094W		1066	901	1.18	0.24	0.19	0.45
YLR044C	PDC1	5927	5012	1.18	0.24	0.19	0.45
YPR047W	MSF1	1360	1150	1.18	0.24	0.19	0.45
YCL043C	PDI1	4610	3899	1.18	0.24	0.19	0.45
YLR414C		400	338	1.18	0.24	0.19	0.45
YGL112C	TAF60	745	630	1.18	0.24	0.19	0.45
YPL082C	MOT1	6300	5331	1.18	0.24	0.18	0.45
YKL161C		1030	872	1.18	0.24	0.18	0.45
YOR207C	RET1	1959	1658	1.18	0.24	0.18	0.45
YCL063W		1567	1326	1.18	0.24	0.18	0.45
YKL088W		1070	906	1.18	0.24	0.18	0.45
YNL279W		865	732	1.18	0.24	0.18	0.44
YEL041W		1242	1052	1.18	0.24	0.18	0.44
YAR029W		1406	1191	1.18	0.24	0.18	0.44
YAR052C		1357	1150	1.18	0.24	0.18	0.44
YBR021W	FUR4	650	551	1.18	0.24	0.18	0.44
YML004C	GLO1	2040	1729	1.18	0.24	0.18	0.44
YNR020C		2829	2397	1.18	0.24	0.18	0.44
YBR090C-A		3241	2747	1.18	0.24	0.18	0.44
YHR206W	SKN7	1896	1607	1.18	0.24	0.18	0.44
YDR095C		1665	1411	1.18	0.24	0.18	0.44
YDR493W		461	391	1.18	0.24	0.18	0.44
YDL106C	GRF10	1415	1200	1.18	0.24	0.18	0.44
YOR050C		680	577	1.18	0.24	0.18	0.44
YOR034C	AKR2	1494	1267	1.18	0.24	0.18	0.44

YDR295C		994	843	1.18	0.24	0.18	0.44
YPL077C		1129	957	1.18	0.24	0.18	0.44
YKL089W	MIF2	928	787	1.18	0.24	0.18	0.44
YLL013C		2988	2536	1.18	0.24	0.18	0.44
YOR344C	TYE7	2284	1938	1.18	0.24	0.18	0.44
YLL052C	AQY2	3356	2849	1.18	0.24	0.18	0.44
YML010W	SPT5	2769	2351	1.18	0.24	0.18	0.43
YKL110C	KTI12	1729	1468	1.18	0.24	0.18	0.43
YJL169W		1685	1431	1.18	0.24	0.18	0.43
YOR367W	SCP1	2285	1940	1.18	0.24	0.18	0.43
YHR007C	ERG11	9024	7666	1.18	0.24	0.18	0.43
YOR079C	ATX2	498	423	1.18	0.23	0.18	0.43
YBR065C	ECM2	827	703	1.18	0.23	0.18	0.43
YDR443C	SSN2	1593	1355	1.18	0.23	0.18	0.43
YNL023C	FAP1	2043	1737	1.18	0.23	0.18	0.43
YNL124W		3352	2852	1.18	0.23	0.18	0.43
YDL151C		2354	2003	1.18	0.23	0.18	0.43
YMR090W		1430	1217	1.18	0.23	0.18	0.43
YMR305C	SCW10	2934	2497	1.17	0.23	0.18	0.43
YDR085C	AFR1	1906	1623	1.17	0.23	0.18	0.42
YPL240C	HSP82	3790	3228	1.17	0.23	0.18	0.42
YPR144C		1391	1185	1.17	0.23	0.18	0.42
YDR510W	SMT3	1623	1382	1.17	0.23	0.18	0.42
YJR099W	YUH1	1080	920	1.17	0.23	0.18	0.42
YLR146C	SPE4	4773	4066	1.17	0.23	0.18	0.42
YDR275W		1369	1167	1.17	0.23	0.17	0.42
YPR128C		2240	1909	1.17	0.23	0.17	0.42
YKL091C		682	581	1.17	0.23	0.17	0.42
YHR107C	CDC12	2119	1806	1.17	0.23	0.17	0.42
YGL162W	SUT1	415	354	1.17	0.23	0.17	0.42
YGR268C		3479	2967	1.17	0.23	0.17	0.42
YHR140W		1848	1576	1.17	0.23	0.17	0.42
YER128W		443	378	1.17	0.23	0.17	0.42
YCR107W	AAD3	1832	1563	1.17	0.23	0.17	0.42
YPL199C		768	655	1.17	0.23	0.17	0.42
YLR019W		2611	2228	1.17	0.23	0.17	0.42

YPR034W	ARP7	2753	2350	1.17	0.23	0.17	0.42
YJR014W		1122	958	1.17	0.23	0.17	0.42
YOR101W	RAS1	3245	2771	1.17	0.23	0.17	0.41
YOL152W	FRE7	2743	2342	1.17	0.23	0.17	0.41
YNL186W	UBP10	817	698	1.17	0.23	0.17	0.41
YHL010C		567	484	1.17	0.23	0.17	0.41
YER186C		705	602	1.17	0.23	0.17	0.41
YJL083W		1152	984	1.17	0.23	0.17	0.41
YGL109W		1465	1252	1.17	0.23	0.17	0.41
YNL146W		1145	979	1.17	0.23	0.17	0.41
YDR370C		659	563	1.17	0.23	0.17	0.41
YER125W	RSP5	2819	2410	1.17	0.23	0.17	0.41
YCL007C	CWH36	2425	2074	1.17	0.23	0.17	0.41
YJL206C-A		1331	1138	1.17	0.23	0.17	0.41
YPL262W	FUM1	1399	1196	1.17	0.23	0.17	0.41
YBR300C		817	699	1.17	0.23	0.17	0.41
YIL102C		1124	961	1.17	0.23	0.17	0.41
YJR093C	FIP1	598	512	1.17	0.22	0.17	0.41
YMR187C		730	625	1.17	0.22	0.17	0.41
YCLX01W		2439	2088	1.17	0.22	0.17	0.41
YDL228C		5486	4698	1.17	0.22	0.17	0.40
YDL203C		2068	1771	1.17	0.22	0.17	0.40
YJR089W	BIR1	2284	1956	1.17	0.22	0.17	0.40
YBR174C		812	696	1.17	0.22	0.17	0.40
YER146W	LSM5	713	611	1.17	0.22	0.17	0.40
YKL148C	SDH1	5066	4341	1.17	0.22	0.17	0.40
YKL165C	MCD4	3014	2583	1.17	0.22	0.17	0.40
YHR020W		2554	2189	1.17	0.22	0.17	0.40
YJL014W	CCT3	5547	4755	1.17	0.22	0.17	0.40
YMR269W		3091	2650	1.17	0.22	0.17	0.40
YBR172C	SMY2	3183	2729	1.17	0.22	0.17	0.40
YIL113W		984	844	1.17	0.22	0.17	0.40
YDR522C	SPS2	602	516	1.17	0.22	0.17	0.40
YJL006C	CTK2	1133	972	1.17	0.22	0.17	0.40
YAL007C	ERP2	2801	2402	1.17	0.22	0.17	0.40
YJL183W	MNN11	2470	2119	1.17	0.22	0.17	0.40

YMR200W	ROT1	529	454	1.17	0.22	0.16	0.40
YOL083W		1024	879	1.17	0.22	0.16	0.40
YNL192W	CHS1	1821	1563	1.17	0.22	0.16	0.40
YDR273W		2683	2303	1.17	0.22	0.16	0.40
YGR231C	PHB2	1842	1581	1.16	0.22	0.16	0.40
YNL121C	TOM70	1740	1495	1.16	0.22	0.16	0.39
YPR203W		2469	2122	1.16	0.22	0.16	0.39
YJR153W	PGU1	1142	982	1.16	0.22	0.16	0.39
YFL060C	SNO3	1962	1687	1.16	0.22	0.16	0.39
YGL200C	EMP24	1578	1357	1.16	0.22	0.16	0.39
YNL098C	RAS2	320	275	1.16	0.22	0.16	0.39
YKL116C		1535	1321	1.16	0.22	0.16	0.39
YNL120C		5296	4557	1.16	0.22	0.16	0.39
YMR207C	HFA1	1068	919	1.16	0.22	0.16	0.39
YFR025C	HIS2	1900	1635	1.16	0.22	0.16	0.39
YLR266C		1231	1060	1.16	0.22	0.16	0.39
YMR260C	TIF11	2623	2258	1.16	0.22	0.16	0.39
YLL029W		4210	3625	1.16	0.22	0.16	0.39
YBR281C		4841	4169	1.16	0.22	0.16	0.39
YDR175C		2856	2460	1.16	0.22	0.16	0.38
YPL124W	NIP29	536	462	1.16	0.22	0.16	0.38
YHR113W		2651	2283	1.16	0.22	0.16	0.38
YLR204W	QRI5	2674	2303	1.16	0.22	0.16	0.38
YHR005C	GPA1	1125	969	1.16	0.22	0.16	0.38
YKL202W		506	436	1.16	0.22	0.16	0.38
YMR006C	PLB2	1007	868	1.16	0.21	0.16	0.38
YPL116W	HOS3	860	741	1.16	0.21	0.16	0.38
YKR071C		3750	3233	1.16	0.21	0.16	0.38
YIL018W	RPL2B	5842	5038	1.16	0.21	0.16	0.38
YHR019C	DED81	2522	2175	1.16	0.21	0.16	0.38
YDR360W		3809	3285	1.16	0.21	0.16	0.38
YHR076W		1340	1156	1.16	0.21	0.16	0.38
YCR053W	THR4	2647	2283	1.16	0.21	0.16	0.38
YML100W	TSL1	2695	2325	1.16	0.21	0.16	0.38
YHR004C	NEM1	816	704	1.16	0.21	0.16	0.38
YBR133C	HSL7	3181	2744	1.16	0.21	0.16	0.38

YDR405W	MRP20	2303	1987	1.16	0.21	0.16	0.38
YMR251W-A	HOR7	460	397	1.16	0.21	0.16	0.38
YBR164C	ARL1	1839	1587	1.16	0.21	0.16	0.38
YGL038C	OCH1	3732	3220	1.16	0.21	0.16	0.38
YIR023W	DAL81	1941	1675	1.16	0.21	0.16	0.38
YJL154C	VPS35	3556	3069	1.16	0.21	0.16	0.38
YKL136W		377	325	1.16	0.21	0.16	0.38
YNL018C		1799	1553	1.16	0.21	0.16	0.38
YHR053C	CUP1-1	2249	1941	1.16	0.21	0.16	0.38
YFL033C	RIM15	2434	2101	1.16	0.21	0.16	0.38
YER081W	SER3	1843	1591	1.16	0.21	0.16	0.38
YJR158W	HXT16	694	599	1.16	0.21	0.16	0.38
YGL173C	KEM1	2480	2141	1.16	0.21	0.16	0.38
YOR247W	SRL1	952	822	1.16	0.21	0.16	0.37
YBR062C		1030	890	1.16	0.21	0.16	0.37
YLL028W	TPO1	1372	1185	1.16	0.21	0.16	0.37
YDR354W	TRP4	1160	1002	1.16	0.21	0.16	0.37
YML078W	CPR3	1958	1692	1.16	0.21	0.15	0.37
YJR090C	GRR1	1408	1217	1.16	0.21	0.15	0.37
YOR223W		834	721	1.16	0.21	0.15	0.37
YLR378C	SEC61	2602	2249	1.16	0.21	0.15	0.37
YIL041W		649	561	1.16	0.21	0.15	0.37
YDR282C		1681	1453	1.16	0.21	0.15	0.37
YOL153C		1600	1383	1.16	0.21	0.15	0.37
YKL086W		5922	5121	1.16	0.21	0.15	0.37
YBR004C		868	751	1.16	0.21	0.15	0.37
YDR258C	HSP78	1147	992	1.16	0.21	0.15	0.37
YDR073W	SNF11	1797	1555	1.16	0.21	0.15	0.37
YPL190C	NAB3	6243	5403	1.16	0.21	0.15	0.37
YCR070W		1399	1211	1.15	0.21	0.15	0.37
YOR211C	MGM1	1494	1294	1.15	0.21	0.15	0.37
YGR198W		2215	1918	1.15	0.21	0.15	0.37
YOR172W		745	645	1.15	0.21	0.15	0.37
YOR342C		1958	1696	1.15	0.21	0.15	0.36
YNL005C	MRP7	2648	2294	1.15	0.21	0.15	0.36
YKR037C	SPC34	591	512	1.15	0.21	0.15	0.36

YOR280C		1648	1428	1.15	0.21	0.15	0.36
YGR134W		1890	1638	1.15	0.21	0.15	0.36
YOL104C	NDJ1	832	721	1.15	0.21	0.15	0.36
YFR056C		2542	2203	1.15	0.21	0.15	0.36
YMR199W	CLN1	1437	1246	1.15	0.21	0.15	0.36
YOL088C	MPD2	1629	1412	1.15	0.21	0.15	0.36
YOR390W		1142	990	1.15	0.21	0.15	0.36
YBR105C	VID24	1311	1137	1.15	0.21	0.15	0.36
YGL185C		2738	2374	1.15	0.21	0.15	0.36
YOR332W	VMA4	775	672	1.15	0.21	0.15	0.36
YNR026C	SEC12	1886	1636	1.15	0.21	0.15	0.36
YOR031W	CRS5	314	272	1.15	0.21	0.15	0.36
YGR113W	DAM1	1130	980	1.15	0.21	0.15	0.36
YPL115C	BEM3	1815	1575	1.15	0.20	0.15	0.36
YLR056W	ERG3	4607	3998	1.15	0.20	0.15	0.36
YNL096C	RPS7B	310	269	1.15	0.20	0.15	0.36
YOR339C	UBC11	1234	1071	1.15	0.20	0.15	0.36
YEL036C	ANP1	5606	4867	1.15	0.20	0.15	0.36
YBR056W		1747	1517	1.15	0.20	0.15	0.36
YPR161C	SGV1	2215	1923	1.15	0.20	0.15	0.36
YGL194C	HOS2	470	408	1.15	0.20	0.15	0.36
YCR021C	HSP30	3605	3131	1.15	0.20	0.15	0.36
YBR106W	PHO88	2585	2246	1.15	0.20	0.15	0.35
YNL081C		372	323	1.15	0.20	0.15	0.35
YKL216W	URA1	673	585	1.15	0.20	0.15	0.35
YLR202C		1713	1489	1.15	0.20	0.15	0.35
YOR188W	MSB1	1827	1588	1.15	0.20	0.15	0.35
YMR079W	SEC14	1336	1161	1.15	0.20	0.15	0.35
YDR127W	ARO1	1438	1251	1.15	0.20	0.15	0.35
YJR057W	CDC8	865	752	1.15	0.20	0.15	0.35
YMR263W		2722	2368	1.15	0.20	0.14	0.35
YGR192C	TDH3	10580	9206	1.15	0.20	0.14	0.35
YLR324W		5358	4662	1.15	0.20	0.14	0.35
YKL069W		3947	3436	1.15	0.20	0.14	0.35
YJL197W	UBP12	1385	1206	1.15	0.20	0.14	0.35
YOR307C	SLY41	1314	1144	1.15	0.20	0.14	0.35

YMR028W	TAP42	1218	1061	1.15	0.20	0.14	0.35
YNL072W	RNH35	2072	1805	1.15	0.20	0.14	0.34
YNL043C		1432	1248	1.15	0.20	0.14	0.34
YGL108C		1124	979	1.15	0.20	0.14	0.34
YBR189W	RPS9B	2661	2319	1.15	0.20	0.14	0.34
YGR183C	QCR9	524	457	1.15	0.20	0.14	0.34
YLR232W		541	472	1.15	0.20	0.14	0.34
YHR197W		1579	1377	1.15	0.20	0.14	0.34
YIR021W	MRS1	3566	3110	1.15	0.20	0.14	0.34
YDR531W		1664	1451	1.15	0.20	0.14	0.34
YDR436W	PPZ2	2915	2542	1.15	0.20	0.14	0.34
YMR285C		3904	3405	1.15	0.20	0.14	0.34
YBR043C		2672	2331	1.15	0.20	0.14	0.34
YBR127C	VMA2	3209	2799	1.15	0.20	0.14	0.34
YJR106W	ECM27	1435	1252	1.15	0.20	0.14	0.34
YMR098C		748	653	1.15	0.20	0.14	0.34
YCR020C	PET18	1303	1137	1.15	0.20	0.14	0.34
YFL067W		3729	3255	1.15	0.20	0.14	0.34
YEL002C	WBP1	3273	2857	1.15	0.20	0.14	0.34
YDR508C	GNP1	3670	3204	1.15	0.20	0.14	0.34
YGR215W		844	737	1.15	0.20	0.14	0.34
YMR143W	RPS16A	4766	4162	1.15	0.20	0.14	0.34
YJR132W	NMD5	1307	1141	1.15	0.20	0.14	0.34
YKL036C		2085	1821	1.15	0.20	0.14	0.34
YJL153C	INO1	628	549	1.14	0.19	0.14	0.34
YFL022C	FRS2	5710	4989	1.14	0.19	0.14	0.33
YAL056W		774	676	1.14	0.19	0.14	0.33
YFL001W	DEG1	773	675	1.14	0.19	0.14	0.33
YOR276W	CAF20	458	400	1.14	0.19	0.14	0.33
YDR178W	SDH4	1298	1135	1.14	0.19	0.14	0.33
YBR206W		1235	1080	1.14	0.19	0.14	0.33
YPL105C		3231	2825	1.14	0.19	0.14	0.33
YLR028C	ADE16	4979	4354	1.14	0.19	0.14	0.33
YHL050C		7805	6826	1.14	0.19	0.14	0.33
YDL133C-A	YDL133C-A	654	572	1.14	0.19	0.14	0.33
YKL053C-A	YKL053C-A	1040	910	1.14	0.19	0.14	0.33

YJL121C	RPE1	733	641	1.14	0.19	0.14	0.33
YGR077C	PEX8	4101	3589	1.14	0.19	0.14	0.33
YLR188W	MDL1	2598	2275	1.14	0.19	0.14	0.33
YOL015W		1934	1693	1.14	0.19	0.14	0.33
YMR261C	TPS3	1712	1499	1.14	0.19	0.14	0.33
YEL017C-A	PMP2	1524	1335	1.14	0.19	0.14	0.33
YGR284C		1845	1616	1.14	0.19	0.14	0.33
YPL059W	GRX5	921	807	1.14	0.19	0.14	0.33
YDR372C		9740	8531	1.14	0.19	0.14	0.33
YPL134C		2904	2544	1.14	0.19	0.13	0.33
YLL042C	APG10	5362	4698	1.14	0.19	0.13	0.32
YOR260W	GCD1	1148	1006	1.14	0.19	0.13	0.32
YIL074C	SER33	3795	3326	1.14	0.19	0.13	0.32
YDR146C	SWI5	1245	1091	1.14	0.19	0.13	0.32
YKL033W		1280	1122	1.14	0.19	0.13	0.32
YML034W	SRC1	549	481	1.14	0.19	0.13	0.32
YFR010W	UBP6	2447	2146	1.14	0.19	0.13	0.32
YKR039W	GAP1	2073	1818	1.14	0.19	0.13	0.32
YDR388W	RVS167	2131	1869	1.14	0.19	0.13	0.32
YGL177W		351	308	1.14	0.19	0.13	0.32
YIL161W		3698	3244	1.14	0.19	0.13	0.32
YBR195C	MSI1	9900	8685	1.14	0.19	0.13	0.32
YER097W		524	460	1.14	0.19	0.13	0.32
YER189W		8472	7434	1.14	0.19	0.13	0.32
YGR082W	TOM20	2107	1849	1.14	0.19	0.13	0.32
YDL220C	CDC13	1120	983	1.14	0.19	0.13	0.32
YGR179C		1043	915	1.14	0.19	0.13	0.32
YER014W	HEM14	2180	1913	1.14	0.19	0.13	0.32
YJR009C	TDH2	9083	7972	1.14	0.19	0.13	0.32
YML058W	SML1	1058	929	1.14	0.19	0.13	0.32
YBR027C		923	810	1.14	0.19	0.13	0.32
YLL055W		1074	943	1.14	0.19	0.13	0.32
YLR194C		862	757	1.14	0.19	0.13	0.32
YNL012W	SPO1	2868	2519	1.14	0.19	0.13	0.32
YGL252C	RTG2	877	770	1.14	0.19	0.13	0.32
YHR214W		1524	1339	1.14	0.19	0.13	0.32

YOR185C	GSP2	1007	885	1.14	0.19	0.13	0.32
YPL088W		2889	2538	1.14	0.19	0.13	0.32
YKL149C	DBR1	2222	1952	1.14	0.19	0.13	0.32
YNL097C	PHO23	848	745	1.14	0.19	0.13	0.31
YNL128W	TEP1	545	479	1.14	0.19	0.13	0.31
YML124C	TUB3	1258	1106	1.14	0.19	0.13	0.31
YOR194C	TOA1	1971	1733	1.14	0.19	0.13	0.31
YBR082C	UBC4	4859	4272	1.14	0.19	0.13	0.31
YPR145W	ASN1	3488	3067	1.14	0.19	0.13	0.31
YKL177W		580	510	1.14	0.19	0.13	0.31
YNL148C	ALF1	1158	1018	1.14	0.19	0.13	0.31
YJLWdelta8	YJLWdelta8	503	442	1.14	0.19	0.13	0.31
YBR091C	MRS5	465	409	1.14	0.19	0.13	0.31
YBR142W	MAK5	7255	6385	1.14	0.18	0.13	0.31
YDR338C		1348	1186	1.14	0.18	0.13	0.31
YLR191W	PEX13	3165	2786	1.14	0.18	0.13	0.31
YDR120C	TRM1	2483	2186	1.14	0.18	0.13	0.31
YOR064C		607	534	1.14	0.18	0.13	0.31
YPL220W	RPL1A	3123	2749	1.14	0.18	0.13	0.31
YNL084C	END3	2101	1850	1.14	0.18	0.13	0.31
YDR129C	SAC6	1946	1714	1.14	0.18	0.13	0.31
YHR153C	SPO16	1291	1137	1.14	0.18	0.13	0.31
YBL088C	TEL1	5204	4585	1.14	0.18	0.13	0.31
YGL230C		3681	3243	1.14	0.18	0.13	0.31
YGL191W	COX13	1555	1370	1.13	0.18	0.13	0.31
YER061C	CEM1	1916	1688	1.13	0.18	0.13	0.31
YPL280W		1877	1654	1.13	0.18	0.13	0.30
YNL041C		1041	918	1.13	0.18	0.13	0.30
YOR272W	YTM1	1687	1487	1.13	0.18	0.13	0.30
YGL233W	SEC15	4044	3566	1.13	0.18	0.13	0.30
YDL051W	YLA1	1592	1404	1.13	0.18	0.13	0.30
YKR046C		5001	4410	1.13	0.18	0.13	0.30
YIL076W	SEC28	963	849	1.13	0.18	0.13	0.30
YLR330W	CHS5	2309	2037	1.13	0.18	0.12	0.30
YGR036C	CAX4	1828	1613	1.13	0.18	0.12	0.30
YBRCdelta19	YBRCdelta19	816	720	1.13	0.18	0.12	0.30

YCR106W		2305	2034	1.13	0.18	0.12	0.30
YKR024C	DBP7	1905	1681	1.13	0.18	0.12	0.30
YDL192W	ARF1	1585	1399	1.13	0.18	0.12	0.30
YLR159W		5702	5033	1.13	0.18	0.12	0.30
YDL115C		1282	1132	1.13	0.18	0.12	0.30
YGL207W	SPT16	5020	4432	1.13	0.18	0.12	0.30
YJL052W	TDH1	4699	4149	1.13	0.18	0.12	0.30
YIL039W		3462	3057	1.13	0.18	0.12	0.30
YMR284W	YKU70	2430	2146	1.13	0.18	0.12	0.30
YJL095W	BCK1	3199	2825	1.13	0.18	0.12	0.30
YFR034C	PHO4	1573	1389	1.13	0.18	0.12	0.30
YMR142C	RPL13B	6036	5331	1.13	0.18	0.12	0.30
YGL084C		1758	1553	1.13	0.18	0.12	0.30
YGR065C	VHT1	1475	1303	1.13	0.18	0.12	0.30
YNL194C		538	475	1.13	0.18	0.12	0.30
YMR081C	ISF1	867	766	1.13	0.18	0.12	0.30
YGL234W	ADE5,7	2977	2631	1.13	0.18	0.12	0.30
YIL024C		4062	3590	1.13	0.18	0.12	0.30
YDR157W		380	336	1.13	0.18	0.12	0.30
YGR089W		2968	2623	1.13	0.18	0.12	0.29
YER052C	HOM3	1138	1006	1.13	0.18	0.12	0.29
YML046W	PRP39	1206	1066	1.13	0.18	0.12	0.29
YLL035W		3083	2726	1.13	0.18	0.12	0.29
YML007W	YAP1	8592	7598	1.13	0.18	0.12	0.29
YJL167W	ERG20	3008	2660	1.13	0.18	0.12	0.29
YCR029C-A		512	453	1.13	0.18	0.12	0.29
YPR043W	RPL43A	1331	1177	1.13	0.18	0.12	0.29
YBR039W	ATP3	988	874	1.13	0.18	0.12	0.29
YER176W	ECM32	3217	2846	1.13	0.18	0.12	0.29
YML126C	HMGS	4546	4023	1.13	0.18	0.12	0.29
YER056C	FCY2	860	761	1.13	0.18	0.12	0.29
YPR127W		1665	1474	1.13	0.18	0.12	0.29
YKL141W	SDH3	975	863	1.13	0.18	0.12	0.29
YMR039C	SUB1	3158	2796	1.13	0.18	0.12	0.29
YHR203C	RPS4B	788	698	1.13	0.18	0.12	0.29
YAR073W		8981	7954	1.13	0.18	0.12	0.29

YHR049C-A		4795	4248	1.13	0.17	0.12	0.29
YCL048W		3762	3333	1.13	0.17	0.12	0.29
YPR048W	TAH18	4493	3982	1.13	0.17	0.12	0.29
YDR134C		2371	2101	1.13	0.17	0.12	0.29
YMR089C	YTA12	2743	2431	1.13	0.17	0.12	0.28
YDRCdelta9	YDRCdelta9	791	701	1.13	0.17	0.12	0.28
YGL181W	GTS1	6771	6004	1.13	0.17	0.12	0.28
YPR028W	YIP2	835	741	1.13	0.17	0.12	0.28
YER063W	THO1	900	798	1.13	0.17	0.12	0.28
YML125C		1880	1668	1.13	0.17	0.12	0.28
YKR006C	MRPL13	832	738	1.13	0.17	0.12	0.28
YNL066W	SUN4	1740	1544	1.13	0.17	0.12	0.28
YMR131C		2112	1874	1.13	0.17	0.12	0.28
YBR010W	HHT1	1477	1311	1.13	0.17	0.12	0.28
YGR181W	TIM13	696	618	1.13	0.17	0.12	0.28
YDL123W		1326	1177	1.13	0.17	0.12	0.28
YDL241W		517	459	1.13	0.17	0.12	0.28
YPR024W	YME1	1529	1358	1.13	0.17	0.12	0.28
YBL022C	PIM1	4279	3800	1.13	0.17	0.12	0.28
YOL117W		788	700	1.13	0.17	0.12	0.28
YDR242W	AMD2	900	799	1.13	0.17	0.12	0.28
YBL027W	RPL19B	2959	2629	1.13	0.17	0.11	0.28
YKL174C		1738	1544	1.13	0.17	0.11	0.28
YNL026W		3780	3360	1.13	0.17	0.11	0.28
YDR528W		1888	1678	1.12	0.17	0.11	0.27
YER115C	SPR6	682	606	1.12	0.17	0.11	0.27
YDL076C		1409	1253	1.12	0.17	0.11	0.27
YDR034C	LYS14	1406	1250	1.12	0.17	0.11	0.27
YPR124W	CTR1	4254	3783	1.12	0.17	0.11	0.27
YJR147W	HMS2	1968	1750	1.12	0.17	0.11	0.27
YHR079C-B		2083	1853	1.12	0.17	0.11	0.27
YLR258W	GSY2	1906	1696	1.12	0.17	0.11	0.27
YLR246W	ERF2	1076	958	1.12	0.17	0.11	0.27
YNL008C		1011	900	1.12	0.17	0.11	0.27
YDL032W		4072	3625	1.12	0.17	0.11	0.27
YER041W		746	664	1.12	0.17	0.11	0.27

YLR436C	ECM30	3620	3223	1.12	0.17	0.11	0.27
YOL067C	RTG1	1412	1257	1.12	0.17	0.11	0.27
YNR019W	ARE2	1874	1669	1.12	0.17	0.11	0.27
YBR173C	UMP1	4400	3919	1.12	0.17	0.11	0.27
YNL302C	RPS19B	1729	1540	1.12	0.17	0.11	0.27
YOR374W	ALD4	1432	1276	1.12	0.17	0.11	0.27
YPL206C		2203	1962	1.12	0.17	0.11	0.27
YML043C	RRN11	924	823	1.12	0.17	0.11	0.27
YLR049C		1937	1726	1.12	0.17	0.11	0.27
YDR137W	RGP1	2592	2310	1.12	0.17	0.11	0.27
YDL031W	DBP10	2064	1840	1.12	0.17	0.11	0.27
YDL004W	ATP16	1508	1344	1.12	0.17	0.11	0.26
YDR049W		1120	999	1.12	0.16	0.11	0.26
YHR099W	TRA1	4799	4281	1.12	0.16	0.11	0.26
YER013W	PRP22	2322	2072	1.12	0.16	0.11	0.26
YOR329C	SCD5	1264	1128	1.12	0.16	0.11	0.26
YGR125W		2312	2063	1.12	0.16	0.11	0.26
YLR037C		1228	1096	1.12	0.16	0.11	0.26
YJL010C		4590	4098	1.12	0.16	0.11	0.26
YLR003C		880	786	1.12	0.16	0.11	0.26
YOL080C		402	359	1.12	0.16	0.11	0.26
YER174C	GRX4	639	571	1.12	0.16	0.11	0.26
YPR018W	RLF2	2497	2230	1.12	0.16	0.11	0.26
YDL009C		7408	6618	1.12	0.16	0.11	0.26
YCL016C		3451	3085	1.12	0.16	0.11	0.25
YLR384C	IKI3	1302	1164	1.12	0.16	0.11	0.25
YGR027C	RPS25A	845	756	1.12	0.16	0.10	0.25
YBL113C		3430	3069	1.12	0.16	0.10	0.25
YDL242W		653	584	1.12	0.16	0.10	0.25
YGL059W		1535	1374	1.12	0.16	0.10	0.25
YGR046W		2079	1861	1.12	0.16	0.10	0.25
YDL021W	GPM2	1874	1678	1.12	0.16	0.10	0.25
YPR173C	VPS4	2288	2049	1.12	0.16	0.10	0.25
YDL090C	RAM1	1466	1313	1.12	0.16	0.10	0.25
YNL054W	VAC7	2687	2406	1.12	0.16	0.10	0.25
YBR001C	NTH2	1049	939	1.12	0.16	0.10	0.25

YEL055C	POL5	3088	2765	1.12	0.16	0.10	0.25
YHR044C	DOG1	1809	1620	1.12	0.16	0.10	0.25
YAL023C	PMT2	4383	3927	1.12	0.16	0.10	0.25
YDR099W	BMH2	3216	2882	1.12	0.16	0.10	0.25
YAL061W		1849	1657	1.12	0.16	0.10	0.25
YIL173W	VTH1	1307	1171	1.12	0.16	0.10	0.25
YLR195C	NMT1	2215	1985	1.12	0.16	0.10	0.25
YNR009W		965	865	1.12	0.16	0.10	0.25
YNL091W		467	419	1.12	0.16	0.10	0.25
YMR086W		2165	1941	1.12	0.16	0.10	0.24
YLR120C	YPS1	3308	2966	1.12	0.16	0.10	0.24
YDR303C		465	417	1.12	0.16	0.10	0.24
YBR025C		5561	4989	1.11	0.16	0.10	0.24
YBR200W	BEM1	2413	2165	1.11	0.16	0.10	0.24
YPR070W	MED1	645	579	1.11	0.16	0.10	0.24
YMR326C		392	352	1.11	0.16	0.10	0.24
YOR322C		1859	1668	1.11	0.16	0.10	0.24
YPR041W	TIF5	1579	1417	1.11	0.16	0.10	0.24
YGR075C	PRP38	725	651	1.11	0.16	0.10	0.24
YER073W	ALD5	920	826	1.11	0.16	0.10	0.24
YGR078C	PAC10	1309	1175	1.11	0.16	0.10	0.24
YIL070C	MAM33	2768	2485	1.11	0.16	0.10	0.24
YGR224W		1556	1397	1.11	0.16	0.10	0.24
YNL147W	LSM7	1068	959	1.11	0.16	0.10	0.24
YDR480W	DIG2	1137	1021	1.11	0.16	0.10	0.24
YPL030W		4803	4314	1.11	0.16	0.10	0.24
YFL038C	YPT1	3751	3369	1.11	0.16	0.10	0.24
YOR262W		3720	3341	1.11	0.15	0.10	0.24
YBR125C	PTC4	898	807	1.11	0.15	0.10	0.24
YPR100W		2744	2465	1.11	0.15	0.10	0.24
YAR071W	PHO11	7528	6763	1.11	0.15	0.10	0.24
YLR085C	ARP6	2245	2017	1.11	0.15	0.10	0.24
YML014W		1952	1754	1.11	0.15	0.10	0.24
YJL026W	RNR2	4412	3966	1.11	0.15	0.10	0.24
YJL177W	RPL17B	1121	1008	1.11	0.15	0.10	0.24
YDL132W	CDC53	3662	3292	1.11	0.15	0.10	0.24

YPL188W	POS5	2167	1949	1.11	0.15	0.10	0.23
YCR045C		1349	1213	1.11	0.15	0.10	0.23
YJL137C	GLG2	666	599	1.11	0.15	0.10	0.23
YAL032C	PRP45	6338	5702	1.11	0.15	0.10	0.23
YER141W	COX15	1379	1241	1.11	0.15	0.10	0.23
YDL128W	VCX1	2373	2136	1.11	0.15	0.10	0.23
YMR075C-A		835	752	1.11	0.15	0.10	0.23
YHR006W	STP2	1832	1649	1.11	0.15	0.10	0.23
YIL134W	FLX1	1091	982	1.11	0.15	0.10	0.23
YAL044C	GCV3	1346	1212	1.11	0.15	0.10	0.23
YDR434W		1206	1086	1.11	0.15	0.10	0.23
YIL009W	FAA3	2275	2049	1.11	0.15	0.09	0.23
YDL173W		2649	2386	1.11	0.15	0.09	0.23
YLR368W		1417	1276	1.11	0.15	0.09	0.23
YPL042C	SSN3	2109	1900	1.11	0.15	0.09	0.23
YIL035C	CKA1	2213	1994	1.11	0.15	0.09	0.23
YNL157W		785	707	1.11	0.15	0.09	0.23
YPLCTy4-1D	YPLCTy4-1D	504	454	1.11	0.15	0.09	0.23
YPL247C		1363	1229	1.11	0.15	0.09	0.23
YPL209C	IPL1	317	286	1.11	0.15	0.09	0.23
YKR066C	CCP1	2259	2037	1.11	0.15	0.09	0.23
YCL060C		3603	3249	1.11	0.15	0.09	0.23
YER007W	PAC2	1900	1713	1.11	0.15	0.09	0.23
YBR077C		461	416	1.11	0.15	0.09	0.22
YMR066W	SOV1	1406	1268	1.11	0.15	0.09	0.22
YMR182C	RGM1	4012	3619	1.11	0.15	0.09	0.22
YDR465C	RMT2	2590	2337	1.11	0.15	0.09	0.22
YPL028W	ERG10	2339	2111	1.11	0.15	0.09	0.22
YFL039C	ACT1	6878	6207	1.11	0.15	0.09	0.22
YLR319C	BUD6	5554	5013	1.11	0.15	0.09	0.22
YER037W		613	553	1.11	0.15	0.09	0.22
YGR266W		2463	2223	1.11	0.15	0.09	0.22
YMR008C	PLB1	6385	5763	1.11	0.15	0.09	0.22
YEL051W	VMA8	1993	1799	1.11	0.15	0.09	0.22
YKR030W		4624	4176	1.11	0.15	0.09	0.22
YGR092W	DBF2	1901	1717	1.11	0.15	0.09	0.22

YJL155C	FBP26	1214	1097	1.11	0.15	0.09	0.22
YHR103W	SBE22	2619	2366	1.11	0.15	0.09	0.22
YLR305C	STT4	2619	2366	1.11	0.15	0.09	0.22
YGL058W	RAD6	2464	2226	1.11	0.15	0.09	0.22
YKR061W	KTR2	1748	1580	1.11	0.15	0.09	0.22
YOR388C	FDH1	527	476	1.11	0.15	0.09	0.22
YPR056W	TFB4	1567	1416	1.11	0.15	0.09	0.22
YNL300W		2078	1878	1.11	0.15	0.09	0.22
YKR079C		4491	4060	1.11	0.15	0.09	0.22
YCR011C	ADP1	4711	4260	1.11	0.15	0.09	0.22
YOR296W		1698	1535	1.11	0.15	0.09	0.22
YER064C		740	669	1.11	0.15	0.09	0.22
YDL202W	MRPL11	484	438	1.11	0.15	0.09	0.21
YPL267W		789	714	1.11	0.14	0.09	0.21
YER025W	GCD11	1930	1746	1.11	0.14	0.09	0.21
YLR151C		3306	2991	1.11	0.14	0.09	0.21
YMR306W	FKS3	1520	1375	1.11	0.14	0.09	0.21
YHR063C		620	561	1.11	0.14	0.09	0.21
YNR069C		846	766	1.10	0.14	0.09	0.21
YKL125W	RRN3	2406	2178	1.10	0.14	0.09	0.21
YDR494W		1561	1413	1.10	0.14	0.09	0.21
YDR102C		2400	2173	1.10	0.14	0.09	0.21
YMR283C	RIT1	852	772	1.10	0.14	0.09	0.21
YDR263C	DIN7	977	885	1.10	0.14	0.09	0.21
YGR187C	HGH1	2233	2023	1.10	0.14	0.09	0.21
YBR017C	KAP104	994	901	1.10	0.14	0.09	0.21
YGR094W	VAS1	5104	4627	1.10	0.14	0.09	0.21
YBR282W	MRPL27	3927	3561	1.10	0.14	0.08	0.21
YGL116W	CDC20	1586	1439	1.10	0.14	0.08	0.20
YGR034W	RPL26B	2161	1961	1.10	0.14	0.08	0.20
YMR149W	SWP1	783	711	1.10	0.14	0.08	0.20
YHL027W	RIM101	2312	2098	1.10	0.14	0.08	0.20
YKL048C	ELM1	2241	2035	1.10	0.14	0.08	0.20
YDL033C		2701	2453	1.10	0.14	0.08	0.20
YHR047C	AAP1'	559	508	1.10	0.14	0.08	0.20
YKL074C	MUD2	4328	3930	1.10	0.14	0.08	0.20

YOL007C	CSI2	3484	3164	1.10	0.14	0.08	0.20
YKL004W	AUR1	3083	2800	1.10	0.14	0.08	0.20
YHR056C		2349	2134	1.10	0.14	0.08	0.20
YOR359W		3050	2771	1.10	0.14	0.08	0.20
YGR193C	PDX1	977	888	1.10	0.14	0.08	0.20
YBR286W	APE3	5396	4904	1.10	0.14	0.08	0.20
YMR231W	PEP5	4583	4166	1.10	0.14	0.08	0.20
YPL264C		1551	1410	1.10	0.14	0.08	0.20
YOR022C		1464	1331	1.10	0.14	0.08	0.20
YBR145W	ADH5	862	784	1.10	0.14	0.08	0.20
YDL079C	MRK1	1245	1132	1.10	0.14	0.08	0.20
YHL004W	MRP4	2018	1836	1.10	0.14	0.08	0.19
YGL055W	OLE1	6918	6293	1.10	0.14	0.08	0.19
YML047C		1466	1334	1.10	0.14	0.08	0.19
YKL112W	ABF1	1871	1702	1.10	0.14	0.08	0.19
YMR294W-A		2383	2169	1.10	0.14	0.08	0.19
YNL246W		391	356	1.10	0.14	0.08	0.19
YKL078W		861	784	1.10	0.14	0.08	0.19
YOL028C	YAP7	2652	2415	1.10	0.13	0.08	0.19
YBL072C	RPS8A	4958	4517	1.10	0.13	0.08	0.19
YMR054W	STV1	2603	2372	1.10	0.13	0.08	0.19
YGLWdelta4	YGLWdelta4	3588	3269	1.10	0.13	0.08	0.19
YER190W	YRF1-2	4208	3834	1.10	0.13	0.08	0.19
YIL147C	SLN1	2871	2617	1.10	0.13	0.08	0.19
YOR110W		1970	1796	1.10	0.13	0.08	0.19
YKL132C	RMA1	769	701	1.10	0.13	0.08	0.19
YOR368W	RAD17	3935	3588	1.10	0.13	0.08	0.19
YPL265W	DIP5	3395	3096	1.10	0.13	0.08	0.19
YER082C		2648	2415	1.10	0.13	0.08	0.19
YCR088W	ABP1	1716	1565	1.10	0.13	0.08	0.18
YML102W	CAC2	1738	1585	1.10	0.13	0.08	0.18
YDR116C		2246	2049	1.10	0.13	0.08	0.18
YJL059W	YHC3	2023	1846	1.10	0.13	0.08	0.18
YPL109C		682	622	1.10	0.13	0.08	0.18
YOR008C	SLG1	2623	2393	1.10	0.13	0.08	0.18
YGR276C	RNH70	2205	2012	1.10	0.13	0.08	0.18

YOR149C	SMP3	2906	2652	1.10	0.13	0.08	0.18
YKL137W		2351	2146	1.10	0.13	0.08	0.18
YBR064W		3327	3037	1.10	0.13	0.08	0.18
YGR190C		2750	2510	1.10	0.13	0.08	0.18
YNL308C	KRI1	2760	2519	1.10	0.13	0.08	0.18
YCR069W	SCC3	1700	1552	1.10	0.13	0.08	0.18
YBL102W	SFT2	689	629	1.10	0.13	0.08	0.18
YCL057W	PRD1	3933	3591	1.10	0.13	0.08	0.18
YDR038C	ENA5	321	293	1.10	0.13	0.07	0.18
YMR278W		1248	1140	1.10	0.13	0.07	0.18
YGL203C	KEX1	6390	5835	1.10	0.13	0.07	0.18
YFRWdelta10	YFRWdelta10	458	418	1.09	0.13	0.07	0.18
YBR126C	TPS1	4236	3871	1.09	0.13	0.07	0.18
YOL046C		8421	7698	1.09	0.13	0.07	0.18
YJR145C	RPS4A	3863	3531	1.09	0.13	0.07	0.18
YLR218C		1348	1232	1.09	0.13	0.07	0.18
YOR117W	RPT5	2094	1914	1.09	0.13	0.07	0.18
YNL092W		4236	3874	1.09	0.13	0.07	0.18
YDR154C		5077	4645	1.09	0.13	0.07	0.17
YLL056C		854	781	1.09	0.13	0.07	0.17
YGL081W		953	872	1.09	0.13	0.07	0.17
YPL232W	SSO1	4444	4067	1.09	0.13	0.07	0.17
YNL218W		1361	1246	1.09	0.13	0.07	0.17
YGR041W	BUD9	4456	4080	1.09	0.13	0.07	0.17
YDR488C	PAC11	1980	1813	1.09	0.13	0.07	0.17
YDR497C	ITR1	1624	1487	1.09	0.13	0.07	0.17
YDR376W	ARH1	3531	3234	1.09	0.13	0.07	0.17
YNL195C		1354	1240	1.09	0.13	0.07	0.17
YCL041C		3757	3442	1.09	0.13	0.07	0.17
YIR004W	DJP1	2315	2121	1.09	0.13	0.07	0.17
YJR157W		1616	1481	1.09	0.13	0.07	0.17
YKL051W		2537	2325	1.09	0.13	0.07	0.17
YNL283C	WSC2	2568	2354	1.09	0.13	0.07	0.17
YNL220W	ADE12	1244	1140	1.09	0.13	0.07	0.17
YIL138C	TPM2	1298	1190	1.09	0.13	0.07	0.17
YER020W	GPA2	934	856	1.09	0.13	0.07	0.17

YJL081C	ARP4	2009	1842	1.09	0.13	0.07	0.17
YCR058C		1855	1701	1.09	0.13	0.07	0.17
YGR020C	VMA7	10015	9184	1.09	0.12	0.07	0.17
YLR162W		1074	985	1.09	0.12	0.07	0.17
YER111C	SWI4	2267	2079	1.09	0.12	0.07	0.17
YDR080W	VPS41	515	472	1.09	0.12	0.07	0.17
YMR127C	SAS2	6290	5770	1.09	0.12	0.07	0.17
YHR114W		6256	5739	1.09	0.12	0.07	0.17
YDR007W	TRP1	763	700	1.09	0.12	0.07	0.16
YNL299W	TRF5	2305	2116	1.09	0.12	0.07	0.16
YOR246C		2153	1976	1.09	0.12	0.07	0.16
YKL079W	SMY1	1500	1377	1.09	0.12	0.07	0.16
YDR377W	ATP17	2934	2694	1.09	0.12	0.07	0.16
YHL040C	ARN1	1524	1400	1.09	0.12	0.07	0.16
YBR252W	DUT1	410	377	1.09	0.12	0.07	0.16
YNL055C	POR1	2126	1953	1.09	0.12	0.07	0.16
YBR257W	POP4	3335	3063	1.09	0.12	0.07	0.16
YLL043W	FPS1	1953	1794	1.09	0.12	0.07	0.16
YDR193W		1552	1426	1.09	0.12	0.07	0.16
YIL145C		2328	2139	1.09	0.12	0.07	0.16
YIL164C	NIT1	1695	1558	1.09	0.12	0.07	0.16
YLL023C		1713	1575	1.09	0.12	0.07	0.16
YBR112C	CYC8	4028	3704	1.09	0.12	0.07	0.16
YKL209C	STE6	1718	1580	1.09	0.12	0.06	0.16
YIR005W	IST3	4119	3789	1.09	0.12	0.06	0.16
YCL027W	FUS1	3128	2877	1.09	0.12	0.06	0.16
YLR467W	YRF1-5	6493	5974	1.09	0.12	0.06	0.15
YDR104C		3478	3200	1.09	0.12	0.06	0.15
YCR084C	TUP1	6049	5567	1.09	0.12	0.06	0.15
YBR256C	RIB5	1842	1695	1.09	0.12	0.06	0.15
YML031W	NDC1	2682	2469	1.09	0.12	0.06	0.15
YJL044C	GYP6	959	883	1.09	0.12	0.06	0.15
YER059W	PCL6	917	844	1.09	0.12	0.06	0.15
YNL093W	YPT53	1189	1095	1.09	0.12	0.06	0.15
YDR256C	CTA1	1219	1123	1.09	0.12	0.06	0.15
YMR171C		2275	2096	1.09	0.12	0.06	0.15

YDL154W	MSH5	576	531	1.09	0.12	0.06	0.15
YLL018C	DPS1	2418	2228	1.09	0.12	0.06	0.15
YIL012W		5248	4836	1.09	0.12	0.06	0.15
YDR458C		3846	3544	1.09	0.12	0.06	0.15
YER088C	DOT6	763	703	1.09	0.12	0.06	0.15
YNR021W		2391	2204	1.09	0.12	0.06	0.15
YOL057W		2888	2662	1.08	0.12	0.06	0.15
YBL105C	PKC1	507	468	1.08	0.12	0.06	0.15
YEL004W	YEA4	1373	1267	1.08	0.12	0.06	0.14
YBL061C	SKT5	7043	6501	1.08	0.12	0.06	0.14
YDRWdelta7	YDRWdelta7	741	684	1.08	0.12	0.06	0.14
YLR035C		1400	1292	1.08	0.12	0.06	0.14
YJR051W	OSM1	1348	1244	1.08	0.12	0.06	0.14
YDR072C	IPT1	2270	2096	1.08	0.12	0.06	0.14
YPR103W	PRE2	3674	3392	1.08	0.12	0.06	0.14
YIL038C	NOT3	3683	3401	1.08	0.12	0.06	0.14
YFL059W	SNZ3	919	849	1.08	0.11	0.06	0.14
YGR095C	RRP46	704	650	1.08	0.11	0.06	0.14
YPR039W		438	405	1.08	0.11	0.06	0.14
YOR230W	WTM1	1202	1111	1.08	0.11	0.06	0.14
YPR021C		1171	1082	1.08	0.11	0.06	0.14
YEL050C	RML2	2838	2623	1.08	0.11	0.06	0.14
YJR019C	TES1	1545	1428	1.08	0.11	0.06	0.14
YLR226W	BUR2	569	526	1.08	0.11	0.06	0.14
YGL123W	RPS2	4515	4175	1.08	0.11	0.06	0.14
YDR460W	TFB3	1893	1750	1.08	0.11	0.06	0.14
YFR022W		1790	1655	1.08	0.11	0.06	0.14
YPL182C		2314	2140	1.08	0.11	0.06	0.14
YEL048C		414	383	1.08	0.11	0.06	0.14
YLL050C	COF1	2483	2297	1.08	0.11	0.06	0.14
YKR099W	BAS1	3704	3426	1.08	0.11	0.06	0.14
YNL291C	MID1	4992	4618	1.08	0.11	0.06	0.14
YNL268W	LYP1	2184	2021	1.08	0.11	0.06	0.14
YLL027W	ISA1	441	408	1.08	0.11	0.06	0.13
YGR128C		5056	4681	1.08	0.11	0.06	0.13
YGL034C		840	778	1.08	0.11	0.06	0.13

YBL101C	ECM21	920	852	1.08	0.11	0.06	0.13
YDL155W	CLB3	2908	2693	1.08	0.11	0.05	0.13
YPR162C	ORC4	1338	1239	1.08	0.11	0.05	0.13
YPL266W	DIM1	1217	1127	1.08	0.11	0.05	0.13
YDR098C	GRX3	598	554	1.08	0.11	0.05	0.13
YBR267W		2849	2639	1.08	0.11	0.05	0.13
YLR182W	SWI6	4561	4225	1.08	0.11	0.05	0.13
YGL245W		4705	4360	1.08	0.11	0.05	0.13
YBL090W	MRP21	565	524	1.08	0.11	0.05	0.13
YMR017W	SPO20	417	386	1.08	0.11	0.05	0.13
YGL135W	RPL1B	3907	3621	1.08	0.11	0.05	0.13
YBR092C	PHO3	1802	1670	1.08	0.11	0.05	0.13
YNL020C	ARK1	1512	1403	1.08	0.11	0.05	0.13
YDR539W		3415	3168	1.08	0.11	0.05	0.13
YKR063C	LAS1	2466	2289	1.08	0.11	0.05	0.12
YNL028W		4774	4431	1.08	0.11	0.05	0.12
YCL049C		2308	2142	1.08	0.11	0.05	0.12
YEL034W	HYP2	4916	4563	1.08	0.11	0.05	0.12
YNL286W	CUS2	1378	1279	1.08	0.11	0.05	0.12
YPL041C		850	789	1.08	0.11	0.05	0.12
YOR264W		1103	1024	1.08	0.11	0.05	0.12
YBR055C	PRP6	389	361	1.08	0.11	0.05	0.12
YBL060W		1963	1823	1.08	0.11	0.05	0.12
YDR280W	RRP45	1715	1593	1.08	0.11	0.05	0.12
YCRX14W		7850	7294	1.08	0.11	0.05	0.12
YGL251C	HFM1	2536	2356	1.08	0.11	0.05	0.12
YBL043W	ECM13	2513	2335	1.08	0.11	0.05	0.12
YCR071C	IMG2	1855	1724	1.08	0.11	0.05	0.12
YDR368W	YPR1	501	466	1.08	0.11	0.05	0.12
YNL064C	YDJ1	597	555	1.08	0.11	0.05	0.12
YDR391C		1046	973	1.08	0.10	0.05	0.12
YAR030C		479	446	1.08	0.10	0.05	0.12
YGL051W		1026	954	1.08	0.10	0.05	0.12
YFR015C	GSY1	2089	1943	1.07	0.10	0.05	0.12
YBR242W		3544	3297	1.07	0.10	0.05	0.12
YDR513W	TTR1	517	481	1.07	0.10	0.05	0.12

YLR101C		7252	6748	1.07	0.10	0.05	0.12
YLR290C		516	480	1.07	0.10	0.05	0.12
YDL174C	DLD1	7854	7309	1.07	0.10	0.05	0.11
YBL084C	CDC27	3846	3579	1.07	0.10	0.05	0.11
YMR061W	RNA14	4694	4369	1.07	0.10	0.05	0.11
YML042W	CAT2	2454	2285	1.07	0.10	0.05	0.11
YPL039W		618	575	1.07	0.10	0.05	0.11
YDR328C	SKP1	835	778	1.07	0.10	0.05	0.11
YDR506C		1137	1059	1.07	0.10	0.05	0.11
YDR490C	PKH1	1430	1332	1.07	0.10	0.05	0.11
YPR032W	SRO7	1561	1454	1.07	0.10	0.05	0.11
YMR135C		1379	1285	1.07	0.10	0.05	0.11
YDL143W	CCT4	3011	2806	1.07	0.10	0.05	0.11
YDL005C	MED2	2706	2522	1.07	0.10	0.05	0.11
YKL035W	UGP1	5157	4806	1.07	0.10	0.05	0.11
YKL043W	PHD1	2572	2397	1.07	0.10	0.05	0.11
YER101C	AST2	424	395	1.07	0.10	0.05	0.11
YJL157C	FAR1	414	386	1.07	0.10	0.05	0.11
YDL038C		1531	1427	1.07	0.10	0.04	0.11
YBR229C	ROT2	4507	4202	1.07	0.10	0.04	0.11
YLR221C		1258	1173	1.07	0.10	0.04	0.11
YDR471W	RPL27B	3858	3598	1.07	0.10	0.04	0.11
YDR392W	SPT3	1131	1055	1.07	0.10	0.04	0.11
YJL117W	PHO86	1327	1238	1.07	0.10	0.04	0.11
YLR140W		2109	1968	1.07	0.10	0.04	0.11
YOR324C		989	923	1.07	0.10	0.04	0.11
YNL104C	LEU4	8402	7840	1.07	0.10	0.04	0.11
YCR015C		1044	974	1.07	0.10	0.04	0.11
YOR025W	HST3	1297	1211	1.07	0.10	0.04	0.10
YOL106W		3279	3062	1.07	0.10	0.04	0.10
YLR005W	SSL1	1028	960	1.07	0.10	0.04	0.10
YJL030W	MAD2	7028	6563	1.07	0.10	0.04	0.10
YMR168C	CEP3	2469	2306	1.07	0.10	0.04	0.10
YIR043C		785	733	1.07	0.10	0.04	0.10
YML133C		7586	7087	1.07	0.10	0.04	0.10
YGL048C	RPT6	1780	1663	1.07	0.10	0.04	0.10

YGL100W	SEH1	1625	1519	1.07	0.10	0.04	0.10
YDL006W	PTC1	1367	1278	1.07	0.10	0.04	0.10
YOR127W	RGA1	477	446	1.07	0.10	0.04	0.10
YKL134C	36433	1247	1166	1.07	0.10	0.04	0.10
YPL070W		1366	1277	1.07	0.10	0.04	0.10
YBR138C	HDR1	1916	1791	1.07	0.10	0.04	0.10
YGR021W		1365	1276	1.07	0.10	0.04	0.10
YOR328W	PDR10	4066	3802	1.07	0.10	0.04	0.10
YIL045W	PIG2	1877	1755	1.07	0.10	0.04	0.10
YCL019W		9633	9011	1.07	0.10	0.04	0.10
YJL162C		1222	1143	1.07	0.10	0.04	0.10
YOR039W	CKB2	1880	1760	1.07	0.10	0.04	0.10
YBR066C	NRG2	2425	2270	1.07	0.10	0.04	0.10
YGR208W	SER2	1744	1632	1.07	0.10	0.04	0.09
YLR303W	MET17	8995	8420	1.07	0.10	0.04	0.09
YJL085W	EXO70	980	917	1.07	0.10	0.04	0.09
YGL120C	PRP43	3959	3707	1.07	0.10	0.04	0.09
YLR446W		1757	1645	1.07	0.09	0.04	0.09
YPL278C		435	407	1.07	0.09	0.04	0.09
YDR159W	SAC3	4782	4477	1.07	0.09	0.04	0.09
YNL263C	YIF1	2526	2365	1.07	0.09	0.04	0.09
YGL239C		1566	1466	1.07	0.09	0.04	0.09
YNL298W	CLA4	2548	2386	1.07	0.09	0.04	0.09
YDL159W	STE7	5387	5046	1.07	0.09	0.04	0.09
YLR118C		929	870	1.07	0.09	0.04	0.09
YBL083C		1748	1638	1.07	0.09	0.04	0.09
YJL025W	RRN7	2286	2142	1.07	0.09	0.04	0.09
YGR037C	ACB1	2675	2507	1.07	0.09	0.04	0.09
YLL039C	UBI4	4303	4034	1.07	0.09	0.04	0.09
YBL059W		1164	1091	1.07	0.09	0.04	0.09
YBR277C		4700	4408	1.07	0.09	0.04	0.09
YBR248C	HIS7	2876	2698	1.07	0.09	0.04	0.09
YBR071W		3968	3723	1.07	0.09	0.04	0.09
YGL027C	CWH41	4910	4607	1.07	0.09	0.04	0.09
YJL032W		524	492	1.07	0.09	0.04	0.09
YDL138W	RGT2	2825	2652	1.07	0.09	0.03	0.08

YIR037W	HYR1	10727	10072	1.07	0.09	0.03	0.08
YOR132W	VPS17	972	913	1.06	0.09	0.03	0.08
YPR110C	RPC40	3212	3017	1.06	0.09	0.03	0.08
YBL069W	AST1	1521	1429	1.06	0.09	0.03	0.08
YPR123C		3476	3267	1.06	0.09	0.03	0.08
YDR101C		2684	2523	1.06	0.09	0.03	0.08
YKR060W		614	577	1.06	0.09	0.03	0.08
YCRX02C		3612	3395	1.06	0.09	0.03	0.08
YIL066C	RNR3	3386	3183	1.06	0.09	0.03	0.08
YDR357C		1390	1307	1.06	0.09	0.03	0.08
YAL046C		1860	1749	1.06	0.09	0.03	0.08
YIL042C		1283	1207	1.06	0.09	0.03	0.08
YMR244W		1693	1592	1.06	0.09	0.03	0.08
YPR029C	APL4	2879	2708	1.06	0.09	0.03	0.08
YOL105C	WSC3	1298	1221	1.06	0.09	0.03	0.08
YNR067C		2657	2499	1.06	0.09	0.03	0.08
YJL181W		601	565	1.06	0.09	0.03	0.08
YJL134W	LCB3	1609	1514	1.06	0.09	0.03	0.08
YMR247C		2905	2733	1.06	0.09	0.03	0.08
YMR322C		2091	1968	1.06	0.09	0.03	0.08
YGL193C		529	498	1.06	0.09	0.03	0.08
YGR197C	SNG1	754	710	1.06	0.09	0.03	0.08
YDR326C		3628	3415	1.06	0.09	0.03	0.07
YBL036C		3303	3110	1.06	0.09	0.03	0.07
YOR245C		1902	1791	1.06	0.09	0.03	0.07
YBL025W	RRN10	1952	1838	1.06	0.09	0.03	0.07
YAL043C-A		5150	4850	1.06	0.09	0.03	0.07
YFR003C		4799	4520	1.06	0.09	0.03	0.07
YGR200C		5737	5405	1.06	0.09	0.03	0.07
YIR018W	YAP5	1418	1336	1.06	0.09	0.03	0.07
YMR273C	ZDS1	2189	2063	1.06	0.09	0.03	0.07
YGR001C		1534	1446	1.06	0.09	0.03	0.07
YGR153W		1069	1008	1.06	0.09	0.03	0.07
YDL135C	RDI1	3797	3580	1.06	0.08	0.03	0.07
YLR183C		1693	1596	1.06	0.08	0.03	0.07
YBR269C		5710	5387	1.06	0.08	0.03	0.07

YDR425W		2235	2109	1.06	0.08	0.03	0.07
YAR050W	FLO1	7546	7120	1.06	0.08	0.03	0.07
YMR024W	MRPL3	1104	1042	1.06	0.08	0.03	0.07
YDR190C	RVB1	5577	5266	1.06	0.08	0.03	0.06
YDR191W	HST4	1046	988	1.06	0.08	0.03	0.06
YELCdelta4	YELCdelta4	2173	2053	1.06	0.08	0.03	0.06
YBR302C	COS2	2990	2824	1.06	0.08	0.03	0.06
YMR048W		850	803	1.06	0.08	0.03	0.06
YFR006W		2314	2186	1.06	0.08	0.03	0.06
YHR161C	YAP1801	1469	1388	1.06	0.08	0.03	0.06
YLL024C	SSA2	7730	7306	1.06	0.08	0.03	0.06
YJR043C	POL32	603	570	1.06	0.08	0.03	0.06
YOL112W	MSB4	1371	1296	1.06	0.08	0.02	0.06
YGR163W	GTR2	1102	1042	1.06	0.08	0.02	0.06
YGR026W		666	630	1.06	0.08	0.02	0.06
YBLWdelta6	YBLWdelta6	974	921	1.06	0.08	0.02	0.06
YJL192C		2005	1897	1.06	0.08	0.02	0.06
YOL103W	ITR2	2238	2117	1.06	0.08	0.02	0.06
YKR001C	VPS1	1521	1439	1.06	0.08	0.02	0.06
YPL043W	NOP4	4759	4503	1.06	0.08	0.02	0.06
YJL049W		638	604	1.06	0.08	0.02	0.06
YNL172W	APC1	4093	3875	1.06	0.08	0.02	0.05
YPR064W		1343	1272	1.06	0.08	0.02	0.05
YDR092W	UBC13	412	390	1.06	0.08	0.02	0.05
YLR331C		805	763	1.06	0.08	0.02	0.05
YOL119C		3137	2973	1.06	0.08	0.02	0.05
YDR337W	MRPS28	1092	1035	1.06	0.08	0.02	0.05
YGR074W	SMD1	361	342	1.06	0.08	0.02	0.05
YDR393W	SHE9	3174	3008	1.06	0.08	0.02	0.05
YAR018C	KIN3	1917	1817	1.05	0.08	0.02	0.05
YOL151W	GRE2	2039	1933	1.05	0.08	0.02	0.05
YJR150C	DAN1	2011	1907	1.05	0.08	0.02	0.05
YJL118W		1798	1705	1.05	0.08	0.02	0.05
YNL141W	AAH1	3433	3255	1.05	0.08	0.02	0.05
YOL029C		1495	1418	1.05	0.08	0.02	0.05
YOR038C	HIR2	1331	1262	1.05	0.08	0.02	0.05

YBR132C	AGP2	987	936	1.05	0.08	0.02	0.05
YBR263W	SHM1	5824	5527	1.05	0.08	0.02	0.05
YDR442W		1095	1039	1.05	0.08	0.02	0.05
YLR199C		2316	2199	1.05	0.08	0.02	0.05
YDR173C	ARG82	1359	1290	1.05	0.07	0.02	0.04
YJL198W		2531	2403	1.05	0.07	0.02	0.04
YML052W	SUR7	1058	1005	1.05	0.07	0.02	0.04
YKR031C	SPO14	1074	1020	1.05	0.07	0.02	0.04
YGL097W	SRM1	1171	1112	1.05	0.07	0.02	0.04
YHR165C	PRP8	1460	1387	1.05	0.07	0.02	0.04
YDL097C	RPN6	1884	1790	1.05	0.07	0.02	0.04
YKL071W		1988	1889	1.05	0.07	0.02	0.04
YMR229C	RRP5	1135	1078	1.05	0.07	0.02	0.04
YDR408C	ADE8	5759	5472	1.05	0.07	0.02	0.04
YGL016W	KAP122	2460	2338	1.05	0.07	0.02	0.04
YNL257C	SIP3	1213	1153	1.05	0.07	0.02	0.04
YDR111C		1452	1380	1.05	0.07	0.02	0.04
YGL165C		6181	5876	1.05	0.07	0.02	0.04
YAL027W		3445	3275	1.05	0.07	0.02	0.04
YNL330C	RPD3	2322	2208	1.05	0.07	0.02	0.04
YMR001C	CDC5	2760	2624	1.05	0.07	0.02	0.04
YHR201C	PPX1	2048	1947	1.05	0.07	0.02	0.04
YPL009C		2807	2669	1.05	0.07	0.02	0.04
YAR020C	PAU7	3278	3117	1.05	0.07	0.02	0.04
YGL151W	NUT1	4762	4530	1.05	0.07	0.02	0.04
YIL071C		3860	3672	1.05	0.07	0.02	0.04
YOL145C	CTR9	694	660	1.05	0.07	0.02	0.04
YBR103W		2596	2470	1.05	0.07	0.02	0.04
YGR264C	MES1	4665	4439	1.05	0.07	0.02	0.04
YPL032C	SVL3	1464	1393	1.05	0.07	0.02	0.04
YGL060W		1718	1635	1.05	0.07	0.02	0.04
YGL136C		1430	1361	1.05	0.07	0.02	0.04
YKL133C		1263	1202	1.05	0.07	0.02	0.04
YOL037C		1040	990	1.05	0.07	0.02	0.04
YFL044C		1882	1791	1.05	0.07	0.02	0.04
YGL153W	PEX14	1540	1466	1.05	0.07	0.02	0.04

YIL054W		4389	4178	1.05	0.07	0.02	0.04
YGL126W	SCS3	1936	1843	1.05	0.07	0.02	0.04
YIL047C	SYG1	2761	2628	1.05	0.07	0.01	0.04
YHL021C		5695	5421	1.05	0.07	0.01	0.04
YDR444W		2181	2076	1.05	0.07	0.01	0.04
YKL214C		650	619	1.05	0.07	0.01	0.04
YOR009W		966	920	1.05	0.07	0.01	0.04
YER171W	RAD3	1528	1455	1.05	0.07	0.01	0.04
YPL183C		1615	1539	1.05	0.07	0.01	0.03
YMR292W	GOT1	3300	3144	1.05	0.07	0.01	0.03
YPL263C	KEL3	3429	3268	1.05	0.07	0.01	0.03
YLR433C	CNA1	3310	3155	1.05	0.07	0.01	0.03
YOR241W		762	726	1.05	0.07	0.01	0.03
YKL028W	TFA1	1545	1473	1.05	0.07	0.01	0.03
YOL071W		1857	1771	1.05	0.07	0.01	0.03
YBR104W	YMC2	1512	1442	1.05	0.07	0.01	0.03
YLR395C	COX8	384	366	1.05	0.07	0.01	0.03
YPR091C		1556	1484	1.05	0.07	0.01	0.03
YJL146W	IDS2	1524	1454	1.05	0.07	0.01	0.03
YDR232W	HEM1	5117	4881	1.05	0.07	0.01	0.03
YGR147C	NAT2	593	566	1.05	0.07	0.01	0.03
YHL025W	SNF6	2221	2119	1.05	0.07	0.01	0.03
YGL061C	DUO1	615	587	1.05	0.07	0.01	0.03
YGR013W	SNU71	2264	2161	1.05	0.07	0.01	0.03
YMR232W	FUS2	1695	1618	1.05	0.07	0.01	0.03
YKL068W	NUP100	5144	4910	1.05	0.07	0.01	0.03
YKL006W	RPL14A	2509	2396	1.05	0.07	0.01	0.02
YJR151C		2975	2842	1.05	0.07	0.01	0.02
YLR437C		2069	1977	1.05	0.07	0.01	0.02
YBL016W	FUS3	2589	2474	1.05	0.07	0.01	0.02
YLR197W	SIK1	2365	2260	1.05	0.07	0.01	0.02
YLR208W	SEC13	3042	2907	1.05	0.07	0.01	0.02
YER098W	UBP9	523	500	1.05	0.07	0.01	0.02
YGR002C		1207	1154	1.05	0.07	0.01	0.02
YKL109W	HAP4	983	940	1.05	0.06	0.01	0.02
YLR229C	CDC42	1682	1608	1.05	0.06	0.01	0.02

YIL148W	RPL40A	1843	1762	1.05	0.06	0.01	0.02
YBR270C		4222	4038	1.05	0.06	0.01	0.02
YHR097C		659	630	1.05	0.06	0.01	0.02
YKL197C	PEX1	2473	2365	1.05	0.06	0.01	0.02
YBR023C	CHS3	696	666	1.05	0.06	0.01	0.02
YCRX13W		5086	4865	1.05	0.06	0.01	0.02
YGL246C	RAI1	4262	4079	1.04	0.06	0.01	0.02
YHR048W		2482	2376	1.04	0.06	0.01	0.02
YOL078W		5559	5323	1.04	0.06	0.01	0.02
YOR027W	STI1	2468	2363	1.04	0.06	0.01	0.02
YOR196C	LIP5	1730	1657	1.04	0.06	0.01	0.02
YHR218W		11880	11377	1.04	0.06	0.01	0.02
YBL030C	PET9	3635	3482	1.04	0.06	0.01	0.01
YLR130C	ZRT2	2385	2285	1.04	0.06	0.01	0.01
YPR105C		1361	1304	1.04	0.06	0.01	0.01
YGR099W	TEL2	3738	3583	1.04	0.06	0.00	0.01
YOR275C		745	714	1.04	0.06	0.00	0.01
YPL005W		925	887	1.04	0.06	0.00	0.01
YLR273C	PIG1	406	389	1.04	0.06	0.00	0.01
YGL040C	HEM2	2515	2412	1.04	0.06	0.00	0.01
YCRX05W		4688	4498	1.04	0.06	0.00	0.01
YOR197W		2044	1961	1.04	0.06	0.00	0.01
YCR046C	IMG1	2740	2629	1.04	0.06	0.00	0.01
YOL146W		337	323	1.04	0.06	0.00	0.01
YLR007W		852	818	1.04	0.06	0.00	0.01
YHL037C		7159	6870	1.04	0.06	0.00	0.01
YKL198C	PTK1	1412	1355	1.04	0.06	0.00	0.01
YMR044W		1359	1304	1.04	0.06	0.00	0.01
YJR056C		2288	2197	1.04	0.06	0.00	0.01
YDR088C	SLU7	2166	2080	1.04	0.06	0.00	0.01
YCRCdelta6	YCRCdelta6	662	636	1.04	0.06	0.00	0.01
YOL039W	RPP2A	1167	1121	1.04	0.06	0.00	0.01
YMR005W	MPT1	1175	1128	1.04	0.06	0.00	0.01
YJR152W	DAL5	1277	1227	1.04	0.06	0.00	0.00
YGR040W	KSS1	1030	990	1.04	0.06	0.00	0.00
YGR291C		1667	1602	1.04	0.06	0.00	0.00

YBR154C	RPB5	2814	2704	1.04	0.06	0.00	0.00
YLR009W		828	796	1.04	0.06	0.00	0.00
YHR146W		3302	3174	1.04	0.06	0.00	0.00
YDR519W	FKB2	1719	1653	1.04	0.06	0.00	0.00
YOR251C		604	581	1.04	0.06	0.00	0.00
YBL002W	HTB2	5842	5621	1.04	0.06	0.00	0.00
YLR349W		953	917	1.04	0.06	0.00	0.00
YNL187W		498	479	1.04	0.06	0.00	0.00
YKR083C		1075	1035	1.04	0.06	0.00	0.00
YMR049C		3821	3679	1.04	0.05	0.00	0.00
YNR038W	DBP6	3369	3244	1.04	0.05	0.00	0.00
YLL003W	SFI1	1050	1011	1.04	0.05	0.00	0.00
YMR177W	MMT1	1005	968	1.04	0.05	0.00	0.00
YGR260W		6266	6036	1.04	0.05	0.00	-0.01
YDR140W		385	371	1.04	0.05	0.00	-0.01
YIL096C		6043	5826	1.04	0.05	0.00	-0.01
YJR069C	HAM1	601	579	1.04	0.05	0.00	-0.01
YNR032W	PPG1	2463	2375	1.04	0.05	0.00	-0.01
YDR042C		1011	975	1.04	0.05	0.00	-0.01
YOL124C		1755	1692	1.04	0.05	0.00	-0.01
YDR082W	STN1	332	320	1.04	0.05	0.00	-0.01
YLR228C	ECM22	892	861	1.04	0.05	0.00	-0.01
YCR005C	CIT2	3734	3603	1.04	0.05	0.00	-0.01
YMR246W	FAA4	7453	7191	1.04	0.05	0.00	-0.01
YOR358W	HAP5	877	846	1.04	0.05	0.00	-0.01
YML110C	COQ5	2342	2260	1.04	0.05	0.00	-0.01
YAR043C		2872	2772	1.04	0.05	0.00	-0.01
YGR227W	DIE2	1615	1559	1.04	0.05	0.00	-0.01
YPL162C		6359	6138	1.04	0.05	-0.01	-0.01
YBR241C		1420	1371	1.04	0.05	-0.01	-0.01
YPR125W		1527	1474	1.04	0.05	-0.01	-0.01
YGR115C		2091	2019	1.04	0.05	-0.01	-0.01
YNL088W	TOP2	3508	3387	1.04	0.05	-0.01	-0.01
YLR288C	MEC3	1345	1299	1.04	0.05	-0.01	-0.01
YMR195W		458	442	1.04	0.05	-0.01	-0.01
YBR253W	SRB6	3944	3809	1.04	0.05	-0.01	-0.01

YOR051C		1276	1233	1.04	0.05	-0.01	-0.02
YMR318C		1539	1487	1.03	0.05	-0.01	-0.02
YHR214W-A		3479	3362	1.03	0.05	-0.01	-0.02
YHR018C	ARG4	3740	3615	1.03	0.05	-0.01	-0.02
YIR026C	YVH1	2318	2240	1.03	0.05	-0.01	-0.02
YPL086C	ELP3	2195	2122	1.03	0.05	-0.01	-0.02
YNL335W		632	611	1.03	0.05	-0.01	-0.02
YMR316C-A		3201	3095	1.03	0.05	-0.01	-0.02
YKL058W	TOA2	2459	2378	1.03	0.05	-0.01	-0.02
YLL008W	DRS1	3752	3629	1.03	0.05	-0.01	-0.02
YDR289C	RTT103	1288	1246	1.03	0.05	-0.01	-0.02
YMR111C		3729	3608	1.03	0.05	-0.01	-0.02
YPL024W	NCE4	1149	1112	1.03	0.05	-0.01	-0.02
YNL267W	PIK1	3278	3172	1.03	0.05	-0.01	-0.02
YBL019W	APN2	760	736	1.03	0.05	-0.01	-0.02
YDL120W	YFH1	2822	2732	1.03	0.05	-0.01	-0.02
YER077C		563	545	1.03	0.05	-0.01	-0.02
YBR019C	GAL10	1041	1008	1.03	0.05	-0.01	-0.02
YCR030C		5098	4935	1.03	0.05	-0.01	-0.02
YNL002C	RLP7	2678	2593	1.03	0.05	-0.01	-0.02
YGL104C		745	721	1.03	0.05	-0.01	-0.02
YFL026W	STE2	3164	3064	1.03	0.05	-0.01	-0.02
YPL126W	NAN1	5238	5073	1.03	0.05	-0.01	-0.02
YDR499W		1757	1702	1.03	0.05	-0.01	-0.02
YBL103C	RTG3	1302	1261	1.03	0.05	-0.01	-0.02
YJR006W	HYS2	4051	3925	1.03	0.05	-0.01	-0.03
YGR240C	PFK1	8891	8614	1.03	0.05	-0.01	-0.03
YJR141W		1287	1247	1.03	0.05	-0.01	-0.03
YGR071C		3608	3496	1.03	0.05	-0.01	-0.03
YJL099W	CHS6	2063	2000	1.03	0.04	-0.01	-0.03
YLR394W		655	635	1.03	0.04	-0.01	-0.03
YLR179C		1338	1297	1.03	0.04	-0.01	-0.03
YBR272C	HSM3	1422	1378	1.03	0.04	-0.01	-0.03
YCR090C		4779	4633	1.03	0.04	-0.01	-0.03
YDL042C	SIR2	926	898	1.03	0.04	-0.01	-0.03
YIL026C	IRR1	4513	4376	1.03	0.04	-0.01	-0.03

YJL195C		1188	1152	1.03	0.04	-0.01	-0.03
YJL038C		868	842	1.03	0.04	-0.01	-0.03
YOR070C	GYP1	1208	1172	1.03	0.04	-0.01	-0.03
YGL244W	RTF1	1451	1407	1.03	0.04	-0.01	-0.03
YPL214C	THI6	1992	1932	1.03	0.04	-0.01	-0.03
YOR220W		666	646	1.03	0.04	-0.01	-0.03
YPR120C	CLB5	3549	3443	1.03	0.04	-0.01	-0.03
YBL009W		1926	1869	1.03	0.04	-0.01	-0.03
YPL160W	CDC60	5699	5531	1.03	0.04	-0.01	-0.03
YDR055W	PST1	1549	1503	1.03	0.04	-0.01	-0.03
YEL057C		1355	1315	1.03	0.04	-0.01	-0.03
YKR092C	SRP40	1202	1167	1.03	0.04	-0.01	-0.03
YJR160C		539	523	1.03	0.04	-0.01	-0.03
YML029W		1263	1226	1.03	0.04	-0.01	-0.03
YKR002W	PAP1	1861	1807	1.03	0.04	-0.01	-0.03
YMR277W	FCP1	1047	1017	1.03	0.04	-0.01	-0.03
YOL054W		1109	1077	1.03	0.04	-0.01	-0.03
YBR151W	APD1	2189	2126	1.03	0.04	-0.01	-0.03
YPL118W	MRP51	1920	1865	1.03	0.04	-0.01	-0.03
YDL116W	NUP84	2332	2265	1.03	0.04	-0.01	-0.03
YKR004C	ECM9	1189	1155	1.03	0.04	-0.01	-0.03
YJL212C		1305	1268	1.03	0.04	-0.01	-0.03
YGL128C		2320	2254	1.03	0.04	-0.01	-0.04
YMR275C	BUL1	2567	2494	1.03	0.04	-0.01	-0.04
YBR211C	AME1	5405	5252	1.03	0.04	-0.01	-0.04
YDR487C	RIB3	3630	3528	1.03	0.04	-0.01	-0.04
YHR111W		4372	4250	1.03	0.04	-0.02	-0.04
YKR097W	PCK1	2485	2417	1.03	0.04	-0.02	-0.04
YNL125C	ESBP6	2988	2907	1.03	0.04	-0.02	-0.04
YEL037C	RAD23	2675	2602	1.03	0.04	-0.02	-0.04
YNL275W		2387	2322	1.03	0.04	-0.02	-0.04
YIL131C	FKH1	1577	1534	1.03	0.04	-0.02	-0.04
YBL010C		1101	1071	1.03	0.04	-0.02	-0.04
YER010C		645	628	1.03	0.04	-0.02	-0.04
YGR131W		454	442	1.03	0.04	-0.02	-0.04
YPR030W	CSR2	5001	4868	1.03	0.04	-0.02	-0.04

YMR117C	SPC24	3109	3027	1.03	0.04	-0.02	-0.04
YML121W	GTR1	2018	1965	1.03	0.04	-0.02	-0.04
YOR254C	SEC63	1267	1233	1.03	0.04	-0.02	-0.04
YGR211W	ZPR1	3343	3255	1.03	0.04	-0.02	-0.04
YCLX12W		1590	1548	1.03	0.04	-0.02	-0.04
YJL127C	SPT10	3646	3550	1.03	0.04	-0.02	-0.04
YJL021C		1796	1749	1.03	0.04	-0.02	-0.04
YLR438W	CAR2	3785	3687	1.03	0.04	-0.02	-0.04
YDL092W	SRP14	1147	1117	1.03	0.04	-0.02	-0.04
YPL014W		2401	2339	1.03	0.04	-0.02	-0.04
YBR240C	THI2	1098	1070	1.03	0.04	-0.02	-0.04
YNL154C	YCK2	787	767	1.03	0.04	-0.02	-0.05
YDR145W	TAF61	2250	2193	1.03	0.04	-0.02	-0.05
YBL111C		7942	7742	1.03	0.04	-0.02	-0.05
YGR178C	PBP1	5336	5202	1.03	0.04	-0.02	-0.05
YPL248C	GAL4	5876	5729	1.03	0.04	-0.02	-0.05
YDL165W	CDC36	1529	1491	1.03	0.04	-0.02	-0.05
YPL271W	ATP15	791	771	1.03	0.04	-0.02	-0.05
YLL038C	ENT4	989	965	1.03	0.04	-0.02	-0.05
YOR364W		561	547	1.02	0.04	-0.02	-0.05
YMR148W		1047	1022	1.02	0.04	-0.02	-0.05
YPL043W	NOP4	487	475	1.02	0.04	-0.02	-0.05
YKL045W	PRI2	1099	1073	1.02	0.04	-0.02	-0.05
YNL069C	RPL16B	1110	1084	1.02	0.03	-0.02	-0.05
YIR025W		662	647	1.02	0.03	-0.02	-0.05
YKR058W	GLG1	1079	1054	1.02	0.03	-0.02	-0.05
YJL087C	TRL1	1356	1325	1.02	0.03	-0.02	-0.05
YBR244W	AMI1	2654	2594	1.02	0.03	-0.02	-0.06
YAR061W		9263	9053	1.02	0.03	-0.02	-0.06
YKR067W		1710	1671	1.02	0.03	-0.02	-0.06
YOR114W		515	503	1.02	0.03	-0.02	-0.06
YBL015W	ACH1	1535	1501	1.02	0.03	-0.02	-0.06
YOR310C	NOP58	2265	2215	1.02	0.03	-0.02	-0.06
YBR096W		1310	1281	1.02	0.03	-0.02	-0.06
YKLCdelta2	YKLCdelta2	1086	1062	1.02	0.03	-0.02	-0.06
YOR309C		3124	3055	1.02	0.03	-0.02	-0.06

YLL063C	AYT1	2348	2296	1.02	0.03	-0.02	-0.06
YJR064W	CCT5	1723	1685	1.02	0.03	-0.02	-0.06
YEL063C	CAN1	3312	3239	1.02	0.03	-0.02	-0.06
YJL186W	MNN5	1728	1690	1.02	0.03	-0.02	-0.06
YCR101C		3470	3395	1.02	0.03	-0.02	-0.06
YDL175C		1662	1627	1.02	0.03	-0.02	-0.06
YOR341W	RPA190	6589	6449	1.02	0.03	-0.03	-0.06
YAL014C		4198	4109	1.02	0.03	-0.03	-0.06
YLR235C		1706	1670	1.02	0.03	-0.03	-0.06
YNL250W	RAD50	1574	1541	1.02	0.03	-0.03	-0.06
YDL058W	USO1	2056	2013	1.02	0.03	-0.03	-0.06
YIL067C		998	977	1.02	0.03	-0.03	-0.06
YML065W	ORC1	3936	3855	1.02	0.03	-0.03	-0.06
YML131W		1185	1161	1.02	0.03	-0.03	-0.06
YDL134C-A		2043	2001	1.02	0.03	-0.03	-0.06
YML062C	MFT1	1613	1580	1.02	0.03	-0.03	-0.06
YMR031C		2321	2274	1.02	0.03	-0.03	-0.06
YKL221W		903	885	1.02	0.03	-0.03	-0.06
YMRCdelta11	YMRCdelta11	1650	1617	1.02	0.03	-0.03	-0.06
YLR337C	YLR337C	649	636	1.02	0.03	-0.03	-0.06
YML127W		1231	1207	1.02	0.03	-0.03	-0.07
YML035C	AMD1	5343	5239	1.02	0.03	-0.03	-0.07
YDL145C	COP1	5347	5244	1.02	0.03	-0.03	-0.07
YLR416C		1453	1425	1.02	0.03	-0.03	-0.07
YMR276W	DSK2	529	519	1.02	0.03	-0.03	-0.07
YCR018C	SRD1	2787	2734	1.02	0.03	-0.03	-0.07
YAR064W		4363	4280	1.02	0.03	-0.03	-0.07
YPR090W		1626	1596	1.02	0.03	-0.03	-0.07
YOR085W	OST3	1612	1582	1.02	0.03	-0.03	-0.07
YDL085W		2182	2142	1.02	0.03	-0.03	-0.07
YDL108W	KIN28	1847	1813	1.02	0.03	-0.03	-0.07
YIL075C	RPN2	8394	8243	1.02	0.03	-0.03	-0.07
YDR441C	APT2	4353	4275	1.02	0.03	-0.03	-0.07
YJR071W		1241	1219	1.02	0.03	-0.03	-0.07
YGL025C	PGD1	1452	1426	1.02	0.03	-0.03	-0.07
YOR092W	ECM3	889	873	1.02	0.03	-0.03	-0.07

YGL257C	MNT2	1534	1507	1.02	0.03	-0.03	-0.07
YCR026C		2141	2103	1.02	0.03	-0.03	-0.07
YLL047W		3480	3419	1.02	0.03	-0.03	-0.07
YHR155W		842	827	1.02	0.03	-0.03	-0.07
YER029C	SMB1	1600	1572	1.02	0.03	-0.03	-0.08
YER003C	PMI40	2561	2517	1.02	0.02	-0.03	-0.08
YFR040W	SAP155	5710	5613	1.02	0.02	-0.03	-0.08
YIL094C	LYS12	960	944	1.02	0.02	-0.03	-0.08
YDR266C		1946	1913	1.02	0.02	-0.03	-0.08
YCL076W		2046	2012	1.02	0.02	-0.03	-0.08
YDR346C		5070	4985	1.02	0.02	-0.03	-0.08
YNL311C		2584	2541	1.02	0.02	-0.03	-0.08
YPR058W	YMC1	843	829	1.02	0.02	-0.03	-0.08
YBR301W		5643	5551	1.02	0.02	-0.03	-0.08
YGL130W	CEG1	2037	2004	1.02	0.02	-0.03	-0.08
YMR289W		2529	2489	1.02	0.02	-0.03	-0.08
YGR145W		1876	1846	1.02	0.02	-0.03	-0.08
YIL056W		2646	2604	1.02	0.02	-0.03	-0.08
YJR041C		1966	1936	1.02	0.02	-0.03	-0.08
YNL287W	SEC21	6137	6042	1.02	0.02	-0.03	-0.08
YIL166C		1707	1681	1.02	0.02	-0.03	-0.08
YCL033C		5142	5064	1.02	0.02	-0.03	-0.08
YKL127W	PGM1	5438	5355	1.02	0.02	-0.03	-0.08
YDL153C	SAS10	4197	4134	1.02	0.02	-0.03	-0.08
YLL007C		1793	1767	1.01	0.02	-0.03	-0.08
YGL022W	STT3	3395	3346	1.01	0.02	-0.04	-0.08
YPR093C		1143	1127	1.01	0.02	-0.04	-0.09
YGR024C		718	708	1.01	0.02	-0.04	-0.09
YDL178W	AIP2	2322	2290	1.01	0.02	-0.04	-0.09
YLR138W	NHA1	930	917	1.01	0.02	-0.04	-0.09
YJR027W		829	818	1.01	0.02	-0.04	-0.09
YMR096W	SNZ1	2338	2306	1.01	0.02	-0.04	-0.09
YLR142W	PUT1	1253	1236	1.01	0.02	-0.04	-0.09
YGR105W	VMA21	2624	2588	1.01	0.02	-0.04	-0.09
YGL232W		1084	1069	1.01	0.02	-0.04	-0.09
YDR482C		4041	3986	1.01	0.02	-0.04	-0.09

YNR052C	POP2	3007	2966	1.01	0.02	-0.04	-0.09
YJL079C	PRY1	865	853	1.01	0.02	-0.04	-0.09
YOR164C		597	589	1.01	0.02	-0.04	-0.09
YBR201W	DER1	1851	1827	1.01	0.02	-0.04	-0.09
YIL142W	CCT2	2601	2567	1.01	0.02	-0.04	-0.09
YNL243W	SLA2	3122	3082	1.01	0.02	-0.04	-0.09
YCL024W		4010	3961	1.01	0.02	-0.04	-0.09
YGL214W		1414	1397	1.01	0.02	-0.04	-0.09
YMR019W	STB4	2844	2810	1.01	0.02	-0.04	-0.09
YOL160W		881	871	1.01	0.02	-0.04	-0.09
YAR047C		2514	2484	1.01	0.02	-0.04	-0.09
YLR277C	YSH1	929	918	1.01	0.02	-0.04	-0.10
YPR194C		2785	2753	1.01	0.02	-0.04	-0.10
YNL009W	IDP3	1610	1592	1.01	0.02	-0.04	-0.10
YKL126W	YPK1	1149	1136	1.01	0.02	-0.04	-0.10
YHRCTy1-1B	YHRCTy1-1B	5119	5062	1.01	0.02	-0.04	-0.10
YCR025C		1000	989	1.01	0.02	-0.04	-0.10
YPR067W	ISA2	3758	3717	1.01	0.02	-0.04	-0.10
YFR033C	QCR6	2143	2120	1.01	0.02	-0.04	-0.10
YMR306C-A		3492	3455	1.01	0.02	-0.04	-0.10
YMR304W	UBP15	757	749	1.01	0.01	-0.04	-0.10
YBL056W	PTC3	7128	7055	1.01	0.01	-0.04	-0.10
YKR013W	PRY2	874	866	1.01	0.01	-0.04	-0.10
YDL118W		564	559	1.01	0.01	-0.04	-0.10
YHR034C		2384	2362	1.01	0.01	-0.04	-0.10
YKL032C	IXR1	1510	1496	1.01	0.01	-0.04	-0.10
YBLWTy2-1A	YBLWTy2-1A	16228	16079	1.01	0.01	-0.04	-0.10
YCR002C	CDC10	3031	3003	1.01	0.01	-0.04	-0.10
YFL019C		1012	1003	1.01	0.01	-0.04	-0.10
YMR222C		2157	2137	1.01	0.01	-0.04	-0.10
YJR013W		1568	1554	1.01	0.01	-0.04	-0.10
YIL159W	BNR1	2436	2415	1.01	0.01	-0.04	-0.11
YCL028W	RNQ1	4187	4151	1.01	0.01	-0.04	-0.11
YNR068C		1177	1167	1.01	0.01	-0.04	-0.11
YHR078W		706	700	1.01	0.01	-0.04	-0.11
YPL103C		965	957	1.01	0.01	-0.04	-0.11

YPL140C	MKK2	1176	1166	1.01	0.01	-0.04	-0.11
YNL122C		573	568	1.01	0.01	-0.04	-0.11
YNR028W	CPR8	1043	1034	1.01	0.01	-0.04	-0.11
YLR106C		2272	2253	1.01	0.01	-0.04	-0.11
YGL170C		2735	2713	1.01	0.01	-0.04	-0.11
YGL168W		1120	1111	1.01	0.01	-0.04	-0.11
YDR090C		1288	1278	1.01	0.01	-0.04	-0.11
YPR159W	KRE6	3748	3718	1.01	0.01	-0.04	-0.11
YMR092C	AIP1	3308	3282	1.01	0.01	-0.04	-0.11
YKLCdelta4	YKLCdelta4	1328	1318	1.01	0.01	-0.04	-0.11
YLR379W		829	823	1.01	0.01	-0.04	-0.11
YOL150C		432	429	1.01	0.01	-0.05	-0.11
YLR149C		3557	3531	1.01	0.01	-0.05	-0.11
YER074W	RPS24A	3208	3184	1.01	0.01	-0.05	-0.11
YOR126C	IAH1	731	726	1.01	0.01	-0.05	-0.11
YGL008C	PMA1	7880	7823	1.01	0.01	-0.05	-0.11
YER158C		809	803	1.01	0.01	-0.05	-0.11
YIR010W		2252	2236	1.01	0.01	-0.05	-0.11
YIR028W	DAL4	557	553	1.01	0.01	-0.05	-0.11
YGL146C		665	660	1.01	0.01	-0.05	-0.11
YMR157C		2066	2052	1.01	0.01	-0.05	-0.11
YPR066W	UBA3	1127	1120	1.01	0.01	-0.05	-0.11
YOR033C	DHS1	1520	1510	1.01	0.01	-0.05	-0.11
YDR194C	MSS116	881	876	1.01	0.01	-0.05	-0.11
YGR146C		6706	6665	1.01	0.01	-0.05	-0.11
YIL040W		2355	2341	1.01	0.01	-0.05	-0.11
YGR023W	MTL1	1534	1525	1.01	0.01	-0.05	-0.11
YKL005C		2609	2594	1.01	0.01	-0.05	-0.11
YMR124W		2644	2629	1.01	0.01	-0.05	-0.12
YLR178C	TFS1	587	584	1.01	0.01	-0.05	-0.12
YCL045C		2188	2176	1.01	0.01	-0.05	-0.12
YBR068C	BAP2	3065	3048	1.01	0.01	-0.05	-0.12
YDR474C		3335	3317	1.01	0.01	-0.05	-0.12
YGR173W		1326	1319	1.01	0.01	-0.05	-0.12
YDL063C		1609	1601	1.01	0.01	-0.05	-0.12
YDR259C	YAP6	1603	1595	1.01	0.01	-0.05	-0.12

YMR309C	NIP1	1804	1795	1.01	0.01	-0.05	-0.12
YMR238W	DFG5	886	882	1.00	0.01	-0.05	-0.12
YLR365W		1145	1140	1.00	0.01	-0.05	-0.12
YGL256W	ADH4	2623	2611	1.00	0.01	-0.05	-0.12
YJL180C	ATP12	1372	1366	1.00	0.01	-0.05	-0.12
YML106W	URA5	2537	2526	1.00	0.01	-0.05	-0.12
YML130C	ERO1	1771	1764	1.00	0.01	-0.05	-0.12
YGR057C	LST7	4117	4100	1.00	0.01	-0.05	-0.12
YJL024C	APS3	1540	1534	1.00	0.01	-0.05	-0.12
YBL031W	SHE1	1039	1035	1.00	0.01	-0.05	-0.12
YJR131W	MNS1	1330	1325	1.00	0.00	-0.05	-0.12
YKL129C	MYO3	2571	2562	1.00	0.00	-0.05	-0.12
YDL225W	SHS1	1282	1278	1.00	0.00	-0.05	-0.12
YDR310C	SUM1	3800	3787	1.00	0.00	-0.05	-0.12
YML074C	NPI46	1702	1697	1.00	0.00	-0.05	-0.12
YLR217W		829	827	1.00	0.00	-0.05	-0.13
YOL077C		1285	1281	1.00	0.00	-0.05	-0.13
YGR126W		1185	1182	1.00	0.00	-0.05	-0.13
YLR458W		5237	5222	1.00	0.00	-0.05	-0.13
YGL019W	CKB1	2629	2622	1.00	0.00	-0.05	-0.13
YDR023W	SES1	1498	1494	1.00	0.00	-0.05	-0.13
YPR174C		1672	1668	1.00	0.00	-0.05	-0.13
YGR124W	ASN2	6388	6373	1.00	0.00	-0.05	-0.13
YIL003W		1886	1882	1.00	0.00	-0.05	-0.13
YNL089C		512	511	1.00	0.00	-0.05	-0.13
YIL133C	RPL16A	3461	3454	1.00	0.00	-0.05	-0.13
YLR449W	FPR4	5460	5451	1.00	0.00	-0.05	-0.13
YLR020C		2521	2517	1.00	0.00	-0.05	-0.13
YHR149C		1188	1186	1.00	0.00	-0.05	-0.13
YLR244C	MAP1	3084	3080	1.00	0.00	-0.05	-0.13
YIL008W		2447	2444	1.00	0.00	-0.05	-0.13
YPR031W		1625	1623	1.00	0.00	-0.05	-0.13
YMR193C-A		561	560	1.00	0.00	-0.05	-0.13
YPL170W		2574	2572	1.00	0.00	-0.05	-0.13
YCR010C		340	340	1.00	0.00	-0.06	-0.13
YHR069C	RRP4	1908	1907	1.00	0.00	-0.06	-0.13

YFR001W		1346	1346	1.00	0.00	-0.06	-0.13
YML117W-A		1299	1299	1.00	0.00	-0.06	-0.13
YEL010W		1793	1793	1.00	0.00	-0.06	-0.14
YJR020W		672	672	1.00	0.00	-0.06	-0.14
YER108C		1377	1377	1.00	0.00	-0.06	-0.14
YHR038W	FIL1	4864	4865	1.00	0.00	-0.06	-0.14
YDR541C		757	757	1.00	0.00	-0.06	-0.14
YER151C	UBP3	6707	6709	1.00	0.00	-0.06	-0.14
YER185W		492	492	1.00	0.00	-0.06	-0.14
YBR284W		536	537	1.00	0.00	-0.06	-0.14
YMR241W	YHM2	3132	3136	1.00	0.00	-0.06	-0.14
YNL139C	RLR1	930	931	1.00	0.00	-0.06	-0.14
YBL070C		6375	6388	1.00	0.00	-0.06	-0.14
YMRCtau1	YMRCtau1	1742	1746	1.00	0.00	-0.06	-0.14
YJL217W		670	671	1.00	0.00	-0.06	-0.14
YDL082W	RPL13A	6288	6302	1.00	0.00	-0.06	-0.14
YER070W	RNR1	5351	5363	1.00	0.00	-0.06	-0.14
YDL187C		3331	3339	1.00	0.00	-0.06	-0.14
YML048W	GSF2	1833	1838	1.00	0.00	-0.06	-0.14
YHR037W	PUT2	3486	3496	1.00	0.00	-0.06	-0.15
YDR200C		2010	2016	1.00	0.00	-0.06	-0.15
YPR098C		1095	1098	1.00	0.00	-0.06	-0.15
YBR119W	MUD1	1653	1658	1.00	0.00	-0.06	-0.15
YNL224C		1633	1639	1.00	0.00	-0.06	-0.15
YBL087C	RPL23A	3011	3022	1.00	-0.01	-0.06	-0.15
YCL061C		1497	1502	1.00	-0.01	-0.06	-0.15
YHR003C		1472	1478	1.00	-0.01	-0.06	-0.15
YLR397C	AFG2	2792	2803	1.00	-0.01	-0.06	-0.15
YCL031C	RRP7	1741	1748	1.00	-0.01	-0.06	-0.15
YOR261C	RPN8	1639	1646	1.00	-0.01	-0.06	-0.15
YBR250W		943	947	1.00	-0.01	-0.06	-0.15
YHR084W	STE12	1970	1978	1.00	-0.01	-0.06	-0.15
YCR091W	KIN82	1600	1606	1.00	-0.01	-0.06	-0.15
YGL241W	KAP114	769	772	1.00	-0.01	-0.06	-0.15
YML061C	PIF1	1133	1138	1.00	-0.01	-0.06	-0.15
YPR096C		2361	2371	1.00	-0.01	-0.06	-0.15

YPL130W	SPO19	8721	8757	1.00	-0.01	-0.06	-0.15
YLR354C	TAL1	2199	2209	1.00	-0.01	-0.06	-0.15
YOR021C		1493	1500	1.00	-0.01	-0.06	-0.15
YDR219C		1515	1522	1.00	-0.01	-0.06	-0.15
YLR260W	LCB5	3359	3375	1.00	-0.01	-0.06	-0.15
YGL077C	HNM1	1346	1353	1.00	-0.01	-0.06	-0.15
YDR207C	UME6	1983	1993	1.00	-0.01	-0.06	-0.15
YEL061C	CIN8	547	550	1.00	-0.01	-0.06	-0.15
YER079W		558	561	1.00	-0.01	-0.06	-0.15
YBR294W	SUL1	1863	1873	0.99	-0.01	-0.06	-0.15
YPR112C	MRD1	5324	5353	0.99	-0.01	-0.06	-0.15
YOR326W	MYO2	2895	2911	0.99	-0.01	-0.06	-0.15
YNL318C	HXT14	840	845	0.99	-0.01	-0.06	-0.15
YDL162C		1252	1259	0.99	-0.01	-0.06	-0.15
YGR202C	PCT1	4078	4101	0.99	-0.01	-0.06	-0.16
YEL029C		2062	2074	0.99	-0.01	-0.06	-0.16
YER109C	FLO8	3709	3731	0.99	-0.01	-0.06	-0.16
YDL037C		5219	5250	0.99	-0.01	-0.06	-0.16
YNL024C		3216	3236	0.99	-0.01	-0.07	-0.16
YGR012W		1339	1348	0.99	-0.01	-0.07	-0.16
YCR055C		2573	2590	0.99	-0.01	-0.07	-0.16
YPL034W		1156	1164	0.99	-0.01	-0.07	-0.16
YDL222C		741	746	0.99	-0.01	-0.07	-0.16
YMR197C	VTI1	1989	2003	0.99	-0.01	-0.07	-0.16
YKR082W	NUP133	2304	2320	0.99	-0.01	-0.07	-0.16
YOR014W	RTS1	2781	2801	0.99	-0.01	-0.07	-0.16
YIL177C		7643	7697	0.99	-0.01	-0.07	-0.16
YNL040W		661	666	0.99	-0.01	-0.07	-0.16
YJL102W	MEF2	2423	2441	0.99	-0.01	-0.07	-0.16
YBR198C	TAF90	3401	3427	0.99	-0.01	-0.07	-0.16
YOR037W	CYC2	2734	2755	0.99	-0.01	-0.07	-0.16
YKL130C	SHE2	598	603	0.99	-0.01	-0.07	-0.16
YJL050W	MTR4	3969	4002	0.99	-0.01	-0.07	-0.16
YKL042W	SPC42	3201	3228	0.99	-0.01	-0.07	-0.16
YDR427W	RPN9	1074	1083	0.99	-0.01	-0.07	-0.16
YMR323W		1408	1420	0.99	-0.01	-0.07	-0.16

YHR002W		3955	3988	0.99	-0.01	-0.07	-0.16
YHR039C-B		2240	2259	0.99	-0.01	-0.07	-0.16
YFL036W	RPO41	2502	2523	0.99	-0.01	-0.07	-0.17
YLR062C		1012	1021	0.99	-0.01	-0.07	-0.17
YPL219W	PCL8	1338	1350	0.99	-0.01	-0.07	-0.17
YNR076W	PAU6	2810	2836	0.99	-0.01	-0.07	-0.17
YAL060W		1390	1403	0.99	-0.01	-0.07	-0.17
YML119W		1024	1034	0.99	-0.01	-0.07	-0.17
YOR213C	SAS5	1696	1713	0.99	-0.01	-0.07	-0.17
YBL042C	FUI1	4829	4877	0.99	-0.01	-0.07	-0.17
YDL209C		2204	2226	0.99	-0.01	-0.07	-0.17
YLR205C		3998	4039	0.99	-0.01	-0.07	-0.17
YER173W	RAD24	6709	6780	0.99	-0.02	-0.07	-0.17
YGR008C	STF2	1067	1078	0.99	-0.02	-0.07	-0.17
YMR139W	RIM11	1872	1892	0.99	-0.02	-0.07	-0.17
YLR269C		325	328	0.99	-0.02	-0.07	-0.17
YNL164C		871	880	0.99	-0.02	-0.07	-0.17
YJR133W	XPT1	746	754	0.99	-0.02	-0.07	-0.17
YDR524C		1413	1429	0.99	-0.02	-0.07	-0.17
YHR116W		1477	1494	0.99	-0.02	-0.07	-0.17
YGR076C	MRPL25	895	905	0.99	-0.02	-0.07	-0.17
YPL075W	GCR1	1762	1782	0.99	-0.02	-0.07	-0.18
YCR103C		967	979	0.99	-0.02	-0.07	-0.18
YFL023W		2778	2811	0.99	-0.02	-0.07	-0.18
YLR187W		2492	2522	0.99	-0.02	-0.07	-0.18
YLL067C		12923	13080	0.99	-0.02	-0.07	-0.18
YNL235C		929	940	0.99	-0.02	-0.07	-0.18
YGL139W		4640	4697	0.99	-0.02	-0.07	-0.18
YMR113W		627	635	0.99	-0.02	-0.07	-0.18
YCR082W		2299	2328	0.99	-0.02	-0.07	-0.18
YDR058C	TGL2	1286	1302	0.99	-0.02	-0.07	-0.18
YPL146C		4183	4236	0.99	-0.02	-0.07	-0.18
YMR299C		2136	2163	0.99	-0.02	-0.07	-0.18
YMR228W	MTF1	610	618	0.99	-0.02	-0.07	-0.18
YHL024W	NOS1	363	368	0.99	-0.02	-0.07	-0.18
YPL089C	RLM1	3959	4012	0.99	-0.02	-0.08	-0.18

YMR063W	RIM9	2005	2032	0.99	-0.02	-0.08	-0.18
YGL152C		954	967	0.99	-0.02	-0.08	-0.18
YHR200W	RPN10	2094	2122	0.99	-0.02	-0.08	-0.18
YPL210C	SRP72	3332	3378	0.99	-0.02	-0.08	-0.18
YDL163W		5305	5379	0.99	-0.02	-0.08	-0.18
YJR113C		1945	1972	0.99	-0.02	-0.08	-0.18
YIR033W	MGA2	2682	2720	0.99	-0.02	-0.08	-0.18
YKR016W		1374	1394	0.99	-0.02	-0.08	-0.19
YHRWtau3	YHRWtau3	1660	1684	0.99	-0.02	-0.08	-0.19
YER060W	FCY21	1960	1989	0.99	-0.02	-0.08	-0.19
YJL098W	SAP185	3149	3196	0.99	-0.02	-0.08	-0.19
YMR163C		677	687	0.99	-0.02	-0.08	-0.19
YLR210W	CLB4	693	703	0.99	-0.02	-0.08	-0.19
YGL142C	GPI10	2164	2197	0.98	-0.02	-0.08	-0.19
YDL074C		929	943	0.98	-0.02	-0.08	-0.19
YOL002C		401	407	0.98	-0.02	-0.08	-0.19
YPL108W		728	739	0.98	-0.02	-0.08	-0.19
YMR014W		8009	8136	0.98	-0.02	-0.08	-0.19
YBR162C		9778	9935	0.98	-0.02	-0.08	-0.19
YLL031C		3063	3113	0.98	-0.02	-0.08	-0.19
YBR084W	MIS1	4173	4242	0.98	-0.02	-0.08	-0.19
YOR316C	COT1	1833	1864	0.98	-0.02	-0.08	-0.19
YKL016C	ATP7	1068	1086	0.98	-0.02	-0.08	-0.19
YLL016W	SDC25	1212	1233	0.98	-0.02	-0.08	-0.20
YDR156W	RPA14	1170	1190	0.98	-0.02	-0.08	-0.20
YDR527W		10901	11092	0.98	-0.03	-0.08	-0.20
YHRCdelta5	YHRCdelta5	631	642	0.98	-0.03	-0.08	-0.20
YBL028C		4776	4860	0.98	-0.03	-0.08	-0.20
YNL271C	BNI1	2428	2471	0.98	-0.03	-0.08	-0.20
YOL011W	PLB3	2605	2651	0.98	-0.03	-0.08	-0.20
YCR085W		10435	10623	0.98	-0.03	-0.08	-0.20
YLR047C		3453	3516	0.98	-0.03	-0.08	-0.20
YHL041W		2921	2974	0.98	-0.03	-0.08	-0.20
YPR045C		2032	2069	0.98	-0.03	-0.08	-0.20
YJL080C	SCP160	2690	2740	0.98	-0.03	-0.08	-0.20
YER156C		1729	1761	0.98	-0.03	-0.08	-0.20

YJR088C		2158	2198	0.98	-0.03	-0.08	-0.20
YML038C	YMD8	2361	2406	0.98	-0.03	-0.08	-0.20
YDRCTy1-2B	YDRCTy1-2B	20412	20809	0.98	-0.03	-0.08	-0.20
YNR060W	FRE4	1343	1369	0.98	-0.03	-0.08	-0.20
YHR045W		392	400	0.98	-0.03	-0.08	-0.20
YJL034W	KAR2	7040	7178	0.98	-0.03	-0.08	-0.20
YKR049C		1527	1557	0.98	-0.03	-0.08	-0.20
YDR477W	SNF1	1779	1814	0.98	-0.03	-0.08	-0.20
YLR262C-A	YLR262C-A	948	967	0.98	-0.03	-0.08	-0.20
YLR122C		613	625	0.98	-0.03	-0.08	-0.20
YER153C	PET122	1333	1360	0.98	-0.03	-0.08	-0.21
YKR098C	UBP11	1848	1885	0.98	-0.03	-0.08	-0.21
YOL148C	SPT20	2362	2410	0.98	-0.03	-0.08	-0.21
YPL269W	KAR9	1338	1365	0.98	-0.03	-0.08	-0.21
YBL046W		4257	4345	0.98	-0.03	-0.09	-0.21
YPL150W		4122	4208	0.98	-0.03	-0.09	-0.21
YDR057W		7713	7876	0.98	-0.03	-0.09	-0.21
YGR029W	ERV1	1633	1668	0.98	-0.03	-0.09	-0.21
YJL107C		896	915	0.98	-0.03	-0.09	-0.21
YOR099W	KTR1	1437	1468	0.98	-0.03	-0.09	-0.21
YGL018C	JAC1	1017	1039	0.98	-0.03	-0.09	-0.21
YHR147C	MRPL6	4365	4460	0.98	-0.03	-0.09	-0.21
YOLWtau1	YOLWtau1	567	579	0.98	-0.03	-0.09	-0.21
YLR291C	GCD7	2888	2951	0.98	-0.03	-0.09	-0.21
YOL114C		1862	1903	0.98	-0.03	-0.09	-0.21
YIL072W	HOP1	2020	2064	0.98	-0.03	-0.09	-0.21
YGL087C	MMS2	1407	1438	0.98	-0.03	-0.09	-0.21
YLL059C		2023	2068	0.98	-0.03	-0.09	-0.21
YDR503C	LPP1	1589	1624	0.98	-0.03	-0.09	-0.21
YLR116W	MSL5	1807	1847	0.98	-0.03	-0.09	-0.21
YDR420W	HKR1	11111	11360	0.98	-0.03	-0.09	-0.21
YPL186C		1165	1191	0.98	-0.03	-0.09	-0.21
YER075C	PTP3	2115	2163	0.98	-0.03	-0.09	-0.21
YPR072W	NOT5	6324	6468	0.98	-0.03	-0.09	-0.21
YOR391C		487	498	0.98	-0.03	-0.09	-0.22
YDR358W		1034	1058	0.98	-0.03	-0.09	-0.22

YLR030W		942	964	0.98	-0.03	-0.09	-0.22
YDR450W	RPS18A	2720	2783	0.98	-0.03	-0.09	-0.22
YCR051W		2292	2346	0.98	-0.03	-0.09	-0.22
YGR244C	LSC2	4984	5101	0.98	-0.03	-0.09	-0.22
YBL075C	SSA3	1816	1859	0.98	-0.03	-0.09	-0.22
YKL050C		3021	3093	0.98	-0.03	-0.09	-0.22
YDR401W		454	465	0.98	-0.03	-0.09	-0.22
YGL026C	TRP5	2854	2923	0.98	-0.03	-0.09	-0.22
YMR212C		1078	1104	0.98	-0.03	-0.09	-0.22
YDL099W		1652	1692	0.98	-0.03	-0.09	-0.22
YCL013W		4027	4126	0.98	-0.03	-0.09	-0.22
YFR050C	PRE4	4357	4464	0.98	-0.04	-0.09	-0.22
YPL242C	IQG1	2900	2972	0.98	-0.04	-0.09	-0.22
YDR126W		467	479	0.98	-0.04	-0.09	-0.22
YOL085C		482	494	0.98	-0.04	-0.09	-0.22
YAL015C	NTG1	1454	1491	0.98	-0.04	-0.09	-0.22
YGL216W	KIP3	2367	2428	0.97	-0.04	-0.09	-0.22
YPL166W		1842	1889	0.97	-0.04	-0.09	-0.22
YDL008W	APC11	3155	3237	0.97	-0.04	-0.09	-0.22
YKL113C	RAD27	1418	1455	0.97	-0.04	-0.09	-0.23
YLL064C		459	471	0.97	-0.04	-0.09	-0.23
YHR212C		1778	1825	0.97	-0.04	-0.09	-0.23
YIL057C		1109	1139	0.97	-0.04	-0.09	-0.23
YMR218C	TRS130	671	689	0.97	-0.04	-0.09	-0.23
YLL062C		1366	1403	0.97	-0.04	-0.09	-0.23
YLR403W	SFP1	2178	2238	0.97	-0.04	-0.10	-0.23
YDR375C	BCS1	1333	1370	0.97	-0.04	-0.10	-0.23
YKL027W		1120	1151	0.97	-0.04	-0.10	-0.23
YPL231W	FAS2	3871	3979	0.97	-0.04	-0.10	-0.23
YOR120W	GCY1	619	636	0.97	-0.04	-0.10	-0.23
YDR247W		1302	1338	0.97	-0.04	-0.10	-0.23
YCL058C		2097	2156	0.97	-0.04	-0.10	-0.23
YDR158W	HOM2	4726	4860	0.97	-0.04	-0.10	-0.23
YKR093W	PTR2	1807	1858	0.97	-0.04	-0.10	-0.23
YDR483W	KRE2	3240	3333	0.97	-0.04	-0.10	-0.23
YDR122W	KIN1	1509	1552	0.97	-0.04	-0.10	-0.23

YJL054W	TIM54	2896	2980	0.97	-0.04	-0.10	-0.23
YDR223W		1103	1135	0.97	-0.04	-0.10	-0.23
YPR129W	SCD6	2516	2589	0.97	-0.04	-0.10	-0.23
YOR040W	GLO4	2635	2712	0.97	-0.04	-0.10	-0.24
YBR107C	MCM19	334	344	0.97	-0.04	-0.10	-0.24
YOR116C	RPO31	889	915	0.97	-0.04	-0.10	-0.24
YDR386W	MUS81	624	642	0.97	-0.04	-0.10	-0.24
YFR032C		1333	1373	0.97	-0.04	-0.10	-0.24
YMR072W	ABF2	637	656	0.97	-0.04	-0.10	-0.24
YJL056C	ZAP1	2183	2249	0.97	-0.04	-0.10	-0.24
YGL235W		2094	2157	0.97	-0.04	-0.10	-0.24
YOR118W		1735	1788	0.97	-0.04	-0.10	-0.24
YBL013W		1925	1984	0.97	-0.04	-0.10	-0.24
YDL184C	RPL41A	3962	4083	0.97	-0.04	-0.10	-0.24
YAR068W		2813	2899	0.97	-0.04	-0.10	-0.24
YGR172C	YIP1	1338	1379	0.97	-0.04	-0.10	-0.24
YLR203C	MSS51	1723	1776	0.97	-0.04	-0.10	-0.24
YER105C	NUP157	5896	6077	0.97	-0.04	-0.10	-0.24
YDL098C	SNU23	3996	4119	0.97	-0.04	-0.10	-0.24
YDR160W	SSY1	1024	1056	0.97	-0.04	-0.10	-0.24
YJL116C	NCA3	570	588	0.97	-0.04	-0.10	-0.24
YER031C	YPT31	2534	2613	0.97	-0.04	-0.10	-0.24
YNL144C		1613	1663	0.97	-0.04	-0.10	-0.24
YLR006C	SSK1	1729	1783	0.97	-0.04	-0.10	-0.24
YKL094W	YJU3	1103	1138	0.97	-0.04	-0.10	-0.24
YPR097W		2988	3083	0.97	-0.04	-0.10	-0.24
YMR033W	ARP9	1336	1378	0.97	-0.05	-0.10	-0.24
YDR468C	TLG1	2189	2259	0.97	-0.05	-0.10	-0.24
YNL059C	ARP5	2669	2754	0.97	-0.05	-0.10	-0.25
YLR066W	SPC3	800	826	0.97	-0.05	-0.10	-0.25
YGL107C		4375	4516	0.97	-0.05	-0.10	-0.25
YLR113W	HOG1	2008	2073	0.97	-0.05	-0.10	-0.25
YEL077C		6134	6335	0.97	-0.05	-0.10	-0.25
YIR003W		2131	2201	0.97	-0.05	-0.10	-0.25
YBR185C	MBA1	5882	6076	0.97	-0.05	-0.10	-0.25
YDR342C	HXT7	3069	3171	0.97	-0.05	-0.10	-0.25

YFL010C		1717	1774	0.97	-0.05	-0.10	-0.25
YBR199W	KTR4	2211	2285	0.97	-0.05	-0.10	-0.25
YHR064C	PDR13	1084	1120	0.97	-0.05	-0.10	-0.25
YPR140W		1281	1324	0.97	-0.05	-0.10	-0.25
YMR294W	JNM1	495	512	0.97	-0.05	-0.10	-0.25
YBR224W		2640	2729	0.97	-0.05	-0.10	-0.25
YOR294W	RRS1	1428	1476	0.97	-0.05	-0.10	-0.25
YNL259C	ATX1	4955	5123	0.97	-0.05	-0.10	-0.25
YKR011C		475	491	0.97	-0.05	-0.10	-0.25
YDR424C	DYN2	5552	5743	0.97	-0.05	-0.10	-0.25
YLR175W	CBF5	6111	6324	0.97	-0.05	-0.11	-0.25
YBR163W	DEM1	6522	6750	0.97	-0.05	-0.11	-0.26
YDL124W		1577	1632	0.97	-0.05	-0.11	-0.26
YLL041C	SDH2	1346	1394	0.97	-0.05	-0.11	-0.26
YKL067W	YNK1	1064	1102	0.97	-0.05	-0.11	-0.26
YDL126C	CDC48	7300	7560	0.97	-0.05	-0.11	-0.26
YNL334C	SNO2	929	962	0.97	-0.05	-0.11	-0.26
YER086W	ILV1	3031	3140	0.97	-0.05	-0.11	-0.26
YGL179C		666	690	0.97	-0.05	-0.11	-0.26
YAL016W	TPD3	3470	3596	0.97	-0.05	-0.11	-0.26
YFL056C	AAD6	3021	3131	0.96	-0.05	-0.11	-0.26
YDR296W	MHR1	670	694	0.96	-0.05	-0.11	-0.26
YDR067C		4733	4906	0.96	-0.05	-0.11	-0.26
YLR050C		598	620	0.96	-0.05	-0.11	-0.26
YFL042C		1528	1584	0.96	-0.05	-0.11	-0.26
YLR026C	SED5	1076	1116	0.96	-0.05	-0.11	-0.26
YJL016W		389	403	0.96	-0.05	-0.11	-0.26
YLR225C		1161	1204	0.96	-0.05	-0.11	-0.26
YMR115W		805	835	0.96	-0.05	-0.11	-0.26
YDL112W	TRM3	6602	6846	0.96	-0.05	-0.11	-0.26
YGR262C		682	707	0.96	-0.05	-0.11	-0.26
YPR052C	NHP6A	1036	1075	0.96	-0.05	-0.11	-0.26
YCL026C		2096	2174	0.96	-0.05	-0.11	-0.26
YMR058W	FET3	3079	3194	0.96	-0.05	-0.11	-0.26
YDR451C		2988	3101	0.96	-0.05	-0.11	-0.26
YPL174C	NIP100	2657	2757	0.96	-0.05	-0.11	-0.26

YMR043W	MCM1	3363	3490	0.96	-0.05	-0.11	-0.27
YIL058W		3175	3296	0.96	-0.05	-0.11	-0.27
YBL062W		5627	5842	0.96	-0.05	-0.11	-0.27
YDR334W	SWR1	6954	7220	0.96	-0.05	-0.11	-0.27
YIRCdelta6	YIRCdelta6	897	932	0.96	-0.05	-0.11	-0.27
YDR276C		2630	2732	0.96	-0.05	-0.11	-0.27
YKR034W	DAL80	1905	1979	0.96	-0.06	-0.11	-0.27
YCL055W	KAR4	1920	1996	0.96	-0.06	-0.11	-0.27
YJL179W	PFD1	627	652	0.96	-0.06	-0.11	-0.27
YPL215W	CBP3	958	996	0.96	-0.06	-0.11	-0.27
YJL148W	RPA34	2037	2118	0.96	-0.06	-0.11	-0.27
YGL054C	ERV14	2470	2569	0.96	-0.06	-0.11	-0.27
YBR184W		1741	1811	0.96	-0.06	-0.11	-0.27
YOR119C	RIO1	2917	3034	0.96	-0.06	-0.11	-0.27
YML129C	COX14	957	995	0.96	-0.06	-0.11	-0.27
YAL042W	FUN9	2603	2708	0.96	-0.06	-0.11	-0.27
YLR176C	RFX1	630	655	0.96	-0.06	-0.11	-0.27
YPR075C	OPY2	1949	2028	0.96	-0.06	-0.11	-0.27
YPL203W	PKA3	1137	1183	0.96	-0.06	-0.11	-0.27
YFLCdelta6	YFLCdelta6	2209	2298	0.96	-0.06	-0.11	-0.27
YER155C	BEM2	2745	2857	0.96	-0.06	-0.11	-0.27
YLR355C	ILV5	5905	6147	0.96	-0.06	-0.11	-0.28
YJRWdelta20	YJRWdelta20	1297	1350	0.96	-0.06	-0.11	-0.28
YNL289W	PCL1	316	329	0.96	-0.06	-0.11	-0.28
YMR076C	PDS5	2745	2858	0.96	-0.06	-0.11	-0.28
YMR012W	CLU1	1937	2018	0.96	-0.06	-0.11	-0.28
YLR422W		5468	5696	0.96	-0.06	-0.12	-0.28
YMR002W		1600	1668	0.96	-0.06	-0.12	-0.28
YPR193C	HPA2	4102	4278	0.96	-0.06	-0.12	-0.28
YKL157W	APE2	5910	6164	0.96	-0.06	-0.12	-0.28
YOL035C		1344	1402	0.96	-0.06	-0.12	-0.28
YNL297C		6209	6477	0.96	-0.06	-0.12	-0.28
YGL115W	SNF4	2010	2097	0.96	-0.06	-0.12	-0.28
YCR029C		1159	1209	0.96	-0.06	-0.12	-0.28
YLL022C	HIF1	1011	1055	0.96	-0.06	-0.12	-0.28
YNL153C	GIM3	2594	2707	0.96	-0.06	-0.12	-0.28

YGL072C		1446	1509	0.96	-0.06	-0.12	-0.28
YIL130W		3702	3864	0.96	-0.06	-0.12	-0.29
YOR379C		1825	1905	0.96	-0.06	-0.12	-0.29
YCL012W		6876	7179	0.96	-0.06	-0.12	-0.29
YER167W	BCK2	6978	7286	0.96	-0.06	-0.12	-0.29
YOR215C		1814	1894	0.96	-0.06	-0.12	-0.29
YGL132W		815	851	0.96	-0.06	-0.12	-0.29
YGR025W		4338	4532	0.96	-0.06	-0.12	-0.29
YDL095W	PMT1	3140	3280	0.96	-0.06	-0.12	-0.29
YIL163C		804	840	0.96	-0.06	-0.12	-0.29
YBR291C	CTP1	1964	2052	0.96	-0.06	-0.12	-0.29
YIL044C		753	787	0.96	-0.06	-0.12	-0.29
YNL226W		867	906	0.96	-0.06	-0.12	-0.29
YCR089W	FIG2	3666	3832	0.96	-0.06	-0.12	-0.29
YER089C	PTC2	2400	2509	0.96	-0.06	-0.12	-0.29
YBL052C	SAS3	5196	5433	0.96	-0.06	-0.12	-0.29
YPL208W		1018	1065	0.96	-0.06	-0.12	-0.29
YNL315C	ATP11	1780	1862	0.96	-0.07	-0.12	-0.29
YGL127C	SOH1	3711	3883	0.96	-0.07	-0.12	-0.29
YBR093C	PHO5	481	503	0.96	-0.07	-0.12	-0.29
YNL313C		4971	5202	0.96	-0.07	-0.12	-0.29
YER157W	SEC34	3180	3328	0.96	-0.07	-0.12	-0.29
YFLWdelta1	YFLWdelta1	765	801	0.96	-0.07	-0.12	-0.29
YLR323C		2194	2297	0.96	-0.07	-0.12	-0.30
YJR128W		615	644	0.96	-0.07	-0.12	-0.30
YDR260C	SWM1	3807	3987	0.95	-0.07	-0.12	-0.30
YKR095W	MLP1	4881	5112	0.95	-0.07	-0.12	-0.30
YOR365C		379	397	0.95	-0.07	-0.12	-0.30
YER038C		1650	1729	0.95	-0.07	-0.12	-0.30
YDL013W	HEX3	2783	2916	0.95	-0.07	-0.12	-0.30
YOR209C	NPT1	3870	4056	0.95	-0.07	-0.12	-0.30
YBL063W	KIP1	5107	5354	0.95	-0.07	-0.12	-0.30
YMR172W	HOT1	3245	3402	0.95	-0.07	-0.12	-0.30
YGR256W	GND2	1836	1925	0.95	-0.07	-0.12	-0.30
YLR002C		2899	3040	0.95	-0.07	-0.12	-0.30
YFL013C		2705	2837	0.95	-0.07	-0.12	-0.30

YLL019C	KNS1	3312	3474	0.95	-0.07	-0.13	-0.30
YHR096C	HXT5	4067	4267	0.95	-0.07	-0.13	-0.30
YMR230W	RPS10B	923	968	0.95	-0.07	-0.13	-0.30
YGL197W	MDS3	4738	4971	0.95	-0.07	-0.13	-0.30
YHR204W		846	888	0.95	-0.07	-0.13	-0.30
YJL071W	ARG2	1885	1978	0.95	-0.07	-0.13	-0.30
YJL196C	ELO1	1715	1799	0.95	-0.07	-0.13	-0.30
YDR335W	MSN5	1911	2005	0.95	-0.07	-0.13	-0.30
YGL149W		1244	1305	0.95	-0.07	-0.13	-0.30
YDL223C		2915	3059	0.95	-0.07	-0.13	-0.30
YKR007W		375	394	0.95	-0.07	-0.13	-0.30
YDL206W		2068	2171	0.95	-0.07	-0.13	-0.30
YHRCdelta4	YHRCdelta4	487	511	0.95	-0.07	-0.13	-0.30
YDL207W	GLE1	2384	2503	0.95	-0.07	-0.13	-0.31
YIR024C	GIF1	963	1011	0.95	-0.07	-0.13	-0.31
YLR215C		815	856	0.95	-0.07	-0.13	-0.31
YDR454C	GUK1	2623	2755	0.95	-0.07	-0.13	-0.31
YLR207W	HRD3	2854	2998	0.95	-0.07	-0.13	-0.31
YMR103C		1184	1244	0.95	-0.07	-0.13	-0.31
YDR040C	ENA1	4451	4678	0.95	-0.07	-0.13	-0.31
YJL033W	HCA4	3757	3949	0.95	-0.07	-0.13	-0.31
YLR068W		1419	1492	0.95	-0.07	-0.13	-0.31
YDR404C	RPB7	5077	5338	0.95	-0.07	-0.13	-0.31
YJL088W	ARG3	1135	1193	0.95	-0.07	-0.13	-0.31
YML006C	GIS4	915	963	0.95	-0.07	-0.13	-0.31
YLR287C-A	RPS30A	6470	6806	0.95	-0.07	-0.13	-0.31
YKL121W		3579	3766	0.95	-0.07	-0.13	-0.31
YBR131W	CCZ1	3478	3659	0.95	-0.07	-0.13	-0.31
YBL038W	MRPL16	1833	1929	0.95	-0.07	-0.13	-0.31
YCRX19W		1821	1918	0.95	-0.07	-0.13	-0.32
YLR166C	SEC10	1706	1797	0.95	-0.07	-0.13	-0.32
YOR084W		567	597	0.95	-0.08	-0.13	-0.32
YGL047W		3852	4058	0.95	-0.08	-0.13	-0.32
YJR134C	SGM1	3336	3515	0.95	-0.08	-0.13	-0.32
YDR447C	RPS17B	3106	3273	0.95	-0.08	-0.13	-0.32
YDL188C	PPH22	1798	1895	0.95	-0.08	-0.13	-0.32

YER011W	TIR1	2845	2999	0.95	-0.08	-0.13	-0.32
YKL150W	MCR1	1434	1511	0.95	-0.08	-0.13	-0.32
YOL158C		1363	1437	0.95	-0.08	-0.13	-0.32
YDL232W	OST4	894	942	0.95	-0.08	-0.13	-0.32
YDR536W	STL1	1334	1407	0.95	-0.08	-0.13	-0.32
YGL006W	PMC1	2738	2887	0.95	-0.08	-0.13	-0.32
YML059C		3481	3671	0.95	-0.08	-0.13	-0.32
YER047C	SAP1	1541	1626	0.95	-0.08	-0.13	-0.32
YDR231C	COX20	1460	1541	0.95	-0.08	-0.13	-0.32
YLR088W	GAA1	2762	2915	0.95	-0.08	-0.13	-0.32
YOR351C	MEK1	1402	1480	0.95	-0.08	-0.13	-0.32
YLR465C		4340	4581	0.95	-0.08	-0.13	-0.32
YJL100W		844	891	0.95	-0.08	-0.13	-0.32
YNL215W		3367	3558	0.95	-0.08	-0.14	-0.33
YHR022C		1464	1547	0.95	-0.08	-0.14	-0.33
YCL054W	SPB1	10808	11424	0.95	-0.08	-0.14	-0.33
YEL021W	URA3	1544	1632	0.95	-0.08	-0.14	-0.33
YDL034W		4800	5075	0.95	-0.08	-0.14	-0.33
YGR073C		6359	6725	0.95	-0.08	-0.14	-0.33
YOL094C	RFC4	850	899	0.95	-0.08	-0.14	-0.33
YER095W	RAD51	3419	3617	0.95	-0.08	-0.14	-0.33
YDR294C	DPL1	1719	1819	0.95	-0.08	-0.14	-0.33
YLR160C	ASP3-4	1058	1120	0.94	-0.08	-0.14	-0.33
YCRX10W		6040	6392	0.94	-0.08	-0.14	-0.33
YJR117W	STE24	1982	2098	0.94	-0.08	-0.14	-0.33
YHR215W	PHO12	4725	5001	0.94	-0.08	-0.14	-0.33
YKL114C	APN1	713	755	0.94	-0.08	-0.14	-0.33
YPL119C	DBP1	631	668	0.94	-0.08	-0.14	-0.33
YOR087W		1115	1181	0.94	-0.08	-0.14	-0.34
YOR354C		1878	1989	0.94	-0.08	-0.14	-0.34
YDL204W		2118	2243	0.94	-0.08	-0.14	-0.34
YFL068W		6183	6549	0.94	-0.08	-0.14	-0.34
YDR463W	STP1	7333	7768	0.94	-0.08	-0.14	-0.34
YGR220C	MRPL9	3945	4180	0.94	-0.08	-0.14	-0.34
YNR036C		1987	2106	0.94	-0.08	-0.14	-0.34
YAL026C	DRS2	2884	3057	0.94	-0.08	-0.14	-0.34

YDR047W	HEM12	2175	2305	0.94	-0.08	-0.14	-0.34
YGL254W	FZF1	722	765	0.94	-0.08	-0.14	-0.34
YDL018C	ERP3	1055	1118	0.94	-0.08	-0.14	-0.34
YPR176C	BET2	1313	1392	0.94	-0.08	-0.14	-0.34
YGR191W	HIP1	4487	4759	0.94	-0.08	-0.14	-0.34
YIL117C		4185	4438	0.94	-0.08	-0.14	-0.34
YER002W		2682	2845	0.94	-0.09	-0.14	-0.34
YHR023W	MYO1	6657	7062	0.94	-0.09	-0.14	-0.34
YDR056C		3214	3409	0.94	-0.09	-0.14	-0.34
YPL194W	DDC1	2681	2844	0.94	-0.09	-0.14	-0.34
YBL073W		2204	2338	0.94	-0.09	-0.14	-0.34
YBL101W-B		2003	2125	0.94	-0.09	-0.14	-0.34
YGL039W		2048	2173	0.94	-0.09	-0.14	-0.34
YDL172C		2781	2952	0.94	-0.09	-0.14	-0.34
YHR196W		1669	1771	0.94	-0.09	-0.14	-0.34
YLR261C		976	1036	0.94	-0.09	-0.14	-0.34
YPL158C		3216	3414	0.94	-0.09	-0.14	-0.34
YCR079W		1911	2029	0.94	-0.09	-0.14	-0.34
YJL151C		521	553	0.94	-0.09	-0.14	-0.35
YNL281W	HCH1	2695	2863	0.94	-0.09	-0.14	-0.35
YDR069C	DOA4	1451	1541	0.94	-0.09	-0.14	-0.35
YLR201C		706	750	0.94	-0.09	-0.14	-0.35
YKR005C		1331	1414	0.94	-0.09	-0.14	-0.35
YPR089W		486	516	0.94	-0.09	-0.14	-0.35
YNL265C	IST1	2845	3024	0.94	-0.09	-0.14	-0.35
YER019W		1667	1772	0.94	-0.09	-0.14	-0.35
YGR265W		1311	1394	0.94	-0.09	-0.14	-0.35
YFR020W		1568	1667	0.94	-0.09	-0.14	-0.35
YNL251C	NRD1	3789	4029	0.94	-0.09	-0.14	-0.35
YER136W	GDI1	8419	8956	0.94	-0.09	-0.15	-0.35
YMR281W	GPI12	1856	1975	0.94	-0.09	-0.15	-0.35
YJR049C	UTR1	2555	2719	0.94	-0.09	-0.15	-0.35
YCR006C		2223	2366	0.94	-0.09	-0.15	-0.35
YLR082C	SRL2	1837	1955	0.94	-0.09	-0.15	-0.35
YHR115C		1734	1846	0.94	-0.09	-0.15	-0.35
YGL215W	CLG1	3043	3241	0.94	-0.09	-0.15	-0.36

YNL333W	SNZ2	501	534	0.94	-0.09	-0.15	-0.36
YBL048W		6082	6480	0.94	-0.09	-0.15	-0.36
YDR187C		3520	3751	0.94	-0.09	-0.15	-0.36
YPL234C	TFP3	1112	1185	0.94	-0.09	-0.15	-0.36
YOR153W	PDR5	7510	8007	0.94	-0.09	-0.15	-0.36
YLL066C		7730	8242	0.94	-0.09	-0.15	-0.36
YKL205W	LOS1	2373	2531	0.94	-0.09	-0.15	-0.36
YGL195W	GCN1	3758	4009	0.94	-0.09	-0.15	-0.36
YJL158C	CIS3	2707	2888	0.94	-0.09	-0.15	-0.36
YIL051C	MMD1	872	930	0.94	-0.09	-0.15	-0.36
YIR042C		2108	2250	0.94	-0.09	-0.15	-0.36
YPL218W	SAR1	4690	5006	0.94	-0.09	-0.15	-0.36
YKR035C		1010	1078	0.94	-0.09	-0.15	-0.36
YLR098C	CHA4	2963	3163	0.94	-0.09	-0.15	-0.36
YOR232W	MGE1	2399	2561	0.94	-0.09	-0.15	-0.36
YOR181W	LAS17	2489	2658	0.94	-0.09	-0.15	-0.36
YIR035C		970	1036	0.94	-0.10	-0.15	-0.37
YML016C	PPZ1	5088	5436	0.94	-0.10	-0.15	-0.37
YBR069C	VAP1	3616	3863	0.94	-0.10	-0.15	-0.37
YMR193W	MRPL24	346	370	0.94	-0.10	-0.15	-0.37
YCL003W		1849	1976	0.94	-0.10	-0.15	-0.37
YOR019W		1912	2044	0.94	-0.10	-0.15	-0.37
YMR095C	SNO1	1260	1348	0.94	-0.10	-0.15	-0.37
YLR079W	SIC1	3289	3518	0.93	-0.10	-0.15	-0.37
YBR255W		2537	2714	0.93	-0.10	-0.15	-0.37
YDL101C	DUN1	2393	2561	0.93	-0.10	-0.15	-0.37
YIL132C		1239	1326	0.93	-0.10	-0.15	-0.37
YCL034W		752	805	0.93	-0.10	-0.15	-0.37
YBR140C	IRA1	5882	6299	0.93	-0.10	-0.15	-0.37
YIL017C	YIL017C	3139	3361	0.93	-0.10	-0.15	-0.37
YHR100C		1092	1170	0.93	-0.10	-0.16	-0.38
YOL155C		3629	3888	0.93	-0.10	-0.16	-0.38
YNR056C	BIO5	2044	2190	0.93	-0.10	-0.16	-0.38
YOR228C		1091	1169	0.93	-0.10	-0.16	-0.38
YML003W		8550	9162	0.93	-0.10	-0.16	-0.38
YML056C		4788	5131	0.93	-0.10	-0.16	-0.38

YHR189W		756	810	0.93	-0.10	-0.16	-0.38
YDR507C	GIN4	3530	3784	0.93	-0.10	-0.16	-0.38
YIL158W		390	418	0.93	-0.10	-0.16	-0.38
YCRX01W		8987	9640	0.93	-0.10	-0.16	-0.38
YBR057C	MUM2	4260	4570	0.93	-0.10	-0.16	-0.38
YFR004W	RPN11	2198	2358	0.93	-0.10	-0.16	-0.38
YMR152W	YIM1	1064	1142	0.93	-0.10	-0.16	-0.38
YLR386W		1942	2085	0.93	-0.10	-0.16	-0.38
YILWTy3-1D	YILWTy3-1D	2975	3194	0.93	-0.10	-0.16	-0.38
YNL329C	PEX6	2256	2422	0.93	-0.10	-0.16	-0.38
YKL181W	PRS1	3697	3971	0.93	-0.10	-0.16	-0.38
YDR180W	SCC2	6740	7240	0.93	-0.10	-0.16	-0.39
YKL099C		4424	4753	0.93	-0.10	-0.16	-0.39
YDR198C		3151	3387	0.93	-0.10	-0.16	-0.39
YDL105W	QRI2	1585	1704	0.93	-0.10	-0.16	-0.39
YLR442C	SIR3	9043	9724	0.93	-0.10	-0.16	-0.39
YNL234W		1359	1461	0.93	-0.10	-0.16	-0.39
YML114C		781	840	0.93	-0.11	-0.16	-0.39
YML082W		2527	2718	0.93	-0.11	-0.16	-0.39
YAL065C-A		3083	3317	0.93	-0.11	-0.16	-0.39
YGL143C	MRF1	6139	6606	0.93	-0.11	-0.16	-0.39
YLR169W		1078	1160	0.93	-0.11	-0.16	-0.39
YOR146W		957	1030	0.93	-0.11	-0.16	-0.39
YJL132W		1000	1077	0.93	-0.11	-0.16	-0.39
YGL053W		2331	2510	0.93	-0.11	-0.16	-0.39
YAL002W	VPS8	2316	2495	0.93	-0.11	-0.16	-0.39
YBR222C	FAT2	3084	3322	0.93	-0.11	-0.16	-0.39
YJR066W	TOR1	1508	1624	0.93	-0.11	-0.16	-0.39
YMR209C		1259	1357	0.93	-0.11	-0.16	-0.40
YER005W	YND1	2212	2384	0.93	-0.11	-0.16	-0.40
YOL053C-A		711	766	0.93	-0.11	-0.16	-0.40
YJL115W	ASF1	2167	2336	0.93	-0.11	-0.16	-0.40
YLR452C	SST2	3842	4141	0.93	-0.11	-0.16	-0.40
YLR357W	RSC2	2888	3113	0.93	-0.11	-0.16	-0.40
YGL229C	SAP4	2641	2847	0.93	-0.11	-0.16	-0.40
YMR094W	CTF13	1121	1209	0.93	-0.11	-0.16	-0.40

YNL156C		1327	1431	0.93	-0.11	-0.16	-0.40
YCR057C	PWP2	5782	6235	0.93	-0.11	-0.16	-0.40
YCL074W		1699	1832	0.93	-0.11	-0.17	-0.40
YBL053W		2424	2615	0.93	-0.11	-0.17	-0.40
YDL025C		5910	6376	0.93	-0.11	-0.17	-0.40
YGR028W	MSP1	1035	1117	0.93	-0.11	-0.17	-0.40
YNL228W		498	537	0.93	-0.11	-0.17	-0.40
YMR169C	ALD3	1118	1206	0.93	-0.11	-0.17	-0.40
YCL005W		2590	2795	0.93	-0.11	-0.17	-0.40
YDR311W	TFB1	812	877	0.93	-0.11	-0.17	-0.40
YKR009C	FOX2	568	613	0.93	-0.11	-0.17	-0.40
YJL046W		1781	1923	0.93	-0.11	-0.17	-0.40
YMR120C	ADE17	1607	1735	0.93	-0.11	-0.17	-0.40
YML053C		532	574	0.93	-0.11	-0.17	-0.40
YFRWdelta7	YFRWdelta7	972	1050	0.93	-0.11	-0.17	-0.40
YGL201C	MCM6	3028	3271	0.93	-0.11	-0.17	-0.40
YBR015C	MNN2	1179	1274	0.93	-0.11	-0.17	-0.40
YNR031C	SSK2	865	934	0.93	-0.11	-0.17	-0.40
YLR131C	ACE2	2366	2557	0.93	-0.11	-0.17	-0.41
YBR094W		4053	4381	0.93	-0.11	-0.17	-0.41
YPL044C		5534	5983	0.92	-0.11	-0.17	-0.41
YNL323W		7068	7642	0.92	-0.11	-0.17	-0.41
YBR273C		2333	2523	0.92	-0.11	-0.17	-0.41
YIL175W		1198	1295	0.92	-0.11	-0.17	-0.41
YOR225W		2497	2701	0.92	-0.11	-0.17	-0.41
YLL054C		1156	1251	0.92	-0.11	-0.17	-0.41
YER169W	RPH1	2167	2345	0.92	-0.11	-0.17	-0.41
YEL066W	HPA3	1457	1576	0.92	-0.11	-0.17	-0.41
YKL191W	DPH2	1753	1897	0.92	-0.11	-0.17	-0.41
YOR244W	ESA1	660	714	0.92	-0.11	-0.17	-0.41
YCRX12W		1253	1356	0.92	-0.11	-0.17	-0.41
YHL038C	CBP2	3821	4135	0.92	-0.11	-0.17	-0.41
YDR112W		472	511	0.92	-0.11	-0.17	-0.41
YDR041W		6403	6929	0.92	-0.11	-0.17	-0.41
YLR069C	MEF1	2606	2820	0.92	-0.11	-0.17	-0.41
YERCdelta8	YERCdelta8	464	502	0.92	-0.11	-0.17	-0.41

YCRX21C		4345	4703	0.92	-0.11	-0.17	-0.41
YOR145C		2056	2226	0.92	-0.11	-0.17	-0.41
YGR083C	GCD2	4417	4783	0.92	-0.11	-0.17	-0.41
YDL205C	HEM3	2887	3126	0.92	-0.11	-0.17	-0.41
YDR307W		739	800	0.92	-0.12	-0.17	-0.41
YPR020W	ATP20	354	383	0.92	-0.12	-0.17	-0.41
YLR402W		2917	3160	0.92	-0.12	-0.17	-0.41
YGL148W	ARO2	2567	2781	0.92	-0.12	-0.17	-0.41
YHR219W		6424	6963	0.92	-0.12	-0.17	-0.42
YHR213W		1242	1347	0.92	-0.12	-0.17	-0.42
YBR135W	CKS1	3742	4059	0.92	-0.12	-0.17	-0.42
YLR388W	RPS29A	1235	1340	0.92	-0.12	-0.17	-0.42
YCR047C		5414	5876	0.92	-0.12	-0.17	-0.42
YMR046W-A	YMR046W-A	3082	3345	0.92	-0.12	-0.17	-0.42
YAL024C	LTE1	665	722	0.92	-0.12	-0.18	-0.42
YBL076C	ILS1	6563	7128	0.92	-0.12	-0.18	-0.42
YIL046W	MET30	2044	2220	0.92	-0.12	-0.18	-0.42
YCL004W	PGS1	780	847	0.92	-0.12	-0.18	-0.42
YLR025W	SNF7	649	705	0.92	-0.12	-0.18	-0.42
YGR218W	CRM1	3620	3933	0.92	-0.12	-0.18	-0.42
YDR502C	SAM2	3639	3953	0.92	-0.12	-0.18	-0.42
YIL004C	BET1	998	1084	0.92	-0.12	-0.18	-0.42
YGL261C		1446	1571	0.92	-0.12	-0.18	-0.43
YJL072C		1058	1150	0.92	-0.12	-0.18	-0.43
YFR011C		418	454	0.92	-0.12	-0.18	-0.43
YLL036C	PRP19	1372	1491	0.92	-0.12	-0.18	-0.43
YGL056C	SDS23	1724	1874	0.92	-0.12	-0.18	-0.43
YMR158W		3149	3423	0.92	-0.12	-0.18	-0.43
YCRX18C		5489	5966	0.92	-0.12	-0.18	-0.43
YBR293W		1771	1926	0.92	-0.12	-0.18	-0.43
YDL166C		1124	1222	0.92	-0.12	-0.18	-0.43
YOL111C		1360	1479	0.92	-0.12	-0.18	-0.43
YKL066W		917	997	0.92	-0.12	-0.18	-0.43
YDR184C	ATC1	3526	3836	0.92	-0.12	-0.18	-0.43
YMR016C	SOK2	2597	2827	0.92	-0.12	-0.18	-0.43
YEL019C	MMS21	2176	2369	0.92	-0.12	-0.18	-0.43

YPR126C		1814	1975	0.92	-0.12	-0.18	-0.43
YOR169C		2072	2256	0.92	-0.12	-0.18	-0.43
YBL024W	NCL1	3645	3969	0.92	-0.12	-0.18	-0.43
YCR102C		691	753	0.92	-0.12	-0.18	-0.43
YHR036W		2529	2755	0.92	-0.12	-0.18	-0.43
YPL106C	SSE1	6094	6638	0.92	-0.12	-0.18	-0.43
YEL038W	UTR4	5397	5879	0.92	-0.12	-0.18	-0.43
YAL029C	MYO4	1245	1356	0.92	-0.12	-0.18	-0.43
YLR229C	CDC42	766	835	0.92	-0.12	-0.18	-0.43
YER008C	SEC3	1570	1711	0.92	-0.12	-0.18	-0.43
YLR053C		1946	2120	0.92	-0.12	-0.18	-0.43
YIL031W	SMT4	1267	1380	0.92	-0.12	-0.18	-0.43
YPR111W	DBF20	2256	2458	0.92	-0.12	-0.18	-0.43
YJR024C		2649	2887	0.92	-0.12	-0.18	-0.43
YCR097WA		2080	2267	0.92	-0.12	-0.18	-0.44
YGR009C	SEC9	3310	3608	0.92	-0.12	-0.18	-0.44
YDR150W	NUM1	3498	3813	0.92	-0.12	-0.18	-0.44
YOR304W	ISW2	897	978	0.92	-0.12	-0.18	-0.44
YLR189C	UGT51	1079	1176	0.92	-0.12	-0.18	-0.44
YER160C		7891	8603	0.92	-0.12	-0.18	-0.44
YKL212W	SAC1	3763	4103	0.92	-0.12	-0.18	-0.44
YBL012C		5064	5521	0.92	-0.12	-0.18	-0.44
YBL004W		2207	2407	0.92	-0.12	-0.18	-0.44
YBR167C	POP7	2959	3227	0.92	-0.12	-0.18	-0.44
YDR060W		1073	1170	0.92	-0.13	-0.18	-0.44
YDRCTy1-2C	YDRCTy1-2C	17873	19498	0.92	-0.13	-0.18	-0.44
YDR315C	IPK1	2539	2770	0.92	-0.13	-0.18	-0.44
YJR126C		1769	1931	0.92	-0.13	-0.18	-0.44
YLR410W	VIP1	1189	1298	0.92	-0.13	-0.18	-0.44
YKL183W		564	616	0.92	-0.13	-0.18	-0.44
YLR024C		8161	8910	0.92	-0.13	-0.18	-0.44
YNL219C	ALG9	1632	1782	0.92	-0.13	-0.18	-0.44
YBR227C	MCX1	7734	8444	0.92	-0.13	-0.18	-0.44
YCR077C	PAT1	2698	2946	0.92	-0.13	-0.18	-0.44
YBL045C	COR1	2023	2210	0.92	-0.13	-0.18	-0.44
YCR062W		3314	3620	0.92	-0.13	-0.18	-0.44

YNL140C		1339	1463	0.92	-0.13	-0.18	-0.44
YGL036W	MTC2	1960	2142	0.92	-0.13	-0.18	-0.44
YOR200W		1390	1519	0.92	-0.13	-0.18	-0.44
YJR065C	ARP3	2958	3233	0.91	-0.13	-0.18	-0.45
YCL069W		3141	3433	0.91	-0.13	-0.18	-0.45
YER023W	PRO3	1078	1178	0.91	-0.13	-0.18	-0.45
YDL066W	IDP1	10848	11862	0.91	-0.13	-0.19	-0.45
YGL037C		2140	2340	0.91	-0.13	-0.19	-0.45
YCRX09C		3924	4293	0.91	-0.13	-0.19	-0.45
YJL029C		2912	3186	0.91	-0.13	-0.19	-0.45
YJL084C		1241	1358	0.91	-0.13	-0.19	-0.45
YOL025W	LAG2	7674	8400	0.91	-0.13	-0.19	-0.45
YLR223C	IFH1	8998	9850	0.91	-0.13	-0.19	-0.45
YHL005C		383	419	0.91	-0.13	-0.19	-0.45
YBR170C	NPL4	1897	2078	0.91	-0.13	-0.19	-0.45
YDL012C		3691	4042	0.91	-0.13	-0.19	-0.45
YJL130C	URA2	6972	7640	0.91	-0.13	-0.19	-0.45
YOR229W	WTM2	2010	2203	0.91	-0.13	-0.19	-0.45
YOR135C		1303	1428	0.91	-0.13	-0.19	-0.46
YDR105C		2100	2302	0.91	-0.13	-0.19	-0.46
YIL078W	THS1	8527	9350	0.91	-0.13	-0.19	-0.46
YDL231C		2169	2378	0.91	-0.13	-0.19	-0.46
YPL072W	UBP16	2027	2223	0.91	-0.13	-0.19	-0.46
YCRX03C		3105	3405	0.91	-0.13	-0.19	-0.46
YLR213C	CRR1	976	1071	0.91	-0.13	-0.19	-0.46
YER019C-A	SBH2	1185	1301	0.91	-0.13	-0.19	-0.46
YBR208C	DUR1,2	3490	3830	0.91	-0.13	-0.19	-0.46
YGL099W		3337	3663	0.91	-0.13	-0.19	-0.46
YGR251W		680	747	0.91	-0.13	-0.19	-0.46
YAL033W	POP5	823	904	0.91	-0.13	-0.19	-0.46
YCL075W		1356	1489	0.91	-0.13	-0.19	-0.46
YGR061C	ADE6	4818	5291	0.91	-0.14	-0.19	-0.46
YCR017C		9687	10638	0.91	-0.14	-0.19	-0.46
YNL320W		800	879	0.91	-0.14	-0.19	-0.46
YML128C		463	508	0.91	-0.14	-0.19	-0.46
YER090W	TRP2	2693	2958	0.91	-0.14	-0.19	-0.46

YLR413W		3118	3425	0.91	-0.14	-0.19	-0.46
YCL073C		6903	7583	0.91	-0.14	-0.19	-0.46
YER021W	RPN3	1712	1881	0.91	-0.14	-0.19	-0.46
YOR323C	PRO2	949	1043	0.91	-0.14	-0.19	-0.46
YDL102W	CDC2	828	910	0.91	-0.14	-0.19	-0.46
YCR081W	SRB8	4690	5153	0.91	-0.14	-0.19	-0.46
YGL249W	ZIP2	2180	2395	0.91	-0.14	-0.19	-0.46
YML111W	BUL2	1659	1823	0.91	-0.14	-0.19	-0.46
YLR272C	LOC7	7552	8300	0.91	-0.14	-0.19	-0.46
YML087C		1229	1351	0.91	-0.14	-0.19	-0.47
YML081W		4931	5420	0.91	-0.14	-0.19	-0.47
YGR252W	GCN5	4440	4882	0.91	-0.14	-0.19	-0.47
YIR031C	DAL7	1275	1402	0.91	-0.14	-0.19	-0.47
YNL155W		1424	1566	0.91	-0.14	-0.19	-0.47
YNL184C		2303	2533	0.91	-0.14	-0.19	-0.47
YHR054C		1069	1176	0.91	-0.14	-0.19	-0.47
YGL005C		4467	4914	0.91	-0.14	-0.19	-0.47
YCR076C		2963	3260	0.91	-0.14	-0.19	-0.47
YDR491C		929	1022	0.91	-0.14	-0.19	-0.47
YER006W		5616	6180	0.91	-0.14	-0.19	-0.47
YFL043C		586	645	0.91	-0.14	-0.19	-0.47
YOR363C	PIP2	728	801	0.91	-0.14	-0.19	-0.47
YNL276C		797	877	0.91	-0.14	-0.19	-0.47
YHR123W	EPT1	1692	1862	0.91	-0.14	-0.19	-0.47
YLR104W		1851	2038	0.91	-0.14	-0.19	-0.47
YBL020W	RFT1	6717	7396	0.91	-0.14	-0.20	-0.47
YOR005C	DNL4	1933	2128	0.91	-0.14	-0.20	-0.47
YOR384W	FRE5	2265	2495	0.91	-0.14	-0.20	-0.47
YCLX11W		1361	1499	0.91	-0.14	-0.20	-0.47
YAR070C		4702	5179	0.91	-0.14	-0.20	-0.47
YGL082W		1693	1865	0.91	-0.14	-0.20	-0.47
YLR008C		3836	4227	0.91	-0.14	-0.20	-0.47
YJR033C		3321	3660	0.91	-0.14	-0.20	-0.47
YDR530C	APA2	1101	1213	0.91	-0.14	-0.20	-0.47
YML051W	GAL80	2871	3164	0.91	-0.14	-0.20	-0.47
YAR007C	RFA1	1658	1827	0.91	-0.14	-0.20	-0.47

YOR184W	SER1	1400	1543	0.91	-0.14	-0.20	-0.48
YML075C	HMG1	2862	3157	0.91	-0.14	-0.20	-0.48
YDR475C		1609	1775	0.91	-0.14	-0.20	-0.48
YNL007C	SIS1	2222	2451	0.91	-0.14	-0.20	-0.48
YML049C	RSE1	332	366	0.91	-0.14	-0.20	-0.48
YIL127C		1156	1276	0.91	-0.14	-0.20	-0.48
YLR233C	EST1	806	890	0.91	-0.14	-0.20	-0.48
YOL121C	RPS19A	2407	2657	0.91	-0.14	-0.20	-0.48
YBR075W		3553	3922	0.91	-0.14	-0.20	-0.48
YGR204W	ADE3	4077	4501	0.91	-0.14	-0.20	-0.48
YHL036W	MUP3	1165	1286	0.91	-0.14	-0.20	-0.48
YOL048C		546	603	0.91	-0.14	-0.20	-0.48
YER033C		636	702	0.91	-0.14	-0.20	-0.48
YCRX16C		696	769	0.91	-0.14	-0.20	-0.48
YDR201W	SPC19	4018	4438	0.91	-0.14	-0.20	-0.48
YDR309C	GIC2	2723	3009	0.91	-0.14	-0.20	-0.48
YBR095C		699	772	0.90	-0.14	-0.20	-0.48
YBL054W		8838	9767	0.90	-0.14	-0.20	-0.48
YDL142C	CRD1	1511	1670	0.90	-0.14	-0.20	-0.48
YGR143W	SKN1	1610	1780	0.90	-0.14	-0.20	-0.48
YDL169C	UGX2	4422	4889	0.90	-0.14	-0.20	-0.49
YNL316C	PHA2	1719	1901	0.90	-0.14	-0.20	-0.49
YAR060C		1589	1757	0.90	-0.15	-0.20	-0.49
YIL150C	DNA43	1096	1212	0.90	-0.15	-0.20	-0.49
YLR448W	RPL6B	4093	4528	0.90	-0.15	-0.20	-0.49
YGR062C	COX18	763	844	0.90	-0.15	-0.20	-0.49
YNL317W	PFS2	4559	5044	0.90	-0.15	-0.20	-0.49
YDR431W		374	414	0.90	-0.15	-0.20	-0.49
YBR245C	ISW1	6229	6892	0.90	-0.15	-0.20	-0.49
YOL092W		1036	1147	0.90	-0.15	-0.20	-0.49
YIL061C	SNP1	3711	4107	0.90	-0.15	-0.20	-0.49
YIL112W		3969	4393	0.90	-0.15	-0.20	-0.49
YDL007W	RPT2	1936	2143	0.90	-0.15	-0.20	-0.49
YNL119W		5192	5747	0.90	-0.15	-0.20	-0.49
YPR143W		1107	1225	0.90	-0.15	-0.20	-0.49
YDL073W		3243	3590	0.90	-0.15	-0.20	-0.49

YER066W		1425	1578	0.90	-0.15	-0.20	-0.49
YPR133W-A	YPR133W-A	625	692	0.90	-0.15	-0.20	-0.49
YOR338W		664	735	0.90	-0.15	-0.20	-0.49
YNL044W	YIP3	1461	1619	0.90	-0.15	-0.20	-0.49
YKL075C		802	888	0.90	-0.15	-0.20	-0.49
YDL156W		2451	2715	0.90	-0.15	-0.20	-0.49
YOL027C		2596	2876	0.90	-0.15	-0.20	-0.49
YMR088C		2011	2229	0.90	-0.15	-0.20	-0.49
YDR421W		2379	2637	0.90	-0.15	-0.20	-0.49
YOR002W	ALG6	1721	1907	0.90	-0.15	-0.20	-0.49
YLR181C		2148	2381	0.90	-0.15	-0.20	-0.49
YOR148C	SPP2	2356	2612	0.90	-0.15	-0.20	-0.49
YLR389C	STE23	1493	1655	0.90	-0.15	-0.20	-0.49
YPL187W	MF(ALPHA)1	696	772	0.90	-0.15	-0.21	-0.50
YPL073C		1699	1884	0.90	-0.15	-0.21	-0.50
YGR098C	ESP1	2882	3196	0.90	-0.15	-0.21	-0.50
YJR149W		1040	1154	0.90	-0.15	-0.21	-0.50
YJL063C	MRPL8	557	618	0.90	-0.15	-0.21	-0.50
YMR034C		1592	1767	0.90	-0.15	-0.21	-0.50
YCR004C	YCP4	2476	2748	0.90	-0.15	-0.21	-0.50
YGL043W	DST1	5509	6115	0.90	-0.15	-0.21	-0.50
YGL186C		1872	2078	0.90	-0.15	-0.21	-0.50
YPL217C	BMS1	6868	7626	0.90	-0.15	-0.21	-0.50
YOL012C	HTA3	896	995	0.90	-0.15	-0.21	-0.50
YNL191W		713	792	0.90	-0.15	-0.21	-0.50
YBR225W		3457	3839	0.90	-0.15	-0.21	-0.50
YKRCdelta11	YKRCdelta11	3053	3390	0.90	-0.15	-0.21	-0.50
YOL135C	MED7	2075	2305	0.90	-0.15	-0.21	-0.50
YMR164C	MSS11	3107	3451	0.90	-0.15	-0.21	-0.50
YNR024W		1534	1704	0.90	-0.15	-0.21	-0.50
YDR206W	EBS1	4981	5533	0.90	-0.15	-0.21	-0.50
YFL009W	CDC4	2264	2515	0.90	-0.15	-0.21	-0.50
YLL018C-A	COX19	2850	3166	0.90	-0.15	-0.21	-0.50
YNL077W		937	1041	0.90	-0.15	-0.21	-0.50
YGL066W		1590	1766	0.90	-0.15	-0.21	-0.50
YNR057C	BIO4	368	409	0.90	-0.15	-0.21	-0.50

YHR122W		3266	3629	0.90	-0.15	-0.21	-0.50
YPR007C	SPO69	322	358	0.90	-0.15	-0.21	-0.50
YIR014W		819	910	0.90	-0.15	-0.21	-0.50
YCRX17W		4345	4830	0.90	-0.15	-0.21	-0.50
YDL208W	NHP2	1039	1155	0.90	-0.15	-0.21	-0.50
YGR269W		846	941	0.90	-0.15	-0.21	-0.50
YPL138C		1315	1462	0.90	-0.15	-0.21	-0.50
YDR302W		2055	2285	0.90	-0.15	-0.21	-0.51
YIL080W		3130	3481	0.90	-0.15	-0.21	-0.51
YJR005W	APL1	1561	1736	0.90	-0.15	-0.21	-0.51
YER060w-A	FCY22	2560	2848	0.90	-0.15	-0.21	-0.51
YDR197W	CBS2	1883	2095	0.90	-0.15	-0.21	-0.51
YGR186W	TFG1	3141	3495	0.90	-0.15	-0.21	-0.51
YNR018W		687	764	0.90	-0.15	-0.21	-0.51
YAL064W		2856	3179	0.90	-0.15	-0.21	-0.51
YBR074W		2615	2911	0.90	-0.15	-0.21	-0.51
YJL036W	SNX4	4259	4740	0.90	-0.15	-0.21	-0.51
YDR262W		3168	3526	0.90	-0.15	-0.21	-0.51
YIL053W	RHR2	3610	4018	0.90	-0.15	-0.21	-0.51
YJL221C	FSP2	518	577	0.90	-0.15	-0.21	-0.51
YCR049C		2341	2606	0.90	-0.15	-0.21	-0.51
YBL058W	SHP1	1445	1609	0.90	-0.16	-0.21	-0.51
YKL055C	OAR1	725	808	0.90	-0.16	-0.21	-0.51
YKR055W	RHO4	337	375	0.90	-0.16	-0.21	-0.51
YML073C	RPL6A	1748	1948	0.90	-0.16	-0.21	-0.51
YGL227W	TIN1	1230	1371	0.90	-0.16	-0.21	-0.51
YDR345C	HXT3	2058	2294	0.90	-0.16	-0.21	-0.51
YDR238C	SEC26	6508	7254	0.90	-0.16	-0.21	-0.51
YIL059C		1161	1295	0.90	-0.16	-0.21	-0.52
YNR042W		1251	1395	0.90	-0.16	-0.21	-0.52
YGR072W	UPF3	4284	4778	0.90	-0.16	-0.21	-0.52
YLR171W		1537	1715	0.90	-0.16	-0.21	-0.52
YIR040C		1185	1324	0.90	-0.16	-0.22	-0.52
YBR230C		2199	2457	0.89	-0.16	-0.22	-0.52
YDR070C		2318	2590	0.89	-0.16	-0.22	-0.52
YFL-TYA		14217	15889	0.89	-0.16	-0.22	-0.52

YJL104W		1981	2215	0.89	-0.16	-0.22	-0.52
YNL266W		1529	1710	0.89	-0.16	-0.22	-0.52
YNL247W		4658	5212	0.89	-0.16	-0.22	-0.53
YLR031W		2568	2874	0.89	-0.16	-0.22	-0.53
YOLCdelta2	YOLCdelta2	2791	3124	0.89	-0.16	-0.22	-0.53
YIL023C		940	1052	0.89	-0.16	-0.22	-0.53
YAL012W	CYS3	1099	1231	0.89	-0.16	-0.22	-0.53
YJL150W		2090	2340	0.89	-0.16	-0.22	-0.53
YPR115W		739	827	0.89	-0.16	-0.22	-0.53
YCR066W	RAD18	2413	2702	0.89	-0.16	-0.22	-0.53
YJL022W		1930	2163	0.89	-0.16	-0.22	-0.53
YDL122W	UBP1	4360	4887	0.89	-0.16	-0.22	-0.53
YER137C		1499	1681	0.89	-0.17	-0.22	-0.53
YJR142W		781	876	0.89	-0.17	-0.22	-0.54
YOR189W		926	1039	0.89	-0.17	-0.22	-0.54
YDL078C	MDH3	3100	3478	0.89	-0.17	-0.22	-0.54
YDR264C	AKR1	1371	1538	0.89	-0.17	-0.22	-0.54
YKR081C		2613	2932	0.89	-0.17	-0.22	-0.54
YGL202W	ARO8	2785	3126	0.89	-0.17	-0.22	-0.54
YBR209W		2044	2295	0.89	-0.17	-0.22	-0.54
YBR026C	MRF1'	5949	6680	0.89	-0.17	-0.22	-0.54
YCL017C	NFS1	2982	3349	0.89	-0.17	-0.22	-0.54
YOR293W	RPS10A	1059	1190	0.89	-0.17	-0.22	-0.54
YMR064W	AEP1	1590	1786	0.89	-0.17	-0.22	-0.54
YDR366C		2745	3084	0.89	-0.17	-0.22	-0.54
YJL042W	MHP1	1545	1736	0.89	-0.17	-0.22	-0.54
YPL110C		3227	3626	0.89	-0.17	-0.22	-0.54
YGR042W		426	479	0.89	-0.17	-0.22	-0.54
YGL114W		1701	1912	0.89	-0.17	-0.22	-0.54
YKR028W	SAP190	1109	1247	0.89	-0.17	-0.23	-0.54
YOR004W		3350	3767	0.89	-0.17	-0.23	-0.54
YIL082W-A		6060	6814	0.89	-0.17	-0.23	-0.54
YHR071W	PCL5	3955	4449	0.89	-0.17	-0.23	-0.55
YLR411W	CTR3	722	812	0.89	-0.17	-0.23	-0.55
YPL251W		1537	1729	0.89	-0.17	-0.23	-0.55
YDR123C	INO2	2510	2825	0.89	-0.17	-0.23	-0.55

YGR017W		2287	2575	0.89	-0.17	-0.23	-0.55
YGL069C		1190	1340	0.89	-0.17	-0.23	-0.55
YGR056W	RSC1	1369	1542	0.89	-0.17	-0.23	-0.55
YOR165W		3448	3884	0.89	-0.17	-0.23	-0.55
YNL169C	PSD1	712	802	0.89	-0.17	-0.23	-0.55
YGR079W		2209	2488	0.89	-0.17	-0.23	-0.55
YHR110W	ERP5	4886	5504	0.89	-0.17	-0.23	-0.55
YAL054C	ACS1	2390	2693	0.89	-0.17	-0.23	-0.55
YLR340W	RPP0	2533	2854	0.89	-0.17	-0.23	-0.55
YLR141W	RRN5	1419	1599	0.89	-0.17	-0.23	-0.55
YOL013C	HRD1	1378	1553	0.89	-0.17	-0.23	-0.55
YMR178W		3568	4022	0.89	-0.17	-0.23	-0.55
YGR006W	PRP18	557	628	0.89	-0.17	-0.23	-0.55
YOR273C		580	654	0.89	-0.17	-0.23	-0.55
YNL249C	MPA43	2154	2429	0.89	-0.17	-0.23	-0.55
YJL066C		1269	1431	0.89	-0.17	-0.23	-0.55
YFR030W	MET10	1166	1315	0.89	-0.17	-0.23	-0.55
YGR030C	POP6	1831	2065	0.89	-0.17	-0.23	-0.55
YDR074W	TPS2	2360	2662	0.89	-0.17	-0.23	-0.56
YBR073W	RDH54	2881	3251	0.89	-0.17	-0.23	-0.56
YJR054W		1603	1810	0.89	-0.18	-0.23	-0.56
YOL063C		1546	1746	0.89	-0.18	-0.23	-0.56
YIL014W	MNT3	1506	1701	0.89	-0.18	-0.23	-0.56
YBL085W	BOI1	1137	1284	0.89	-0.18	-0.23	-0.56
YJL091C		2929	3308	0.89	-0.18	-0.23	-0.56
YMR173W-A		1580	1785	0.89	-0.18	-0.23	-0.56
YOL041C		2446	2763	0.89	-0.18	-0.23	-0.56
YDR492W		1491	1684	0.89	-0.18	-0.23	-0.56
YDR402C	DIT2	464	524	0.89	-0.18	-0.23	-0.56
YGL258W		855	966	0.88	-0.18	-0.23	-0.56
YDL190C	UFD2	6270	7087	0.88	-0.18	-0.23	-0.56
YDL060W		2104	2379	0.88	-0.18	-0.23	-0.56
YJR120W		1011	1143	0.88	-0.18	-0.23	-0.56
YOR292C		568	642	0.88	-0.18	-0.23	-0.56
YGR156W		2061	2330	0.88	-0.18	-0.23	-0.56
YML086C	ALO1	1748	1977	0.88	-0.18	-0.23	-0.56

YPR046W	MCM16	1319	1491	0.88	-0.18	-0.23	-0.56
YDR449C		1194	1350	0.88	-0.18	-0.23	-0.56
YDR390C	UBA2	1474	1667	0.88	-0.18	-0.23	-0.57
YMR018W		642	726	0.88	-0.18	-0.23	-0.57
YLR092W	SUL2	1466	1659	0.88	-0.18	-0.23	-0.57
YDR349C	YPS7	1300	1472	0.88	-0.18	-0.23	-0.57
YGR242W		1195	1353	0.88	-0.18	-0.24	-0.57
YERWdelta21	YERWdelta21	8436	9553	0.88	-0.18	-0.24	-0.57
YMR077C		1129	1278	0.88	-0.18	-0.24	-0.57
YEL001C		3005	3406	0.88	-0.18	-0.24	-0.57
YPL056C		2012	2281	0.88	-0.18	-0.24	-0.57
YCR105W		4089	4636	0.88	-0.18	-0.24	-0.57
YLR095C		3981	4515	0.88	-0.18	-0.24	-0.57
YAR074C		5078	5760	0.88	-0.18	-0.24	-0.57
YFR045W		920	1044	0.88	-0.18	-0.24	-0.58
YGR249W	MGA1	955	1084	0.88	-0.18	-0.24	-0.58
YOR141C	ARP8	3934	4465	0.88	-0.18	-0.24	-0.58
YMR293C		527	598	0.88	-0.18	-0.24	-0.58
YDR148C	KGD2	1547	1757	0.88	-0.18	-0.24	-0.58
YHR171W	APG7	1137	1291	0.88	-0.18	-0.24	-0.58
YLR371W	ROM2	2654	3014	0.88	-0.18	-0.24	-0.58
YPLCdelta10	YPLCdelta10	436	495	0.88	-0.18	-0.24	-0.58
YGL063W	PUS2	1856	2108	0.88	-0.18	-0.24	-0.58
YGL032C	AGA2	384	436	0.88	-0.18	-0.24	-0.58
YBR246W		3249	3691	0.88	-0.18	-0.24	-0.58
YDR251W	PAM1	1905	2164	0.88	-0.18	-0.24	-0.58
YAL028W		3195	3631	0.88	-0.18	-0.24	-0.58
YGR228W		2074	2357	0.88	-0.18	-0.24	-0.58
YML085C	TUB1	2286	2599	0.88	-0.19	-0.24	-0.58
YIR009W	MSL1	752	855	0.88	-0.19	-0.24	-0.58
YDR254W	CHL4	2916	3316	0.88	-0.19	-0.24	-0.58
YKR045C		2370	2695	0.88	-0.19	-0.24	-0.58
YPR040W		809	920	0.88	-0.19	-0.24	-0.58
YNL076W	MKS1	5742	6531	0.88	-0.19	-0.24	-0.58
YJL201W	ECM25	1707	1943	0.88	-0.19	-0.24	-0.59
YML112W	CTK3	803	914	0.88	-0.19	-0.24	-0.59

YOR103C	OST2	1122	1278	0.88	-0.19	-0.24	-0.59
YIL126W	STH1	3579	4077	0.88	-0.19	-0.24	-0.59
YBR218C	PYC2	2819	3211	0.88	-0.19	-0.24	-0.59
YPL155C	KIP2	1219	1389	0.88	-0.19	-0.24	-0.59
YMR194W	RPL36A	2572	2930	0.88	-0.19	-0.24	-0.59
YNL108C		2115	2410	0.88	-0.19	-0.24	-0.59
YEL060C	PRB1	2769	3155	0.88	-0.19	-0.24	-0.59
YOR023C	AHC1	925	1054	0.88	-0.19	-0.24	-0.59
YJL214W	HXT8	1188	1354	0.88	-0.19	-0.25	-0.59
YBR243C	ALG7	4163	4748	0.88	-0.19	-0.25	-0.59
YIL030C	SSM4	6235	7111	0.88	-0.19	-0.25	-0.59
YKL158W		591	674	0.88	-0.19	-0.25	-0.59
YGR109C	CLB6	1534	1750	0.88	-0.19	-0.25	-0.59
YJL128C	PBS2	1057	1206	0.88	-0.19	-0.25	-0.59
YLR454W		6741	7692	0.88	-0.19	-0.25	-0.60
YDR222W		4452	5081	0.88	-0.19	-0.25	-0.60
YPR071W		415	474	0.88	-0.19	-0.25	-0.60
YNL039W	TFC5	704	803	0.88	-0.19	-0.25	-0.60
YBR287W		1007	1150	0.88	-0.19	-0.25	-0.60
YDR087C	RRP1	2468	2817	0.88	-0.19	-0.25	-0.60
YFL057C		5486	6263	0.88	-0.19	-0.25	-0.60
YGR232W		751	857	0.88	-0.19	-0.25	-0.60
YBR237W	PRP5	5538	6323	0.88	-0.19	-0.25	-0.60
YBR144C		1756	2005	0.88	-0.19	-0.25	-0.60
YOLCdelta9	YOLCdelta9	1448	1654	0.88	-0.19	-0.25	-0.60
YBR042C		10389	11867	0.88	-0.19	-0.25	-0.60
YIL015C-A		2682	3065	0.88	-0.19	-0.25	-0.60
YBL034C	STU1	2083	2381	0.87	-0.19	-0.25	-0.60
YMR112C	MED11	3290	3762	0.87	-0.19	-0.25	-0.60
YDR235W	PRP42	1454	1663	0.87	-0.19	-0.25	-0.60
YNR072W	HXT17	1067	1220	0.87	-0.19	-0.25	-0.60
YLR016C		1740	1990	0.87	-0.19	-0.25	-0.60
YOR030W	DFG16	4176	4777	0.87	-0.19	-0.25	-0.60
YCL030C	HIS4	1716	1963	0.87	-0.19	-0.25	-0.60
YJL020C		5356	6128	0.87	-0.19	-0.25	-0.60
YGL231C		3329	3809	0.87	-0.19	-0.25	-0.61

YBR084C-A	RPL19A	2308	2641	0.87	-0.19	-0.25	-0.61
YJL114W		1082	1239	0.87	-0.19	-0.25	-0.61
YBL044W		3852	4409	0.87	-0.19	-0.25	-0.61
YGL088W		1047	1199	0.87	-0.20	-0.25	-0.61
YDR479C		3088	3535	0.87	-0.20	-0.25	-0.61
YDR535C		1194	1367	0.87	-0.20	-0.25	-0.61
YGRWdelta19	YGRWdelta19	1911	2188	0.87	-0.20	-0.25	-0.61
YNR007C	AUT1	3603	4127	0.87	-0.20	-0.25	-0.61
YIR036C		1333	1527	0.87	-0.20	-0.25	-0.61
YDR024W		3775	4325	0.87	-0.20	-0.25	-0.61
YILCdelta2	YILCdelta2	5345	6123	0.87	-0.20	-0.25	-0.61
YCR020W-B	YCR020W-B	2295	2629	0.87	-0.20	-0.25	-0.61
YPR062W	FCY1	2572	2947	0.87	-0.20	-0.25	-0.61
YDR481C	PHO8	1465	1679	0.87	-0.20	-0.25	-0.61
YKL208W	CBT1	2842	3257	0.87	-0.20	-0.25	-0.61
YDL002C	NHP10	2404	2755	0.87	-0.20	-0.25	-0.61
YMR174C	PAI3	3866	4433	0.87	-0.20	-0.25	-0.61
YBR202W	CDC47	3032	3477	0.87	-0.20	-0.25	-0.61
YDR139C	RUB1	1533	1758	0.87	-0.20	-0.25	-0.61
YMR175W	SIP18	6511	7467	0.87	-0.20	-0.25	-0.61
YHR102W	NRK1	1781	2043	0.87	-0.20	-0.25	-0.61
YLR224W		1728	1982	0.87	-0.20	-0.25	-0.61
YBR124W		3199	3670	0.87	-0.20	-0.25	-0.61
YER184C		3812	4373	0.87	-0.20	-0.25	-0.61
YPR013C		1521	1745	0.87	-0.20	-0.25	-0.61
YLR219W		2146	2462	0.87	-0.20	-0.25	-0.61
YLR185W	RPL37A	575	660	0.87	-0.20	-0.25	-0.61
YDR172W	SUP35	1893	2172	0.87	-0.20	-0.25	-0.62
YCR086W		1125	1291	0.87	-0.20	-0.25	-0.62
YGL021W	ALK1	8544	9806	0.87	-0.20	-0.25	-0.62
YER187W		2603	2988	0.87	-0.20	-0.25	-0.62
YDR526C		9669	11099	0.87	-0.20	-0.26	-0.62
YIL062C	ARC15	2039	2341	0.87	-0.20	-0.26	-0.62
YGR141W		1499	1721	0.87	-0.20	-0.26	-0.62
YML028W	TSA1	2631	3021	0.87	-0.20	-0.26	-0.62
YCR087W		1374	1578	0.87	-0.20	-0.26	-0.62

YNL285W		1706	1959	0.87	-0.20	-0.26	-0.62
YOL109W	ZEO1	1207	1387	0.87	-0.20	-0.26	-0.62
YBL101W-A		8311	9550	0.87	-0.20	-0.26	-0.62
YGR127W		2454	2821	0.87	-0.20	-0.26	-0.62
YJL028W		683	785	0.87	-0.20	-0.26	-0.62
YDR291W		633	728	0.87	-0.20	-0.26	-0.62
YHR214C-B		3959	4552	0.87	-0.20	-0.26	-0.62
YLL021W	SPA2	5020	5772	0.87	-0.20	-0.26	-0.62
YDL200C	MGT1	747	859	0.87	-0.20	-0.26	-0.62
YGR068C		608	699	0.87	-0.20	-0.26	-0.62
YIL068C	SEC6	3079	3541	0.87	-0.20	-0.26	-0.62
YIR029W	DAL2	873	1004	0.87	-0.20	-0.26	-0.62
YIR007W		716	823	0.87	-0.20	-0.26	-0.62
YOR026W	BUB3	2508	2884	0.87	-0.20	-0.26	-0.62
YKL115C		2187	2515	0.87	-0.20	-0.26	-0.62
YDR512C		789	908	0.87	-0.20	-0.26	-0.62
YMR225C	MRPL44	2395	2755	0.87	-0.20	-0.26	-0.62
YIL160C	POT1	1574	1811	0.87	-0.20	-0.26	-0.62
YNL042W	BOP3	5337	6141	0.87	-0.20	-0.26	-0.62
YBLWTy2-1B	YBLWTy2-1B	10957	12608	0.87	-0.20	-0.26	-0.62
YJR053W	BFA1	917	1055	0.87	-0.20	-0.26	-0.63
YDL177C		1820	2095	0.87	-0.20	-0.26	-0.63
YDR440W	DOT1	3010	3466	0.87	-0.20	-0.26	-0.63
YMR074C		906	1043	0.87	-0.20	-0.26	-0.63
YER096W	SHC1	668	770	0.87	-0.20	-0.26	-0.63
YDR439W	LRS4	4250	4897	0.87	-0.20	-0.26	-0.63
YBR169C	SSE2	3465	3993	0.87	-0.20	-0.26	-0.63
YHR052W		3122	3597	0.87	-0.20	-0.26	-0.63
YBL039C	URA7	4053	4670	0.87	-0.20	-0.26	-0.63
YKR064W		2135	2460	0.87	-0.20	-0.26	-0.63
YGLWdelta10	YGLWdelta10	2149	2477	0.87	-0.20	-0.26	-0.63
YML090W		621	716	0.87	-0.20	-0.26	-0.63
YGR263C		1248	1439	0.87	-0.21	-0.26	-0.63
YEL018W		1533	1767	0.87	-0.21	-0.26	-0.63
YHRWdelta9	YHRWdelta9	3643	4202	0.87	-0.21	-0.26	-0.63
YDL193W		2657	3064	0.87	-0.21	-0.26	-0.63

YLR100W		2750	3172	0.87	-0.21	-0.26	-0.63
YFR047C		2917	3366	0.87	-0.21	-0.26	-0.63
YML097C	VPS9	2205	2545	0.87	-0.21	-0.26	-0.64
YML117W		5603	6467	0.87	-0.21	-0.26	-0.64
YLR387C		2213	2555	0.87	-0.21	-0.26	-0.64
YBR179C	FZO1	8643	9978	0.87	-0.21	-0.26	-0.64
YGL250W		1235	1426	0.87	-0.21	-0.26	-0.64
YPL176C		1265	1461	0.87	-0.21	-0.26	-0.64
YJL008C	CCT8	2642	3051	0.87	-0.21	-0.26	-0.64
YLR129W	DIP2	2343	2706	0.87	-0.21	-0.26	-0.64
YKR080W	MTD1	1516	1751	0.87	-0.21	-0.26	-0.64
YIL129C	TAO3	1184	1368	0.87	-0.21	-0.26	-0.64
YOR024W		1742	2013	0.87	-0.21	-0.26	-0.64
YGL067W		4194	4846	0.87	-0.21	-0.26	-0.64
YBR120C	CBP6	1572	1816	0.87	-0.21	-0.26	-0.64
YPR092W		1167	1349	0.87	-0.21	-0.26	-0.64
YBR155W	CNS1	1564	1808	0.87	-0.21	-0.26	-0.64
YPL172C	COX10	1587	1835	0.87	-0.21	-0.27	-0.64
YBR288C	APM3	539	623	0.86	-0.21	-0.27	-0.64
YOR088W		4495	5197	0.86	-0.21	-0.27	-0.64
YML077W	BET5	2049	2369	0.86	-0.21	-0.27	-0.64
YMR020W	FMS1	3209	3711	0.86	-0.21	-0.27	-0.64
YDR517W	GRH1	3042	3518	0.86	-0.21	-0.27	-0.64
YGR289C	MAL11	2020	2336	0.86	-0.21	-0.27	-0.64
YIL115C	NUP159	2258	2612	0.86	-0.21	-0.27	-0.64
YBL011W	SCT1	3034	3510	0.86	-0.21	-0.27	-0.64
YGR207C		1121	1297	0.86	-0.21	-0.27	-0.64
YDR472W	TRS31	1623	1877	0.86	-0.21	-0.27	-0.64
YEL053C	MAK10	1758	2035	0.86	-0.21	-0.27	-0.65
YDL046W		1781	2062	0.86	-0.21	-0.27	-0.65
YLR133W	CKI1	4146	4800	0.86	-0.21	-0.27	-0.65
YMR215W		771	893	0.86	-0.21	-0.27	-0.65
YOR029W		2000	2316	0.86	-0.21	-0.27	-0.65
YJL119C		958	1109	0.86	-0.21	-0.27	-0.65
YDR356W	NUF1	5667	6563	0.86	-0.21	-0.27	-0.65
YBR212W	NGR1	6828	7908	0.86	-0.21	-0.27	-0.65

YOL056W	GPM3	2067	2394	0.86	-0.21	-0.27	-0.65
YOR343C		572	662	0.86	-0.21	-0.27	-0.65
YOR212W	STE4	3709	4296	0.86	-0.21	-0.27	-0.65
YJR119C		4142	4799	0.86	-0.21	-0.27	-0.65
YBL107C		1079	1250	0.86	-0.21	-0.27	-0.65
YLR320W		1256	1455	0.86	-0.21	-0.27	-0.65
YDR469W		4698	5444	0.86	-0.21	-0.27	-0.65
YHR094C	HXT1	3901	4521	0.86	-0.21	-0.27	-0.65
YBL001C	ECM15	2269	2631	0.86	-0.21	-0.27	-0.65
YCR050C		2217	2571	0.86	-0.21	-0.27	-0.65
YLR012C		3438	3987	0.86	-0.21	-0.27	-0.65
YGR047C	TFC4	3844	4460	0.86	-0.21	-0.27	-0.65
YGL093W	SPC105	1940	2251	0.86	-0.21	-0.27	-0.65
YER016W	BIM1	2370	2752	0.86	-0.22	-0.27	-0.66
YPL093W	NOG1	1564	1817	0.86	-0.22	-0.27	-0.66
YGR271W		1332	1547	0.86	-0.22	-0.27	-0.66
YBR247C	ENP1	1304	1515	0.86	-0.22	-0.27	-0.66
YJR129C		2792	3245	0.86	-0.22	-0.27	-0.66
YDR230W		1320	1534	0.86	-0.22	-0.27	-0.66
YMR108W	ILV2	3360	3906	0.86	-0.22	-0.27	-0.66
YEL052W	AFG1	4096	4762	0.86	-0.22	-0.27	-0.66
YBR259W		2427	2821	0.86	-0.22	-0.27	-0.66
YLR464W		2613	3038	0.86	-0.22	-0.27	-0.66
YPR099C		618	718	0.86	-0.22	-0.27	-0.66
YLR256W	HAP1	5959	6930	0.86	-0.22	-0.27	-0.66
YBR100W		1339	1558	0.86	-0.22	-0.27	-0.66
YMR029C		1280	1489	0.86	-0.22	-0.27	-0.66
YKR033C		1016	1182	0.86	-0.22	-0.27	-0.66
YDL110C		2295	2670	0.86	-0.22	-0.27	-0.66
YBR146W	MRPS9	2262	2632	0.86	-0.22	-0.27	-0.66
YHRCdelta11	YHRCdelta11	689	802	0.86	-0.22	-0.28	-0.66
YBR128C	APG14	812	946	0.86	-0.22	-0.28	-0.67
YLR255C		2809	3271	0.86	-0.22	-0.28	-0.67
YPL268W	PLC1	1715	1997	0.86	-0.22	-0.28	-0.67
YDR299W	BFR2	1522	1773	0.86	-0.22	-0.28	-0.67
YLR063W		2841	3310	0.86	-0.22	-0.28	-0.67

YIL021W	RPB3	3982	4641	0.86	-0.22	-0.28	-0.67
YDR083W		2506	2921	0.86	-0.22	-0.28	-0.67
YMR147W		2904	3384	0.86	-0.22	-0.28	-0.67
YHR050W	SMF2	865	1008	0.86	-0.22	-0.28	-0.67
YNL331C	AAD14	2095	2443	0.86	-0.22	-0.28	-0.67
YNL200C		1930	2251	0.86	-0.22	-0.28	-0.67
YBR088C	POL30	8334	9720	0.86	-0.22	-0.28	-0.67
YKL052C		1820	2123	0.86	-0.22	-0.28	-0.67
YKR096W		2344	2735	0.86	-0.22	-0.28	-0.67
YPR077C		1452	1694	0.86	-0.22	-0.28	-0.67
YGL041C		2707	3160	0.86	-0.22	-0.28	-0.67
YMR242C	RPL20A	1061	1238	0.86	-0.22	-0.28	-0.67
YAL059W	ECM1	1480	1727	0.86	-0.22	-0.28	-0.67
YMR176W	ECM5	984	1149	0.86	-0.22	-0.28	-0.67
YEL054C	RPL12A	5102	5956	0.86	-0.22	-0.28	-0.67
YLR322W		811	947	0.86	-0.22	-0.28	-0.67
YOR168W	GLN4	3675	4290	0.86	-0.22	-0.28	-0.67
YJL001W	PRE3	1465	1711	0.86	-0.22	-0.28	-0.68
YOR054C		1337	1562	0.86	-0.22	-0.28	-0.68
YGR274C	TAF145	4337	5069	0.86	-0.22	-0.28	-0.68
YPR104C	FHL1	4251	4969	0.86	-0.23	-0.28	-0.68
YNL045W		3111	3637	0.86	-0.23	-0.28	-0.68
YHL020C	OPI1	1791	2094	0.86	-0.23	-0.28	-0.68
YOR104W		2655	3105	0.86	-0.23	-0.28	-0.68
YLR058C	SHM2	929	1086	0.86	-0.23	-0.28	-0.68
YPLCTy4-1C	YPLCTy4-1C	835	977	0.86	-0.23	-0.28	-0.68
YBR234C	ARC40	2480	2901	0.85	-0.23	-0.28	-0.68
YDR071C		2471	2892	0.85	-0.23	-0.28	-0.68
YLR117C	SYF3	4661	5454	0.85	-0.23	-0.28	-0.68
YJL184W		1315	1539	0.85	-0.23	-0.28	-0.68
YNL016W	PUB1	877	1026	0.85	-0.23	-0.28	-0.68
YEL049W	PAU2	2170	2540	0.85	-0.23	-0.28	-0.68
YKL082C		3147	3684	0.85	-0.23	-0.28	-0.68
YBR130C	SHE3	2511	2940	0.85	-0.23	-0.28	-0.68
YLR363C	NMD4	878	1028	0.85	-0.23	-0.28	-0.69
YDR195W	REF2	1746	2045	0.85	-0.23	-0.28	-0.69

YNR004W		2183	2557	0.85	-0.23	-0.28	-0.69
YBL071C		1343	1573	0.85	-0.23	-0.28	-0.69
YHR065C	RRP3	2792	3271	0.85	-0.23	-0.28	-0.69
YDL170W	UGA3	1131	1325	0.85	-0.23	-0.28	-0.69
YOL067C	RTG1	969	1135	0.85	-0.23	-0.28	-0.69
YPL022W	RAD1	1871	2192	0.85	-0.23	-0.28	-0.69
YMR013C	SEC59	2090	2449	0.85	-0.23	-0.28	-0.69
YLR065C		1271	1490	0.85	-0.23	-0.29	-0.69
YNL227C		638	748	0.85	-0.23	-0.29	-0.69
YFL012W		2876	3371	0.85	-0.23	-0.29	-0.69
YGL263W	COS12	398	467	0.85	-0.23	-0.29	-0.69
YFR046C		8457	9915	0.85	-0.23	-0.29	-0.69
YJR021C	REC107	2031	2381	0.85	-0.23	-0.29	-0.69
YMR141C		1240	1454	0.85	-0.23	-0.29	-0.69
YIL101C	XBP1	798	936	0.85	-0.23	-0.29	-0.69
YDL181W	INH1	869	1020	0.85	-0.23	-0.29	-0.69
YOL001W	PHO80	786	922	0.85	-0.23	-0.29	-0.69
YHLWdelta2	YHLWdelta2	989	1161	0.85	-0.23	-0.29	-0.69
YMR075W		2516	2953	0.85	-0.23	-0.29	-0.69
YMR059W	SEN15	883	1036	0.85	-0.23	-0.29	-0.69
YDR476C		911	1069	0.85	-0.23	-0.29	-0.69
YMR136W	GAT2	1106	1298	0.85	-0.23	-0.29	-0.69
YGL101W		442	519	0.85	-0.23	-0.29	-0.70
YKR078W		2262	2658	0.85	-0.23	-0.29	-0.70
YDR514C		2207	2594	0.85	-0.23	-0.29	-0.70
YJL047C	RTT101	5306	6236	0.85	-0.23	-0.29	-0.70
YDL140C	RPO21	4047	4756	0.85	-0.23	-0.29	-0.70
YPR130C		2351	2764	0.85	-0.23	-0.29	-0.70
YBR089W		1336	1570	0.85	-0.23	-0.29	-0.70
YEL039C	CYC7	618	726	0.85	-0.23	-0.29	-0.70
YLR143W		1879	2210	0.85	-0.23	-0.29	-0.70
YOR017W	PET127	1590	1870	0.85	-0.23	-0.29	-0.70
YCL009C	ILV6	1483	1745	0.85	-0.23	-0.29	-0.70
YDL067C	COX9	1166	1372	0.85	-0.23	-0.29	-0.70
YDR511W		4738	5575	0.85	-0.23	-0.29	-0.70
YDR136C		2085	2454	0.85	-0.24	-0.29	-0.70

YFR017C		763	898	0.85	-0.24	-0.29	-0.70
YCL020W		17680	20820	0.85	-0.24	-0.29	-0.71
YMR153W	NUP53	3166	3729	0.85	-0.24	-0.29	-0.71
YNL327W	EGT2	7012	8258	0.85	-0.24	-0.29	-0.71
YKL201C	MNN4	942	1110	0.85	-0.24	-0.29	-0.71
YNL038W		3046	3590	0.85	-0.24	-0.29	-0.71
YGR069W		2848	3356	0.85	-0.24	-0.29	-0.71
YLR017W	MEU1	2816	3319	0.85	-0.24	-0.29	-0.71
YOR306C		1729	2038	0.85	-0.24	-0.29	-0.71
YDL057W		2171	2559	0.85	-0.24	-0.29	-0.71
YGL119W	ABC1	1328	1566	0.85	-0.24	-0.29	-0.71
YDR409W		2661	3138	0.85	-0.24	-0.29	-0.71
YLL040C	VPS13	1369	1615	0.85	-0.24	-0.29	-0.71
YBR180W		2686	3168	0.85	-0.24	-0.29	-0.71
YLR307W	CDA1	2178	2570	0.85	-0.24	-0.29	-0.71
YJL089W	SIP4	1259	1486	0.85	-0.24	-0.29	-0.71
YDR378C	LSM6	1217	1436	0.85	-0.24	-0.29	-0.71
YDL189W		2391	2822	0.85	-0.24	-0.29	-0.71
YIR032C	DAL3	1956	2308	0.85	-0.24	-0.30	-0.71
YGR206W		1879	2218	0.85	-0.24	-0.30	-0.71
YBL095W		1419	1675	0.85	-0.24	-0.30	-0.71
YDR330W		804	949	0.85	-0.24	-0.30	-0.71
YGR140W	CBF2	3113	3675	0.85	-0.24	-0.30	-0.71
YDR445C		386	456	0.85	-0.24	-0.30	-0.71
YNL212W		1221	1441	0.85	-0.24	-0.30	-0.71
YNL223W	AUT2	1888	2229	0.85	-0.24	-0.30	-0.71
YPR051W	MAK3	907	1071	0.85	-0.24	-0.30	-0.71
YBR116C		1152	1360	0.85	-0.24	-0.30	-0.72
YMR317W		670	791	0.85	-0.24	-0.30	-0.72
YCR064C		2537	2997	0.85	-0.24	-0.30	-0.72
YBL040C	ERD2	3326	3930	0.85	-0.24	-0.30	-0.72
YER035W		464	548	0.85	-0.24	-0.30	-0.72
YLL009C	COX17	618	730	0.85	-0.24	-0.30	-0.72
YFL014W	HSP12	1349	1595	0.85	-0.24	-0.30	-0.72
YMR153C-A		2210	2613	0.85	-0.24	-0.30	-0.72
YOL134C		392	464	0.85	-0.24	-0.30	-0.72

YPL104W	MSD1	1303	1541	0.85	-0.24	-0.30	-0.72
YDL103C	QRI1	653	772	0.85	-0.24	-0.30	-0.72
YGR053C		1240	1467	0.85	-0.24	-0.30	-0.72
YHR095W		1998	2364	0.85	-0.24	-0.30	-0.72
YJR003C		1290	1526	0.85	-0.24	-0.30	-0.72
YPL125W		1883	2229	0.84	-0.24	-0.30	-0.72
YDR003W		483	572	0.84	-0.24	-0.30	-0.72
YLR036C		1770	2096	0.84	-0.24	-0.30	-0.72
YHR138C		2814	3332	0.84	-0.24	-0.30	-0.72
YBL108W		1298	1537	0.84	-0.24	-0.30	-0.72
YFL018W-A		3486	4128	0.84	-0.24	-0.30	-0.72
YDR267C		2746	3252	0.84	-0.24	-0.30	-0.72
YOR013W		578	684	0.84	-0.24	-0.30	-0.72
YDR153C		2971	3520	0.84	-0.24	-0.30	-0.73
YDR459C		501	594	0.84	-0.24	-0.30	-0.73
YPL283C	YRF1-7	3882	4600	0.84	-0.24	-0.30	-0.73
YKL175W		2385	2827	0.84	-0.25	-0.30	-0.73
YNR054C		1258	1491	0.84	-0.25	-0.30	-0.73
YPL136W		2129	2524	0.84	-0.25	-0.30	-0.73
YGL124C		1997	2368	0.84	-0.25	-0.30	-0.73
YLR420W	URA4	1486	1762	0.84	-0.25	-0.30	-0.73
YGR064W		1033	1225	0.84	-0.25	-0.30	-0.73
YLL037W		2682	3181	0.84	-0.25	-0.30	-0.73
YHR117W	TOM71	3700	4389	0.84	-0.25	-0.30	-0.73
YMR303C	ADH2	2436	2890	0.84	-0.25	-0.30	-0.73
YPL092W	SSU1	1949	2313	0.84	-0.25	-0.30	-0.73
YDR344C		604	717	0.84	-0.25	-0.30	-0.73
YKLCdelta6	YKLCdelta6	2293	2722	0.84	-0.25	-0.30	-0.73
YCR073C	SSK22	9353	11101	0.84	-0.25	-0.30	-0.73
YLR457C	NBP1	532	631	0.84	-0.25	-0.30	-0.73
YOL113W	SKM1	573	680	0.84	-0.25	-0.30	-0.73
YGL094C	PAN2	666	791	0.84	-0.25	-0.30	-0.74
YJR083C		3750	4455	0.84	-0.25	-0.30	-0.74
YHL008C		406	482	0.84	-0.25	-0.30	-0.74
YCL051W	LRE1	438	521	0.84	-0.25	-0.31	-0.74
YDR308C	SRB7	2693	3201	0.84	-0.25	-0.31	-0.74

YOL110W	SHR5	2086	2480	0.84	-0.25	-0.31	-0.74
YDL010W		2068	2459	0.84	-0.25	-0.31	-0.74
YDL113C		1563	1859	0.84	-0.25	-0.31	-0.74
YCL037C	SRO9	3808	4530	0.84	-0.25	-0.31	-0.74
YJR109C	CPA2	2339	2783	0.84	-0.25	-0.31	-0.74
YDR039C	ENA2	810	964	0.84	-0.25	-0.31	-0.74
YJR136C		659	784	0.84	-0.25	-0.31	-0.74
YDR518W	EUG1	4174	4968	0.84	-0.25	-0.31	-0.74
YDR167W	TAF25	1207	1437	0.84	-0.25	-0.31	-0.74
YPL142C		5844	6956	0.84	-0.25	-0.31	-0.74
YNR043W	MVD1	3116	3709	0.84	-0.25	-0.31	-0.74
YBR285W		3829	4559	0.84	-0.25	-0.31	-0.74
YDR216W	ADR1	4403	5243	0.84	-0.25	-0.31	-0.74
YKL053W		1773	2112	0.84	-0.25	-0.31	-0.75
YDL096C		4929	5875	0.84	-0.25	-0.31	-0.75
YPR204W		2525	3009	0.84	-0.25	-0.31	-0.75
YDR448W	ADA2	1185	1413	0.84	-0.25	-0.31	-0.75
YER188W		1619	1930	0.84	-0.25	-0.31	-0.75
YLR022C		1373	1637	0.84	-0.25	-0.31	-0.75
YHRCTy1-1A	YHRCTy1-1A	10264	12239	0.84	-0.25	-0.31	-0.75
YCR060W		4117	4909	0.84	-0.25	-0.31	-0.75
YCRX06W		2408	2873	0.84	-0.25	-0.31	-0.75
YNL185C	MRPL19	2278	2717	0.84	-0.25	-0.31	-0.75
YBR070C		820	978	0.84	-0.25	-0.31	-0.75
YMR047C	NUP116	3760	4489	0.84	-0.26	-0.31	-0.75
YKL062W	MSN4	1233	1472	0.84	-0.26	-0.31	-0.75
YPL007C		1808	2159	0.84	-0.26	-0.31	-0.75
YDL045C	FAD1	1894	2262	0.84	-0.26	-0.31	-0.75
YIL020C	HIS6	2865	3423	0.84	-0.26	-0.31	-0.76
YML067C		526	628	0.84	-0.26	-0.31	-0.76
YBR298C	MAL31	2210	2641	0.84	-0.26	-0.31	-0.76
YAL019W	FUN30	5462	6528	0.84	-0.26	-0.31	-0.76
YMR181C		2486	2971	0.84	-0.26	-0.31	-0.76
YDR515W	SLF1	1882	2250	0.84	-0.26	-0.31	-0.76
YDR103W	STE5	2951	3529	0.84	-0.26	-0.31	-0.76
YJL051W		1607	1922	0.84	-0.26	-0.31	-0.76

YBR134W		4134	4945	0.84	-0.26	-0.31	-0.76
YLR198C		3676	4398	0.84	-0.26	-0.31	-0.76
YGR032W	GSC2	4415	5287	0.84	-0.26	-0.32	-0.76
YPL253C	VIK1	3451	4133	0.83	-0.26	-0.32	-0.76
YIL099W	SGA1	492	589	0.83	-0.26	-0.32	-0.77
YMR026C	PEX12	320	383	0.83	-0.26	-0.32	-0.77
YNL071W	LAT1	2261	2709	0.83	-0.26	-0.32	-0.77
YCRX15W		812	974	0.83	-0.26	-0.32	-0.77
YER166W		601	720	0.83	-0.26	-0.32	-0.77
YBR147W		6914	8289	0.83	-0.26	-0.32	-0.77
YHR178W	STB5	7230	8670	0.83	-0.26	-0.32	-0.77
YOL154W		2453	2942	0.83	-0.26	-0.32	-0.77
YGR014W	MSB2	3647	4374	0.83	-0.26	-0.32	-0.77
YAR035W	YAT1	3502	4201	0.83	-0.26	-0.32	-0.77
YFL051C		1139	1367	0.83	-0.26	-0.32	-0.77
YDR176W	NGG1	1690	2028	0.83	-0.26	-0.32	-0.77
YGL125W	MET13	1642	1970	0.83	-0.26	-0.32	-0.77
YDL081C	RPP1A	1206	1447	0.83	-0.26	-0.32	-0.77
YPL171C	OYE3	582	699	0.83	-0.26	-0.32	-0.77
YDL130W	RPP1B	1947	2337	0.83	-0.26	-0.32	-0.77
YGR144W	THI4	530	637	0.83	-0.26	-0.32	-0.77
YHR145C		961	1155	0.83	-0.26	-0.32	-0.77
YGR203W		495	595	0.83	-0.26	-0.32	-0.77
YML100W-A		929	1116	0.83	-0.27	-0.32	-0.78
YEL065W	SIT1	1740	2091	0.83	-0.27	-0.32	-0.78
YJL082W	IML2	1629	1958	0.83	-0.27	-0.32	-0.78
YCR008W	SAT4	1394	1676	0.83	-0.27	-0.32	-0.78
YKL084W		4229	5087	0.83	-0.27	-0.32	-0.78
YGL121C		889	1069	0.83	-0.27	-0.32	-0.78
YHR173C		952	1145	0.83	-0.27	-0.32	-0.78
YDL158C		4693	5647	0.83	-0.27	-0.32	-0.78
YMR135W-A		1267	1524	0.83	-0.27	-0.32	-0.78
YNL137C	NAM9	773	930	0.83	-0.27	-0.32	-0.78
YDR010C		648	780	0.83	-0.27	-0.32	-0.78
YDL017W	CDC7	2684	3234	0.83	-0.27	-0.32	-0.78
YDL044C	MTF2	1609	1939	0.83	-0.27	-0.33	-0.79

YDL028C	MPS1	1993	2402	0.83	-0.27	-0.33	-0.79
YGL117W		1570	1893	0.83	-0.27	-0.33	-0.79
YBR114W	RAD16	2450	2953	0.83	-0.27	-0.33	-0.79
YMR109W	MYO5	2180	2630	0.83	-0.27	-0.33	-0.79
YDR462W	MRPL28	3139	3788	0.83	-0.27	-0.33	-0.79
YGL144C		3910	4719	0.83	-0.27	-0.33	-0.79
YBR226C		826	997	0.83	-0.27	-0.33	-0.79
YHR129C	ARP1	1866	2253	0.83	-0.27	-0.33	-0.79
YDR329C	PEX3	5868	7087	0.83	-0.27	-0.33	-0.79
YDR355C		2267	2738	0.83	-0.27	-0.33	-0.79
YBL041W	PRE7	1883	2275	0.83	-0.27	-0.33	-0.79
YIL048W	NEO1	3211	3881	0.83	-0.27	-0.33	-0.80
YDL197C	ASF2	1076	1301	0.83	-0.27	-0.33	-0.80
YOR217W	RFC1	4291	5187	0.83	-0.27	-0.33	-0.80
YDR523C	SPS1	3116	3766	0.83	-0.27	-0.33	-0.80
YBL008W	HIR1	6621	8005	0.83	-0.27	-0.33	-0.80
YJL168C	SET2	2562	3099	0.83	-0.27	-0.33	-0.80
YFL013W-A		1373	1661	0.83	-0.27	-0.33	-0.80
YCLCdelta1	YCLCdelta1	1550	1875	0.83	-0.27	-0.33	-0.80
YBL057C		1245	1506	0.83	-0.27	-0.33	-0.80
YDL239C		1005	1216	0.83	-0.27	-0.33	-0.80
YPL083C	SEN54	1129	1366	0.83	-0.28	-0.33	-0.80
YPR088C	SRP54	2853	3454	0.83	-0.28	-0.33	-0.80
YGL217C		1700	2059	0.83	-0.28	-0.33	-0.80
YMR161W	HLJ1	4793	5806	0.83	-0.28	-0.33	-0.80
YJLWdelta9	YJLWdelta9	3973	4813	0.83	-0.28	-0.33	-0.80
YOR291W		557	675	0.83	-0.28	-0.33	-0.81
YNL183C	NPR1	3947	4784	0.83	-0.28	-0.33	-0.81
YPL184C		2447	2966	0.83	-0.28	-0.33	-0.81
YKL210W	UBA1	1028	1246	0.83	-0.28	-0.33	-0.81
YDR084C		2750	3333	0.83	-0.28	-0.33	-0.81
YGR112W	SHY1	1403	1701	0.83	-0.28	-0.33	-0.81
YOL058W	ARG1	898	1089	0.83	-0.28	-0.33	-0.81
YPR175W	DPB2	1685	2044	0.82	-0.28	-0.33	-0.81
YML040W		16197	19645	0.82	-0.28	-0.33	-0.81
YLR292C	SEC72	903	1096	0.82	-0.28	-0.34	-0.81

YIL149C		2810	3410	0.82	-0.28	-0.34	-0.81
YDR100W		2149	2608	0.82	-0.28	-0.34	-0.81
YDR318W	MCM21	1499	1819	0.82	-0.28	-0.34	-0.81
YGR258C	RAD2	2291	2780	0.82	-0.28	-0.34	-0.81
YJR118C	ILM1	1940	2354	0.82	-0.28	-0.34	-0.81
YDR121W	DPB4	1675	2033	0.82	-0.28	-0.34	-0.81
YPL213W	LEA1	407	494	0.82	-0.28	-0.34	-0.81
YMR101C	SRT1	3152	3828	0.82	-0.28	-0.34	-0.81
YGR158C	MTR3	1739	2112	0.82	-0.28	-0.34	-0.81
YCR074C		936	1137	0.82	-0.28	-0.34	-0.81
YNL135C	FPR1	2177	2645	0.82	-0.28	-0.34	-0.81
YML093W		1863	2264	0.82	-0.28	-0.34	-0.81
YKL105C		3037	3690	0.82	-0.28	-0.34	-0.81
YLR083C	EMP70	4760	5785	0.82	-0.28	-0.34	-0.82
YDR268W	MSW1	2629	3195	0.82	-0.28	-0.34	-0.82
YBR115C	LYS2	3055	3714	0.82	-0.28	-0.34	-0.82
YOL093W		748	910	0.82	-0.28	-0.34	-0.82
YLL006W	MMM1	2152	2617	0.82	-0.28	-0.34	-0.82
YMR140W		1360	1654	0.82	-0.28	-0.34	-0.82
YPL008W	CHL1	1567	1906	0.82	-0.28	-0.34	-0.82
YOL087C		2155	2622	0.82	-0.28	-0.34	-0.82
YIL043C	CBR1	2636	3207	0.82	-0.28	-0.34	-0.82
YCRX11W		678	825	0.82	-0.28	-0.34	-0.82
YPL178W	MUD13	2813	3423	0.82	-0.28	-0.34	-0.82
YPR038W		440	535	0.82	-0.28	-0.34	-0.82
YKL083W		1066	1298	0.82	-0.28	-0.34	-0.82
YDR437W		3740	4558	0.82	-0.29	-0.34	-0.82
YNL208W		1145	1396	0.82	-0.29	-0.34	-0.82
YBR086C	IST2	5486	6687	0.82	-0.29	-0.34	-0.83
YDR332W		1724	2102	0.82	-0.29	-0.34	-0.83
YGR259C		3079	3755	0.82	-0.29	-0.34	-0.83
YLL017W		937	1143	0.82	-0.29	-0.34	-0.83
YBR058C	UBP14	10701	13052	0.82	-0.29	-0.34	-0.83
YIR038C	GTT1	970	1183	0.82	-0.29	-0.34	-0.83
YIR030C	DCG1	1023	1248	0.82	-0.29	-0.34	-0.83
YLR271W		1919	2341	0.82	-0.29	-0.34	-0.83

YGR188C	BUB1	3409	4158	0.82	-0.29	-0.34	-0.83
YNR037C		823	1005	0.82	-0.29	-0.34	-0.83
YDR257C	RMS1	1722	2102	0.82	-0.29	-0.34	-0.83
YCR054C	CTR86	1537	1876	0.82	-0.29	-0.34	-0.83
YHR051W	COX6	1257	1535	0.82	-0.29	-0.34	-0.83
YER119C-A		2414	2949	0.82	-0.29	-0.34	-0.83
YMR132C		1941	2371	0.82	-0.29	-0.34	-0.83
YBLWdelta10	YBLWdelta10	6523	7969	0.82	-0.29	-0.35	-0.83
YGR137W		2201	2690	0.82	-0.29	-0.35	-0.83
YDR509W		1136	1388	0.82	-0.29	-0.35	-0.83
YCL032W	STE50	3831	4686	0.82	-0.29	-0.35	-0.84
YGL085W		2360	2887	0.82	-0.29	-0.35	-0.84
YCRX08W		2319	2837	0.82	-0.29	-0.35	-0.84
YGR236C		1460	1787	0.82	-0.29	-0.35	-0.84
YOR055W		533	652	0.82	-0.29	-0.35	-0.84
YLR275W	SMD2	507	621	0.82	-0.29	-0.35	-0.84
YHR108W		748	916	0.82	-0.29	-0.35	-0.84
YMR279C		2229	2729	0.82	-0.29	-0.35	-0.84
YARCdelta3	YARCdelta3	700	857	0.82	-0.29	-0.35	-0.84
YBL029W		737	903	0.82	-0.29	-0.35	-0.84
YBR101C		4181	5121	0.82	-0.29	-0.35	-0.84
YCL035C	GRX1	1288	1578	0.82	-0.29	-0.35	-0.84
YPL282C		2769	3392	0.82	-0.29	-0.35	-0.84
YML107C		1834	2248	0.82	-0.29	-0.35	-0.84
YOR336W	KRE5	2244	2750	0.82	-0.29	-0.35	-0.84
YDR287W		763	935	0.82	-0.29	-0.35	-0.84
YDL183C		1270	1557	0.82	-0.29	-0.35	-0.84
YGR241C	YAP1802	1045	1281	0.82	-0.29	-0.35	-0.85
YHR132C	ECM14	1964	2408	0.82	-0.29	-0.35	-0.85
YDR114C		630	772	0.82	-0.29	-0.35	-0.85
YFR005C	SAD1	2676	3281	0.82	-0.29	-0.35	-0.85
YER053C		475	582	0.82	-0.29	-0.35	-0.85
YPL169C	MEX67	3989	4891	0.82	-0.29	-0.35	-0.85
YLR102C	APC9	1172	1438	0.82	-0.29	-0.35	-0.85
YPR068C	HOS1	344	422	0.81	-0.30	-0.35	-0.85
YFR016C		4648	5705	0.81	-0.30	-0.35	-0.85

YGL071W	RCS1	3094	3798	0.81	-0.30	-0.35	-0.85
YGR130C		7776	9547	0.81	-0.30	-0.35	-0.85
YHR169W	DBP8	888	1090	0.81	-0.30	-0.35	-0.85
YGL089C	MF(ALPHA)2	1096	1346	0.81	-0.30	-0.35	-0.85
YPL161C	BEM4	477	586	0.81	-0.30	-0.35	-0.85
YML010W-B		1438	1767	0.81	-0.30	-0.35	-0.85
YGL243W	TAD1	624	766	0.81	-0.30	-0.35	-0.85
YOL164W		1587	1950	0.81	-0.30	-0.35	-0.85
YGL196W		1066	1309	0.81	-0.30	-0.35	-0.85
YKRWdelta10	YKRWdelta10	603	741	0.81	-0.30	-0.35	-0.85
YML066C		2313	2842	0.81	-0.30	-0.35	-0.85
YGR247W		620	762	0.81	-0.30	-0.35	-0.85
YOR216C	RUD3	2575	3168	0.81	-0.30	-0.35	-0.86
YML103C	NUP188	576	709	0.81	-0.30	-0.35	-0.86
YBR051W		346	426	0.81	-0.30	-0.35	-0.86
YMR151W	YIM2	1769	2177	0.81	-0.30	-0.36	-0.86
YMR105C	PGM2	1802	2218	0.81	-0.30	-0.36	-0.86
YCR063W		1388	1709	0.81	-0.30	-0.36	-0.86
YDR149C		1740	2143	0.81	-0.30	-0.36	-0.86
YKL182W	FAS1	1200	1478	0.81	-0.30	-0.36	-0.86
YJL057C	IKS1	2370	2920	0.81	-0.30	-0.36	-0.86
YDR486C		4423	5452	0.81	-0.30	-0.36	-0.86
YLR155C	ASP3-1	2783	3430	0.81	-0.30	-0.36	-0.86
YLR173W		659	812	0.81	-0.30	-0.36	-0.86
YMR031W-A		2232	2752	0.81	-0.30	-0.36	-0.87
YNL010W		1468	1811	0.81	-0.30	-0.36	-0.87
YLR114C		2215	2734	0.81	-0.30	-0.36	-0.87
YNL339C	YRF1-6	1556	1921	0.81	-0.30	-0.36	-0.87
YEL005C	VAB31	2901	3582	0.81	-0.30	-0.36	-0.87
YCR033W		5521	6820	0.81	-0.30	-0.36	-0.87
YPL060W		579	715	0.81	-0.30	-0.36	-0.87
YDR495C	VPS3	11096	13712	0.81	-0.31	-0.36	-0.87
YKR032W		829	1025	0.81	-0.31	-0.36	-0.87
YNL006W	LST8	3992	4934	0.81	-0.31	-0.36	-0.87
YBR194W		1078	1332	0.81	-0.31	-0.36	-0.87
YNL105W		946	1169	0.81	-0.31	-0.36	-0.87

YGR011W		1064	1317	0.81	-0.31	-0.36	-0.88
YPL012W		1793	2219	0.81	-0.31	-0.36	-0.88
YML032C-A		898	1112	0.81	-0.31	-0.36	-0.88
YPL098C		2812	3480	0.81	-0.31	-0.36	-0.88
YFR049W	YMR31	1425	1764	0.81	-0.31	-0.36	-0.88
YDRWdelta23	YDRWdelta23	3803	4708	0.81	-0.31	-0.36	-0.88
YOL132W		2223	2752	0.81	-0.31	-0.36	-0.88
YLL025W		1505	1864	0.81	-0.31	-0.36	-0.88
YJL053W	PEP8	2899	3592	0.81	-0.31	-0.37	-0.88
YDR516C		2545	3154	0.81	-0.31	-0.37	-0.88
YOR178C	GAC1	1115	1382	0.81	-0.31	-0.37	-0.88
YLR466W	YRF1-4	5566	6898	0.81	-0.31	-0.37	-0.88
YGR016W		1070	1327	0.81	-0.31	-0.37	-0.88
YPL153C	RAD53	4266	5289	0.81	-0.31	-0.37	-0.88
YNL087W		1836	2276	0.81	-0.31	-0.37	-0.88
YDR186C		3421	4242	0.81	-0.31	-0.37	-0.89
YDL048C	STP4	6526	8097	0.81	-0.31	-0.37	-0.89
YJR029W		2236	2775	0.81	-0.31	-0.37	-0.89
YOR386W	PHR1	838	1040	0.81	-0.31	-0.37	-0.89
YLL015W	BPT1	7540	9359	0.81	-0.31	-0.37	-0.89
YML001W	YPT7	2207	2740	0.81	-0.31	-0.37	-0.89
YBR136W	ESR1	2313	2871	0.81	-0.31	-0.37	-0.89
YIL162W	SUC2	1517	1884	0.81	-0.31	-0.37	-0.89
YER152C		4863	6039	0.81	-0.31	-0.37	-0.89
YDR314C		1302	1617	0.80	-0.31	-0.37	-0.89
YBR097W	VPS15	814	1011	0.80	-0.31	-0.37	-0.89
YBR177C	EHT1	5283	6564	0.80	-0.31	-0.37	-0.89
YOR020C	HSP10	2410	2994	0.80	-0.31	-0.37	-0.89
YPR146C		523	650	0.80	-0.31	-0.37	-0.89
YLR336C	SGD1	518	644	0.80	-0.31	-0.37	-0.89
YDL015C		4359	5418	0.80	-0.31	-0.37	-0.89
YJL171C		602	748	0.80	-0.31	-0.37	-0.89
YJL043W		1265	1573	0.80	-0.31	-0.37	-0.89
YHR021C	RPS27B	634	789	0.80	-0.31	-0.37	-0.89
YOR356W		3945	4907	0.80	-0.31	-0.37	-0.90
YJL005W	CYR1	3114	3874	0.80	-0.31	-0.37	-0.90

YDR086C	SSS1	2624	3265	0.80	-0.32	-0.37	-0.90
YDR164C	SEC1	1049	1305	0.80	-0.32	-0.37	-0.90
YHR101C	BIG1	1533	1908	0.80	-0.32	-0.37	-0.90
YDR532C		1210	1506	0.80	-0.32	-0.37	-0.90
YML013C-A		906	1128	0.80	-0.32	-0.37	-0.90
YDR106W	ARP10	429	534	0.80	-0.32	-0.37	-0.90
YAL041W	CDC24	2366	2948	0.80	-0.32	-0.37	-0.90
YIL073C	SPO22	1102	1373	0.80	-0.32	-0.37	-0.90
YKLWdelta1	YKLWdelta1	1458	1817	0.80	-0.32	-0.37	-0.90
YHR082C	KSP1	1725	2151	0.80	-0.32	-0.37	-0.90
YIL005W		2523	3146	0.80	-0.32	-0.37	-0.90
YLR001C		1796	2240	0.80	-0.32	-0.38	-0.91
YLR372W	SUR4	839	1047	0.80	-0.32	-0.38	-0.91
YOL143C	RIB4	2642	3297	0.80	-0.32	-0.38	-0.91
YDL157C		2664	3326	0.80	-0.32	-0.38	-0.91
YJL068C		1241	1550	0.80	-0.32	-0.38	-0.91
YDR265W	PEX10	4754	5937	0.80	-0.32	-0.38	-0.91
YNL295W		1207	1508	0.80	-0.32	-0.38	-0.91
YFL025C	BST1	1587	1983	0.80	-0.32	-0.38	-0.91
YJL009W		1715	2142	0.80	-0.32	-0.38	-0.91
YPL222W		711	888	0.80	-0.32	-0.38	-0.91
YDR534C		2815	3518	0.80	-0.32	-0.38	-0.91
YKL143W	LTV1	2537	3172	0.80	-0.32	-0.38	-0.91
YHR049W		1866	2334	0.80	-0.32	-0.38	-0.92
YNL107W		3289	4114	0.80	-0.32	-0.38	-0.92
YML099C	ARG81	1126	1409	0.80	-0.32	-0.38	-0.92
YOL123W	HRP1	2895	3623	0.80	-0.32	-0.38	-0.92
YML091C	RPM2	2686	3362	0.80	-0.32	-0.38	-0.92
YOL008W		645	808	0.80	-0.32	-0.38	-0.92
YDL080C	THI3	3743	4686	0.80	-0.32	-0.38	-0.92
YNL205C		593	743	0.80	-0.32	-0.38	-0.92
YGR019W	UGA1	2172	2721	0.80	-0.32	-0.38	-0.92
YOR012W		323	405	0.80	-0.33	-0.38	-0.92
YNL284C	MRPL10	464	581	0.80	-0.33	-0.38	-0.92
YKR090W		440	551	0.80	-0.33	-0.38	-0.92
YKL095W	YJU2	743	932	0.80	-0.33	-0.38	-0.92

YDR331W	GPI8	2763	3464	0.80	-0.33	-0.38	-0.92
YKL160W		3206	4020	0.80	-0.33	-0.38	-0.92
YCRX20C		4541	5694	0.80	-0.33	-0.38	-0.92
YKL014C		2150	2697	0.80	-0.33	-0.38	-0.92
YER104W	RTT105	1804	2263	0.80	-0.33	-0.38	-0.93
YDL125C	HNT1	1815	2277	0.80	-0.33	-0.38	-0.93
YDR455C		2533	3178	0.80	-0.33	-0.38	-0.93
YER183C		4802	6025	0.80	-0.33	-0.38	-0.93
YAR008W	SEN34	1448	1817	0.80	-0.33	-0.38	-0.93
YDR489W		2703	3391	0.80	-0.33	-0.38	-0.93
YGR182C		627	787	0.80	-0.33	-0.38	-0.93
YKL044W		1369	1719	0.80	-0.33	-0.38	-0.93
YOL014W		3383	4248	0.80	-0.33	-0.38	-0.93
YDR239C		1032	1296	0.80	-0.33	-0.39	-0.93
YML027W	YOX1	2003	2517	0.80	-0.33	-0.39	-0.93
YKL064W	MNR2	3881	4878	0.80	-0.33	-0.39	-0.93
YJL023C	PET130	1018	1280	0.80	-0.33	-0.39	-0.93
YBL077W		2655	3338	0.80	-0.33	-0.39	-0.93
YOR183W		1417	1781	0.80	-0.33	-0.39	-0.93
YPR172W		657	827	0.80	-0.33	-0.39	-0.93
YLL033W		338	425	0.79	-0.33	-0.39	-0.94
YBR162W-A	YSY6	2058	2590	0.79	-0.33	-0.39	-0.94
YDR498C	SEC20	1924	2422	0.79	-0.33	-0.39	-0.94
YDR306C		823	1037	0.79	-0.33	-0.39	-0.94
YDL194W	SNF3	3258	4104	0.79	-0.33	-0.39	-0.94
YDR182W	CDC1	2506	3157	0.79	-0.33	-0.39	-0.94
YLR421C		2119	2669	0.79	-0.33	-0.39	-0.94
YLL053C		2588	3260	0.79	-0.33	-0.39	-0.94
YEL068C		1652	2081	0.79	-0.33	-0.39	-0.94
YGL064C		3383	4263	0.79	-0.33	-0.39	-0.94
YDR119W		3507	4419	0.79	-0.33	-0.39	-0.94
YFL011W	HXT10	861	1085	0.79	-0.33	-0.39	-0.94
YJL086C		1712	2158	0.79	-0.33	-0.39	-0.94
YNL123W		647	815	0.79	-0.33	-0.39	-0.94
YPR168W	NUT2	854	1076	0.79	-0.33	-0.39	-0.94
YBR152W	SPP381	2296	2895	0.79	-0.33	-0.39	-0.94

YLR362W	STE11	1260	1590	0.79	-0.34	-0.39	-0.94
YOR370C	MRS6	1986	2505	0.79	-0.34	-0.39	-0.94
YIL007C		1432	1807	0.79	-0.34	-0.39	-0.94
YKR074W		441	556	0.79	-0.34	-0.39	-0.94
YGL169W	SUA5	2466	3113	0.79	-0.34	-0.39	-0.95
YGL004C		1595	2013	0.79	-0.34	-0.39	-0.95
YML039W		8965	11321	0.79	-0.34	-0.39	-0.95
YILCdelta5	YILCdelta5	1455	1838	0.79	-0.34	-0.39	-0.95
YNL253W		1389	1754	0.79	-0.34	-0.39	-0.95
YLR239C		1452	1834	0.79	-0.34	-0.39	-0.95
YFL027C		2264	2862	0.79	-0.34	-0.39	-0.95
YFL054C		1337	1690	0.79	-0.34	-0.39	-0.95
YHR143W-A	RPC10	312	395	0.79	-0.34	-0.39	-0.95
YNL201C		2371	2998	0.79	-0.34	-0.39	-0.95
YJR050W	ISY1	1662	2105	0.79	-0.34	-0.40	-0.96
YLL057C		1053	1334	0.79	-0.34	-0.40	-0.96
YKR065C		651	825	0.79	-0.34	-0.40	-0.96
YBR087W	RFC5	1788	2265	0.79	-0.34	-0.40	-0.96
YORCdelta15	YORCdelta15	631	799	0.79	-0.34	-0.40	-0.96
YLR034C	SMF3	2734	3464	0.79	-0.34	-0.40	-0.96
YGR194C	XKS1	2737	3468	0.79	-0.34	-0.40	-0.96
YKL030W		1964	2491	0.79	-0.34	-0.40	-0.96
YBL055C		777	986	0.79	-0.34	-0.40	-0.96
YDR066C		355	450	0.79	-0.34	-0.40	-0.96
YLR400W		436	553	0.79	-0.34	-0.40	-0.96
YEL067C		3017	3828	0.79	-0.34	-0.40	-0.96
YJL120W		4985	6325	0.79	-0.34	-0.40	-0.96
YJL211C		1007	1279	0.79	-0.34	-0.40	-0.97
YAR042W	SWH1	1913	2429	0.79	-0.34	-0.40	-0.97
YCR020C-A	MAK31	1487	1888	0.79	-0.34	-0.40	-0.97
YML120C	NDI1	2630	3341	0.79	-0.35	-0.40	-0.97
YLR356W		1579	2006	0.79	-0.35	-0.40	-0.97
YDL035C	GPR1	3525	4480	0.79	-0.35	-0.40	-0.97
YML069W	POB3	1931	2455	0.79	-0.35	-0.40	-0.97
YFL040W		1340	1704	0.79	-0.35	-0.40	-0.97
YLR097C		1056	1343	0.79	-0.35	-0.40	-0.97

YOR191W	RIS1	1867	2375	0.79	-0.35	-0.40	-0.97
YGR003W		2859	3638	0.79	-0.35	-0.40	-0.98
YFL058W	THI5	868	1105	0.79	-0.35	-0.40	-0.98
YER138C		5276	6715	0.79	-0.35	-0.40	-0.98
YPL164C		1468	1868	0.79	-0.35	-0.40	-0.98
YLR434C		903	1150	0.79	-0.35	-0.40	-0.98
YHR131C		769	979	0.79	-0.35	-0.40	-0.98
YLR132C		1206	1536	0.79	-0.35	-0.41	-0.98
YLR067C	PET309	3659	4663	0.78	-0.35	-0.41	-0.98
YDR270W	CCC2	2590	3301	0.78	-0.35	-0.41	-0.98
YLR381W		1552	1979	0.78	-0.35	-0.41	-0.98
YER044C-A	MEI4	788	1005	0.78	-0.35	-0.41	-0.98
YMR211W		1437	1834	0.78	-0.35	-0.41	-0.98
YFL-TYB		10121	12917	0.78	-0.35	-0.41	-0.99
YBR153W	RIB7	2804	3579	0.78	-0.35	-0.41	-0.99
YBR150C	TBS1	5044	6438	0.78	-0.35	-0.41	-0.99
YDL001W		4698	5999	0.78	-0.35	-0.41	-0.99
YLL032C		1377	1758	0.78	-0.35	-0.41	-0.99
YML030W		1150	1469	0.78	-0.35	-0.41	-0.99
YKL189W	HYM1	1137	1452	0.78	-0.35	-0.41	-0.99
YMR030W		2221	2836	0.78	-0.35	-0.41	-0.99
YDL109C		1884	2407	0.78	-0.35	-0.41	-0.99
YGR022C		540	690	0.78	-0.35	-0.41	-0.99
YPL058C	PDR12	1102	1408	0.78	-0.35	-0.41	-0.99
YNR046W		1126	1439	0.78	-0.35	-0.41	-0.99
YER168C	CCA1	3797	4854	0.78	-0.35	-0.41	-0.99
YLR283W		510	652	0.78	-0.36	-0.41	-0.99
YBR085W	AAC3	871	1114	0.78	-0.36	-0.41	-0.99
YNL162W	RPL42A	449	575	0.78	-0.36	-0.41	-1.00
YHR185C	ADY1	1791	2293	0.78	-0.36	-0.41	-1.00
YEL035C	UTR5	1859	2380	0.78	-0.36	-0.41	-1.00
YDR461W	MFA1	1208	1549	0.78	-0.36	-0.41	-1.00
YIL082W		4602	5900	0.78	-0.36	-0.41	-1.00
YPR073C	LTP1	381	489	0.78	-0.36	-0.41	-1.00
YJR161C	COS5	1359	1742	0.78	-0.36	-0.41	-1.00
YMR104C	YPK2	2290	2939	0.78	-0.36	-0.42	-1.01

YER017C	AFG3	2711	3480	0.78	-0.36	-0.42	-1.01
YDL089W		2497	3206	0.78	-0.36	-0.42	-1.01
YPR119W	CLB2	3941	5062	0.78	-0.36	-0.42	-1.01
YLR352W		515	661	0.78	-0.36	-0.42	-1.01
YAR003W		1722	2212	0.78	-0.36	-0.42	-1.01
YDR418W	RPL12B	3268	4200	0.78	-0.36	-0.42	-1.01
YOR161C		1539	1978	0.78	-0.36	-0.42	-1.01
YOR357C	GRD19	768	987	0.78	-0.36	-0.42	-1.01
YMR156C		1342	1725	0.78	-0.36	-0.42	-1.01
YLL005C		2816	3622	0.78	-0.36	-0.42	-1.01
YKL033W-A	YKL033W-A	2500	3216	0.78	-0.36	-0.42	-1.01
YOR279C		367	472	0.78	-0.36	-0.42	-1.01
YKL179C		532	684	0.78	-0.36	-0.42	-1.01
YDL094C		1677	2158	0.78	-0.36	-0.42	-1.01
YDRCTy1-2D	YDRCTy1-2D	15850	20415	0.78	-0.37	-0.42	-1.02
YEL069C	HXT13	3481	4489	0.78	-0.37	-0.42	-1.02
YDR525W		679	876	0.78	-0.37	-0.42	-1.02
YLR438C-A	YLR438C-A	2165	2792	0.78	-0.37	-0.42	-1.02
YMR158C-B	YMR158C-B	4703	6065	0.78	-0.37	-0.42	-1.02
YLL018C-A	YLL018C-A	630	812	0.78	-0.37	-0.42	-1.02
YOR203W		392	506	0.78	-0.37	-0.42	-1.02
YFR002W	NIC96	3166	4084	0.78	-0.37	-0.42	-1.02
YDL149W	AUT9	1263	1629	0.78	-0.37	-0.42	-1.02
YLR086W	SMC4	3116	4021	0.77	-0.37	-0.42	-1.02
YNL136W		3016	3894	0.77	-0.37	-0.42	-1.03
YGL255W	ZRT1	314	405	0.77	-0.37	-0.43	-1.03
YBR215W	HPC2	2393	3090	0.77	-0.37	-0.43	-1.03
YGL260W		1903	2459	0.77	-0.37	-0.43	-1.03
YPL165C		373	482	0.77	-0.37	-0.43	-1.03
YHR128W	FUR1	1845	2385	0.77	-0.37	-0.43	-1.03
YAL062W	GDH3	964	1246	0.77	-0.37	-0.43	-1.03
YJR103W	URA8	2416	3123	0.77	-0.37	-0.43	-1.03
YMR316W		2993	3871	0.77	-0.37	-0.43	-1.03
YDR470C		3445	4456	0.77	-0.37	-0.43	-1.03
YJL019W		2117	2738	0.77	-0.37	-0.43	-1.03
YER015W	FAA2	1535	1987	0.77	-0.37	-0.43	-1.03

YKL034W		3504	4535	0.77	-0.37	-0.43	-1.03
YPR012W		1287	1666	0.77	-0.37	-0.43	-1.03
YPR087W		924	1196	0.77	-0.37	-0.43	-1.03
YBR072W	HSP26	2459	3184	0.77	-0.37	-0.43	-1.04
YJR086W	STE18	684	886	0.77	-0.37	-0.43	-1.04
YIL015W	BAR1	328	425	0.77	-0.37	-0.43	-1.04
YGL106W	MLC1	2446	3168	0.77	-0.37	-0.43	-1.04
YNR062C		539	698	0.77	-0.37	-0.43	-1.04
YFR057W		3280	4250	0.77	-0.37	-0.43	-1.04
YCR093W	CDC39	9215	11940	0.77	-0.37	-0.43	-1.04
YGL083W	SCY1	3000	3888	0.77	-0.37	-0.43	-1.04
YDR473C	PRP3	3068	3976	0.77	-0.37	-0.43	-1.04
YCLX02C		3520	4562	0.77	-0.37	-0.43	-1.04
YKL166C	TPK3	1327	1720	0.77	-0.37	-0.43	-1.04
YNL056W		332	430	0.77	-0.37	-0.43	-1.04
YFL052W		925	1199	0.77	-0.37	-0.43	-1.04
YIL174W		1175	1523	0.77	-0.37	-0.43	-1.04
YCR032W	BPH1	4485	5817	0.77	-0.38	-0.43	-1.04
YOL091W	SPO21	575	746	0.77	-0.38	-0.43	-1.04
YDR464W	SPP41	1046	1357	0.77	-0.38	-0.43	-1.04
YAR053W		1055	1368	0.77	-0.38	-0.43	-1.04
YBL026W	LSM2	1491	1934	0.77	-0.38	-0.43	-1.04
YHR001W-A	QCR10	2710	3517	0.77	-0.38	-0.43	-1.04
YLR240W	VPS34	2910	3777	0.77	-0.38	-0.43	-1.04
YBR053C		802	1041	0.77	-0.38	-0.43	-1.05
YOR199W		1544	2005	0.77	-0.38	-0.43	-1.05
YDRCTy1-2A	YDRCTy1-2A	10355	13455	0.77	-0.38	-0.43	-1.05
YOL137W		857	1114	0.77	-0.38	-0.43	-1.05
YLR447C	VMA6	1511	1964	0.77	-0.38	-0.43	-1.05
YDL240W	LRG1	1411	1834	0.77	-0.38	-0.43	-1.05
YNR022C		1573	2045	0.77	-0.38	-0.43	-1.05
YOL072W		1814	2359	0.77	-0.38	-0.44	-1.05
YJR038C		1154	1501	0.77	-0.38	-0.44	-1.05
YBR190W		1009	1312	0.77	-0.38	-0.44	-1.05
YGL102C		7196	9361	0.77	-0.38	-0.44	-1.05
YJL070C		2799	3644	0.77	-0.38	-0.44	-1.05

YBR166C	TYR1	2934	3821	0.77	-0.38	-0.44	-1.06
YJR034W	PET191	1445	1882	0.77	-0.38	-0.44	-1.06
YOL115W	TRF4	1591	2073	0.77	-0.38	-0.44	-1.06
YML011C		3502	4563	0.77	-0.38	-0.44	-1.06
YPLWdelta9	YPLWdelta9	528	688	0.77	-0.38	-0.44	-1.06
YDR115W		5023	6549	0.77	-0.38	-0.44	-1.06
YCR048W	ARE1	4016	5238	0.77	-0.38	-0.44	-1.06
YGRWdelta26	YGRWdelta26	1353	1764	0.77	-0.38	-0.44	-1.06
YPR177C		1551	2023	0.77	-0.38	-0.44	-1.06
YGR081C		1245	1624	0.77	-0.38	-0.44	-1.06
YDL171C	GLT1	6949	9067	0.77	-0.38	-0.44	-1.06
YPL141C		3099	4044	0.77	-0.38	-0.44	-1.06
YDR144C	MKC7	1889	2466	0.77	-0.38	-0.44	-1.06
YDR504C		1311	1712	0.77	-0.38	-0.44	-1.06
YGL138C		1782	2327	0.77	-0.38	-0.44	-1.06
YER140W		1474	1924	0.77	-0.38	-0.44	-1.06
YCR098C	GIT1	2107	2751	0.77	-0.38	-0.44	-1.06
YKR015C		2970	3879	0.77	-0.39	-0.44	-1.07
YHR208W	BAT1	645	843	0.77	-0.39	-0.44	-1.07
YBR262C		1357	1773	0.77	-0.39	-0.44	-1.07
YBL106C	SRO77	2337	3053	0.77	-0.39	-0.44	-1.07
YOL122C	SMF1	2367	3093	0.77	-0.39	-0.44	-1.07
YIL069C	RPS24B	2505	3274	0.77	-0.39	-0.44	-1.07
YLL004W	ORC3	969	1267	0.76	-0.39	-0.44	-1.07
YPR049C		1178	1541	0.76	-0.39	-0.44	-1.07
YBLWdelta8	YBLWdelta8	823	1077	0.76	-0.39	-0.44	-1.07
YHR090C	NBN1	1982	2593	0.76	-0.39	-0.44	-1.07
YLR276C	DBP9	2741	3587	0.76	-0.39	-0.44	-1.07
YBR193C	MED8	3025	3958	0.76	-0.39	-0.44	-1.07
YER172C	BRR2	4636	6069	0.76	-0.39	-0.44	-1.07
YAL058W	CNE1	1527	1999	0.76	-0.39	-0.44	-1.07
YOR193W		1497	1960	0.76	-0.39	-0.45	-1.08
YJL018W		1844	2416	0.76	-0.39	-0.45	-1.08
YML072C		5847	7661	0.76	-0.39	-0.45	-1.08
YOR180C	DCI1	3221	4220	0.76	-0.39	-0.45	-1.08
YGR018C		2330	3053	0.76	-0.39	-0.45	-1.08

YPL121C	MEI5	2474	3243	0.76	-0.39	-0.45	-1.08
YAR015W	ADE1	4756	6239	0.76	-0.39	-0.45	-1.08
YKL037W		1992	2613	0.76	-0.39	-0.45	-1.08
YOL024W		1751	2298	0.76	-0.39	-0.45	-1.08
YGR196C		3559	4673	0.76	-0.39	-0.45	-1.08
YNR055C	HOL1	2887	3791	0.76	-0.39	-0.45	-1.09
YAL031C	FUN21	1684	2212	0.76	-0.39	-0.45	-1.09
YGRCTy1-2A	YGRCTy1-2A	11244	14768	0.76	-0.39	-0.45	-1.09
YDR453C		3356	4410	0.76	-0.39	-0.45	-1.09
YOR137C		731	961	0.76	-0.39	-0.45	-1.09
YBR197C		1602	2105	0.76	-0.39	-0.45	-1.09
YBR098W	MMS4	1442	1895	0.76	-0.39	-0.45	-1.09
YJL037W		775	1019	0.76	-0.39	-0.45	-1.09
YML055W	SPC2	1411	1856	0.76	-0.40	-0.45	-1.09
YDR183W		3539	4657	0.76	-0.40	-0.45	-1.09
YDR131C		1067	1404	0.76	-0.40	-0.45	-1.09
YML026C	RPS18B	4369	5750	0.76	-0.40	-0.45	-1.09
YJL176C	SWI3	2241	2950	0.76	-0.40	-0.45	-1.09
YMR162C		820	1079	0.76	-0.40	-0.45	-1.09
YMR166C		1106	1456	0.76	-0.40	-0.45	-1.10
YOR086C		700	922	0.76	-0.40	-0.45	-1.10
YKL111C		2285	3011	0.76	-0.40	-0.45	-1.10
YLR418C	CDC73	1612	2124	0.76	-0.40	-0.45	-1.10
YML076C		3002	3956	0.76	-0.40	-0.45	-1.10
YLL046C	RNP1	3611	4760	0.76	-0.40	-0.45	-1.10
YDR521W		2756	3634	0.76	-0.40	-0.46	-1.10
YDR435C		1325	1747	0.76	-0.40	-0.46	-1.10
YPR141C	KAR3	854	1126	0.76	-0.40	-0.46	-1.10
YLR462W		3136	4137	0.76	-0.40	-0.46	-1.10
YIL027C		5442	7179	0.76	-0.40	-0.46	-1.10
YHR211W	FLO5	1451	1914	0.76	-0.40	-0.46	-1.10
YDRWdelta12	YDRWdelta12	12403	16366	0.76	-0.40	-0.46	-1.10
YOR100C	CRC1	374	494	0.76	-0.40	-0.46	-1.10
YGR067C		2052	2711	0.76	-0.40	-0.46	-1.11
YPR116W		524	693	0.76	-0.40	-0.46	-1.11
YCR095C		1532	2025	0.76	-0.40	-0.46	-1.11

YPR044C		989	1308	0.76	-0.40	-0.46	-1.11
YOR257W	CDC31	346	458	0.76	-0.40	-0.46	-1.11
YIL116W	HIS5	1142	1511	0.76	-0.40	-0.46	-1.11
YKL176C		3233	4279	0.76	-0.40	-0.46	-1.11
YHR174W	ENO2	753	998	0.75	-0.41	-0.46	-1.12
YMR123W	PKR1	1468	1945	0.75	-0.41	-0.46	-1.12
YDL049C	KNH1	1011	1340	0.75	-0.41	-0.46	-1.12
YDR008C		1309	1735	0.75	-0.41	-0.46	-1.12
YER078C		2690	3567	0.75	-0.41	-0.46	-1.12
YLR318W	EST2	559	742	0.75	-0.41	-0.46	-1.12
YEL023C		1877	2492	0.75	-0.41	-0.47	-1.12
YLRWdelta12	YLRWdelta12	1121	1489	0.75	-0.41	-0.47	-1.12
YMR216C	SKY1	3036	4032	0.75	-0.41	-0.47	-1.12
YKL026C		2149	2855	0.75	-0.41	-0.47	-1.12
YDR170C	SEC7	4271	5676	0.75	-0.41	-0.47	-1.13
YGR045C		1997	2654	0.75	-0.41	-0.47	-1.13
YMR051C		8926	11865	0.75	-0.41	-0.47	-1.13
YNL103W	MET4	1963	2609	0.75	-0.41	-0.47	-1.13
YER138W-A	YER138W-A	580	771	0.75	-0.41	-0.47	-1.13
YER124C		2844	3783	0.75	-0.41	-0.47	-1.13
YKL080W	VMA5	2991	3978	0.75	-0.41	-0.47	-1.13
YLR018C		2879	3830	0.75	-0.41	-0.47	-1.13
YMR172C-A		1583	2106	0.75	-0.41	-0.47	-1.13
YJL225C		1736	2312	0.75	-0.41	-0.47	-1.13
YOL003C		1091	1453	0.75	-0.41	-0.47	-1.13
YGR239C	PEX21	1452	1934	0.75	-0.41	-0.47	-1.13
YKL090W		2413	3215	0.75	-0.41	-0.47	-1.13
YFR014C	CMK1	1526	2033	0.75	-0.41	-0.47	-1.14
YGRCTy1-2C	YGRCTy1-2C	14979	19968	0.75	-0.41	-0.47	-1.14
YJR055W	HIT1	386	515	0.75	-0.42	-0.47	-1.14
YDR438W		1550	2067	0.75	-0.42	-0.47	-1.14
YGL164C		2448	3266	0.75	-0.42	-0.47	-1.14
YNL199C	GCR2	3941	5258	0.75	-0.42	-0.47	-1.14
YAR037W		2833	3781	0.75	-0.42	-0.47	-1.14
YAR009C		5224	6973	0.75	-0.42	-0.47	-1.14
YOR010C	TIR2	619	826	0.75	-0.42	-0.47	-1.14

YMRWdelta21	YMRWdelta21	430	574	0.75	-0.42	-0.47	-1.14
YDR340W		981	1310	0.75	-0.42	-0.47	-1.14
YLR419W		2573	3437	0.75	-0.42	-0.47	-1.14
YGL020C		731	977	0.75	-0.42	-0.47	-1.14
YAR014C		1872	2502	0.75	-0.42	-0.47	-1.15
YLR089C		1314	1756	0.75	-0.42	-0.47	-1.15
YBR090C		1165	1557	0.75	-0.42	-0.47	-1.15
YCL029C	BIK1	8925	11933	0.75	-0.42	-0.48	-1.15
YPL216W		1453	1943	0.75	-0.42	-0.48	-1.15
YCR001W		3989	5339	0.75	-0.42	-0.48	-1.15
YPR082C	DIB1	1331	1782	0.75	-0.42	-0.48	-1.15
YGL079W		490	657	0.75	-0.42	-0.48	-1.15
YPR059C		433	581	0.75	-0.42	-0.48	-1.16
YHR127W	HSN1	967	1297	0.75	-0.42	-0.48	-1.16
YBR183W	YPC1	1247	1672	0.75	-0.42	-0.48	-1.16
YMR194C-A	YMR194C-A	2412	3234	0.75	-0.42	-0.48	-1.16
YDR151C	CTH1	1285	1723	0.75	-0.42	-0.48	-1.16
YHR193C	EGD2	2022	2712	0.75	-0.42	-0.48	-1.16
YNL252C	MRPL17	371	497	0.75	-0.42	-0.48	-1.16
YNR040W		5334	7155	0.75	-0.42	-0.48	-1.16
YKL147C		1744	2339	0.75	-0.42	-0.48	-1.16
YMR128W	ECM16	3107	4169	0.75	-0.42	-0.48	-1.16
YBR274W	CHK1	694	931	0.75	-0.42	-0.48	-1.16
YJL064W		304	408	0.74	-0.42	-0.48	-1.16
YJL003W		1343	1803	0.74	-0.43	-0.48	-1.16
YDR252W	BTT1	1790	2403	0.74	-0.43	-0.48	-1.16
YGL247W		2337	3138	0.74	-0.43	-0.48	-1.16
YMR118C		561	753	0.74	-0.43	-0.48	-1.16
YCR019W	MAK32	4272	5741	0.74	-0.43	-0.48	-1.17
YLR245C	CDD1	430	578	0.74	-0.43	-0.48	-1.17
YNL022C		6478	8712	0.74	-0.43	-0.48	-1.17
YML092C	PRE8	1894	2547	0.74	-0.43	-0.48	-1.17
YMR179W	SPT21	2255	3033	0.74	-0.43	-0.48	-1.17
YOR353C		2142	2882	0.74	-0.43	-0.48	-1.17
YOR018W	ROD1	1001	1348	0.74	-0.43	-0.48	-1.17
YOLCdelta8	YOLCdelta8	498	670	0.74	-0.43	-0.49	-1.17

YPR156C		929	1251	0.74	-0.43	-0.49	-1.17
YML101C		2041	2750	0.74	-0.43	-0.49	-1.17
YMR259C		473	638	0.74	-0.43	-0.49	-1.17
YFR018C		951	1281	0.74	-0.43	-0.49	-1.18
YBR214W	SDS24	1030	1389	0.74	-0.43	-0.49	-1.18
YCRX07W		896	1208	0.74	-0.43	-0.49	-1.18
YHR032W		442	596	0.74	-0.43	-0.49	-1.18
YBR295W	PCA1	2033	2744	0.74	-0.43	-0.49	-1.18
YLR099C	ICT1	2351	3175	0.74	-0.43	-0.49	-1.18
YKR018C		992	1339	0.74	-0.43	-0.49	-1.18
YIL036W		2412	3257	0.74	-0.43	-0.49	-1.18
YNL222W	SSU72	2118	2860	0.74	-0.43	-0.49	-1.18
YGL057C		1707	2307	0.74	-0.43	-0.49	-1.18
YMR291W		2640	3567	0.74	-0.43	-0.49	-1.18
YDR543C		2259	3055	0.74	-0.44	-0.49	-1.19
YNR011C	PRP2	2755	3725	0.74	-0.44	-0.49	-1.19
YOL023W	IFM1	2361	3193	0.74	-0.44	-0.49	-1.19
YBR099C		3010	4072	0.74	-0.44	-0.49	-1.19
YGR275W	RTT102	5162	6983	0.74	-0.44	-0.49	-1.19
YGR212W		1994	2698	0.74	-0.44	-0.49	-1.19
YGR039W		879	1190	0.74	-0.44	-0.49	-1.19
YKR003W		1739	2355	0.74	-0.44	-0.49	-1.19
YBR182C	SMP1	1144	1550	0.74	-0.44	-0.49	-1.19
YBLWdelta3	YBLWdelta3	1516	2054	0.74	-0.44	-0.49	-1.19
YJR102C		1550	2100	0.74	-0.44	-0.49	-1.19
YHR079C	IRE1	2549	3454	0.74	-0.44	-0.49	-1.19
YDL224C	WHI4	2169	2939	0.74	-0.44	-0.49	-1.19
YLR430W	SEN1	2459	3335	0.74	-0.44	-0.50	-1.20
YKL065C	YET1	1314	1782	0.74	-0.44	-0.50	-1.20
YLR111W		2792	3788	0.74	-0.44	-0.50	-1.20
YDL129W		511	694	0.74	-0.44	-0.50	-1.20
YDR406W	PDR15	5919	8039	0.74	-0.44	-0.50	-1.20
YOR072W		1517	2061	0.74	-0.44	-0.50	-1.20
YPL074W	YTA6	518	704	0.74	-0.44	-0.50	-1.20
YFR021W		1771	2407	0.74	-0.44	-0.50	-1.20
YDR407C	TRS120	3998	5434	0.74	-0.44	-0.50	-1.20

YKL162C-A	YKL162C-A	2034	2765	0.74	-0.44	-0.50	-1.20
YPL252C	YAH1	760	1034	0.74	-0.44	-0.50	-1.21
YPL038W	MET31	597	812	0.74	-0.44	-0.50	-1.21
YCR097WB		2999	4080	0.74	-0.44	-0.50	-1.21
YCL053C		1957	2662	0.74	-0.44	-0.50	-1.21
YBL104C		437	595	0.73	-0.44	-0.50	-1.21
YDR138W	HPR1	2779	3783	0.73	-0.45	-0.50	-1.21
YNL029C	KTR5	1305	1777	0.73	-0.45	-0.50	-1.21
YDR244W	PEX5	2741	3733	0.73	-0.45	-0.50	-1.21
YNR066C		515	701	0.73	-0.45	-0.50	-1.21
YMR165C	SMP2	3875	5281	0.73	-0.45	-0.50	-1.21
YGL163C	RAD54	925	1261	0.73	-0.45	-0.50	-1.21
YER066C-A		450	614	0.73	-0.45	-0.50	-1.21
YKL131W		1554	2118	0.73	-0.45	-0.50	-1.22
YPR192W	AQY1	653	890	0.73	-0.45	-0.50	-1.22
YIL033C	SRA1	3663	4995	0.73	-0.45	-0.50	-1.22
YBR102C	EXO84	2257	3079	0.73	-0.45	-0.50	-1.22
YLR145W		682	930	0.73	-0.45	-0.50	-1.22
YLR064W		1673	2283	0.73	-0.45	-0.50	-1.22
YDR319C		1066	1454	0.73	-0.45	-0.50	-1.22
YER076C		2233	3048	0.73	-0.45	-0.51	-1.22
YML096W		4222	5770	0.73	-0.45	-0.51	-1.22
YGL199C		1224	1673	0.73	-0.45	-0.51	-1.22
YKL018W		1228	1678	0.73	-0.45	-0.51	-1.22
YJL131C		808	1104	0.73	-0.45	-0.51	-1.22
YBR178W		913	1248	0.73	-0.45	-0.51	-1.22
YNL206C		1107	1513	0.73	-0.45	-0.51	-1.22
YHR067W		3410	4663	0.73	-0.45	-0.51	-1.23
YGL052W		592	810	0.73	-0.45	-0.51	-1.23
YBR297W	MAL33	974	1332	0.73	-0.45	-0.51	-1.23
YOR094W	ARF3	487	666	0.73	-0.45	-0.51	-1.23
YNL238W	KEX2	2396	3280	0.73	-0.45	-0.51	-1.23
YBR213W	MET8	2368	3242	0.73	-0.45	-0.51	-1.23
YML070W	DAK1	1514	2072	0.73	-0.45	-0.51	-1.23
YIR015W	RPR2	1942	2659	0.73	-0.45	-0.51	-1.23
YNR016C	ACC1	5854	8026	0.73	-0.46	-0.51	-1.23

YJL035C	TAD2	1236	1695	0.73	-0.46	-0.51	-1.24
YDR246W	TRS23	1047	1437	0.73	-0.46	-0.51	-1.24
YML108W		896	1230	0.73	-0.46	-0.51	-1.24
YLR431C		1018	1398	0.73	-0.46	-0.51	-1.24
YKL003C	MRP17	1171	1608	0.73	-0.46	-0.51	-1.24
YGR044C	RME1	1992	2737	0.73	-0.46	-0.51	-1.24
YBR012W-A		7653	10516	0.73	-0.46	-0.51	-1.24
YBR278W	DPB3	1587	2182	0.73	-0.46	-0.52	-1.24
YIL037C		6753	9284	0.73	-0.46	-0.52	-1.24
YNL167C	SKO1	2630	3616	0.73	-0.46	-0.52	-1.24
YNL260C		452	621	0.73	-0.46	-0.52	-1.24
YMR050C		12748	17531	0.73	-0.46	-0.52	-1.25
YCR083W	TRX3	1129	1553	0.73	-0.46	-0.52	-1.25
YNR039C		3245	4466	0.73	-0.46	-0.52	-1.25
YIR039C	YPS6	305	420	0.73	-0.46	-0.52	-1.25
YMR065W	KAR5	1144	1575	0.73	-0.46	-0.52	-1.25
YHL009C	YAP3	2704	3723	0.73	-0.46	-0.52	-1.25
YMR213W	CEF1	1593	2195	0.73	-0.46	-0.52	-1.25
YER004W		2753	3793	0.73	-0.46	-0.52	-1.25
YGR052W		676	931	0.73	-0.46	-0.52	-1.25
YDR371W		3057	4213	0.73	-0.46	-0.52	-1.25
YJL136C	RPS21B	2104	2900	0.73	-0.46	-0.52	-1.25
YCL022C		3755	5175	0.73	-0.46	-0.52	-1.25
YIR017C	MET28	633	873	0.73	-0.46	-0.52	-1.25
YBR260C	RGD1	7502	10345	0.73	-0.46	-0.52	-1.25
YDL161W	ENT1	7638	10535	0.73	-0.46	-0.52	-1.26
YBL079W	NUP170	6609	9117	0.72	-0.46	-0.52	-1.26
YDL050C		3100	4277	0.72	-0.46	-0.52	-1.26
YMR290C	HAS1	456	630	0.72	-0.46	-0.52	-1.26
YMR126C		1747	2413	0.72	-0.47	-0.52	-1.26
YJR026W		6227	8601	0.72	-0.47	-0.52	-1.26
YHR190W	ERG9	516	713	0.72	-0.47	-0.52	-1.26
YGRCdelta20	YGRCdelta20	1217	1683	0.72	-0.47	-0.52	-1.27
YKL142W	MRP8	693	958	0.72	-0.47	-0.52	-1.27
YNL143C		2408	3332	0.72	-0.47	-0.52	-1.27
YHR075C		2671	3696	0.72	-0.47	-0.52	-1.27

YLR405W		2829	3914	0.72	-0.47	-0.52	-1.27
YDR214W		2274	3147	0.72	-0.47	-0.52	-1.27
YHRCdelta12	YHRCdelta12	809	1120	0.72	-0.47	-0.53	-1.27
YNL011C		503	696	0.72	-0.47	-0.53	-1.27
YHR210C		998	1382	0.72	-0.47	-0.53	-1.27
YDR229W		2848	3945	0.72	-0.47	-0.53	-1.27
YDR241W		796	1102	0.72	-0.47	-0.53	-1.27
YLR302C		2167	3003	0.72	-0.47	-0.53	-1.27
YMR100W	MUB1	914	1267	0.72	-0.47	-0.53	-1.27
YJR018W		828	1149	0.72	-0.47	-0.53	-1.28
YPL010W	RET3	613	850	0.72	-0.47	-0.53	-1.28
YER094C	PUP3	1820	2525	0.72	-0.47	-0.53	-1.28
YIL085C	KTR7	3326	4617	0.72	-0.47	-0.53	-1.28
YLR450W	HMG2	1598	2219	0.72	-0.47	-0.53	-1.28
YLR048W	RPS0B	2031	2822	0.72	-0.47	-0.53	-1.28
YDR501W	PLM2	2657	3692	0.72	-0.47	-0.53	-1.28
YPR061C		865	1202	0.72	-0.48	-0.53	-1.28
YOL043C	NTG2	1739	2418	0.72	-0.48	-0.53	-1.28
YDR166C	SEC5	1040	1447	0.72	-0.48	-0.53	-1.29
YBL078C	AUT7	1853	2578	0.72	-0.48	-0.53	-1.29
YIL144W	TID3	3343	4653	0.72	-0.48	-0.53	-1.29
YGL259W	YPS5	2624	3652	0.72	-0.48	-0.53	-1.29
YMR319C	FET4	1434	1996	0.72	-0.48	-0.53	-1.29
YOL116W	MSN1	2077	2893	0.72	-0.48	-0.53	-1.29
YGR038W	ORM1	518	722	0.72	-0.48	-0.53	-1.29
YMR045C		5875	8185	0.72	-0.48	-0.53	-1.29
YML013W		1863	2597	0.72	-0.48	-0.53	-1.29
YPL192C		655	913	0.72	-0.48	-0.54	-1.29
YDR025W	RPS11A	2314	3226	0.72	-0.48	-0.54	-1.29
YHR133C		2198	3064	0.72	-0.48	-0.54	-1.29
YLR156W		747	1042	0.72	-0.48	-0.54	-1.29
YBL018C	POP8	523	730	0.72	-0.48	-0.54	-1.30
YGR080W	TWF1	1277	1783	0.72	-0.48	-0.54	-1.30
YPL152W	RRD2	2143	2992	0.72	-0.48	-0.54	-1.30
YBR289W	SNF5	3748	5233	0.72	-0.48	-0.54	-1.30
YBR186W	PCH2	2418	3379	0.72	-0.48	-0.54	-1.30

YLR308W	CDA2	5668	7921	0.72	-0.48	-0.54	-1.30
YFL011W-A		2129	2976	0.72	-0.48	-0.54	-1.30
YJL161W		398	557	0.72	-0.48	-0.54	-1.30
YDR271C		1646	2302	0.71	-0.48	-0.54	-1.31
YGL015C		1405	1966	0.71	-0.49	-0.54	-1.31
YDR466W		2452	3433	0.71	-0.49	-0.54	-1.31
YJL152W		2958	4143	0.71	-0.49	-0.54	-1.31
YMR122C		2031	2844	0.71	-0.49	-0.54	-1.31
YLR278C		1087	1523	0.71	-0.49	-0.54	-1.31
YCL042W		2067	2896	0.71	-0.49	-0.54	-1.31
YGR248W	SOL4	647	906	0.71	-0.49	-0.54	-1.31
YJR125C	ENT3	2767	3877	0.71	-0.49	-0.54	-1.31
YGL140C		2994	4196	0.71	-0.49	-0.54	-1.31
YKL222C		639	896	0.71	-0.49	-0.54	-1.31
YAR069C		3665	5138	0.71	-0.49	-0.54	-1.31
YGL133W		2557	3586	0.71	-0.49	-0.54	-1.31
YML057W	CMP2	4686	6571	0.71	-0.49	-0.54	-1.31
YDRWdelta31	YDRWdelta31	461	647	0.71	-0.49	-0.54	-1.31
YKR029C		1842	2584	0.71	-0.49	-0.54	-1.32
YJL200C		1601	2246	0.71	-0.49	-0.54	-1.32
YNL233W	BNI4	1762	2473	0.71	-0.49	-0.55	-1.32
YCL023C		4646	6522	0.71	-0.49	-0.55	-1.32
YEL033W		1540	2164	0.71	-0.49	-0.55	-1.32
YDR537C		1801	2530	0.71	-0.49	-0.55	-1.32
YDL216C		553	778	0.71	-0.49	-0.55	-1.32
YPR001W	CIT3	810	1139	0.71	-0.49	-0.55	-1.32
YPR050C		972	1368	0.71	-0.49	-0.55	-1.33
YKL164C	PIR1	2092	2944	0.71	-0.49	-0.55	-1.33
YAL063C	FLO9	1471	2073	0.71	-0.49	-0.55	-1.33
YOR243C		1012	1427	0.71	-0.50	-0.55	-1.33
YML010W-A		1742	2457	0.71	-0.50	-0.55	-1.33
YJRWdelta14	YJRWdelta14	315	444	0.71	-0.50	-0.55	-1.33
YDL093W	PMT5	3559	5019	0.71	-0.50	-0.55	-1.33
YPR095C	SYT1	2384	3363	0.71	-0.50	-0.55	-1.33
YOL009C	MDM12	701	989	0.71	-0.50	-0.55	-1.33
YIL052C	RPL34B	1275	1799	0.71	-0.50	-0.55	-1.33

YIL165C		1201	1695	0.71	-0.50	-0.55	-1.34
YNL216W	RAP1	1710	2416	0.71	-0.50	-0.55	-1.34
YDR321W	ASP1	1925	2721	0.71	-0.50	-0.56	-1.34
YCL010C		2538	3588	0.71	-0.50	-0.56	-1.34
YGR015C		1298	1836	0.71	-0.50	-0.56	-1.34
YDR456W	NHX1	2012	2848	0.71	-0.50	-0.56	-1.35
YOR162C	YRR1	1824	2581	0.71	-0.50	-0.56	-1.35
YMR133W	REC114	952	1348	0.71	-0.50	-0.56	-1.35
YLR463C		2054	2909	0.71	-0.50	-0.56	-1.35
YLRCdelta16	YLRCdelta16	990	1404	0.71	-0.50	-0.56	-1.35
YBL093C	ROX3	2244	3182	0.71	-0.50	-0.56	-1.35
YOR305W		1109	1573	0.71	-0.50	-0.56	-1.35
YIL077C		742	1053	0.70	-0.50	-0.56	-1.35
YLR157C	ASP3-2	713	1012	0.70	-0.50	-0.56	-1.35
YGR142W	BTN2	981	1392	0.70	-0.51	-0.56	-1.36
YDR484W	SAC2	1554	2205	0.70	-0.51	-0.56	-1.36
YAL010C	MDM10	1989	2824	0.70	-0.51	-0.56	-1.36
YDR281C		3102	4404	0.70	-0.51	-0.56	-1.36
YML081C-A	YML081C-A	2471	3510	0.70	-0.51	-0.56	-1.36
YDR457W	TOM1	1693	2405	0.70	-0.51	-0.56	-1.36
YDR313C	PIB1	2529	3596	0.70	-0.51	-0.56	-1.36
YCRX04W		2991	4255	0.70	-0.51	-0.56	-1.36
YIL063C	YRB2	622	886	0.70	-0.51	-0.57	-1.37
YCL006C		7584	10796	0.70	-0.51	-0.57	-1.37
YJLWdelta16	YJLWdelta16	667	950	0.70	-0.51	-0.57	-1.37
YMR119W-A		1844	2628	0.70	-0.51	-0.57	-1.37
YGR225W	SPO70	980	1396	0.70	-0.51	-0.57	-1.37
YJL067W		2509	3576	0.70	-0.51	-0.57	-1.37
YMR062C	ECM40	1244	1773	0.70	-0.51	-0.57	-1.37
YKL224C		1430	2039	0.70	-0.51	-0.57	-1.37
YGR288W	MAL13	881	1257	0.70	-0.51	-0.57	-1.37
YKL211C	TRP3	921	1314	0.70	-0.51	-0.57	-1.37
YGR110W		448	639	0.70	-0.51	-0.57	-1.37
YGL113W		1012	1446	0.70	-0.51	-0.57	-1.38
YGL065C	ALG2	498	711	0.70	-0.51	-0.57	-1.38
YOR333C		350	500	0.70	-0.51	-0.57	-1.38

YMR150C	IMP1	976	1395	0.70	-0.52	-0.57	-1.38
YJL039C	NUP192	963	1376	0.70	-0.52	-0.57	-1.38
YCRWdelta11	YCRWdelta11	320	457	0.70	-0.52	-0.57	-1.38
YIL111W	COX5B	1015	1452	0.70	-0.52	-0.57	-1.38
YML116W	ATR1	2060	2948	0.70	-0.52	-0.57	-1.38
YIR016W		1738	2487	0.70	-0.52	-0.57	-1.38
YLL020C		2351	3366	0.70	-0.52	-0.57	-1.39
YIR020W-B	YIR020W-B	1319	1888	0.70	-0.52	-0.57	-1.39
YPL168W		861	1233	0.70	-0.52	-0.57	-1.39
YMR258C		639	916	0.70	-0.52	-0.57	-1.39
YML017W	PSP2	1368	1962	0.70	-0.52	-0.58	-1.39
YGR114C		2479	3555	0.70	-0.52	-0.58	-1.39
YLR112W		1349	1936	0.70	-0.52	-0.58	-1.39
YNR010W	CSE2	773	1109	0.70	-0.52	-0.58	-1.40
YBR012C		621	892	0.70	-0.52	-0.58	-1.40
YJL147C		610	877	0.70	-0.52	-0.58	-1.40
YNR006W	VPS27	2600	3736	0.70	-0.52	-0.58	-1.40
YDR277C	MTH1	2795	4021	0.70	-0.52	-0.58	-1.40
YOL010W		2739	3942	0.69	-0.53	-0.58	-1.40
YPL120W	VPS30	1119	1611	0.69	-0.53	-0.58	-1.41
YBR007C		869	1252	0.69	-0.53	-0.58	-1.41
YBR292C		11589	16690	0.69	-0.53	-0.58	-1.41
YHRCTy1-1C	YHRCTy1-1C	7788	11219	0.69	-0.53	-0.58	-1.41
YDR261C	EXG2	3841	5534	0.69	-0.53	-0.58	-1.41
YDL114W		3497	5041	0.69	-0.53	-0.58	-1.41
YHR195W	VAB36	892	1287	0.69	-0.53	-0.58	-1.41
YJR087W		2048	2953	0.69	-0.53	-0.58	-1.41
YBL089W		1290	1860	0.69	-0.53	-0.58	-1.41
YGL007W		886	1279	0.69	-0.53	-0.59	-1.41
YOL075C		3169	4573	0.69	-0.53	-0.59	-1.41
YPL076W	GPI2	662	956	0.69	-0.53	-0.59	-1.41
YER009W	NTF2	1129	1629	0.69	-0.53	-0.59	-1.41
YLL014W		636	918	0.69	-0.53	-0.59	-1.42
YNR023W	SNF12	2899	4187	0.69	-0.53	-0.59	-1.42
YGL073W	HSF1	1820	2629	0.69	-0.53	-0.59	-1.42
YPR017C	DSS4	662	957	0.69	-0.53	-0.59	-1.42

YHR194W		5075	7338	0.69	-0.53	-0.59	-1.42
YPL132W	COX11	464	671	0.69	-0.53	-0.59	-1.42
YLR338W		1193	1726	0.69	-0.53	-0.59	-1.42
YLR404W		1646	2383	0.69	-0.53	-0.59	-1.43
YJL216C		978	1417	0.69	-0.53	-0.59	-1.43
YDL029W	ARP2	1701	2464	0.69	-0.53	-0.59	-1.43
YLR230W		4222	6121	0.69	-0.54	-0.59	-1.43
YJL027C		500	725	0.69	-0.54	-0.59	-1.43
YHR112C		1676	2430	0.69	-0.54	-0.59	-1.43
YJR101W		727	1055	0.69	-0.54	-0.59	-1.44
YHL042W		522	758	0.69	-0.54	-0.59	-1.44
YDR004W	RAD57	332	482	0.69	-0.54	-0.59	-1.44
YPL236C		679	987	0.69	-0.54	-0.60	-1.44
YIL055C		1814	2636	0.69	-0.54	-0.60	-1.44
YDR227W	SIR4	1101	1600	0.69	-0.54	-0.60	-1.44
YMR130W		759	1104	0.69	-0.54	-0.60	-1.44
YNL151C	RPC31	2893	4207	0.69	-0.54	-0.60	-1.44
YBL047C		1629	2371	0.69	-0.54	-0.60	-1.44
YGR063C	SPT4	2900	4223	0.69	-0.54	-0.60	-1.44
YLR084C	RAX2	1682	2450	0.69	-0.54	-0.60	-1.45
YOL142W	RRP40	930	1355	0.69	-0.54	-0.60	-1.45
YJR082C		1573	2293	0.69	-0.54	-0.60	-1.45
YDR135C	YCF1	9308	13574	0.69	-0.54	-0.60	-1.45
YOR210W	RPB10	1871	2731	0.69	-0.55	-0.60	-1.45
YJLWdelta15	YJLWdelta15	4414	6453	0.68	-0.55	-0.60	-1.46
YDR243C	PRP28	1040	1521	0.68	-0.55	-0.60	-1.46
YDR228C	PCF11	3507	5136	0.68	-0.55	-0.61	-1.47
YGL122C	NAB2	2233	3271	0.68	-0.55	-0.61	-1.47
YPL200W		1103	1616	0.68	-0.55	-0.61	-1.47
YDL016C		1259	1845	0.68	-0.55	-0.61	-1.47
YGR070W	ROM1	865	1267	0.68	-0.55	-0.61	-1.47
YGR060W	ERG25	2342	3432	0.68	-0.55	-0.61	-1.47
YIL060W		748	1097	0.68	-0.55	-0.61	-1.47
YPL001W	HAT1	1465	2148	0.68	-0.55	-0.61	-1.47
YIL001W		2281	3345	0.68	-0.55	-0.61	-1.47
YDR374C		4938	7243	0.68	-0.55	-0.61	-1.47

YGR223C		3371	4947	0.68	-0.55	-0.61	-1.47
YGLCdelta5	YGLCdelta5	402	590	0.68	-0.55	-0.61	-1.47
YGR184C	UBR1	4297	6309	0.68	-0.55	-0.61	-1.47
YDR292C	SRP101	1874	2754	0.68	-0.56	-0.61	-1.48
YBR276C	PPS1	9801	14411	0.68	-0.56	-0.61	-1.48
YIR001C	SGN1	1206	1775	0.68	-0.56	-0.61	-1.48
YPL040C	ISM1	3740	5507	0.68	-0.56	-0.61	-1.48
YNR071C		4125	6077	0.68	-0.56	-0.61	-1.49
YER045C		983	1449	0.68	-0.56	-0.62	-1.49
YDR196C		3883	5724	0.68	-0.56	-0.62	-1.49
YNR027W		2286	3370	0.68	-0.56	-0.62	-1.49
YGR093W		1600	2359	0.68	-0.56	-0.62	-1.49
YMR138W	CIN4	1671	2465	0.68	-0.56	-0.62	-1.49
YJL160C		1470	2169	0.68	-0.56	-0.62	-1.49
YOR177C		1052	1553	0.68	-0.56	-0.62	-1.49
YCL056C		1764	2604	0.68	-0.56	-0.62	-1.49
YPL114W		1850	2731	0.68	-0.56	-0.62	-1.49
YCL039W		5036	7437	0.68	-0.56	-0.62	-1.49
YLL030C		1791	2645	0.68	-0.56	-0.62	-1.49
YJR004C	SAG1	2322	3433	0.68	-0.56	-0.62	-1.50
YDR485C		3424	5062	0.68	-0.56	-0.62	-1.50
YHR143W		1303	1926	0.68	-0.56	-0.62	-1.50
YLR080W		1721	2549	0.68	-0.57	-0.62	-1.50
YBR165W	UBS1	1323	1961	0.67	-0.57	-0.62	-1.51
YGR174C	CBP4	707	1048	0.67	-0.57	-0.62	-1.51
YLR432W		5979	8863	0.67	-0.57	-0.62	-1.51
YIL017W		5399	8012	0.67	-0.57	-0.63	-1.51
YDL065C	PEX19	2498	3708	0.67	-0.57	-0.63	-1.51
YNL152W		987	1465	0.67	-0.57	-0.63	-1.51
YML132W	COS3	4971	7379	0.67	-0.57	-0.63	-1.51
YDR249C		647	960	0.67	-0.57	-0.63	-1.51
YIL079C		605	899	0.67	-0.57	-0.63	-1.51
YFR012W		811	1205	0.67	-0.57	-0.63	-1.51
YIR013C	GAT4	1774	2635	0.67	-0.57	-0.63	-1.51
YDR525W-A	YDR525W-A	477	709	0.67	-0.57	-0.63	-1.52
YBR264C	YPT10	2166	3221	0.67	-0.57	-0.63	-1.52

YPL087W	YDC1	346	515	0.67	-0.57	-0.63	-1.52
YCR096C	A2	2642	3934	0.67	-0.57	-0.63	-1.52
YGR162W	TIF4631	2114	3148	0.67	-0.57	-0.63	-1.52
YMR185W		1445	2151	0.67	-0.57	-0.63	-1.52
YGR129W	SYF2	949	1414	0.67	-0.57	-0.63	-1.52
YJR035W	RAD26	524	781	0.67	-0.57	-0.63	-1.52
YHRCdelta6	YHRCdelta6	642	956	0.67	-0.58	-0.63	-1.52
YDR181C	SAS4	1447	2157	0.67	-0.58	-0.63	-1.53
YLL060C	GTT2	423	630	0.67	-0.58	-0.63	-1.53
YBR210W		716	1067	0.67	-0.58	-0.63	-1.53
YDR361C		3889	5806	0.67	-0.58	-0.63	-1.53
YHR098C	SFB3	3871	5787	0.67	-0.58	-0.64	-1.54
YJR116W		1003	1499	0.67	-0.58	-0.64	-1.54
YDR009W	GAL3	621	929	0.67	-0.58	-0.64	-1.54
YOR352W		636	951	0.67	-0.58	-0.64	-1.54
YFR031C	SMC2	5724	8578	0.67	-0.58	-0.64	-1.55
YNL075W	IMP4	865	1296	0.67	-0.58	-0.64	-1.55
YER093C		2750	4123	0.67	-0.58	-0.64	-1.55
YHR033W		872	1309	0.67	-0.59	-0.64	-1.55
YDL062W		1201	1807	0.66	-0.59	-0.65	-1.56
YMR287C	MSU1	3033	4565	0.66	-0.59	-0.65	-1.56
YIL064W		2310	3480	0.66	-0.59	-0.65	-1.56
YLR265C		688	1038	0.66	-0.59	-0.65	-1.57
YCR067C	SED4	2090	3157	0.66	-0.60	-0.65	-1.57
YCR003W	MRPL32	1684	2550	0.66	-0.60	-0.65	-1.58
YIR020C		1987	3011	0.66	-0.60	-0.66	-1.58
YHR157W	REC104	668	1013	0.66	-0.60	-0.66	-1.58
YGL080W		1839	2788	0.66	-0.60	-0.66	-1.58
YJL045W		843	1278	0.66	-0.60	-0.66	-1.59
YAR033W		2791	4234	0.66	-0.60	-0.66	-1.59
YBR137W		1947	2955	0.66	-0.60	-0.66	-1.59
YPL144W	SNR17B	878	1333	0.66	-0.60	-0.66	-1.59
YGR035C		1006	1530	0.66	-0.60	-0.66	-1.60
YDL061C	RPS29B	2443	3718	0.66	-0.61	-0.66	-1.60
YAL011W		1972	3002	0.66	-0.61	-0.66	-1.60
YDL139C		1440	2193	0.66	-0.61	-0.66	-1.60

YIL100W		2206	3363	0.66	-0.61	-0.66	-1.60
YPRWdelta14	YPRWdelta14	880	1342	0.66	-0.61	-0.66	-1.60
YPL156C		683	1042	0.66	-0.61	-0.67	-1.61
YCL001W	RER1	3508	5355	0.66	-0.61	-0.67	-1.61
YNR074C		528	806	0.65	-0.61	-0.67	-1.61
YJL017W		880	1345	0.65	-0.61	-0.67	-1.61
YLL034C		3688	5647	0.65	-0.61	-0.67	-1.62
YJR098C		1880	2884	0.65	-0.62	-0.67	-1.63
YEL006W		1119	1717	0.65	-0.62	-0.67	-1.63
YCR034W	FEN1	1309	2009	0.65	-0.62	-0.67	-1.63
YGL070C	RPB9	3880	5959	0.65	-0.62	-0.68	-1.63
YIL032C		1222	1877	0.65	-0.62	-0.68	-1.63
YERWdelta13	YERWdelta13	1038	1595	0.65	-0.62	-0.68	-1.63
YML105C	SEC65	2280	3505	0.65	-0.62	-0.68	-1.63
YFR019W	FAB1	2775	4268	0.65	-0.62	-0.68	-1.64
YML098W	TAF19	637	980	0.65	-0.62	-0.68	-1.64
YOR071C		1064	1638	0.65	-0.62	-0.68	-1.64
YDR533C		2232	3437	0.65	-0.62	-0.68	-1.64
YMLCdelta1	YMLCdelta1	2871	4424	0.65	-0.62	-0.68	-1.64
YER018C	SPC25	515	794	0.65	-0.63	-0.68	-1.65
YDR169C	STB3	3503	5406	0.65	-0.63	-0.68	-1.65
YKL097C		677	1045	0.65	-0.63	-0.68	-1.65
YBR022W		602	930	0.65	-0.63	-0.68	-1.65
YDR500C	RPL37B	1327	2052	0.65	-0.63	-0.68	-1.65
YML045W		18016	27862	0.65	-0.63	-0.69	-1.65
YHR179W	OYE2	1078	1667	0.65	-0.63	-0.69	-1.66
YPL185W		4700	7274	0.65	-0.63	-0.69	-1.66
YER032W	FIR1	1845	2860	0.65	-0.63	-0.69	-1.66
YCL021W		3435	5332	0.64	-0.63	-0.69	-1.67
YPL221W	BOP1	2057	3195	0.64	-0.64	-0.69	-1.67
YIL049W	DFG10	3398	5279	0.64	-0.64	-0.69	-1.67
YOR325W		661	1028	0.64	-0.64	-0.69	-1.67
YDR165W		6371	9918	0.64	-0.64	-0.69	-1.68
YLRWdelta4	YLRWdelta4	384	598	0.64	-0.64	-0.70	-1.68
YLRCtau1	YLRCtau1	889	1385	0.64	-0.64	-0.70	-1.68
YKL019W	RAM2	2469	3850	0.64	-0.64	-0.70	-1.68

YPL102C		1463	2282	0.64	-0.64	-0.70	-1.69
YLR013W	GAT3	375	586	0.64	-0.64	-0.70	-1.69
YGL134W	PCL10	4254	6650	0.64	-0.64	-0.70	-1.69
YNL217W		969	1515	0.64	-0.65	-0.70	-1.69
YLR072W		2002	3131	0.64	-0.65	-0.70	-1.69
YDR325W	YCG1	1652	2587	0.64	-0.65	-0.70	-1.70
YGRCTy1-2D	YGRCTy1-2D	14715	23053	0.64	-0.65	-0.70	-1.70
YGL096W		1621	2541	0.64	-0.65	-0.70	-1.70
YPR121W	THI22	309	484	0.64	-0.65	-0.70	-1.70
YGL090W	LIF1	2525	3959	0.64	-0.65	-0.71	-1.70
YNL269W		1853	2906	0.64	-0.65	-0.71	-1.70
YLR127C	APC2	2262	3551	0.64	-0.65	-0.71	-1.71
YEL003W	GIM4	928	1458	0.64	-0.65	-0.71	-1.71
YNR014W		386	608	0.64	-0.65	-0.71	-1.72
YKL063C		647	1019	0.63	-0.66	-0.71	-1.72
YLR032W	RAD5	2337	3687	0.63	-0.66	-0.71	-1.72
YER106W		369	583	0.63	-0.66	-0.71	-1.73
YHR092C	HXT4	808	1275	0.63	-0.66	-0.71	-1.73
YCR080W		1759	2778	0.63	-0.66	-0.72	-1.73
YNR005C		551	870	0.63	-0.66	-0.72	-1.73
YPL157W		1543	2438	0.63	-0.66	-0.72	-1.73
YOL101C		994	1572	0.63	-0.66	-0.72	-1.73
YCR031C	RPS14A	2496	3948	0.63	-0.66	-0.72	-1.73
YGL131C		1856	2937	0.63	-0.66	-0.72	-1.73
YJL011C		389	615	0.63	-0.66	-0.72	-1.73
YIL006W		3317	5251	0.63	-0.66	-0.72	-1.74
YDR211W	GCD6	1429	2263	0.63	-0.66	-0.72	-1.74
YDR278C		1537	2436	0.63	-0.66	-0.72	-1.74
YGR189C	CRH1	2520	3997	0.63	-0.67	-0.72	-1.74
YGR051C		937	1487	0.63	-0.67	-0.72	-1.74
YDR293C	SSD1	3519	5587	0.63	-0.67	-0.72	-1.75
YDR341C		1007	1602	0.63	-0.67	-0.73	-1.75
YDR245W	MNN10	1564	2488	0.63	-0.67	-0.73	-1.75
YJLCdelta6	YJLCdelta6	3519	5599	0.63	-0.67	-0.73	-1.75
YJRWtau3	YJRWtau3	4049	6446	0.63	-0.67	-0.73	-1.76
YER121W		427	681	0.63	-0.67	-0.73	-1.76

YIL002C	INP51	5026	8015	0.63	-0.67	-0.73	-1.76
YLR341W		553	883	0.63	-0.67	-0.73	-1.76
YGL074C		2711	4329	0.63	-0.68	-0.73	-1.77
YIL016W	SNL1	754	1205	0.63	-0.68	-0.73	-1.77
YPL123C	RNY1	644	1030	0.63	-0.68	-0.73	-1.77
YDR215C		1849	2957	0.63	-0.68	-0.73	-1.77
YHR086W	NAM8	724	1161	0.62	-0.68	-0.74	-1.78
YNR002C	FUN34	770	1235	0.62	-0.68	-0.74	-1.78
YJR135C	MCM22	766	1230	0.62	-0.68	-0.74	-1.78
YGR050C		855	1375	0.62	-0.69	-0.74	-1.79
YPR083W		2360	3797	0.62	-0.69	-0.74	-1.79
YPL201C		1763	2843	0.62	-0.69	-0.75	-1.80
YJL015C		488	787	0.62	-0.69	-0.75	-1.80
YOL126C	MDH2	1294	2089	0.62	-0.69	-0.75	-1.80
YER135C		1566	2530	0.62	-0.69	-0.75	-1.81
YPL205C		2837	4585	0.62	-0.69	-0.75	-1.81
YLR115W	CFT2	2813	4549	0.62	-0.69	-0.75	-1.81
YHR017W	YSC83	2747	4444	0.62	-0.69	-0.75	-1.81
YGR097W	ASK10	1348	2181	0.62	-0.69	-0.75	-1.81
YPL173W	MRPL40	1171	1898	0.62	-0.70	-0.75	-1.82
YLR010C		305	495	0.62	-0.70	-0.75	-1.82
YBR148W	YSW1	3365	5463	0.62	-0.70	-0.76	-1.82
YLR096W	KIN2	1471	2390	0.62	-0.70	-0.76	-1.83
YML088W		4461	7252	0.62	-0.70	-0.76	-1.83
YJL165C	HAL5	835	1361	0.61	-0.70	-0.76	-1.84
YCL002C		931	1520	0.61	-0.71	-0.76	-1.84
YDR212W	TCP1	5185	8467	0.61	-0.71	-0.76	-1.84
YPR094W		1436	2346	0.61	-0.71	-0.76	-1.85
YBL094C		667	1090	0.61	-0.71	-0.76	-1.85
YHR068W	DYS1	3723	6086	0.61	-0.71	-0.77	-1.85
YNL231C	PDR16	484	792	0.61	-0.71	-0.77	-1.85
YGR066C		636	1040	0.61	-0.71	-0.77	-1.85
YJR114W		1263	2068	0.61	-0.71	-0.77	-1.85
YOL127W	RPL25	1046	1715	0.61	-0.71	-0.77	-1.86
YGL211W		693	1137	0.61	-0.72	-0.77	-1.86
YIL146C	ECM37	2690	4423	0.61	-0.72	-0.77	-1.87

YGL154C	LYS5	982	1616	0.61	-0.72	-0.77	-1.87
YEL007W		3533	5821	0.61	-0.72	-0.78	-1.87
YJR028W		2757	4554	0.61	-0.72	-0.78	-1.88
YJL135W		741	1226	0.60	-0.73	-0.78	-1.89
YIL065C		5490	9083	0.60	-0.73	-0.78	-1.89
YKR051W		317	526	0.60	-0.73	-0.79	-1.90
YDR170W-A	YDR170W-A	20078	33298	0.60	-0.73	-0.79	-1.90
YBL074C	AAR2	1142	1896	0.60	-0.73	-0.79	-1.90
YDR297W	SUR2	3220	5348	0.60	-0.73	-0.79	-1.90
YMR003W		1210	2010	0.60	-0.73	-0.79	-1.90
YML002W		1809	3008	0.60	-0.73	-0.79	-1.91
YOL157C		448	745	0.60	-0.73	-0.79	-1.91
YDR283C	GCN2	1501	2497	0.60	-0.73	-0.79	-1.91
YKL146W		568	946	0.60	-0.74	-0.79	-1.91
YMR304C-A		335	559	0.60	-0.74	-0.79	-1.92
YEL017W		1126	1878	0.60	-0.74	-0.79	-1.92
YGL141W		1474	2458	0.60	-0.74	-0.79	-1.92
YIL013C	PDR11	765	1277	0.60	-0.74	-0.79	-1.92
YPL167C	REV3	1078	1799	0.60	-0.74	-0.80	-1.92
YBR296C	PHO89	1799	3004	0.60	-0.74	-0.80	-1.92
YBR275C	RIF1	1361	2273	0.60	-0.74	-0.80	-1.92
YOR278W	HEM4	1342	2243	0.60	-0.74	-0.80	-1.93
YLR051C		1161	1943	0.60	-0.74	-0.80	-1.93
YMR274C	RCE1	662	1114	0.59	-0.75	-0.81	-1.95
YBR041W	FAT1	2419	4070	0.59	-0.75	-0.81	-1.95
YMR107W		1578	2655	0.59	-0.75	-0.81	-1.95
YGL262W		2706	4555	0.59	-0.75	-0.81	-1.95
YNR025C		414	699	0.59	-0.76	-0.81	-1.96
YCR099C		1987	3357	0.59	-0.76	-0.81	-1.96
YKL192C	ACP1	1585	2678	0.59	-0.76	-0.81	-1.96
YMR099C		632	1068	0.59	-0.76	-0.81	-1.96
YNR070W		1211	2046	0.59	-0.76	-0.81	-1.96
YOR227W		1865	3161	0.59	-0.76	-0.82	-1.97
YAR010C		18436	31279	0.59	-0.76	-0.82	-1.98
YGL167C	PMR1	2602	4417	0.59	-0.76	-0.82	-1.98
YMR046C		9859	16740	0.59	-0.76	-0.82	-1.98

YDR316W		1592	2704	0.59	-0.76	-0.82	-1.98
YOL159C		716	1216	0.59	-0.76	-0.82	-1.98
YHR059W		1424	2421	0.59	-0.77	-0.82	-1.98
YGL180W	APG1	1162	1977	0.59	-0.77	-0.82	-1.99
YPL062W		748	1274	0.59	-0.77	-0.82	-1.99
YHRCdelta8	YHRCdelta8	1972	3359	0.59	-0.77	-0.82	-1.99
YHRCTy1-1D	YHRCTy1-1D	3620	6166	0.59	-0.77	-0.82	-1.99
YLR287C		1183	2018	0.59	-0.77	-0.83	-2.00
YBR280C		1392	2383	0.58	-0.78	-0.83	-2.01
YFL030W		466	798	0.58	-0.78	-0.83	-2.01
YPRCdelta15	YPRCdelta15	379	649	0.58	-0.78	-0.83	-2.01
YKR062W	TFA2	1638	2807	0.58	-0.78	-0.83	-2.01
YKL159C	RCN1	1389	2380	0.58	-0.78	-0.83	-2.01
YMR184W		687	1179	0.58	-0.78	-0.84	-2.02
YAR040C		1050	1804	0.58	-0.78	-0.84	-2.02
YPL137C		5770	9915	0.58	-0.78	-0.84	-2.02
YJL004C	SYS1	1307	2247	0.58	-0.78	-0.84	-2.02
YIL084C	SDS3	1160	1996	0.58	-0.78	-0.84	-2.03
YIL029C		379	653	0.58	-0.78	-0.84	-2.03
YDR422C	SIP1	6328	10916	0.58	-0.79	-0.84	-2.04
YOL047C		487	840	0.58	-0.79	-0.84	-2.04
YDL141W	BPL1	1291	2229	0.58	-0.79	-0.84	-2.04
YIL095W	PRK1	1658	2868	0.58	-0.79	-0.85	-2.05
YGR043C		426	738	0.58	-0.79	-0.85	-2.05
YGR290W		403	700	0.58	-0.80	-0.85	-2.06
YMR173W	DDR48	951	1656	0.57	-0.80	-0.86	-2.07
YKL173W	SNU114	2262	3951	0.57	-0.80	-0.86	-2.08
YBRWdelta16	YBRWdelta16	394	688	0.57	-0.81	-0.86	-2.08
YMR155W		1104	1934	0.57	-0.81	-0.86	-2.09
YGR243W		435	764	0.57	-0.81	-0.87	-2.10
YML060W	OGG1	3249	5715	0.57	-0.81	-0.87	-2.10
YGR161C		1287	2273	0.57	-0.82	-0.88	-2.12
YIL028W		431	762	0.57	-0.82	-0.88	-2.12
YDR199W		1797	3178	0.57	-0.82	-0.88	-2.12
YER001W	MNN1	1331	2356	0.56	-0.82	-0.88	-2.13
YLRWdelta15	YLRWdelta15	378	669	0.56	-0.82	-0.88	-2.13

YER080W		952	1688	0.56	-0.83	-0.88	-2.13
YDR545W	YRF1-1	704	1250	0.56	-0.83	-0.89	-2.14
YML050W		454	808	0.56	-0.83	-0.89	-2.14
YGL150C	INO80	3981	7103	0.56	-0.84	-0.89	-2.15
YMR106C	YKU80	1246	2230	0.56	-0.84	-0.90	-2.16
YBR271W		649	1166	0.56	-0.85	-0.90	-2.18
YKR019C	IRS4	966	1738	0.56	-0.85	-0.90	-2.18
YILCdelta3	YILCdelta3	576	1038	0.56	-0.85	-0.90	-2.19
YBR268W	MRPL37	372	672	0.55	-0.85	-0.91	-2.20
YPL054W	LEE1	433	784	0.55	-0.85	-0.91	-2.20
YKR094C	RPL40B	1156	2092	0.55	-0.86	-0.91	-2.20
YMR078C	CTF18	1340	2431	0.55	-0.86	-0.92	-2.21
YNR059W	MNT4	1532	2780	0.55	-0.86	-0.92	-2.21
YGR205W		710	1292	0.55	-0.86	-0.92	-2.22
YBR258C		499	909	0.55	-0.87	-0.92	-2.23
YEL070W		440	801	0.55	-0.87	-0.92	-2.23
YGL086W	MAD1	4194	7669	0.55	-0.87	-0.93	-2.24
YPL026C	SKS1	1896	3485	0.54	-0.88	-0.93	-2.26
YNR075W	COS10	1537	2832	0.54	-0.88	-0.94	-2.27
YNR044W	AGA1	1959	3616	0.54	-0.88	-0.94	-2.27
YDR179W-A		702	1297	0.54	-0.89	-0.94	-2.27
YOL107W		304	563	0.54	-0.89	-0.95	-2.28
YPR200C	ARR2	366	679	0.54	-0.89	-0.95	-2.29
YEL022W	GEA2	2440	4555	0.54	-0.90	-0.96	-2.31
YORWdelta22	YORWdelta22	793	1482	0.54	-0.90	-0.96	-2.31
YHL030W	ECM29	450	843	0.53	-0.90	-0.96	-2.32
YPL189W		826	1549	0.53	-0.91	-0.96	-2.33
YDL210W	UGA4	1827	3441	0.53	-0.91	-0.97	-2.34
YOR134W	BAG7	442	837	0.53	-0.92	-0.98	-2.36
YNL232W	CSL4	1876	3559	0.53	-0.92	-0.98	-2.37
YHR162W		2703	5149	0.52	-0.93	-0.99	-2.38
YMR180C	CTL1	769	1468	0.52	-0.93	-0.99	-2.39
YOR182C	RPS30B	653	1247	0.52	-0.93	-0.99	-2.39
YPL021W	ECM23	512	980	0.52	-0.94	-0.99	-2.40
YDR107C		638	1223	0.52	-0.94	-0.99	-2.40
YML115C	VAN1	1176	2258	0.52	-0.94	-1.00	-2.41

YBL005W-A		21298	40946	0.52	-0.94	-1.00	-2.41
YJL041W	NSP1	834	1607	0.52	-0.95	-1.00	-2.42
YGL118C		4106	7919	0.52	-0.95	-1.00	-2.42
YJR023C		1363	2631	0.52	-0.95	-1.00	-2.43
YML089C		684	1321	0.52	-0.95	-1.01	-2.43
YBR231C		433	840	0.52	-0.95	-1.01	-2.44
YFL029C	CAK1	1006	1953	0.52	-0.96	-1.01	-2.45
YOR226C	ISU2	386	752	0.51	-0.96	-1.02	-2.46
YMR154C	RIM13	889	1752	0.51	-0.98	-1.04	-2.50
YGR157W	CHO2	866	1738	0.50	-1.01	-1.06	-2.56
YEL064C		1099	2208	0.50	-1.01	-1.06	-2.56
YGR209C	TRX2	327	665	0.49	-1.02	-1.08	-2.61
YJLWtau4	YJLWtau4	1821	3714	0.49	-1.03	-1.08	-2.62
YGRCTy1-2B	YGRCTy1-2B	5477	11200	0.49	-1.03	-1.09	-2.63
YBR216C		2068	4241	0.49	-1.04	-1.09	-2.64
YLR081W	GAL2	748	1538	0.49	-1.04	-1.10	-2.65
YGR221C		1224	2522	0.49	-1.04	-1.10	-2.65
YDL077C	VAM6	1719	3552	0.48	-1.05	-1.10	-2.66
YMR125W	STO1	1618	3368	0.48	-1.06	-1.11	-2.69
YML079W		656	1386	0.47	-1.08	-1.13	-2.74
YDR089W		1076	2293	0.47	-1.09	-1.15	-2.77
YLR128W		1181	2519	0.47	-1.09	-1.15	-2.78
YGR237C		644	1376	0.47	-1.09	-1.15	-2.78
YDR452W		1624	3483	0.47	-1.10	-1.16	-2.79
YML102C-A		2418	5314	0.46	-1.14	-1.19	-2.88
YMR210W		329	740	0.45	-1.17	-1.22	-2.96
YGL024W		1141	2563	0.45	-1.17	-1.22	-2.96
YMR160W		1236	2822	0.44	-1.19	-1.25	-3.01
YGL092W	NUP145	928	2125	0.44	-1.20	-1.25	-3.02
YDR147W	EKI1	3904	8988	0.43	-1.20	-1.26	-3.04
YJL103C		356	826	0.43	-1.21	-1.27	-3.07
YDR505C	PSP1	1067	2476	0.43	-1.21	-1.27	-3.07
YDR324C		1390	3230	0.43	-1.22	-1.27	-3.07
YGL183C		432	1005	0.43	-1.22	-1.27	-3.07
YML063W	RPS1B	1442	3396	0.42	-1.24	-1.29	-3.12
YDR520C		972	2316	0.42	-1.25	-1.31	-3.16

YER091C	MET6	1601	4003	0.40	-1.32	-1.38	-3.33
YNR058W	BIO3	633	1590	0.40	-1.33	-1.38	-3.34
YPL135W	ISU1	665	1674	0.40	-1.33	-1.39	-3.35
YCR035C	RRP43	408	1118	0.36	-1.45	-1.51	-3.65
YPR015C		329	915	0.36	-1.47	-1.53	-3.70
YKR050W	TRK2	838	2357	0.36	-1.49	-1.55	-3.74
YLR172C	DPH5	549	1605	0.34	-1.55	-1.61	-3.88
YPL250C		521	1546	0.34	-1.57	-1.63	-3.93
