# Supplementary material for: Genome-scale approaches for discovering novel nonconventional splicing substrates of the Ire1 nuclease
Source: Genome Biol. 2004 Dec 22;6(1):R3. doi: 10.1186/gb-2004-6-1-r3 (PMC549064; doi:10.1186/gb-2004-6-1-r3)
Supplement: Additional data file 2 — Supplementary Table 2, which lists the complete output of the computational screen [file gb-2004-6-1-r3-s2.pdf]

Supplimentary Table 2

ORF	5'	3'	5' stem	loop	3' stem
YBR140C	10126	10142	TTCT	TCTGATGAG	AGAA
YBR284W	1970	1986	AAGC	CCCGACGGG	GCTT
YDL115C	832	848	TTTC	TCCGGTGTG	GAAA
YDR279W	-596	-580	ACAT	CCAGTTGTG	ATGT
YDR323C	1593	1609	TTGG	TCCGCGGAG	CCAA
YDR477W	-888	-872	TTTG	TCTGGAGGG	CAAA
YER007W	1178	1194	CCAA	TCTGCCGCG	TTGG
YER103W	1263	1279	AAAA	TCGGAAGTG	TTTT
YER147C	1724	1740	GCCG	CCAGCAGGG	CGGC
YFL031W	654	670	TAAT	CCAGCCGTG	ATTA
YFL031W	906	922	ACTG	TCCGAAGCG	CAGT
YFL061W	48	64	CATT	CCAGTGGTG	AATG
YGL014W	3304	3320	AACT	CCGGTAGTG	AGTT
YGL124C	2614	2630	TTTC	TCTGCCGGG	GAAA
YGL197W	3879	3895	AGAT	TCTGCTGAG	ATCT
YGR111W	283	299	GTTT	CCAGTTGCG	AAAC
YGR178C	2013	2029	GTAT	CCAGGAGGG	ATAC
YGR200C	352	368	TCAG	TCGGTTGTG	CTGA
YGR210C	-311	-295	TAGC	CCTGATGGG	GCTA
YGR250C	1019	1035	ACTT	TCCGAAGAG	AAGT
YHR037W	1167	1183	AATT	TCTGGTGGG	AATT
YHR092C	-566	-550	TCTT	TCTGTGGAG	AAGA
YIL036W	1086	1102	TACT	CCAGCGGAG	AGTA
YIR002C	1623	1639	GAGC	TCTGAAGAG	GCTC
YKL130C	1003	1019	CTCC	TCTGATGTG	GGAG
YKL157W	3478	3494	TTCC	TCCGATGTG	GGAA
YKR018C	1758	1774	AATG	TCTGAAGAG	CATT
YLR266C	944	960	TATG	CCAGGGGTG	CATA
YLR417W	2086	2102	AATT	CCTGTGGCG	AATT
YML093W	657	673	TGGC	TCAGGTGAG	GCCA

YML131W	793	809	ATAG	CCTGTGGTG	CTAT
YMR008C	1392	1408	TGTT	CCAGATGTG	AACA
YMR012W	-276	-260	ACTC	CCCGTAGCG	GAGT
YMR315W	1611	1627	ACGA	CCAGAAGTG	TCGT
YNL072W	627	643	GGAT	CCCGATGAG	ATCC
YNL115C	162	178	TGAA	TCCGATGGG	TTCA
YNL177C	1688	1704	AGTA	CCTGGAGCG	TACT
YNL221C	-650	-634	TTAC	TCGGTCGAG	GTAA
YNL236W	1512	1528	TCCA	TCGGTTGAG	TGGA
YNL248C	1338	1354	TCCT	CCTGATGAG	AGGA
YNL262W	-878	-862	TATT	CCAGATGAG	AATA
YNL335W	48	64	CATT	CCAGTGGTG	AATG
YOR154W	609	625	CAAT	CCAGAAGCG	ATTG
YOR176W	741	757	CTAC	CCAGCTGAG	GTAG
YOR209C	414	430	AATA	TCAGAAGCG	TATT
YOR249C	621	637	GATC	TCGGTGGAG	GATC
YOR315W	1492	1508	GCAA	TCAGCTGTG	TTGC
YPL179W	255	271	CATT	CCTGGTGAG	AATG
YPL270W	1160	1176	TTTT	TCAGGTGGG	AAAA
YPR118W	1198	1214	AATT	CCAGTGGTG	AATT
YPR128C	657	673	TTTG	TCAGCAGTG	CAAA
