# Supplementary material for: Genome-scale approaches for discovering novel nonconventional splicing substrates of the Ire1 nuclease
Source: Genome Biol. 2004 Dec 22;6(1):R3. doi: 10.1186/gb-2004-6-1-r3 (PMC549064; doi:10.1186/gb-2004-6-1-r3)
Supplement: Additional data file 3 — Supplementary Table 3 which lists all data displayed in Figure 5 [file gb-2004-6-1-r3-s3.pdf]

Niwa et al.

Supplementary Table 3

ORF ID	Name	X=Cy3	Y=Cy5	(X/Y)	log2(X/Y)	X/Y)-mean(log2(X/Y))	Nsigma
YFL031W	HAC1	40988	14536	2.82	1.50	1.38	5.03
YLR413W		8846	4207	2.10	1.07	0.96	3.49
YKR075C		4689	2305	2.03	1.02	0.91	3.32
YDR033W	MRH1	8157	4067	2.01	1.00	0.89	3.24
YDR345C	HXT3	1177	592	1.99	0.99	0.88	3.20
YDR146C	SWI5	4108	2117	1.94	0.96	0.84	3.07
YGR245C		1832	947	1.93	0.95	0.84	3.05
YOR154W		2979	1546	1.93	0.95	0.83	3.03
YHR159W		1614	841	1.92	0.94	0.83	3.01
YDL148C		5296	2771	1.91	0.93	0.82	2.99
YOR294W	RRS1	7955	4236	1.88	0.91	0.80	2.90
YOR293W	RPS10A	5966	3197	1.87	0.90	0.79	2.86
YPR002W	PDH1	1189	651	1.83	0.87	0.76	2.75
YPR112C	MRD1	8712	4872	1.79	0.84	0.73	2.64
YMR273C	ZDS1	3537	1978	1.79	0.84	0.73	2.64
YLL011W	SOF1	14004	7841	1.79	0.84	0.72	2.63
YCR057C	PWP2	4625	2602	1.78	0.83	0.72	2.61
YHR186C		1967	1112	1.77	0.82	0.71	2.58
YJL033W	HCA4	5599	3177	1.76	0.82	0.71	2.56
YIL019W		2757	1571	1.76	0.81	0.70	2.54
YKL078W		3530	2012	1.75	0.81	0.70	2.54
YMR032W	HOF1	3824	2183	1.75	0.81	0.70	2.53
YNL068C	FKH2	4923	2824	1.74	0.80	0.69	2.51
YGR103W		10282	5904	1.74	0.80	0.69	2.50
YNL198C		891	512	1.74	0.80	0.69	2.50
YNL062C	GCD10	8647	4974	1.74	0.80	0.69	2.49
YLR184W		544	313	1.74	0.80	0.69	2.49
YGL116W	CDC20	1151	664	1.73	0.79	0.68	2.48
YPR119W	CLB2	5969	3445	1.73	0.79	0.68	2.47

YMR230W	RPS10B	5181	2992	1.73	0.79	0.68	2.47
YNL182C		8142	4703	1.73	0.79	0.68	2.47
YML080W		6086	3524	1.73	0.79	0.68	2.46
YOR234C	RPL33B	21304	12359	1.72	0.79	0.67	2.45
YJL109C		4964	2889	1.72	0.78	0.67	2.43
YKL029C	MAE1	5677	3312	1.71	0.78	0.67	2.42
YCLX02C		5535	3232	1.71	0.78	0.66	2.41
YML034W	SRC1	1839	1074	1.71	0.78	0.66	2.41
YFL051C		530	310	1.71	0.77	0.66	2.41
YIL131C	FKH1	8729	5110	1.71	0.77	0.66	2.40
YPL155C	KIP2	7139	4204	1.70	0.76	0.65	2.37
YKR099W	BAS1	2171	1280	1.70	0.76	0.65	2.36
YIL091C		11608	6887	1.69	0.75	0.64	2.33
YDR465C	RMT2	11102	6597	1.68	0.75	0.64	2.32
YKL027W		3614	2152	1.68	0.75	0.64	2.31
YMR193C-A		1765	1051	1.68	0.75	0.64	2.31
YOR302W		3568	2125	1.68	0.75	0.64	2.31
YDR399W	HPT1	26517	15859	1.67	0.74	0.63	2.29
YDL055C	PSA1	18489	11066	1.67	0.74	0.63	2.28
YNR053C		15322	9228	1.66	0.73	0.62	2.25
YLR276C	DBP9	17308	10451	1.66	0.73	0.62	2.24
YOR272W	YTM1	10016	6049	1.66	0.73	0.62	2.24
YLR014C	PPR1	1854	1121	1.65	0.73	0.61	2.23
YCR055C		9098	5512	1.65	0.72	0.61	2.22
YCR006C		1322	806	1.64	0.71	0.60	2.19
YHR103W	SBE22	5649	3445	1.64	0.71	0.60	2.19
YBL039C	URA7	9636	5878	1.64	0.71	0.60	2.18
YOR146W		13527	8254	1.64	0.71	0.60	2.18
YER111C	SWI4	4436	2723	1.63	0.70	0.59	2.15
YJR089W	BIR1	1979	1216	1.63	0.70	0.59	2.15
YKR057W	RPS21A	17884	10991	1.63	0.70	0.59	2.15
YIL149C		3701	2277	1.63	0.70	0.59	2.14
YPL141C		2979	1837	1.62	0.70	0.58	2.13
YOR361C	PRT1	16420	10154	1.62	0.69	0.58	2.11
YFR053C	HXK1	10560	6539	1.61	0.69	0.58	2.11
YDR497C	ITR1	3911	2422	1.61	0.69	0.58	2.11

YJL010C		8513	5275	1.61	0.69	0.58	2.10
YLR222C		5754	3566	1.61	0.69	0.58	2.10
YJL098W	SAP185	6269	3890	1.61	0.69	0.58	2.09
YGL078C	DBP3	6180	3835	1.61	0.69	0.58	2.09
YPL143W	RPL33A	14577	9049	1.61	0.69	0.58	2.09
YHR091C	MSR1	618	384	1.61	0.69	0.57	2.09
YHR032W		474	295	1.61	0.69	0.57	2.08
YGR025W		1724	1073	1.61	0.68	0.57	2.08
YBR012W-A		10059	6288	1.60	0.68	0.57	2.06
YCL063W		4869	3049	1.60	0.68	0.56	2.05
YGR089W		3389	2122	1.60	0.68	0.56	2.05
YNL296W		548	345	1.59	0.67	0.56	2.02
YDR212W	TCP1	1300	819	1.59	0.67	0.55	2.02
YBR027C		1296	817	1.59	0.67	0.55	2.01
YGL170C		909	574	1.58	0.66	0.55	2.00
YKR094C	RPL40B	10707	6763	1.58	0.66	0.55	2.00
YKR081C		4761	3011	1.58	0.66	0.55	2.00
YDR060W		1381	874	1.58	0.66	0.55	1.99
YDR269C		744	471	1.58	0.66	0.55	1.99
YLR074C		9646	6116	1.58	0.66	0.54	1.98
YHR145C		2909	1845	1.58	0.66	0.54	1.98
YMR001C	CDC5	7106	4508	1.58	0.66	0.54	1.98
YMR185W		3761	2389	1.57	0.65	0.54	1.97
YHR179W	OYE2	4715	2999	1.57	0.65	0.54	1.96
YGR087C	PDC6	2596	1652	1.57	0.65	0.54	1.96
YGL076C	RPL7A	2488	1584	1.57	0.65	0.54	1.96
YLR086W	SMC4	1579	1006	1.57	0.65	0.54	1.96
YER054C	GIP2	1065	679	1.57	0.65	0.54	1.95
YDR343C	HXT6	1524	974	1.56	0.65	0.53	1.94
YDR449C		7733	4942	1.56	0.65	0.53	1.94
YCR058C		2038	1303	1.56	0.65	0.53	1.94
YHR182W		4174	2669	1.56	0.64	0.53	1.94
YNL114C		16750	10720	1.56	0.64	0.53	1.93
YNL226W		1290	826	1.56	0.64	0.53	1.93
YMR080C	NAM7	6931	4438	1.56	0.64	0.53	1.93
YBR092C	PHO3	15716	10065	1.56	0.64	0.53	1.93

YGR145W		5128	3286	1.56	0.64	0.53	1.93
YGL158W	RCK1	565	362	1.56	0.64	0.53	1.92
YPR136C		2470	1584	1.56	0.64	0.53	1.92
YGR159C	NSR1	17155	11005	1.56	0.64	0.53	1.92
YPR104C	FHL1	1589	1020	1.56	0.64	0.53	1.92
YOL124C		3532	2270	1.56	0.64	0.53	1.91
YGR108W	CLB1	8983	5773	1.56	0.64	0.53	1.91
YLR278C		1162	747	1.56	0.64	0.53	1.91
YDL242W		2392	1538	1.56	0.64	0.52	1.91
YDR158W	HOM2	13133	8446	1.55	0.64	0.52	1.91
YPL079W	RPL21B	14750	9487	1.55	0.64	0.52	1.91
YPL111W	CAR1	7322	4711	1.55	0.64	0.52	1.90
YBR190W		1546	995	1.55	0.64	0.52	1.90
YMR031W-A		503	324	1.55	0.63	0.52	1.90
YBR029C	CDS1	1784	1149	1.55	0.63	0.52	1.90
YOR381W	FRE3	2294	1478	1.55	0.63	0.52	1.90
YDR299W	BFR2	3228	2080	1.55	0.63	0.52	1.90
YPR142C		3446	2222	1.55	0.63	0.52	1.89
YCR056W		2122	1368	1.55	0.63	0.52	1.89
YCL012W		1028	663	1.55	0.63	0.52	1.89
YOR345C		4528	2921	1.55	0.63	0.52	1.89
YBR178W		1191	769	1.55	0.63	0.52	1.88
YPR090W		2418	1563	1.55	0.63	0.52	1.88
YDR164C	SEC1	3433	2219	1.55	0.63	0.52	1.88
YEL010W		447	289	1.55	0.63	0.52	1.88
YDR020C		13986	9048	1.55	0.63	0.52	1.88
YCR037C	PHO87	8272	5352	1.55	0.63	0.52	1.88
YPL126W	NAN1	13832	8962	1.54	0.63	0.51	1.87
YPL269W	KAR9	4152	2692	1.54	0.63	0.51	1.86
YOL078W		843	547	1.54	0.62	0.51	1.86
YLR397C	AFG2	4131	2681	1.54	0.62	0.51	1.86
YPL093W	NOG1	8026	5209	1.54	0.62	0.51	1.86
YGR079W		13839	8982	1.54	0.62	0.51	1.86
YGR128C		18601	12083	1.54	0.62	0.51	1.85
YBR266C		6440	4185	1.54	0.62	0.51	1.85
YJL061W	NUP82	4522	2939	1.54	0.62	0.51	1.85

YGL014W		3341	2172	1.54	0.62	0.51	1.85
YMR290C	HAS1	13822	8998	1.54	0.62	0.51	1.84
YGR059W	SPR3	562	366	1.54	0.62	0.51	1.84
YPL073C		1076	702	1.53	0.62	0.50	1.83
YIL031W	SMT4	2484	1622	1.53	0.61	0.50	1.83
YER155C	BEM2	473	309	1.53	0.61	0.50	1.82
YLR272C	LOC7	4375	2858	1.53	0.61	0.50	1.82
YCR092C	MSH3	2316	1513	1.53	0.61	0.50	1.82
YJL135W		577	377	1.53	0.61	0.50	1.82
YMR131C		12194	7977	1.53	0.61	0.50	1.82
YKL166C	TPK3	1139	745	1.53	0.61	0.50	1.82
YPL005W		880	576	1.53	0.61	0.50	1.82
YPR190C	RPC82	5937	3888	1.53	0.61	0.50	1.81
YAR040C		554	363	1.53	0.61	0.50	1.81
YER160C		15573	10213	1.52	0.61	0.50	1.80
YNL207W		5688	3733	1.52	0.61	0.50	1.80
YPR196W		965	634	1.52	0.61	0.49	1.80
YOR206W		11126	7314	1.52	0.61	0.49	1.79
YBL097W	BRN1	2913	1915	1.52	0.61	0.49	1.79
YJL209W	CBP1	4600	3025	1.52	0.60	0.49	1.79
YKL109W	HAP4	1926	1267	1.52	0.60	0.49	1.79
YPL261C		1333	877	1.52	0.60	0.49	1.79
YDR481C	PHO8	1665	1097	1.52	0.60	0.49	1.78
YDR406W	PDR15	2055	1354	1.52	0.60	0.49	1.78
YGR205W		5750	3799	1.51	0.60	0.49	1.77
YGL021W	ALK1	8111	5359	1.51	0.60	0.49	1.77
YPL183C		6969	4607	1.51	0.60	0.48	1.76
YKL182W	FAS1	2278	1506	1.51	0.60	0.48	1.76
YOL076W	MDM20	8262	5467	1.51	0.60	0.48	1.76
YJL051W		8011	5301	1.51	0.60	0.48	1.76
YHL015W	RPS20	39259	25999	1.51	0.59	0.48	1.75
YGL188C		8380	5552	1.51	0.59	0.48	1.75
YOL128C		4063	2694	1.51	0.59	0.48	1.75
YHL008C		1155	766	1.51	0.59	0.48	1.75
YLR357W	RSC2	7200	4781	1.51	0.59	0.48	1.74
YDR521W		1055	701	1.51	0.59	0.48	1.74

YMR216C	SKY1	2503	1663	1.51	0.59	0.48	1.74
YBR202W	CDC47	3057	2031	1.51	0.59	0.48	1.74
YNL126W	SPC98	3411	2267	1.50	0.59	0.48	1.73
YHR157W	REC104	3193	2123	1.50	0.59	0.48	1.73
YNL065W		1277	850	1.50	0.59	0.47	1.73
YJR128W		1403	934	1.50	0.59	0.47	1.72
YOR001W	RRP6	2306	1536	1.50	0.59	0.47	1.72
YJR140C	HIR3	2037	1357	1.50	0.59	0.47	1.72
YGL030W	RPL30	14697	9795	1.50	0.59	0.47	1.72
YML035C-A		1087	725	1.50	0.58	0.47	1.71
YOL160W		2842	1896	1.50	0.58	0.47	1.71
YNL141W	AAH1	22648	15112	1.50	0.58	0.47	1.71
YMR093W		12699	8475	1.50	0.58	0.47	1.71
YHR201C	PPX1	3126	2087	1.50	0.58	0.47	1.71
YAR047C		532	355	1.50	0.58	0.47	1.71
YML043C	RRN11	1974	1319	1.50	0.58	0.47	1.71
YDR080W	VPS41	2149	1439	1.49	0.58	0.47	1.70
YJR033C		452	303	1.49	0.58	0.47	1.70
YNL162W	RPL42A	19791	13264	1.49	0.58	0.46	1.69
YNL120C		10801	7239	1.49	0.58	0.46	1.69
YLR325C	RPL38	24038	16114	1.49	0.58	0.46	1.69
YPL029W	SUV3	3437	2304	1.49	0.58	0.46	1.69
YLR190W		5277	3538	1.49	0.58	0.46	1.69
YOL144W	NOP8	3497	2346	1.49	0.58	0.46	1.69
YCL054W	SPB1	4388	2945	1.49	0.58	0.46	1.68
YOR135C		7848	5273	1.49	0.57	0.46	1.68
YDR245W	MNN10	5423	3644	1.49	0.57	0.46	1.68
YHR059W		15002	10091	1.49	0.57	0.46	1.67
YEL065W	SIT1	16614	11176	1.49	0.57	0.46	1.67
YBR298C	MAL31	1880	1265	1.49	0.57	0.46	1.67
YIL148W	RPL40A	20476	13789	1.48	0.57	0.46	1.67
YHR079C	IRE1	2446	1647	1.48	0.57	0.46	1.67
YIL162W	SUC2	6353	4281	1.48	0.57	0.46	1.66
YDL117W		3431	2314	1.48	0.57	0.46	1.66
YLR313C	SPH1	5113	3448	1.48	0.57	0.46	1.66
YDL077C	VAM6	841	567	1.48	0.57	0.46	1.66

YDR450W	RPS18A	10545	7117	1.48	0.57	0.45	1.65
YIL047C	SYG1	5069	3423	1.48	0.57	0.45	1.65
YPL102C		2559	1729	1.48	0.57	0.45	1.65
YMR194W	RPL36A	2548	1722	1.48	0.57	0.45	1.65
YDL196W		503	340	1.48	0.57	0.45	1.65
YIL096C		5199	3515	1.48	0.56	0.45	1.64
YBR113W		2042	1381	1.48	0.56	0.45	1.64
YDR375C	BCS1	9438	6382	1.48	0.56	0.45	1.64
YCL061C		435	294	1.48	0.56	0.45	1.64
YNL228W		3701	2504	1.48	0.56	0.45	1.64
YLR401C		5975	4046	1.48	0.56	0.45	1.64
YJR145C	RPS4A	3394	2299	1.48	0.56	0.45	1.64
YPL193W	RSA1	5710	3876	1.47	0.56	0.45	1.62
YNL119W		18134	12314	1.47	0.56	0.45	1.62
YCL023C		540	367	1.47	0.56	0.44	1.62
YOL046C		987	671	1.47	0.56	0.44	1.62
YJR115W		1285	874	1.47	0.56	0.44	1.61
YER105C	NUP157	8246	5611	1.47	0.56	0.44	1.61
YFR057W		786	535	1.47	0.56	0.44	1.61
YPR156C		2005	1365	1.47	0.56	0.44	1.61
YDR331W	GPI8	6533	4447	1.47	0.55	0.44	1.61
YMR132C		3880	2641	1.47	0.55	0.44	1.61
YHL039W		18521	12610	1.47	0.55	0.44	1.61
YAL048C		1527	1040	1.47	0.55	0.44	1.61
YGR289C	MAL11	3035	2067	1.47	0.55	0.44	1.61
YKR079C		4869	3319	1.47	0.55	0.44	1.60
YNL087W		5999	4089	1.47	0.55	0.44	1.60
YFL032W		2368	1614	1.47	0.55	0.44	1.60
YGL236C	MTO1	7140	4868	1.47	0.55	0.44	1.60
YNL338W		524	357	1.47	0.55	0.44	1.60
YPL033C		625	426	1.47	0.55	0.44	1.60
YCL051W	LRE1	1131	771	1.47	0.55	0.44	1.60
YMR128W	ECM16	1300	887	1.47	0.55	0.44	1.60
YPL242C	IQG1	2299	1569	1.47	0.55	0.44	1.60
YPL012W		10577	7218	1.47	0.55	0.44	1.60
YBR172C	SMY2	4161	2840	1.47	0.55	0.44	1.60

YNL124W		6545	4468	1.46	0.55	0.44	1.59
YNL256W	FOL1	5316	3631	1.46	0.55	0.44	1.59
YGL128C		526	360	1.46	0.55	0.44	1.58
YPL243W	SRP68	19541	13366	1.46	0.55	0.44	1.58
YMR011W	HXT2	25881	17703	1.46	0.55	0.44	1.58
YGR265W		4257	2913	1.46	0.55	0.43	1.58
YDR447C	RPS17B	20113	13766	1.46	0.55	0.43	1.58
YCL014W	BUD3	3452	2363	1.46	0.55	0.43	1.58
YGL190C	CDC55	7040	4820	1.46	0.55	0.43	1.58
YDR195W	REF2	2734	1872	1.46	0.55	0.43	1.58
YNL186W	UBP10	16589	11361	1.46	0.55	0.43	1.58
YLR302C		1095	750	1.46	0.55	0.43	1.58
YPR022C		3934	2695	1.46	0.55	0.43	1.58
YHR140W		6325	4334	1.46	0.55	0.43	1.57
YJL217W		8775	6013	1.46	0.55	0.43	1.57
YMR224C	MRE11	541	371	1.46	0.55	0.43	1.57
YBR250W		1378	945	1.46	0.54	0.43	1.57
YOL166C		439	301	1.46	0.54	0.43	1.57
YMR031C		7789	5343	1.46	0.54	0.43	1.57
YJL087C	TRL1	3234	2219	1.46	0.54	0.43	1.57
YCL029C	BIK1	1805	1239	1.46	0.54	0.43	1.56
YJR072C		5137	3528	1.46	0.54	0.43	1.56
YMR049C		15407	10581	1.46	0.54	0.43	1.56
YDR443C	SSN2	1753	1204	1.46	0.54	0.43	1.56
YGL146C		700	481	1.46	0.54	0.43	1.56
YLR434C		2055	1412	1.46	0.54	0.43	1.56
YML006C	GIS4	2067	1421	1.45	0.54	0.43	1.56
YLR419W		1798	1236	1.45	0.54	0.43	1.56
YDR025W	RPS11A	11126	7652	1.45	0.54	0.43	1.56
YDL071C		919	632	1.45	0.54	0.43	1.55
YMR119W		3445	2370	1.45	0.54	0.43	1.55
YDR237W	MRPL7	1210	833	1.45	0.54	0.43	1.55
YPR018W	RLF2	3727	2566	1.45	0.54	0.43	1.55
YKL142W	MRP8	466	321	1.45	0.54	0.43	1.55
YML032C-A		1824	1256	1.45	0.54	0.43	1.55
YCL053C		9166	6313	1.45	0.54	0.43	1.55

YOR333C		1813	1249	1.45	0.54	0.42	1.55
YFR023W	PES4	425	293	1.45	0.54	0.42	1.54
YHR197W		830	572	1.45	0.54	0.42	1.54
YDL194W	SNF3	1095	755	1.45	0.54	0.42	1.54
YLR002C		13922	9600	1.45	0.54	0.42	1.54
YDR120C	TRM1	15731	10848	1.45	0.54	0.42	1.54
YMR163C		1099	758	1.45	0.54	0.42	1.54
YOR066W		1988	1371	1.45	0.54	0.42	1.54
YDR283C	GCN2	455	314	1.45	0.54	0.42	1.54
YGL094C	PAN2	1993	1375	1.45	0.54	0.42	1.54
YOR218C		2763	1906	1.45	0.54	0.42	1.54
YER164W	CHD1	5717	3946	1.45	0.53	0.42	1.54
YHR055C	CUP1-2	8966	6189	1.45	0.53	0.42	1.54
YGR125W		3765	2599	1.45	0.53	0.42	1.54
YMR144W		4982	3439	1.45	0.53	0.42	1.54
YIL115C	NUP159	1897	1310	1.45	0.53	0.42	1.53
YNL112W	DBP2	20098	13876	1.45	0.53	0.42	1.53
YJR158W	HXT16	1951	1347	1.45	0.53	0.42	1.53
YDL164C	CDC9	2960	2044	1.45	0.53	0.42	1.53
YHR189W		1835	1267	1.45	0.53	0.42	1.53
YMR259C		815	563	1.45	0.53	0.42	1.53
YMR177W	MMT1	2245	1552	1.45	0.53	0.42	1.53
YHR021C	RPS27B	12652	8754	1.45	0.53	0.42	1.52
YGR093W		5523	3824	1.44	0.53	0.42	1.52
YDL003W	MCD1	4994	3458	1.44	0.53	0.42	1.52
YER082C		4827	3343	1.44	0.53	0.42	1.52
YNL175C		9861	6830	1.44	0.53	0.42	1.52
YNL188W	KAR1	5940	4115	1.44	0.53	0.42	1.52
YPL226W		6480	4490	1.44	0.53	0.42	1.52
YCRX03C		390	270	1.44	0.53	0.42	1.52
YJL086C		1326	919	1.44	0.53	0.42	1.51
YNL091W		6302	4368	1.44	0.53	0.42	1.51
YJR070C		10161	7044	1.44	0.53	0.42	1.51
YDR267C		3003	2083	1.44	0.53	0.42	1.51
YDL057W		960	666	1.44	0.53	0.41	1.51
YER187W		402	279	1.44	0.53	0.41	1.51

YGR290W		1049	728	1.44	0.53	0.41	1.51
YDL017W	CDC7	3371	2342	1.44	0.53	0.41	1.50
YDR179W-A		1061	737	1.44	0.53	0.41	1.50
YDR384C		13904	9670	1.44	0.52	0.41	1.50
YLL031C		6644	4621	1.44	0.52	0.41	1.50
YHR199C		1172	815	1.44	0.52	0.41	1.50
YPL019C		16890	11750	1.44	0.52	0.41	1.49
YDR170C	SEC7	1844	1283	1.44	0.52	0.41	1.49
YOR366W		364	253	1.44	0.52	0.41	1.49
YER126C		24179	16833	1.44	0.52	0.41	1.49
YDR297W	SUR2	2498	1739	1.44	0.52	0.41	1.49
YLR407W		3597	2506	1.44	0.52	0.41	1.49
YJL189W	RPL39	11234	7828	1.44	0.52	0.41	1.49
YER097W		982	684	1.44	0.52	0.41	1.49
YNL132W		6740	4697	1.43	0.52	0.41	1.49
YPL189W		551	384	1.43	0.52	0.41	1.48
YNL110C		27506	19176	1.43	0.52	0.41	1.48
YAL019W	FUN30	2912	2031	1.43	0.52	0.41	1.48
YDL063C		4016	2802	1.43	0.52	0.41	1.48
YDR408C	ADE8	3136	2188	1.43	0.52	0.41	1.48
YCR026C		2220	1549	1.43	0.52	0.41	1.48
YHR163W	SOL3	4000	2793	1.43	0.52	0.41	1.48
YNL328C	MDJ2	571	399	1.43	0.52	0.41	1.48
YER106W		392	274	1.43	0.52	0.41	1.47
YNL262W	POL2	912	637	1.43	0.52	0.41	1.47
YNL303W		5293	3699	1.43	0.52	0.40	1.47
YLR359W	ADE13	3589	2508	1.43	0.52	0.40	1.47
YPL263C	KEL3	12405	8670	1.43	0.52	0.40	1.47
YPR168W	NUT2	1049	733	1.43	0.52	0.40	1.47
YHL023C		4619	3230	1.43	0.52	0.40	1.47
YDR471W	RPL27B	22291	15590	1.43	0.52	0.40	1.47
YBR057C	MUM2	8931	6247	1.43	0.52	0.40	1.47
YDL041W		688	481	1.43	0.52	0.40	1.47
YOR106W	VAM3	5054	3536	1.43	0.52	0.40	1.46
YML109W	ZDS2	1657	1160	1.43	0.51	0.40	1.46
YDR363W	ESC2	6869	4810	1.43	0.51	0.40	1.46

YDR024W		5516	3864	1.43	0.51	0.40	1.46
YOL059W	GPD2	21696	15209	1.43	0.51	0.40	1.45
YPL267W		9684	6791	1.43	0.51	0.40	1.45
YPL133C		2029	1423	1.43	0.51	0.40	1.45
YKR095W	MLP1	1866	1309	1.43	0.51	0.40	1.45
YMR290W-A		3824	2683	1.43	0.51	0.40	1.45
YDL134C-A		8550	6001	1.42	0.51	0.40	1.45
YNL144C		1832	1286	1.42	0.51	0.40	1.45
YPL207W		9439	6627	1.42	0.51	0.40	1.45
YNR063W		748	525	1.42	0.51	0.40	1.45
YCL059C	KRR1	17102	12010	1.42	0.51	0.40	1.45
YDR402C	DIT2	659	463	1.42	0.51	0.40	1.44
YOR188W	MSB1	3608	2535	1.42	0.51	0.40	1.44
YEL035C	UTR5	386	271	1.42	0.51	0.40	1.44
YEL055C	POL5	4213	2962	1.42	0.51	0.40	1.44
YML061C	PIF1	1762	1239	1.42	0.51	0.40	1.44
YJR112W	NNF1	5579	3924	1.42	0.51	0.40	1.44
YCR035C	RRP43	1855	1305	1.42	0.51	0.40	1.44
YDR136C		1290	908	1.42	0.51	0.39	1.44
YLL009C	COX17	13712	9649	1.42	0.51	0.39	1.43
YPL125W		2349	1653	1.42	0.51	0.39	1.43
YLR168C	MSF1'	8823	6215	1.42	0.51	0.39	1.43
YNL238W	KEX2	5876	4141	1.42	0.50	0.39	1.43
YKR027W		4247	2995	1.42	0.50	0.39	1.42
YIR013C	GAT4	380	268	1.42	0.50	0.39	1.42
YLL013C		7643	5391	1.42	0.50	0.39	1.42
YOR004W		25923	18292	1.42	0.50	0.39	1.42
YEL017W		1085	766	1.42	0.50	0.39	1.42
YBL035C	POL12	2726	1924	1.42	0.50	0.39	1.42
YNR003C	RPC34	6135	4332	1.42	0.50	0.39	1.42
YER074W	RPS24A	8461	5974	1.42	0.50	0.39	1.42
YNL023C	FAP1	3701	2613	1.42	0.50	0.39	1.42
YBR084W	MIS1	9784	6909	1.42	0.50	0.39	1.42
YIL012W		715	505	1.42	0.50	0.39	1.42
YGL201C	MCM6	5053	3569	1.42	0.50	0.39	1.41
YOR270C	VPH1	5863	4144	1.41	0.50	0.39	1.41

YNL316C	PHA2	570	403	1.41	0.50	0.39	1.41
YBL071C		4733	3346	1.41	0.50	0.39	1.41
YGL060W		2129	1505	1.41	0.50	0.39	1.41
YBL081W		3407	2409	1.41	0.50	0.39	1.41
YOR264W		8817	6238	1.41	0.50	0.39	1.41
YER169W	RPH1	2712	1920	1.41	0.50	0.39	1.40
YGR081C		14801	10482	1.41	0.50	0.39	1.40
YML064C	TEM1	10238	7251	1.41	0.50	0.39	1.40
YGR067C		1687	1195	1.41	0.50	0.39	1.40
YGR251W		6848	4851	1.41	0.50	0.38	1.40
YOR162C	YRR1	3505	2484	1.41	0.50	0.38	1.40
YDR014W		1762	1249	1.41	0.50	0.38	1.40
YCL049C		932	661	1.41	0.50	0.38	1.40
YPL217C	BMS1	6668	4728	1.41	0.50	0.38	1.40
YGL016W	KAP122	1336	947	1.41	0.50	0.38	1.40
YDR349C	YPS7	1206	855	1.41	0.50	0.38	1.39
YER138C		19636	13927	1.41	0.50	0.38	1.39
YHL007C	STE20	6678	4740	1.41	0.49	0.38	1.39
YDR010C		2355	1672	1.41	0.49	0.38	1.39
YLR084C	RAX2	975	692	1.41	0.49	0.38	1.39
YMR147W		2744	1948	1.41	0.49	0.38	1.39
YDR395W	SXM1	13441	9543	1.41	0.49	0.38	1.39
YLR353W	BUD8	1815	1289	1.41	0.49	0.38	1.39
YMR101C	SRT1	514	365	1.41	0.49	0.38	1.39
YOR341W	RPA190	10444	7418	1.41	0.49	0.38	1.39
YBR049C	REB1	1464	1040	1.41	0.49	0.38	1.39
YGL046W		859	610	1.41	0.49	0.38	1.38
YHR046C		645	459	1.41	0.49	0.38	1.38
YAL058W	CNE1	1932	1375	1.40	0.49	0.38	1.37
YLR090W	XDJ1	3582	2550	1.40	0.49	0.38	1.37
YKL118W		822	585	1.40	0.49	0.38	1.37
YNL184C		1722	1227	1.40	0.49	0.38	1.37
YLR307W	CDA1	837	596	1.40	0.49	0.38	1.37
YDR527W		5184	3693	1.40	0.49	0.38	1.37
YKL009W	MRT4	13582	9677	1.40	0.49	0.38	1.37
YMR006C	PLB2	1798	1281	1.40	0.49	0.38	1.37

YDR439W	LRS4	1274	908	1.40	0.49	0.38	1.37
YPL081W	RPS9A	14943	10650	1.40	0.49	0.38	1.37
YDR496C		11969	8533	1.40	0.49	0.38	1.37
YDL019C		2458	1753	1.40	0.49	0.38	1.36
YOL080C		13895	9913	1.40	0.49	0.37	1.36
YLR297W		3882	2770	1.40	0.49	0.37	1.36
YPR070W	MED1	5501	3925	1.40	0.49	0.37	1.36
YLL006W	MMM1	1781	1271	1.40	0.49	0.37	1.36
YIR041W		446	318	1.40	0.49	0.37	1.36
YLL007C		2005	1431	1.40	0.49	0.37	1.36
YKL055C	OAR1	1508	1076	1.40	0.49	0.37	1.36
YLR048W	RPS0B	18720	13361	1.40	0.49	0.37	1.36
YGR165W		6119	4368	1.40	0.49	0.37	1.36
YOL028C	YAP7	2271	1621	1.40	0.49	0.37	1.36
YMR058W	FET3	3187	2275	1.40	0.49	0.37	1.36
YHR213W		797	569	1.40	0.49	0.37	1.36
YBL019W	APN2	1560	1114	1.40	0.49	0.37	1.36
YBR145W	ADH5	6973	4981	1.40	0.49	0.37	1.36
YKR009C	FOX2	529	378	1.40	0.49	0.37	1.35
YDL228C		16112	11512	1.40	0.49	0.37	1.35
YJL047C	RTT101	2716	1941	1.40	0.48	0.37	1.35
YCL036W		10656	7617	1.40	0.48	0.37	1.35
YDL152W		2339	1672	1.40	0.48	0.37	1.35
YLL047W		541	387	1.40	0.48	0.37	1.35
YPR122W	AXL1	2768	1979	1.40	0.48	0.37	1.35
YJR097W		4176	2986	1.40	0.48	0.37	1.35
YDL049C	KNH1	912	652	1.40	0.48	0.37	1.35
YMR204C		934	668	1.40	0.48	0.37	1.35
YKR005C		503	360	1.40	0.48	0.37	1.35
YOR048C	RAT1	503	360	1.40	0.48	0.37	1.35
YNL014W		1955	1399	1.40	0.48	0.37	1.35
YDR420W	HKR1	1718	1230	1.40	0.48	0.37	1.35
YJR098C		1219	873	1.40	0.48	0.37	1.34
YOR074C	CDC21	1543	1105	1.40	0.48	0.37	1.34
YFR016C		1419	1016	1.40	0.48	0.37	1.34
YLR447C	VMA6	10180	7290	1.40	0.48	0.37	1.34

YLR425W	TUS1	1173	840	1.40	0.48	0.37	1.34
YLR131C	ACE2	7295	5225	1.40	0.48	0.37	1.34
YGR003W		3794	2718	1.40	0.48	0.37	1.34
YFL027C		5340	3827	1.40	0.48	0.37	1.34
YKR098C	UBP11	2458	1762	1.39	0.48	0.37	1.34
YOL069W	NUF2	5274	3782	1.39	0.48	0.37	1.34
YPR160W	GPH1	1326	951	1.39	0.48	0.37	1.34
YBR133C	HSL7	6607	4739	1.39	0.48	0.37	1.33
YKL125W	RRN3	7560	5423	1.39	0.48	0.37	1.33
YML099C	ARG81	2988	2145	1.39	0.48	0.37	1.33
YLR311C		880	632	1.39	0.48	0.37	1.33
YIL159W	BNR1	4029	2893	1.39	0.48	0.37	1.33
YBL112C		13846	9945	1.39	0.48	0.36	1.33
YOR296W		1899	1364	1.39	0.48	0.36	1.33
YMR122C		2008	1443	1.39	0.48	0.36	1.33
YKL085W	MDH1	6737	4841	1.39	0.48	0.36	1.32
YOR282W		5747	4130	1.39	0.48	0.36	1.32
YDR096W	GIS1	3579	2572	1.39	0.48	0.36	1.32
YGR243W		1561	1122	1.39	0.48	0.36	1.32
YGR017W		11531	8290	1.39	0.48	0.36	1.32
YFL049W		4536	3261	1.39	0.48	0.36	1.32
YML111W	BUL2	2077	1493	1.39	0.48	0.36	1.32
YHL029C		9118	6556	1.39	0.48	0.36	1.32
YER167W	BCK2	1524	1096	1.39	0.48	0.36	1.32
YOL106W		5701	4099	1.39	0.48	0.36	1.32
YFL033C	RIM15	3717	2673	1.39	0.48	0.36	1.32
YIL015W	BAR1	816	587	1.39	0.48	0.36	1.32
YDR021W	FAL1	2941	2115	1.39	0.48	0.36	1.32
YNL102W	POL1	2452	1764	1.39	0.48	0.36	1.32
YPL014W		901	648	1.39	0.48	0.36	1.32
YNR029C		5746	4134	1.39	0.47	0.36	1.32
YMR316C-B		595	428	1.39	0.47	0.36	1.32
YDR360W		942	678	1.39	0.47	0.36	1.32
YGR139W		514	370	1.39	0.47	0.36	1.32
YHR187W	IKI1	436	314	1.39	0.47	0.36	1.31
YPL072W	UBP16	5959	4294	1.39	0.47	0.36	1.31

YNL057W		8510	6134	1.39	0.47	0.36	1.31
YDR114C		2250	1622	1.39	0.47	0.36	1.31
YIL079C		6700	4830	1.39	0.47	0.36	1.31
YNL139C	RLR1	3822	2756	1.39	0.47	0.36	1.31
YDR365C		12988	9369	1.39	0.47	0.36	1.30
YLR287C-A	RPS30A	19203	13853	1.39	0.47	0.36	1.30
YGR107W		496	358	1.39	0.47	0.36	1.30
YDL156W		916	661	1.39	0.47	0.36	1.30
YMR036C	MIH1	3075	2219	1.39	0.47	0.36	1.30
YDR110W	FOB1	7452	5380	1.39	0.47	0.36	1.30
YMR105C	PGM2	4615	3333	1.38	0.47	0.36	1.30
YDR543C		436	315	1.38	0.47	0.36	1.30
YHR033W		674	487	1.38	0.47	0.36	1.30
YHR151C		1180	853	1.38	0.47	0.36	1.29
YPR148C		6637	4800	1.38	0.47	0.36	1.29
YKL033W		1691	1223	1.38	0.47	0.36	1.29
YDR431W		967	699	1.38	0.47	0.36	1.29
YDR239C		973	704	1.38	0.47	0.35	1.29
YML090W		619	448	1.38	0.47	0.35	1.29
YML122C		521	377	1.38	0.47	0.35	1.29
YNL240C	NAR1	1982	1435	1.38	0.47	0.35	1.29
YAR029W		403	292	1.38	0.47	0.35	1.29
YMR100W	MUB1	5064	3667	1.38	0.47	0.35	1.28
YDL231C		3606	2612	1.38	0.47	0.35	1.28
YHR076W		7777	5633	1.38	0.47	0.35	1.28
YJL050W	MTR4	4313	3124	1.38	0.47	0.35	1.28
YIR012W	SQT1	13139	9518	1.38	0.47	0.35	1.28
YLR274W	CDC46	6800	4926	1.38	0.47	0.35	1.28
YPR030W	CSR2	1144	829	1.38	0.46	0.35	1.28
YOR244W	ESA1	12361	8956	1.38	0.46	0.35	1.28
YLR062C		8931	6473	1.38	0.46	0.35	1.28
YLR296W		610	442	1.38	0.46	0.35	1.28
YLR403W	SFP1	3290	2386	1.38	0.46	0.35	1.28
YDR130C		3301	2394	1.38	0.46	0.35	1.28
YOL120C	RPL18A	31750	23025	1.38	0.46	0.35	1.28
YOR056C		8380	6078	1.38	0.46	0.35	1.28

YDL001W		1941	1408	1.38	0.46	0.35	1.28
YGL120C	PRP43	5035	3652	1.38	0.46	0.35	1.28
YER115C	SPR6	11821	8578	1.38	0.46	0.35	1.27
YKL152C	GPM1	8956	6499	1.38	0.46	0.35	1.27
YAR015W	ADE1	5247	3808	1.38	0.46	0.35	1.27
YDR374C		353	256	1.38	0.46	0.35	1.27
YER110C	KAP123	12376	8984	1.38	0.46	0.35	1.27
YER123W	YCK3	7430	5394	1.38	0.46	0.35	1.27
YER171W	RAD3	3643	2645	1.38	0.46	0.35	1.27
YNR065C		2946	2139	1.38	0.46	0.35	1.27
YDR285W	ZIP1	373	271	1.38	0.46	0.35	1.27
YCL002C		1906	1384	1.38	0.46	0.35	1.27
YPL097W	MSY1	3686	2677	1.38	0.46	0.35	1.27
YDR176W	NGG1	1167	848	1.38	0.46	0.35	1.27
YGL082W		1569	1140	1.38	0.46	0.35	1.27
YMR095C	SNO1	1003	729	1.38	0.46	0.35	1.27
YDR437W		1813	1317	1.38	0.46	0.35	1.27
YBR224W		618	449	1.38	0.46	0.35	1.27
YJL195C		2142	1557	1.38	0.46	0.35	1.27
YOR290C	SNF2	860	625	1.38	0.46	0.35	1.26
YNL017C		684	497	1.38	0.46	0.35	1.26
YPR120C	CLB5	6020	4378	1.38	0.46	0.35	1.26
YHR056C		4070	2961	1.37	0.46	0.35	1.26
YHR109W		26756	19467	1.37	0.46	0.35	1.26
YIL061C	SNP1	1579	1149	1.37	0.46	0.35	1.26
YDR523C	SPS1	809	589	1.37	0.46	0.35	1.26
YLR096W	KIN2	1740	1267	1.37	0.46	0.35	1.26
YDR277C	MTH1	893	650	1.37	0.46	0.35	1.26
YMR288W		2759	2009	1.37	0.46	0.35	1.25
YHL035C		3475	2531	1.37	0.46	0.34	1.25
YGR069W		3191	2324	1.37	0.46	0.34	1.25
YGR143W	SKN1	2934	2137	1.37	0.46	0.34	1.25
YLR441C	RPS1A	24722	18016	1.37	0.46	0.34	1.25
YCR097WA		379	276	1.37	0.46	0.34	1.25
YMR117C	SPC24	1833	1337	1.37	0.46	0.34	1.25
YLR032W	RAD5	3671	2677	1.37	0.46	0.34	1.25

YOR307C	SLY41	8682	6333	1.37	0.46	0.34	1.25
YEL014C		496	362	1.37	0.46	0.34	1.25
YPR124W	CTR1	32052	23382	1.37	0.46	0.34	1.25
YPR008W		4904	3578	1.37	0.45	0.34	1.24
YGR249W	MGA1	562	410	1.37	0.45	0.34	1.24
YCR085W		675	493	1.37	0.45	0.34	1.24
YMR017W	SPO20	726	530	1.37	0.45	0.34	1.24
YOR144C	EFD1	2567	1876	1.37	0.45	0.34	1.24
YJR071W		7191	5255	1.37	0.45	0.34	1.24
YML024W	RPS17A	11016	8051	1.37	0.45	0.34	1.24
YDL075W	RPL31A	14200	10385	1.37	0.45	0.34	1.23
YDR030C	RAD28	682	499	1.37	0.45	0.34	1.23
YDR466W		435	318	1.37	0.45	0.34	1.23
YER109C	FLO8	1923	1407	1.37	0.45	0.34	1.23
YJL085W	EXO70	3856	2821	1.37	0.45	0.34	1.23
YIR026C	YVH1	12413	9087	1.37	0.45	0.34	1.23
YML091C	RPM2	9630	7050	1.37	0.45	0.34	1.23
YBR021W	FUR4	1355	992	1.37	0.45	0.34	1.23
YDR488C	PAC11	2577	1887	1.37	0.45	0.34	1.23
YAR014C		2388	1749	1.37	0.45	0.34	1.23
YOR377W	ATF1	7106	5207	1.36	0.45	0.34	1.22
YJL071W	ARG2	2824	2071	1.36	0.45	0.34	1.22
YBL005W-B		1099	806	1.36	0.45	0.34	1.22
YNL076W	MKS1	6522	4783	1.36	0.45	0.34	1.22
YLR068W		5788	4245	1.36	0.45	0.33	1.22
YOL102C	TPT1	4957	3636	1.36	0.45	0.33	1.22
YJR157W		476	349	1.36	0.45	0.33	1.22
YGR051C		931	683	1.36	0.45	0.33	1.22
YGR083C	GCD2	9141	6709	1.36	0.45	0.33	1.21
YDL105W	QRI2	2036	1495	1.36	0.45	0.33	1.21
YGR094W	VAS1	15632	11482	1.36	0.45	0.33	1.21
YPL085W	SEC16	995	731	1.36	0.45	0.33	1.21
YNL121C	TOM70	8250	6060	1.36	0.45	0.33	1.21
YLR236C		960	705	1.36	0.44	0.33	1.21
YBR247C	ENP1	34633	25446	1.36	0.44	0.33	1.21
YLL034C		614	451	1.36	0.44	0.33	1.21

YOR160W	MTR10	2081	1530	1.36	0.44	0.33	1.20
YMR270C	RRN9	4305	3166	1.36	0.44	0.33	1.20
YOR124C	UBP2	986	725	1.36	0.44	0.33	1.20
YGL122C	NAB2	983	723	1.36	0.44	0.33	1.20
YDR463W	STP1	3181	2340	1.36	0.44	0.33	1.20
YJL022W		1610	1185	1.36	0.44	0.33	1.20
YLL035W		7692	5662	1.36	0.44	0.33	1.20
YNR012W	URK1	14049	10342	1.36	0.44	0.33	1.20
YPR144C		4468	3289	1.36	0.44	0.33	1.20
YFR024C-A		1256	925	1.36	0.44	0.33	1.20
YNL324W		617	454	1.36	0.44	0.33	1.20
YPR095C	SYT1	595	438	1.36	0.44	0.33	1.20
YBR012W-B		394	290	1.36	0.44	0.33	1.20
YLR305C	STT4	4130	3043	1.36	0.44	0.33	1.19
YGR021W		12562	9256	1.36	0.44	0.33	1.19
YDL180W		1465	1080	1.36	0.44	0.33	1.19
YDR042C		876	646	1.36	0.44	0.33	1.19
YGR061C	ADE6	7360	5426	1.36	0.44	0.33	1.19
YER016W	BIM1	2429	1791	1.36	0.44	0.33	1.19
YLR320W		2718	2004	1.36	0.44	0.33	1.19
YOR371C		2176	1605	1.36	0.44	0.33	1.19
YLL005C		2319	1710	1.36	0.44	0.33	1.19
YLR400W		946	698	1.36	0.44	0.33	1.19
YIR002C	MPH1	4730	3492	1.35	0.44	0.33	1.18
YHR169W	DBP8	3191	2356	1.35	0.44	0.33	1.18
YMR206W		570	421	1.35	0.44	0.32	1.18
YDR219C		1133	837	1.35	0.44	0.32	1.18
YJL200C		18016	13307	1.35	0.44	0.32	1.18
YDR028C	REG1	1807	1335	1.35	0.44	0.32	1.18
YJL043W		686	507	1.35	0.44	0.32	1.18
YMR046C		33588	24816	1.35	0.44	0.32	1.18
YBR186W	PCH2	1202	888	1.35	0.44	0.32	1.18
YOL152W	FRE7	7073	5227	1.35	0.44	0.32	1.18
YIR010W		5870	4338	1.35	0.44	0.32	1.18
YBL017C	PEP1	798	590	1.35	0.44	0.32	1.18
YML005W		6980	5159	1.35	0.44	0.32	1.18

YER140W		6815	5039	1.35	0.44	0.32	1.17
YDR128W		2878	2128	1.35	0.44	0.32	1.17
YBR065C	ECM2	5003	3702	1.35	0.43	0.32	1.17
YNL016W	PUB1	6262	4634	1.35	0.43	0.32	1.17
YDR389W	SAC7	9486	7020	1.35	0.43	0.32	1.17
YDR194C	MSS116	2681	1984	1.35	0.43	0.32	1.17
YDR393W	SHE9	8033	5945	1.35	0.43	0.32	1.17
YJL125C	GCD14	5407	4002	1.35	0.43	0.32	1.17
YER122C	GLO3	17713	13113	1.35	0.43	0.32	1.17
YHR058C	MED6	5154	3816	1.35	0.43	0.32	1.17
YKR082W	NUP133	3426	2537	1.35	0.43	0.32	1.17
YLR288C	MEC3	3323	2462	1.35	0.43	0.32	1.16
YOR140W	SFL1	2659	1970	1.35	0.43	0.32	1.16
YMR228W	MTF1	1701	1260	1.35	0.43	0.32	1.16
YHL019C	APM2	3805	2819	1.35	0.43	0.32	1.16
YDL103C	QRI1	1057	783	1.35	0.43	0.32	1.16
YOL061W	PRS5	13307	9861	1.35	0.43	0.32	1.16
YCR033W		1147	850	1.35	0.43	0.32	1.16
YBL023C	MCM2	2401	1780	1.35	0.43	0.32	1.16
YGR022C		600	445	1.35	0.43	0.32	1.16
YIL158W		10772	7988	1.35	0.43	0.32	1.16
YOR072W		597	443	1.35	0.43	0.32	1.16
YMR129W	POM152	2772	2056	1.35	0.43	0.32	1.16
YKR060W		3355	2488	1.35	0.43	0.32	1.16
YBR238C		2957	2193	1.35	0.43	0.32	1.16
YMR050C		8068	5984	1.35	0.43	0.32	1.16
YKR021W		2641	1959	1.35	0.43	0.32	1.16
YDR138W	HPR1	4366	3239	1.35	0.43	0.32	1.16
YDR172W	SUP35	480	356	1.35	0.43	0.32	1.16
YPR010C	RPA135	9072	6733	1.35	0.43	0.32	1.16
YJL028W		1207	896	1.35	0.43	0.32	1.15
YOR314W		1206	895	1.35	0.43	0.32	1.15
YGL032C	AGA2	548	407	1.35	0.43	0.32	1.15
YKR024C	DBP7	1709	1269	1.35	0.43	0.32	1.15
YLR304C	ACO1	22801	16933	1.35	0.43	0.32	1.15
YDL062W		5951	4420	1.35	0.43	0.32	1.15

YLL003W	SFI1	2428	1804	1.35	0.43	0.32	1.15
YOR182C	RPS30B	12131	9018	1.35	0.43	0.32	1.15
YLR072W		824	613	1.34	0.43	0.31	1.15
YMR076C	PDS5	1799	1338	1.34	0.43	0.31	1.14
YOR029W		488	363	1.34	0.43	0.31	1.14
YDR052C	DBF4	4007	2982	1.34	0.43	0.31	1.14
YOR391C		392	292	1.34	0.43	0.31	1.14
YBR155W	CNS1	6137	4568	1.34	0.43	0.31	1.14
YCRX19W		848	631	1.34	0.43	0.31	1.14
YKL006W	RPL14A	1381	1028	1.34	0.43	0.31	1.14
YNL166C	BNI5	11669	8689	1.34	0.43	0.31	1.14
YLL008W	DRS1	11655	8679	1.34	0.43	0.31	1.14
YBR085W	AAC3	2171	1617	1.34	0.42	0.31	1.14
YER125W	RSP5	12690	9453	1.34	0.42	0.31	1.14
YNL021W	HDA1	3202	2385	1.34	0.42	0.31	1.14
YGR281W	YOR1	5433	4047	1.34	0.42	0.31	1.14
YPR027C		1982	1477	1.34	0.42	0.31	1.13
YNL082W	PMS1	5046	3760	1.34	0.42	0.31	1.13
YPL027W		637	475	1.34	0.42	0.31	1.13
YOL121C	RPS19A	12580	9381	1.34	0.42	0.31	1.13
YOR346W	REV1	4558	3399	1.34	0.42	0.31	1.13
YGL216W	KIP3	5316	3965	1.34	0.42	0.31	1.13
YGL169W	SUA5	11184	8341	1.34	0.42	0.31	1.13
YBR064W		761	568	1.34	0.42	0.31	1.13
YGR099W	TEL2	2540	1895	1.34	0.42	0.31	1.13
YDL085W		1215	907	1.34	0.42	0.31	1.13
YDR355C		1537	1147	1.34	0.42	0.31	1.13
YKL037W		1196	893	1.34	0.42	0.31	1.12
YEL025C		2391	1785	1.34	0.42	0.31	1.12
YJR032W	CPR7	7198	5374	1.34	0.42	0.31	1.12
YGR296W	YRF1-3	1254	936	1.34	0.42	0.31	1.12
YJR082C		3818	2852	1.34	0.42	0.31	1.12
YPR031W		2312	1727	1.34	0.42	0.31	1.12
YIR020C		950	710	1.34	0.42	0.31	1.12
YJL042W	MHP1	2653	1983	1.34	0.42	0.31	1.12
YDR324C		6432	4807	1.34	0.42	0.31	1.12

YIL100W		440	329	1.34	0.42	0.31	1.12
YPL160W	CDC60	8671	6482	1.34	0.42	0.31	1.12
YCR047C		6595	4930	1.34	0.42	0.31	1.12
YOR170W		2170	1623	1.34	0.42	0.31	1.11
YNL164C		7023	5255	1.34	0.42	0.31	1.11
YDR379W	RGA2	4621	3459	1.34	0.42	0.31	1.11
YBL079W	NUP170	578	433	1.34	0.42	0.30	1.11
YOR359W		5452	4084	1.33	0.42	0.30	1.11
YHR158C	KEL1	2429	1820	1.33	0.42	0.30	1.11
YLR152C		3604	2700	1.33	0.42	0.30	1.11
YEL039C	CYC7	1118	838	1.33	0.42	0.30	1.10
YDR441C	APT2	2228	1670	1.33	0.42	0.30	1.10
YLL021W	SPA2	5449	4084	1.33	0.42	0.30	1.10
YPL103C		3053	2288	1.33	0.42	0.30	1.10
YKL095W	YJU2	1538	1153	1.33	0.42	0.30	1.10
YNL248C	RPA49	13635	10226	1.33	0.42	0.30	1.10
YJL025W	RRN7	3554	2666	1.33	0.41	0.30	1.10
YER032W	FIR1	3057	2293	1.33	0.41	0.30	1.10
YAL064W		643	482	1.33	0.41	0.30	1.10
YEL023C		1337	1003	1.33	0.41	0.30	1.10
YOR034C	AKR2	4640	3481	1.33	0.41	0.30	1.10
YIL165C		1850	1388	1.33	0.41	0.30	1.10
YDL031W	DBP10	3213	2411	1.33	0.41	0.30	1.10
YMR280C	CAT8	525	394	1.33	0.41	0.30	1.10
YMR094W	CTF13	1713	1286	1.33	0.41	0.30	1.10
YGR119C	NUP57	6518	4894	1.33	0.41	0.30	1.09
YDR359C		5787	4346	1.33	0.41	0.30	1.09
YNL242W		1103	829	1.33	0.41	0.30	1.09
YLR223C	IFH1	5308	3989	1.33	0.41	0.30	1.09
YMR019W	STB4	3703	2783	1.33	0.41	0.30	1.09
YCL037C	SRO9	9660	7260	1.33	0.41	0.30	1.09
YFL067W		12089	9087	1.33	0.41	0.30	1.09
YOR312C	RPL20B	11733	8822	1.33	0.41	0.30	1.09
YLR122C		633	476	1.33	0.41	0.30	1.09
YOL141W		972	731	1.33	0.41	0.30	1.09
YFR031C-A	RPL2A	15729	11834	1.33	0.41	0.30	1.08

YPR123C		9107	6854	1.33	0.41	0.30	1.08
YPR092W		2269	1708	1.33	0.41	0.30	1.08
YDR112W		1228	924	1.33	0.41	0.30	1.08
YHR006W	STP2	6118	4606	1.33	0.41	0.30	1.08
YDR108W	GSG1	6317	4757	1.33	0.41	0.30	1.08
YJL197W	UBP12	1754	1321	1.33	0.41	0.30	1.08
YDR191W	HST4	1613	1215	1.33	0.41	0.30	1.08
YDR223W		688	518	1.33	0.41	0.30	1.08
YJL067W		1873	1411	1.33	0.41	0.30	1.08
YKL104C	GFA1	3699	2789	1.33	0.41	0.30	1.07
YOR028C	CIN5	1457	1099	1.33	0.41	0.29	1.07
YHR219W		16273	12273	1.33	0.41	0.29	1.07
YGR157W	CHO2	2778	2095	1.33	0.41	0.29	1.07
YGR272C		16674	12577	1.33	0.41	0.29	1.07
YHR043C	DOG2	12984	9794	1.33	0.41	0.29	1.07
YCRX01W		1053	794	1.33	0.41	0.29	1.07
YNR067C		9110	6872	1.33	0.41	0.29	1.07
YJR136C		5751	4339	1.33	0.41	0.29	1.07
YBL092W	RPL32	5919	4466	1.33	0.41	0.29	1.07
YDR445C		1429	1078	1.33	0.41	0.29	1.07
YOR026W	BUB3	2369	1788	1.33	0.41	0.29	1.07
YER099C	PRS2	19990	15088	1.32	0.41	0.29	1.07
YOL004W	SIN3	602	454	1.32	0.41	0.29	1.07
YML037C		815	615	1.32	0.41	0.29	1.07
YNL143C		2442	1843	1.32	0.41	0.29	1.07
YGR123C	PPT1	6451	4870	1.32	0.41	0.29	1.07
YMR179W	SPT21	5733	4329	1.32	0.41	0.29	1.07
YMR126C		5684	4292	1.32	0.41	0.29	1.07
YAR018C	KIN3	3252	2457	1.32	0.40	0.29	1.06
YCL052C	PBN1	2909	2198	1.32	0.40	0.29	1.06
YKR063C	LAS1	6666	5037	1.32	0.40	0.29	1.06
YBR292C		1158	875	1.32	0.40	0.29	1.06
YDL073W		742	561	1.32	0.40	0.29	1.06
YPL268W	PLC1	4558	3445	1.32	0.40	0.29	1.06
YDR004W	RAD57	1754	1326	1.32	0.40	0.29	1.06
YDR174W	HMO1	15363	11615	1.32	0.40	0.29	1.06

YHL003C	LAG1	12998	9827	1.32	0.40	0.29	1.06
YMR057C		731	553	1.32	0.40	0.29	1.06
YPL026C	SKS1	1157	875	1.32	0.40	0.29	1.05
YJR132W	NMD5	4820	3647	1.32	0.40	0.29	1.05
YER008C	SEC3	834	631	1.32	0.40	0.29	1.05
YHL025W	SNF6	10521	7961	1.32	0.40	0.29	1.05
YGR183C	QCR9	8826	6679	1.32	0.40	0.29	1.05
YLR162W		4865	3682	1.32	0.40	0.29	1.05
YFL008W	SMC1	3748	2837	1.32	0.40	0.29	1.05
YOR266W	PNT1	2395	1813	1.32	0.40	0.29	1.05
YNL078W		10118	7659	1.32	0.40	0.29	1.05
YLR056W	ERG3	17655	13367	1.32	0.40	0.29	1.05
YAL035W	FUN12	6242	4726	1.32	0.40	0.29	1.05
YDR255C		581	440	1.32	0.40	0.29	1.05
YPL135W	ISU1	3666	2777	1.32	0.40	0.29	1.05
YFL035C-B		26049	19732	1.32	0.40	0.29	1.05
YER056C	FCY2	3147	2384	1.32	0.40	0.29	1.05
YFL052W		452	343	1.32	0.40	0.29	1.04
YGR085C	RPL11B	26554	20131	1.32	0.40	0.29	1.04
YLR457C	NBP1	2416	1832	1.32	0.40	0.29	1.04
YNL247W		19958	15139	1.32	0.40	0.29	1.04
YLR406C	RPL31B	995	755	1.32	0.40	0.29	1.04
YDL101C	DUN1	2422	1838	1.32	0.40	0.29	1.04
YOR376W		362	275	1.32	0.40	0.29	1.04
YDR307W		1049	796	1.32	0.40	0.29	1.04
YBR267W		17014	12918	1.32	0.40	0.28	1.04
YJL057C	IKS1	4923	3738	1.32	0.40	0.28	1.04
YPL158C		9296	7059	1.32	0.40	0.28	1.04
YHR155W		5214	3960	1.32	0.40	0.28	1.03
YIL161W		9237	7017	1.32	0.40	0.28	1.03
YLR309C	IMH1	3018	2293	1.32	0.40	0.28	1.03
YMR069W		1027	780	1.32	0.40	0.28	1.03
YGL150C	INO80	2898	2202	1.32	0.40	0.28	1.03
YIL147C	SLN1	4234	3217	1.32	0.40	0.28	1.03
YMR198W	CIK1	1023	777	1.32	0.40	0.28	1.03
YBR218C	PYC2	986	749	1.32	0.40	0.28	1.03

YGL238W	CSE1	12171	9249	1.32	0.40	0.28	1.03
YMR116C	ASC1	21549	16376	1.32	0.40	0.28	1.03
YML078W	CPR3	7941	6035	1.32	0.40	0.28	1.03
YOR351C	MEK1	772	587	1.32	0.40	0.28	1.03
YER117W	RPL23B	23520	17875	1.32	0.40	0.28	1.03
YNL054W	VAC7	1595	1213	1.32	0.40	0.28	1.03
YKR055W	RHO4	1136	864	1.31	0.39	0.28	1.03
YDR459C		2746	2089	1.31	0.39	0.28	1.03
YDR107C		582	443	1.31	0.39	0.28	1.03
YNL031C	HHT2	26103	19857	1.31	0.39	0.28	1.03
YOR325W		2321	1766	1.31	0.39	0.28	1.03
YGR221C		545	415	1.31	0.39	0.28	1.03
YLR174W	IDP2	714	543	1.31	0.39	0.28	1.02
YLR063W		3085	2348	1.31	0.39	0.28	1.02
YKR059W	TIF1	21282	16196	1.31	0.39	0.28	1.02
YKR073C		1275	971	1.31	0.39	0.28	1.02
YPL280W		402	306	1.31	0.39	0.28	1.02
YCR001W		734	559	1.31	0.39	0.28	1.02
YKR029C		3601	2742	1.31	0.39	0.28	1.02
YDR034C	LYS14	3964	3020	1.31	0.39	0.28	1.02
YDR515W	SLF1	8801	6706	1.31	0.39	0.28	1.02
YPR014C		2157	1644	1.31	0.39	0.28	1.02
YPR149W	NCE102	45932	35008	1.31	0.39	0.28	1.02
YKL089W	MIF2	1982	1511	1.31	0.39	0.28	1.02
YHR214C-B		4379	3338	1.31	0.39	0.28	1.01
YMR065W	KAR5	3258	2484	1.31	0.39	0.28	1.01
YDR057W		5870	4476	1.31	0.39	0.28	1.01
YJL014W	CCT3	14760	11255	1.31	0.39	0.28	1.01
YMR097C		5162	3937	1.31	0.39	0.28	1.01
YNR045W	PET494	384	293	1.31	0.39	0.28	1.01
YAR043C		800	610	1.31	0.39	0.28	1.01
YDR341C		797	608	1.31	0.39	0.28	1.01
YOR393W	ERR1	1211	924	1.31	0.39	0.28	1.01
YHR045W		956	729	1.31	0.39	0.28	1.01
YER088C	DOT6	1239	945	1.31	0.39	0.28	1.01
YKR077W		5932	4526	1.31	0.39	0.28	1.01

YKL116C		3612	2756	1.31	0.39	0.28	1.01
YDL205C	HEM3	19579	14942	1.31	0.39	0.28	1.01
YMR171C		6036	4607	1.31	0.39	0.28	1.01
YKL057C	NUP120	1679	1282	1.31	0.39	0.28	1.01
YDR435C		1113	850	1.31	0.39	0.28	1.00
YAR035W	YAT1	711	543	1.31	0.39	0.28	1.00
YDR346C		2316	1769	1.31	0.39	0.28	1.00
YDR106W	ARP10	987	754	1.31	0.39	0.28	1.00
YJL049W		4682	3577	1.31	0.39	0.28	1.00
YBL105C	PKC1	1352	1033	1.31	0.39	0.28	1.00
YML106W	URA5	9664	7385	1.31	0.39	0.28	1.00
YJR060W	CBF1	1951	1491	1.31	0.39	0.28	1.00
YDR149C		548	419	1.31	0.39	0.28	1.00
YJR126C		1856	1419	1.31	0.39	0.28	1.00
YOL100W	PKH2	7184	5493	1.31	0.39	0.27	1.00
YCR067C	SED4	2291	1752	1.31	0.39	0.27	1.00
YPR026W	ATH1	2016	1542	1.31	0.39	0.27	1.00
YDL170W	UGA3	3811	2915	1.31	0.39	0.27	1.00
YML107C		1768	1352	1.31	0.39	0.27	1.00
YMR189W	GCV2	599	458	1.31	0.39	0.27	1.00
YCR072C		4969	3801	1.31	0.39	0.27	1.00
YHL011C	PRS3	20927	16008	1.31	0.39	0.27	1.00
YBR115C	LYS2	5199	3977	1.31	0.39	0.27	1.00
YJL129C	TRK1	2537	1941	1.31	0.39	0.27	1.00
YOL067C	RTG1	2735	2093	1.31	0.39	0.27	1.00
YNL118C	DCP2	7189	5501	1.31	0.39	0.27	1.00
YDR485C		1954	1495	1.31	0.39	0.27	0.99
YBR011C	IPP1	20953	16035	1.31	0.39	0.27	0.99
YFL036W	RPO41	3070	2350	1.31	0.39	0.27	0.99
YPR042C		619	474	1.31	0.39	0.27	0.99
YBR055C	PRP6	2559	1959	1.31	0.39	0.27	0.99
YER132C	PMD1	4226	3235	1.31	0.39	0.27	0.99
YDL112W	TRM3	3106	2378	1.31	0.39	0.27	0.99
YHR183W	GND1	3398	2602	1.31	0.39	0.27	0.99
YPL175W	SPT14	3513	2690	1.31	0.39	0.27	0.99
YNL075W	IMP4	11904	9116	1.31	0.39	0.27	0.99

YJL084C		3260	2498	1.31	0.38	0.27	0.99
YLR289W	GUF1	4437	3400	1.31	0.38	0.27	0.99
YLR445W		432	331	1.31	0.38	0.27	0.99
YNL174W		14063	10776	1.31	0.38	0.27	0.99
YGR217W	CCH1	718	550	1.30	0.38	0.27	0.99
YLR129W	DIP2	10718	8215	1.30	0.38	0.27	0.99
YLR381W		399	306	1.30	0.38	0.27	0.99
YAR071W	PHO11	18768	14388	1.30	0.38	0.27	0.99
YFL003C	MSH4	595	456	1.30	0.38	0.27	0.98
YMR323W		622	477	1.30	0.38	0.27	0.98
YCL019W		23874	18312	1.30	0.38	0.27	0.98
YNL061W	NOP2	18671	14323	1.30	0.38	0.27	0.98
YJR134C	SGM1	5255	4032	1.30	0.38	0.27	0.98
YHR072W	ERG7	1955	1500	1.30	0.38	0.27	0.98
YAR052C		1418	1088	1.30	0.38	0.27	0.98
YDR036C		2640	2026	1.30	0.38	0.27	0.98
YDR023W	SES1	3195	2452	1.30	0.38	0.27	0.98
YGL164C		3519	2701	1.30	0.38	0.27	0.98
YNL123W		7922	6081	1.30	0.38	0.27	0.98
YDR509W		718	551	1.30	0.38	0.27	0.98
YHR017W	YSC83	990	760	1.30	0.38	0.27	0.98
YJR002W	MPP10	1813	1392	1.30	0.38	0.27	0.98
YGL229C	SAP4	1400	1075	1.30	0.38	0.27	0.98
YDL240W	LRG1	2245	1724	1.30	0.38	0.27	0.98
YLR103C	CDC45	2842	2183	1.30	0.38	0.27	0.98
YBR138C	HDR1	2022	1553	1.30	0.38	0.27	0.98
YBL049W		710	545	1.30	0.38	0.27	0.98
YPL211W	NIP7	15616	11998	1.30	0.38	0.27	0.97
YAR009C		35520	27291	1.30	0.38	0.27	0.97
YHR074W		5427	4170	1.30	0.38	0.27	0.97
YCRX20C		1248	959	1.30	0.38	0.27	0.97
YML119W		17616	13536	1.30	0.38	0.27	0.97
YMR021C	MAC1	5463	4198	1.30	0.38	0.27	0.97
YCL004W	PGS1	1081	831	1.30	0.38	0.27	0.97
YKR041W		1106	850	1.30	0.38	0.27	0.97
YLR008C		7315	5626	1.30	0.38	0.27	0.97

YAL002W	VPS8	1541	1185	1.30	0.38	0.27	0.97
YKL134C	36433	1485	1142	1.30	0.38	0.27	0.97
YBR097W	VPS15	370	285	1.30	0.38	0.27	0.97
YJL019W		1956	1505	1.30	0.38	0.27	0.97
YJL136C	RPS21B	20193	15536	1.30	0.38	0.27	0.97
YMR012W	CLU1	897	690	1.30	0.38	0.27	0.97
YCL076W		577	444	1.30	0.38	0.27	0.96
YFL023W		8361	6436	1.30	0.38	0.27	0.96
YBR147W		1065	820	1.30	0.38	0.26	0.96
YGL098W		4507	3471	1.30	0.38	0.26	0.96
YBL061C	SKT5	2087	1608	1.30	0.38	0.26	0.96
YJR076C	CDC11	3915	3016	1.30	0.38	0.26	0.96
YOL113W	SKM1	3319	2557	1.30	0.38	0.26	0.96
YGR045C		1686	1299	1.30	0.38	0.26	0.96
YJL191W	RPS14B	15329	11814	1.30	0.38	0.26	0.96
YGL131C		3211	2475	1.30	0.38	0.26	0.96
YJR042W	NUP85	1984	1529	1.30	0.38	0.26	0.96
YJR029W		381	294	1.30	0.38	0.26	0.96
YMR099C		8026	6187	1.30	0.38	0.26	0.96
YGL194C	HOS2	8291	6392	1.30	0.38	0.26	0.96
YMR133W	REC114	912	703	1.30	0.38	0.26	0.96
YEL031W	SPF1	554	427	1.30	0.37	0.26	0.95
YHR093W	AHT1	9691	7478	1.30	0.37	0.26	0.95
YDR525W		671	518	1.30	0.37	0.26	0.95
YKR054C	DYN1	495	382	1.30	0.37	0.26	0.95
YBR121C	GRS1	18087	13962	1.30	0.37	0.26	0.95
YPR096C		1854	1431	1.30	0.37	0.26	0.95
YMR137C	PSO2	5137	3966	1.30	0.37	0.26	0.95
YKL063C		8813	6804	1.30	0.37	0.26	0.95
YMR242C	RPL20A	22824	17622	1.30	0.37	0.26	0.95
YCL074W		958	740	1.30	0.37	0.26	0.95
YOR092W	ECM3	1349	1042	1.29	0.37	0.26	0.95
YGR283C		9502	7339	1.29	0.37	0.26	0.95
YOR315W		2593	2003	1.29	0.37	0.26	0.95
YOL070C		8171	6312	1.29	0.37	0.26	0.95
YGL256W	ADH4	10142	7836	1.29	0.37	0.26	0.94

YMR176W	ECM5	2228	1722	1.29	0.37	0.26	0.94
YOR025W	HST3	4494	3473	1.29	0.37	0.26	0.94
YOL052C	SPE2	15482	11966	1.29	0.37	0.26	0.94
YHR117W	TOM71	2483	1919	1.29	0.37	0.26	0.94
YLR431C		2071	1601	1.29	0.37	0.26	0.94
YPR164W	KIM3	2455	1898	1.29	0.37	0.26	0.94
YMR232W	FUS2	361	279	1.29	0.37	0.26	0.94
YOR084W		3170	2451	1.29	0.37	0.26	0.94
YEL053C	MAK10	1750	1353	1.29	0.37	0.26	0.94
YGL007W		1791	1385	1.29	0.37	0.26	0.94
YNL083W		4509	3487	1.29	0.37	0.26	0.94
YLR451W	LEU3	4243	3282	1.29	0.37	0.26	0.94
YJL039C	NUP192	1303	1008	1.29	0.37	0.26	0.94
YDR440W	DOT1	1539	1191	1.29	0.37	0.26	0.94
YDR398W		11199	8665	1.29	0.37	0.26	0.94
YBR179C	FZO1	4165	3223	1.29	0.37	0.26	0.94
YBR300C		2503	1937	1.29	0.37	0.26	0.94
YIL093C		10776	8341	1.29	0.37	0.26	0.94
YER073W	ALD5	3995	3092	1.29	0.37	0.26	0.93
YKL164C	PIR1	540	418	1.29	0.37	0.26	0.93
YOL015W		3169	2453	1.29	0.37	0.26	0.93
YKL004W	AUR1	18812	14563	1.29	0.37	0.26	0.93
YDR097C	MSH6	4786	3706	1.29	0.37	0.26	0.93
YDL060W		5731	4438	1.29	0.37	0.26	0.93
YNL122C		10566	8183	1.29	0.37	0.26	0.93
YBL034C	STU1	1116	864	1.29	0.37	0.26	0.93
YGL099W		8457	6550	1.29	0.37	0.26	0.93
YGL234W	ADE5,7	8466	6558	1.29	0.37	0.26	0.93
YKL156W	RPS27A	12565	9733	1.29	0.37	0.26	0.93
YPL253C	VIK1	1706	1322	1.29	0.37	0.26	0.93
YLR409C		3180	2464	1.29	0.37	0.26	0.93
YJL027C		643	498	1.29	0.37	0.26	0.93
YLR323C		3969	3076	1.29	0.37	0.26	0.93
YDR321W	ASP1	4923	3816	1.29	0.37	0.26	0.93
YER041W		15219	11799	1.29	0.37	0.25	0.93
YMR124W		3835	2973	1.29	0.37	0.25	0.93

YMR143W	RPS16A	12217	9476	1.29	0.37	0.25	0.92
YDR220C		1042	808	1.29	0.37	0.25	0.92
YOR076C		2545	1975	1.29	0.37	0.25	0.92
YGR173W		8711	6759	1.29	0.37	0.25	0.92
YER040W	GLN3	5865	4551	1.29	0.37	0.25	0.92
YHR203C	RPS4B	1683	1306	1.29	0.37	0.25	0.92
YGL034C		403	313	1.29	0.37	0.25	0.92
YLR124W		604	469	1.29	0.37	0.25	0.92
YDL087C	LUC7	1558	1210	1.29	0.37	0.25	0.92
YKL184W	SPE1	6096	4733	1.29	0.37	0.25	0.92
YAL060W		9573	7436	1.29	0.36	0.25	0.92
YLL059C		1125	874	1.29	0.36	0.25	0.92
YNL270C	ALP1	859	667	1.29	0.36	0.25	0.92
YPL095C		3102	2410	1.29	0.36	0.25	0.92
YOR116C	RPO31	1471	1143	1.29	0.36	0.25	0.91
YOR046C	DBP5	11932	9272	1.29	0.36	0.25	0.91
YLL046C	RNP1	854	664	1.29	0.36	0.25	0.91
YPL266W	DIM1	16290	12660	1.29	0.36	0.25	0.91
YGL044C	RNA15	3962	3079	1.29	0.36	0.25	0.91
YBR043C		4804	3734	1.29	0.36	0.25	0.91
YNL140C		2268	1763	1.29	0.36	0.25	0.91
YMR236W	TAF17	10779	8381	1.29	0.36	0.25	0.91
YIR004W	DJP1	10014	7791	1.29	0.36	0.25	0.91
YMR239C	RNT1	7922	6164	1.29	0.36	0.25	0.91
YPR116W		1009	785	1.29	0.36	0.25	0.91
YOR112W		3213	2500	1.29	0.36	0.25	0.91
YLL065W	GIN11	663	516	1.28	0.36	0.25	0.91
YER053C		2201	1713	1.28	0.36	0.25	0.91
YAL058C-A		1172	912	1.28	0.36	0.25	0.91
YIL127C		20632	16063	1.28	0.36	0.25	0.90
YFR021W		2550	1985	1.28	0.36	0.25	0.90
YOR365C		1906	1484	1.28	0.36	0.25	0.90
YDR213W		1099	856	1.28	0.36	0.25	0.90
YDR144C	MKC7	782	609	1.28	0.36	0.25	0.90
YOR060C		1959	1526	1.28	0.36	0.25	0.90
YNL013C		1010	787	1.28	0.36	0.25	0.90

YPR180W	AOS1	4373	3407	1.28	0.36	0.25	0.90
YJR075W	HOC1	4535	3533	1.28	0.36	0.25	0.90
YCL069W		5718	4456	1.28	0.36	0.25	0.90
YML113W	DAT1	7725	6021	1.28	0.36	0.25	0.90
YGL215W	CLG1	20243	15779	1.28	0.36	0.25	0.90
YOL148C	SPT20	2833	2208	1.28	0.36	0.25	0.90
YHR185C	ADY1	416	324	1.28	0.36	0.25	0.90
YOR235W	SNR17A	519	405	1.28	0.36	0.25	0.90
YLR060W	FRS1	16589	12935	1.28	0.36	0.25	0.90
YIL129C	TAO3	1658	1293	1.28	0.36	0.25	0.90
YLR144C	ACF2	3908	3048	1.28	0.36	0.25	0.90
YNL146W		3139	2448	1.28	0.36	0.25	0.90
YDL232W	OST4	4867	3796	1.28	0.36	0.25	0.89
YOR018W	ROD1	3658	2854	1.28	0.36	0.25	0.89
YDR101C		10144	7914	1.28	0.36	0.25	0.89
YHR038W	FIL1	5524	4311	1.28	0.36	0.25	0.89
YBR103W		6518	5087	1.28	0.36	0.25	0.89
YFL053W	DAK2	980	765	1.28	0.36	0.25	0.89
YLL015W	BPT1	569	444	1.28	0.36	0.24	0.89
YNL049C	SFB2	3206	2503	1.28	0.36	0.24	0.89
YIR042C		4798	3747	1.28	0.36	0.24	0.89
YJL075C		1275	996	1.28	0.36	0.24	0.89
YDR401W		1154	901	1.28	0.36	0.24	0.89
YDR064W	RPS13	34465	26923	1.28	0.36	0.24	0.89
YMR063W	RIM9	2254	1761	1.28	0.36	0.24	0.89
YDR179C		3891	3040	1.28	0.36	0.24	0.89
YPL036W	PMA2	5198	4062	1.28	0.36	0.24	0.88
YJL207C		697	545	1.28	0.36	0.24	0.88
YFR055W		10005	7822	1.28	0.36	0.24	0.88
YOR250C	CLP1	2439	1907	1.28	0.35	0.24	0.88
YFL012W		416	325	1.28	0.35	0.24	0.88
YOR319W	HSH49	6328	4949	1.28	0.35	0.24	0.88
YER067W		4770	3731	1.28	0.35	0.24	0.88
YOL034W		2897	2266	1.28	0.35	0.24	0.88
YMR247C		372	291	1.28	0.35	0.24	0.88
YHR026W	PPA1	4379	3427	1.28	0.35	0.24	0.88

YLR373C		4431	3468	1.28	0.35	0.24	0.88
YLR256W	HAP1	2477	1939	1.28	0.35	0.24	0.88
YBL087C	RPL23A	10715	8388	1.28	0.35	0.24	0.88
YDR006C	SOK1	1479	1158	1.28	0.35	0.24	0.88
YKR037C	SPC34	8651	6773	1.28	0.35	0.24	0.87
YDR026C		6295	4929	1.28	0.35	0.24	0.87
YFR047C		678	531	1.28	0.35	0.24	0.87
YKL079W	SMY1	3601	2821	1.28	0.35	0.24	0.87
YGL068W		9597	7519	1.28	0.35	0.24	0.87
YNR045W	PET494	3826	2998	1.28	0.35	0.24	0.87
YJR003C		2264	1774	1.28	0.35	0.24	0.87
YER124C		20139	15784	1.28	0.35	0.24	0.87
YML049C	RSE1	957	750	1.28	0.35	0.24	0.87
YER113C		12618	9891	1.28	0.35	0.24	0.87
YPL201C		362	284	1.28	0.35	0.24	0.87
YOR273C		610	478	1.28	0.35	0.24	0.87
YPR184W		2607	2044	1.28	0.35	0.24	0.87
YFR045W		2377	1864	1.28	0.35	0.24	0.87
YKL045W	PRI2	4977	3903	1.28	0.35	0.24	0.87
YKL021C	MAK11	13471	10565	1.28	0.35	0.24	0.87
YPL233W		6403	5022	1.28	0.35	0.24	0.87
YML017W	PSP2	511	401	1.28	0.35	0.24	0.87
YJL155C	FBP26	1192	935	1.27	0.35	0.24	0.87
YHR048W		16269	12761	1.27	0.35	0.24	0.87
YKR101W	SIR1	1966	1542	1.27	0.35	0.24	0.87
YJR110W		2664	2090	1.27	0.35	0.24	0.87
YGR160W		5297	4156	1.27	0.35	0.24	0.86
YKL036C		2313	1815	1.27	0.35	0.24	0.86
YPR089W		1861	1460	1.27	0.35	0.24	0.86
YDR333C		826	648	1.27	0.35	0.24	0.86
YDR184C	ATC1	26604	20879	1.27	0.35	0.24	0.86
YPL283C	YRF1-7	3188	2502	1.27	0.35	0.24	0.86
YFR013W		4327	3396	1.27	0.35	0.24	0.86
YBR236C	ABD1	3996	3137	1.27	0.35	0.24	0.86
YHR139C	SPS100	1828	1435	1.27	0.35	0.24	0.86
YDL033C		2272	1784	1.27	0.35	0.24	0.86

YGR097W	ASK10	2232	1753	1.27	0.35	0.24	0.86
YNR033W	ABZ1	5243	4117	1.27	0.35	0.24	0.86
YAL038W	CDC19	83318	65435	1.27	0.35	0.24	0.86
YNL227C		3821	3001	1.27	0.35	0.24	0.86
YJR053W	BFA1	555	436	1.27	0.35	0.24	0.86
YCR020C	PET18	8526	6698	1.27	0.35	0.24	0.86
YJL089W	SIP4	684	537	1.27	0.35	0.24	0.86
YBR034C	HMT1	20592	16178	1.27	0.35	0.24	0.86
YKL083W		369	290	1.27	0.35	0.24	0.86
YBL103C	RTG3	2484	1952	1.27	0.35	0.24	0.86
YGL218W		4306	3385	1.27	0.35	0.23	0.85
YLR358C		1429	1123	1.27	0.35	0.23	0.85
YIL008W		11445	8999	1.27	0.35	0.23	0.85
YPR019W	CDC54	3628	2853	1.27	0.35	0.23	0.85
YLR347C	KAP95	7435	5847	1.27	0.35	0.23	0.85
YMR121C	RPL15B	25059	19709	1.27	0.35	0.23	0.85
YDL129W		2813	2213	1.27	0.35	0.23	0.85
YML026C	RPS18B	13476	10601	1.27	0.35	0.23	0.85
YOR138C		3081	2424	1.27	0.35	0.23	0.85
YPL281C	ERR2	1686	1326	1.27	0.35	0.23	0.85
YNR031C	SSK2	1757	1382	1.27	0.35	0.23	0.85
YLR383W	RHC18	1683	1324	1.27	0.35	0.23	0.85
YKL010C	UFD4	7329	5768	1.27	0.35	0.23	0.85
YNL258C		2786	2193	1.27	0.35	0.23	0.85
YOL022C		11739	9241	1.27	0.35	0.23	0.85
YJR062C	NTA1	2257	1777	1.27	0.34	0.23	0.85
YDR221W		1773	1396	1.27	0.34	0.23	0.85
YOL153C		1128	888	1.27	0.34	0.23	0.85
YPL209C	IPL1	1572	1238	1.27	0.34	0.23	0.84
YJR094W-A	RPL43B	1481	1166	1.27	0.34	0.23	0.84
YML082W		4405	3469	1.27	0.34	0.23	0.84
YDL025C		1601	1261	1.27	0.34	0.23	0.84
YMR061W	RNA14	16984	13379	1.27	0.34	0.23	0.84
YDL035C	GPR1	835	658	1.27	0.34	0.23	0.84
YGL257C	MNT2	407	321	1.27	0.34	0.23	0.84
YOR349W	CIN1	2921	2302	1.27	0.34	0.23	0.84

YOR038C	HIR2	1664	1311	1.27	0.34	0.23	0.84
YOL056W	GPM3	4282	3374	1.27	0.34	0.23	0.84
YNR011C	PRP2	1874	1477	1.27	0.34	0.23	0.84
YHR010W	RPL27A	30704	24199	1.27	0.34	0.23	0.84
YIL173W	VTH1	2570	2026	1.27	0.34	0.23	0.84
YDR053W		1055	832	1.27	0.34	0.23	0.84
YKL099C		20189	15922	1.27	0.34	0.23	0.84
YKR067W		3271	2580	1.27	0.34	0.23	0.84
YLR287C		7301	5759	1.27	0.34	0.23	0.84
YKR056W	RNC1	2267	1788	1.27	0.34	0.23	0.84
YPL025C		976	770	1.27	0.34	0.23	0.84
YIL009W	FAA3	15951	12588	1.27	0.34	0.23	0.83
YOL130W	ALR1	1820	1436	1.27	0.34	0.23	0.83
YBR124W		536	423	1.27	0.34	0.23	0.83
YLR057W		3681	2906	1.27	0.34	0.23	0.83
YMR014W		9795	7732	1.27	0.34	0.23	0.83
YBR274W	CHK1	3707	2927	1.27	0.34	0.23	0.83
YER044C-A	MEI4	774	611	1.27	0.34	0.23	0.83
YLR088W	GAA1	1098	867	1.27	0.34	0.23	0.83
YGL144C		480	379	1.27	0.34	0.23	0.83
YOR178C	GAC1	2741	2165	1.27	0.34	0.23	0.83
YDL138W	RGT2	1854	1465	1.27	0.34	0.23	0.83
YHL027W	RIM101	6886	5442	1.27	0.34	0.23	0.83
YHR127W	HSN1	1442	1140	1.27	0.34	0.23	0.82
YCL047C		524	414	1.26	0.34	0.23	0.82
YDL093W	PMT5	928	734	1.26	0.34	0.23	0.82
YIL150C	DNA43	1010	799	1.26	0.34	0.23	0.82
YMR075C-A		4576	3619	1.26	0.34	0.23	0.82
YDR491C		2890	2286	1.26	0.34	0.23	0.82
YDL150W	RPC53	3236	2560	1.26	0.34	0.23	0.82
YJL093C	TOK1	2879	2278	1.26	0.34	0.23	0.82
YMR142C	RPL13B	14299	11315	1.26	0.34	0.23	0.82
YBR023C	CHS3	4029	3188	1.26	0.34	0.23	0.82
YKR103W		752	595	1.26	0.34	0.23	0.82
YNR077C		551	436	1.26	0.34	0.23	0.82
YMR166C		4444	3518	1.26	0.34	0.22	0.82

YIL141W		1196	947	1.26	0.34	0.22	0.82
YKL216W	URA1	13282	10516	1.26	0.34	0.22	0.82
YOR274W	MOD5	3385	2680	1.26	0.34	0.22	0.82
YJL113W		3483	2758	1.26	0.34	0.22	0.82
YLR393W	ATP10	1222	968	1.26	0.34	0.22	0.81
YOR328W	PDR10	1733	1373	1.26	0.34	0.22	0.81
YLR335W	NUP2	2723	2157	1.26	0.34	0.22	0.81
YDR104C		2622	2077	1.26	0.34	0.22	0.81
YFL015C		774	613	1.26	0.34	0.22	0.81
YFR019W	FAB1	1646	1304	1.26	0.34	0.22	0.81
YGL167C	PMR1	444	352	1.26	0.34	0.22	0.81
YDR243C	PRP28	967	766	1.26	0.34	0.22	0.81
YBL109W		1654	1311	1.26	0.34	0.22	0.81
YBL028C		18585	14731	1.26	0.34	0.22	0.81
YKL132C	RMA1	1243	985	1.26	0.34	0.22	0.81
YGR188C	BUB1	1550	1229	1.26	0.34	0.22	0.81
YHL013C		16640	13193	1.26	0.33	0.22	0.81
YGL217C		1400	1110	1.26	0.33	0.22	0.81
YOR335C	ALA1	7632	6052	1.26	0.33	0.22	0.81
YLR204W	QRI5	8740	6932	1.26	0.33	0.22	0.81
YJR150C	DAN1	1844	1463	1.26	0.33	0.22	0.81
YER025W	GCD11	11620	9219	1.26	0.33	0.22	0.81
YDR505C	PSP1	2149	1705	1.26	0.33	0.22	0.81
YBR083W	TEC1	8543	6780	1.26	0.33	0.22	0.80
YJR138W	IML1	514	408	1.26	0.33	0.22	0.80
YMR134W		4031	3200	1.26	0.33	0.22	0.80
YDR499W		4738	3762	1.26	0.33	0.22	0.80
YHR215W	PHO12	29884	23728	1.26	0.33	0.22	0.80
YPR007C	SPO69	528	419	1.26	0.33	0.22	0.80
YDL171C	GLT1	2247	1785	1.26	0.33	0.22	0.80
YIL110W		7319	5814	1.26	0.33	0.22	0.80
YMR075W		1479	1175	1.26	0.33	0.22	0.80
YPL282C		1638	1301	1.26	0.33	0.22	0.80
YCRX12W		969	770	1.26	0.33	0.22	0.80
YLR182W	SWI6	3511	2789	1.26	0.33	0.22	0.80
YLL043W	FPS1	8608	6839	1.26	0.33	0.22	0.80

YLR238W		3224	2561	1.26	0.33	0.22	0.80
YLR392C		1794	1425	1.26	0.33	0.22	0.80
YDL238C		1949	1549	1.26	0.33	0.22	0.80
YHR024C	MAS2	7383	5867	1.26	0.33	0.22	0.80
YBR142W	MAK5	8276	6577	1.26	0.33	0.22	0.80
YIL144W	TID3	3616	2874	1.26	0.33	0.22	0.80
YBR284W		2187	1739	1.26	0.33	0.22	0.79
YDR312W	SSF2	10853	8629	1.26	0.33	0.22	0.79
YDR211W	GCD6	3146	2501	1.26	0.33	0.22	0.79
YOL154W		4757	3783	1.26	0.33	0.22	0.79
YHR214W		1788	1422	1.26	0.33	0.22	0.79
YCR091W	KIN82	1191	947	1.26	0.33	0.22	0.79
YBR153W	RIB7	6919	5504	1.26	0.33	0.22	0.79
YDR531W		14614	11626	1.26	0.33	0.22	0.79
YKL172W	EBP2	6910	5498	1.26	0.33	0.22	0.79
YKL158W		686	546	1.26	0.33	0.22	0.79
YMR240C	CUS1	1137	905	1.26	0.33	0.22	0.79
YGL147C	RPL9A	36030	28669	1.26	0.33	0.22	0.79
YJL077C	ICS3	997	793	1.26	0.33	0.22	0.79
YOR110W		1914	1523	1.26	0.33	0.22	0.79
YOR058C	ASE1	1430	1138	1.26	0.33	0.22	0.79
YGL110C		3228	2569	1.26	0.33	0.22	0.79
YOL138C		2402	1912	1.26	0.33	0.22	0.79
YBR134W		1456	1159	1.26	0.33	0.22	0.79
YKL120W	OAC1	2152	1713	1.26	0.33	0.22	0.79
YDR166C	SEC5	1129	899	1.26	0.33	0.22	0.79
YMR005W	MPT1	12466	9925	1.26	0.33	0.22	0.79
YBR184W		819	652	1.26	0.33	0.22	0.79
YDR415C		7229	5756	1.26	0.33	0.22	0.79
YLR134W	PDC5	23948	19070	1.26	0.33	0.22	0.79
YNL033W		602	479	1.26	0.33	0.22	0.79
YLR098C	CHA4	3114	2480	1.26	0.33	0.22	0.79
YJL127C	SPT10	1200	956	1.26	0.33	0.22	0.78
YGR065C	VHT1	8610	6858	1.26	0.33	0.22	0.78
YKR080W	MTD1	4503	3587	1.26	0.33	0.22	0.78
YDR193W		1690	1346	1.26	0.33	0.22	0.78

YHR129C	ARP1	1597	1272	1.26	0.33	0.22	0.78
YER157W	SEC34	7032	5603	1.26	0.33	0.22	0.78
YKR051W		3748	2988	1.25	0.33	0.21	0.78
YBR039W	ATP3	21594	17214	1.25	0.33	0.21	0.78
YNL298W	CLA4	2664	2124	1.25	0.33	0.21	0.78
YDR461W	MFA1	912	727	1.25	0.33	0.21	0.78
YEL064C		1288	1027	1.25	0.33	0.21	0.78
YDR495C	VPS3	878	700	1.25	0.33	0.21	0.78
YHR054C		13635	10878	1.25	0.33	0.21	0.78
YDL211C		1158	924	1.25	0.33	0.21	0.77
YGR020C	VMA7	11985	9564	1.25	0.33	0.21	0.77
YLR344W	RPL26A	14100	11252	1.25	0.33	0.21	0.77
YCR096C	A2	15269	12185	1.25	0.33	0.21	0.77
YLR028C	ADE16	12904	10298	1.25	0.33	0.21	0.77
YJR093C	FIP1	2831	2260	1.25	0.33	0.21	0.77
YOL024W		807	644	1.25	0.32	0.21	0.77
YOR337W	TEA1	7671	6126	1.25	0.32	0.21	0.77
YIL163C		1040	831	1.25	0.32	0.21	0.77
YLL017W		802	641	1.25	0.32	0.21	0.77
YFL002C	SPB4	11249	8987	1.25	0.32	0.21	0.77
YKL168C	KKQ8	1334	1066	1.25	0.32	0.21	0.77
YPL187W	MF(ALPHA)1	18189	14532	1.25	0.32	0.21	0.77
YDR037W	KRS1	10793	8624	1.25	0.32	0.21	0.77
YJR036C		636	508	1.25	0.32	0.21	0.77
YLR176C	RFX1	1537	1228	1.25	0.32	0.21	0.77
YFR035C		1817	1452	1.25	0.32	0.21	0.77
YOL045W		841	672	1.25	0.32	0.21	0.77
YER119C-A		2689	2150	1.25	0.32	0.21	0.76
YCR099C		1631	1304	1.25	0.32	0.21	0.76
YIL071C		2053	1642	1.25	0.32	0.21	0.76
YNL094W		4879	3903	1.25	0.32	0.21	0.76
YLR024C		759	607	1.25	0.32	0.21	0.76
YDL184C	RPL41A	23636	18910	1.25	0.32	0.21	0.76
YDR489W		6138	4911	1.25	0.32	0.21	0.76
YOR379C		1107	886	1.25	0.32	0.21	0.76
YGL149W		1355	1084	1.25	0.32	0.21	0.76

YDL133W		4772	3819	1.25	0.32	0.21	0.76
YML084W		828	663	1.25	0.32	0.21	0.76
YLR295C	ATP14	15187	12155	1.25	0.32	0.21	0.76
YOL114C		1595	1277	1.25	0.32	0.21	0.76
YNL151C	RPC31	7214	5775	1.25	0.32	0.21	0.76
YNR057C	BIO4	1347	1078	1.25	0.32	0.21	0.76
YNL308C	KRI1	8294	6640	1.25	0.32	0.21	0.76
YAR062W		1042	834	1.25	0.32	0.21	0.76
YPL147W	PXA1	7796	6242	1.25	0.32	0.21	0.76
YOL025W	LAG2	2469	1977	1.25	0.32	0.21	0.76
YFR026C		662	530	1.25	0.32	0.21	0.76
YBR041W	FAT1	4141	3317	1.25	0.32	0.21	0.75
YDR091C	RLI1	13594	10891	1.25	0.32	0.21	0.75
YLL037W		4018	3219	1.25	0.32	0.21	0.75
YOR284W		5966	4780	1.25	0.32	0.21	0.75
YDR123C	INO2	9020	7227	1.25	0.32	0.21	0.75
YLL041C	SDH2	15363	12310	1.25	0.32	0.21	0.75
YDR261C	EXG2	1560	1250	1.25	0.32	0.21	0.75
YDR493W		9315	7466	1.25	0.32	0.21	0.75
YNL216W	RAP1	10331	8282	1.25	0.32	0.21	0.75
YLR371W	ROM2	2008	1610	1.25	0.32	0.21	0.75
YDR022C	CIS1	4525	3628	1.25	0.32	0.21	0.75
YCLX03C		511	410	1.25	0.32	0.21	0.75
YPL039W		2860	2294	1.25	0.32	0.21	0.75
YLR130C	ZRT2	4483	3596	1.25	0.32	0.21	0.75
YDR514C		2556	2051	1.25	0.32	0.21	0.75
YLL032C		2865	2299	1.25	0.32	0.21	0.75
YMR282C	AEP2	4107	3295	1.25	0.32	0.21	0.75
YGL162W	SUT1	2596	2083	1.25	0.32	0.21	0.75
YJL045W		1284	1030	1.25	0.32	0.21	0.75
YKL102C		422	339	1.25	0.32	0.20	0.75
YGL211W		5038	4044	1.25	0.32	0.20	0.74
YKL071W		1557	1250	1.25	0.32	0.20	0.74
YLR385C		995	799	1.25	0.32	0.20	0.74
YGR117C		3888	3122	1.25	0.32	0.20	0.74
YBR079C	RPG1	14638	11755	1.25	0.32	0.20	0.74

YMR293C		2236	1796	1.25	0.32	0.20	0.74
YHL045W		811	651	1.25	0.32	0.20	0.74
YDR095C		1368	1099	1.25	0.32	0.20	0.74
YJR049C	UTR1	1578	1267	1.25	0.32	0.20	0.74
YDL061C	RPS29B	19137	15370	1.25	0.32	0.20	0.74
YKL049C	CSE4	5668	4553	1.24	0.32	0.20	0.74
YBR276C	PPS1	1058	850	1.24	0.32	0.20	0.74
YGR023W	MTL1	3215	2583	1.24	0.32	0.20	0.74
YAL051W	YAF1	779	626	1.24	0.32	0.20	0.74
YLR106C		2029	1630	1.24	0.32	0.20	0.74
YBR140C	IRA1	1736	1395	1.24	0.32	0.20	0.74
YGR115C		1027	825	1.24	0.32	0.20	0.74
YIL160C	POT1	1470	1181	1.24	0.32	0.20	0.74
YBR174C		1580	1270	1.24	0.32	0.20	0.74
YPR035W	GLN1	28617	22998	1.24	0.32	0.20	0.74
YBR086C	IST2	13944	11207	1.24	0.32	0.20	0.74
YNL029C	KTR5	1770	1423	1.24	0.32	0.20	0.74
YGR043C		1604	1289	1.24	0.32	0.20	0.74
YPL119C	DBP1	636	511	1.24	0.31	0.20	0.74
YFL050C	ALR2	2126	1709	1.24	0.31	0.20	0.74
YNR005C		1848	1486	1.24	0.31	0.20	0.74
YBR090C		9281	7462	1.24	0.31	0.20	0.74
YBL067C	UBP13	1846	1484	1.24	0.31	0.20	0.73
YFR017C		1295	1041	1.24	0.31	0.20	0.73
YFR001W		17491	14066	1.24	0.31	0.20	0.73
YEL006W		5375	4324	1.24	0.31	0.20	0.73
YNL286W	CUS2	2975	2393	1.24	0.31	0.20	0.73
YJL069C		7724	6214	1.24	0.31	0.20	0.73
YBL009W		2807	2258	1.24	0.31	0.20	0.73
YDR339C		9553	7686	1.24	0.31	0.20	0.73
YML025C		6595	5306	1.24	0.31	0.20	0.73
YLL033W		2515	2024	1.24	0.31	0.20	0.73
YOR363C	PIP2	1613	1298	1.24	0.31	0.20	0.73
YKL186C	MTR2	7214	5805	1.24	0.31	0.20	0.73
YCR094W	CDC50	11947	9615	1.24	0.31	0.20	0.73
YAL037W		491	395	1.24	0.31	0.20	0.73

YHR052W		3677	2960	1.24	0.31	0.20	0.73
YDR082W	STN1	3296	2653	1.24	0.31	0.20	0.73
YIL099W	SGA1	843	679	1.24	0.31	0.20	0.73
YOR024W		6212	5001	1.24	0.31	0.20	0.73
YDR500C	RPL37B	14935	12024	1.24	0.31	0.20	0.73
YNL002C	RLP7	40329	32471	1.24	0.31	0.20	0.73
YPR058W	YMC1	6704	5398	1.24	0.31	0.20	0.73
YKR040C		1817	1463	1.24	0.31	0.20	0.73
YML039W		34104	27463	1.24	0.31	0.20	0.73
YOL004W	SIN3	2380	1917	1.24	0.31	0.20	0.73
YDR342C	HXT7	19982	16096	1.24	0.31	0.20	0.73
YBR294W	SUL1	1378	1110	1.24	0.31	0.20	0.73
YOL066C	RIB2	2931	2361	1.24	0.31	0.20	0.73
YGR199W	PMT6	5617	4525	1.24	0.31	0.20	0.73
YPR032W	SRO7	3307	2664	1.24	0.31	0.20	0.73
YDR397C	NCB2	17055	13741	1.24	0.31	0.20	0.72
YPL275W		930	749	1.24	0.31	0.20	0.72
YGR105W	VMA21	633	510	1.24	0.31	0.20	0.72
YML010W-B		1306	1052	1.24	0.31	0.20	0.72
YDR152W		15784	12719	1.24	0.31	0.20	0.72
YJR131W	MNS1	1061	855	1.24	0.31	0.20	0.72
YPL115C	BEM3	2211	1782	1.24	0.31	0.20	0.72
YDL011C		2340	1887	1.24	0.31	0.20	0.72
YHR003C		8518	6869	1.24	0.31	0.20	0.72
YNL136W		5366	4327	1.24	0.31	0.20	0.72
YBR009C	HHF1	23489	18942	1.24	0.31	0.20	0.72
YJR012C		1841	1485	1.24	0.31	0.20	0.72
YDR276C		1054	850	1.24	0.31	0.20	0.72
YHL005C		2268	1829	1.24	0.31	0.20	0.72
YOL132W		387	312	1.24	0.31	0.20	0.72
YMR215W		1374	1108	1.24	0.31	0.20	0.72
YCR024C-A	PMP1	29217	23566	1.24	0.31	0.20	0.72
YCR016W		27099	21863	1.24	0.31	0.20	0.72
YIR008C	PRI1	12082	9748	1.24	0.31	0.20	0.72
YBR059C	AKL1	3065	2473	1.24	0.31	0.20	0.72
YGL251C	HFM1	1802	1454	1.24	0.31	0.20	0.72

YJR056C		4805	3878	1.24	0.31	0.20	0.72
YDL113C		1295	1045	1.24	0.31	0.20	0.71
YER006W		19103	15421	1.24	0.31	0.20	0.71
YBR003W	COQ1	3218	2598	1.24	0.31	0.20	0.71
YLR255C		633	511	1.24	0.31	0.20	0.71
YDL044C	MTF2	2491	2011	1.24	0.31	0.20	0.71
YOR086C		1199	968	1.24	0.31	0.20	0.71
YPL251W		6688	5400	1.24	0.31	0.20	0.71
YHL043W	ECM34	1513	1222	1.24	0.31	0.20	0.71
YDR048C		1683	1359	1.24	0.31	0.20	0.71
YOR305W		11068	8938	1.24	0.31	0.20	0.71
YDR009W	GAL3	539	435	1.24	0.31	0.20	0.71
YLR405W		8638	6977	1.24	0.31	0.20	0.71
YOL118C		2822	2279	1.24	0.31	0.20	0.71
YBR033W		5666	4577	1.24	0.31	0.20	0.71
YHL026C		2530	2044	1.24	0.31	0.20	0.71
YOR200W		2250	1818	1.24	0.31	0.20	0.71
YML089C		470	380	1.24	0.31	0.20	0.71
YDR278C		2282	1844	1.24	0.31	0.19	0.71
YGR292W	MAL12	1364	1103	1.24	0.31	0.19	0.71
YNL230C	ELA1	1572	1271	1.24	0.31	0.19	0.71
YIL025C		714	577	1.24	0.31	0.19	0.71
YPL063W		7848	6346	1.24	0.31	0.19	0.71
YMR313C		1501	1214	1.24	0.31	0.19	0.70
YJL130C	URA2	6452	5219	1.24	0.31	0.19	0.70
YCRX08W		2779	2248	1.24	0.31	0.19	0.70
YOL065C	INP54	4198	3396	1.24	0.31	0.19	0.70
YPR044C		1072	867	1.24	0.31	0.19	0.70
YNL224C		4299	3478	1.24	0.31	0.19	0.70
YBR148W	YSW1	373	302	1.24	0.31	0.19	0.70
YIL085C	KTR7	4503	3644	1.24	0.31	0.19	0.70
YJR160C		1106	895	1.24	0.31	0.19	0.70
YJR048W	CYC1	17579	14226	1.24	0.31	0.19	0.70
YDR197W	CBS2	3240	2622	1.24	0.31	0.19	0.70
YJR108W	ABM1	1530	1238	1.24	0.31	0.19	0.70
YLR243W		11705	9473	1.24	0.31	0.19	0.70

YMR015C	ERG5	30874	24988	1.24	0.31	0.19	0.70
YIL095W	PRK1	6957	5632	1.24	0.30	0.19	0.70
YBR226C		1342	1087	1.24	0.30	0.19	0.70
YJR121W	ATP2	5124	4149	1.23	0.30	0.19	0.70
YLR210W	CLB4	2705	2191	1.23	0.30	0.19	0.70
YDL227C	HO	919	744	1.23	0.30	0.19	0.70
YBL013W		510	413	1.23	0.30	0.19	0.70
YJR080C		6208	5028	1.23	0.30	0.19	0.70
YKL022C	CDC16	1720	1393	1.23	0.30	0.19	0.70
YFR012W		1107	897	1.23	0.30	0.19	0.70
YDR541C		1779	1441	1.23	0.30	0.19	0.70
YDL109C		2007	1626	1.23	0.30	0.19	0.70
YER172C	BRR2	2487	2015	1.23	0.30	0.19	0.69
YLR334C		634	514	1.23	0.30	0.19	0.69
YDR275W		3500	2836	1.23	0.30	0.19	0.69
YGL156W	AMS1	2396	1942	1.23	0.30	0.19	0.69
YGR054W		14234	11536	1.23	0.30	0.19	0.69
YKL220C	FRE2	684	554	1.23	0.30	0.19	0.69
YCR010C		1795	1455	1.23	0.30	0.19	0.69
YOL020W	TAT2	12380	10036	1.23	0.30	0.19	0.69
YER188W		2021	1638	1.23	0.30	0.19	0.69
YPR043W	RPL43A	13105	10624	1.23	0.30	0.19	0.69
YER190W	YRF1-2	10946	8875	1.23	0.30	0.19	0.69
YDR357C		9388	7612	1.23	0.30	0.19	0.69
YPL181W		5601	4542	1.23	0.30	0.19	0.69
YMR172W	HOT1	1232	999	1.23	0.30	0.19	0.69
YCR021C	HSP30	2689	2181	1.23	0.30	0.19	0.69
YOL095C	HMI1	2649	2149	1.23	0.30	0.19	0.69
YNL170W		3885	3152	1.23	0.30	0.19	0.69
YBR180W		1805	1464	1.23	0.30	0.19	0.69
YDL158C		839	681	1.23	0.30	0.19	0.69
YGL111W		20893	16954	1.23	0.30	0.19	0.69
YPR115W		811	658	1.23	0.30	0.19	0.69
YPR056W	TFB4	6574	5337	1.23	0.30	0.19	0.68
YPL273W		1204	978	1.23	0.30	0.19	0.68
YLR214W	FRE1	15608	12675	1.23	0.30	0.19	0.68

YNL264C	PDR17	4725	3837	1.23	0.30	0.19	0.68
YLR430W	SEN1	1338	1087	1.23	0.30	0.19	0.68
YOR373W	NUD1	7224	5867	1.23	0.30	0.19	0.68
YNL201C		865	703	1.23	0.30	0.19	0.68
YDR473C	PRP3	3739	3038	1.23	0.30	0.19	0.68
YNR015W	SMM1	7373	5991	1.23	0.30	0.19	0.68
YKL115C		2231	1813	1.23	0.30	0.19	0.68
YEL032W	MCM3	1059	861	1.23	0.30	0.19	0.68
YDR369C	XRS2	2597	2111	1.23	0.30	0.19	0.68
YGR095C	RRP46	7414	6026	1.23	0.30	0.19	0.68
YPL065W	VPS28	2566	2086	1.23	0.30	0.19	0.68
YBR036C	CSG2	478	389	1.23	0.30	0.19	0.68
YDR271C		846	688	1.23	0.30	0.19	0.68
YJL114W		1042	847	1.23	0.30	0.19	0.68
YGR011W		2786	2266	1.23	0.30	0.19	0.68
YER104W	RTT105	2174	1768	1.23	0.30	0.19	0.67
YMR254C		817	665	1.23	0.30	0.19	0.67
YER107C	GLE2	15169	12340	1.23	0.30	0.19	0.67
YPL086C	ELP3	16053	13060	1.23	0.30	0.19	0.67
YOR011W		1230	1001	1.23	0.30	0.19	0.67
YPR132W	RPS23B	20897	17002	1.23	0.30	0.19	0.67
YJL157C	FAR1	1241	1010	1.23	0.30	0.19	0.67
YGR239C	PEX21	3732	3037	1.23	0.30	0.18	0.67
YNL059C	ARP5	8857	7208	1.23	0.30	0.18	0.67
YJR051W	OSM1	1068	869	1.23	0.30	0.18	0.67
YKL039W	PTM1	1735	1412	1.23	0.30	0.18	0.67
YNL172W	APC1	2450	1994	1.23	0.30	0.18	0.67
YDR315C	IPK1	2674	2177	1.23	0.30	0.18	0.67
YJR154W		472	384	1.23	0.30	0.18	0.67
YPL137C		472	384	1.23	0.30	0.18	0.67
YGR073C		6204	5051	1.23	0.30	0.18	0.67
YPR110C	RPC40	31737	25841	1.23	0.30	0.18	0.67
YGL212W	VAM7	2753	2242	1.23	0.30	0.18	0.67
YLR141W	RRN5	1739	1416	1.23	0.30	0.18	0.67
YMR212C		977	796	1.23	0.30	0.18	0.67
YLR193C		32130	26172	1.23	0.30	0.18	0.67

YBR131W	CCZ1	2455	2000	1.23	0.30	0.18	0.67
YCR039C	MATALPHA2	20869	17000	1.23	0.30	0.18	0.67
YGL197W	MDS3	2112	1721	1.23	0.30	0.18	0.67
YOR338W		528	430	1.23	0.30	0.18	0.67
YCL060C		1031	840	1.23	0.30	0.18	0.66
YDR005C	MAF1	4489	3659	1.23	0.30	0.18	0.66
YBR001C	NTH2	682	556	1.23	0.30	0.18	0.66
YDL216C		1435	1170	1.23	0.29	0.18	0.66
YIL054W		813	663	1.23	0.29	0.18	0.66
YOR094W	ARF3	4287	3495	1.23	0.29	0.18	0.66
YNL015W	PBI2	6886	5614	1.23	0.29	0.18	0.66
YNL232W	CSL4	19068	15546	1.23	0.29	0.18	0.66
YML073C	RPL6A	6461	5268	1.23	0.29	0.18	0.66
YHR027C	RPN1	5553	4528	1.23	0.29	0.18	0.66
YHR217C		1049	855	1.23	0.29	0.18	0.66
YNL158W		8004	6527	1.23	0.29	0.18	0.66
YNL018C		2108	1719	1.23	0.29	0.18	0.66
YFR005C	SAD1	7453	6078	1.23	0.29	0.18	0.66
YHR171W	APG7	1725	1407	1.23	0.29	0.18	0.66
YFL040W		559	456	1.23	0.29	0.18	0.66
YLR319C	BUD6	5765	4702	1.23	0.29	0.18	0.66
YPL011C	TAF47	10221	8337	1.23	0.29	0.18	0.66
YBR088C	POL30	8567	6988	1.23	0.29	0.18	0.66
YDL037C		403	329	1.23	0.29	0.18	0.66
YLL018C-A	COX19	12242	9988	1.23	0.29	0.18	0.66
YLR411W	CTR3	1232	1005	1.23	0.29	0.18	0.66
YKR035C		12819	10460	1.23	0.29	0.18	0.66
YHR107C	CDC12	8353	6816	1.23	0.29	0.18	0.66
YDL213C		5039	4112	1.23	0.29	0.18	0.66
YGR112W	SHY1	7422	6057	1.23	0.29	0.18	0.66
YPR015C		591	482	1.23	0.29	0.18	0.66
YLR113W	HOG1	17453	14249	1.22	0.29	0.18	0.66
YAR007C	RFA1	3776	3083	1.22	0.29	0.18	0.65
YGR100W	MDR1	3858	3150	1.22	0.29	0.18	0.65
YCL006C		2269	1853	1.22	0.29	0.18	0.65
YBR208C	DUR1,2	811	662	1.22	0.29	0.18	0.65

YKL148C	SDH1	5824	4756	1.22	0.29	0.18	0.65
YBL037W	APL3	1878	1534	1.22	0.29	0.18	0.65
YDR427W	RPN9	1830	1495	1.22	0.29	0.18	0.65
YML096W		2897	2366	1.22	0.29	0.18	0.65
YLR312C		1055	862	1.22	0.29	0.18	0.65
YDL121C		5107	4173	1.22	0.29	0.18	0.65
YIL133C	RPL16A	22422	18321	1.22	0.29	0.18	0.65
YJL175W		1774	1450	1.22	0.29	0.18	0.65
YMR159C	APG16	1446	1182	1.22	0.29	0.18	0.65
YAL056W		1668	1363	1.22	0.29	0.18	0.65
YNR013C		5137	4199	1.22	0.29	0.18	0.65
YFL013W-A		1013	828	1.22	0.29	0.18	0.65
YML047C		500	409	1.22	0.29	0.18	0.65
YNL105W		779	637	1.22	0.29	0.18	0.65
YJL026W	RNR2	12638	10332	1.22	0.29	0.18	0.65
YER151C	UBP3	535	437	1.22	0.29	0.18	0.65
YEL057C		651	532	1.22	0.29	0.18	0.65
YDL136W	RPL35B	17396	14223	1.22	0.29	0.18	0.65
YCR051W		11263	9209	1.22	0.29	0.18	0.65
YGR071C		2525	2065	1.22	0.29	0.18	0.65
YIL119C	RPI1	6239	5103	1.22	0.29	0.18	0.65
YPR141C	KAR3	5716	4675	1.22	0.29	0.18	0.65
YGL246C	RAI1	17464	14285	1.22	0.29	0.18	0.65
YBR299W	MAL32	3446	2819	1.22	0.29	0.18	0.65
YPL278C		1040	851	1.22	0.29	0.18	0.65
YNL073W	MSK1	5818	4760	1.22	0.29	0.18	0.64
YGL086W	MAD1	2051	1678	1.22	0.29	0.18	0.64
YOR360C	PDE2	7359	6022	1.22	0.29	0.18	0.64
YLL063C	AYT1	2867	2346	1.22	0.29	0.18	0.64
YHR212C		644	527	1.22	0.29	0.18	0.64
YOL131W		448	367	1.22	0.29	0.18	0.64
YOR364W		585	479	1.22	0.29	0.18	0.64
YDR187C		2007	1643	1.22	0.29	0.18	0.64
YAL011W		1720	1408	1.22	0.29	0.18	0.64
YPL146C		17099	14000	1.22	0.29	0.18	0.64
YJR130C		5180	4241	1.22	0.29	0.18	0.64

YNL019C		800	655	1.22	0.29	0.18	0.64
YMR047C	NUP116	8716	7139	1.22	0.29	0.18	0.64
YGL055W	OLE1	16726	13704	1.22	0.29	0.18	0.64
YOL116W	MSN1	2685	2200	1.22	0.29	0.17	0.64
YJR005W	APL1	2662	2181	1.22	0.29	0.17	0.64
YLL001W	DNM1	2025	1659	1.22	0.29	0.17	0.64
YOR108W		3002	2460	1.22	0.29	0.17	0.64
YPR034W	ARP7	6935	5683	1.22	0.29	0.17	0.64
YKR097W	PCK1	3806	3119	1.22	0.29	0.17	0.64
YIL059C		2122	1739	1.22	0.29	0.17	0.63
YBR177C	EHT1	1486	1218	1.22	0.29	0.17	0.63
YDR102C		1088	892	1.22	0.29	0.17	0.63
YEL045C		1586	1300	1.22	0.29	0.17	0.63
YOR242C	SSP2	4708	3860	1.22	0.29	0.17	0.63
YJR102C		4556	3736	1.22	0.29	0.17	0.63
YJL193W		5557	4557	1.22	0.29	0.17	0.63
YGR047C	TFC4	4041	3314	1.22	0.29	0.17	0.63
YHL009C	YAP3	3543	2906	1.22	0.29	0.17	0.63
YNL086W		1094	897	1.22	0.29	0.17	0.63
YBR048W	RPS11B	3021	2478	1.22	0.29	0.17	0.63
YLR128W		1837	1507	1.22	0.29	0.17	0.63
YJL083W		912	748	1.22	0.29	0.17	0.63
YLR321C	SFH1	8867	7274	1.22	0.29	0.17	0.63
YDL223C		686	563	1.22	0.29	0.17	0.63
YGL134W	PCL10	2372	1946	1.22	0.29	0.17	0.63
YNL225C	CNM67	3373	2768	1.22	0.28	0.17	0.63
YJL099W	CHS6	3943	3236	1.22	0.28	0.17	0.63
YER182W		7769	6377	1.22	0.28	0.17	0.63
YGL186C		9211	7561	1.22	0.28	0.17	0.63
YLL028W	TPO1	8111	6658	1.22	0.28	0.17	0.63
YDR347W	MRP1	23664	19426	1.22	0.28	0.17	0.63
YPR088C	SRP54	8599	7059	1.22	0.28	0.17	0.63
YIL132C		2983	2449	1.22	0.28	0.17	0.63
YLR282C		3005	2467	1.22	0.28	0.17	0.63
YJL074C	SMC3	3517	2888	1.22	0.28	0.17	0.63
YGR287C		4038	3316	1.22	0.28	0.17	0.63

YOR191W	RIS1	4026	3306	1.22	0.28	0.17	0.62
YOL115W	TRF4	3367	2765	1.22	0.28	0.17	0.62
YMR187C		958	787	1.22	0.28	0.17	0.62
YAL065C		634	521	1.22	0.28	0.17	0.62
YDR530C	APA2	578	475	1.22	0.28	0.17	0.62
YJL091C		1679	1379	1.22	0.28	0.17	0.62
YDR535C		934	767	1.22	0.28	0.17	0.62
YJR094C	IME1	689	566	1.22	0.28	0.17	0.62
YJL179W	PFD1	7135	5862	1.22	0.28	0.17	0.62
YJR127C	ZMS1	2729	2242	1.22	0.28	0.17	0.62
YCR089W	FIG2	4934	4054	1.22	0.28	0.17	0.62
YKL110C	KTI12	8293	6814	1.22	0.28	0.17	0.62
YPL151C	PRP46	4111	3379	1.22	0.28	0.17	0.62
YNL221C	POP1	5092	4186	1.22	0.28	0.17	0.62
YOR343C		507	417	1.22	0.28	0.17	0.62
YLR234W	TOP3	2082	1712	1.22	0.28	0.17	0.62
YJR066W	TOR1	2346	1929	1.22	0.28	0.17	0.62
YOR043W	WHI2	10902	8966	1.22	0.28	0.17	0.62
YPL105C		6101	5018	1.22	0.28	0.17	0.62
YEL043W		1504	1237	1.22	0.28	0.17	0.62
YCR044C		4848	3988	1.22	0.28	0.17	0.62
YOR268C		1459	1200	1.22	0.28	0.17	0.62
YER005W	YND1	2621	2156	1.22	0.28	0.17	0.62
YLL054C		2686	2210	1.22	0.28	0.17	0.62
YBR240C	THI2	2924	2406	1.22	0.28	0.17	0.61
YDR088C	SLU7	5736	4720	1.22	0.28	0.17	0.61
YPR021C		2766	2276	1.22	0.28	0.17	0.61
YDL222C		2175	1790	1.22	0.28	0.17	0.61
YER168C	CCA1	12949	10657	1.22	0.28	0.17	0.61
YJL046W		4688	3858	1.22	0.28	0.17	0.61
YBL008W	HIR1	2622	2158	1.22	0.28	0.17	0.61
YIL055C		1485	1222	1.21	0.28	0.17	0.61
YNL272C	SEC2	3482	2866	1.21	0.28	0.17	0.61
YLR082C	SRL2	3408	2806	1.21	0.28	0.17	0.61
YLR052W		5705	4697	1.21	0.28	0.17	0.61
YOL158C		1992	1640	1.21	0.28	0.17	0.61

YCRX09C		816	672	1.21	0.28	0.17	0.61
YOR048C	RAT1	1545	1272	1.21	0.28	0.17	0.61
YOR322C		935	770	1.21	0.28	0.17	0.61
YJR120W		1282	1056	1.21	0.28	0.17	0.61
YPL092W	SSU1	6733	5545	1.21	0.28	0.17	0.61
YDR446W	ECM11	618	509	1.21	0.28	0.17	0.61
YBL053W		1170	964	1.21	0.28	0.17	0.61
YLL030C		617	508	1.21	0.28	0.17	0.61
YDR340W		7999	6591	1.21	0.28	0.17	0.61
YDR403W	DIT1	2518	2075	1.21	0.28	0.17	0.61
YMR111C		4580	3774	1.21	0.28	0.17	0.61
YCR018C	SRD1	809	667	1.21	0.28	0.17	0.61
YJR026W		16962	13980	1.21	0.28	0.17	0.61
YPL198W	RPL7B	27740	22863	1.21	0.28	0.17	0.61
YOR104W		3631	2993	1.21	0.28	0.17	0.60
YMR145C		6982	5755	1.21	0.28	0.17	0.60
YKR078W		1993	1643	1.21	0.28	0.17	0.60
YOR317W	FAA1	4604	3796	1.21	0.28	0.17	0.60
YLR020C		10524	8677	1.21	0.28	0.17	0.60
YBR051W		622	513	1.21	0.28	0.17	0.60
YHR113W		6349	5236	1.21	0.28	0.17	0.60
YHR079C-B		1318	1087	1.21	0.28	0.17	0.60
YHR111W		7198	5937	1.21	0.28	0.17	0.60
YPR150W		1906	1572	1.21	0.28	0.17	0.60
YDR472W	TRS31	9655	7965	1.21	0.28	0.17	0.60
YCL055W	KAR4	13568	11193	1.21	0.28	0.17	0.60
YNL250W	RAD50	1198	988	1.21	0.28	0.17	0.60
YGL004C		2908	2399	1.21	0.28	0.17	0.60
YPL241C	CIN2	6262	5167	1.21	0.28	0.17	0.60
YGL252C	RTG2	12880	10630	1.21	0.28	0.16	0.60
YPL185W		458	378	1.21	0.28	0.16	0.60
YOR355W	GDS1	16153	13333	1.21	0.28	0.16	0.60
YOR070C	GYP1	3012	2486	1.21	0.28	0.16	0.60
YML019W	OST6	5509	4548	1.21	0.28	0.16	0.60
YNL282W	POP3	2573	2124	1.21	0.28	0.16	0.60
YJR052W	RAD7	2481	2049	1.21	0.28	0.16	0.60

YGR261C	APL6	3784	3126	1.21	0.28	0.16	0.59
YJL187C	SWE1	1003	829	1.21	0.28	0.16	0.59
YPR012W		1370	1132	1.21	0.28	0.16	0.59
YLR271W		2012	1663	1.21	0.28	0.16	0.59
YML010W	SPT5	4021	3323	1.21	0.27	0.16	0.59
YDR405W	MRP20	10470	8654	1.21	0.27	0.16	0.59
YGL130W	CEG1	4197	3469	1.21	0.27	0.16	0.59
YGL176C		4354	3599	1.21	0.27	0.16	0.59
YOR233W	KIN4	1377	1138	1.21	0.27	0.16	0.59
YLR047C		893	738	1.21	0.27	0.16	0.59
YDR083W		6238	5157	1.21	0.27	0.16	0.59
YGL124C		651	538	1.21	0.27	0.16	0.59
YGR092W	DBF2	8889	7349	1.21	0.27	0.16	0.59
YGL114W		2213	1830	1.21	0.27	0.16	0.59
YLR394W		1762	1457	1.21	0.27	0.16	0.59
YMR278W		1111	919	1.21	0.27	0.16	0.59
YNL131W	TOM22	3070	2539	1.21	0.27	0.16	0.59
YOL136C	PFK27	13288	10989	1.21	0.27	0.16	0.59
YHR071W	PCL5	2135	1766	1.21	0.27	0.16	0.59
YOR254C	SEC63	5006	4141	1.21	0.27	0.16	0.59
YDR421W		5587	4622	1.21	0.27	0.16	0.59
YLL034C		6727	5566	1.21	0.27	0.16	0.59
YDR377W	ATP17	12934	10702	1.21	0.27	0.16	0.58
YAR042W	SWH1	3457	2861	1.21	0.27	0.16	0.58
YGL235W		1676	1387	1.21	0.27	0.16	0.58
YLR453C	RIF2	1185	981	1.21	0.27	0.16	0.58
YJR027W		2712	2245	1.21	0.27	0.16	0.58
YHR143W-A	RPC10	1475	1221	1.21	0.27	0.16	0.58
YDL236W	PHO13	8185	6775	1.21	0.27	0.16	0.58
YDR539W		1984	1642	1.21	0.27	0.16	0.58
YGL222C		5617	4650	1.21	0.27	0.16	0.58
YNL233W	BNI4	3591	2973	1.21	0.27	0.16	0.58
YLR080W		965	799	1.21	0.27	0.16	0.58
YOR353C		8123	6726	1.21	0.27	0.16	0.58
YOL150C		9163	7588	1.21	0.27	0.16	0.58
YMR051C		8910	7379	1.21	0.27	0.16	0.58

YHR165C	PRP8	1044	865	1.21	0.27	0.16	0.58
YGL107C		9233	7647	1.21	0.27	0.16	0.58
YMR146C	TIF34	18452	15286	1.21	0.27	0.16	0.58
YGR241C	YAP1802	361	299	1.21	0.27	0.16	0.58
YFL025C	BST1	6841	5668	1.21	0.27	0.16	0.58
YKR105C		1450	1202	1.21	0.27	0.16	0.58
YIL014W	MNT3	2406	1994	1.21	0.27	0.16	0.58
YOL050C		3043	2522	1.21	0.27	0.16	0.58
YBR081C	SPT7	3698	3065	1.21	0.27	0.16	0.58
YCRX16C		10570	8763	1.21	0.27	0.16	0.57
YKL174C		1630	1351	1.21	0.27	0.16	0.57
YJR028W		6760	5605	1.21	0.27	0.16	0.57
YER186C		9878	8191	1.21	0.27	0.16	0.57
YDR301W	CFT1	1348	1118	1.21	0.27	0.16	0.57
YJL070C		3675	3048	1.21	0.27	0.16	0.57
YCR053W	THR4	15344	12729	1.21	0.27	0.16	0.57
YNL034W		1560	1294	1.21	0.27	0.16	0.57
YPL230W	USV1	988	820	1.21	0.27	0.16	0.57
YCR032W	BPH1	591	490	1.21	0.27	0.16	0.57
YCL013W		3244	2692	1.21	0.27	0.16	0.57
YMR067C		3038	2521	1.20	0.27	0.16	0.57
YDR322W	MRPL35	6001	4981	1.20	0.27	0.16	0.57
YCR045C		8629	7163	1.20	0.27	0.16	0.57
YLR264W	RPS28B	26261	21800	1.20	0.27	0.16	0.57
YJR040W	GEF1	3532	2933	1.20	0.27	0.16	0.57
YKR085C	MRPL20	6071	5041	1.20	0.27	0.16	0.57
YDR424C	DYN2	1851	1537	1.20	0.27	0.16	0.57
YAL017W	FUN31	4236	3518	1.20	0.27	0.16	0.57
YOL079W		626	520	1.20	0.27	0.16	0.57
YBL031W	SHE1	2272	1887	1.20	0.27	0.16	0.57
YNL304W	YPT11	2018	1676	1.20	0.27	0.16	0.56
YLR435W		6915	5744	1.20	0.27	0.16	0.56
YDR241W		1128	937	1.20	0.27	0.16	0.56
YJL030W	MAD2	10290	8549	1.20	0.27	0.15	0.56
YML072C		2376	1974	1.20	0.27	0.15	0.56
YDR189W	SLY1	3966	3295	1.20	0.27	0.15	0.56

YDR337W	MRPS28	6904	5737	1.20	0.27	0.15	0.56
YEL063C	CAN1	4280	3557	1.20	0.27	0.15	0.56
YOR022C		3423	2845	1.20	0.27	0.15	0.56
YGL228W	SHE10	18548	15416	1.20	0.27	0.15	0.56
YBR075W		5635	4685	1.20	0.27	0.15	0.56
YPR006C	ICL2	4303	3578	1.20	0.27	0.15	0.56
YOL104C	NDJ1	4571	3801	1.20	0.27	0.15	0.56
YFR009W	GCN20	11229	9337	1.20	0.27	0.15	0.56
YOR153W	PDR5	24118	20054	1.20	0.27	0.15	0.56
YMR218C	TRS130	510	424	1.20	0.27	0.15	0.56
YIL006W		1029	856	1.20	0.27	0.15	0.56
YLR310C	CDC25	489	407	1.20	0.27	0.15	0.56
YPL277C		1687	1403	1.20	0.27	0.15	0.56
YBR127C	VMA2	20659	17182	1.20	0.27	0.15	0.56
YOR334W	MRS2	3348	2785	1.20	0.27	0.15	0.56
YEL042W	GDA1	3932	3271	1.20	0.27	0.15	0.56
YDR316W		6420	5341	1.20	0.27	0.15	0.56
YOL018C	TLG2	8529	7096	1.20	0.27	0.15	0.56
YMR256C	COX7	5569	4634	1.20	0.27	0.15	0.56
YHL004W	MRP4	9628	8012	1.20	0.27	0.15	0.56
YDR062W	LCB2	16001	13317	1.20	0.26	0.15	0.55
YOR065W	CYT1	4663	3881	1.20	0.26	0.15	0.55
YHR030C	SLT2	753	627	1.20	0.26	0.15	0.55
YJL182C		793	660	1.20	0.26	0.15	0.55
YIL174W		812	676	1.20	0.26	0.15	0.55
YNL204C	SPS18	1307	1088	1.20	0.26	0.15	0.55
YMR164C	MSS11	2789	2322	1.20	0.26	0.15	0.55
YJR035W	RAD26	425	354	1.20	0.26	0.15	0.55
YDR351W	SBE2	5979	4979	1.20	0.26	0.15	0.55
YGR257C		5798	4828	1.20	0.26	0.15	0.55
YGR169C		2280	1899	1.20	0.26	0.15	0.55
YBR105C	VID24	5198	4329	1.20	0.26	0.15	0.55
YJR142W		4800	3998	1.20	0.26	0.15	0.55
YAL046C		6033	5026	1.20	0.26	0.15	0.55
YAR020C	PAU7	521	434	1.20	0.26	0.15	0.55
YDR371W		9107	7592	1.20	0.26	0.15	0.55

YLL051C	FRE6	10241	8537	1.20	0.26	0.15	0.55
YCR015C		1038	865	1.20	0.26	0.15	0.55
YKL075C		3014	2513	1.20	0.26	0.15	0.55
YOR243C		3970	3310	1.20	0.26	0.15	0.54
YNL212W		478	399	1.20	0.26	0.15	0.54
YKR025W	RPC37	14839	12375	1.20	0.26	0.15	0.54
YGL139W		9125	7610	1.20	0.26	0.15	0.54
YDR259C	YAP6	644	537	1.20	0.26	0.15	0.54
YBL063W	KIP1	1714	1430	1.20	0.26	0.15	0.54
YNL176C		9958	8310	1.20	0.26	0.15	0.54
YLR448W	RPL6B	13230	11042	1.20	0.26	0.15	0.54
YNL236W	SIN4	2551	2129	1.20	0.26	0.15	0.54
YGR039W		2688	2244	1.20	0.26	0.15	0.54
YIL039W		9240	7716	1.20	0.26	0.15	0.54
YER180C	ISC10	7952	6641	1.20	0.26	0.15	0.54
YGR247W		2306	1926	1.20	0.26	0.15	0.54
YLR241W		4692	3919	1.20	0.26	0.15	0.54
YML093W		3352	2800	1.20	0.26	0.15	0.54
YFR040W	SAP155	5720	4778	1.20	0.26	0.15	0.54
YGR190C		6725	5618	1.20	0.26	0.15	0.53
YPR039W		2212	1848	1.20	0.26	0.15	0.53
YBR007C		787	658	1.20	0.26	0.15	0.53
YNL229C	URE2	15349	12828	1.20	0.26	0.15	0.53
YDL039C		1342	1122	1.20	0.26	0.15	0.53
YLR092W	SUL2	3182	2660	1.20	0.26	0.15	0.53
YGR194C	XKS1	1830	1530	1.20	0.26	0.15	0.53
YER047C	SAP1	4778	3994	1.20	0.26	0.15	0.53
YPL179W	PPQ1	4085	3415	1.20	0.26	0.15	0.53
YKR087C		8309	6947	1.20	0.26	0.15	0.53
YLR156W		2301	1924	1.20	0.26	0.15	0.53
YGL239C		1498	1253	1.20	0.26	0.15	0.53
YNL106C	INP52	4714	3942	1.20	0.26	0.15	0.53
YBL004W		705	590	1.20	0.26	0.15	0.53
YMR158W		8406	7031	1.20	0.26	0.15	0.53
YDR228C	PCF11	2258	1889	1.20	0.26	0.15	0.53
YGL258W		1143	956	1.20	0.26	0.15	0.53

YLR004C		1409	1179	1.20	0.26	0.15	0.53
YLL029W		6476	5418	1.20	0.26	0.14	0.53
YIL122W		4685	3920	1.20	0.26	0.14	0.53
YAL027W		4066	3402	1.20	0.26	0.14	0.53
YBR150C	TBS1	2137	1788	1.20	0.26	0.14	0.53
YJL018W		3501	2930	1.19	0.26	0.14	0.53
YPR064W		1053	881	1.19	0.26	0.14	0.53
YCL064C	CHA1	8418	7046	1.19	0.26	0.14	0.52
YHL017W		9951	8329	1.19	0.26	0.14	0.52
YDR442W		786	658	1.19	0.26	0.14	0.52
YIL052C	RPL34B	37398	31306	1.19	0.26	0.14	0.52
YLR147C	SMD3	5813	4866	1.19	0.26	0.14	0.52
YDR462W	MRPL28	5799	4855	1.19	0.26	0.14	0.52
YDR076W	RAD55	2809	2352	1.19	0.26	0.14	0.52
YPL255W	BBP1	11485	9616	1.19	0.26	0.14	0.52
YNL001W	DOM34	8994	7531	1.19	0.26	0.14	0.52
YOL091W	SPO21	831	696	1.19	0.26	0.14	0.52
YER176W	ECM32	4834	4048	1.19	0.26	0.14	0.52
YBR019C	GAL10	793	664	1.19	0.26	0.14	0.52
YEL004W	YEA4	1989	1666	1.19	0.26	0.14	0.52
YCRX18C		2098	1758	1.19	0.26	0.14	0.52
YPR013C		1571	1316	1.19	0.26	0.14	0.52
YMR243C	ZRC1	5379	4507	1.19	0.26	0.14	0.52
YOL140W	ARG8	5989	5018	1.19	0.26	0.14	0.52
YGL203C	KEX1	5311	4450	1.19	0.26	0.14	0.52
YBL111C		1033	866	1.19	0.26	0.14	0.52
YMR191W		1211	1015	1.19	0.25	0.14	0.52
YOR101W	RAS1	6883	5769	1.19	0.25	0.14	0.52
YOR350C	MNE1	2738	2295	1.19	0.25	0.14	0.52
YPR166C	MRP2	4037	3384	1.19	0.25	0.14	0.52
YOL036W		7418	6218	1.19	0.25	0.14	0.52
YOL145C	CTR9	4904	4111	1.19	0.25	0.14	0.52
YHR090C	NBN1	1345	1128	1.19	0.25	0.14	0.51
YOR054C		1753	1470	1.19	0.25	0.14	0.51
YBL033C	RIB1	3960	3322	1.19	0.25	0.14	0.51
YDR477W	SNF1	4675	3922	1.19	0.25	0.14	0.51

YOL053W		6001	5035	1.19	0.25	0.14	0.51
YPR055W	SEC8	3096	2599	1.19	0.25	0.14	0.51
YLR171W		17974	15087	1.19	0.25	0.14	0.51
YNL252C	MRPL17	5556	4664	1.19	0.25	0.14	0.51
YIL027C		5445	4572	1.19	0.25	0.14	0.51
YHR022C		3217	2701	1.19	0.25	0.14	0.51
YIL105C		8865	7446	1.19	0.25	0.14	0.51
YFR056C		10807	9077	1.19	0.25	0.14	0.51
YPR040W		5859	4922	1.19	0.25	0.14	0.51
YMR089C	YTA12	7636	6415	1.19	0.25	0.14	0.51
YJL214W	HXT8	1437	1207	1.19	0.25	0.14	0.51
YHR066W	SSF1	2606	2189	1.19	0.25	0.14	0.51
YBR234C	ARC40	2071	1740	1.19	0.25	0.14	0.51
YIL151C		1176	988	1.19	0.25	0.14	0.50
YIL094C	LYS12	16319	13715	1.19	0.25	0.14	0.50
YDR484W	SAC2	1174	987	1.19	0.25	0.14	0.50
YDR122W	KIN1	3226	2712	1.19	0.25	0.14	0.50
YGR036C	CAX4	4197	3528	1.19	0.25	0.14	0.50
YDL244W	THI13	1044	878	1.19	0.25	0.14	0.50
YBR271W		19738	16593	1.19	0.25	0.14	0.50
YNL285W		2133	1793	1.19	0.25	0.14	0.50
YML033W		3090	2598	1.19	0.25	0.14	0.50
YGL084C		3787	3185	1.19	0.25	0.14	0.50
YOR367W	SCP1	5929	4987	1.19	0.25	0.14	0.50
YAL021C	CCR4	3646	3067	1.19	0.25	0.14	0.50
YJR083C		5917	4978	1.19	0.25	0.14	0.50
YOR080W		2448	2060	1.19	0.25	0.14	0.50
YJL095W	BCK1	1319	1110	1.19	0.25	0.14	0.50
YCL030C	HIS4	14420	12135	1.19	0.25	0.14	0.50
YAR061W		1107	932	1.19	0.25	0.14	0.50
YGR141W		2232	1879	1.19	0.25	0.14	0.50
YLR454W		1252	1054	1.19	0.25	0.14	0.50
YKR031C	SPO14	707	595	1.19	0.25	0.14	0.49
YBR189W	RPS9B	44678	37623	1.19	0.25	0.14	0.49
YHR121W		2051	1727	1.19	0.25	0.14	0.49
YFL004W		13403	11289	1.19	0.25	0.14	0.49

YDR227W	SIR4	632	532	1.19	0.25	0.14	0.49
YJL105W		1497	1261	1.19	0.25	0.14	0.49
YOR192C		1676	1412	1.19	0.25	0.13	0.49
YML048W-A		432	364	1.19	0.25	0.13	0.49
YGR280C		5009	4221	1.19	0.25	0.13	0.49
YGL112C	TAF60	8138	6858	1.19	0.25	0.13	0.49
YBR031W	RPL4A	17133	14439	1.19	0.25	0.13	0.49
YNR038W	DBP6	15813	13327	1.19	0.25	0.13	0.49
YOR216C	RUD3	5157	4346	1.19	0.25	0.13	0.49
YLR363C	NMD4	6055	5103	1.19	0.25	0.13	0.49
YDL218W		1275	1075	1.19	0.25	0.13	0.49
YGL049C	TIF4632	10904	9190	1.19	0.25	0.13	0.49
YPL022W	RAD1	1650	1391	1.19	0.25	0.13	0.49
YOR382W		2859	2410	1.19	0.25	0.13	0.49
YGL172W	NUP49	11610	9790	1.19	0.25	0.13	0.49
YCRX06W		2390	2015	1.19	0.25	0.13	0.49
YLR263W	RED1	1297	1094	1.19	0.25	0.13	0.49
YOL098C		10545	8893	1.19	0.25	0.13	0.49
YDL013W	HEX3	6999	5903	1.19	0.25	0.13	0.48
YDL016C		3228	2723	1.19	0.25	0.13	0.48
YGR068C		1435	1211	1.19	0.25	0.13	0.48
YBR216C		2532	2136	1.19	0.25	0.13	0.48
YGL160W		11753	9916	1.19	0.25	0.13	0.48
YPL161C	BEM4	1166	984	1.19	0.24	0.13	0.48
YPR118W		18977	16015	1.18	0.24	0.13	0.48
YNR070W		1316	1111	1.18	0.24	0.13	0.48
YOR260W	GCD1	11911	10053	1.18	0.24	0.13	0.48
YJR101W		569	480	1.18	0.24	0.13	0.48
YMR217W	GUA1	13398	11310	1.18	0.24	0.13	0.48
YLR258W	GSY2	3754	3169	1.18	0.24	0.13	0.48
YNR047W		3576	3019	1.18	0.24	0.13	0.48
YOL087C		1073	906	1.18	0.24	0.13	0.48
YHR078W		1497	1264	1.18	0.24	0.13	0.48
YHR020W		15453	13049	1.18	0.24	0.13	0.48
YBR146W	MRPS9	10518	8883	1.18	0.24	0.13	0.48
YMR052C-A		3263	2756	1.18	0.24	0.13	0.48

YIL097W		4562	3853	1.18	0.24	0.13	0.48
YBR232C		1971	1665	1.18	0.24	0.13	0.48
YEL009C	GCN4	23672	19995	1.18	0.24	0.13	0.48
YOL094C	RFC4	9453	7985	1.18	0.24	0.13	0.48
YGL240W	DOC1	1299	1097	1.18	0.24	0.13	0.48
YBR017C	KAP104	751	634	1.18	0.24	0.13	0.48
YAL025C	MAK16	8569	7240	1.18	0.24	0.13	0.48
YNL206C		12394	10473	1.18	0.24	0.13	0.47
YDR407C	TRS120	3162	2672	1.18	0.24	0.13	0.47
YJR095W	SFC1	1214	1026	1.18	0.24	0.13	0.47
YPR109W		2126	1797	1.18	0.24	0.13	0.47
YPL080C		2280	1928	1.18	0.24	0.13	0.47
YKL136W		1184	1001	1.18	0.24	0.13	0.47
YLR017W	MEU1	6302	5330	1.18	0.24	0.13	0.47
YDL146W		1698	1436	1.18	0.24	0.13	0.47
YER174C	GRX4	9871	8349	1.18	0.24	0.13	0.47
YGL108C		5661	4788	1.18	0.24	0.13	0.47
YHR065C	RRP3	2632	2226	1.18	0.24	0.13	0.47
YMR135W-A		3969	3357	1.18	0.24	0.13	0.47
YAR050W	FLO1	1984	1678	1.18	0.24	0.13	0.47
YHR173C		1118	946	1.18	0.24	0.13	0.47
YDL032W		2235	1891	1.18	0.24	0.13	0.47
YDR517W	GRH1	3457	2925	1.18	0.24	0.13	0.47
YDL029W	ARP2	8476	7171	1.18	0.24	0.13	0.47
YLR232W		2293	1940	1.18	0.24	0.13	0.47
YNL214W	PEX17	3650	3089	1.18	0.24	0.13	0.47
YEL019C	MMS21	688	582	1.18	0.24	0.13	0.47
YLR422W		2253	1907	1.18	0.24	0.13	0.47
YNL180C	RHO5	14243	12057	1.18	0.24	0.13	0.47
YMR245W		2174	1840	1.18	0.24	0.13	0.47
YDL167C	NRP1	21316	18047	1.18	0.24	0.13	0.46
YPL145C	KES1	11636	9854	1.18	0.24	0.13	0.46
YCR080W		1170	991	1.18	0.24	0.13	0.46
YGR098C	ESP1	6619	5606	1.18	0.24	0.13	0.46
YFL019C		779	660	1.18	0.24	0.13	0.46
YLR275W	SMD2	4720	3999	1.18	0.24	0.13	0.46

YLR009W		12180	10319	1.18	0.24	0.13	0.46
YDL179W	PCL9	15478	13115	1.18	0.24	0.13	0.46
YGR035C		1087	921	1.18	0.24	0.13	0.46
YMR158W-A		983	833	1.18	0.24	0.13	0.46
YOL029C		5113	4333	1.18	0.24	0.13	0.46
YOR329C	SCD5	4863	4121	1.18	0.24	0.13	0.46
YBR061C		17906	15175	1.18	0.24	0.13	0.46
YGR126W		1877	1591	1.18	0.24	0.13	0.46
YKL068W	NUP100	9791	8300	1.18	0.24	0.13	0.46
YDR545W	YRF1-1	3295	2793	1.18	0.24	0.13	0.46
YGR077C	PEX8	10185	8634	1.18	0.24	0.13	0.46
YJR029W		7997	6780	1.18	0.24	0.13	0.46
YML100W-A		1341	1137	1.18	0.24	0.13	0.46
YDR409W		2457	2083	1.18	0.24	0.13	0.46
YNL313C		13204	11197	1.18	0.24	0.13	0.46
YBR206W		3981	3376	1.18	0.24	0.13	0.46
YBL069W	AST1	2674	2268	1.18	0.24	0.13	0.46
YLR023C		3954	3354	1.18	0.24	0.12	0.45
YGL209W	MIG2	607	515	1.18	0.24	0.12	0.45
YGL254W	FZF1	7061	5991	1.18	0.24	0.12	0.45
YBR215W	HPC2	12428	10545	1.18	0.24	0.12	0.45
YPL191C		2342	1987	1.18	0.24	0.12	0.45
YLR254C		6168	5234	1.18	0.24	0.12	0.45
YKL215C		2753	2337	1.18	0.24	0.12	0.45
YHR205W	SCH9	775	658	1.18	0.24	0.12	0.45
YJL037W		519	441	1.18	0.24	0.12	0.45
YIL066C	RNR3	5066	4301	1.18	0.24	0.12	0.45
YNL145W	MFA2	910	773	1.18	0.24	0.12	0.45
YPR182W	SMX3	5334	4529	1.18	0.24	0.12	0.45
YHR110W	ERP5	5418	4600	1.18	0.24	0.12	0.45
YMR192W		1963	1667	1.18	0.24	0.12	0.45
YDR386W	MUS81	2489	2114	1.18	0.24	0.12	0.45
YER089C	PTC2	1512	1284	1.18	0.24	0.12	0.45
YGR034W	RPL26B	26630	22617	1.18	0.24	0.12	0.45
YJR030C		3692	3136	1.18	0.24	0.12	0.45
YMR151W	YIM2	1382	1174	1.18	0.24	0.12	0.45

YLR260W	LCB5	6604	5610	1.18	0.24	0.12	0.45
YOR207C	RET1	14666	12459	1.18	0.24	0.12	0.45
YOR308C	SNU66	1388	1179	1.18	0.23	0.12	0.45
YAL067C	SEO1	634	539	1.18	0.23	0.12	0.45
YGL136C		1564	1329	1.18	0.23	0.12	0.45
YNL234W		3195	2715	1.18	0.23	0.12	0.44
YML052W	SUR7	43115	36643	1.18	0.23	0.12	0.44
YOR262W		11001	9350	1.18	0.23	0.12	0.44
YOL041C		14653	12455	1.18	0.23	0.12	0.44
YFL054C		1357	1154	1.18	0.23	0.12	0.44
YFR019W	FAB1	2485	2113	1.18	0.23	0.12	0.44
YOR032C	HMS1	1043	887	1.18	0.23	0.12	0.44
YPR186C	PZF1	2495	2122	1.18	0.23	0.12	0.44
YEL061C	CIN8	2915	2480	1.18	0.23	0.12	0.44
YDL154W	MSH5	1332	1133	1.18	0.23	0.12	0.44
YDR160W	SSY1	1675	1425	1.18	0.23	0.12	0.44
YDL245C	HXT15	1322	1125	1.18	0.23	0.12	0.44
YIL137C		4715	4013	1.17	0.23	0.12	0.44
YMR118C		3360	2860	1.17	0.23	0.12	0.44
YPL259C	APM1	9878	8408	1.17	0.23	0.12	0.44
YHR170W	NMD3	5003	4259	1.17	0.23	0.12	0.44
YLR395C	COX8	13121	11170	1.17	0.23	0.12	0.44
YPL140C	MKK2	13470	11467	1.17	0.23	0.12	0.44
YIL068C	SEC6	4647	3956	1.17	0.23	0.12	0.44
YOR135C		18284	15567	1.17	0.23	0.12	0.44
YJR064W	CCT5	14493	12341	1.17	0.23	0.12	0.43
YGL248W	PDE1	9516	8104	1.17	0.23	0.12	0.43
YCR093W	CDC39	1390	1184	1.17	0.23	0.12	0.43
YOR389W		3054	2601	1.17	0.23	0.12	0.43
YML097C	VPS9	6299	5365	1.17	0.23	0.12	0.43
YBR188C	NTC20	2312	1969	1.17	0.23	0.12	0.43
YJL206C-A		3150	2683	1.17	0.23	0.12	0.43
YNR061C		5591	4763	1.17	0.23	0.12	0.43
YGR096W		5811	4951	1.17	0.23	0.12	0.43
YNR021W		16663	14198	1.17	0.23	0.12	0.43
YNL127W		1733	1477	1.17	0.23	0.12	0.43

YER158C		2745	2339	1.17	0.23	0.12	0.43
YML063W	RPS1B	25467	21700	1.17	0.23	0.12	0.43
YJR055W	HIT1	1044	890	1.17	0.23	0.12	0.43
YMR125W	STO1	6156	5246	1.17	0.23	0.12	0.43
YGL204C		8298	7072	1.17	0.23	0.12	0.43
YHR060W	VMA22	2848	2427	1.17	0.23	0.12	0.43
YBL089W		1522	1297	1.17	0.23	0.12	0.43
YOR394W		1033	881	1.17	0.23	0.12	0.43
YPR049C		3861	3291	1.17	0.23	0.12	0.43
YJR106W	ECM27	3096	2640	1.17	0.23	0.12	0.43
YMR068W		1237	1055	1.17	0.23	0.12	0.43
YOL049W	GSH2	3777	3221	1.17	0.23	0.12	0.43
YNL244C	SUI1	17996	15347	1.17	0.23	0.12	0.43
YHR001W		5414	4617	1.17	0.23	0.12	0.43
YCR064C		1887	1609	1.17	0.23	0.12	0.43
YBR022W		3780	3224	1.17	0.23	0.12	0.43
YDR018C		1202	1025	1.17	0.23	0.12	0.43
YNL025C	SSN8	2033	1734	1.17	0.23	0.12	0.43
YER129W	PAK1	2808	2396	1.17	0.23	0.12	0.42
YLR192C	HCR1	15785	13472	1.17	0.23	0.12	0.42
YER139C		4154	3546	1.17	0.23	0.12	0.42
YGL255W	ZRT1	2872	2452	1.17	0.23	0.12	0.42
YJL221C	FSP2	816	697	1.17	0.23	0.12	0.42
YFL063W		1322	1129	1.17	0.23	0.12	0.42
YMR309C	NIP1	10385	8870	1.17	0.23	0.12	0.42
YCR028C	FEN2	4649	3971	1.17	0.23	0.12	0.42
YNL254C		1647	1407	1.17	0.23	0.12	0.42
YML094W	GIM5	6939	5927	1.17	0.23	0.12	0.42
YBR095C		6103	5213	1.17	0.23	0.11	0.42
YHR025W	THR1	3340	2853	1.17	0.23	0.11	0.42
YLR437C		4301	3674	1.17	0.23	0.11	0.42
YLR328W		6227	5320	1.17	0.23	0.11	0.42
YDL119C		11650	9954	1.17	0.23	0.11	0.42
YOL112W	MSB4	1777	1519	1.17	0.23	0.11	0.41
YOR292C		9855	8424	1.17	0.23	0.11	0.41
YNR025C		2634	2252	1.17	0.23	0.11	0.41

YHR011W		2236	1912	1.17	0.23	0.11	0.41
YLR044C	PDC1	14109	12064	1.17	0.23	0.11	0.41
YDL045C	FAD1	4001	3422	1.17	0.23	0.11	0.41
YOR009W		719	615	1.17	0.23	0.11	0.41
YBR262C		6719	5748	1.17	0.23	0.11	0.41
YHR149C		2586	2213	1.17	0.22	0.11	0.41
YCR086W		12312	10534	1.17	0.22	0.11	0.41
YBR220C		3400	2909	1.17	0.22	0.11	0.41
YMR200W	ROT1	7636	6534	1.17	0.22	0.11	0.41
YNL205C		725	620	1.17	0.22	0.11	0.41
YJR152W	DAL5	3759	3218	1.17	0.22	0.11	0.41
YKR019C	IRS4	1293	1107	1.17	0.22	0.11	0.41
YMR319C	FET4	5747	4920	1.17	0.22	0.11	0.41
YIR021W	MRS1	5128	4390	1.17	0.22	0.11	0.41
YIL035C	CKA1	3601	3083	1.17	0.22	0.11	0.41
YOR078W		10961	9385	1.17	0.22	0.11	0.41
YNL058C		12521	10721	1.17	0.22	0.11	0.41
YML065W	ORC1	4216	3610	1.17	0.22	0.11	0.41
YGL118C		585	501	1.17	0.22	0.11	0.41
YJR068W	RFC2	4783	4096	1.17	0.22	0.11	0.41
YER096W	SHC1	1947	1667	1.17	0.22	0.11	0.40
YDR066C		2306	1975	1.17	0.22	0.11	0.40
YLL038C	ENT4	2633	2255	1.17	0.22	0.11	0.40
YOR241W		2928	2508	1.17	0.22	0.11	0.40
YIL130W		4413	3780	1.17	0.22	0.11	0.40
YPL200W		576	493	1.17	0.22	0.11	0.40
YIR003W		10085	8640	1.17	0.22	0.11	0.40
YPL101W		4477	3836	1.17	0.22	0.11	0.40
YGR193C	PDX1	5989	5132	1.17	0.22	0.11	0.40
YKL017C	HCS1	3684	3157	1.17	0.22	0.11	0.40
YOR145C		30519	26154	1.17	0.22	0.11	0.40
YOR269W	PAC1	3379	2896	1.17	0.22	0.11	0.40
YLL057C		2446	2096	1.17	0.22	0.11	0.40
YGL178W	MPT5	843	723	1.17	0.22	0.11	0.40
YGL064C		827	709	1.17	0.22	0.11	0.40
YMR113W		3379	2897	1.17	0.22	0.11	0.40

YHR030C	SLT2	9422	8077	1.17	0.22	0.11	0.40
YPL074W	YTA6	6986	5989	1.17	0.22	0.11	0.40
YLR333C	RPS25B	25498	21861	1.17	0.22	0.11	0.40
YJR153W	PGU1	930	797	1.17	0.22	0.11	0.40
YBL070C		4742	4066	1.17	0.22	0.11	0.40
YKL214C		5308	4552	1.17	0.22	0.11	0.40
YLR410W	VIP1	2853	2447	1.17	0.22	0.11	0.40
YCL045C		349	299	1.17	0.22	0.11	0.40
YDR011W	SNQ2	649	557	1.17	0.22	0.11	0.40
YIR033W	MGA2	4433	3803	1.17	0.22	0.11	0.40
YMR059W	SEN15	3062	2627	1.17	0.22	0.11	0.40
YOR169C		4178	3584	1.17	0.22	0.11	0.40
YBL077W		4195	3600	1.17	0.22	0.11	0.39
YOL063C		1217	1044	1.17	0.22	0.11	0.39
YEL059C-A	SOM1	2928	2513	1.17	0.22	0.11	0.39
YCR084C	TUP1	8499	7294	1.17	0.22	0.11	0.39
YGL202W	ARO8	18362	15760	1.17	0.22	0.11	0.39
YOR096W	RPS7A	19802	16998	1.16	0.22	0.11	0.39
YGL012W	ERG4	10573	9076	1.16	0.22	0.11	0.39
YCL062W		1937	1663	1.16	0.22	0.11	0.39
YER037W		3777	3243	1.16	0.22	0.11	0.39
YDL108W	KIN28	4004	3438	1.16	0.22	0.11	0.39
YMR234W	RNH1	1345	1155	1.16	0.22	0.11	0.39
YMR130W		1226	1053	1.16	0.22	0.11	0.39
YNL004W	HRB1	28335	24338	1.16	0.22	0.11	0.39
YDR159W	SAC3	5290	4545	1.16	0.22	0.11	0.39
YOL027C		11259	9674	1.16	0.22	0.11	0.39
YMR098C		2584	2220	1.16	0.22	0.11	0.39
YNL130C	CPT1	14107	12123	1.16	0.22	0.11	0.39
YOR177C		1405	1208	1.16	0.22	0.11	0.39
YKL053W		2465	2119	1.16	0.22	0.11	0.38
YLL022C	HIF1	5653	4860	1.16	0.22	0.11	0.38
YBL045C	COR1	9296	7994	1.16	0.22	0.11	0.38
YBR143C	SUP45	27496	23645	1.16	0.22	0.11	0.38
YLR279W		934	803	1.16	0.22	0.11	0.38
YAL010C	MDM10	3158	2716	1.16	0.22	0.11	0.38

YGR269W		723	622	1.16	0.22	0.11	0.38
YDR205W		2130	1832	1.16	0.22	0.10	0.38
YGR116W	SPT6	1306	1123	1.16	0.22	0.10	0.38
YJL020C		7558	6502	1.16	0.22	0.10	0.38
YBR203W		4007	3447	1.16	0.22	0.10	0.38
YGR191W	HIP1	8843	7608	1.16	0.22	0.10	0.38
YJR074W	MOG1	7508	6459	1.16	0.22	0.10	0.38
YPL235W	RVB2	14679	12629	1.16	0.22	0.10	0.38
YDL030W	PRP9	675	581	1.16	0.22	0.10	0.38
YPR106W	ISR1	4097	3525	1.16	0.22	0.10	0.38
YHR102W	NRK1	8442	7263	1.16	0.22	0.10	0.38
YOR143C	THI80	15496	13333	1.16	0.22	0.10	0.38
YLR367W	RPS22B	35132	30236	1.16	0.22	0.10	0.38
YDR469W		2276	1959	1.16	0.22	0.10	0.38
YBL042C	FUI1	12633	10875	1.16	0.22	0.10	0.38
YLR329W	REC102	897	772	1.16	0.22	0.10	0.38
YCR097WB		664	572	1.16	0.22	0.10	0.38
YNR054C		14215	12238	1.16	0.22	0.10	0.38
YGR013W	SNU71	6342	5460	1.16	0.22	0.10	0.38
YDL079C	MRK1	2757	2374	1.16	0.22	0.10	0.38
YPL157W		2425	2088	1.16	0.22	0.10	0.38
YER015W	FAA2	919	791	1.16	0.22	0.10	0.38
YKR023W		7321	6304	1.16	0.22	0.10	0.38
YMR106C	YKU80	1288	1109	1.16	0.22	0.10	0.38
YDL051W	YLA1	14086	12132	1.16	0.22	0.10	0.37
YKR102W	FLO10	509	438	1.16	0.22	0.10	0.37
YBR098W	MMS4	1308	1127	1.16	0.22	0.10	0.37
YHR070W		13504	11633	1.16	0.22	0.10	0.37
YIL098C	FMC1	6384	5500	1.16	0.22	0.10	0.37
YMR048W		6142	5292	1.16	0.21	0.10	0.37
YLR262C	YPT6	13157	11336	1.16	0.21	0.10	0.37
YGR177C	ATF2	4941	4257	1.16	0.21	0.10	0.37
YBR246W		8124	7001	1.16	0.21	0.10	0.37
YMR003W		2678	2308	1.16	0.21	0.10	0.37
YPR086W	SUA7	16339	14083	1.16	0.21	0.10	0.37
YJR067C	YAE1	2920	2517	1.16	0.21	0.10	0.37

YJL150W		1614	1392	1.16	0.21	0.10	0.37
YNL022C		12878	11103	1.16	0.21	0.10	0.37
YGL227W	TIN1	1468	1266	1.16	0.21	0.10	0.37
YKL203C	TOR2	2569	2215	1.16	0.21	0.10	0.37
YDL021W	GPM2	543	468	1.16	0.21	0.10	0.37
YKL170W	MRPL38	10239	8830	1.16	0.21	0.10	0.37
YMR162C		688	593	1.16	0.21	0.10	0.37
YCL073C		7409	6390	1.16	0.21	0.10	0.37
YGR127W		1829	1578	1.16	0.21	0.10	0.37
YGL088W		983	848	1.16	0.21	0.10	0.37
YLR235C		3382	2918	1.16	0.21	0.10	0.37
YPR016C	TIF6	20443	17638	1.16	0.21	0.10	0.37
YCR049C		1440	1242	1.16	0.21	0.10	0.37
YLR026C	SED5	6548	5650	1.16	0.21	0.10	0.37
YDR419W	RAD30	5748	4960	1.16	0.21	0.10	0.37
YNL179C		3783	3264	1.16	0.21	0.10	0.36
YPL227C	ALG5	17951	15490	1.16	0.21	0.10	0.36
YJR122W	CAF17	1293	1116	1.16	0.21	0.10	0.36
YMR229C	RRP5	2592	2237	1.16	0.21	0.10	0.36
YDL191W	RPL35A	41532	35846	1.16	0.21	0.10	0.36
YMR082C		494	426	1.16	0.21	0.10	0.36
YDR207C	UME6	1936	1671	1.16	0.21	0.10	0.36
YNL128W	TEP1	1661	1434	1.16	0.21	0.10	0.36
YPL018W	CTF19	3162	2730	1.16	0.21	0.10	0.36
YOR122C	PFY1	13131	11340	1.16	0.21	0.10	0.36
YHL020C	OPI1	7174	6197	1.16	0.21	0.10	0.36
YPL030W		3174	2742	1.16	0.21	0.10	0.36
YDR281C		5100	4406	1.16	0.21	0.10	0.36
YBR297W	MAL33	4154	3589	1.16	0.21	0.10	0.36
YLR455W		5777	4991	1.16	0.21	0.10	0.36
YIR040C		1412	1220	1.16	0.21	0.10	0.36
YML081W		3146	2718	1.16	0.21	0.10	0.36
YGL193C		2451	2118	1.16	0.21	0.10	0.36
YMR160W		2700	2333	1.16	0.21	0.10	0.36
YCR081W	SRB8	3204	2769	1.16	0.21	0.10	0.36
YKL188C	PXA2	753	651	1.16	0.21	0.10	0.36

YML062C	MFT1	5397	4665	1.16	0.21	0.10	0.36
YLR372W	SUR4	7721	6674	1.16	0.21	0.10	0.36
YOR090C	PTC5	3121	2698	1.16	0.21	0.10	0.36
YGR018C		4843	4188	1.16	0.21	0.10	0.35
YDR167W	TAF25	11960	10343	1.16	0.21	0.10	0.35
YGR114C		3444	2978	1.16	0.21	0.10	0.35
YGR129W	SYF2	2558	2212	1.16	0.21	0.10	0.35
YOR323C	PRO2	15098	13057	1.16	0.21	0.10	0.35
YDR142C	PEX7	5523	4777	1.16	0.21	0.10	0.35
YCL039W		2037	1762	1.16	0.21	0.10	0.35
YPL153C	RAD53	2119	1833	1.16	0.21	0.10	0.35
YEL027W	CUP5	12600	10900	1.16	0.21	0.10	0.35
YDR300C	PRO1	15973	13818	1.16	0.21	0.10	0.35
YOR383C		1893	1638	1.16	0.21	0.10	0.35
YGL028C	SCW11	9706	8397	1.16	0.21	0.10	0.35
YLR166C	SEC10	2647	2290	1.16	0.21	0.10	0.35
YOL117W		1121	970	1.16	0.21	0.10	0.35
YDR451C		7180	6213	1.16	0.21	0.10	0.35
YIL092W		2614	2262	1.16	0.21	0.10	0.35
YOR354C		5882	5091	1.16	0.21	0.10	0.35
YOR286W		11717	10141	1.16	0.21	0.10	0.35
YDL176W		2588	2240	1.16	0.21	0.10	0.35
YOL021C	DIS3	8082	6996	1.16	0.21	0.10	0.35
YKL173W	SNU114	1073	929	1.16	0.21	0.10	0.35
YPL245W		5716	4948	1.16	0.21	0.10	0.35
YLR443W	ECM7	4105	3554	1.16	0.21	0.10	0.35
YDR494W		13074	11319	1.16	0.21	0.10	0.35
YGL206C	CHC1	782	677	1.16	0.21	0.10	0.35
YNR016C	ACC1	2130	1844	1.16	0.21	0.10	0.35
YJL201W	ECM25	3023	2617	1.15	0.21	0.10	0.35
YBR152W	SPP381	3096	2681	1.15	0.21	0.10	0.35
YAL062W	GDH3	1100	953	1.15	0.21	0.10	0.35
YDR118W	APC4	5769	4996	1.15	0.21	0.10	0.35
YKR071C		18507	16028	1.15	0.21	0.10	0.35
YHR135C	YCK1	2757	2388	1.15	0.21	0.10	0.35
YEL002C	WBP1	16908	14643	1.15	0.21	0.10	0.35

YDR302W		10150	8791	1.15	0.21		0.10	0.35
YBR104W	YMC2	11300	9787	1.15	0.21		0.09	0.35
YOR303W	CPA1	12163	10535	1.15	0.21		0.09	0.34
YOL010W		8582	7435	1.15	0.21		0.09	0.34
YDR257C	RMS1	5414	4690	1.15	0.21		0.09	0.34
YNL202W	SPS19	2410	2088	1.15	0.21		0.09	0.34
YDR433W		11076	9596	1.15	0.21		0.09	0.34
YCR014C	POL4	2105	1824	1.15	0.21		0.09	0.34
YNL103W	MET4	4573	3962	1.15	0.21		0.09	0.34
YLR039C	RIC1	1660	1438	1.15	0.21		0.09	0.34
YOL033W	MSE1	4614	3998	1.15	0.21		0.09	0.34
YHR014W	SPO13	1103	956	1.15	0.21		0.09	0.34
YDR229W		3880	3362	1.15	0.21		0.09	0.34
YGR180C	RNR4	16206	14045	1.15	0.21		0.09	0.34
YPR072W	NOT5	5090	4411	1.15	0.21		0.09	0.34
YMR183C	SSO2	17441	15117	1.15	0.21		0.09	0.34
YHL047C		4048	3509	1.15	0.21		0.09	0.34
YPL173W	MRPL40	4253	3687	1.15	0.21		0.09	0.34
YHR018C	ARG4	30606	26535	1.15	0.21		0.09	0.34
YDR135C	YCF1	723	627	1.15	0.21		0.09	0.34
YLL044W		28987	25133	1.15	0.21		0.09	0.34
YPL171C	OYE3	745	646	1.15	0.21		0.09	0.34
YNL154C	YCK2	22342	19372	1.15	0.21		0.09	0.34
YDR287W		4049	3511	1.15	0.21		0.09	0.34
YNL053W	MSG5	5478	4750	1.15	0.21		0.09	0.34
YOL096C	COQ3	6823	5917	1.15	0.21		0.09	0.34
YMR193W	MRPL24	9177	7959	1.15	0.21		0.09	0.34
YBL020W	RFT1	15848	13746	1.15	0.21		0.09	0.34
YJL005W	CYR1	824	715	1.15	0.21		0.09	0.34
YIL045W	PIG2	2957	2565	1.15	0.21		0.09	0.34
YDR506C		853	740	1.15	0.21		0.09	0.34
YOR166C		3375	2928	1.15	0.21		0.09	0.34
YPL129W	ANC1	11665	10120	1.15	0.20		0.09	0.34
YDR381W	YRA1	17995	15612	1.15	0.20		0.09	0.34
YCRX14W		4187	3633	1.15	0.20		0.09	0.34
YDR416W	SYF1	4179	3626	1.15	0.20		0.09	0.34

YNR009W		3429	2975	1.15	0.20	0.09	0.34
YGL123W	RPS2	29541	25634	1.15	0.20	0.09	0.34
YLR018C		16262	14112	1.15	0.20	0.09	0.34
YOR049C		2715	2356	1.15	0.20	0.09	0.34
YEL029C		3803	3301	1.15	0.20	0.09	0.33
YOR014W	RTS1	1612	1399	1.15	0.20	0.09	0.33
YDR190C	RVB1	16092	13968	1.15	0.20	0.09	0.33
YPR192W	AQY1	1068	927	1.15	0.20	0.09	0.33
YNL169C	PSD1	3990	3465	1.15	0.20	0.09	0.33
YDL116W	NUP84	10317	8959	1.15	0.20	0.09	0.33
YDR263C	DIN7	1132	983	1.15	0.20	0.09	0.33
YLR073C		19978	17350	1.15	0.20	0.09	0.33
YJL090C	DPB11	5103	4432	1.15	0.20	0.09	0.33
YMR138W	CIN4	2627	2282	1.15	0.20	0.09	0.33
YPL177C	CUP9	12410	10780	1.15	0.20	0.09	0.33
YDL058W	USO1	7239	6288	1.15	0.20	0.09	0.33
YHR119W	SET1	7601	6603	1.15	0.20	0.09	0.33
YER166W		772	671	1.15	0.20	0.09	0.33
YFL011W-A		14004	12166	1.15	0.20	0.09	0.33
YJR073C	OPI3	4900	4257	1.15	0.20	0.09	0.33
YBL101C	ECM21	429	373	1.15	0.20	0.09	0.33
YCRX11W		815	708	1.15	0.20	0.09	0.33
YMR103C		1811	1574	1.15	0.20	0.09	0.33
YBR204C		1869	1624	1.15	0.20	0.09	0.33
YCLX06C		10643	9249	1.15	0.20	0.09	0.33
YGR091W	PRP31	1634	1420	1.15	0.20	0.09	0.33
YIL029C		1191	1035	1.15	0.20	0.09	0.33
YDR199W		878	763	1.15	0.20	0.09	0.33
YGR230W	BNS1	8560	7443	1.15	0.20	0.09	0.33
YLR167W	RPS31	39470	34318	1.15	0.20	0.09	0.32
YBL106C	SRO77	1109	964	1.15	0.20	0.09	0.32
YBR248C	HIS7	5072	4410	1.15	0.20	0.09	0.32
YGR027C	RPS25A	26355	22919	1.15	0.20	0.09	0.32
YMR287C	MSU1	6386	5553	1.15	0.20	0.09	0.32
YKL143W	LTV1	17293	15043	1.15	0.20	0.09	0.32
YPL075W	GCR1	5102	4438	1.15	0.20	0.09	0.32

YDL005C	MED2	9423	8197	1.15	0.20	0.09	0.32
YPR194C		1445	1257	1.15	0.20	0.09	0.32
YML004C	GLO1	11133	9685	1.15	0.20	0.09	0.32
YOL075C		1524	1326	1.15	0.20	0.09	0.32
YKL131W		3494	3041	1.15	0.20	0.09	0.32
YJL141C	YAK1	1851	1611	1.15	0.20	0.09	0.32
YJL137C	GLG2	1656	1441	1.15	0.20	0.09	0.32
YDR140W		15904	13844	1.15	0.20	0.09	0.32
YER153C	PET122	964	839	1.15	0.20	0.09	0.32
YER108C		2093	1822	1.15	0.20	0.09	0.32
YOR280C		5587	4864	1.15	0.20	0.09	0.32
YLR224W		9606	8363	1.15	0.20	0.09	0.32
YLR346C		1697	1477	1.15	0.20	0.09	0.32
YGR197C	SNG1	3356	2922	1.15	0.20	0.09	0.32
YPL195W	APL5	4391	3823	1.15	0.20	0.09	0.32
YDR479C		4432	3859	1.15	0.20	0.09	0.32
YAL059W	ECM1	20831	18139	1.15	0.20	0.09	0.32
YOR336W	KRE5	3583	3120	1.15	0.20	0.09	0.32
YPL047W		3739	3256	1.15	0.20	0.09	0.32
YOL006C	TOP1	5899	5137	1.15	0.20	0.09	0.32
YLR015W		1880	1637	1.15	0.20	0.09	0.32
YGL171W	ROK1	5501	4791	1.15	0.20	0.09	0.32
YLR067C	PET309	4167	3629	1.15	0.20	0.09	0.32
YMR252C		1619	1410	1.15	0.20	0.09	0.32
YER070W	RNR1	15941	13887	1.15	0.20	0.09	0.31
YJL052W	TDH1	35524	30951	1.15	0.20	0.09	0.31
YOL055C	THI20	5226	4554	1.15	0.20	0.09	0.31
YCR101C		2172	1893	1.15	0.20	0.09	0.31
YDL190C	UFD2	379	330	1.15	0.20	0.09	0.31
YKL180W	RPL17A	7371	6426	1.15	0.20	0.09	0.31
YGL042C		3222	2809	1.15	0.20	0.09	0.31
YER162C	RAD4	2694	2349	1.15	0.20	0.09	0.31
YPR011C		5040	4395	1.15	0.20	0.09	0.31
YOR309C		21664	18891	1.15	0.20	0.09	0.31
YPL082C	MOT1	11964	10434	1.15	0.20	0.09	0.31
YER046W		1322	1153	1.15	0.20	0.08	0.31

YDL248W	COS7	2365	2063	1.15	0.20	0.08	0.31
YLR294C		9192	8019	1.15	0.20	0.08	0.31
YNR040W		2581	2252	1.15	0.20	0.08	0.31
YGL192W	IME4	9755	8512	1.15	0.20	0.08	0.31
YOR180C	DCI1	4253	3711	1.15	0.20	0.08	0.31
YDR370C		5494	4795	1.15	0.20	0.08	0.31
YDR116C		16454	14361	1.15	0.20	0.08	0.30
YDR507C	GIN4	1493	1303	1.15	0.20	0.08	0.30
YPL264C		1705	1488	1.15	0.20	0.08	0.30
YLR061W	RPL22A	44113	38506	1.15	0.20	0.08	0.30
YOR339C	UBC11	2227	1944	1.15	0.20	0.08	0.30
YLR240W	VPS34	4986	4353	1.15	0.20	0.08	0.30
YKL162C		1921	1677	1.15	0.20	0.08	0.30
YDR434W		7882	6883	1.15	0.20	0.08	0.30
YBR139W		9072	7922	1.15	0.20	0.08	0.30
YNL187W		1594	1392	1.15	0.20	0.08	0.30
YEL069C	HXT13	2179	1903	1.15	0.20	0.08	0.30
YJL204C		3173	2771	1.15	0.20	0.08	0.30
YIL145C		14689	12830	1.14	0.20	0.08	0.30
YOR016C	ERP4	6480	5660	1.14	0.20	0.08	0.30
YER154W	OXA1	15073	13167	1.14	0.20	0.08	0.30
YLR233C	EST1	1448	1265	1.14	0.19	0.08	0.30
YDL187C		849	742	1.14	0.19	0.08	0.30
YDL034W		1557	1361	1.14	0.19	0.08	0.30
YOL103W	ITR2	8625	7539	1.14	0.19	0.08	0.30
YML023C		3923	3430	1.14	0.19	0.08	0.30
YNL332W	THI12	785	686	1.14	0.19	0.08	0.30
YBR282W	MRPL27	8398	7342	1.14	0.19	0.08	0.30
YDR423C	CAD1	5985	5234	1.14	0.19	0.08	0.29
YHL040C	ARN1	3214	2811	1.14	0.19	0.08	0.29
YBR047W		1774	1552	1.14	0.19	0.08	0.29
YGR264C	MES1	13098	11456	1.14	0.19	0.08	0.29
YGR293C		4707	4117	1.14	0.19	0.08	0.29
YKL007W	CAP1	8972	7849	1.14	0.19	0.08	0.29
YML053C		5182	4534	1.14	0.19	0.08	0.29
YPL176C		9148	8004	1.14	0.19	0.08	0.29

YFR015C	GSY1	3113	2724	1.14	0.19	0.08	0.29
YDL153C	SAS10	30845	26995	1.14	0.19	0.08	0.29
YDR094W		2178	1906	1.14	0.19	0.08	0.29
YOR256C		4007	3507	1.14	0.19	0.08	0.29
YGL054C	ERV14	14011	12264	1.14	0.19	0.08	0.29
YIL135C		7090	6207	1.14	0.19	0.08	0.29
YMR175W	SIP18	562	492	1.14	0.19	0.08	0.29
YPR126C		5526	4840	1.14	0.19	0.08	0.29
YHR075C		2499	2189	1.14	0.19	0.08	0.29
YDR413C		17117	14994	1.14	0.19	0.08	0.29
YHR161C	YAP1801	3639	3188	1.14	0.19	0.08	0.29
YJL169W		934	818	1.14	0.19	0.08	0.29
YDR075W	PPH3	4682	4102	1.14	0.19	0.08	0.29
YMR291W		4749	4161	1.14	0.19	0.08	0.28
YOR021C		15137	13263	1.14	0.19	0.08	0.28
YNL088W	TOP2	2468	2162	1.14	0.19	0.08	0.28
YLR114C		2723	2386	1.14	0.19	0.08	0.28
YCRX10W		2604	2282	1.14	0.19	0.08	0.28
YMR008C	PLB1	10942	9588	1.14	0.19	0.08	0.28
YLR439W	MRPL4	2763	2421	1.14	0.19	0.08	0.28
YPR111W	DBF20	1871	1640	1.14	0.19	0.08	0.28
YOR238W		9352	8196	1.14	0.19	0.08	0.28
YMR260C	TIF11	20554	18013	1.14	0.19	0.08	0.28
YJL194W	CDC6	1470	1288	1.14	0.19	0.08	0.28
YER061C	CEM1	5478	4801	1.14	0.19	0.08	0.28
YBL090W	MRP21	8802	7715	1.14	0.19	0.08	0.28
YDR058C	TGL2	1359	1191	1.14	0.19	0.08	0.28
YKL005C		3565	3125	1.14	0.19	0.08	0.28
YCL017C	NFS1	7267	6370	1.14	0.19	0.08	0.28
YGL209W	MIG2	6956	6097	1.14	0.19	0.08	0.28
YAR027W		895	785	1.14	0.19	0.08	0.28
YLR087C		936	821	1.14	0.19	0.08	0.28
YFL047W		8655	7588	1.14	0.19	0.08	0.28
YBL007C	SLA1	7146	6265	1.14	0.19	0.08	0.28
YDR078C	PUN1	5754	5045	1.14	0.19	0.08	0.28
YBR251W	MRPS5	12152	10655	1.14	0.19	0.08	0.28

YLR415C		1307	1146	1.14	0.19	0.08	0.28
YPR071W		4310	3781	1.14	0.19	0.08	0.28
YOR300W		1784	1565	1.14	0.19	0.08	0.28
YMR168C	CEP3	2346	2058	1.14	0.19	0.08	0.28
YCR106W		2448	2148	1.14	0.19	0.08	0.28
YAL069W		830	728	1.14	0.19	0.08	0.28
YKR090W		2707	2375	1.14	0.19	0.08	0.28
YDR366C		8987	7887	1.14	0.19	0.08	0.28
YBR268W	MRPL37	8042	7059	1.14	0.19	0.08	0.28
YBR270C		1292	1134	1.14	0.19	0.08	0.28
YML124C	TUB3	10004	8783	1.14	0.19	0.08	0.27
YML040W		31811	27933	1.14	0.19	0.08	0.27
YLR369W	SSQ1	3165	2779	1.14	0.19	0.08	0.27
YLR094C	GIS3	3635	3192	1.14	0.19	0.08	0.27
YJR156C	THI11	1787	1569	1.14	0.19	0.08	0.27
YER024W		2127	1868	1.14	0.19	0.08	0.27
YML031W	NDC1	7580	6657	1.14	0.19	0.07	0.27
YDR455C		3578	3142	1.14	0.19	0.07	0.27
YNL199C	GCR2	3692	3243	1.14	0.19	0.07	0.27
YLR273C	PIG1	2030	1783	1.14	0.19	0.07	0.27
YDR292C	SRP101	3185	2798	1.14	0.19	0.07	0.27
YGL008C	PMA1	42383	37232	1.14	0.19	0.07	0.27
YDL094C		3836	3370	1.14	0.19	0.07	0.27
YEL022W	GEA2	4827	4241	1.14	0.19	0.07	0.27
YER128W		12597	11068	1.14	0.19	0.07	0.27
YGR140W	CBF2	679	597	1.14	0.19	0.07	0.27
YFL043C		1114	979	1.14	0.19	0.07	0.27
YNL320W		863	758	1.14	0.19	0.07	0.27
YPL231W	FAS2	10245	9003	1.14	0.19	0.07	0.27
YDR015C		401	352	1.14	0.19	0.07	0.27
YGL102C		19265	16930	1.14	0.19	0.07	0.27
YMR257C	PET111	916	805	1.14	0.19	0.07	0.27
YDR201W	SPC19	1370	1204	1.14	0.19	0.07	0.27
YHR125W		3531	3104	1.14	0.19	0.07	0.27
YER011W	TIR1	474	417	1.14	0.19	0.07	0.27
YIL103W		12156	10686	1.14	0.19	0.07	0.27

YFL026W	STE2	1021	898	1.14	0.19	0.07	0.27
YPR077C		494	434	1.14	0.19	0.07	0.27
YNR064C		1805	1587	1.14	0.19	0.07	0.27
YDL217C	TIM22	3954	3477	1.14	0.19	0.07	0.27
YDR474C		2071	1822	1.14	0.18	0.07	0.26
YLR384C	IKI3	1120	985	1.14	0.18	0.07	0.26
YMR123W	PKR1	11244	9893	1.14	0.18	0.07	0.26
YHR049W		2716	2390	1.14	0.18	0.07	0.26
YIL057C		2108	1855	1.14	0.18	0.07	0.26
YGR212W		1832	1612	1.14	0.18	0.07	0.26
YHR077C	NMD2	17050	15004	1.14	0.18	0.07	0.26
YGL148W	ARO2	2221	1955	1.14	0.18	0.07	0.26
YGR007W	MUQ1	14324	12606	1.14	0.18	0.07	0.26
YEL016C		1763	1552	1.14	0.18	0.07	0.26
YDR516C		2968	2613	1.14	0.18	0.07	0.26
YMR042W	ARG80	3478	3062	1.14	0.18	0.07	0.26
YOR251C		3255	2866	1.14	0.18	0.07	0.26
YMR070W	MOT3	6055	5332	1.14	0.18	0.07	0.26
YHR211W	FLO5	4547	4005	1.14	0.18	0.07	0.26
YOR298W		1960	1727	1.14	0.18	0.07	0.26
YOR139C		1431	1261	1.14	0.18	0.07	0.26
YCR003W	MRPL32	10793	9509	1.14	0.18	0.07	0.26
YNR046W		14388	12677	1.13	0.18	0.07	0.26
YJR092W	BUD4	1344	1184	1.13	0.18	0.07	0.26
YFL061W		2794	2462	1.13	0.18	0.07	0.26
YKL072W	STB6	3301	2909	1.13	0.18	0.07	0.26
YJL203W	PRP21	3054	2691	1.13	0.18	0.07	0.26
YOR340C	RPA43	11092	9774	1.13	0.18	0.07	0.26
YCR054C	CTR86	2712	2390	1.13	0.18	0.07	0.25
YDL018C	ERP3	10267	9048	1.13	0.18	0.07	0.25
YPL132W	COX11	15709	13845	1.13	0.18	0.07	0.25
YIR028W	DAL4	1228	1082	1.13	0.18	0.07	0.25
YBR144C		1161	1023	1.13	0.18	0.07	0.25
YBL051C		5166	4554	1.13	0.18	0.07	0.25
YMR325W		1472	1298	1.13	0.18	0.07	0.25
YCR004C	YCP4	17617	15532	1.13	0.18	0.07	0.25

YIL114C	POR2	6693	5901	1.13	0.18	0.07	0.25
YBR151W	APD1	17967	15841	1.13	0.18	0.07	0.25
YCR087W		5280	4655	1.13	0.18	0.07	0.25
YOL077C		37795	33328	1.13	0.18	0.07	0.25
YJL186W	MNN5	830	732	1.13	0.18	0.07	0.25
YHL001W	RPL14B	31467	27759	1.13	0.18	0.07	0.25
YNL220W	ADE12	17366	15321	1.13	0.18	0.07	0.25
YGR270W	YTA7	4252	3751	1.13	0.18	0.07	0.25
YDL206W		1266	1117	1.13	0.18	0.07	0.25
YHR036W		8958	7905	1.13	0.18	0.07	0.25
YDR003W		1676	1479	1.13	0.18	0.07	0.25
YLR085C	ARP6	16808	14833	1.13	0.18	0.07	0.25
YPL172C	COX10	14975	13217	1.13	0.18	0.07	0.25
YNL027W	CRZ1	3062	2703	1.13	0.18	0.07	0.25
YDL163W		1825	1611	1.13	0.18	0.07	0.25
YHR162W		19356	17088	1.13	0.18	0.07	0.25
YPL068C		10038	8865	1.13	0.18	0.07	0.24
YFR031C	SMC2	745	658	1.13	0.18	0.07	0.24
YGL113W		3758	3319	1.13	0.18	0.07	0.24
YKR045C		1608	1420	1.13	0.18	0.07	0.24
YIL107C	PFK26	3353	2962	1.13	0.18	0.07	0.24
YGL075C	MPS2	3517	3107	1.13	0.18	0.07	0.24
YLR418C	CDC73	8555	7557	1.13	0.18	0.07	0.24
YGL257C	MNT2	7198	6358	1.13	0.18	0.07	0.24
YIL156W	UBP7	5616	4961	1.13	0.18	0.07	0.24
YCR059C		6226	5501	1.13	0.18	0.07	0.24
YPL134C		3675	3247	1.13	0.18	0.07	0.24
YDR529C	QCR7	17288	15277	1.13	0.18	0.07	0.24
YNL195C		1183	1045	1.13	0.18	0.07	0.24
YER023W	PRO3	3367	2976	1.13	0.18	0.07	0.24
YBR054W	YRO2	1876	1658	1.13	0.18	0.07	0.24
YNL268W	LYP1	13859	12252	1.13	0.18	0.07	0.24
YML046W	PRP39	3271	2892	1.13	0.18	0.07	0.24
YDR364C	CDC40	2052	1814	1.13	0.18	0.07	0.24
YGR181W	TIM13	7755	6856	1.13	0.18	0.07	0.24
YLR135W		4550	4022	1.13	0.18	0.07	0.24

YOL081W	IRA2	701	620	1.13	0.18	0.07	0.24
YOR042W		2130	1883	1.13	0.18	0.07	0.24
YNL290W	RFC3	6338	5604	1.13	0.18	0.07	0.24
YOR059C		1604	1418	1.13	0.18	0.07	0.24
YJR079W		759	671	1.13	0.18	0.06	0.24
YJL177W	RPL17B	30720	27176	1.13	0.18	0.06	0.23
YKL091C		2782	2461	1.13	0.18	0.06	0.23
YER133W	GLC7	21211	18765	1.13	0.18	0.06	0.23
YEL021W	URA3	6101	5398	1.13	0.18	0.06	0.23
YBR225W		4514	3994	1.13	0.18	0.06	0.23
YNL009W	IDP3	1187	1050	1.13	0.18	0.06	0.23
YPR147C		1943	1719	1.13	0.18	0.06	0.23
YNL165W		4328	3830	1.13	0.18	0.06	0.23
YGR196C		2079	1840	1.13	0.18	0.06	0.23
YFL001W	DEG1	4856	4297	1.13	0.18	0.06	0.23
YML050W		6226	5510	1.13	0.18	0.06	0.23
YKL019W	RAM2	5110	4523	1.13	0.18	0.06	0.23
YCR066W	RAD18	2772	2454	1.13	0.18	0.06	0.23
YBR182C	SMP1	1021	904	1.13	0.18	0.06	0.23
YGL043W	DST1	4670	4134	1.13	0.18	0.06	0.23
YHR047C	AAP1'	1528	1353	1.13	0.18	0.06	0.23
YHL022C	SPO11	826	731	1.13	0.18	0.06	0.23
YDR182W	CDC1	17260	15282	1.13	0.18	0.06	0.23
YDL162C		500	443	1.13	0.18	0.06	0.23
YOR195W	SLK19	7867	6966	1.13	0.18	0.06	0.23
YMR220W	ERG8	5340	4728	1.13	0.18	0.06	0.23
YBL073W		994	880	1.13	0.18	0.06	0.23
YGL061C	DUO1	9326	8258	1.13	0.18	0.06	0.23
YLR230W		7281	6448	1.13	0.18	0.06	0.23
YOR126C	IAH1	3075	2723	1.13	0.18	0.06	0.23
YMR127C	SAS2	2614	2316	1.13	0.17	0.06	0.23
YDR343C	HXT6	627	556	1.13	0.17	0.06	0.23
YOR311C		8124	7199	1.13	0.17	0.06	0.23
YAL047C	SPC72	3228	2860	1.13	0.17	0.06	0.23
YER064C		576	510	1.13	0.17	0.06	0.23
YLR177W		2826	2504	1.13	0.17	0.06	0.23

YBL052C	SAS3	9846	8726	1.13	0.17	0.06	0.22
YOL045W		3198	2834	1.13	0.17	0.06	0.22
YPR200C	ARR2	671	595	1.13	0.17	0.06	0.22
YBL048W		2071	1836	1.13	0.17	0.06	0.22
YGR057C	LST7	7170	6357	1.13	0.17	0.06	0.22
YPL116W	HOS3	3949	3502	1.13	0.17	0.06	0.22
YIL121W		3833	3400	1.13	0.17	0.06	0.22
YOR008C	SLG1	7979	7078	1.13	0.17	0.06	0.22
YHR087W		13374	11863	1.13	0.17	0.06	0.22
YPR003C		2409	2137	1.13	0.17	0.06	0.22
YGL093W	SPC105	1200	1065	1.13	0.17	0.06	0.22
YDR289C	RTT103	2931	2601	1.13	0.17	0.06	0.22
YOL082W		9702	8610	1.13	0.17	0.06	0.22
YHR167W		2891	2566	1.13	0.17	0.06	0.22
YLR180W	SAM1	11461	10172	1.13	0.17	0.06	0.22
YPR068C	HOS1	4216	3742	1.13	0.17	0.06	0.22
YLR427W		2880	2557	1.13	0.17	0.06	0.22
YJR006W	HYS2	18548	16466	1.13	0.17	0.06	0.22
YIL112W		3148	2795	1.13	0.17	0.06	0.22
YAL065C-A		1440	1278	1.13	0.17	0.06	0.22
YOR171C	LCB4	998	886	1.13	0.17	0.06	0.22
YOR202W	HIS3	26353	23398	1.13	0.17	0.06	0.22
YFL024C	EPL1	473	420	1.13	0.17	0.06	0.21
YNL113W	RPC19	17922	15914	1.13	0.17	0.06	0.21
YKR083C		5262	4673	1.13	0.17	0.06	0.21
YNL051W		10070	8943	1.13	0.17	0.06	0.21
YDL084W	SUB2	11995	10653	1.13	0.17	0.06	0.21
YPR046W	MCM16	9385	8336	1.13	0.17	0.06	0.21
YDR141C	DOP1	2921	2595	1.13	0.17	0.06	0.21
YBR112C	CYC8	6187	5497	1.13	0.17	0.06	0.21
YKL030W		2790	2479	1.13	0.17	0.06	0.21
YGR033C		17820	15833	1.13	0.17	0.06	0.21
YLL045C	RPL8B	13761	12227	1.13	0.17	0.06	0.21
YDR460W	TFB3	3591	3191	1.13	0.17	0.06	0.21
YPL179W	PPQ1	1426	1267	1.13	0.17	0.06	0.21
YOR033C	DHS1	1139	1012	1.13	0.17	0.06	0.21

YPL035C		2112	1877	1.13	0.17	0.06	0.21
YNL194C		1524	1355	1.12	0.17	0.06	0.21
YBR195C	MSI1	9822	8732	1.12	0.17	0.06	0.21
YGL249W	ZIP2	3267	2905	1.12	0.17	0.06	0.21
YML016C	PPZ1	9427	8382	1.12	0.17	0.06	0.21
YGL129C		10783	9588	1.12	0.17	0.06	0.21
YOR306C		2429	2160	1.12	0.17	0.06	0.21
YDR325W	YCG1	824	733	1.12	0.17	0.06	0.21
YJR050W	ISY1	5427	4826	1.12	0.17	0.06	0.21
YLR387C		7934	7056	1.12	0.17	0.06	0.21
YIL125W	KGD1	6365	5661	1.12	0.17	0.06	0.21
YHR068W	DYS1	1862	1656	1.12	0.17	0.06	0.21
YMR317W		1064	946	1.12	0.17	0.06	0.21
YLR362W	STE11	2602	2314	1.12	0.17	0.06	0.21
YOL155C		3389	3015	1.12	0.17	0.06	0.20
YAL043C-A		8012	7129	1.12	0.17	0.06	0.20
YCR030C		3573	3180	1.12	0.17	0.06	0.20
YBL084C	CDC27	1445	1286	1.12	0.17	0.06	0.20
YKL038W	RGT1	801	713	1.12	0.17	0.06	0.20
YMR279C		1362	1212	1.12	0.17	0.06	0.20
YMR088C		1992	1773	1.12	0.17	0.06	0.20
YDR522C	SPS2	686	611	1.12	0.17	0.06	0.20
YJL133W	MRS3	4789	4264	1.12	0.17	0.06	0.20
YER014W	HEM14	1981	1764	1.12	0.17	0.05	0.20
YBR181C	RPS6B	44600	39723	1.12	0.17	0.05	0.20
YBR157C	ICS2	1552	1382	1.12	0.17	0.05	0.20
YDR185C		1167	1040	1.12	0.17	0.05	0.20
YBR084C-A	RPL19A	25902	23074	1.12	0.17	0.05	0.20
YDR016C		7393	6586	1.12	0.17	0.05	0.20
YER095W	RAD51	2541	2264	1.12	0.17	0.05	0.20
YPR117W		984	877	1.12	0.17	0.05	0.20
YHL010C		2921	2603	1.12	0.17	0.05	0.20
YFR038W		7245	6456	1.12	0.17	0.05	0.20
YGR101W		6005	5353	1.12	0.17	0.05	0.19
YOR083W		556	496	1.12	0.17	0.05	0.19
YGL224C		9896	8823	1.12	0.17	0.05	0.19

YOR378W		1306	1164	1.12	0.17	0.05	0.19
YPL040C	ISM1	3516	3135	1.12	0.17	0.05	0.19
YGR031W		2797	2494	1.12	0.17	0.05	0.19
YKL074C	MUD2	9122	8135	1.12	0.17	0.05	0.19
YPR024W	YME1	9280	8276	1.12	0.17	0.05	0.19
YLR458W		3776	3368	1.12	0.16	0.05	0.19
YMR219W	ESC1	660	589	1.12	0.16	0.05	0.19
YJL006C	CTK2	10297	9185	1.12	0.16	0.05	0.19
YGR006W	PRP18	1301	1161	1.12	0.16	0.05	0.19
YGR041W	BUD9	4252	3793	1.12	0.16	0.05	0.19
YDL215C	GDH2	1456	1299	1.12	0.16	0.05	0.19
YLR442C	SIR3	964	860	1.12	0.16	0.05	0.19
YDR313C	PIB1	3550	3168	1.12	0.16	0.05	0.19
YDL151C		11632	10380	1.12	0.16	0.05	0.19
YNL330C	RPD3	6142	5481	1.12	0.16	0.05	0.19
YPR036W	VMA13	25249	22532	1.12	0.16	0.05	0.19
YLR058C	SHM2	5288	4719	1.12	0.16	0.05	0.19
YCR065W	HCM1	5722	5107	1.12	0.16	0.05	0.19
YOR179C		8807	7860	1.12	0.16	0.05	0.19
YNL066W	SUN4	29526	26356	1.12	0.16	0.05	0.19
YPR084W		6444	5753	1.12	0.16	0.05	0.19
YML095C-A		6078	5426	1.12	0.16	0.05	0.19
YKL048C	ELM1	1717	1533	1.12	0.16	0.05	0.19
YBR069C	VAP1	13963	12467	1.12	0.16	0.05	0.19
YKL050C		1307	1167	1.12	0.16	0.05	0.19
YMR104C	YPK2	1697	1515	1.12	0.16	0.05	0.18
YPR140W		3014	2692	1.12	0.16	0.05	0.18
YMR167W	MLH1	3710	3314	1.12	0.16	0.05	0.18
YBR094W		3172	2833	1.12	0.16	0.05	0.18
YDR273W		971	867	1.12	0.16	0.05	0.18
YLR116W	MSL5	3579	3197	1.12	0.16	0.05	0.18
YNR023W	SNF12	4116	3677	1.12	0.16	0.05	0.18
YBR258C		3597	3213	1.12	0.16	0.05	0.18
YOL089C	HAL9	2081	1859	1.12	0.16	0.05	0.18
YPR163C	TIF3	1284	1147	1.12	0.16	0.05	0.18
YMR302C	PRP12	4004	3578	1.12	0.16	0.05	0.18

YEL077C		1930	1725	1.12	0.16	0.05	0.18
YIL026C	IRR1	7854	7020	1.12	0.16	0.05	0.18
YKL177W		917	820	1.12	0.16	0.05	0.18
YCR107W	AAD3	2813	2515	1.12	0.16	0.05	0.18
YGR215W		9005	8050	1.12	0.16	0.05	0.18
YNL005C	MRP7	9219	8242	1.12	0.16	0.05	0.18
YPR085C		726	649	1.12	0.16	0.05	0.18
YGR233C	PHO81	1390	1243	1.12	0.16	0.05	0.18
YFL018C	LPD1	22163	19815	1.12	0.16	0.05	0.18
YMR258C		719	643	1.12	0.16	0.05	0.18
YNR072W	HXT17	2709	2423	1.12	0.16	0.05	0.18
YGL191W	COX13	8865	7928	1.12	0.16	0.05	0.18
YPR005C	HAL1	823	736	1.12	0.16	0.05	0.18
YMR064W	AEP1	8180	7316	1.12	0.16	0.05	0.18
YER170W	ADK2	9643	8625	1.12	0.16	0.05	0.18
YJL016W		1442	1290	1.12	0.16	0.05	0.18
YPR093C		2215	1981	1.12	0.16	0.05	0.18
YMR195W		7079	6332	1.12	0.16	0.05	0.18
YHR002W		6030	5395	1.12	0.16	0.05	0.18
YCR063W		4651	4161	1.12	0.16	0.05	0.17
YIL169C		6101	5459	1.12	0.16	0.05	0.17
YMR157C		4213	3770	1.12	0.16	0.05	0.17
YNR001C	CIT1	10932	9783	1.12	0.16	0.05	0.17
YKL020C	SPT23	489	438	1.12	0.16	0.05	0.17
YDR452W		1162	1040	1.12	0.16	0.05	0.17
YLR112W		2242	2007	1.12	0.16	0.05	0.17
YOR128C	ADE2	3224	2886	1.12	0.16	0.05	0.17
YHL018W		1422	1273	1.12	0.16	0.05	0.17
YHR092C	HXT4	5900	5283	1.12	0.16	0.05	0.17
YBR289W	SNF5	2257	2021	1.12	0.16	0.05	0.17
YHL042W		1506	1349	1.12	0.16	0.05	0.17
YCL024W		7189	6438	1.12	0.16	0.05	0.17
YKR072C	SIS2	5884	5269	1.12	0.16	0.05	0.17
YER134C		6595	5906	1.12	0.16	0.05	0.17
YNL329C	PEX6	10560	9458	1.12	0.16	0.05	0.17
YIL143C	SSL2	14399	12896	1.12	0.16	0.05	0.17

YNL218W		3762	3371	1.12	0.16	0.05	0.17
YDR444W		3997	3582	1.12	0.16	0.05	0.17
YEL062W	NPR2	1840	1649	1.12	0.16	0.05	0.17
YMR136W	GAT2	3036	2721	1.12	0.16	0.05	0.17
YLR242C	ARV1	7862	7046	1.12	0.16	0.05	0.17
YIL064W		14894	13351	1.12	0.16	0.05	0.17
YLR306W	UBC12	5281	4734	1.12	0.16	0.05	0.16
YGL232W		13699	12281	1.12	0.16	0.05	0.16
YCR019W	MAK32	7420	6654	1.12	0.16	0.04	0.16
YAL054C	ACS1	623	559	1.12	0.16	0.04	0.16
YBR015C	MNN2	6130	5497	1.12	0.16	0.04	0.16
YBR288C	APM3	5057	4535	1.12	0.16	0.04	0.16
YPL045W	VPS16	2370	2126	1.11	0.16	0.04	0.16
YDR524C		5623	5043	1.11	0.16	0.04	0.16
YMR205C	PFK2	3705	3323	1.11	0.16	0.04	0.16
YBR025C		16816	15084	1.11	0.16	0.04	0.16
YJL162C		3528	3165	1.11	0.16	0.04	0.16
YBR194W		2310	2073	1.11	0.16	0.04	0.16
YDR457W	TOM1	995	893	1.11	0.16	0.04	0.16
YBR161W		1308	1174	1.11	0.16	0.04	0.16
YPL142C		3508	3148	1.11	0.16	0.04	0.16
YLR375W	STP3	934	838	1.11	0.16	0.04	0.16
YBL101W-B		1457	1308	1.11	0.16	0.04	0.16
YBR008C	FLR1	999	897	1.11	0.16	0.04	0.16
YLR220W	CCC1	3687	3310	1.11	0.16	0.04	0.16
YPR029C	APL4	6201	5568	1.11	0.16	0.04	0.16
YKL054C		427	383	1.11	0.16	0.04	0.16
YJL032W		2462	2211	1.11	0.16	0.04	0.16
YJL012C		16727	15022	1.11	0.16	0.04	0.16
YBR187W		6793	6101	1.11	0.15	0.04	0.15
YIL073C	SPO22	857	770	1.11	0.15	0.04	0.15
YOL137W		4309	3871	1.11	0.15	0.04	0.15
YNR017W	MAS6	10957	9844	1.11	0.15	0.04	0.15
YMR119W-A		861	774	1.11	0.15	0.04	0.15
YKL087C	CYT2	7260	6525	1.11	0.15	0.04	0.15
YBL091C	MAP2	17696	15904	1.11	0.15	0.04	0.15

YJL213W		5956	5353	1.11	0.15	0.04	0.15
YIL167W		5757	5174	1.11	0.15	0.04	0.15
YDL080C	THI3	4748	4268	1.11	0.15	0.04	0.15
YDR044W	HEM13	3642	3274	1.11	0.15	0.04	0.15
YBR073W	RDH54	8947	8044	1.11	0.15	0.04	0.15
YBR014C		5263	4732	1.11	0.15	0.04	0.15
YBL015W	ACH1	1671	1502	1.11	0.15	0.04	0.15
YCR102C		2428	2183	1.11	0.15	0.04	0.15
YOR037W	CYC2	3762	3383	1.11	0.15	0.04	0.15
YDR216W	ADR1	7668	6896	1.11	0.15	0.04	0.15
YMR141C		2573	2314	1.11	0.15	0.04	0.15
YML129C	COX14	9229	8301	1.11	0.15	0.04	0.15
YBR130C	SHE3	6358	5719	1.11	0.15	0.04	0.15
YNL032W	SIW14	19461	17505	1.11	0.15	0.04	0.15
YGL260W		3214	2891	1.11	0.15	0.04	0.15
YHR088W		2779	2500	1.11	0.15	0.04	0.15
YFR033C	QCR6	17703	15925	1.11	0.15	0.04	0.15
YGR070W	ROM1	1452	1306	1.11	0.15	0.04	0.15
YKL015W	PUT3	1248	1123	1.11	0.15	0.04	0.15
YDR183W		3124	2811	1.11	0.15	0.04	0.14
YCRX04W		8865	7979	1.11	0.15	0.04	0.14
YCR031C	RPS14A	8715	7844	1.11	0.15	0.04	0.14
YKL011C	CCE1	4111	3700	1.11	0.15	0.04	0.14
YDL122W	UBP1	8627	7767	1.11	0.15	0.04	0.14
YOL135C	MED7	2144	1930	1.11	0.15	0.04	0.14
YPR105C		6667	6004	1.11	0.15	0.04	0.14
YKL012W	PRP40	5553	5002	1.11	0.15	0.04	0.14
YOR224C	RPB8	17502	15766	1.11	0.15	0.04	0.14
YDR074W	TPS2	10014	9021	1.11	0.15	0.04	0.14
YJR124C		6063	5462	1.11	0.15	0.04	0.14
YDR127W	ARO1	2875	2590	1.11	0.15	0.04	0.14
YJR109C	CPA2	1157	1042	1.11	0.15	0.04	0.14
YHR061C	GIC1	5785	5213	1.11	0.15	0.04	0.14
YJL176C	SWI3	4976	4484	1.11	0.15	0.04	0.14
YPL136W		1945	1753	1.11	0.15	0.04	0.14
YDL067C	COX9	9316	8396	1.11	0.15	0.04	0.14

YNL274C		2135	1925	1.11	0.15	0.04	0.14
YDR429C	TIF35	29074	26213	1.11	0.15	0.04	0.13
YKR064W		1623	1463	1.11	0.15	0.04	0.13
YDR305C	HNT2	5567	5019	1.11	0.15	0.04	0.13
YLL036C	PRP19	5695	5136	1.11	0.15	0.04	0.13
YIR015W	RPR2	1215	1096	1.11	0.15	0.04	0.13
YLR221C		6545	5903	1.11	0.15	0.04	0.13
YNR028W	CPR8	9013	8129	1.11	0.15	0.04	0.13
YJL007C		904	815	1.11	0.15	0.04	0.13
YMR140W		4253	3836	1.11	0.15	0.04	0.13
YOR205C		11941	10771	1.11	0.15	0.04	0.13
YGR228W		8938	8062	1.11	0.15	0.04	0.13
YPL007C		8474	7644	1.11	0.15	0.04	0.13
YCR041W		10001	9022	1.11	0.15	0.04	0.13
YFL035C-A		31198	28145	1.11	0.15	0.04	0.13
YHR005C	GPA1	15757	14215	1.11	0.15	0.04	0.13
YOR141C	ARP8	7546	6808	1.11	0.15	0.04	0.13
YMR310C		14789	13343	1.11	0.15	0.04	0.13
YOL042W		10353	9341	1.11	0.15	0.04	0.13
YPL228W	CET1	2751	2482	1.11	0.15	0.04	0.13
YLR034C	SMF3	13840	12490	1.11	0.15	0.04	0.13
YIL157C		10519	9496	1.11	0.15	0.04	0.13
YCR100C		730	659	1.11	0.15	0.04	0.13
YML020W		7018	6336	1.11	0.15	0.04	0.13
YML076C		1337	1207	1.11	0.15	0.04	0.13
YML118W		432	390	1.11	0.15	0.04	0.13
YCL022C		4022	3631	1.11	0.15	0.04	0.13
YER027C	GAL83	2386	2154	1.11	0.15	0.03	0.13
YMR172C-A		1207	1090	1.11	0.15	0.03	0.13
YDR247W		1213	1095	1.11	0.15	0.03	0.13
YDL056W	MBP1	2763	2495	1.11	0.15	0.03	0.13
YHR153C	SPO16	5567	5028	1.11	0.15	0.03	0.13
YCLX10C		8949	8083	1.11	0.15	0.03	0.13
YIR044C		3265	2949	1.11	0.15	0.03	0.12
YER022W	SRB4	1455	1314	1.11	0.15	0.03	0.12
YJL029C		4217	3810	1.11	0.15	0.03	0.12

YJL013C	MAD3	4130	3731	1.11	0.15	0.03	0.12
YGR151C		3844	3473	1.11	0.15	0.03	0.12
YDR203W		1442	1303	1.11	0.15	0.03	0.12
YKR089C		1174	1061	1.11	0.15	0.03	0.12
YNL116W		4293	3879	1.11	0.15	0.03	0.12
YER049W		21426	19362	1.11	0.15	0.03	0.12
YLR196W	PWP1	4429	4003	1.11	0.15	0.03	0.12
YOR375C	GDH1	2889	2611	1.11	0.15	0.03	0.12
YDL024C		1751	1583	1.11	0.15	0.03	0.12
YMR023C	MSS1	4347	3930	1.11	0.15	0.03	0.12
YER069W	ARG5,6	388	351	1.11	0.15	0.03	0.12
YNL056W		13420	12133	1.11	0.15	0.03	0.12
YHR019C	DED81	22707	20529	1.11	0.15	0.03	0.12
YER029C	SMB1	8190	7405	1.11	0.15	0.03	0.12
YMR044W		2276	2058	1.11	0.15	0.03	0.12
YOR276W	CAF20	24257	21933	1.11	0.15	0.03	0.12
YMR182C	RGM1	2513	2272	1.11	0.15	0.03	0.12
YDR227W	SIR4	1135	1026	1.11	0.15	0.03	0.12
YFL013C		8165	7384	1.11	0.15	0.03	0.12
YIL083C		3923	3548	1.11	0.15	0.03	0.12
YDL235C	YPD1	4224	3820	1.11	0.15	0.03	0.12
YHR120W	MSH1	3043	2752	1.11	0.15	0.03	0.12
YPL120W	VPS30	7589	6863	1.11	0.14	0.03	0.12
YOR211C	MGM1	827	748	1.11	0.14	0.03	0.12
YNL100W		11234	10161	1.11	0.14	0.03	0.12
YDL107W	MSS2	2018	1825	1.11	0.14	0.03	0.12
YOR114W		1803	1631	1.11	0.14	0.03	0.12
YJL220W		942	852	1.11	0.14	0.03	0.12
YLR148W	PEP3	1137	1029	1.11	0.14	0.03	0.12
YOR392W		392	355	1.11	0.14	0.03	0.12
YDL083C	RPS16B	27572	24948	1.11	0.14	0.03	0.12
YIL058W		3433	3106	1.11	0.14	0.03	0.12
YBL005W	PDR3	3800	3439	1.11	0.14	0.03	0.12
YDR319C		3308	2994	1.10	0.14	0.03	0.11
YOR006C		12242	11080	1.10	0.14	0.03	0.11
YHR195W	VAB36	2483	2247	1.10	0.14	0.03	0.11

YDR178W	SDH4	4087	3700	1.10	0.14	0.03	0.11
YDR126W		3684	3335	1.10	0.14	0.03	0.11
YFR028C	CDC14	4682	4239	1.10	0.14	0.03	0.11
YAL035C-A		2362	2139	1.10	0.14	0.03	0.11
YGL262W		563	510	1.10	0.14	0.03	0.11
YPR091C		3140	2844	1.10	0.14	0.03	0.11
YKL200C		734	665	1.10	0.14	0.03	0.11
YHR028C	DAP2	16023	14515	1.10	0.14	0.03	0.11
YBR010W	HHT1	30992	28076	1.10	0.14	0.03	0.11
YDR436W	PPZ2	2305	2088	1.10	0.14	0.03	0.11
YOR120W	GCY1	2045	1853	1.10	0.14	0.03	0.11
YLR083C	EMP70	16973	15380	1.10	0.14	0.03	0.11
YDR290W		2187	1982	1.10	0.14	0.03	0.11
YEL017C-A	PMP2	22931	20783	1.10	0.14	0.03	0.11
YNL163C		3174	2877	1.10	0.14	0.03	0.11
YGL115W	SNF4	16667	15107	1.10	0.14	0.03	0.11
YOL125W		3332	3020	1.10	0.14	0.03	0.11
YOR201C	PET56	13158	11928	1.10	0.14	0.03	0.11
YLR132C		1982	1797	1.10	0.14	0.03	0.11
YEL012W	UBC8	1856	1683	1.10	0.14	0.03	0.11
YML008C	ERG6	26932	24417	1.10	0.14	0.03	0.11
YHR083W		1388	1258	1.10	0.14	0.03	0.11
YBR154C	RPB5	28663	25987	1.10	0.14	0.03	0.11
YHR196W		1141	1035	1.10	0.14	0.03	0.11
YNL041C		3396	3079	1.10	0.14	0.03	0.11
YEL068C		5325	4828	1.10	0.14	0.03	0.10
YDL220C	CDC13	3024	2742	1.10	0.14	0.03	0.10
YOR258W		7655	6945	1.10	0.14	0.03	0.10
YFL007W	BLM3	6344	5756	1.10	0.14	0.03	0.10
YML115C	VAN1	8841	8021	1.10	0.14	0.03	0.10
YDR478W	SNM1	3587	3255	1.10	0.14	0.03	0.10
YLL004W	ORC3	3813	3460	1.10	0.14	0.03	0.10
YLR247C		4734	4297	1.10	0.14	0.03	0.10
YGR200C		13888	12606	1.10	0.14	0.03	0.10
YIL070C	MAM33	24301	22060	1.10	0.14	0.03	0.10
YDR367W		12688	11518	1.10	0.14	0.03	0.10

YPL276W		964	875	1.10	0.14	0.03	0.10
YLR246W	ERF2	9416	8549	1.10	0.14	0.03	0.10
YLR170C	APS1	14756	13398	1.10	0.14	0.03	0.10
YDR483W	KRE2	4468	4057	1.10	0.14	0.03	0.10
YFR037C	RSC8	10694	9712	1.10	0.14	0.03	0.10
YML059C		6334	5752	1.10	0.14	0.03	0.10
YNL109W		7136	6483	1.10	0.14	0.03	0.09
YJL222W	VTH2	2186	1986	1.10	0.14	0.03	0.09
YFR029W	PTR3	5213	4736	1.10	0.14	0.03	0.09
YPL049C	DIG1	3036	2759	1.10	0.14	0.03	0.09
YFL045C	SEC53	38883	35334	1.10	0.14	0.03	0.09
YDL048C	STP4	6071	5517	1.10	0.14	0.03	0.09
YLR244C	MAP1	21716	19734	1.10	0.14	0.03	0.09
YLR049C		15367	13965	1.10	0.14	0.03	0.09
YJL123C		8923	8109	1.10	0.14	0.03	0.09
YDL102W	CDC2	947	861	1.10	0.14	0.03	0.09
YFL055W	AGP3	1951	1773	1.10	0.14	0.03	0.09
YDR109C		2638	2398	1.10	0.14	0.03	0.09
YHR124W	NDT80	4600	4181	1.10	0.14	0.03	0.09
YNL297C		3218	2925	1.10	0.14	0.03	0.09
YNL278W		4046	3678	1.10	0.14	0.03	0.09
YDL183C		5799	5272	1.10	0.14	0.03	0.09
YDR526C		1722	1566	1.10	0.14	0.03	0.09
YKL106W	AAT1	6793	6176	1.10	0.14	0.02	0.09
YEL041W		1013	921	1.10	0.14	0.02	0.09
YGL126W	SCS3	1114	1013	1.10	0.14	0.02	0.09
YIR005W	IST3	2941	2674	1.10	0.14	0.02	0.09
YNL302C	RPS19B	27154	24694	1.10	0.14	0.02	0.09
YGL244W	RTF1	15095	13728	1.10	0.14	0.02	0.09
YCL065W		13327	12122	1.10	0.14	0.02	0.09
YFR054C		735	669	1.10	0.14	0.02	0.09
YDL221W		370	337	1.10	0.14	0.02	0.09
YKL165C	MCD4	640	582	1.10	0.14	0.02	0.09
YPL192C		2176	1980	1.10	0.14	0.02	0.09
YBR093C	PHO5	550	500	1.10	0.14	0.02	0.09
YBL054W		582	530	1.10	0.14	0.02	0.09

YOR229W	WTM2	6764	6156	1.10	0.14	0.02	0.09
YJR034W	PET191	8891	8092	1.10	0.14	0.02	0.09
YDR467C		3337	3037	1.10	0.14	0.02	0.09
YBL018C	POP8	10201	9284	1.10	0.14	0.02	0.09
YBR237W	PRP5	5590	5088	1.10	0.14	0.02	0.08
YKL112W	ABF1	5416	4930	1.10	0.14	0.02	0.08
YOR245C		4118	3748	1.10	0.14	0.02	0.08
YJR054W		923	840	1.10	0.14	0.02	0.08
YFL059W	SNZ3	3103	2825	1.10	0.14	0.02	0.08
YDL182W	LYS20	19882	18102	1.10	0.14	0.02	0.08
YOL146W		5769	5253	1.10	0.14	0.02	0.08
YNL167C	SKO1	3244	2954	1.10	0.14	0.02	0.08
YOL017W		2819	2567	1.10	0.14	0.02	0.08
YOR217W	RFC1	1880	1712	1.10	0.13	0.02	0.08
YBR252W	DUT1	11128	10136	1.10	0.13	0.02	0.08
YDR480W	DIG2	2536	2310	1.10	0.13	0.02	0.08
YDR170C	SEC7	947	863	1.10	0.13	0.02	0.08
YHR142W	CHS7	5014	4568	1.10	0.13	0.02	0.08
YDR417C		25159	22923	1.10	0.13	0.02	0.08
YOL134C		5166	4707	1.10	0.13	0.02	0.08
YMR244C-A		2165	1973	1.10	0.13	0.02	0.08
YDR017C	KCS1	879	801	1.10	0.13	0.02	0.08
YLR143W		5862	5342	1.10	0.13	0.02	0.08
YMR178W		2062	1879	1.10	0.13	0.02	0.08
YKL144C	RPC25	6354	5792	1.10	0.13	0.02	0.08
YOR199W		4820	4394	1.10	0.13	0.02	0.08
YER059W	PCL6	3221	2936	1.10	0.13	0.02	0.08
YNL137C	NAM9	11434	10426	1.10	0.13	0.02	0.08
YDR068W	DOS2	16197	14769	1.10	0.13	0.02	0.08
YNL294C		4180	3812	1.10	0.13	0.02	0.08
YPL051W	ARL3	4424	4034	1.10	0.13	0.02	0.08
YJR144W	MGM101	14187	12938	1.10	0.13	0.02	0.07
YOR158W	PET123	4034	3679	1.10	0.13	0.02	0.07
YHR174W	ENO2	1169	1066	1.10	0.13	0.02	0.07
YNL030W	HHF2	25001	22802	1.10	0.13	0.02	0.07
YOR387C		2625	2394	1.10	0.13	0.02	0.07

YOL063C		3121	2847	1.10	0.13	0.02	0.07
YMR211W		2163	1973	1.10	0.13	0.02	0.07
YNL273W	TOF1	3599	3284	1.10	0.13	0.02	0.07
YOR017W	PET127	1337	1220	1.10	0.13	0.02	0.07
YCR105W		2176	1986	1.10	0.13	0.02	0.07
YLR450W	HMG2	1375	1255	1.10	0.13	0.02	0.07
YDL186W		828	756	1.10	0.13	0.02	0.07
YCR075C	ERS1	4427	4040	1.10	0.13	0.02	0.07
YEL054C	RPL12A	21651	19765	1.10	0.13	0.02	0.07
YBR136W	ESR1	711	649	1.10	0.13	0.02	0.07
YMR153C-A		5351	4885	1.10	0.13	0.02	0.07
YMR283C	RIT1	5623	5134	1.10	0.13	0.02	0.07
YLR277C	YSH1	5113	4669	1.10	0.13	0.02	0.07
YDR198C		8403	7674	1.10	0.13	0.02	0.07
YBL060W		9464	8643	1.10	0.13	0.02	0.07
YIL002C	INP51	2975	2717	1.09	0.13	0.02	0.07
YBR135W	CKS1	9182	8387	1.09	0.13	0.02	0.07
YER084W		1275	1165	1.09	0.13	0.02	0.07
YPR130C		6421	5865	1.09	0.13	0.02	0.07
YPL017C		781	713	1.09	0.13	0.02	0.07
YHR178W	STB5	4171	3810	1.09	0.13	0.02	0.07
YGR090W		11713	10700	1.09	0.13	0.02	0.07
YIL030C	SSM4	6037	5515	1.09	0.13	0.02	0.07
YKL221W		1738	1588	1.09	0.13	0.02	0.06
YOL151W	GRE2	5218	4769	1.09	0.13	0.02	0.06
YDR081C	PDC2	6179	5648	1.09	0.13	0.02	0.06
YKL146W		1884	1722	1.09	0.13	0.02	0.06
YMR202W	ERG2	11333	10360	1.09	0.13	0.02	0.06
YAL041W	CDC24	3537	3233	1.09	0.13	0.02	0.06
YDR475C		1325	1211	1.09	0.13	0.02	0.06
YOR310C	NOP58	11173	10215	1.09	0.13	0.02	0.06
YFR007W		5103	4666	1.09	0.13	0.02	0.06
YJR013W		787	720	1.09	0.13	0.02	0.06
YGL058W	RAD6	10827	9901	1.09	0.13	0.02	0.06
YOR263C		4394	4018	1.09	0.13	0.02	0.06
YKL034W		2617	2393	1.09	0.13	0.02	0.06

YDL212W	SHR3	5580	5104	1.09	0.13	0.02	0.06
YDR032C		12571	11499	1.09	0.13	0.02	0.06
YDL047W	SIT4	4889	4472	1.09	0.13	0.02	0.06
YNL047C		8776	8029	1.09	0.13	0.02	0.06
YOR163W		1483	1357	1.09	0.13	0.02	0.06
YGR263C		3090	2827	1.09	0.13	0.02	0.06
YGR238C	KEL2	3817	3493	1.09	0.13	0.02	0.06
YDR501W	PLM2	2096	1918	1.09	0.13	0.02	0.06
YNL178W	RPS3	34578	31647	1.09	0.13	0.02	0.06
YBL024W	NCL1	14504	13276	1.09	0.13	0.02	0.06
YBL003C	HTA2	21769	19929	1.09	0.13	0.02	0.05
YGR203W		2215	2028	1.09	0.13	0.01	0.05
YNL306W		11289	10339	1.09	0.13	0.01	0.05
YKL093W	MBR1	1508	1381	1.09	0.13	0.01	0.05
YOL092W		23062	21122	1.09	0.13	0.01	0.05
YGR004W		2446	2240	1.09	0.13	0.01	0.05
YHR210C		8580	7859	1.09	0.13	0.01	0.05
YJL076W	NET1	11613	10638	1.09	0.13	0.01	0.05
YMR013C	SEC59	8108	7428	1.09	0.13	0.01	0.05
YJR151C		1820	1667	1.09	0.13	0.01	0.05
YGL035C	MIG1	12154	11136	1.09	0.13	0.01	0.05
YIL177C		12238	11213	1.09	0.13	0.01	0.05
YPL062W		649	595	1.09	0.13	0.01	0.05
YHR034C		17301	15854	1.09	0.13	0.01	0.05
YIL090W		7463	6839	1.09	0.13	0.01	0.05
YGR234W	YHB1	50314	46111	1.09	0.13	0.01	0.05
YJL153C	INO1	567	520	1.09	0.13	0.01	0.05
YCR103C		1556	1426	1.09	0.13	0.01	0.05
YMR262W		2528	2317	1.09	0.13	0.01	0.05
YMR083W	ADH3	13884	12729	1.09	0.13	0.01	0.05
YAL032C	PRP45	10465	9595	1.09	0.13	0.01	0.05
YOR374W	ALD4	6474	5937	1.09	0.13	0.01	0.05
YPL083C	SEN54	3703	3396	1.09	0.12	0.01	0.05
YLR045C	STU2	10443	9577	1.09	0.12	0.01	0.05
YGR171C	MSM1	2241	2055	1.09	0.12	0.01	0.04
YDR335W	MSN5	2164	1985	1.09	0.12	0.01	0.04

YDL202W	MRPL11	6833	6268	1.09	0.12	0.01	0.04
YLR118C		3925	3601	1.09	0.12	0.01	0.04
YKL191W	DPH2	10492	9627	1.09	0.12	0.01	0.04
YPR169W		28215	25892	1.09	0.12	0.01	0.04
YLR286C	CTS1	30314	27819	1.09	0.12	0.01	0.04
YLR291C	GCD7	16838	15452	1.09	0.12	0.01	0.04
YLR117C	SYF3	10327	9477	1.09	0.12	0.01	0.04
YDL230W	PTP1	5014	4602	1.09	0.12	0.01	0.04
YGR076C	MRPL25	6243	5730	1.09	0.12	0.01	0.04
YHR175W	CTR2	8362	7675	1.09	0.12	0.01	0.04
YLR198C		8480	7783	1.09	0.12	0.01	0.04
YGR134W		7028	6451	1.09	0.12	0.01	0.04
YDR453C		3100	2846	1.09	0.12	0.01	0.04
YOL044W	PEX15	7300	6701	1.09	0.12	0.01	0.04
YDL140C	RPO21	2969	2726	1.09	0.12	0.01	0.04
YCRX05W		707	649	1.09	0.12	0.01	0.04
YDR148C	KGD2	17378	15958	1.09	0.12	0.01	0.04
YDL106C	GRF10	4072	3740	1.09	0.12	0.01	0.04
YCL068C		556	511	1.09	0.12	0.01	0.04
YML104C	MDM1	802	737	1.09	0.12	0.01	0.04
YGL205W	POX1	738	678	1.09	0.12	0.01	0.04
YDR310C	SUM1	5779	5308	1.09	0.12	0.01	0.04
YER114C	BOI2	3571	3280	1.09	0.12	0.01	0.04
YOL037C		1887	1733	1.09	0.12	0.01	0.04
YGL208W	SIP2	3591	3299	1.09	0.12	0.01	0.04
YGL059W		3803	3494	1.09	0.12	0.01	0.04
YPL174C	NIP100	2509	2305	1.09	0.12	0.01	0.04
YMR284W	YKU70	2265	2082	1.09	0.12	0.01	0.03
YLR399C	BDF1	13616	12515	1.09	0.12	0.01	0.03
YHR085W		29057	26709	1.09	0.12	0.01	0.03
YER019W		1230	1131	1.09	0.12	0.01	0.03
YCL008C	STP22	3199	2941	1.09	0.12	0.01	0.03
YPR137W	RRP9	644	592	1.09	0.12	0.01	0.03
YML120C	NDI1	5475	5035	1.09	0.12	0.01	0.03
YPR152C		1174	1080	1.09	0.12	0.01	0.03
YBR245C	ISW1	711	654	1.09	0.12	0.01	0.03

YKR036C	CAF4	1109	1020	1.09	0.12	0.01	0.03
YLR181C		4819	4434	1.09	0.12	0.01	0.03
YNL084C	END3	16070	14788	1.09	0.12	0.01	0.03
YOR165W		15974	14701	1.09	0.12	0.01	0.03
YGL005C		15139	13936	1.09	0.12	0.01	0.03
YER083C		3195	2941	1.09	0.12	0.01	0.03
YJR078W		719	662	1.09	0.12	0.01	0.03
YBR002C	RER2	3228	2972	1.09	0.12	0.01	0.02
YPL128C	TBF1	9070	8353	1.09	0.12	0.01	0.02
YMR109W	MYO5	4411	4063	1.09	0.12	0.01	0.02
YPL222W		823	758	1.09	0.12	0.01	0.02
YPL279C		17688	16293	1.09	0.12	0.01	0.02
YHL014C	YLF2	2373	2186	1.09	0.12	0.01	0.02
YHL038C	CBP2	554	510	1.09	0.12	0.01	0.02
YKL122C	SRP21	9345	8609	1.09	0.12	0.01	0.02
YDR235W	PRP42	980	903	1.09	0.12	0.01	0.02
YPL202C		12812	11804	1.09	0.12	0.01	0.02
YOR136W	IDH2	16186	14914	1.09	0.12	0.01	0.02
YOR304W	ISW2	1215	1120	1.09	0.12	0.01	0.02
YKL025C	PAN3	5266	4853	1.09	0.12	0.01	0.02
YHR057C	CYP2	2830	2608	1.09	0.12	0.01	0.02
YBR200W	BEM1	2729	2515	1.08	0.12	0.01	0.02
YMR053C	STB2	2692	2481	1.08	0.12	0.01	0.02
YIR023W	DAL81	4042	3726	1.08	0.12	0.01	0.02
YDR470C		1907	1758	1.08	0.12	0.01	0.02
YNL090W	RHO2	10961	10106	1.08	0.12	0.00	0.02
YHR039C		20992	19355	1.08	0.12	0.00	0.02
YKR092C	SRP40	2807	2588	1.08	0.12	0.00	0.02
YDR234W	LYS4	1657	1528	1.08	0.12	0.00	0.02
YGR113W	DAM1	4089	3771	1.08	0.12	0.00	0.02
YOR051C		13289	12258	1.08	0.12	0.00	0.02
YGL036W	MTC2	4478	4132	1.08	0.12	0.00	0.01
YPL229W		8056	7433	1.08	0.12	0.00	0.01
YOR279C		4101	3784	1.08	0.12	0.00	0.01
YNL315C	ATP11	26160	24138	1.08	0.12	0.00	0.01
YJR043C	POL32	4466	4121	1.08	0.12	0.00	0.01

YPL150W		3971	3665	1.08	0.12	0.00	0.01
YFR022W		1681	1552	1.08	0.12	0.00	0.01
YOR053W		6319	5832	1.08	0.12	0.00	0.01
YMR326C		887	819	1.08	0.12	0.00	0.01
YOR287C		5594	5164	1.08	0.12	0.00	0.01
YIL128W	MET18	5003	4619	1.08	0.12	0.00	0.01
YCR073C	SSK22	1132	1045	1.08	0.12	0.00	0.01
YNL279W		3389	3129	1.08	0.12	0.00	0.01
YMR055C	BUB2	3482	3215	1.08	0.12	0.00	0.01
YOR155C		360	332	1.08	0.11	0.00	0.01
YOL159C		2245	2073	1.08	0.11	0.00	0.01
YKL032C	IXR1	2660	2457	1.08	0.11	0.00	0.01
YFR011C		12837	11857	1.08	0.11	0.00	0.01
YPR020W	ATP20	18340	16941	1.08	0.11	0.00	0.01
YML086C	ALO1	5323	4918	1.08	0.11	0.00	0.01
YNL183C	NPR1	4131	3817	1.08	0.11	0.00	0.01
YMR156C		2536	2343	1.08	0.11	0.00	0.01
YPL017C		6104	5640	1.08	0.11	0.00	0.01
YPL197C		42835	39580	1.08	0.11	0.00	0.01
YJR090C	GRR1	2462	2275	1.08	0.11	0.00	0.01
YNR018W		22124	20444	1.08	0.11	0.00	0.01
YMR096W	SNZ1	2062	1905	1.08	0.11	0.00	0.01
YJR084W		1750	1617	1.08	0.11	0.00	0.01
YPR069C	SPE3	24506	22646	1.08	0.11	0.00	0.01
YPL130W	SPO19	1277	1180	1.08	0.11	0.00	0.01
YGR084C	MRP13	12126	11208	1.08	0.11	0.00	0.00
YDR536W	STL1	2031	1878	1.08	0.11	0.00	0.00
YJL183W	MNN11	5896	5451	1.08	0.11	0.00	0.00
YER127W	LCP5	4342	4014	1.08	0.11	0.00	0.00
YMR298W		12466	11527	1.08	0.11	0.00	0.00
YDL210W	UGA4	849	785	1.08	0.11	0.00	0.00
YDR168W	CDC37	14778	13669	1.08	0.11	0.00	0.00
YLR301W		12379	11453	1.08	0.11	0.00	0.00
YDR251W	PAM1	1332	1232	1.08	0.11	0.00	0.00
YAL033W	POP5	3463	3204	1.08	0.11	0.00	0.00
YMR214W	SCJ1	2569	2377	1.08	0.11	0.00	0.00

YOR372C	NDD1	4411	4083	1.08	0.11	0.00	0.00
YGR219W		7389	6839	1.08	0.11	0.00	0.00
YGR135W	PRE9	10619	9829	1.08	0.11	0.00	0.00
YDL043C	PRP11	2900	2684	1.08	0.11	0.00	0.00
YAR003W		6010	5563	1.08	0.11	0.00	0.00
YPR189W	SKI3	3411	3158	1.08	0.11	0.00	0.00
YEL048C		12190	11289	1.08	0.11	0.00	-0.01
YGL182C		1053	975	1.08	0.11	0.00	-0.01
YOL108C	INO4	7478	6927	1.08	0.11	0.00	-0.01
YER121W		2083	1930	1.08	0.11	0.00	-0.01
YOL093W		13341	12359	1.08	0.11	0.00	-0.01
YKR038C		8994	8332	1.08	0.11	0.00	-0.01
YBL096C		2749	2547	1.08	0.11	0.00	-0.01
YOL062C	APM4	11785	10919	1.08	0.11	0.00	-0.01
YJR135C	MCM22	3293	3052	1.08	0.11	0.00	-0.01
YHR073W		3666	3398	1.08	0.11	0.00	-0.01
YJR001W		10379	9619	1.08	0.11	0.00	-0.01
YJL103C		1024	949	1.08	0.11	0.00	-0.01
YOR246C		17386	16114	1.08	0.11	0.00	-0.01
YDR093W		6737	6244	1.08	0.11	0.00	-0.01
YJR100C		5501	5099	1.08	0.11	0.00	-0.01
YHR037W	PUT2	358	332	1.08	0.11	0.00	-0.01
YPR087W		3996	3704	1.08	0.11	0.00	-0.01
YJR096W		4757	4410	1.08	0.11	0.00	-0.01
YNL074C	MLF3	16808	15581	1.08	0.11	0.00	-0.01
YPR097W		5163	4787	1.08	0.11	0.00	-0.01
YGL081W		2060	1910	1.08	0.11	0.00	-0.01
YMR114C		2704	2507	1.08	0.11	0.00	-0.01
YJL134W	LCB3	20401	18916	1.08	0.11	0.00	-0.01
YJL024C	APS3	6856	6357	1.08	0.11	0.00	-0.01
YMR304W	UBP15	5099	4729	1.08	0.11	0.00	-0.01
YJR041C		983	912	1.08	0.11	0.00	-0.02
YBL027W	RPL19B	29033	26940	1.08	0.11	0.00	-0.02
YPL031C	PHO85	5176	4803	1.08	0.11	0.00	-0.02
YML002W		1613	1497	1.08	0.11	0.00	-0.02
YLR208W	SEC13	9517	8834	1.08	0.11	0.00	-0.02

YPR059C		2551	2368	1.08	0.11	-0.01	-0.02
YOR304C-A		4469	4149	1.08	0.11	-0.01	-0.02
YJL082W	IML2	7509	6972	1.08	0.11	-0.01	-0.02
YLR040C		14063	13058	1.08	0.11	-0.01	-0.02
YLR035C		1948	1809	1.08	0.11	-0.01	-0.02
YMR120C	ADE17	6452	5992	1.08	0.11	-0.01	-0.02
YMR244W		522	485	1.08	0.11	-0.01	-0.02
YPR146C		1247	1158	1.08	0.11	-0.01	-0.02
YMR084W		725	673	1.08	0.11	-0.01	-0.02
YKL088W		11196	10400	1.08	0.11	-0.01	-0.02
YJL009W		4776	4437	1.08	0.11	-0.01	-0.02
YMR227C	TAF67	2328	2163	1.08	0.11	-0.01	-0.02
YFR014C	CMK1	4709	4376	1.08	0.11	-0.01	-0.02
YER098W	UBP9	1803	1676	1.08	0.11	-0.01	-0.03
YGL066W		919	854	1.08	0.11	-0.01	-0.03
YGR001C		14757	13720	1.08	0.11	-0.01	-0.03
YDR206W	EBS1	7891	7336	1.08	0.11	-0.01	-0.03
YBR032W		2463	2291	1.08	0.10	-0.01	-0.03
YMR277W	FCP1	3783	3518	1.08	0.10	-0.01	-0.03
YGR075C	PRP38	7999	7440	1.08	0.10	-0.01	-0.03
YNL196C	SLZ1	1958	1821	1.08	0.10	-0.01	-0.03
YER136W	GDI1	22358	20798	1.08	0.10	-0.01	-0.03
YLR228C	ECM22	9636	8964	1.08	0.10	-0.01	-0.03
YPR191W	QCR2	23140	21527	1.07	0.10	-0.01	-0.03
YNL070W	TOM7	12971	12067	1.07	0.10	-0.01	-0.03
YNL299W	TRF5	5245	4880	1.07	0.10	-0.01	-0.03
YHR116W		3404	3167	1.07	0.10	-0.01	-0.03
YDR534C		1025	954	1.07	0.10	-0.01	-0.03
YLR022C		14493	13486	1.07	0.10	-0.01	-0.03
YCL067C	HMLALPHA2	23671	22028	1.07	0.10	-0.01	-0.03
YNL301C	RPL18B	41036	38198	1.07	0.10	-0.01	-0.03
YNR048W		10097	9400	1.07	0.10	-0.01	-0.03
YNL271C	BNI1	3789	3528	1.07	0.10	-0.01	-0.03
YDL174C	DLD1	2160	2011	1.07	0.10	-0.01	-0.03
YCR090C		13120	12215	1.07	0.10	-0.01	-0.03
YJL131C		2335	2174	1.07	0.10	-0.01	-0.03

YDR487C	RIB3	11647	10845	1.07	0.10	-0.01	-0.03
YKL185W	ASH1	3185	2966	1.07	0.10	-0.01	-0.03
YGR256W	GND2	7471	6957	1.07	0.10	-0.01	-0.03
YOR331C		24998	23282	1.07	0.10	-0.01	-0.04
YPR155C	NCA2	1424	1327	1.07	0.10	-0.01	-0.04
YPR061C		1404	1308	1.07	0.10	-0.01	-0.04
YBL095W		3906	3639	1.07	0.10	-0.01	-0.04
YOR168W	GLN4	11217	10451	1.07	0.10	-0.01	-0.04
YDR137W	RGP1	5943	5537	1.07	0.10	-0.01	-0.04
YKL149C	DBR1	5783	5388	1.07	0.10	-0.01	-0.04
YLR003C		13691	12757	1.07	0.10	-0.01	-0.04
YOR212W	STE4	9949	9272	1.07	0.10	-0.01	-0.04
YGL117W		16585	15458	1.07	0.10	-0.01	-0.04
YGL230C		1241	1157	1.07	0.10	-0.01	-0.04
YJR129C		902	841	1.07	0.10	-0.01	-0.04
YJR133W	XPT1	7933	7395	1.07	0.10	-0.01	-0.04
YOR129C		1021	952	1.07	0.10	-0.01	-0.04
YER021W	RPN3	6167	5749	1.07	0.10	-0.01	-0.04
YNL260C		3720	3468	1.07	0.10	-0.01	-0.04
YIL171W	HXT12	822	766	1.07	0.10	-0.01	-0.04
YPL159C		4949	4614	1.07	0.10	-0.01	-0.04
YNL098C	RAS2	23424	21844	1.07	0.10	-0.01	-0.04
YLL027W	ISA1	13675	12756	1.07	0.10	-0.01	-0.04
YNR027W		3452	3220	1.07	0.10	-0.01	-0.04
YMR213W	CEF1	1742	1625	1.07	0.10	-0.01	-0.05
YGR225W	SPO70	442	412	1.07	0.10	-0.01	-0.05
YNL292W	PUS4	6282	5862	1.07	0.10	-0.01	-0.05
YLR280C		2344	2188	1.07	0.10	-0.01	-0.05
YMR306W	FKS3	1199	1119	1.07	0.10	-0.01	-0.05
YGL183C		381	356	1.07	0.10	-0.01	-0.05
YGR005C	TFG2	20962	19566	1.07	0.10	-0.01	-0.05
YBR119W	MUD1	6932	6471	1.07	0.10	-0.01	-0.05
YER031C	YPT31	7359	6870	1.07	0.10	-0.01	-0.05
YDR065W		3266	3049	1.07	0.10	-0.01	-0.05
YPL117C	IDI1	20838	19457	1.07	0.10	-0.01	-0.05
YGR167W	CLC1	8603	8034	1.07	0.10	-0.01	-0.05

YLR217W		14055	13127	1.07	0.10	-0.01	-0.05
YAR030C		1565	1462	1.07	0.10	-0.01	-0.05
YEL066W	HPA3	4171	3896	1.07	0.10	-0.01	-0.05
YMR210W		1248	1166	1.07	0.10	-0.01	-0.05
YML108W		11594	10834	1.07	0.10	-0.01	-0.05
YGL133W		2269	2121	1.07	0.10	-0.01	-0.05
YOR127W	RGA1	2884	2696	1.07	0.10	-0.01	-0.05
YMR024W	MRPL3	10883	10173	1.07	0.10	-0.02	-0.05
YJL081C	ARP4	4093	3826	1.07	0.10	-0.02	-0.05
YJR114W		7716	7213	1.07	0.10	-0.02	-0.06
YGL056C	SDS23	7444	6959	1.07	0.10	-0.02	-0.06
YDR265W	PEX10	2671	2498	1.07	0.10	-0.02	-0.06
YLR253W		6468	6049	1.07	0.10	-0.02	-0.06
YNL095C		1619	1514	1.07	0.10	-0.02	-0.06
YNL280C	ERG24	8224	7695	1.07	0.10	-0.02	-0.06
YIL038C	NOT3	9143	8555	1.07	0.10	-0.02	-0.06
YDL015C		24894	23295	1.07	0.10	-0.02	-0.06
YLR317W		2533	2371	1.07	0.10	-0.02	-0.06
YMR154C	RIM13	2450	2293	1.07	0.10	-0.02	-0.06
YMR286W	MRPL33	7885	7382	1.07	0.10	-0.02	-0.06
YML070W	DAK1	5427	5081	1.07	0.10	-0.02	-0.06
YDL050C		24798	23220	1.07	0.09	-0.02	-0.06
YDL038C		2693	2522	1.07	0.09	-0.02	-0.06
YBR255W		10172	9526	1.07	0.09	-0.02	-0.06
YKL090W		3839	3595	1.07	0.09	-0.02	-0.06
YGL173C	KEM1	5245	4913	1.07	0.09	-0.02	-0.07
YIL069C	RPS24B	22230	20822	1.07	0.09	-0.02	-0.07
YKL016C	ATP7	19504	18269	1.07	0.09	-0.02	-0.07
YNR066C		3713	3478	1.07	0.09	-0.02	-0.07
YFL041W	FET5	7314	6851	1.07	0.09	-0.02	-0.07
YJL092W	HPR5	3605	3377	1.07	0.09	-0.02	-0.07
YNL246W		1933	1811	1.07	0.09	-0.02	-0.07
YGL020C		12045	11285	1.07	0.09	-0.02	-0.07
YLR424W		5306	4971	1.07	0.09	-0.02	-0.07
YDR390C	UBA2	7859	7364	1.07	0.09	-0.02	-0.07
YPL038W	MET31	7784	7294	1.07	0.09	-0.02	-0.07

YHR128W	FUR1	627	588	1.07	0.09	-0.02	-0.07
YBR290W	BSD2	6558	6146	1.07	0.09	-0.02	-0.07
YDR376W	ARH1	6611	6196	1.07	0.09	-0.02	-0.07
YLR194C		20276	19004	1.07	0.09	-0.02	-0.07
YNL125C	ESBP6	6094	5712	1.07	0.09	-0.02	-0.07
YNL152W		11010	10320	1.07	0.09	-0.02	-0.07
YIR018W	YAP5	7431	6966	1.07	0.09	-0.02	-0.07
YBR168W		1746	1637	1.07	0.09	-0.02	-0.07
YKL150W	MCR1	6871	6444	1.07	0.09	-0.02	-0.07
YKL059C		3120	2926	1.07	0.09	-0.02	-0.07
YDL214C		455	427	1.07	0.09	-0.02	-0.07
YLR398C	SKI2	4010	3761	1.07	0.09	-0.02	-0.07
YLL055W		2656	2492	1.07	0.09	-0.02	-0.07
YDL229W	SSB1	2854	2678	1.07	0.09	-0.02	-0.07
YMR025W		4301	4035	1.07	0.09	-0.02	-0.07
YCR002C	CDC10	12571	11795	1.07	0.09	-0.02	-0.07
YOR380W		1620	1520	1.07	0.09	-0.02	-0.07
YOR156C	NFI1	1441	1352	1.07	0.09	-0.02	-0.07
YGL071W	RCS1	2577	2418	1.07	0.09	-0.02	-0.07
YOR252W		15080	14153	1.07	0.09	-0.02	-0.08
YMR294W	JNM1	10094	9474	1.07	0.09	-0.02	-0.08
YDL069C	CBS1	1680	1577	1.07	0.09	-0.02	-0.08
YJL008C	CCT8	22381	21013	1.07	0.09	-0.02	-0.08
YNL339C	YRF1-6	1008	946	1.07	0.09	-0.02	-0.08
YOR330C	MIP1	3330	3128	1.06	0.09	-0.02	-0.08
YDL246C		399	375	1.06	0.09	-0.02	-0.08
YPL131W	RPL5	46865	44031	1.06	0.09	-0.02	-0.08
YKR069W	MET1	1389	1305	1.06	0.09	-0.02	-0.08
YER118C	SSU81	11690	10984	1.06	0.09	-0.02	-0.08
YGL242C		9445	8875	1.06	0.09	-0.02	-0.08
YLR349W		5280	4961	1.06	0.09	-0.02	-0.08
YHR147C	MRPL6	4328	4067	1.06	0.09	-0.02	-0.08
YOR121C		3080	2895	1.06	0.09	-0.02	-0.08
YDR303C		1300	1222	1.06	0.09	-0.02	-0.08
YDL009C		4489	4220	1.06	0.09	-0.02	-0.08
YBR242W		9847	9256	1.06	0.09	-0.02	-0.08

YDL082W	RPL13A	16894	15881	1.06	0.09	-0.02	-0.08
YLR054C		652	613	1.06	0.09	-0.02	-0.08
YJR141W		7136	6709	1.06	0.09	-0.02	-0.08
YER056C-A	RPL34A	29948	28158	1.06	0.09	-0.02	-0.09
YNL177C		8221	7731	1.06	0.09	-0.02	-0.09
YOR295W		5124	4819	1.06	0.09	-0.02	-0.09
YLR178C	TFS1	9547	8980	1.06	0.09	-0.02	-0.09
YPL043W	NOP4	4581	4309	1.06	0.09	-0.02	-0.09
YKR047W		8932	8402	1.06	0.09	-0.02	-0.09
YBL014C	RRN6	5746	5407	1.06	0.09	-0.02	-0.09
YPL099C		3032	2854	1.06	0.09	-0.03	-0.09
YDR249C		372	350	1.06	0.09	-0.03	-0.09
YKR030W		1263	1189	1.06	0.09	-0.03	-0.09
YGR147C	NAT2	5810	5469	1.06	0.09	-0.03	-0.09
YLR248W	RCK2	11776	11085	1.06	0.09	-0.03	-0.09
YLR183C		15807	14882	1.06	0.09	-0.03	-0.09
YML021C	UNG1	4287	4037	1.06	0.09	-0.03	-0.09
YFR002W	NIC96	8901	8382	1.06	0.09	-0.03	-0.09
YDL224C	WHI4	5542	5219	1.06	0.09	-0.03	-0.09
YDR438W		2465	2322	1.06	0.09	-0.03	-0.10
YCL010C		2085	1964	1.06	0.09	-0.03	-0.10
YMR086C-A		1496	1409	1.06	0.09	-0.03	-0.10
YAR031W		2141	2017	1.06	0.09	-0.03	-0.10
YPL165C		935	881	1.06	0.09	-0.03	-0.10
YLL048C	YBT1	42405	39954	1.06	0.09	-0.03	-0.10
YMR321C		6474	6101	1.06	0.09	-0.03	-0.10
YDR070C		2313	2180	1.06	0.09	-0.03	-0.10
YBR156C	SLI15	25006	23572	1.06	0.09	-0.03	-0.10
YFR039C		3940	3714	1.06	0.09	-0.03	-0.10
YBR042C		2735	2579	1.06	0.08	-0.03	-0.10
YKR033C		1363	1285	1.06	0.08	-0.03	-0.10
YEL006W		833	785	1.06	0.08	-0.03	-0.10
YHR084W	STE12	2956	2787	1.06	0.08	-0.03	-0.10
YPL042C	SSN3	8790	8289	1.06	0.08	-0.03	-0.10
YOL084W		742	700	1.06	0.08	-0.03	-0.10
YCR029C		898	847	1.06	0.08	-0.03	-0.10

YBL055C		4224	3984	1.06	0.08	-0.03	-0.10
YPL122C	TFB2	11516	10862	1.06	0.08	-0.03	-0.10
YHR004C	NEM1	6339	5979	1.06	0.08	-0.03	-0.10
YPL090C	RPS6A	33815	31895	1.06	0.08	-0.03	-0.10
YNL028W		1506	1421	1.06	0.08	-0.03	-0.10
YPR082C	DIB1	12130	11442	1.06	0.08	-0.03	-0.10
YBR116C		406	383	1.06	0.08	-0.03	-0.10
YBL113C		3385	3193	1.06	0.08	-0.03	-0.10
YER173W	RAD24	7020	6623	1.06	0.08	-0.03	-0.10
YMR247C		2189	2065	1.06	0.08	-0.03	-0.10
YDR264C	AKR1	5769	5444	1.06	0.08	-0.03	-0.10
YJR116W		1856	1752	1.06	0.08	-0.03	-0.10
YGL052W		1163	1098	1.06	0.08	-0.03	-0.11
YOR388C	FDH1	828	782	1.06	0.08	-0.03	-0.11
YML069W	POB3	9211	8695	1.06	0.08	-0.03	-0.11
YOL043C	NTG2	5051	4769	1.06	0.08	-0.03	-0.11
YOR347C	PYK2	4246	4009	1.06	0.08	-0.03	-0.11
YCLX12W		9788	9242	1.06	0.08	-0.03	-0.11
YBR192W	RIM2	2649	2502	1.06	0.08	-0.03	-0.11
YDL149W	AUT9	2990	2824	1.06	0.08	-0.03	-0.11
YML003W		1638	1547	1.06	0.08	-0.03	-0.11
YPL118W	MRP51	20817	19665	1.06	0.08	-0.03	-0.11
YOL057W		14004	13230	1.06	0.08	-0.03	-0.11
YOR091W		14655	13846	1.06	0.08	-0.03	-0.11
YCR022C		3891	3676	1.06	0.08	-0.03	-0.11
YDR482C		876	828	1.06	0.08	-0.03	-0.11
YJL216C		1560	1474	1.06	0.08	-0.03	-0.11
YOL072W		3625	3426	1.06	0.08	-0.03	-0.11
YNL092W		1686	1593	1.06	0.08	-0.03	-0.11
YER159C	BUR6	7602	7185	1.06	0.08	-0.03	-0.11
YPL272C		2064	1951	1.06	0.08	-0.03	-0.11
YEL047C		5776	5459	1.06	0.08	-0.03	-0.11
YDR125C	ECM18	908	858	1.06	0.08	-0.03	-0.11
YAR053W		1352	1278	1.06	0.08	-0.03	-0.11
YNL096C	RPS7B	27211	25724	1.06	0.08	-0.03	-0.11
YLR051C		25129	23757	1.06	0.08	-0.03	-0.11

YOR275C		3623	3425	1.06	0.08	-0.03	-0.11
YHR040W		3612	3415	1.06	0.08	-0.03	-0.11
YOR119C	RIO1	4420	4179	1.06	0.08	-0.03	-0.11
YNL097C	PHO23	3344	3162	1.06	0.08	-0.03	-0.12
YAR037W		2134	2018	1.06	0.08	-0.03	-0.12
YJL041W	NSP1	686	649	1.06	0.08	-0.03	-0.12
YKL069W		6159	5827	1.06	0.08	-0.03	-0.12
YML051W	GAL80	6018	5694	1.06	0.08	-0.03	-0.12
YLL012W		4386	4150	1.06	0.08	-0.03	-0.12
YDR346C		5006	4738	1.06	0.08	-0.03	-0.12
YLR326W		3489	3302	1.06	0.08	-0.03	-0.12
YPR060C	ARO7	26210	24808	1.06	0.08	-0.03	-0.12
YPR102C	RPL11A	36979	35002	1.06	0.08	-0.03	-0.12
YJR151C		4335	4104	1.06	0.08	-0.03	-0.12
YLR197W	SIK1	8499	8047	1.06	0.08	-0.03	-0.12
YJL063C	MRPL8	8841	8371	1.06	0.08	-0.03	-0.12
YGR274C	TAF145	9185	8698	1.06	0.08	-0.03	-0.12
YKL176C		2589	2452	1.06	0.08	-0.03	-0.12
YOR073W		2633	2494	1.06	0.08	-0.03	-0.12
YMR269W		9352	8859	1.06	0.08	-0.03	-0.12
YCL042W		3014	2856	1.06	0.08	-0.03	-0.13
YLR071C	RGR1	1833	1737	1.06	0.08	-0.03	-0.13
YGR148C	RPL24B	36902	34966	1.06	0.08	-0.03	-0.13
YHR105W		4551	4313	1.06	0.08	-0.03	-0.13
YDR396W		9465	8971	1.06	0.08	-0.03	-0.13
YCR034W	FEN1	20991	19895	1.06	0.08	-0.04	-0.13
YJL132W		4709	4464	1.05	0.08	-0.04	-0.13
YKL113C	RAD27	11047	10472	1.05	0.08	-0.04	-0.13
YLR007W		4591	4352	1.05	0.08	-0.04	-0.13
YGR150C		2094	1986	1.05	0.08	-0.04	-0.13
YGL174W		5375	5098	1.05	0.08	-0.04	-0.13
YLR212C	TUB4	10833	10275	1.05	0.08	-0.04	-0.13
YAL022C	FUN26	10786	10233	1.05	0.08	-0.04	-0.13
YLR164W		980	930	1.05	0.08	-0.04	-0.13
YPL023C	MET12	10876	10320	1.05	0.08	-0.04	-0.13
YEL003W	GIM4	20720	19661	1.05	0.08	-0.04	-0.13

YGR030C	POP6	3239	3073	1.05	0.08	-0.04	-0.13
YBL016W	FUS3	12087	11473	1.05	0.08	-0.04	-0.13
YGL097W	SRM1	14495	13758	1.05	0.08	-0.04	-0.13
YGR214W	RPS0A	30899	29330	1.05	0.08	-0.04	-0.14
YPL109C		753	715	1.05	0.08	-0.04	-0.14
YDL036C		5098	4839	1.05	0.08	-0.04	-0.14
YLR010C		3609	3426	1.05	0.08	-0.04	-0.14
YMR233W		2984	2833	1.05	0.08	-0.04	-0.14
YCL058C		6429	6104	1.05	0.07	-0.04	-0.14
YGL074C		785	745	1.05	0.07	-0.04	-0.14
YBR191W	RPL21A	40398	38355	1.05	0.07	-0.04	-0.14
YIR027C	DAL1	1083	1028	1.05	0.07	-0.04	-0.14
YGR187C	HGH1	10480	9951	1.05	0.07	-0.04	-0.14
YGR072W	UPF3	6193	5881	1.05	0.07	-0.04	-0.14
YDL247W		604	574	1.05	0.07	-0.04	-0.14
YER001W	MNN1	5675	5391	1.05	0.07	-0.04	-0.14
YAL034W-A	MTW1	1839	1747	1.05	0.07	-0.04	-0.14
YKL101W	HSL1	1636	1555	1.05	0.07	-0.04	-0.14
YJR143C	PMT4	13018	12372	1.05	0.07	-0.04	-0.14
YCR043C		13871	13183	1.05	0.07	-0.04	-0.14
YGR133W	PEX4	1416	1346	1.05	0.07	-0.04	-0.14
YGR053C		1822	1732	1.05	0.07	-0.04	-0.14
YLR149C		1785	1697	1.05	0.07	-0.04	-0.14
YER066W		1977	1879	1.05	0.07	-0.04	-0.14
YHR143W		2551	2425	1.05	0.07	-0.04	-0.14
YMR165C	SMP2	826	785	1.05	0.07	-0.04	-0.14
YLR298C	YHC1	5661	5383	1.05	0.07	-0.04	-0.14
YJR146W		4470	4251	1.05	0.07	-0.04	-0.14
YBR278W	DPB3	3275	3115	1.05	0.07	-0.04	-0.15
YPR004C		18912	17986	1.05	0.07	-0.04	-0.15
YKL042W	SPC42	4098	3898	1.05	0.07	-0.04	-0.15
YFR032C		924	879	1.05	0.07	-0.04	-0.15
YMR174C	PAI3	1370	1303	1.05	0.07	-0.04	-0.15
YOR386W	PHR1	1292	1229	1.05	0.07	-0.04	-0.15
YOR342C		21352	20313	1.05	0.07	-0.04	-0.15
YKR043C		20815	19803	1.05	0.07	-0.04	-0.15

YBL029W		1591	1514	1.05	0.07	-0.04	-0.15
YGL045W		2636	2508	1.05	0.07	-0.04	-0.15
YLR245C	CDD1	2571	2447	1.05	0.07	-0.04	-0.15
YCLX11W		2261	2152	1.05	0.07	-0.04	-0.15
YCR098C	GIT1	1498	1426	1.05	0.07	-0.04	-0.15
YGL029W	CGR1	18477	17586	1.05	0.07	-0.04	-0.15
YPL182C		5623	5352	1.05	0.07	-0.04	-0.15
YDR354W	TRP4	11363	10817	1.05	0.07	-0.04	-0.15
YAR002W		3438	3273	1.05	0.07	-0.04	-0.15
YNL161W	CBK1	4906	4671	1.05	0.07	-0.04	-0.15
YDR383C		5926	5642	1.05	0.07	-0.04	-0.15
YHR131C		3312	3154	1.05	0.07	-0.04	-0.15
YMR308C	PSE1	18477	17594	1.05	0.07	-0.04	-0.15
YKL070W		3434	3270	1.05	0.07	-0.04	-0.15
YKL062W	MSN4	5187	4939	1.05	0.07	-0.04	-0.15
YHR144C	DCD1	20470	19494	1.05	0.07	-0.04	-0.15
YOL090W	MSH2	8671	8258	1.05	0.07	-0.04	-0.15
YPR187W	RPO26	16618	15827	1.05	0.07	-0.04	-0.15
YDR448W	ADA2	3694	3518	1.05	0.07	-0.04	-0.15
YOL060C	AMI3	7074	6738	1.05	0.07	-0.04	-0.15
YGR195W	SKI6	6096	5806	1.05	0.07	-0.04	-0.15
YNL035C		5614	5348	1.05	0.07	-0.04	-0.15
YCR060W		3497	3331	1.05	0.07	-0.04	-0.15
YGL243W	TAD1	5636	5369	1.05	0.07	-0.04	-0.15
YLL067C		12548	11953	1.05	0.07	-0.04	-0.15
YEL033W		11018	10496	1.05	0.07	-0.04	-0.15
YOR278W	HEM4	8358	7962	1.05	0.07	-0.04	-0.15
YIL020C	HIS6	14884	14179	1.05	0.07	-0.04	-0.15
YGL185C		5322	5070	1.05	0.07	-0.04	-0.15
YGR244C	LSC2	809	771	1.05	0.07	-0.04	-0.15
YPL236C		4643	4423	1.05	0.07	-0.04	-0.15
YBR060C	ORC2	2405	2291	1.05	0.07	-0.04	-0.15
YDR150W	NUM1	1948	1856	1.05	0.07	-0.04	-0.15
YDR111C		1821	1735	1.05	0.07	-0.04	-0.16
YNL311C		8660	8253	1.05	0.07	-0.04	-0.16
YNL117W	MLS1	1210	1153	1.05	0.07	-0.04	-0.16

YER003C	PMI40	6235	5943	1.05	0.07	-0.04	-0.16
YBR170C	NPL4	3519	3354	1.05	0.07	-0.04	-0.16
YOR039W	CKB2	17422	16606	1.05	0.07	-0.04	-0.16
YHR188C		1705	1625	1.05	0.07	-0.04	-0.16
YLR361C		1587	1513	1.05	0.07	-0.04	-0.16
YJR113C		3210	3060	1.05	0.07	-0.04	-0.16
YPL260W		14316	13649	1.05	0.07	-0.04	-0.16
YHL024W	NOS1	1919	1830	1.05	0.07	-0.04	-0.16
YMR027W	HRT2	10727	10229	1.05	0.07	-0.04	-0.16
YER156C		17967	17133	1.05	0.07	-0.04	-0.16
YIL017W		1922	1833	1.05	0.07	-0.04	-0.16
YBR275C	RIF1	1265	1206	1.05	0.07	-0.04	-0.16
YNL314W	DAL82	3126	2982	1.05	0.07	-0.04	-0.16
YJL055W		10081	9615	1.05	0.07	-0.04	-0.16
YNL222W	SSU72	4759	4539	1.05	0.07	-0.04	-0.16
YJR058C	APS2	7036	6712	1.05	0.07	-0.04	-0.16
YJR117W	STE24	3200	3053	1.05	0.07	-0.04	-0.16
YJL058C		3126	2983	1.05	0.07	-0.04	-0.16
YFL022C	FRS2	15016	14329	1.05	0.07	-0.04	-0.16
YIL067C		1899	1812	1.05	0.07	-0.04	-0.16
YDR330W		5200	4963	1.05	0.07	-0.05	-0.16
YNL190W		29152	27823	1.05	0.07	-0.05	-0.16
YFL021W	GAT1	8891	8487	1.05	0.07	-0.05	-0.16
YGR286C	BIO2	6489	6195	1.05	0.07	-0.05	-0.17
YDL065C	PEX19	5550	5299	1.05	0.07	-0.05	-0.17
YDR248C		390	372	1.05	0.07	-0.05	-0.17
YGL214W		2212	2112	1.05	0.07	-0.05	-0.17
YJR077C	MIR1	2175	2077	1.05	0.07	-0.05	-0.17
YPL178W	MUD13	886	846	1.05	0.07	-0.05	-0.17
YGR111W		3453	3298	1.05	0.07	-0.05	-0.17
YNL039W	TFC5	4392	4195	1.05	0.07	-0.05	-0.17
YIL078W	THS1	36485	34847	1.05	0.07	-0.05	-0.17
YPR048W	TAH18	9109	8700	1.05	0.07	-0.05	-0.17
YPL248C	GAL4	2344	2240	1.05	0.07	-0.05	-0.17
YMR207C	HFA1	822	785	1.05	0.07	-0.05	-0.17
YBR037C	SCO1	2617	2500	1.05	0.07	-0.05	-0.17

YNL291C	MID1	11680	11160	1.05	0.07	-0.05	-0.17
YDL111C	RRP42	9858	9419	1.05	0.07	-0.05	-0.17
YGR015C		6280	6001	1.05	0.07	-0.05	-0.17
YBL005W-A		46044	43998	1.05	0.07	-0.05	-0.17
YDL204W		2707	2587	1.05	0.07	-0.05	-0.17
YML018C		16253	15534	1.05	0.07	-0.05	-0.17
YKR093W	PTR2	11143	10650	1.05	0.07	-0.05	-0.17
YDR087C	RRP1	10115	9670	1.05	0.06	-0.05	-0.17
YLR030W		1403	1341	1.05	0.06	-0.05	-0.17
YGL231C		22694	21698	1.05	0.06	-0.05	-0.17
YIL166C		1362	1302	1.05	0.06	-0.05	-0.17
YNL148C	ALF1	3911	3740	1.05	0.06	-0.05	-0.17
YDR368W	YPR1	1713	1638	1.05	0.06	-0.05	-0.17
YNR044W	AGA1	823	787	1.05	0.06	-0.05	-0.17
YLR461W	PAU4	912	872	1.05	0.06	-0.05	-0.17
YJR125C	ENT3	8015	7665	1.05	0.06	-0.05	-0.17
YHR008C	SOD2	4319	4130	1.05	0.06	-0.05	-0.17
YIR029W	DAL2	1441	1378	1.05	0.06	-0.05	-0.17
YJL038C		1303	1246	1.05	0.06	-0.05	-0.17
YBR123C	TFC1	6078	5813	1.05	0.06	-0.05	-0.17
YJL056C	ZAP1	4298	4111	1.05	0.06	-0.05	-0.18
YOR180C	DCI1	3680	3520	1.05	0.06	-0.05	-0.18
YOL054W		4719	4514	1.05	0.06	-0.05	-0.18
YBL093C	ROX3	13690	13096	1.05	0.06	-0.05	-0.18
YLR146C	SPE4	5028	4810	1.05	0.06	-0.05	-0.18
YDR329C	PEX3	2712	2595	1.05	0.06	-0.05	-0.18
YOR036W	PEP12	3457	3308	1.05	0.06	-0.05	-0.18
YOR132W	VPS17	3345	3201	1.05	0.06	-0.05	-0.18
YCLX07W		763	730	1.04	0.06	-0.05	-0.18
YOR183W		988	946	1.04	0.06	-0.05	-0.18
YPL223C	GRE1	1329	1272	1.04	0.06	-0.05	-0.18
YLR355C	ILV5	25047	23981	1.04	0.06	-0.05	-0.18
YLR360W	VPS38	4079	3906	1.04	0.06	-0.05	-0.18
YMR208W	ERG12	5787	5541	1.04	0.06	-0.05	-0.18
YOR087W		5768	5523	1.04	0.06	-0.05	-0.18
YGL033W	HOP2	1065	1020	1.04	0.06	-0.05	-0.18

YPR131C	NAT3	4573	4380	1.04	0.06	-0.05	-0.18
YDR304C	CYP5	6189	5928	1.04	0.06	-0.05	-0.18
YLR283W		4191	4015	1.04	0.06	-0.05	-0.18
YMR091C	NPL6	12395	11874	1.04	0.06	-0.05	-0.18
YBL032W		12999	12453	1.04	0.06	-0.05	-0.18
YML035C	AMD1	15157	14520	1.04	0.06	-0.05	-0.18
YEL020C		5470	5240	1.04	0.06	-0.05	-0.18
YNR055C	HOL1	4961	4754	1.04	0.06	-0.05	-0.19
YER148W	SPT15	23912	22916	1.04	0.06	-0.05	-0.19
YKL183W		2354	2256	1.04	0.06	-0.05	-0.19
YLL058W		2794	2678	1.04	0.06	-0.05	-0.19
YGR066C		902	865	1.04	0.06	-0.05	-0.19
YGR149W		2120	2033	1.04	0.06	-0.05	-0.19
YPL168W		6189	5934	1.04	0.06	-0.05	-0.19
YML114C		5388	5166	1.04	0.06	-0.05	-0.19
YMR155W		4124	3955	1.04	0.06	-0.05	-0.19
YDR244W	PEX5	510	489	1.04	0.06	-0.05	-0.19
YOR316C	COT1	5345	5126	1.04	0.06	-0.05	-0.19
YNR039C		3230	3098	1.04	0.06	-0.05	-0.19
YHR160C	PEX18	7267	6970	1.04	0.06	-0.05	-0.19
YNL269W		1239	1188	1.04	0.06	-0.05	-0.19
YML085C	TUB1	4500	4317	1.04	0.06	-0.05	-0.19
YKL114C	APN1	5307	5091	1.04	0.06	-0.05	-0.19
YMR102C		3463	3322	1.04	0.06	-0.05	-0.19
YPL184C		8137	7806	1.04	0.06	-0.05	-0.19
YLR404W		4809	4614	1.04	0.06	-0.05	-0.19
YPR201W	ARR3	2593	2488	1.04	0.06	-0.05	-0.19
YEL072W		3500	3358	1.04	0.06	-0.05	-0.19
YOR023C	AHC1	6424	6164	1.04	0.06	-0.05	-0.19
YJR162C		1770	1699	1.04	0.06	-0.05	-0.19
YPR204W		13770	13218	1.04	0.06	-0.05	-0.19
YLR417W	VPS36	2801	2689	1.04	0.06	-0.05	-0.19
YGL152C		2398	2302	1.04	0.06	-0.05	-0.19
YDL166C		10011	9611	1.04	0.06	-0.05	-0.19
YOR221C	MCT1	678	651	1.04	0.06	-0.05	-0.20
YNL319W		1867	1793	1.04	0.06	-0.05	-0.20

YNR049C	MSO1	4421	4245	1.04	0.06	-0.05	-0.20
YIL116W	HIS5	15050	14452	1.04	0.06	-0.05	-0.20
YHR051W	COX6	6931	6656	1.04	0.06	-0.05	-0.20
YDL040C	NAT1	5580	5359	1.04	0.06	-0.05	-0.20
YCL041C		3709	3562	1.04	0.06	-0.05	-0.20
YLR139C	SLS1	3005	2886	1.04	0.06	-0.05	-0.20
YGL177W		995	956	1.04	0.06	-0.05	-0.20
YDL054C		8895	8545	1.04	0.06	-0.05	-0.20
YJR155W	AAD10	1929	1853	1.04	0.06	-0.05	-0.20
YJL163C		5415	5202	1.04	0.06	-0.05	-0.20
YLR029C	RPL15A	46273	44458	1.04	0.06	-0.05	-0.20
YIL021W	RPB3	6596	6338	1.04	0.06	-0.05	-0.20
YDR280W	RRP45	21630	20785	1.04	0.06	-0.05	-0.20
YOL123W	HRP1	5125	4925	1.04	0.06	-0.05	-0.20
YIL065C		2048	1968	1.04	0.06	-0.06	-0.20
YOR267C		3620	3479	1.04	0.06	-0.06	-0.20
YCL025C	AGP1	7141	6864	1.04	0.06	-0.06	-0.20
YIL075C	RPN2	12071	11603	1.04	0.06	-0.06	-0.20
YOR010C	TIR2	1092	1050	1.04	0.06	-0.06	-0.20
YEL028W		5047	4852	1.04	0.06	-0.06	-0.20
YPL205C		1814	1744	1.04	0.06	-0.06	-0.20
YJR085C		13583	13059	1.04	0.06	-0.06	-0.20
YBR283C	SSH1	42599	40956	1.04	0.06	-0.06	-0.20
YML015C	TAF40	3383	3253	1.04	0.06	-0.06	-0.20
YLR449W	FPR4	12612	12127	1.04	0.06	-0.06	-0.20
YKL081W	TEF4	7847	7545	1.04	0.06	-0.06	-0.20
YGR240C	PFK1	31832	30608	1.04	0.06	-0.06	-0.20
YPL254W	HFI1	3290	3164	1.04	0.06	-0.06	-0.20
YJR063W	RPA12	9742	9369	1.04	0.06	-0.06	-0.20
YLR436C	ECM30	8880	8541	1.04	0.06	-0.06	-0.20
YKL139W	CTK1	1698	1633	1.04	0.06	-0.06	-0.20
YHR029C		9430	9070	1.04	0.06	-0.06	-0.20
YGR262C		7437	7153	1.04	0.06	-0.06	-0.20
YHR007C	ERG11	46934	45145	1.04	0.06	-0.06	-0.20
YPR153W		2786	2680	1.04	0.06	-0.06	-0.21
YPL262W	FUM1	10427	10031	1.04	0.06	-0.06	-0.21

YJR088C		6399	6157	1.04	0.06	-0.06	-0.21
YAL066W		785	755	1.04	0.06	-0.06	-0.21
YCL021W		2912	2802	1.04	0.06	-0.06	-0.21
YIL051C	MMD1	11245	10822	1.04	0.06	-0.06	-0.21
YNL295W		2399	2309	1.04	0.06	-0.06	-0.21
YER026C	CHO1	25363	24410	1.04	0.06	-0.06	-0.21
YDR001C	NTH1	749	721	1.04	0.06	-0.06	-0.21
YOR113W	AZF1	2056	1979	1.04	0.06	-0.06	-0.21
YMR016C	SOK2	6874	6616	1.04	0.06	-0.06	-0.21
YFR048W		6219	5986	1.04	0.06	-0.06	-0.21
YLR440C		6391	6153	1.04	0.05	-0.06	-0.21
YPL270W	MDL2	4298	4138	1.04	0.05	-0.06	-0.21
YJL011C		5050	4862	1.04	0.05	-0.06	-0.21
YEL067C		3835	3692	1.04	0.05	-0.06	-0.21
YCR023C		4967	4783	1.04	0.05	-0.06	-0.21
YLR097C		11439	11021	1.04	0.05	-0.06	-0.21
YLR284C	ECI1	3073	2961	1.04	0.05	-0.06	-0.21
YDR132C		6930	6678	1.04	0.05	-0.06	-0.21
YJL102W	MEF2	4998	4817	1.04	0.05	-0.06	-0.22
YKL208W	CBT1	2373	2288	1.04	0.05	-0.06	-0.22
YMR115W		5120	4936	1.04	0.05	-0.06	-0.22
YDL104C	QRI7	4902	4727	1.04	0.05	-0.06	-0.22
YLR249W	YEF3	34809	33569	1.04	0.05	-0.06	-0.22
YBR176W	ECM31	1841	1776	1.04	0.05	-0.06	-0.22
YDR311W	TFB1	399	385	1.04	0.05	-0.06	-0.22
YJL218W		1776	1713	1.04	0.05	-0.06	-0.22
YDL089W		811	782	1.04	0.05	-0.06	-0.22
YDR490C	PKH1	1003	968	1.04	0.05	-0.06	-0.22
YCR052W	RSC6	5295	5108	1.04	0.05	-0.06	-0.22
YMR181C		6346	6123	1.04	0.05	-0.06	-0.22
YJL223C	PAU1	990	955	1.04	0.05	-0.06	-0.22
YIR034C	LYS1	16708	16123	1.04	0.05	-0.06	-0.22
YJR069C	HAM1	4779	4612	1.04	0.05	-0.06	-0.22
YCR068W		1211	1169	1.04	0.05	-0.06	-0.22
YLR078C	BOS1	6533	6306	1.04	0.05	-0.06	-0.22
YOR044W		1630	1573	1.04	0.05	-0.06	-0.22

YKL023W		1467	1416	1.04	0.05	-0.06	-0.22
YJL119C		975	941	1.04	0.05	-0.06	-0.22
YDR404C	RPB7	9087	8773	1.04	0.05	-0.06	-0.22
YBL026W	LSM2	13277	12819	1.04	0.05	-0.06	-0.22
YGL018C	JAC1	1568	1514	1.04	0.05	-0.06	-0.22
YJR086W	STE18	5304	5122	1.04	0.05	-0.06	-0.23
YMR246W	FAA4	9131	8817	1.04	0.05	-0.06	-0.23
YOL048C		3489	3369	1.04	0.05	-0.06	-0.23
YDR392W	SPT3	5728	5532	1.04	0.05	-0.06	-0.23
YKL064W	MNR2	6086	5878	1.04	0.05	-0.06	-0.23
YDR029W		597	577	1.04	0.05	-0.06	-0.23
YDL115C		2448	2365	1.04	0.05	-0.06	-0.23
YLR229C	CDC42	29951	28935	1.04	0.05	-0.06	-0.23
YGL233W	SEC15	10457	10102	1.04	0.05	-0.06	-0.23
YMR045C		7047	6809	1.03	0.05	-0.06	-0.23
YDL177C		7322	7075	1.03	0.05	-0.06	-0.23
YOL107W		4276	4132	1.03	0.05	-0.06	-0.23
YKL026C		2185	2111	1.03	0.05	-0.06	-0.23
YEL049W	PAU2	1260	1218	1.03	0.05	-0.06	-0.23
YER045C		2098	2028	1.03	0.05	-0.06	-0.23
YLR402W		800	773	1.03	0.05	-0.06	-0.23
YBR212W	NGR1	4830	4669	1.03	0.05	-0.06	-0.23
YER066C-A		1475	1426	1.03	0.05	-0.06	-0.23
YKL154W	SRP102	8688	8401	1.03	0.05	-0.06	-0.23
YGL253W	HXK2	37435	36203	1.03	0.05	-0.06	-0.23
YLL010C		3424	3312	1.03	0.05	-0.06	-0.23
YDR161W	TCI1	39162	37878	1.03	0.05	-0.06	-0.23
YOL086C	ADH1	33737	32632	1.03	0.05	-0.06	-0.23
YDR175C		22236	21509	1.03	0.05	-0.06	-0.23
YLR268W	SEC22	15855	15338	1.03	0.05	-0.06	-0.23
YLR467W	YRF1-5	5821	5633	1.03	0.05	-0.07	-0.24
YLR250W	SSP120	15884	15372	1.03	0.05	-0.07	-0.24
YBL021C	HAP3	3182	3080	1.03	0.05	-0.07	-0.24
YBL011W	SCT1	4865	4709	1.03	0.05	-0.07	-0.24
YER116C		5435	5261	1.03	0.05	-0.07	-0.24
YDR045C	RPC11	3100	3001	1.03	0.05	-0.07	-0.24

YMR188C		14436	13975	1.03	0.05	-0.07	-0.24
YPR050C		7531	7292	1.03	0.05	-0.07	-0.24
YJR107W		1608	1557	1.03	0.05	-0.07	-0.24
YMR203W	TOM40	5204	5041	1.03	0.05	-0.07	-0.24
YPL110C		3390	3284	1.03	0.05	-0.07	-0.24
YLR341W		1995	1932	1.03	0.05	-0.07	-0.24
YBR159W		8692	8421	1.03	0.05	-0.07	-0.24
YMR029C		11390	11036	1.03	0.05	-0.07	-0.24
YKR004C	ECM9	2494	2416	1.03	0.05	-0.07	-0.24
YOR031W	CRS5	2227	2158	1.03	0.05	-0.07	-0.24
YJL198W		7661	7424	1.03	0.05	-0.07	-0.24
YGR144W	THI4	1612	1562	1.03	0.05	-0.07	-0.24
YGL159W		4301	4168	1.03	0.05	-0.07	-0.24
YLR270W		6144	5954	1.03	0.05	-0.07	-0.24
YLR337C	VRP1	2431	2356	1.03	0.04	-0.07	-0.25
YMR107W		1378	1336	1.03	0.04	-0.07	-0.25
YFL065C		3016	2924	1.03	0.04	-0.07	-0.25
YLR377C	FBP1	474	460	1.03	0.04	-0.07	-0.25
YCL031C	RRP7	15854	15374	1.03	0.04	-0.07	-0.25
YAR019C	CDC15	420	407	1.03	0.04	-0.07	-0.25
YPR143W		30034	29131	1.03	0.04	-0.07	-0.25
YGL210W	YPT32	9263	8985	1.03	0.04	-0.07	-0.25
YGR267C	FOL2	13557	13151	1.03	0.04	-0.07	-0.25
YLL018C	DPS1	14105	13684	1.03	0.04	-0.07	-0.25
YLR179C		12420	12051	1.03	0.04	-0.07	-0.25
YGR164W		4417	4286	1.03	0.04	-0.07	-0.25
YGR044C	RME1	6015	5837	1.03	0.04	-0.07	-0.25
YDR153C		13247	12855	1.03	0.04	-0.07	-0.25
YGR235C		10272	9969	1.03	0.04	-0.07	-0.25
YLR465C		1314	1275	1.03	0.04	-0.07	-0.25
YCL046W		351	341	1.03	0.04	-0.07	-0.25
YEL036C	ANP1	13861	13453	1.03	0.04	-0.07	-0.25
YNL024C		3554	3450	1.03	0.04	-0.07	-0.25
YPR083W		8187	7947	1.03	0.04	-0.07	-0.25
YDR508C	GNP1	9835	9546	1.03	0.04	-0.07	-0.25
YOR055W		3357	3259	1.03	0.04	-0.07	-0.25

YJR087W		9125	8858	1.03	0.04	-0.07	-0.25
YML079W		7005	6800	1.03	0.04	-0.07	-0.25
YMR225C	MRPL44	3881	3768	1.03	0.04	-0.07	-0.25
YJL015C		1729	1679	1.03	0.04	-0.07	-0.25
YOR240W		7424	7209	1.03	0.04	-0.07	-0.25
YBR122C	MRPL36	9159	8895	1.03	0.04	-0.07	-0.26
YBL099W	ATP1	643	624	1.03	0.04	-0.07	-0.26
YOR320C		2331	2264	1.03	0.04	-0.07	-0.26
YDR520C		2878	2796	1.03	0.04	-0.07	-0.26
YPR128C		9021	8766	1.03	0.04	-0.07	-0.26
YGL180W	APG1	2968	2884	1.03	0.04	-0.07	-0.26
YGR162W	TIF4631	18470	17949	1.03	0.04	-0.07	-0.26
YPL213W	LEA1	5830	5667	1.03	0.04	-0.07	-0.26
YFR008W		2216	2154	1.03	0.04	-0.07	-0.26
YNL037C	IDH1	14004	13615	1.03	0.04	-0.07	-0.26
YOR093C		2484	2415	1.03	0.04	-0.07	-0.26
YKL092C	BUD2	6101	5932	1.03	0.04	-0.07	-0.26
YLR368W		1773	1724	1.03	0.04	-0.07	-0.26
YGR176W		7494	7288	1.03	0.04	-0.07	-0.26
YLL025W		3106	3021	1.03	0.04	-0.07	-0.26
YLR070C		887	863	1.03	0.04	-0.07	-0.26
YGL213C	SKI8	8961	8717	1.03	0.04	-0.07	-0.26
YJL065C		5676	5522	1.03	0.04	-0.07	-0.26
YEL030W	ECM10	9014	8770	1.03	0.04	-0.07	-0.26
YML041C		3712	3611	1.03	0.04	-0.07	-0.26
YOR118W		2466	2400	1.03	0.04	-0.07	-0.27
YOL014W		4670	4545	1.03	0.04	-0.07	-0.27
YOR203W		23266	22646	1.03	0.04	-0.07	-0.27
YER010C		2455	2390	1.03	0.04	-0.07	-0.27
YEL005C	VAB31	5846	5691	1.03	0.04	-0.07	-0.27
YKL006C-A	SFT1	5960	5803	1.03	0.04	-0.07	-0.27
YDR226W	ADK1	3465	3374	1.03	0.04	-0.07	-0.27
YNL325C	FIG4	5479	5335	1.03	0.04	-0.07	-0.27
YPR038W		7515	7317	1.03	0.04	-0.07	-0.27
YDR113C	PDS1	5867	5714	1.03	0.04	-0.07	-0.27
YDR215C		454	442	1.03	0.04	-0.07	-0.27

YDR208W	MSS4	1411	1374	1.03	0.04	-0.07	-0.27
YOR040W	GLO4	4079	3973	1.03	0.04	-0.07	-0.27
YJL148W	RPA34	45277	44104	1.03	0.04	-0.07	-0.27
YHR016C	YSC84	2535	2469	1.03	0.04	-0.07	-0.27
YKL008C	LAC1	10085	9825	1.03	0.04	-0.07	-0.27
YLR266C		6927	6749	1.03	0.04	-0.07	-0.27
YOL030W		17822	17365	1.03	0.04	-0.07	-0.27
YOR349W	CIN1	2045	1993	1.03	0.04	-0.08	-0.27
YBL086C		1271	1239	1.03	0.04	-0.08	-0.27
YOR172W		2871	2798	1.03	0.04	-0.08	-0.27
YEL015W		22097	21537	1.03	0.04	-0.08	-0.27
YBR198C	TAF90	2458	2396	1.03	0.04	-0.08	-0.27
YCL057W	PRD1	9070	8842	1.03	0.04	-0.08	-0.27
YER007C-A		9259	9027	1.03	0.04	-0.08	-0.28
YPL164C		2682	2615	1.03	0.04	-0.08	-0.28
YOR356W		2286	2229	1.03	0.04	-0.08	-0.28
YKR053C	YSR3	2700	2634	1.03	0.04	-0.08	-0.28
YOL097C	WRS1	22524	21974	1.03	0.04	-0.08	-0.28
YKR003W		3564	3477	1.02	0.04	-0.08	-0.28
YGL019W	CKB1	7885	7694	1.02	0.04	-0.08	-0.28
YDR143C	SAN1	3873	3779	1.02	0.04	-0.08	-0.28
YPL194W	DDC1	4293	4189	1.02	0.04	-0.08	-0.28
YDR338C		2209	2156	1.02	0.04	-0.08	-0.28
YLR115W	CFT2	3271	3193	1.02	0.03	-0.08	-0.28
YMR299C		13829	13498	1.02	0.03	-0.08	-0.28
YMR081C	ISF1	1222	1193	1.02	0.03	-0.08	-0.28
YFR043C		2403	2346	1.02	0.03	-0.08	-0.28
YPL166W		1532	1496	1.02	0.03	-0.08	-0.28
YPL104W	MSD1	6234	6087	1.02	0.03	-0.08	-0.28
YOR019W		861	841	1.02	0.03	-0.08	-0.28
YPL069C	BTS1	2689	2626	1.02	0.03	-0.08	-0.28
YDR177W	UBC1	20046	19580	1.02	0.03	-0.08	-0.29
YEL024W	RIP1	14883	14539	1.02	0.03	-0.08	-0.29
YDL226C	GCS1	11043	10788	1.02	0.03	-0.08	-0.29
YDR012W	RPL4B	15802	15437	1.02	0.03	-0.08	-0.29
YNL142W	MEP2	7744	7566	1.02	0.03	-0.08	-0.29

YNL210W	MER1	1926	1882	1.02	0.03	-0.08	-0.29
YLR200W	YKE2	7256	7090	1.02	0.03	-0.08	-0.29
YML032C	RAD52	11671	11404	1.02	0.03	-0.08	-0.29
YBR044C	TCM62	2119	2071	1.02	0.03	-0.08	-0.29
YPL237W	SUI3	27977	27345	1.02	0.03	-0.08	-0.29
YMR153W	NUP53	8526	8335	1.02	0.03	-0.08	-0.29
YFL-TYA		27263	26653	1.02	0.03	-0.08	-0.29
YDL201W		33877	33121	1.02	0.03	-0.08	-0.29
YJR147W	HMS2	4068	3978	1.02	0.03	-0.08	-0.29
YJL212C		7526	7359	1.02	0.03	-0.08	-0.29
YLR016C		14576	14254	1.02	0.03	-0.08	-0.29
YDR385W	EFT2	37219	36400	1.02	0.03	-0.08	-0.29
YGR010W		5472	5352	1.02	0.03	-0.08	-0.29
YDR412W		8865	8671	1.02	0.03	-0.08	-0.29
YAL039C	CYC3	4242	4149	1.02	0.03	-0.08	-0.29
YKL135C	APL2	9841	9626	1.02	0.03	-0.08	-0.29
YKL197C	PEX1	3353	3280	1.02	0.03	-0.08	-0.29
YPL186C		3117	3049	1.02	0.03	-0.08	-0.29
YDR266C		3210	3140	1.02	0.03	-0.08	-0.29
YCL027W	FUS1	4206	4115	1.02	0.03	-0.08	-0.29
YLR127C	APC2	3902	3819	1.02	0.03	-0.08	-0.30
YIL109C	SEC24	12302	12042	1.02	0.03	-0.08	-0.30
YOR370C	MRS6	1039	1017	1.02	0.03	-0.08	-0.30
YAL013W	DEP1	2682	2626	1.02	0.03	-0.08	-0.30
YLR239C		4984	4880	1.02	0.03	-0.08	-0.30
YKL098W		3794	3715	1.02	0.03	-0.08	-0.30
YKR062W	TFA2	2722	2666	1.02	0.03	-0.08	-0.30
YPL239W	YAR1	28500	27918	1.02	0.03	-0.08	-0.30
YMR074C		12122	11875	1.02	0.03	-0.08	-0.30
YLR091W		8329	8160	1.02	0.03	-0.08	-0.30
YJR149W		1409	1381	1.02	0.03	-0.08	-0.30
YBR108W		1904	1866	1.02	0.03	-0.08	-0.30
YBR076W	ECM8	1671	1637	1.02	0.03	-0.08	-0.30
YBR291C	CTP1	11185	10961	1.02	0.03	-0.08	-0.30
YPR001W	CIT3	2366	2319	1.02	0.03	-0.08	-0.30
YAR010C		62869	61612	1.02	0.03	-0.08	-0.30

YNL261W	ORC5	17560	17209	1.02	0.03	-0.08	-0.30
YFR046C		12588	12337	1.02	0.03	-0.08	-0.30
YOR123C	LEO1	10405	10198	1.02	0.03	-0.08	-0.30
YGL199C		7333	7189	1.02	0.03	-0.08	-0.30
YPR171W		1208	1185	1.02	0.03	-0.08	-0.31
YJR061W		570	559	1.02	0.03	-0.08	-0.31
YFL037W	TUB2	34128	33468	1.02	0.03	-0.08	-0.31
YLR389C	STE23	4615	4526	1.02	0.03	-0.08	-0.31
YOR012W		798	783	1.02	0.03	-0.08	-0.31
YPR195C		674	661	1.02	0.03	-0.08	-0.31
YIL036W		11528	11307	1.02	0.03	-0.08	-0.31
YML117W		1516	1487	1.02	0.03	-0.08	-0.31
YAL063C	FLO9	774	759	1.02	0.03	-0.08	-0.31
YPL257W		4807	4715	1.02	0.03	-0.08	-0.31
YOR196C	LIP5	6962	6830	1.02	0.03	-0.08	-0.31
YKL190W	CNB1	4905	4812	1.02	0.03	-0.08	-0.31
YFL006W		2509	2461	1.02	0.03	-0.08	-0.31
YER147C		3722	3652	1.02	0.03	-0.08	-0.31
YNL189W	SRP1	19933	19557	1.02	0.03	-0.08	-0.31
YER152C		5519	5415	1.02	0.03	-0.09	-0.31
YAL034C	FUN19	1806	1772	1.02	0.03	-0.09	-0.31
YLR126C		3599	3532	1.02	0.03	-0.09	-0.31
YEL001C		3986	3912	1.02	0.03	-0.09	-0.31
YER090W	TRP2	1368	1343	1.02	0.03	-0.09	-0.31
YER165W	PAB1	35062	34411	1.02	0.03	-0.09	-0.31
YKL225W		764	750	1.02	0.03	-0.09	-0.31
YGR174C	CBP4	6957	6828	1.02	0.03	-0.09	-0.31
YLR460C		10764	10565	1.02	0.03	-0.09	-0.31
YER039C	HVG1	972	954	1.02	0.03	-0.09	-0.31
YKR096W		1456	1429	1.02	0.03	-0.09	-0.31
YPL138C		9810	9629	1.02	0.03	-0.09	-0.31
YLR338W		935	918	1.02	0.03	-0.09	-0.31
YGL250W		2157	2118	1.02	0.03	-0.09	-0.31
YDL155W	CLB3	7511	7375	1.02	0.03	-0.09	-0.31
YJR091C	JSN1	1364	1340	1.02	0.03	-0.09	-0.31
YDR180W	SCC2	5232	5139	1.02	0.03	-0.09	-0.31

YOR062C		2879	2829	1.02	0.03	-0.09	-0.32
YGL006W	PMC1	2142	2105	1.02	0.03	-0.09	-0.32
YBL044W		1183	1162	1.02	0.03	-0.09	-0.32
YPL077C		1481	1455	1.02	0.03	-0.09	-0.32
YLR125W		2189	2151	1.02	0.03	-0.09	-0.32
YIL101C	XBP1	1058	1040	1.02	0.02	-0.09	-0.32
YML128C		2708	2662	1.02	0.02	-0.09	-0.32
YBL102W	SFT2	1383	1360	1.02	0.02	-0.09	-0.32
YOL002C		8681	8534	1.02	0.02	-0.09	-0.32
YLR160C	ASP3-4	13398	13172	1.02	0.02	-0.09	-0.32
YNL080C		11768	11569	1.02	0.02	-0.09	-0.32
YGL083W	SCY1	4813	4733	1.02	0.02	-0.09	-0.32
YPL001W	HAT1	5921	5822	1.02	0.02	-0.09	-0.32
YDR139C	RUB1	5873	5776	1.02	0.02	-0.09	-0.32
YMR007W		1773	1744	1.02	0.02	-0.09	-0.32
YGR277C		18718	18414	1.02	0.02	-0.09	-0.32
YLR102C	APC9	2267	2230	1.02	0.02	-0.09	-0.32
YBR281C		8407	8272	1.02	0.02	-0.09	-0.32
YKR058W	GLG1	2269	2233	1.02	0.02	-0.09	-0.32
YHL033C	RPL8A	40534	39888	1.02	0.02	-0.09	-0.32
YBL056W	PTC3	14015	13792	1.02	0.02	-0.09	-0.32
YDR002W	YRB1	29114	28651	1.02	0.02	-0.09	-0.32
YKL128C	PMU1	7915	7790	1.02	0.02	-0.09	-0.33
YPL162C		514	506	1.02	0.02	-0.09	-0.33
YIL152W		2957	2911	1.02	0.02	-0.09	-0.33
YIL004C	BET1	2913	2868	1.02	0.02	-0.09	-0.33
YMR085W		13295	13091	1.02	0.02	-0.09	-0.33
YDR119W		14477	14256	1.02	0.02	-0.09	-0.33
YNL235C		2545	2507	1.02	0.02	-0.09	-0.33
YLR077W		12220	12036	1.02	0.02	-0.09	-0.33
YGR060W	ERG25	3072	3026	1.02	0.02	-0.09	-0.33
YGR029W	ERV1	10323	10173	1.01	0.02	-0.09	-0.33
YGR223C		2909	2867	1.01	0.02	-0.09	-0.33
YBR229C	ROT2	6884	6786	1.01	0.02	-0.09	-0.33
YGR246C	BRF1	6134	6048	1.01	0.02	-0.09	-0.33
YHR134W		3493	3444	1.01	0.02	-0.09	-0.33

YOL153C		3475	3427	1.01	0.02	-0.09	-0.34
YPL199C		11556	11397	1.01	0.02	-0.09	-0.34
YIL120W		2465	2431	1.01	0.02	-0.09	-0.34
YMR272C	SCS7	3128	3085	1.01	0.02	-0.09	-0.34
YOL111C		8492	8376	1.01	0.02	-0.09	-0.34
YKL160W		14243	14049	1.01	0.02	-0.09	-0.34
YBR209W		1867	1842	1.01	0.02	-0.09	-0.34
YNR051C		5482	5410	1.01	0.02	-0.09	-0.34
YBR249C	ARO4	46128	45524	1.01	0.02	-0.09	-0.34
YOR227W		2971	2932	1.01	0.02	-0.09	-0.34
YOL083W		3126	3086	1.01	0.02	-0.09	-0.34
YIL084C	SDS3	4715	4655	1.01	0.02	-0.09	-0.34
YMR149W	SWP1	10258	10128	1.01	0.02	-0.09	-0.34
YDR240C	SNU56	14194	14016	1.01	0.02	-0.09	-0.34
YBR162W-A	YSY6	15869	15670	1.01	0.02	-0.09	-0.34
YOR147W		11036	10898	1.01	0.02	-0.09	-0.34
YNL287W	SEC21	14528	14346	1.01	0.02	-0.09	-0.34
YPR198W	SGE1	2265	2237	1.01	0.02	-0.09	-0.34
YHL012W		1054	1041	1.01	0.02	-0.09	-0.34
YER101C	AST2	4386	4332	1.01	0.02	-0.09	-0.34
YPR057W	BRR1	6596	6516	1.01	0.02	-0.09	-0.34
YNL191W		3506	3464	1.01	0.02	-0.09	-0.34
YNL257C	SIP3	4157	4107	1.01	0.02	-0.09	-0.34
YMR190C	SGS1	1981	1957	1.01	0.02	-0.09	-0.35
YOL126C	MDH2	5852	5782	1.01	0.02	-0.09	-0.35
YCRX15W		1754	1733	1.01	0.02	-0.10	-0.35
YNL246W		2629	2598	1.01	0.02	-0.10	-0.35
YDR035W	ARO3	5873	5804	1.01	0.02	-0.10	-0.35
YLR191W	PEX13	9899	9783	1.01	0.02	-0.10	-0.35
YNL209W	SSB2	52280	51672	1.01	0.02	-0.10	-0.35
YDL118W		4366	4316	1.01	0.02	-0.10	-0.35
YPL271W	ATP15	11655	11522	1.01	0.02	-0.10	-0.35
YOL164W		1031	1019	1.01	0.02	-0.10	-0.35
YNR062C		2863	2830	1.01	0.02	-0.10	-0.35
YOR068C		1128	1115	1.01	0.02	-0.10	-0.35
YNR008W	LRO1	10123	10008	1.01	0.02	-0.10	-0.35

YNL042W	BOP3	8398	8303	1.01	0.02	-0.10	-0.35
YOL035C		2909	2877	1.01	0.02	-0.10	-0.35
YKL077W		8154	8065	1.01	0.02	-0.10	-0.35
YDL243C	AAD4	411	407	1.01	0.02	-0.10	-0.35
YMR196W		384	380	1.01	0.02	-0.10	-0.35
YNL284C	MRPL10	8476	8385	1.01	0.02	-0.10	-0.35
YHL016C	DUR3	1645	1627	1.01	0.02	-0.10	-0.35
YMR322C		1039	1028	1.01	0.02	-0.10	-0.35
YLR322W		2543	2516	1.01	0.02	-0.10	-0.35
YOL051W	GAL11	6980	6907	1.01	0.02	-0.10	-0.35
YDL091C		1286	1273	1.01	0.02	-0.10	-0.35
YHR067W		748	740	1.01	0.02	-0.10	-0.35
YIL139C	REV7	7338	7263	1.01	0.01	-0.10	-0.35
YDR098C	GRX3	14584	14434	1.01	0.01	-0.10	-0.35
YGR056W	RSC1	3922	3882	1.01	0.01	-0.10	-0.35
YHL006C		1219	1207	1.01	0.01	-0.10	-0.36
YJR011C		6023	5963	1.01	0.01	-0.10	-0.36
YLR380W	CSR1	6150	6089	1.01	0.01	-0.10	-0.36
YDR181C	SAS4	2812	2784	1.01	0.01	-0.10	-0.36
YDL042C	SIR2	4946	4897	1.01	0.01	-0.10	-0.36
YLL066C		7938	7860	1.01	0.01	-0.10	-0.36
YPR074C	TKL1	15584	15431	1.01	0.01	-0.10	-0.36
YPL216W		6103	6043	1.01	0.01	-0.10	-0.36
YMR026C	PEX12	4485	4442	1.01	0.01	-0.10	-0.36
YHR104W	GRE3	6961	6894	1.01	0.01	-0.10	-0.36
YNL107W		4548	4505	1.01	0.01	-0.10	-0.36
YPR041W	TIF5	24673	24439	1.01	0.01	-0.10	-0.36
YIL176C		2212	2191	1.01	0.01	-0.10	-0.36
YKL223W		5526	5474	1.01	0.01	-0.10	-0.36
YKL138C	MRPL31	4796	4751	1.01	0.01	-0.10	-0.36
YDL028C	MPS1	953	944	1.01	0.01	-0.10	-0.36
YDL053C		7716	7647	1.01	0.01	-0.10	-0.36
YDR334W	SWR1	5222	5176	1.01	0.01	-0.10	-0.36
YBL030C	PET9	66009	65428	1.01	0.01	-0.10	-0.36
YJL185C		1386	1374	1.01	0.01	-0.10	-0.36
YGR202C	PCT1	12677	12566	1.01	0.01	-0.10	-0.36

YGR260W		3645	3613	1.01	0.01	-0.10	-0.36
YLR201C		12286	12180	1.01	0.01	-0.10	-0.36
YGL226W		4973	4931	1.01	0.01	-0.10	-0.36
YPR139C		4068	4034	1.01	0.01	-0.10	-0.36
YLR081W	GAL2	984	976	1.01	0.01	-0.10	-0.36
YFL-TYB		37665	37350	1.01	0.01	-0.10	-0.36
YPL127C	HHO1	5517	5472	1.01	0.01	-0.10	-0.37
YOR173W		4988	4948	1.01	0.01	-0.10	-0.37
YNR044W	AGA1	6258	6208	1.01	0.01	-0.10	-0.37
YNL203C		660	655	1.01	0.01	-0.10	-0.37
YDR326C		8354	8290	1.01	0.01	-0.10	-0.37
YLR314C	CDC3	13594	13494	1.01	0.01	-0.10	-0.37
YBR091C	MRS5	9796	9727	1.01	0.01	-0.10	-0.37
YIL082W-A		1456	1446	1.01	0.01	-0.10	-0.37
YHR089C	GAR1	13133	13044	1.01	0.01	-0.10	-0.37
YPR076W		10101	10033	1.01	0.01	-0.10	-0.37
YDL090C	RAM1	9383	9321	1.01	0.01	-0.10	-0.37
YIL043C	CBR1	9783	9718	1.01	0.01	-0.10	-0.37
YHL037C		756	751	1.01	0.01	-0.10	-0.37
YKL204W		1911	1899	1.01	0.01	-0.10	-0.38
YPL258C	THI21	3036	3017	1.01	0.01	-0.10	-0.38
YKR049C		4379	4351	1.01	0.01	-0.10	-0.38
YDR268W	MSW1	4068	4043	1.01	0.01	-0.10	-0.38
YDR054C	CDC34	12135	12060	1.01	0.01	-0.10	-0.38
YBL066C	SEF1	7163	7119	1.01	0.01	-0.10	-0.38
YDR092W	UBC13	10211	10148	1.01	0.01	-0.10	-0.38
YKL205W	LOS1	5598	5564	1.01	0.01	-0.10	-0.38
YGL175C	SAE2	857	852	1.01	0.01	-0.10	-0.38
YDR124W		2152	2139	1.01	0.01	-0.10	-0.38
YGL142C	GPI10	3058	3040	1.01	0.01	-0.10	-0.38
YLR269C		4328	4303	1.01	0.01	-0.10	-0.38
YCL048W		422	420	1.01	0.01	-0.10	-0.38
YJR007W	SUI2	9311	9259	1.01	0.01	-0.10	-0.38
YLR459W	CDC91	7074	7035	1.01	0.01	-0.10	-0.38
YCRX17W		2130	2118	1.01	0.01	-0.10	-0.38
YBR114W	RAD16	1129	1123	1.01	0.01	-0.10	-0.38

YOL147C	PEX11	8421	8375	1.01	0.01	-0.10	-0.38
YEL044W		8986	8938	1.01	0.01	-0.10	-0.38
YCR036W	RBK1	6554	6519	1.01	0.01	-0.10	-0.38
YDR332W		2021	2010	1.01	0.01	-0.10	-0.38
YOR222W		22861	22745	1.01	0.01	-0.11	-0.38
YLR011W		3750	3731	1.01	0.01	-0.11	-0.38
YPR121W	THI22	2474	2462	1.01	0.01	-0.11	-0.38
YLR055C	SPT8	4720	4697	1.00	0.01	-0.11	-0.38
YJL110C	GZF3	19971	19873	1.00	0.01	-0.11	-0.38
YIL082W		2800	2786	1.00	0.01	-0.11	-0.38
YOL047C		607	604	1.00	0.01	-0.11	-0.38
YPL055C		2375	2363	1.00	0.01	-0.11	-0.38
YIL106W	MOB1	5803	5776	1.00	0.01	-0.11	-0.38
YKL210W	UBA1	1102	1097	1.00	0.01	-0.11	-0.39
YLR293C	GSP1	34828	34676	1.00	0.01	-0.11	-0.39
YOL156W	HXT11	1215	1210	1.00	0.01	-0.11	-0.39
YLR154C		5709	5684	1.00	0.01	-0.11	-0.39
YPR177C		7013	6984	1.00	0.01	-0.11	-0.39
YDR260C	SWM1	13799	13744	1.00	0.01	-0.11	-0.39
YGL025C	PGD1	14230	14175	1.00	0.01	-0.11	-0.39
YGR220C	MRPL9	7866	7836	1.00	0.01	-0.11	-0.39
YPR051W	MAK3	7173	7146	1.00	0.01	-0.11	-0.39
YNL138W	SRV2	11662	11619	1.00	0.01	-0.11	-0.39
YNL048W	ALG11	4965	4947	1.00	0.01	-0.11	-0.39
YGR288W	MAL13	1959	1952	1.00	0.01	-0.11	-0.39
YKL082C		6730	6708	1.00	0.00	-0.11	-0.39
YNL050C		6016	5997	1.00	0.00	-0.11	-0.39
YBL076C	ILS1	24540	24465	1.00	0.00	-0.11	-0.39
YOL157C		494	492	1.00	0.00	-0.11	-0.39
YJR014W		6964	6943	1.00	0.00	-0.11	-0.39
YEL018W		13371	13332	1.00	0.00	-0.11	-0.39
YBR185C	MBA1	7368	7347	1.00	0.00	-0.11	-0.39
YCR024C		4878	4864	1.00	0.00	-0.11	-0.39
YOR321W	PMT3	2976	2968	1.00	0.00	-0.11	-0.39
YKL187C		1716	1711	1.00	0.00	-0.11	-0.39
YOL142W	RRP40	13330	13294	1.00	0.00	-0.11	-0.39

YBR165W	UBS1	9121	9097	1.00	0.00	-0.11	-0.39
YER183C		5925	5910	1.00	0.00	-0.11	-0.40
YDR165W		4053	4044	1.00	0.00	-0.11	-0.40
YBR024W	SCO2	804	802	1.00	0.00	-0.11	-0.40
YPL265W	DIP5	22190	22141	1.00	0.00	-0.11	-0.40
YBR183W	YPC1	12160	12134	1.00	0.00	-0.11	-0.40
YDR225W	HTA1	28666	28605	1.00	0.00	-0.11	-0.40
YPR066W	UBA3	1989	1985	1.00	0.00	-0.11	-0.40
YJR009C	TDH2	50608	50511	1.00	0.00	-0.11	-0.40
YMR043W	MCM1	2828	2823	1.00	0.00	-0.11	-0.40
YHR050W	SMF2	16796	16765	1.00	0.00	-0.11	-0.40
YPR100W		18498	18464	1.00	0.00	-0.11	-0.40
YBR110W	ALG1	6880	6868	1.00	0.00	-0.11	-0.40
YGL196W		6980	6968	1.00	0.00	-0.11	-0.40
YJR031C	GEA1	1707	1705	1.00	0.00	-0.11	-0.40
YER043C	SAH1	14284	14265	1.00	0.00	-0.11	-0.40
YLR345W		1310	1308	1.00	0.00	-0.11	-0.40
YJR021C	REC107	7550	7541	1.00	0.00	-0.11	-0.40
YPL089C	RLM1	2967	2963	1.00	0.00	-0.11	-0.40
YKR074W		9578	9569	1.00	0.00	-0.11	-0.40
YAL015C	NTG1	2066	2064	1.00	0.00	-0.11	-0.40
YOR047C	STD1	16440	16429	1.00	0.00	-0.11	-0.41
YLR265C		6971	6966	1.00	0.00	-0.11	-0.41
YLR150W	STM1	33936	33919	1.00	0.00	-0.11	-0.41
YLR331C		5091	5089	1.00	0.00	-0.11	-0.41
YOR105W		5739	5737	1.00	0.00	-0.11	-0.41
YEL013W	VAC8	23915	23911	1.00	0.00	-0.11	-0.41
YDR456W	NHX1	3193	3193	1.00	0.00	-0.11	-0.41
YKL179C		2552	2552	1.00	0.00	-0.11	-0.41
YDL197C	ASF2	8316	8316	1.00	0.00	-0.11	-0.41
YMR052W	FAR3	1795	1795	1.00	0.00	-0.11	-0.41
YML068W		2562	2562	1.00	0.00	-0.11	-0.41
YDR090C		7347	7347	1.00	0.00	-0.11	-0.41
YGL119W	ABC1	14119	14122	1.00	0.00	-0.11	-0.41
YPR134W	MSS18	1589	1589	1.00	0.00	-0.11	-0.41
YDR540C		2953	2954	1.00	0.00	-0.11	-0.41

YFL009W	CDC4	3470	3471	1.00	0.00	-0.11	-0.41
YKR020W		4297	4299	1.00	0.00	-0.11	-0.41
YOR255W		1940	1941	1.00	0.00	-0.11	-0.41
YGL063W	PUS2	1620	1621	1.00	0.00	-0.11	-0.41
YGL069C		25614	25631	1.00	0.00	-0.11	-0.41
YOL001W	PHO80	7560	7565	1.00	0.00	-0.11	-0.41
YAR066W		2298	2300	1.00	0.00	-0.11	-0.41
YPR099C		11374	11385	1.00	0.00	-0.11	-0.41
YDR298C	ATP5	15751	15768	1.00	0.00	-0.11	-0.41
YPL219W	PCL8	4115	4120	1.00	0.00	-0.11	-0.42
YIL168W	SDL1	1594	1596	1.00	0.00	-0.11	-0.42
YNL150W		13826	13845	1.00	0.00	-0.11	-0.42
YHR100C		3583	3588	1.00	0.00	-0.11	-0.42
YGL223C		6841	6851	1.00	0.00	-0.11	-0.42
YPL091W	GLR1	8180	8193	1.00	0.00	-0.11	-0.42
YJR020W		1118	1120	1.00	0.00	-0.11	-0.42
YOR204W	DED1	17820	17848	1.00	0.00	-0.11	-0.42
YLR452C	SST2	12074	12094	1.00	0.00	-0.11	-0.42
YDR388W	RVS167	7854	7867	1.00	0.00	-0.11	-0.42
YCL016C		15163	15189	1.00	0.00	-0.11	-0.42
YGL070C	RPB9	10238	10256	1.00	0.00	-0.11	-0.42
YHR190W	ERG9	540	541	1.00	0.00	-0.12	-0.42
YDL088C	ASM4	7125	7140	1.00	0.00	-0.12	-0.42
YKL189W	HYM1	2476	2481	1.00	0.00	-0.12	-0.42
YOR210W	RPB10	11174	11199	1.00	0.00	-0.12	-0.42
YHR176W		606	607	1.00	0.00	-0.12	-0.42
YKL035W	UGP1	7925	7944	1.00	0.00	-0.12	-0.42
YOR190W	SPR1	1229	1232	1.00	0.00	-0.12	-0.42
YMR281W	GPI12	6448	6465	1.00	0.00	-0.12	-0.42
YPR138C	MEP3	6943	6962	1.00	0.00	-0.12	-0.42
YEL050C	RML2	17624	17672	1.00	0.00	-0.12	-0.42
YDR196C		9792	9819	1.00	0.00	-0.12	-0.42
YFL068W		14001	14041	1.00	0.00	-0.12	-0.42
YOR249C	APC5	1113	1116	1.00	0.00	-0.12	-0.42
YER150W	SPI1	1874	1880	1.00	0.00	-0.12	-0.42
YAL036C	FUN11	31292	31389	1.00	0.00	-0.12	-0.42

YDR344C		1848	1854	1.00	0.00	-0.12	-0.42
YNL337W		1366	1370	1.00	0.00	-0.12	-0.43
YBR028C		1701	1706	1.00	0.00	-0.12	-0.43
YJL160C		1100	1104	1.00	0.00	-0.12	-0.43
YGL220W		15785	15836	1.00	0.00	-0.12	-0.43
YML048W	GSF2	7928	7954	1.00	0.00	-0.12	-0.43
YLR456W		7475	7500	1.00	0.00	-0.12	-0.43
YLR019W		7539	7565	1.00	0.00	-0.12	-0.43
YGL100W	SEH1	3800	3814	1.00	-0.01	-0.12	-0.43
YKL047W		7214	7240	1.00	-0.01	-0.12	-0.43
YML105C	SEC65	6824	6849	1.00	-0.01	-0.12	-0.43
YMR285C		15070	15128	1.00	-0.01	-0.12	-0.43
YJL059W	YHC3	2735	2746	1.00	-0.01	-0.12	-0.43
YPR125W		11304	11348	1.00	-0.01	-0.12	-0.43
YOR313C	SPS4	5003	5023	1.00	-0.01	-0.12	-0.43
YGL145W	TIP20	4057	4074	1.00	-0.01	-0.12	-0.43
YGR166W	KRE11	4424	4442	1.00	-0.01	-0.12	-0.43
YOR065W	CYT1	6250	6276	1.00	-0.01	-0.12	-0.43
YDR502C	SAM2	8681	8717	1.00	-0.01	-0.12	-0.43
YNR006W	VPS27	9854	9895	1.00	-0.01	-0.12	-0.43
YFR006W		12020	12072	1.00	-0.01	-0.12	-0.43
YJL053W	PEP8	10809	10856	1.00	-0.01	-0.12	-0.43
YFL042C		2205	2215	1.00	-0.01	-0.12	-0.43
YMR037C	MSN2	1694	1702	1.00	-0.01	-0.12	-0.43
YJL122W		34277	34448	1.00	-0.01	-0.12	-0.43
YGL026C	TRP5	8895	8940	1.00	-0.01	-0.12	-0.43
YJL208C	NUC1	8692	8737	0.99	-0.01	-0.12	-0.44
YLR005W	SSL1	9379	9429	0.99	-0.01	-0.12	-0.44
YLR463C		3290	3308	0.99	-0.01	-0.12	-0.44
YDR270W	CCC2	2450	2463	0.99	-0.01	-0.12	-0.44
YCR104W	PAU3	2135	2147	0.99	-0.01	-0.12	-0.44
YCR071C	IMG2	14090	14169	0.99	-0.01	-0.12	-0.44
YHR081W		13624	13702	0.99	-0.01	-0.12	-0.44
YBR067C	TIP1	15985	16077	0.99	-0.01	-0.12	-0.44
YJL139C	YUR1	1978	1990	0.99	-0.01	-0.12	-0.44
YBR077C		15643	15738	0.99	-0.01	-0.12	-0.44

YLR433C	CNA1	2720	2737	0.99	-0.01	-0.12	-0.44
YIL023C		6895	6938	0.99	-0.01	-0.12	-0.44
YER184C		2827	2845	0.99	-0.01	-0.12	-0.44
YLL016W	SDC25	3368	3390	0.99	-0.01	-0.12	-0.44
YPL002C	SNF8	5237	5271	0.99	-0.01	-0.12	-0.44
YDR537C		5794	5831	0.99	-0.01	-0.12	-0.44
YGR137W		4727	4758	0.99	-0.01	-0.12	-0.44
YMR077C		5869	5907	0.99	-0.01	-0.12	-0.44
YLL052C	AQY2	7266	7315	0.99	-0.01	-0.12	-0.44
YBR235W		3614	3639	0.99	-0.01	-0.12	-0.44
YOR003W	YSP3	1388	1397	0.99	-0.01	-0.12	-0.44
YJL206C		1717	1729	0.99	-0.01	-0.12	-0.45
YDR156W	RPA14	4961	4996	0.99	-0.01	-0.12	-0.45
YLR036C		8152	8211	0.99	-0.01	-0.12	-0.45
YJR137C	ECM17	1835	1848	0.99	-0.01	-0.12	-0.45
YPL208W		11979	12068	0.99	-0.01	-0.12	-0.45
YLR438W	CAR2	3981	4011	0.99	-0.01	-0.12	-0.45
YPL044C		11459	11546	0.99	-0.01	-0.12	-0.45
YLR444C		6014	6060	0.99	-0.01	-0.12	-0.45
YML042W	CAT2	3289	3315	0.99	-0.01	-0.12	-0.45
YLR069C	MEF1	11347	11436	0.99	-0.01	-0.12	-0.45
YDR282C		2040	2056	0.99	-0.01	-0.12	-0.45
YLR315W		2567	2588	0.99	-0.01	-0.12	-0.45
YNL115C		4208	4242	0.99	-0.01	-0.12	-0.45
YGL003C	CDH1	3485	3513	0.99	-0.01	-0.12	-0.45
YGR002C		5517	5563	0.99	-0.01	-0.12	-0.45
YPR080W	TEF1	49461	49871	0.99	-0.01	-0.12	-0.45
YCR007C		1162	1172	0.99	-0.01	-0.12	-0.45
YGR216C	GPI1	8354	8426	0.99	-0.01	-0.12	-0.45
YLR001C		4030	4065	0.99	-0.01	-0.12	-0.45
YCRX07W		3573	3605	0.99	-0.01	-0.13	-0.45
YDR458C		2279	2299	0.99	-0.01	-0.13	-0.45
YMR320W		826	833	0.99	-0.01	-0.13	-0.45
YGL187C	COX4	15531	15671	0.99	-0.01	-0.13	-0.46
YDR056C		17508	17666	0.99	-0.01	-0.13	-0.46
YHL044W		890	898	0.99	-0.01	-0.13	-0.46

YHR042W	NCP1	13176	13295	0.99	-0.01	-0.13	-0.46
YKL126W	YPK1	1945	1963	0.99	-0.01	-0.13	-0.46
YLR046C		2282	2303	0.99	-0.01	-0.13	-0.46
YPL180W		6785	6849	0.99	-0.01	-0.13	-0.46
YGL085W		8748	8831	0.99	-0.01	-0.13	-0.46
YPL190C	NAB3	11106	11212	0.99	-0.01	-0.13	-0.46
YML058W	SML1	12856	12979	0.99	-0.01	-0.13	-0.46
YMR038C	LYS7	23059	23282	0.99	-0.01	-0.13	-0.46
YIR019C	MUC1	708	715	0.99	-0.01	-0.13	-0.46
YJL196C	ELO1	5795	5852	0.99	-0.01	-0.13	-0.46
YAR008W	SEN34	17299	17475	0.99	-0.01	-0.13	-0.46
YOL088C	MPD2	20079	20287	0.99	-0.01	-0.13	-0.46
YOR384W	FRE5	846	855	0.99	-0.01	-0.13	-0.46
YJL036W	SNX4	9429	9527	0.99	-0.01	-0.13	-0.46
YLR261C		6969	7042	0.99	-0.01	-0.13	-0.46
YDL209C		11400	11519	0.99	-0.02	-0.13	-0.46
YLR215C		8451	8540	0.99	-0.02	-0.13	-0.46
YDR353W	TRR1	24874	25140	0.99	-0.02	-0.13	-0.46
YPR188C		8119	8207	0.99	-0.02	-0.13	-0.47
YKL127W	PGM1	10258	10370	0.99	-0.02	-0.13	-0.47
YEL076C		1170	1183	0.99	-0.02	-0.13	-0.47
YPL041C		1599	1617	0.99	-0.02	-0.13	-0.47
YBL064C		14245	14401	0.99	-0.02	-0.13	-0.47
YKL140W	TGL1	3746	3787	0.99	-0.02	-0.13	-0.47
YFL029C	CAK1	2217	2242	0.99	-0.02	-0.13	-0.47
YEL076W-C		2113	2137	0.99	-0.02	-0.13	-0.47
YPL108W		12655	12796	0.99	-0.02	-0.13	-0.47
YLR042C		2185	2209	0.99	-0.02	-0.13	-0.47
YPR114W		10852	10974	0.99	-0.02	-0.13	-0.47
YIL138C	TPM2	15161	15332	0.99	-0.02	-0.13	-0.47
YHR112C		2454	2482	0.99	-0.02	-0.13	-0.47
YNL168C		20381	20616	0.99	-0.02	-0.13	-0.47
YKR086W	PRP16	1606	1625	0.99	-0.02	-0.13	-0.47
YOL105C	WSC3	3290	3329	0.99	-0.02	-0.13	-0.47
YER048C	CAJ1	2763	2796	0.99	-0.02	-0.13	-0.47
YGL125W	MET13	3755	3801	0.99	-0.02	-0.13	-0.47

YBR280C		1293	1309	0.99	-0.02	-0.13	-0.47
YOR194C	TOA1	11344	11483	0.99	-0.02	-0.13	-0.47
YBR272C	HSM3	2319	2347	0.99	-0.02	-0.13	-0.47
YFR036W	CDC26	4239	4292	0.99	-0.02	-0.13	-0.47
YIL046W	MET30	7482	7576	0.99	-0.02	-0.13	-0.47
YPL252C	YAH1	7196	7287	0.99	-0.02	-0.13	-0.47
YDR336W		740	749	0.99	-0.02	-0.13	-0.48
YBR222C	FAT2	2883	2920	0.99	-0.02	-0.13	-0.48
YCRX13W		2879	2916	0.99	-0.02	-0.13	-0.48
YBL107C		17753	17983	0.99	-0.02	-0.13	-0.48
YIL080W		2647	2681	0.99	-0.02	-0.13	-0.48
YJL146W	IDS2	6987	7078	0.99	-0.02	-0.13	-0.48
YDR145W	TAF61	10510	10647	0.99	-0.02	-0.13	-0.48
YER011W	TIR1	494	500	0.99	-0.02	-0.13	-0.48
YDR498C	SEC20	2236	2266	0.99	-0.02	-0.13	-0.48
YFL034W		1981	2007	0.99	-0.02	-0.13	-0.48
YEL051W	VMA8	13490	13672	0.99	-0.02	-0.13	-0.48
YBL057C		8957	9079	0.99	-0.02	-0.13	-0.48
YKL028W	TFA1	9245	9372	0.99	-0.02	-0.13	-0.48
YJL023C	PET130	4570	4633	0.99	-0.02	-0.13	-0.48
YDR296W	MHR1	19999	20277	0.99	-0.02	-0.13	-0.48
YMR238W	DFG5	5178	5251	0.99	-0.02	-0.13	-0.48
YGR224W		4109	4167	0.99	-0.02	-0.13	-0.48
YPL210C	SRP72	23639	23977	0.99	-0.02	-0.13	-0.48
YCR082W		15952	16182	0.99	-0.02	-0.13	-0.48
YIL056W		9289	9423	0.99	-0.02	-0.13	-0.48
YNL213C		6075	6163	0.99	-0.02	-0.13	-0.48
YML121W	GTR1	5219	5295	0.99	-0.02	-0.13	-0.48
YER166W		375	380	0.99	-0.02	-0.13	-0.48
YNR056C	BIO5	5330	5408	0.99	-0.02	-0.13	-0.48
YOR077W	RTS2	5163	5239	0.99	-0.02	-0.13	-0.48
YLR027C	AAT2	23165	23507	0.99	-0.02	-0.13	-0.49
YFL044C		2696	2736	0.99	-0.02	-0.13	-0.49
YHR041C	SRB2	4509	4576	0.99	-0.02	-0.13	-0.49
YIL088C		7818	7937	0.99	-0.02	-0.13	-0.49
YGR019W	UGA1	8384	8512	0.98	-0.02	-0.13	-0.49

YAL023C	PMT2	5695	5782	0.98	-0.02	-0.13	-0.49
YGR259C		3757	3814	0.98	-0.02	-0.13	-0.49
YJL120W		7474	7588	0.98	-0.02	-0.13	-0.49
YOR164C		525	533	0.98	-0.02	-0.13	-0.49
YNL040W		14010	14225	0.98	-0.02	-0.13	-0.49
YIL102C		1828	1856	0.98	-0.02	-0.13	-0.49
YDR358W		2901	2946	0.98	-0.02	-0.13	-0.49
YBR227C	MCX1	3002	3049	0.98	-0.02	-0.13	-0.49
YGR009C	SEC9	3604	3660	0.98	-0.02	-0.13	-0.49
YCR008W	SAT4	503	511	0.98	-0.02	-0.13	-0.49
YJL199C		707	718	0.98	-0.02	-0.14	-0.49
YKL175W		9073	9219	0.98	-0.02	-0.14	-0.49
YMR264W	CUE1	6986	7098	0.98	-0.02	-0.14	-0.49
YLR219W		4368	4438	0.98	-0.02	-0.14	-0.49
YIL142W	CCT2	13954	14179	0.98	-0.02	-0.14	-0.49
YBR264C	YPT10	3930	3994	0.98	-0.02	-0.14	-0.49
YDL219W		3888	3952	0.98	-0.02	-0.14	-0.49
YMR010W		10786	10964	0.98	-0.02	-0.14	-0.49
YDR038C	ENA5	2898	2946	0.98	-0.02	-0.14	-0.49
YDL131W	LYS21	14876	15122	0.98	-0.02	-0.14	-0.49
YER146W	LSM5	13273	13492	0.98	-0.02	-0.14	-0.49
YJL021C		2904	2952	0.98	-0.02	-0.14	-0.50
YKL041W	VPS24	5992	6092	0.98	-0.02	-0.14	-0.50
YOR237W	HES1	491	499	0.98	-0.02	-0.14	-0.50
YGR052W		852	866	0.98	-0.02	-0.14	-0.50
YBR074W		2384	2425	0.98	-0.02	-0.14	-0.50
YKL108W	SLD2	2111	2147	0.98	-0.02	-0.14	-0.50
YKR026C	GCN3	15940	16214	0.98	-0.02	-0.14	-0.50
YBR102C	EXO84	2859	2908	0.98	-0.02	-0.14	-0.50
YGL168W		5296	5388	0.98	-0.02	-0.14	-0.50
YFL058W	THI5	1584	1612	0.98	-0.02	-0.14	-0.50
YGL096W		839	854	0.98	-0.02	-0.14	-0.50
YDR425W		2514	2558	0.98	-0.03	-0.14	-0.50
YJL044C	GYP6	4621	4702	0.98	-0.03	-0.14	-0.50
YLR343W		667	679	0.98	-0.03	-0.14	-0.50
YHR137W	ARO9	2301	2342	0.98	-0.03	-0.14	-0.50

YGR016W		1578	1606	0.98	-0.03	-0.14	-0.50
YER149C	PEA2	6236	6349	0.98	-0.03	-0.14	-0.50
YNL197C	WHI3	6082	6192	0.98	-0.03	-0.14	-0.50
YOR063W	RPL3	40100	40826	0.98	-0.03	-0.14	-0.50
YPL026C	SKS1	1572	1601	0.98	-0.03	-0.14	-0.50
YMR135C		5146	5241	0.98	-0.03	-0.14	-0.51
YDL189W		9729	9910	0.98	-0.03	-0.14	-0.51
YBR166C	TYR1	7908	8056	0.98	-0.03	-0.14	-0.51
YOR187W	TUF1	37545	38246	0.98	-0.03	-0.14	-0.51
YMR062C	ECM40	14018	14280	0.98	-0.03	-0.14	-0.51
YBR233W	PBP2	5651	5758	0.98	-0.03	-0.14	-0.51
YLR446W		2358	2403	0.98	-0.03	-0.14	-0.51
YJL117W	PHO86	18020	18365	0.98	-0.03	-0.14	-0.51
YOR035C	SHE4	837	853	0.98	-0.03	-0.14	-0.51
YOL067C	RTG1	2041	2081	0.98	-0.03	-0.14	-0.51
YOR221C	MCT1	4012	4091	0.98	-0.03	-0.14	-0.51
YNL317W	PFS2	7770	7923	0.98	-0.03	-0.14	-0.51
YLR426W		9323	9509	0.98	-0.03	-0.14	-0.51
YDL165W	CDC36	7543	7694	0.98	-0.03	-0.14	-0.51
YLR033W		6913	7052	0.98	-0.03	-0.14	-0.51
YHR108W		2306	2353	0.98	-0.03	-0.14	-0.51
YLL049W		6675	6811	0.98	-0.03	-0.14	-0.51
YML102C-A		1599	1632	0.98	-0.03	-0.14	-0.51
YGR189C	CRH1	2291	2338	0.98	-0.03	-0.14	-0.51
YPL020C	ULP1	4083	4167	0.98	-0.03	-0.14	-0.51
YIL164C	NIT1	6394	6525	0.98	-0.03	-0.14	-0.52
YMR201C	RAD14	3989	4071	0.98	-0.03	-0.14	-0.52
YJL054W	TIM54	9273	9466	0.98	-0.03	-0.14	-0.52
YJL111W	CCT7	12630	12896	0.98	-0.03	-0.14	-0.52
YOR002W	ALG6	11758	12006	0.98	-0.03	-0.14	-0.52
YIR009W	MSL1	4048	4134	0.98	-0.03	-0.14	-0.52
YOR134W	BAG7	634	648	0.98	-0.03	-0.14	-0.52
YOR151C	RPB2	19744	20174	0.98	-0.03	-0.14	-0.52
YMR231W	PEP5	3128	3196	0.98	-0.03	-0.14	-0.52
YIR039C	YPS6	1394	1425	0.98	-0.03	-0.14	-0.52
YDL014W	NOP1	25013	25563	0.98	-0.03	-0.14	-0.52

YCR095C		5329	5446	0.98	-0.03	-0.14	-0.52
YCR076C		3325	3398	0.98	-0.03	-0.14	-0.52
YJL004C	SYS1	18018	18419	0.98	-0.03	-0.14	-0.52
YOR130C	ORT1	4897	5007	0.98	-0.03	-0.14	-0.53
YMR311C	GLC8	10633	10873	0.98	-0.03	-0.14	-0.53
YDL225W	SHS1	6582	6731	0.98	-0.03	-0.14	-0.53
YBR087W	RFC5	9385	9597	0.98	-0.03	-0.14	-0.53
YNL153C	GIM3	11963	12237	0.98	-0.03	-0.15	-0.53
YIL032C		1036	1060	0.98	-0.03	-0.15	-0.53
YIL005W		9377	9592	0.98	-0.03	-0.15	-0.53
YOR232W	MGE1	15710	16073	0.98	-0.03	-0.15	-0.53
YOR259C	RPT4	12785	13082	0.98	-0.03	-0.15	-0.53
YMR148W		3655	3741	0.98	-0.03	-0.15	-0.53
YPL274W	SAM3	14257	14593	0.98	-0.03	-0.15	-0.53
YLR412W		14402	14742	0.98	-0.03	-0.15	-0.53
YOR390W		12936	13242	0.98	-0.03	-0.15	-0.53
YDR430C		5103	5224	0.98	-0.03	-0.15	-0.53
YLR290C		5110	5231	0.98	-0.03	-0.15	-0.53
YOL139C	CDC33	26701	27338	0.98	-0.03	-0.15	-0.53
YHR098C	SFB3	6995	7163	0.98	-0.03	-0.15	-0.53
YJL147C		2144	2195	0.98	-0.03	-0.15	-0.53
YHR184W	SSP1	4925	5044	0.98	-0.03	-0.15	-0.53
YKL056C		39691	40646	0.98	-0.03	-0.15	-0.53
YBR239C		12585	12888	0.98	-0.03	-0.15	-0.53
YOL040C	RPS15	27046	27698	0.98	-0.03	-0.15	-0.53
YKL043W	PHD1	2671	2736	0.98	-0.03	-0.15	-0.53
YDR050C	TPI1	28556	29249	0.98	-0.03	-0.15	-0.53
YDL199C		4455	4564	0.98	-0.03	-0.15	-0.54
YML100W	TSL1	7237	7415	0.98	-0.04	-0.15	-0.54
YLL061W	MMP1	6282	6436	0.98	-0.04	-0.15	-0.54
YER086W	ILV1	6735	6902	0.98	-0.04	-0.15	-0.54
YPL203W	PKA3	2643	2708	0.98	-0.04	-0.15	-0.54
YCR069W	SCC3	18187	18639	0.98	-0.04	-0.15	-0.54
YOL003C		10399	10660	0.98	-0.04	-0.15	-0.54
YNL046W		4421	4533	0.98	-0.04	-0.15	-0.54
YGL155W	CDC43	4294	4403	0.98	-0.04	-0.15	-0.54

YMR139W	RIM11	7865	8065	0.98	-0.04	-0.15	-0.54
YGL067W		10932	11212	0.98	-0.04	-0.15	-0.54
YGR255C	COQ6	2868	2942	0.97	-0.04	-0.15	-0.54
YNL283C	WSC2	13278	13621	0.97	-0.04	-0.15	-0.54
YIL050W	PCL7	17561	18015	0.97	-0.04	-0.15	-0.54
YML038C	YMD8	16589	17020	0.97	-0.04	-0.15	-0.54
YKR022C		3824	3923	0.97	-0.04	-0.15	-0.54
YLR206W	ENT2	5830	5982	0.97	-0.04	-0.15	-0.54
YPR028W	YIP2	23835	24456	0.97	-0.04	-0.15	-0.54
YHR209W		1174	1205	0.97	-0.04	-0.15	-0.54
YER144C	UBP5	7405	7599	0.97	-0.04	-0.15	-0.54
YLR231C		2041	2095	0.97	-0.04	-0.15	-0.55
YHL049C		5847	6002	0.97	-0.04	-0.15	-0.55
YGR182C		17213	17670	0.97	-0.04	-0.15	-0.55
YJR044C		14185	14562	0.97	-0.04	-0.15	-0.55
YDL012C		8431	8655	0.97	-0.04	-0.15	-0.55
YER034W		12178	12502	0.97	-0.04	-0.15	-0.55
YGL062W	PYC1	468	480	0.97	-0.04	-0.15	-0.55
YFL030W		1318	1353	0.97	-0.04	-0.15	-0.55
YMR169C	ALD3	4365	4482	0.97	-0.04	-0.15	-0.55
YKL181W	PRS1	11747	12062	0.97	-0.04	-0.15	-0.55
YNR020C		6726	6907	0.97	-0.04	-0.15	-0.55
YHL032C	GUT1	2830	2906	0.97	-0.04	-0.15	-0.55
YPR045C		5578	5728	0.97	-0.04	-0.15	-0.55
YDR504C		1979	2033	0.97	-0.04	-0.15	-0.55
YDR486C		10802	11097	0.97	-0.04	-0.15	-0.55
YMR073C		6723	6907	0.97	-0.04	-0.15	-0.55
YLR386W		2163	2222	0.97	-0.04	-0.15	-0.55
YJL156C	SSY5	3426	3520	0.97	-0.04	-0.15	-0.55
YNL288W		6154	6324	0.97	-0.04	-0.15	-0.55
YIL001W		6407	6584	0.97	-0.04	-0.15	-0.55
YKR052C	MRS4	2823	2901	0.97	-0.04	-0.15	-0.55
YDL178W	AIP2	5621	5777	0.97	-0.04	-0.15	-0.55
YPR081C		2831	2910	0.97	-0.04	-0.15	-0.55
YBR089W		14884	15303	0.97	-0.04	-0.15	-0.55
YLR226W	BUR2	2942	3025	0.97	-0.04	-0.15	-0.55

YJL066C		12107	12450	0.97	-0.04	-0.15	-0.56
YDR327W		7241	7447	0.97	-0.04	-0.15	-0.56
YGR226C		1230	1266	0.97	-0.04	-0.15	-0.56
YLR420W	URA4	11408	11740	0.97	-0.04	-0.15	-0.56
YOR064C		3271	3367	0.97	-0.04	-0.15	-0.56
YDL203C		9811	10099	0.97	-0.04	-0.15	-0.56
YFR049W	YMR31	6113	6293	0.97	-0.04	-0.15	-0.56
YGR252W	GCN5	8224	8470	0.97	-0.04	-0.15	-0.56
YDL234C	GYP7	2986	3075	0.97	-0.04	-0.15	-0.56
YKL024C	URA6	14294	14722	0.97	-0.04	-0.15	-0.56
YGR130C		8146	8391	0.97	-0.04	-0.16	-0.56
YMR071C		16115	16600	0.97	-0.04	-0.16	-0.56
YDR131C		2056	2118	0.97	-0.04	-0.16	-0.56
YGR184C	UBR1	1058	1090	0.97	-0.04	-0.16	-0.56
YOR013W		2246	2314	0.97	-0.04	-0.16	-0.56
YOR081C		1967	2027	0.97	-0.04	-0.16	-0.57
YGL077C	HNM1	8619	8883	0.97	-0.04	-0.16	-0.57
YDR492W		7483	7713	0.97	-0.04	-0.16	-0.57
YLL019C	KNS1	6585	6788	0.97	-0.04	-0.16	-0.57
YHR035W		18627	19203	0.97	-0.04	-0.16	-0.57
YPR033C	HTS1	17806	18357	0.97	-0.04	-0.16	-0.57
YLR041W		21009	21662	0.97	-0.04	-0.16	-0.57
YMR301C	ATM1	9654	9955	0.97	-0.04	-0.16	-0.57
YCL011C	GBP2	31074	32047	0.97	-0.04	-0.16	-0.57
YJL168C	SET2	10901	11244	0.97	-0.04	-0.16	-0.57
YOR265W	RBL2	3815	3936	0.97	-0.04	-0.16	-0.57
YCR038C	BUD5	1783	1839	0.97	-0.04	-0.16	-0.57
YBR118W	TEF2	63427	65436	0.97	-0.04	-0.16	-0.57
YLR095C		1895	1955	0.97	-0.05	-0.16	-0.57
YKR007W		7532	7773	0.97	-0.05	-0.16	-0.57
YNL085W	MKT1	4313	4452	0.97	-0.05	-0.16	-0.57
YKL124W	SSH4	1363	1407	0.97	-0.05	-0.16	-0.58
YBR129C	OPY1	5960	6154	0.97	-0.05	-0.16	-0.58
YDL188C	PPH22	7367	7609	0.97	-0.05	-0.16	-0.58
YGR285C	ZUO1	29932	30924	0.97	-0.05	-0.16	-0.58
YHR012W	VPS29	11884	12281	0.97	-0.05	-0.16	-0.58

YML045W		55763	57632	0.97	-0.05	-0.16	-0.58
YBL010C		6671	6895	0.97	-0.05	-0.16	-0.58
YNR035C	ARC35	15470	15989	0.97	-0.05	-0.16	-0.58
YBR265W	TSC10	15515	16039	0.97	-0.05	-0.16	-0.58
YMR087W		2319	2397	0.97	-0.05	-0.16	-0.58
YNL334C	SNO2	1679	1736	0.97	-0.05	-0.16	-0.58
YHR031C	RTT104	2977	3079	0.97	-0.05	-0.16	-0.58
YGR231C	PHB2	12571	13001	0.97	-0.05	-0.16	-0.58
YOR174W	MED4	4503	4658	0.97	-0.05	-0.16	-0.59
YLR330W	CHS5	6402	6623	0.97	-0.05	-0.16	-0.59
YKL129C	MYO3	7126	7373	0.97	-0.05	-0.16	-0.59
YPL249C		2962	3065	0.97	-0.05	-0.16	-0.59
YCR088W	ABP1	9187	9505	0.97	-0.05	-0.16	-0.59
YLR396C	VPS33	3332	3447	0.97	-0.05	-0.16	-0.59
YDL208W	NHP2	15463	15999	0.97	-0.05	-0.16	-0.59
YAR002C-A	ERP1	679	703	0.97	-0.05	-0.16	-0.59
YMR108W	ILV2	18216	18849	0.97	-0.05	-0.16	-0.59
YLL039C	UBI4	7485	7745	0.97	-0.05	-0.16	-0.59
YDR253C	MET32	1899	1965	0.97	-0.05	-0.16	-0.59
YMR066W	SOV1	668	692	0.97	-0.05	-0.16	-0.59
YKL141W	SDH3	20530	21255	0.97	-0.05	-0.16	-0.59
YOR297C	TIM18	5029	5207	0.97	-0.05	-0.16	-0.59
YBR163W	DEM1	8770	9080	0.97	-0.05	-0.16	-0.59
YOL012C	HTA3	6554	6787	0.97	-0.05	-0.16	-0.59
YBR286W	APE3	21296	22055	0.97	-0.05	-0.16	-0.59
YIL077C		3564	3691	0.97	-0.05	-0.16	-0.59
YMR079W	SEC14	25263	26167	0.97	-0.05	-0.16	-0.59
YKL212W	SAC1	545	565	0.97	-0.05	-0.16	-0.59
YLR388W	RPS29A	29702	30770	0.97	-0.05	-0.16	-0.59
YML013W		2176	2255	0.97	-0.05	-0.16	-0.59
YBR213W	MET8	2209	2289	0.97	-0.05	-0.16	-0.59
YLL040C	VPS13	679	704	0.97	-0.05	-0.16	-0.60
YPL024W	NCE4	4004	4149	0.97	-0.05	-0.16	-0.60
YNL111C	CYB5	10422	10802	0.96	-0.05	-0.16	-0.60
YGR104C	SRB5	15085	15636	0.96	-0.05	-0.16	-0.60
YGR170W	PSD2	6599	6840	0.96	-0.05	-0.16	-0.60

YJR148W	BAT2	27414	28421	0.96	-0.05	-0.16	-0.60
YAL040C	CLN3	4938	5120	0.96	-0.05	-0.16	-0.60
YLR376C		2023	2098	0.96	-0.05	-0.16	-0.60
YJL145W		7424	7701	0.96	-0.05	-0.17	-0.60
YCRX02C		13763	14278	0.96	-0.05	-0.17	-0.60
YBL001C	ECM15	5167	5361	0.96	-0.05	-0.17	-0.60
YDR373W	FRQ1	19936	20687	0.96	-0.05	-0.17	-0.60
YGL245W		18502	19200	0.96	-0.05	-0.17	-0.60
YGR185C	TYS1	26544	27548	0.96	-0.05	-0.17	-0.60
YER060W	FCY21	1429	1483	0.96	-0.05	-0.17	-0.60
YDR192C	NUP42	13405	13919	0.96	-0.05	-0.17	-0.61
YDR314C		1884	1956	0.96	-0.05	-0.17	-0.61
YNL193W		1910	1983	0.96	-0.05	-0.17	-0.61
YDL007W	RPT2	18938	19666	0.96	-0.05	-0.17	-0.61
YDL161W	ENT1	14581	15142	0.96	-0.05	-0.17	-0.61
YKR070W		3338	3467	0.96	-0.05	-0.17	-0.61
YPL232W	SSO1	10943	11367	0.96	-0.05	-0.17	-0.61
YBR062C		2760	2867	0.96	-0.05	-0.17	-0.61
YJR018W		3114	3235	0.96	-0.05	-0.17	-0.61
YNL266W		2540	2639	0.96	-0.06	-0.17	-0.61
YLR390W	ECM19	3345	3476	0.96	-0.06	-0.17	-0.61
YML009C	MRPL39	16552	17204	0.96	-0.06	-0.17	-0.61
YMR209C		4127	4290	0.96	-0.06	-0.17	-0.61
YMR112C	MED11	5359	5572	0.96	-0.06	-0.17	-0.61
YMR028W	TAP42	3422	3558	0.96	-0.06	-0.17	-0.61
YLR342W	FKS1	5835	6068	0.96	-0.06	-0.17	-0.61
YML010W-A		2489	2589	0.96	-0.06	-0.17	-0.61
YOL068C	HST1	12822	13335	0.96	-0.06	-0.17	-0.61
YJL167W	ERG20	11885	12362	0.96	-0.06	-0.17	-0.62
YOR253W		19118	19892	0.96	-0.06	-0.17	-0.62
YJL138C	TIF2	62883	65432	0.96	-0.06	-0.17	-0.62
YER055C	HIS1	24666	25667	0.96	-0.06	-0.17	-0.62
YHR097C		5943	6184	0.96	-0.06	-0.17	-0.62
YMR226C		5698	5930	0.96	-0.06	-0.17	-0.62
YPR094W		11166	11621	0.96	-0.06	-0.17	-0.62
YDR362C	TFC6	2160	2248	0.96	-0.06	-0.17	-0.62

YBR128C	APG14	4369	4548	0.96	-0.06	-0.17	-0.62
YNL081C		10127	10544	0.96	-0.06	-0.17	-0.62
YDL139C		3397	3537	0.96	-0.06	-0.17	-0.62
YKL121W		2554	2659	0.96	-0.06	-0.17	-0.62
YGL022W	STT3	8472	8825	0.96	-0.06	-0.17	-0.62
YPL053C	KTR6	8235	8579	0.96	-0.06	-0.17	-0.62
YAL007C	ERP2	6272	6534	0.96	-0.06	-0.17	-0.62
YHR049C-A		13334	13897	0.96	-0.06	-0.17	-0.63
YHR177W		2059	2146	0.96	-0.06	-0.17	-0.63
YPR161C	SGV1	14737	15363	0.96	-0.06	-0.17	-0.63
YHR200W	RPN10	659	687	0.96	-0.06	-0.17	-0.63
YCR074C		1490	1554	0.96	-0.06	-0.17	-0.63
YJR139C	HOM6	18055	18835	0.96	-0.06	-0.17	-0.63
YML126C	HMGS	27784	28989	0.96	-0.06	-0.17	-0.63
YKL218C	SRY1	1273	1328	0.96	-0.06	-0.17	-0.63
YMR054W	STV1	1435	1498	0.96	-0.06	-0.17	-0.63
YOR061W	CKA2	26316	27465	0.96	-0.06	-0.17	-0.63
YPL238C		17552	18321	0.96	-0.06	-0.17	-0.63
YNL223W	AUT2	1532	1600	0.96	-0.06	-0.17	-0.63
YGL040C	HEM2	10845	11323	0.96	-0.06	-0.17	-0.64
YEL056W	HAT2	5662	5912	0.96	-0.06	-0.17	-0.64
YCLX01W		6331	6610	0.96	-0.06	-0.17	-0.64
YER007W	PAC2	1795	1875	0.96	-0.06	-0.18	-0.64
YHR082C	KSP1	1178	1231	0.96	-0.06	-0.18	-0.64
YPR185W	APG13	8025	8383	0.96	-0.06	-0.18	-0.64
YPL169C	MEX67	7763	8111	0.96	-0.06	-0.18	-0.64
YKR044W		715	747	0.96	-0.06	-0.18	-0.64
YKL209C	STE6	725	757	0.96	-0.06	-0.18	-0.64
YIL104C		16934	17693	0.96	-0.06	-0.18	-0.64
YHR139C-A		12860	13437	0.96	-0.06	-0.18	-0.64
YDR544C		3420	3574	0.96	-0.06	-0.18	-0.64
YMR060C	TOM37	7546	7886	0.96	-0.06	-0.18	-0.64
YGR012W		5431	5676	0.96	-0.06	-0.18	-0.64
YGR201C		3705	3872	0.96	-0.06	-0.18	-0.64
YBL012C		7314	7644	0.96	-0.06	-0.18	-0.64
YBR277C		5106	5337	0.96	-0.06	-0.18	-0.64

YKR061W	KTR2	3582	3744	0.96	-0.06	-0.18	-0.64
YJL002C	OST1	27725	28983	0.96	-0.06	-0.18	-0.64
YMR289W		9284	9705	0.96	-0.06	-0.18	-0.64
YNR041C	COQ2	5100	5332	0.96	-0.06	-0.18	-0.64
YMR263W		17569	18369	0.96	-0.06	-0.18	-0.64
YPL096W		7773	8128	0.96	-0.06	-0.18	-0.64
YPR047W	MSF1	2483	2596	0.96	-0.06	-0.18	-0.64
YCR011C	ADP1	2100	2196	0.96	-0.06	-0.18	-0.64
YKL147C		5165	5402	0.96	-0.06	-0.18	-0.64
YOL009C	MDM12	7653	8006	0.96	-0.07	-0.18	-0.65
YGL207W	SPT16	3724	3896	0.96	-0.07	-0.18	-0.65
YDR200C		10287	10763	0.96	-0.07	-0.18	-0.65
YBR228W		3526	3689	0.96	-0.07	-0.18	-0.65
YPR175W	DPB2	10703	11200	0.96	-0.07	-0.18	-0.65
YOL026C		9848	10307	0.96	-0.07	-0.18	-0.65
YNR007C	AUT1	2704	2830	0.96	-0.07	-0.18	-0.65
YDL064W	UBC9	20548	21508	0.96	-0.07	-0.18	-0.65
YBR068C	BAP2	16898	17689	0.96	-0.07	-0.18	-0.65
YIL003W		11280	11810	0.96	-0.07	-0.18	-0.65
YMR152W	YIM1	8198	8584	0.96	-0.07	-0.18	-0.65
YDR512C		3828	4008	0.96	-0.07	-0.18	-0.65
YIL146C	ECM37	1523	1595	0.95	-0.07	-0.18	-0.65
YLR175W	CBF5	42402	44421	0.95	-0.07	-0.18	-0.65
YGR106C		12707	13313	0.95	-0.07	-0.18	-0.65
YNL181W		8637	9050	0.95	-0.07	-0.18	-0.65
YGL237C	HAP2	4521	4737	0.95	-0.07	-0.18	-0.65
YJL104W		30369	31822	0.95	-0.07	-0.18	-0.65
YFR041C		5187	5435	0.95	-0.07	-0.18	-0.65
YDL052C	SLC1	6298	6600	0.95	-0.07	-0.18	-0.65
YPL067C		3921	4109	0.95	-0.07	-0.18	-0.65
YDR306C		518	543	0.95	-0.07	-0.18	-0.65
YGR172C	YIP1	18782	19685	0.95	-0.07	-0.18	-0.66
YNL052W	COX5A	28491	29864	0.95	-0.07	-0.18	-0.66
YJR118C	ILM1	16079	16856	0.95	-0.07	-0.18	-0.66
YOR115C	TRS33	16410	17205	0.95	-0.07	-0.18	-0.66
YPR037C		6465	6778	0.95	-0.07	-0.18	-0.66

YOL161C		1233	1293	0.95	-0.07	-0.18	-0.66
YDL160C	DHH1	12529	13138	0.95	-0.07	-0.18	-0.66
YFL018W-A		6172	6473	0.95	-0.07	-0.18	-0.66
YJR065C	ARP3	5010	5255	0.95	-0.07	-0.18	-0.66
YOR095C	RKI1	34008	35673	0.95	-0.07	-0.18	-0.66
YOR299W	BUD7	1795	1883	0.95	-0.07	-0.18	-0.66
YGL101W		17702	18571	0.95	-0.07	-0.18	-0.66
YOR283W		7844	8230	0.95	-0.07	-0.18	-0.66
YDR186C		1290	1354	0.95	-0.07	-0.18	-0.66
YDL099W		5630	5907	0.95	-0.07	-0.18	-0.66
YNL335W		1828	1918	0.95	-0.07	-0.18	-0.66
YGR082W	TOM20	25850	27130	0.95	-0.07	-0.18	-0.66
YCR020C-A	MAK31	16667	17494	0.95	-0.07	-0.18	-0.66
YHL031C	GOS1	15729	16514	0.95	-0.07	-0.18	-0.66
YLR237W	THI7	2186	2295	0.95	-0.07	-0.18	-0.66
YDR089W		901	946	0.95	-0.07	-0.18	-0.66
YGL247W		6138	6446	0.95	-0.07	-0.18	-0.67
YIL033C	SRA1	6822	7165	0.95	-0.07	-0.18	-0.67
YNR058W	BIO3	3680	3866	0.95	-0.07	-0.18	-0.67
YPL247C		5954	6255	0.95	-0.07	-0.18	-0.67
YAR044W	OSH1	429	451	0.95	-0.07	-0.18	-0.67
YLR076C		42793	44966	0.95	-0.07	-0.18	-0.67
YML095C	RAD10	3356	3527	0.95	-0.07	-0.18	-0.67
YJL072C		26580	27933	0.95	-0.07	-0.18	-0.67
YNL045W		4965	5218	0.95	-0.07	-0.18	-0.67
YGR198W		7649	8039	0.95	-0.07	-0.18	-0.67
YFR044C		21788	22902	0.95	-0.07	-0.18	-0.67
YOR142W	LSC1	13609	14305	0.95	-0.07	-0.18	-0.67
YNL135C	FPR1	15882	16695	0.95	-0.07	-0.18	-0.67
YGR138C		9673	10169	0.95	-0.07	-0.18	-0.67
YGL143C	MRF1	5312	5586	0.95	-0.07	-0.18	-0.67
YLR157C-A	TyA	11133	11707	0.95	-0.07	-0.18	-0.67
YIR022W	SEC11	16019	16846	0.95	-0.07	-0.19	-0.67
YML057W	CMP2	5460	5742	0.95	-0.07	-0.19	-0.67
YBR254C	TRS20	5758	6056	0.95	-0.07	-0.19	-0.67
YDL144C		5669	5963	0.95	-0.07	-0.19	-0.67

YKL013C	ARC19	16782	17652	0.95	-0.07	-0.19	-0.67
YOL073C		8540	8984	0.95	-0.07	-0.19	-0.67
YML123C	PHO84	44791	47134	0.95	-0.07	-0.19	-0.68
YBL062W		10163	10695	0.95	-0.07	-0.19	-0.68
YMR296C	LCB1	8179	8609	0.95	-0.07	-0.19	-0.68
YLL020C		1065	1121	0.95	-0.07	-0.19	-0.68
YGR278W		2280	2401	0.95	-0.07	-0.19	-0.68
YPL225W		35710	37602	0.95	-0.07	-0.19	-0.68
YDL006W	PTC1	3061	3223	0.95	-0.07	-0.19	-0.68
YCR040W	MATALPHA1	7329	7718	0.95	-0.07	-0.19	-0.68
YGR153W		1191	1254	0.95	-0.07	-0.19	-0.68
YDL095W	PMT1	3936	4145	0.95	-0.07	-0.19	-0.68
YLR211C		25613	26982	0.95	-0.08	-0.19	-0.68
YML027W	YOX1	3390	3572	0.95	-0.08	-0.19	-0.68
YPL064C		12746	13430	0.95	-0.08	-0.19	-0.68
YIL041W		12350	13013	0.95	-0.08	-0.19	-0.68
YDR272W	GLO2	9139	9631	0.95	-0.08	-0.19	-0.68
YKR088C		4074	4294	0.95	-0.08	-0.19	-0.68
YLL050C	COF1	17430	18372	0.95	-0.08	-0.19	-0.68
YOR327C	SNC2	15783	16638	0.95	-0.08	-0.19	-0.69
YMR020W	FMS1	2153	2270	0.95	-0.08	-0.19	-0.69
YBR193C	MED8	6729	7094	0.95	-0.08	-0.19	-0.69
YDR067C		3329	3510	0.95	-0.08	-0.19	-0.69
YCR046C	IMG1	7632	8049	0.95	-0.08	-0.19	-0.69
YNR002C	FUN34	8817	9300	0.95	-0.08	-0.19	-0.69
YJL112W		14247	15029	0.95	-0.08	-0.19	-0.69
YCR012W	PGK1	16443	17345	0.95	-0.08	-0.19	-0.69
YPL224C	MMT2	3184	3359	0.95	-0.08	-0.19	-0.69
YCL050C	APA1	971	1024	0.95	-0.08	-0.19	-0.69
YIL089W		3753	3959	0.95	-0.08	-0.19	-0.69
YIL087C		6063	6398	0.95	-0.08	-0.19	-0.69
YGR276C	RNH70	6846	7225	0.95	-0.08	-0.19	-0.69
YKL105C		729	769	0.95	-0.08	-0.19	-0.69
YLR218C		984	1039	0.95	-0.08	-0.19	-0.69
YCL020W		28853	30461	0.95	-0.08	-0.19	-0.69
YGL259W	YPS5	435	459	0.95	-0.08	-0.19	-0.69

YGR024C		9511	10042	0.95	-0.08	-0.19	-0.69
YMR150C	IMP1	3195	3374	0.95	-0.08	-0.19	-0.69
YDR352W		11191	11819	0.95	-0.08	-0.19	-0.70
YPR073C	LTP1	11688	12347	0.95	-0.08	-0.19	-0.70
YNR026C	SEC12	15597	16476	0.95	-0.08	-0.19	-0.70
YBR219C		7094	7494	0.95	-0.08	-0.19	-0.70
YBR167C	POP7	17267	18242	0.95	-0.08	-0.19	-0.70
YPL100W		751	793	0.95	-0.08	-0.19	-0.70
YLR123C		2370	2505	0.95	-0.08	-0.19	-0.70
YNL265C	IST1	13195	13944	0.95	-0.08	-0.19	-0.70
YBR210W		6462	6829	0.95	-0.08	-0.19	-0.70
YKR084C	HBS1	3501	3701	0.95	-0.08	-0.19	-0.70
YOR088W		2023	2139	0.95	-0.08	-0.19	-0.70
YJL170C	ASG7	801	847	0.95	-0.08	-0.19	-0.70
YBR279W	PAF1	1415	1496	0.95	-0.08	-0.19	-0.70
YBR111C	YSA1	16193	17124	0.95	-0.08	-0.19	-0.70
YER078C		3057	3233	0.95	-0.08	-0.19	-0.70
YOR228C		1336	1413	0.95	-0.08	-0.19	-0.70
YLR105C	SEN2	11579	12252	0.95	-0.08	-0.19	-0.71
YDL066W	IDP1	12067	12770	0.94	-0.08	-0.19	-0.71
YLL056C		1159	1227	0.94	-0.08	-0.19	-0.71
YJL188C		39815	42142	0.94	-0.08	-0.19	-0.71
YDR274C		2144	2269	0.94	-0.08	-0.19	-0.71
YPL070W		5137	5439	0.94	-0.08	-0.19	-0.71
YPL212C	PUS1	13505	14302	0.94	-0.08	-0.20	-0.71
YER028C		2733	2894	0.94	-0.08	-0.20	-0.71
YCL001W	RER1	3930	4164	0.94	-0.08	-0.20	-0.71
YCR077C	PAT1	16593	17582	0.94	-0.08	-0.20	-0.71
YHL021C		12604	13358	0.94	-0.08	-0.20	-0.71
YPL113C		548	581	0.94	-0.08	-0.20	-0.71
YNR024W		15340	16258	0.94	-0.08	-0.20	-0.71
YBL100C		3523	3734	0.94	-0.08	-0.20	-0.71
YJL149W		2712	2875	0.94	-0.08	-0.20	-0.71
YKL060C	FBA1	44294	46963	0.94	-0.08	-0.20	-0.72
YDL046W		24016	25464	0.94	-0.08	-0.20	-0.72
YCR083W	TRX3	4947	5246	0.94	-0.08	-0.20	-0.72

YIR001C	SGN1	7377	7823	0.94	-0.08	-0.20	-0.72
YPL009C		5479	5811	0.94	-0.08	-0.20	-0.72
YPR017C	DSS4	10026	10635	0.94	-0.09	-0.20	-0.72
YBR053C		1959	2078	0.94	-0.09	-0.20	-0.72
YDR246W	TRS23	12976	13765	0.94	-0.09	-0.20	-0.72
YML071C		6770	7182	0.94	-0.09	-0.20	-0.72
YDR163W		6826	7242	0.94	-0.09	-0.20	-0.72
YNL101W		9090	9644	0.94	-0.09	-0.20	-0.72
YAL009W	SPO7	1684	1787	0.94	-0.09	-0.20	-0.72
YPR202W		2375	2520	0.94	-0.09	-0.20	-0.72
YDR061W		3208	3405	0.94	-0.09	-0.20	-0.72
YDL096C		6238	6620	0.94	-0.09	-0.20	-0.72
YKL111C		6843	7265	0.94	-0.09	-0.20	-0.72
YDR231C	COX20	4570	4852	0.94	-0.09	-0.20	-0.72
YBL098W		1286	1366	0.94	-0.09	-0.20	-0.72
YHL050C		28360	30119	0.94	-0.09	-0.20	-0.72
YAR064W		2083	2213	0.94	-0.09	-0.20	-0.73
YIL113W		2014	2139	0.94	-0.09	-0.20	-0.73
YJR022W	LSM8	1710	1817	0.94	-0.09	-0.20	-0.73
YML077W	BET5	6668	7085	0.94	-0.09	-0.20	-0.73
YPL076W	GPI2	8109	8616	0.94	-0.09	-0.20	-0.73
YDR279W		997	1059	0.94	-0.09	-0.20	-0.73
YOR344C	TYE7	4540	4825	0.94	-0.09	-0.20	-0.73
YGL219C		4772	5072	0.94	-0.09	-0.20	-0.73
YDL207W	GLE1	12194	12961	0.94	-0.09	-0.20	-0.73
YML056C		32454	34498	0.94	-0.09	-0.20	-0.73
YDR503C	LPP1	5858	6227	0.94	-0.09	-0.20	-0.73
YPR067W	ISA2	6440	6847	0.94	-0.09	-0.20	-0.73
YGR204W	ADE3	6180	6571	0.94	-0.09	-0.20	-0.73
YMR033W	ARP9	12509	13299	0.94	-0.09	-0.20	-0.73
YNL255C	GIS2	31135	33109	0.94	-0.09	-0.20	-0.73
YMR241W	YHM2	2040	2169	0.94	-0.09	-0.20	-0.73
YML014W		18897	20096	0.94	-0.09	-0.20	-0.73
YOR107W		3144	3344	0.94	-0.09	-0.20	-0.73
YDR073W	SNF11	6427	6836	0.94	-0.09	-0.20	-0.73
YJL017W		1366	1453	0.94	-0.09	-0.20	-0.73

YNL321W		6437	6850	0.94	-0.09	-0.20	-0.73
YDL074C		15240	16217	0.94	-0.09	-0.20	-0.73
YJL068C		12250	13037	0.94	-0.09	-0.20	-0.74
YER093C		4286	4561	0.94	-0.09	-0.20	-0.74
YJR159W	SOR1	1706	1816	0.94	-0.09	-0.20	-0.74
YLL042C	APG10	3403	3624	0.94	-0.09	-0.20	-0.74
YDR188W	CCT6	21289	22675	0.94	-0.09	-0.20	-0.74
YIR036C		4313	4594	0.94	-0.09	-0.20	-0.74
YJL173C	RFA3	10950	11664	0.94	-0.09	-0.20	-0.74
YGR207C		10388	11067	0.94	-0.09	-0.20	-0.74
YNL147W	LSM7	8204	8740	0.94	-0.09	-0.20	-0.74
YGL181W	GTS1	6254	6664	0.94	-0.09	-0.20	-0.74
YFR042W		679	724	0.94	-0.09	-0.20	-0.74
YJL096W	MRPL49	21923	23365	0.94	-0.09	-0.20	-0.74
YGL165C		6407	6829	0.94	-0.09	-0.20	-0.74
YDR532C		5733	6111	0.94	-0.09	-0.20	-0.74
YNR032W	PPG1	11192	11932	0.94	-0.09	-0.20	-0.74
YDR046C	BAP3	1548	1650	0.94	-0.09	-0.20	-0.75
YPR113W	PIS1	21941	23396	0.94	-0.09	-0.21	-0.75
YKL193C	SDS22	5930	6324	0.94	-0.09	-0.21	-0.75
YER130C		6472	6902	0.94	-0.09	-0.21	-0.75
YML088W		3871	4129	0.94	-0.09	-0.21	-0.75
YHR154W	ESC4	7422	7916	0.94	-0.09	-0.21	-0.75
YNL275W		7472	7972	0.94	-0.09	-0.21	-0.75
YJL159W	HSP150	4793	5114	0.94	-0.09	-0.21	-0.75
YGR168C		17597	18778	0.94	-0.09	-0.21	-0.75
YDL241W		4186	4467	0.94	-0.09	-0.21	-0.75
YGL050W		4075	4349	0.94	-0.09	-0.21	-0.75
YPR009W		6087	6497	0.94	-0.09	-0.21	-0.75
YPL139C	UME1	4547	4853	0.94	-0.09	-0.21	-0.75
YER175C		1908	2037	0.94	-0.09	-0.21	-0.75
YLR339C		27937	29820	0.94	-0.09	-0.21	-0.75
YBR109C	CMD1	21564	23019	0.94	-0.09	-0.21	-0.75
YFL060C	SNO3	1619	1728	0.94	-0.09	-0.21	-0.75
YLR205C		43098	46019	0.94	-0.09	-0.21	-0.75
YIL010W	DOT5	5606	5987	0.94	-0.09	-0.21	-0.75

YNL312W	RFA2	8403	8974	0.94	-0.09	-0.21	-0.75
YMR300C	ADE4	6072	6486	0.94	-0.10	-0.21	-0.75
YPL003W	ULA1	3511	3750	0.94	-0.10	-0.21	-0.75
YFL057C		3001	3206	0.94	-0.10	-0.21	-0.76
YNL067W	RPL9B	57651	61598	0.94	-0.10	-0.21	-0.76
YIL011W		10023	10710	0.94	-0.10	-0.21	-0.76
YMR294W-A		11427	12211	0.94	-0.10	-0.21	-0.76
YJL211C		2671	2856	0.94	-0.10	-0.21	-0.76
YNL322C	KRE1	5774	6174	0.94	-0.10	-0.21	-0.76
YPL052W		12457	13325	0.93	-0.10	-0.21	-0.76
YEL011W	GLC3	4649	4974	0.93	-0.10	-0.21	-0.76
YKL151C		2211	2365	0.93	-0.10	-0.21	-0.76
YGR088W	CTT1	3262	3490	0.93	-0.10	-0.21	-0.76
YOR137C		3229	3455	0.93	-0.10	-0.21	-0.76
YER092W		5862	6273	0.93	-0.10	-0.21	-0.76
YNL249C	MPA43	6186	6621	0.93	-0.10	-0.21	-0.77
YPL032C	SVL3	9352	10010	0.93	-0.10	-0.21	-0.77
YPL060W		4801	5140	0.93	-0.10	-0.21	-0.77
YOR326W	MYO2	3523	3772	0.93	-0.10	-0.21	-0.77
YJL202C		5050	5407	0.93	-0.10	-0.21	-0.77
YFL010C		19336	20704	0.93	-0.10	-0.21	-0.77
YMR265C		1211	1297	0.93	-0.10	-0.21	-0.77
YMR170C	ALD2	4137	4430	0.93	-0.10	-0.21	-0.77
YDR069C	DOA4	1770	1896	0.93	-0.10	-0.21	-0.77
YGL031C	RPL24A	31564	33805	0.93	-0.10	-0.21	-0.77
YER093C-A		2924	3132	0.93	-0.10	-0.21	-0.77
YJL115W	ASF1	7259	7776	0.93	-0.10	-0.21	-0.77
YEL074W		1289	1381	0.93	-0.10	-0.21	-0.77
YBR207W	FTH1	19981	21405	0.93	-0.10	-0.21	-0.77
YNL072W	RNH35	7930	8498	0.93	-0.10	-0.21	-0.77
YDR317W		802	860	0.93	-0.10	-0.21	-0.77
YMR306C-A		1593	1707	0.93	-0.10	-0.21	-0.77
YLR466W	YRF1-4	4172	4472	0.93	-0.10	-0.21	-0.77
YKL067W	YNK1	8391	8994	0.93	-0.10	-0.21	-0.77
YIL118W	RHO3	14923	15997	0.93	-0.10	-0.21	-0.77
YIL018W	RPL2B	49240	52786	0.93	-0.10	-0.21	-0.77

YNR043W	MVD1	17753	19033	0.93	-0.10	-0.21	-0.77
YLR137W		6279	6732	0.93	-0.10	-0.21	-0.77
YER076C		4558	4888	0.93	-0.10	-0.21	-0.78
YOR057W	SGT1	4573	4906	0.93	-0.10	-0.21	-0.78
YDR031W		2220	2382	0.93	-0.10	-0.21	-0.78
YDR454C	GUK1	12447	13357	0.93	-0.10	-0.21	-0.78
YGR038W	ORM1	6469	6942	0.93	-0.10	-0.21	-0.78
YLR100W		11934	12809	0.93	-0.10	-0.21	-0.78
YNL310C		4619	4958	0.93	-0.10	-0.21	-0.78
YDR008C		3636	3904	0.93	-0.10	-0.21	-0.78
YML012W	ERV25	13047	14009	0.93	-0.10	-0.22	-0.78
YER002W		24725	26548	0.93	-0.10	-0.22	-0.78
YNR019W	ARE2	10708	11501	0.93	-0.10	-0.22	-0.78
YDL002C	NHP10	13051	14019	0.93	-0.10	-0.22	-0.78
YKR015C		566	608	0.93	-0.10	-0.22	-0.78
YPL215W	CBP3	9069	9743	0.93	-0.10	-0.22	-0.78
YLR133W	CKI1	9347	10042	0.93	-0.10	-0.22	-0.79
YFL035C		3902	4193	0.93	-0.10	-0.22	-0.79
YGR058W		3814	4100	0.93	-0.10	-0.22	-0.79
YBR223C		7141	7678	0.93	-0.10	-0.22	-0.79
YER163C		7188	7729	0.93	-0.10	-0.22	-0.79
YPL015C	HST2	6947	7472	0.93	-0.11	-0.22	-0.79
YBR090C-A		18919	20348	0.93	-0.11	-0.22	-0.79
YFL017C	GNA1	5844	6286	0.93	-0.11	-0.22	-0.79
YNL185C	MRPL19	8145	8764	0.93	-0.11	-0.22	-0.79
YIL049W	DFG10	3933	4232	0.93	-0.11	-0.22	-0.79
YLR172C	DPH5	1001	1077	0.93	-0.11	-0.22	-0.79
YHR164C	DNA2	8824	9498	0.93	-0.11	-0.22	-0.79
YBL022C	PIM1	13441	14468	0.93	-0.11	-0.22	-0.79
YNL026W		22586	24314	0.93	-0.11	-0.22	-0.80
YNR014W		3597	3872	0.93	-0.11	-0.22	-0.80
YML132W	COS3	12144	13075	0.93	-0.11	-0.22	-0.80
YPL058C	PDR12	805	867	0.93	-0.11	-0.22	-0.80
YPL214C	THI6	7675	8264	0.93	-0.11	-0.22	-0.80
YHR218W		18380	19796	0.93	-0.11	-0.22	-0.80
YGR042W		6246	6731	0.93	-0.11	-0.22	-0.80

YGL103W	RPL28	60715	65433	0.93	-0.11	-0.22	-0.80
YOR035C	SHE4	2171	2340	0.93	-0.11	-0.22	-0.80
YBR205W	KTR3	22310	24049	0.93	-0.11	-0.22	-0.80
YKR066C	CCP1	9501	10242	0.93	-0.11	-0.22	-0.80
YDR308C	SRB7	12108	13053	0.93	-0.11	-0.22	-0.80
YDL123W		3777	4073	0.93	-0.11	-0.22	-0.80
YOR352W		1997	2154	0.93	-0.11	-0.22	-0.80
YAL061W		4034	4351	0.93	-0.11	-0.22	-0.81
YIR025W		2625	2831	0.93	-0.11	-0.22	-0.81
YOR149C	SMP3	8336	8992	0.93	-0.11	-0.22	-0.81
YCL066W	HMLALPHA1	9482	10228	0.93	-0.11	-0.22	-0.81
YHR214W-A		3018	3256	0.93	-0.11	-0.22	-0.81
YMR004W	MVP1	1975	2131	0.93	-0.11	-0.22	-0.81
YLR374C		2364	2550	0.93	-0.11	-0.22	-0.81
YDR077W	SED1	11165	12046	0.93	-0.11	-0.22	-0.81
YML074C	NPI46	6312	6810	0.93	-0.11	-0.22	-0.81
YGL154C	LYS5	3047	3288	0.93	-0.11	-0.22	-0.81
YIL155C	GUT2	3863	4169	0.93	-0.11	-0.22	-0.81
YNL333W	SNZ2	8324	8983	0.93	-0.11	-0.22	-0.81
YNL331C	AAD14	13237	14286	0.93	-0.11	-0.22	-0.81
YDL145C	COP1	10470	11300	0.93	-0.11	-0.22	-0.81
YCL032W	STE50	16571	17887	0.93	-0.11	-0.22	-0.81
YOR175C		22314	24088	0.93	-0.11	-0.22	-0.81
YFR020W		3273	3533	0.93	-0.11	-0.22	-0.81
YPR129W	SCD6	16884	18231	0.93	-0.11	-0.22	-0.81
YGL017W	ATE1	9005	9724	0.93	-0.11	-0.22	-0.81
YNL089C		9539	10302	0.93	-0.11	-0.22	-0.81
YOR197W		30683	33137	0.93	-0.11	-0.22	-0.81
YHR053C	CUP1-1	10272	11095	0.93	-0.11	-0.22	-0.81
YBR018C	GAL7	593	641	0.93	-0.11	-0.22	-0.81
YJR015W		3702	4001	0.93	-0.11	-0.22	-0.82
YGR208W	SER2	28429	30726	0.93	-0.11	-0.22	-0.82
YKR076W	ECM4	761	823	0.93	-0.11	-0.22	-0.82
YDR041W		15351	16593	0.93	-0.11	-0.22	-0.82
YGR158C	MTR3	14787	15985	0.93	-0.11	-0.22	-0.82
YBL041W	PRE7	20964	22669	0.92	-0.11	-0.23	-0.82

YOR231W	MKK1	16126	17441	0.92	-0.11	-0.23	-0.82
YOR125C	CAT5	10718	11592	0.92	-0.11	-0.23	-0.82
YIR024C	GIF1	6824	7382	0.92	-0.11	-0.23	-0.82
YGR275W	RTT102	5185	5609	0.92	-0.11	-0.23	-0.82
YLR151C		3650	3949	0.92	-0.11	-0.23	-0.82
YNR074C		730	790	0.92	-0.11	-0.23	-0.82
YIR007W		1394	1509	0.92	-0.11	-0.23	-0.82
YDL200C	MGT1	4100	4437	0.92	-0.11	-0.23	-0.82
YFR027W	ECO1	8984	9727	0.92	-0.11	-0.23	-0.83
YNL309W	STB1	7001	7580	0.92	-0.11	-0.23	-0.83
YDR538W	PAD1	4708	5098	0.92	-0.11	-0.23	-0.83
YLR145W		10720	11609	0.92	-0.11	-0.23	-0.83
YLR006C	SSK1	6161	6674	0.92	-0.12	-0.23	-0.83
YDL092W	SRP14	15698	17005	0.92	-0.12	-0.23	-0.83
YCR062W		859	930	0.92	-0.12	-0.23	-0.83
YMR092C	AIP1	10364	11227	0.92	-0.12	-0.23	-0.83
YHR130C		4813	5215	0.92	-0.12	-0.23	-0.83
YHR086W	NAM8	1270	1376	0.92	-0.12	-0.23	-0.83
YGR266W		11241	12180	0.92	-0.12	-0.23	-0.83
YOR369C	RPS12	41308	44758	0.92	-0.12	-0.23	-0.83
YMR295C		28737	31140	0.92	-0.12	-0.23	-0.83
YGR237C		2201	2386	0.92	-0.12	-0.23	-0.83
YLR300W	EXG1	39664	42999	0.92	-0.12	-0.23	-0.83
YGR102C		10882	11802	0.92	-0.12	-0.23	-0.83
YNL104C	LEU4	24454	26522	0.92	-0.12	-0.23	-0.83
YML055W	SPC2	8086	8770	0.92	-0.12	-0.23	-0.83
YMR110C		8697	9433	0.92	-0.12	-0.23	-0.83
YBL046W		17414	18889	0.92	-0.12	-0.23	-0.84
YOR358W	HAP5	3122	3387	0.92	-0.12	-0.23	-0.84
YOL039W	RPP2A	37725	40924	0.92	-0.12	-0.23	-0.84
YPL059W	GRX5	8658	9392	0.92	-0.12	-0.23	-0.84
YGR074W	SMD1	9866	10705	0.92	-0.12	-0.23	-0.84
YFL066C		25937	28146	0.92	-0.12	-0.23	-0.84
YER071C		2703	2933	0.92	-0.12	-0.23	-0.84
YBR026C	MRF1'	3338	3623	0.92	-0.12	-0.23	-0.84
YGL002W	ERP6	3474	3770	0.92	-0.12	-0.23	-0.84

YML127W		18673	20273	0.92	-0.12	-0.23	-0.84
YLR267W	BOP2	1902	2065	0.92	-0.12	-0.23	-0.84
YKL199C	YKT9	2260	2454	0.92	-0.12	-0.23	-0.84
YNL099C		5224	5673	0.92	-0.12	-0.23	-0.84
YOL133W	HRT1	10257	11140	0.92	-0.12	-0.23	-0.84
YKL167C	MRP49	17196	18677	0.92	-0.12	-0.23	-0.84
YLR189C	UGT51	21280	23114	0.92	-0.12	-0.23	-0.84
YNL171C		1688	1834	0.92	-0.12	-0.23	-0.84
YPL014W		2772	3012	0.92	-0.12	-0.23	-0.84
YHR192W		420	456	0.92	-0.12	-0.23	-0.84
YNL149C		12254	13319	0.92	-0.12	-0.23	-0.85
YDR468C	TLG1	10421	11327	0.92	-0.12	-0.23	-0.85
YML098W	TAF19	11929	12968	0.92	-0.12	-0.23	-0.85
YLR252W		1534	1668	0.92	-0.12	-0.23	-0.85
YJR046W	TAH11	3623	3941	0.92	-0.12	-0.23	-0.85
YCL005W		12729	13848	0.92	-0.12	-0.23	-0.85
YIL028W		2625	2856	0.92	-0.12	-0.23	-0.85
YCR079W		3079	3350	0.92	-0.12	-0.23	-0.85
YPR103W	PRE2	11501	12515	0.92	-0.12	-0.23	-0.85
YDR284C	DPP1	8936	9725	0.92	-0.12	-0.23	-0.85
YIL024C		3571	3886	0.92	-0.12	-0.23	-0.85
YLR281C		1513	1647	0.92	-0.12	-0.23	-0.85
YMR197C	VTI1	8774	9549	0.92	-0.12	-0.23	-0.85
YKL002W		12755	13889	0.92	-0.12	-0.24	-0.86
YNR060W	FRE4	2828	3080	0.92	-0.12	-0.24	-0.86
YIR030C	DCG1	8086	8806	0.92	-0.12	-0.24	-0.86
YML125C		37933	41312	0.92	-0.12	-0.24	-0.86
YHR194W		7427	8089	0.92	-0.12	-0.24	-0.86
YDR238C	SEC26	9837	10714	0.92	-0.12	-0.24	-0.86
YNL336W	COS1	9896	10779	0.92	-0.12	-0.24	-0.86
YJR024C		12141	13227	0.92	-0.12	-0.24	-0.86
YOR357C	GRD19	12152	13239	0.92	-0.12	-0.24	-0.86
YOR208W	PTP2	2193	2389	0.92	-0.12	-0.24	-0.86
YEL052W	AFG1	4396	4790	0.92	-0.12	-0.24	-0.86
YAL044C	GCV3	11579	12616	0.92	-0.12	-0.24	-0.86
YPR133C		13215	14400	0.92	-0.12	-0.24	-0.86

YIL134W	FLX1	2437	2656	0.92	-0.12	-0.24	-0.86
YGL141W		734	800	0.92	-0.12	-0.24	-0.86
YLL064C		2252	2454	0.92	-0.12	-0.24	-0.86
YGR218W	CRM1	12416	13535	0.92	-0.12	-0.24	-0.86
YJR047C	ANB1	5576	6079	0.92	-0.12	-0.24	-0.86
YIR035C		8932	9738	0.92	-0.12	-0.24	-0.86
YOR261C	RPN8	10288	11218	0.92	-0.12	-0.24	-0.86
YIR031C	DAL7	4239	4623	0.92	-0.13	-0.24	-0.86
YKL201C	MNN4	1904	2077	0.92	-0.13	-0.24	-0.86
YER065C	ICL1	7254	7912	0.92	-0.13	-0.24	-0.86
YDR528W		7506	8188	0.92	-0.13	-0.24	-0.86
YDR157W		4581	4997	0.92	-0.13	-0.24	-0.86
YJR105W		9256	10099	0.92	-0.13	-0.24	-0.87
YNL129W		5155	5625	0.92	-0.13	-0.24	-0.87
YBR230C		4749	5182	0.92	-0.13	-0.24	-0.87
YGL104C		1228	1340	0.92	-0.13	-0.24	-0.87
YPR179C		3275	3576	0.92	-0.13	-0.24	-0.87
YBR196C	PGI1	28242	30842	0.92	-0.13	-0.24	-0.87
YDR387C		8201	8956	0.92	-0.13	-0.24	-0.87
YDL130W	RPP1B	42458	46373	0.92	-0.13	-0.24	-0.87
YOR005C	DNL4	901	984	0.92	-0.13	-0.24	-0.87
YLR111W		834	911	0.92	-0.13	-0.24	-0.87
YIR032C	DAL3	11578	12648	0.92	-0.13	-0.24	-0.87
YDL027C		1561	1706	0.92	-0.13	-0.24	-0.87
YDR426C		1802	1969	0.92	-0.13	-0.24	-0.87
YKR039W	GAP1	15431	16864	0.92	-0.13	-0.24	-0.87
YJL151C		13228	14458	0.91	-0.13	-0.24	-0.88
YBR211C	AME1	10282	11239	0.91	-0.13	-0.24	-0.88
YOR271C		8746	9562	0.91	-0.13	-0.24	-0.88
YIL060W		7140	7812	0.91	-0.13	-0.24	-0.88
YLR021W		10087	11037	0.91	-0.13	-0.24	-0.88
YIL175W		5802	6348	0.91	-0.13	-0.24	-0.88
YMR303C	ADH2	38550	42208	0.91	-0.13	-0.24	-0.88
YNL259C	ATX1	12446	13627	0.91	-0.13	-0.24	-0.88
YOR368W	RAD17	2421	2651	0.91	-0.13	-0.24	-0.88
YGR124W	ASN2	31449	34439	0.91	-0.13	-0.24	-0.89

YGR064W		5303	5807	0.91	-0.13	-0.24	-0.89
YNL219C	ALG9	11779	12900	0.91	-0.13	-0.24	-0.89
YDL159W	STE7	8302	9094	0.91	-0.13	-0.24	-0.89
YBR035C	PDX3	11867	13000	0.91	-0.13	-0.24	-0.89
YLR169W		1774	1944	0.91	-0.13	-0.24	-0.89
YJL210W	PEX2	2746	3009	0.91	-0.13	-0.24	-0.89
YDR007W	TRP1	5415	5934	0.91	-0.13	-0.24	-0.89
YJL190C	RPS22A	45956	50374	0.91	-0.13	-0.24	-0.89
YGR078C	PAC10	9329	10232	0.91	-0.13	-0.25	-0.89
YMR222C		2652	2909	0.91	-0.13	-0.25	-0.89
YER036C		24956	27380	0.91	-0.13	-0.25	-0.90
YPL050C	MNN9	31128	34157	0.91	-0.13	-0.25	-0.90
YER112W	LSM4	10793	11844	0.91	-0.13	-0.25	-0.90
YCR025C		871	956	0.91	-0.13	-0.25	-0.90
YKR008W	RSC4	3352	3680	0.91	-0.13	-0.25	-0.90
YBL072C	RPS8A	52318	57453	0.91	-0.14	-0.25	-0.90
YKL051W		6685	7341	0.91	-0.14	-0.25	-0.90
YML087C		3125	3432	0.91	-0.14	-0.25	-0.90
YEL073C		3103	3408	0.91	-0.14	-0.25	-0.90
YLR185W	RPL37A	6551	7195	0.91	-0.14	-0.25	-0.90
YNL243W	SLA2	12912	14187	0.91	-0.14	-0.25	-0.90
YDR115W		8997	9887	0.91	-0.14	-0.25	-0.90
YDL142C	CRD1	3595	3952	0.91	-0.14	-0.25	-0.90
YJL165C	HAL5	674	741	0.91	-0.14	-0.25	-0.91
YNR052C	POP2	16197	17809	0.91	-0.14	-0.25	-0.91
YGR242W		6518	7167	0.91	-0.14	-0.25	-0.91
YLR351C	NIT3	13709	15074	0.91	-0.14	-0.25	-0.91
YLR101C		17735	19508	0.91	-0.14	-0.25	-0.91
YDR414C	ERD1	3154	3471	0.91	-0.14	-0.25	-0.91
YGL198W		14782	16269	0.91	-0.14	-0.25	-0.91
YIL015C-A		1584	1744	0.91	-0.14	-0.25	-0.91
YNL038W		18735	20624	0.91	-0.14	-0.25	-0.91
YLR203C	MSS51	23599	25979	0.91	-0.14	-0.25	-0.91
YDR117C		4830	5317	0.91	-0.14	-0.25	-0.91
YGR236C		823	906	0.91	-0.14	-0.25	-0.91
YDR047W	HEM12	9396	10344	0.91	-0.14	-0.25	-0.91

YDR428C		5416	5963	0.91	-0.14	-0.25	-0.91
YER013W	PRP22	2254	2482	0.91	-0.14	-0.25	-0.91
YGL151W	NUT1	8849	9744	0.91	-0.14	-0.25	-0.91
YHR063C		2351	2590	0.91	-0.14	-0.25	-0.92
YFL038C	YPT1	24772	27287	0.91	-0.14	-0.25	-0.92
YIR011C	STS1	12551	13825	0.91	-0.14	-0.25	-0.92
YKR006C	MRPL13	15050	16578	0.91	-0.14	-0.25	-0.92
YPR075C	OPY2	8059	8883	0.91	-0.14	-0.25	-0.92
YIL076W	SEC28	32320	35651	0.91	-0.14	-0.25	-0.92
YER185W		756	834	0.91	-0.14	-0.25	-0.92
YDR348C		1748	1929	0.91	-0.14	-0.25	-0.92
YMR223W	UBP8	5772	6368	0.91	-0.14	-0.25	-0.92
YOR281C		409	451	0.91	-0.14	-0.25	-0.92
YDR105C		8807	9720	0.91	-0.14	-0.25	-0.93
YKL066W		3550	3919	0.91	-0.14	-0.25	-0.93
YFL039C	ACT1	25284	27911	0.91	-0.14	-0.26	-0.93
YJL101C	GSH1	6927	7647	0.91	-0.14	-0.26	-0.93
YDR103W	STE5	4053	4475	0.91	-0.14	-0.26	-0.93
YDL168W	SFA1	8969	9903	0.91	-0.14	-0.26	-0.93
YKR016W		8416	9292	0.91	-0.14	-0.26	-0.93
YOR277C		13750	15184	0.91	-0.14	-0.26	-0.93
YKR014C	YPT52	4834	5341	0.91	-0.14	-0.26	-0.93
YDL004W	ATP16	13940	15405	0.90	-0.14	-0.26	-0.93
YKL217W	JEN1	565	624	0.90	-0.14	-0.26	-0.93
YAL014C		10502	11607	0.90	-0.14	-0.26	-0.93
YLR108C		4731	5231	0.90	-0.14	-0.26	-0.94
YJL166W	QCR8	27861	30810	0.90	-0.15	-0.26	-0.94
YIL053W	RHR2	29563	32693	0.90	-0.15	-0.26	-0.94
YIR016W		4126	4563	0.90	-0.15	-0.26	-0.94
YDR013W		4027	4455	0.90	-0.15	-0.26	-0.94
YNL211C		3286	3636	0.90	-0.15	-0.26	-0.94
YLR285W		12589	13929	0.90	-0.15	-0.26	-0.94
YIL022W	TIM44	15904	17598	0.90	-0.15	-0.26	-0.94
YDL078C	MDH3	16148	17878	0.90	-0.15	-0.26	-0.94
YIL170W	HXT12	2701	2991	0.90	-0.15	-0.26	-0.94
YOL058W	ARG1	6930	7673	0.90	-0.15	-0.26	-0.94

YER141W	COX15	18484	20467	0.90	-0.15	-0.26	-0.94
YHR152W	SPO12	17083	18917	0.90	-0.15	-0.26	-0.94
YJL121C	RPE1	8332	9227	0.90	-0.15	-0.26	-0.94
YHR114W		36975	40949	0.90	-0.15	-0.26	-0.94
YGR178C	PBP1	20705	22931	0.90	-0.15	-0.26	-0.94
YDL198C	YHM1	15177	16810	0.90	-0.15	-0.26	-0.94
YFL028C	CAF16	9820	10877	0.90	-0.15	-0.26	-0.95
YJR057W	CDC8	6369	7056	0.90	-0.15	-0.26	-0.95
YGR062C	COX18	3419	3788	0.90	-0.15	-0.26	-0.95
YGR192C	TDH3	59047	65427	0.90	-0.15	-0.26	-0.95
YNL173C	MDG1	12448	13798	0.90	-0.15	-0.26	-0.95
YLR195C	NMT1	4915	5451	0.90	-0.15	-0.26	-0.95
YDL181W	INH1	25554	28362	0.90	-0.15	-0.26	-0.96
YNL012W	SPO1	5141	5707	0.90	-0.15	-0.26	-0.96
YIR038C	GTT1	9955	11051	0.90	-0.15	-0.26	-0.96
YPR101W	SNT309	7359	8170	0.90	-0.15	-0.26	-0.96
YKL133C		2275	2526	0.90	-0.15	-0.26	-0.96
YPL066W		13995	15544	0.90	-0.15	-0.26	-0.96
YLR136C	TIS11	3032	3368	0.90	-0.15	-0.26	-0.96
YGR211W	ZPR1	13944	15494	0.90	-0.15	-0.26	-0.96
YBR261C		12197	13553	0.90	-0.15	-0.26	-0.96
YML112W	CTK3	15439	17158	0.90	-0.15	-0.26	-0.96
YGL027C	CWH41	8146	9055	0.90	-0.15	-0.26	-0.96
YHL046C		1528	1699	0.90	-0.15	-0.27	-0.96
YJR039W		347	386	0.90	-0.15	-0.27	-0.96
YLL014W		9170	10197	0.90	-0.15	-0.27	-0.97
YFR051C	RET2	9077	10094	0.90	-0.15	-0.27	-0.97
YDR511W		3921	4360	0.90	-0.15	-0.27	-0.97
YKR048C	NAP1	4679	5204	0.90	-0.15	-0.27	-0.97
YDR254W	CHL4	6262	6965	0.90	-0.15	-0.27	-0.97
YFL048C	EMP47	8863	9857	0.90	-0.15	-0.27	-0.97
YJL078C	PRY3	21300	23693	0.90	-0.15	-0.27	-0.97
YNL200C		7714	8583	0.90	-0.15	-0.27	-0.97
YGL023C	PIB2	7415	8250	0.90	-0.15	-0.27	-0.97
YJL172W	CPS1	4839	5386	0.90	-0.15	-0.27	-0.97
YDL114W		446	496	0.90	-0.15	-0.27	-0.97

YKL155C		3249	3618	0.90	-0.15	-0.27	-0.97
YML110C	COQ5	27290	30396	0.90	-0.16	-0.27	-0.97
YPR098C		5865	6534	0.90	-0.16	-0.27	-0.98
YOR015W		2213	2466	0.90	-0.16	-0.27	-0.98
YDL068W		2124	2367	0.90	-0.16	-0.27	-0.98
YNR037C		5422	6041	0.90	-0.16	-0.27	-0.98
YER145C	FTR1	18651	20781	0.90	-0.16	-0.27	-0.98
YDR121W	DPB4	12226	13623	0.90	-0.16	-0.27	-0.98
YML013C-A		3370	3755	0.90	-0.16	-0.27	-0.98
YLR142W	PUT1	4934	5498	0.90	-0.16	-0.27	-0.98
YPL048W	CAM1	22510	25087	0.90	-0.16	-0.27	-0.98
YDR320C		3862	4305	0.90	-0.16	-0.27	-0.98
YBR164C	ARL1	13699	15271	0.90	-0.16	-0.27	-0.98
YKL018W		8779	9787	0.90	-0.16	-0.27	-0.98
YLR173W		8860	9877	0.90	-0.16	-0.27	-0.98
YMR041C		5850	6522	0.90	-0.16	-0.27	-0.98
YOR059C		2848	3175	0.90	-0.16	-0.27	-0.98
YLR299W	ECM38	5165	5759	0.90	-0.16	-0.27	-0.98
YBR256C	RIB5	11627	12967	0.90	-0.16	-0.27	-0.98
YPR173C	VPS4	17547	19571	0.90	-0.16	-0.27	-0.98
YJR104C	SOD1	28350	31627	0.90	-0.16	-0.27	-0.98
YLR140W		8611	9607	0.90	-0.16	-0.27	-0.98
YKL224C		1563	1744	0.90	-0.16	-0.27	-0.98
YGL109W		8235	9192	0.90	-0.16	-0.27	-0.99
YML007W	YAP1	22690	25329	0.90	-0.16	-0.27	-0.99
YER081W	SER3	2790	3116	0.90	-0.16	-0.27	-0.99
YKR018C		12170	13596	0.90	-0.16	-0.27	-0.99
YER120W	SCS2	22172	24777	0.89	-0.16	-0.27	-0.99
YNL326C		2793	3121	0.89	-0.16	-0.27	-0.99
YMR221C		3672	4105	0.89	-0.16	-0.27	-0.99
YER178W	PDA1	21388	23917	0.89	-0.16	-0.27	-1.00
YER057C	HIG1	13468	15063	0.89	-0.16	-0.27	-1.00
YAL065C		3662	4096	0.89	-0.16	-0.27	-1.00
YJL097W		13532	15140	0.89	-0.16	-0.27	-1.00
YLR423C		1691	1892	0.89	-0.16	-0.27	-1.00
YML058C-A		3258	3645	0.89	-0.16	-0.27	-1.00

YGL200C	EMP24	31733	35507	0.89	-0.16	-0.27	-1.00
YHR013C	ARD1	11319	12667	0.89	-0.16	-0.27	-1.00
YKL202W		1021	1143	0.89	-0.16	-0.27	-1.00
YOR148C	SPP2	6321	7077	0.89	-0.16	-0.28	-1.00
YLR348C	DIC1	9918	11108	0.89	-0.16	-0.28	-1.00
YDR169C	STB3	6969	7807	0.89	-0.16	-0.28	-1.00
YBR137W		8375	9382	0.89	-0.16	-0.28	-1.00
YJL064W		15634	17517	0.89	-0.16	-0.28	-1.01
YGL091C	NBP35	10674	11961	0.89	-0.16	-0.28	-1.01
YGL041C		14217	15934	0.89	-0.16	-0.28	-1.01
YOR161C		5487	6151	0.89	-0.16	-0.28	-1.01
YOR102W		11199	12555	0.89	-0.16	-0.28	-1.01
YJL164C	SRA3	8321	9329	0.89	-0.16	-0.28	-1.01
YDR293C	SSD1	1583	1775	0.89	-0.17	-0.28	-1.01
YNL323W		16785	18820	0.89	-0.17	-0.28	-1.01
YPR170C		5580	6259	0.89	-0.17	-0.28	-1.01
YDR027C	LUV1	1509	1693	0.89	-0.17	-0.28	-1.01
YIL072W	HOP1	2134	2394	0.89	-0.17	-0.28	-1.01
YGL189C	RPS26A	34077	38233	0.89	-0.17	-0.28	-1.01
YNR034W	SOL1	8756	9824	0.89	-0.17	-0.28	-1.01
YOR301W	RAX1	2202	2471	0.89	-0.17	-0.28	-1.01
YKL084W		7643	8576	0.89	-0.17	-0.28	-1.01
YLR107W		10198	11443	0.89	-0.17	-0.28	-1.01
YBR285W		1928	2163	0.89	-0.17	-0.28	-1.01
YER161C	SPT2	5701	6399	0.89	-0.17	-0.28	-1.01
YOL085C		828	930	0.89	-0.17	-0.28	-1.02
YJL088W	ARG3	6383	7166	0.89	-0.17	-0.28	-1.02
YMR324C		2626	2949	0.89	-0.17	-0.28	-1.02
YML029W		1867	2097	0.89	-0.17	-0.28	-1.02
YGR155W	CYS4	26031	29236	0.89	-0.17	-0.28	-1.02
YBR120C	CBP6	7234	8126	0.89	-0.17	-0.28	-1.02
YOL149W	DCP1	14234	15991	0.89	-0.17	-0.28	-1.02
YAL003W	EFB1	49176	55248	0.89	-0.17	-0.28	-1.02
YKL119C	VPH2	10517	11818	0.89	-0.17	-0.28	-1.02
YFR024C		13144	14772	0.89	-0.17	-0.28	-1.02
YKL076C		8284	9315	0.89	-0.17	-0.28	-1.02

YDR079W	PET100	3610	4060	0.89	-0.17	-0.28	-1.02
YNR030W	ECM39	16395	18443	0.89	-0.17	-0.28	-1.03
YLR292C	SEC72	17664	19872	0.89	-0.17	-0.28	-1.03
YKL169C		15993	17993	0.89	-0.17	-0.28	-1.03
YPL124W	NIP29	11591	13041	0.89	-0.17	-0.28	-1.03
YJL142C		6977	7851	0.89	-0.17	-0.28	-1.03
YER050C		3032	3412	0.89	-0.17	-0.28	-1.03
YOL071W		15739	17711	0.89	-0.17	-0.28	-1.03
YER177W	BMH1	32801	36916	0.89	-0.17	-0.28	-1.03
YOR133W	EFT1	18843	21209	0.89	-0.17	-0.28	-1.03
YOR176W	HEM15	7497	8439	0.89	-0.17	-0.28	-1.03
YNR050C	LYS9	26578	29926	0.89	-0.17	-0.28	-1.03
YEL037C	RAD23	5456	6144	0.89	-0.17	-0.28	-1.03
YCLX09W		7184	8090	0.89	-0.17	-0.28	-1.03
YBR293W		14640	16488	0.89	-0.17	-0.28	-1.03
YLR066W	SPC3	12120	13651	0.89	-0.17	-0.28	-1.03
YBR217W	APG12	8543	9624	0.89	-0.17	-0.28	-1.03
YPR176C	BET2	4442	5004	0.89	-0.17	-0.28	-1.03
YHR009C		6581	7417	0.89	-0.17	-0.28	-1.04
YOR111W		13665	15402	0.89	-0.17	-0.29	-1.04
YNL011C		2618	2951	0.89	-0.17	-0.29	-1.04
YNL239W	LAP3	10556	11900	0.89	-0.17	-0.29	-1.04
YOR075W	UFE1	11349	12795	0.89	-0.17	-0.29	-1.04
YNL192W	CHS1	9481	10689	0.89	-0.17	-0.29	-1.04
YLL053C		5234	5902	0.89	-0.17	-0.29	-1.04
YBR030W		1310	1477	0.89	-0.17	-0.29	-1.04
YAL020C	ATS1	5162	5822	0.89	-0.17	-0.29	-1.04
YLR379W		6180	6971	0.89	-0.17	-0.29	-1.04
YCR029C-A		25173	28393	0.89	-0.17	-0.29	-1.04
YNL079C	TPM1	16354	18447	0.89	-0.17	-0.29	-1.04
YDR286C		7969	8989	0.89	-0.17	-0.29	-1.04
YIR019C	MUC1	373	421	0.89	-0.17	-0.29	-1.04
YER179W	DMC1	1859	2098	0.89	-0.17	-0.29	-1.04
YDR288W		12688	14319	0.89	-0.17	-0.29	-1.04
YDR063W		4995	5638	0.89	-0.17	-0.29	-1.04
YNL251C	NRD1	14798	16704	0.89	-0.17	-0.29	-1.04

YOL008W		3139	3543	0.89	-0.17	-0.29	-1.04
YDL020C	RPN4	8163	9214	0.89	-0.17	-0.29	-1.04
YPR023C		9128	10304	0.89	-0.17	-0.29	-1.04
YGR063C	SPT4	9862	11133	0.89	-0.17	-0.29	-1.04
YPR174C		1844	2082	0.89	-0.18	-0.29	-1.05
YNL327W	EGT2	25217	28470	0.89	-0.18	-0.29	-1.05
YLR416C		2118	2391	0.89	-0.18	-0.29	-1.05
YHL030W	ECM29	3103	3504	0.89	-0.18	-0.29	-1.05
YPR193C	HPA2	1705	1926	0.89	-0.18	-0.29	-1.05
YAR023C		2610	2949	0.88	-0.18	-0.29	-1.05
YKR010C	TOF2	1357	1534	0.88	-0.18	-0.29	-1.05
YGL121C		4912	5551	0.88	-0.18	-0.29	-1.05
YHR166C	CDC23	4411	4986	0.88	-0.18	-0.29	-1.05
YDR256C	CTA1	2303	2604	0.88	-0.18	-0.29	-1.05
YDL233W		3664	4144	0.88	-0.18	-0.29	-1.05
YNL276C		2487	2813	0.88	-0.18	-0.29	-1.06
YPL037C	EGD1	35765	40459	0.88	-0.18	-0.29	-1.06
YMR253C		3575	4046	0.88	-0.18	-0.29	-1.06
YEL040W	UTR2	13039	14759	0.88	-0.18	-0.29	-1.06
YIL007C		7634	8642	0.88	-0.18	-0.29	-1.06
YDR173C	ARG82	5234	5927	0.88	-0.18	-0.29	-1.06
YFR025C	HIS2	19188	21728	0.88	-0.18	-0.29	-1.06
YOR109W	INP53	8451	9570	0.88	-0.18	-0.29	-1.06
YGL261C		2301	2607	0.88	-0.18	-0.29	-1.06
YKR100C		2437	2762	0.88	-0.18	-0.29	-1.06
YJR099W	YUH1	6349	7194	0.88	-0.18	-0.29	-1.06
YMR267W	PPA2	2556	2897	0.88	-0.18	-0.29	-1.06
YGR295C	COS6	21631	24523	0.88	-0.18	-0.29	-1.07
YLR075W	RPL10	53117	60231	0.88	-0.18	-0.29	-1.07
YLR053C		2763	3133	0.88	-0.18	-0.29	-1.07
YBR295W	PCA1	6991	7929	0.88	-0.18	-0.29	-1.07
YJL161W		1137	1290	0.88	-0.18	-0.29	-1.07
YGL048C	RPT6	14186	16091	0.88	-0.18	-0.29	-1.07
YDR100W		17064	19363	0.88	-0.18	-0.29	-1.07
YOL064C	MET22	18658	21180	0.88	-0.18	-0.30	-1.07
YER080W		10694	12141	0.88	-0.18	-0.30	-1.07

YJL174W	KRE9	6774	7692	0.88	-0.18	-0.30	-1.08
YBR197C		9817	11149	0.88	-0.18	-0.30	-1.08
YJL106W	IME2	1508	1713	0.88	-0.18	-0.30	-1.08
YJL140W	RPB4	26411	30006	0.88	-0.18	-0.30	-1.08
YBR056W		591	671	0.88	-0.18	-0.30	-1.08
YKR034W	DAL80	2424	2754	0.88	-0.18	-0.30	-1.08
YER017C	AFG3	5485	6232	0.88	-0.18	-0.30	-1.08
YDL098C	SNU23	10269	11674	0.88	-0.18	-0.30	-1.08
YFL020C	PAU5	1832	2083	0.88	-0.19	-0.30	-1.08
YBR080C	SEC18	4659	5298	0.88	-0.19	-0.30	-1.08
YKL003C	MRP17	10362	11784	0.88	-0.19	-0.30	-1.08
YDR232W	HEM1	17802	20245	0.88	-0.19	-0.30	-1.08
YGR146C		14466	16454	0.88	-0.19	-0.30	-1.08
YOR181W	LAS17	2846	3240	0.88	-0.19	-0.30	-1.09
YDR129C	SAC6	15150	17248	0.88	-0.19	-0.30	-1.09
YML083C		3039	3460	0.88	-0.19	-0.30	-1.09
YLR428C		6075	6917	0.88	-0.19	-0.30	-1.09
YMR056C	AAC1	2335	2659	0.88	-0.19	-0.30	-1.09
YGR175C	ERG1	23737	27038	0.88	-0.19	-0.30	-1.09
YGR032W	GSC2	2159	2459	0.88	-0.19	-0.30	-1.09
YNL043C		8402	9572	0.88	-0.19	-0.30	-1.09
YKL159C	RCN1	2921	3330	0.88	-0.19	-0.30	-1.10
YGL179C		2178	2483	0.88	-0.19	-0.30	-1.10
YKL061W		2246	2561	0.88	-0.19	-0.30	-1.10
YDR510W	SMT3	20980	23922	0.88	-0.19	-0.30	-1.10
YDL086W		14745	16817	0.88	-0.19	-0.30	-1.10
YJR016C	ILV3	21506	24530	0.88	-0.19	-0.30	-1.10
YOR045W	TOM6	20422	23295	0.88	-0.19	-0.30	-1.10
YDL008W	APC11	5193	5925	0.88	-0.19	-0.30	-1.10
YPL246C		13538	15445	0.88	-0.19	-0.30	-1.10
YOL143C	RIB4	7649	8727	0.88	-0.19	-0.30	-1.10
YPL144W	SNR17B	14258	16269	0.88	-0.19	-0.30	-1.10
YNL008C		10495	11977	0.88	-0.19	-0.30	-1.10
YAR028W		20452	23341	0.88	-0.19	-0.30	-1.10
YGL072C		865	988	0.88	-0.19	-0.30	-1.10
YOR067C	ALG8	5243	5986	0.88	-0.19	-0.30	-1.10

YMR090W		4355	4973	0.88	-0.19	-0.30	-1.10
YKL123W		3453	3943	0.88	-0.19	-0.30	-1.10
YLR159W		1498	1711	0.88	-0.19	-0.30	-1.11
YPL071C		4474	5110	0.88	-0.19	-0.30	-1.11
YHR099W	TRA1	28189	32198	0.88	-0.19	-0.30	-1.11
YML067C		23956	27367	0.88	-0.19	-0.30	-1.11
YER102W	RPS8B	57249	65406	0.88	-0.19	-0.30	-1.11
YLR408C		1304	1490	0.88	-0.19	-0.30	-1.11
YPL107W		2451	2801	0.88	-0.19	-0.30	-1.11
YJR059W	PTK2	3753	4289	0.87	-0.19	-0.31	-1.11
YPR151C		2595	2966	0.87	-0.19	-0.31	-1.11
YDR252W	BTT1	6321	7227	0.87	-0.19	-0.31	-1.11
YBR071W		14869	17003	0.87	-0.19	-0.31	-1.11
YER004W		13308	15220	0.87	-0.19	-0.31	-1.11
YPL148C	PPT2	10474	11983	0.87	-0.19	-0.31	-1.11
YBL101W-A		17494	20015	0.87	-0.19	-0.31	-1.11
YPR079W		4236	4848	0.87	-0.19	-0.31	-1.12
YOL119C		2919	3340	0.87	-0.19	-0.31	-1.12
YJR004C	SAG1	6856	7847	0.87	-0.19	-0.31	-1.12
YLR031W		694	795	0.87	-0.20	-0.31	-1.12
YBL006C		18571	21262	0.87	-0.20	-0.31	-1.12
YGR294W		902	1033	0.87	-0.20	-0.31	-1.12
YHR118C	ORC6	2537	2905	0.87	-0.20	-0.31	-1.12
YNL156C		24241	27755	0.87	-0.20	-0.31	-1.12
YLR199C		15437	17677	0.87	-0.20	-0.31	-1.12
YDL195W	SEC31	15277	17499	0.87	-0.20	-0.31	-1.12
YLR037C		4603	5273	0.87	-0.20	-0.31	-1.12
YKL171W		1821	2087	0.87	-0.20	-0.31	-1.12
YIL154C	IMP2'	4048	4640	0.87	-0.20	-0.31	-1.12
YDR040C	ENA1	3394	3891	0.87	-0.20	-0.31	-1.12
YHR106W	TRR2	12555	14395	0.87	-0.20	-0.31	-1.13
YGR049W	SCM4	6966	7988	0.87	-0.20	-0.31	-1.13
YGL039W		9249	10607	0.87	-0.20	-0.31	-1.13
YMR002W		31837	36513	0.87	-0.20	-0.31	-1.13
YDR410C	STE14	7349	8432	0.87	-0.20	-0.31	-1.13
YGR136W		31549	36199	0.87	-0.20	-0.31	-1.13

YOL099C		1496	1716	0.87	-0.20	-0.31	-1.13
YGL065C	ALG2	2247	2579	0.87	-0.20	-0.31	-1.13
YIL044C		31542	36205	0.87	-0.20	-0.31	-1.13
YJL001W	PRE3	14764	16955	0.87	-0.20	-0.31	-1.13
YNL003C	PET8	5754	6608	0.87	-0.20	-0.31	-1.13
YPL152W	RRD2	9882	11351	0.87	-0.20	-0.31	-1.14
YML102W	CAC2	12097	13895	0.87	-0.20	-0.31	-1.14
YOR079C	ATX2	11021	12660	0.87	-0.20	-0.31	-1.14
YMR180C	CTL1	5243	6023	0.87	-0.20	-0.31	-1.14
YMR255W	GFD1	19887	22847	0.87	-0.20	-0.31	-1.14
YML116W	ATR1	17468	20073	0.87	-0.20	-0.31	-1.14
YKR001C	VPS1	2619	3010	0.87	-0.20	-0.31	-1.14
YOR150W	MRPL23	11366	13062	0.87	-0.20	-0.31	-1.14
YLR161W		30595	35163	0.87	-0.20	-0.31	-1.14
YPR203W		5106	5869	0.87	-0.20	-0.31	-1.14
YBL074C	AAR2	1627	1870	0.87	-0.20	-0.31	-1.14
YAL024C	LTE1	447	514	0.87	-0.20	-0.31	-1.14
YML131W		15698	18054	0.87	-0.20	-0.31	-1.14
YGL161C		16123	18544	0.87	-0.20	-0.31	-1.14
YKR065C		8761	10078	0.87	-0.20	-0.31	-1.14
YDR086C	SSS1	28700	33031	0.87	-0.20	-0.32	-1.15
YHR080C		19783	22772	0.87	-0.20	-0.32	-1.15
YIL048W	NEO1	5184	5970	0.87	-0.20	-0.32	-1.15
YJR010W	MET3	2046	2358	0.87	-0.20	-0.32	-1.15
YGL051W		9300	10719	0.87	-0.20	-0.32	-1.15
YDL192W	ARF1	31843	36724	0.87	-0.21	-0.32	-1.16
YJL180C	ATP12	19437	22417	0.87	-0.21	-0.32	-1.16
YKL052C		9069	10460	0.87	-0.21	-0.32	-1.16
YDL076C		10752	12409	0.87	-0.21	-0.32	-1.16
YDL175C		12556	14495	0.87	-0.21	-0.32	-1.16
YNL263C	YIF1	18174	20980	0.87	-0.21	-0.32	-1.16
YNR042W		11144	12867	0.87	-0.21	-0.32	-1.16
YAL016W	TPD3	24210	27959	0.87	-0.21	-0.32	-1.16
YIL117C		8757	10115	0.87	-0.21	-0.32	-1.16
YGR122W		10280	11874	0.87	-0.21	-0.32	-1.16
YGL095C	VPS45	8124	9389	0.87	-0.21	-0.32	-1.17

YER119C		3620	4184	0.87	-0.21	-0.32	-1.17
YDL134C	PPH21	22271	25752	0.86	-0.21	-0.32	-1.17
YLR165C		21800	25212	0.86	-0.21	-0.32	-1.17
YEL059W		621	718	0.86	-0.21	-0.32	-1.17
YKL161C		6204	7177	0.86	-0.21	-0.32	-1.17
YCR070W		8049	9314	0.86	-0.21	-0.32	-1.17
YNL267W	PIK1	10485	12133	0.86	-0.21	-0.32	-1.17
YFR018C		3464	4011	0.86	-0.21	-0.32	-1.18
YKL040C	NFU1	9050	10485	0.86	-0.21	-0.32	-1.18
YER009W	NTF2	33090	38361	0.86	-0.21	-0.33	-1.18
YBR052C		563	653	0.86	-0.21	-0.33	-1.18
YDR051C		2816	3265	0.86	-0.21	-0.33	-1.18
YPL061W	ALD6	35430	41082	0.86	-0.21	-0.33	-1.19
YCLX05C		5628	6528	0.86	-0.21	-0.33	-1.19
YJL152W		11422	13248	0.86	-0.21	-0.33	-1.19
YML130C	ERO1	4581	5314	0.86	-0.21	-0.33	-1.19
YOR184W	SER1	11099	12877	0.86	-0.21	-0.33	-1.19
YHR168W		2746	3187	0.86	-0.21	-0.33	-1.19
YBR231C		8190	9504	0.86	-0.21	-0.33	-1.19
YLR064W		9572	11115	0.86	-0.22	-0.33	-1.19
YLR050C		6813	7913	0.86	-0.22	-0.33	-1.19
YFR030W	MET10	4522	5253	0.86	-0.22	-0.33	-1.19
YGR154C		6437	7478	0.86	-0.22	-0.33	-1.19
YML036W		18366	21337	0.86	-0.22	-0.33	-1.20
YDL081C	RPP1A	33233	38617	0.86	-0.22	-0.33	-1.20
YGR046W		3364	3910	0.86	-0.22	-0.33	-1.20
YNL159C		2521	2931	0.86	-0.22	-0.33	-1.20
YEL058W	PCM1	11129	12944	0.86	-0.22	-0.33	-1.20
YAR033W		4319	5028	0.86	-0.22	-0.33	-1.21
YML030W		21744	25317	0.86	-0.22	-0.33	-1.21
YDL120W	YFH1	20521	23893	0.86	-0.22	-0.33	-1.21
YJL143W	TIM17	10551	12285	0.86	-0.22	-0.33	-1.21
YBL068W	PRS4	45907	53458	0.86	-0.22	-0.33	-1.21
YER142C	MAG1	3584	4174	0.86	-0.22	-0.33	-1.21
YDR236C	FMN1	4764	5550	0.86	-0.22	-0.33	-1.21
YOR071C		1584	1846	0.86	-0.22	-0.33	-1.21

YCL028W	RNQ1	17169	20004	0.86	-0.22	-0.33	-1.21
YPR167C	MET16	729	849	0.86	-0.22	-0.33	-1.21
YGL079W		12701	14802	0.86	-0.22	-0.33	-1.21
YMR275C	BUL1	2249	2621	0.86	-0.22	-0.33	-1.21
YNL215W		4431	5164	0.86	-0.22	-0.33	-1.21
YOL110W	SHR5	3073	3582	0.86	-0.22	-0.33	-1.21
YJL154C	VPS35	3591	4186	0.86	-0.22	-0.33	-1.21
YHR123W	EPT1	2623	3059	0.86	-0.22	-0.33	-1.21
YER038C		3891	4539	0.86	-0.22	-0.33	-1.22
YPL188W	POS5	15251	17793	0.86	-0.22	-0.33	-1.22
YPL084W	BRO1	12950	15109	0.86	-0.22	-0.33	-1.22
YEL038W	UTR4	24153	28180	0.86	-0.22	-0.33	-1.22
YDR204W	COQ4	5450	6360	0.86	-0.22	-0.34	-1.22
YKL157W	APE2	5840	6816	0.86	-0.22	-0.34	-1.22
YMR009W		12271	14323	0.86	-0.22	-0.34	-1.22
YLR155C	ASP3-1	9221	10765	0.86	-0.22	-0.34	-1.22
YEL007W		10190	11900	0.86	-0.22	-0.34	-1.22
YOR213C	SAS5	13008	15196	0.86	-0.22	-0.34	-1.22
YBR199W	KTR4	29233	34152	0.86	-0.22	-0.34	-1.22
YDL185W	TFP1	36685	42865	0.86	-0.22	-0.34	-1.23
YOR159C	SME1	850	994	0.86	-0.23	-0.34	-1.23
YGL089C	MF(ALPHA)2	11722	13705	0.86	-0.23	-0.34	-1.23
YPR062W	FCY1	26051	30461	0.86	-0.23	-0.34	-1.23
YGR206W		8563	10014	0.86	-0.23	-0.34	-1.23
YIL111W	COX5B	8339	9752	0.86	-0.23	-0.34	-1.23
YGR222W	PET54	15643	18302	0.85	-0.23	-0.34	-1.23
YGR118W	RPS23A	45010	52672	0.85	-0.23	-0.34	-1.23
YHR141C	RPL42B	3539	4142	0.85	-0.23	-0.34	-1.23
YBR160W	CDC28	29254	34236	0.85	-0.23	-0.34	-1.23
YPR108W	RPN7	25192	29491	0.85	-0.23	-0.34	-1.24
YPL046C	ELC1	5897	6904	0.85	-0.23	-0.34	-1.24
YBR070C		6180	7236	0.85	-0.23	-0.34	-1.24
YMR271C	URA10	5952	6970	0.85	-0.23	-0.34	-1.24
YBR214W	SDS24	1945	2278	0.85	-0.23	-0.34	-1.24
YJL184W		19017	22274	0.85	-0.23	-0.34	-1.24
YLR187W		33192	38890	0.85	-0.23	-0.34	-1.24

YER091C	MET6	14833	17379	0.85	-0.23	-0.34	-1.24
YDR350C	TCM10	2982	3494	0.85	-0.23	-0.34	-1.24
YGR050C		2284	2677	0.85	-0.23	-0.34	-1.24
YJR123W	RPS5	31777	37241	0.85	-0.23	-0.34	-1.24
YNL237W	YTP1	2826	3312	0.85	-0.23	-0.34	-1.24
YPL034W		9303	10904	0.85	-0.23	-0.34	-1.24
YCL033C		3836	4498	0.85	-0.23	-0.34	-1.24
YDR318W	MCM21	8515	9987	0.85	-0.23	-0.34	-1.24
YER068W	MOT2	6504	7629	0.85	-0.23	-0.34	-1.25
YAL053W		1218	1429	0.85	-0.23	-0.34	-1.25
YNL069C	RPL16B	28242	33164	0.85	-0.23	-0.34	-1.25
YLR209C		12641	14847	0.85	-0.23	-0.34	-1.25
YFL064C		2757	3239	0.85	-0.23	-0.34	-1.25
YKL100C		5219	6132	0.85	-0.23	-0.34	-1.25
YPR145W	ASN1	54504	64044	0.85	-0.23	-0.35	-1.25
YDR072C	IPT1	14720	17311	0.85	-0.23	-0.35	-1.26
YML022W	APT1	41643	48991	0.85	-0.23	-0.35	-1.26
YOR324C		2684	3158	0.85	-0.23	-0.35	-1.26
YMR318C		37918	44638	0.85	-0.24	-0.35	-1.26
YCR009C	RVS161	14729	17346	0.85	-0.24	-0.35	-1.27
YOR198C	BFR1	11266	13268	0.85	-0.24	-0.35	-1.27
YDL125C	HNT1	13184	15527	0.85	-0.24	-0.35	-1.27
YOR152C		372	438	0.85	-0.24	-0.35	-1.27
YPR054W	SMK1	2815	3316	0.85	-0.24	-0.35	-1.27
YJL100W		6418	7564	0.85	-0.24	-0.35	-1.27
YPL204W	HRR25	29360	34609	0.85	-0.24	-0.35	-1.27
YJL080C	SCP160	18347	21629	0.85	-0.24	-0.35	-1.27
YNL253W		10535	12421	0.85	-0.24	-0.35	-1.27
YGR037C	ACB1	19438	22928	0.85	-0.24	-0.35	-1.27
YNL307C	MCK1	30667	36183	0.85	-0.24	-0.35	-1.28
YLR110C		25870	30535	0.85	-0.24	-0.35	-1.28
YLR324W		3949	4662	0.85	-0.24	-0.35	-1.28
YGR250C		11565	13653	0.85	-0.24	-0.35	-1.28
YDL132W	CDC53	6744	7963	0.85	-0.24	-0.35	-1.28
YLR059C	YNT20	15768	18621	0.85	-0.24	-0.35	-1.28
YLR119W	SRN2	6921	8174	0.85	-0.24	-0.35	-1.28

YLR025W	SNF7	11435	13505	0.85	-0.24	-0.35	-1.28
YIL140W	SRO4	8998	10627	0.85	-0.24	-0.35	-1.28
YLR332W	MID2	9912	11711	0.85	-0.24	-0.35	-1.28
YML001W	YPT7	26332	31144	0.85	-0.24	-0.35	-1.29
YKL073W	LHS1	1382	1636	0.84	-0.24	-0.36	-1.29
YGR253C	PUP2	14262	16884	0.84	-0.24	-0.36	-1.29
YJL062W	LAS21	14489	17153	0.84	-0.24	-0.36	-1.29
YER020W	GPA2	2373	2810	0.84	-0.24	-0.36	-1.29
YGL013C	PDR1	9624	11396	0.84	-0.24	-0.36	-1.30
YBR175W		26204	31033	0.84	-0.24	-0.36	-1.30
YMR250W		1550	1836	0.84	-0.24	-0.36	-1.30
YAL045C		19479	23077	0.84	-0.24	-0.36	-1.30
YBR263W	SHM1	23396	27725	0.84	-0.24	-0.36	-1.30
YJL124C	LSM1	31396	37210	0.84	-0.25	-0.36	-1.30
YML101C		7768	9212	0.84	-0.25	-0.36	-1.30
YDR085C	AFR1	1773	2103	0.84	-0.25	-0.36	-1.30
YDL157C		15598	18502	0.84	-0.25	-0.36	-1.30
YCR027C	RSG1	9461	11224	0.84	-0.25	-0.36	-1.30
YBL094C		857	1017	0.84	-0.25	-0.36	-1.31
YOR163W		4757	5645	0.84	-0.25	-0.36	-1.31
YIL062C	ARC15	30578	36286	0.84	-0.25	-0.36	-1.31
YOR219C	STE13	6992	8298	0.84	-0.25	-0.36	-1.31
YIL136W	OM45	2770	3287	0.84	-0.25	-0.36	-1.31
YKL153W		29828	35400	0.84	-0.25	-0.36	-1.31
YGL010W		2012	2389	0.84	-0.25	-0.36	-1.31
YOL162W		783	930	0.84	-0.25	-0.36	-1.31
YMR022W	QRI8	8994	10678	0.84	-0.25	-0.36	-1.31
YGR258C	RAD2	3959	4702	0.84	-0.25	-0.36	-1.31
YMR199W	CLN1	6701	7959	0.84	-0.25	-0.36	-1.31
YER044C		33953	40352	0.84	-0.25	-0.36	-1.31
YPL013C		17643	20975	0.84	-0.25	-0.36	-1.32
YKL086W		6753	8029	0.84	-0.25	-0.36	-1.32
YJL128C	PBS2	10953	13022	0.84	-0.25	-0.36	-1.32
YER100W	UBC6	11564	13749	0.84	-0.25	-0.36	-1.32
YLR462W		5979	7110	0.84	-0.25	-0.36	-1.32
YJL003W		3684	4383	0.84	-0.25	-0.36	-1.32

YOR215C		23148	27541	0.84	-0.25	-0.36	-1.32
YLR163C	MAS1	23234	27666	0.84	-0.25	-0.36	-1.32
YLR104W		5814	6926	0.84	-0.25	-0.36	-1.33
YBR158W	ICS4	31267	37248	0.84	-0.25	-0.36	-1.33
YBL038W	MRPL16	27009	32188	0.84	-0.25	-0.37	-1.33
YNR036C		19347	23064	0.84	-0.25	-0.37	-1.33
YJL031C	BET4	7599	9061	0.84	-0.25	-0.37	-1.33
YMR072W	ABF2	13401	15982	0.84	-0.25	-0.37	-1.33
YEL034W	HYP2	34195	40810	0.84	-0.26	-0.37	-1.34
YKR068C	BET3	10805	12900	0.84	-0.26	-0.37	-1.34
YOR332W	VMA4	29066	34711	0.84	-0.26	-0.37	-1.34
YKL207W		12712	15191	0.84	-0.26	-0.37	-1.34
YBL082C	RHK1	1874	2240	0.84	-0.26	-0.37	-1.34
YNL093W	YPT53	351	420	0.84	-0.26	-0.37	-1.35
YER143W	DDI1	5008	5988	0.84	-0.26	-0.37	-1.35
YGR055W	MUP1	17136	20496	0.84	-0.26	-0.37	-1.35
YPL057C	SUR1	4406	5270	0.84	-0.26	-0.37	-1.35
YBR106W	PHO88	32647	39052	0.84	-0.26	-0.37	-1.35
YPL054W	LEE1	1837	2198	0.84	-0.26	-0.37	-1.35
YLR421C		14085	16850	0.84	-0.26	-0.37	-1.35
YHL002W		9843	11780	0.84	-0.26	-0.37	-1.35
YGR279C	SCW4	38502	46085	0.84	-0.26	-0.37	-1.35
YBR082C	UBC4	12961	15515	0.84	-0.26	-0.37	-1.35
YLR227C		7966	9538	0.84	-0.26	-0.37	-1.35
YJL107C		1333	1596	0.84	-0.26	-0.37	-1.35
YNL071W	LAT1	12786	15313	0.83	-0.26	-0.37	-1.35
YJL073W	JEM1	2518	3017	0.83	-0.26	-0.37	-1.36
YPL087W	YDC1	5552	6652	0.83	-0.26	-0.37	-1.36
YLR138W	NHA1	14056	16845	0.83	-0.26	-0.37	-1.36
YDL026W		7538	9036	0.83	-0.26	-0.37	-1.36
YJR025C	BNA1	9184	11015	0.83	-0.26	-0.37	-1.36
YKR091W	SRL3	6544	7849	0.83	-0.26	-0.37	-1.36
YBR269C		12206	14642	0.83	-0.26	-0.37	-1.36
YOR193W		7973	9573	0.83	-0.26	-0.38	-1.37
YAL042W	FUN9	21263	25534	0.83	-0.26	-0.38	-1.37
YOL038W	PRE6	23802	28583	0.83	-0.26	-0.38	-1.37

YNR022C		22916	27525	0.83	-0.26	-0.38	-1.37
YIL123W	SIM1	20394	24505	0.83	-0.26	-0.38	-1.37
YCRX21C		6611	7946	0.83	-0.27	-0.38	-1.37
YEL026W	SNU13	44003	52890	0.83	-0.27	-0.38	-1.37
YER058W	PET117	9653	11607	0.83	-0.27	-0.38	-1.38
YDR084C		10440	12559	0.83	-0.27	-0.38	-1.38
YER189W		3434	4131	0.83	-0.27	-0.38	-1.38
YPL163C	SVS1	10470	12597	0.83	-0.27	-0.38	-1.38
YHR096C	HXT5	18984	22841	0.83	-0.27	-0.38	-1.38
YBR132C	AGP2	1710	2058	0.83	-0.27	-0.38	-1.38
YLR429W	CRN1	7739	9313	0.83	-0.27	-0.38	-1.38
YDL059C	RAD59	1103	1328	0.83	-0.27	-0.38	-1.38
YLR464W		7493	9022	0.83	-0.27	-0.38	-1.38
YHR122W		8883	10703	0.83	-0.27	-0.38	-1.39
YGR210C		23017	27748	0.83	-0.27	-0.38	-1.39
YKL178C	STE3	5636	6800	0.83	-0.27	-0.38	-1.39
YKL044W		2355	2842	0.83	-0.27	-0.38	-1.39
YDL128W	VCX1	32064	38712	0.83	-0.27	-0.38	-1.40
YNL010W		43152	52110	0.83	-0.27	-0.38	-1.40
YDR328C	SKP1	35655	43060	0.83	-0.27	-0.38	-1.40
YHR039C-B		14411	17408	0.83	-0.27	-0.38	-1.40
YPL114W		2529	3055	0.83	-0.27	-0.38	-1.40
YBR126C	TPS1	5608	6775	0.83	-0.27	-0.39	-1.40
YKL206C		6868	8299	0.83	-0.27	-0.39	-1.40
YDR099W	BMH2	21336	25784	0.83	-0.27	-0.39	-1.40
YBL040C	ERD2	14749	17838	0.83	-0.27	-0.39	-1.41
YPR197C		8813	10666	0.83	-0.28	-0.39	-1.41
YDR294C	DPL1	17498	21186	0.83	-0.28	-0.39	-1.41
YLR089C		8716	10554	0.83	-0.28	-0.39	-1.41
YLR340W	RPP0	18470	22372	0.83	-0.28	-0.39	-1.41
YJL126W	NIT2	10699	12960	0.83	-0.28	-0.39	-1.41
YGL153W	PEX14	13587	16467	0.83	-0.28	-0.39	-1.42
YIR037W	HYR1	8049	9759	0.82	-0.28	-0.39	-1.42
YLR207W	HRD3	6373	7727	0.82	-0.28	-0.39	-1.42
YPR078C		3065	3720	0.82	-0.28	-0.39	-1.42
YFL016C	MDJ1	20154	24468	0.82	-0.28	-0.39	-1.43

YJR045C	SSC1	4816	5849	0.82	-0.28	-0.39	-1.43
YPR053C		6103	7413	0.82	-0.28	-0.39	-1.43
YHL036W	MUP3	2180	2649	0.82	-0.28	-0.39	-1.43
YBL036C		39847	48414	0.82	-0.28	-0.39	-1.43
YCL056C		5065	6156	0.82	-0.28	-0.39	-1.43
YGR213C	RTA1	1226	1491	0.82	-0.28	-0.39	-1.43
YHR069C	RRP4	28537	34721	0.82	-0.28	-0.40	-1.44
YMR312W		10394	12647	0.82	-0.28	-0.40	-1.44
YML054C	CYB2	2828	3442	0.82	-0.28	-0.40	-1.44
YGL009C	LEU1	42137	51312	0.82	-0.28	-0.40	-1.44
YHR202W		8630	10512	0.82	-0.28	-0.40	-1.44
YER051W		4299	5237	0.82	-0.28	-0.40	-1.44
YFR010W	UBP6	10276	12518	0.82	-0.28	-0.40	-1.44
YAL055W		15977	19463	0.82	-0.28	-0.40	-1.44
YDR400W		8302	10114	0.82	-0.28	-0.40	-1.44
YJL192C		19137	23315	0.82	-0.28	-0.40	-1.44
YKL195W		10999	13403	0.82	-0.29	-0.40	-1.45
YHR136C	SPL2	6329	7713	0.82	-0.29	-0.40	-1.45
YKR002W	PAP1	16481	20086	0.82	-0.29	-0.40	-1.45
YPR107C	YTH1	3281	3999	0.82	-0.29	-0.40	-1.45
YGR086C		38350	46747	0.82	-0.29	-0.40	-1.45
YLL060C	GTT2	3866	4715	0.82	-0.29	-0.40	-1.45
YGL221C	NIF3	10673	13025	0.82	-0.29	-0.40	-1.45
YFL005W	SEC4	17322	21140	0.82	-0.29	-0.40	-1.45
YPL244C		12606	15389	0.82	-0.29	-0.40	-1.46
YOR236W	DFR1	14275	17432	0.82	-0.29	-0.40	-1.46
YLL023C		12063	14734	0.82	-0.29	-0.40	-1.46
YGL135W	RPL1B	38075	46607	0.82	-0.29	-0.40	-1.47
YDR382W	RPP2B	41255	50505	0.82	-0.29	-0.40	-1.47
YHR206W	SKN7	759	929	0.82	-0.29	-0.40	-1.47
YFL046W		3843	4707	0.82	-0.29	-0.41	-1.47
YNL300W		8633	10575	0.82	-0.29	-0.41	-1.47
YOR167C	RPS28A	34452	42212	0.82	-0.29	-0.41	-1.47
YDR214W		12764	15639	0.82	-0.29	-0.41	-1.47
YDL147W	RPN5	27137	33251	0.82	-0.29	-0.41	-1.47
YJR010C-A	SPC1	9807	12019	0.82	-0.29	-0.41	-1.48

YGR026W		15471	18961	0.82	-0.29	-0.41	-1.48
YNL055C	POR1	28813	35328	0.82	-0.29	-0.41	-1.48
YDL097C	RPN6	10211	12520	0.82	-0.29	-0.41	-1.48
YBR096W		20932	25689	0.81	-0.30	-0.41	-1.48
YDL010W		10394	12761	0.81	-0.30	-0.41	-1.49
YPL220W	RPL1A	33677	41353	0.81	-0.30	-0.41	-1.49
YNL217W		15850	19464	0.81	-0.30	-0.41	-1.49
YGR254W	ENO1	53262	65434	0.81	-0.30	-0.41	-1.49
YPL004C		26675	32778	0.81	-0.30	-0.41	-1.49
YBR243C	ALG7	10504	12917	0.81	-0.30	-0.41	-1.49
YBR171W	SEC66	20443	25140	0.81	-0.30	-0.41	-1.49
YKL046C		3450	4247	0.81	-0.30	-0.41	-1.50
YDR411C		11973	14738	0.81	-0.30	-0.41	-1.50
YDR542W		3193	3934	0.81	-0.30	-0.41	-1.50
YER030W		12358	15233	0.81	-0.30	-0.41	-1.51
YOL165C	AAD15	4684	5775	0.81	-0.30	-0.41	-1.51
YLR079W	SIC1	15735	19407	0.81	-0.30	-0.42	-1.51
YBR149W	ARA1	14990	18489	0.81	-0.30	-0.42	-1.51
YNL160W	YGP1	18297	22570	0.81	-0.30	-0.42	-1.51
YPR165W	RHO1	33449	41282	0.81	-0.30	-0.42	-1.51
YMR314W	PRE5	31606	39016	0.81	-0.30	-0.42	-1.51
YPL010W	RET3	18116	22382	0.81	-0.31	-0.42	-1.52
YJL048C		10123	12509	0.81	-0.31	-0.42	-1.52
YPL098C		18139	22418	0.81	-0.31	-0.42	-1.52
YNR075W	COS10	1564	1933	0.81	-0.31	-0.42	-1.52
YBL002W	HTB2	52406	64792	0.81	-0.31	-0.42	-1.52
YPL221W	BOP1	6126	7575	0.81	-0.31	-0.42	-1.52
YGL080W		8518	10534	0.81	-0.31	-0.42	-1.52
YGR152C	RSR1	21727	26870	0.81	-0.31	-0.42	-1.52
YEL046C	GLY1	10666	13192	0.81	-0.31	-0.42	-1.52
YER052C	HOM3	1753	2170	0.81	-0.31	-0.42	-1.53
YPR181C	SEC23	26323	32598	0.81	-0.31	-0.42	-1.53
YGL106W	MLC1	3765	4663	0.81	-0.31	-0.42	-1.53
YML117W-A		4123	5108	0.81	-0.31	-0.42	-1.53
YGL053W		12526	15529	0.81	-0.31	-0.42	-1.54
YCL035C	GRX1	13321	16518	0.81	-0.31	-0.42	-1.54

YOR041C		3580	4441	0.81	-0.31	-0.42	-1.54
YKL107W		2044	2537	0.81	-0.31	-0.42	-1.54
YGR120C	SEC35	19807	24590	0.81	-0.31	-0.42	-1.54
YLR391W		5423	6734	0.81	-0.31	-0.42	-1.54
YLR364W		3344	4154	0.80	-0.31	-0.43	-1.55
YBR301W		5139	6387	0.80	-0.31	-0.43	-1.55
YER019C-A	SBH2	34007	42289	0.80	-0.31	-0.43	-1.55
YPR199C	ARR1	14034	17458	0.80	-0.31	-0.43	-1.55
YOR189W		16943	21081	0.80	-0.32	-0.43	-1.55
YML133C		23795	29609	0.80	-0.32	-0.43	-1.56
YBR101C		12439	15482	0.80	-0.32	-0.43	-1.56
YNR076W	PAU6	4236	5275	0.80	-0.32	-0.43	-1.56
YKL137W		15929	19848	0.80	-0.32	-0.43	-1.56
YNR004W		2421	3017	0.80	-0.32	-0.43	-1.56
YDR513W	TTR1	13087	16338	0.80	-0.32	-0.43	-1.57
YLR043C	TRX1	31483	39315	0.80	-0.32	-0.43	-1.57
YDL110C		17013	21247	0.80	-0.32	-0.43	-1.57
YAL028W		11199	13986	0.80	-0.32	-0.43	-1.57
YMR034C		1396	1744	0.80	-0.32	-0.43	-1.58
YKL145W	RPT1	17445	21804	0.80	-0.32	-0.43	-1.58
YNL293W	MSB3	9565	11958	0.80	-0.32	-0.43	-1.58
YGR156W		5668	7090	0.80	-0.32	-0.44	-1.58
YMR297W	PRC1	28420	35562	0.80	-0.32	-0.44	-1.58
YJL178C		15869	19863	0.80	-0.32	-0.44	-1.59
YER131W	RPS26B	51441	64398	0.80	-0.32	-0.44	-1.59
YFL014W	HSP12	4459	5584	0.80	-0.32	-0.44	-1.59
YIL124W	AYR1	17245	21600	0.80	-0.32	-0.44	-1.59
YNL245C		11368	14243	0.80	-0.33	-0.44	-1.59
YDL137W	ARF2	52216	65437	0.80	-0.33	-0.44	-1.59
YDR378C	LSM6	8362	10481	0.80	-0.33	-0.44	-1.59
YOL011W	PLB3	15260	19129	0.80	-0.33	-0.44	-1.59
YOR223W		13223	16584	0.80	-0.33	-0.44	-1.60
YER063W	THO1	17237	21634	0.80	-0.33	-0.44	-1.60
YGL137W	SEC27	16319	20495	0.80	-0.33	-0.44	-1.60
YIR043C		12502	15710	0.80	-0.33	-0.44	-1.61
YKL080W	VMA5	27695	34812	0.80	-0.33	-0.44	-1.61

YHR156C		3329	4186	0.80	-0.33	-0.44	-1.61
YDL143W	CCT4	32370	40711	0.80	-0.33	-0.44	-1.61
YKR013W	PRY2	1513	1904	0.79	-0.33	-0.44	-1.61
YER087W		3671	4619	0.79	-0.33	-0.44	-1.61
YGR248W	SOL4	1789	2252	0.79	-0.33	-0.44	-1.61
YLR093C	NYV1	25357	31912	0.79	-0.33	-0.44	-1.61
YGL057C		12056	15176	0.79	-0.33	-0.44	-1.62
YBR107C	MCM19	4072	5127	0.79	-0.33	-0.44	-1.62
YNL134C		17556	22113	0.79	-0.33	-0.45	-1.62
YDR380W		4726	5957	0.79	-0.33	-0.45	-1.62
YDR154C		22139	27918	0.79	-0.33	-0.45	-1.63
YOR155C		2116	2669	0.79	-0.33	-0.45	-1.63
YPR167C	MET16	5180	6539	0.79	-0.34	-0.45	-1.63
YLR327C		6826	8624	0.79	-0.34	-0.45	-1.64
YGL001C		37331	47185	0.79	-0.34	-0.45	-1.64
YMR292W	GOT1	21315	26965	0.79	-0.34	-0.45	-1.64
YKL196C	YKT6	4303	5449	0.79	-0.34	-0.45	-1.65
YDL141W	BPL1	6480	8208	0.79	-0.34	-0.45	-1.65
YDR391C		7571	9594	0.79	-0.34	-0.45	-1.65
YDR394W	RPT3	10876	13787	0.79	-0.34	-0.45	-1.65
YLL062C		8931	11326	0.79	-0.34	-0.46	-1.65
YOR285W		13528	17158	0.79	-0.34	-0.46	-1.66
YOR225W		16220	20587	0.79	-0.34	-0.46	-1.66
YHR115C		5547	7044	0.79	-0.34	-0.46	-1.66
YMR276W	DSK2	3176	4035	0.79	-0.35	-0.46	-1.66
YBR201W	DER1	4144	5265	0.79	-0.35	-0.46	-1.67
YDL135C	RDI1	32161	40868	0.79	-0.35	-0.46	-1.67
YDR262W		17044	21665	0.79	-0.35	-0.46	-1.67
YDR134C		30565	38885	0.79	-0.35	-0.46	-1.67
YDR151C	CTH1	10793	13740	0.79	-0.35	-0.46	-1.68
YBR302C	COS2	15021	19124	0.79	-0.35	-0.46	-1.68
YDL127W	PCL2	4328	5511	0.79	-0.35	-0.46	-1.68
YPR127W		5312	6768	0.78	-0.35	-0.46	-1.68
YNL157W		14145	18024	0.78	-0.35	-0.46	-1.68
YDR224C	HTB1	42574	54264	0.78	-0.35	-0.46	-1.68
YLR354C	TAL1	27221	34701	0.78	-0.35	-0.46	-1.68

YHR180W		2163	2759	0.78	-0.35	-0.46	-1.69
YGR186W	TFG1	9003	11488	0.78	-0.35	-0.46	-1.69
YDL172C		7329	9354	0.78	-0.35	-0.46	-1.69
YLR257W		20212	25802	0.78	-0.35	-0.46	-1.69
YOR103C	OST2	20547	26230	0.78	-0.35	-0.46	-1.69
YEL075C		7032	8978	0.78	-0.35	-0.46	-1.69
YKR046C		2602	3322	0.78	-0.35	-0.47	-1.69
YOR020C	HSP10	30302	38717	0.78	-0.35	-0.47	-1.69
YAL068C		6817	8711	0.78	-0.35	-0.47	-1.69
YCLX04W		4384	5602	0.78	-0.35	-0.47	-1.69
YGR028W	MSP1	4148	5301	0.78	-0.35	-0.47	-1.70
YDL023C		2320	2965	0.78	-0.35	-0.47	-1.70
YNL006W	LST8	15817	20218	0.78	-0.35	-0.47	-1.70
YER079W		1152	1474	0.78	-0.36	-0.47	-1.70
YIL042C		3761	4812	0.78	-0.36	-0.47	-1.70
YFR052W	RPN12	21062	26966	0.78	-0.36	-0.47	-1.70
YGL157W		8077	10343	0.78	-0.36	-0.47	-1.71
YFR003C		8670	11105	0.78	-0.36	-0.47	-1.71
YCR005C	CIT2	6372	8171	0.78	-0.36	-0.47	-1.71
YER087C-A	SBH1	13979	17930	0.78	-0.36	-0.47	-1.71
YOR348C	PUT4	2621	3362	0.78	-0.36	-0.47	-1.72
YEL071W		12468	15998	0.78	-0.36	-0.47	-1.72
YDR519W	FKB2	9651	12385	0.78	-0.36	-0.47	-1.72
YOR131C		10187	13099	0.78	-0.36	-0.48	-1.73
YJR019C	TES1	5351	6883	0.78	-0.36	-0.48	-1.73
YGR209C	TRX2	13676	17600	0.78	-0.36	-0.48	-1.73
YPL112C		15985	20576	0.78	-0.36	-0.48	-1.73
YGR268C		6105	7860	0.78	-0.36	-0.48	-1.73
YDR230W		15728	20249	0.78	-0.36	-0.48	-1.73
YNL063W		3862	4974	0.78	-0.37	-0.48	-1.74
YDL126C	CDC48	22354	28800	0.78	-0.37	-0.48	-1.74
YGL011C	SCL1	29666	38229	0.78	-0.37	-0.48	-1.74
YKL211C	TRP3	20059	25868	0.78	-0.37	-0.48	-1.74
YPL170W		19593	25268	0.78	-0.37	-0.48	-1.74
YOR230W	WTM1	11631	15011	0.77	-0.37	-0.48	-1.75
YFL062W	COS4	16800	21687	0.77	-0.37	-0.48	-1.75

YJR008W		7135	9226	0.77	-0.37	-0.48	-1.76
YHL034C	SBP1	21282	27536	0.77	-0.37	-0.48	-1.76
YOR226C	ISU2	8052	10425	0.77	-0.37	-0.49	-1.76
YPL218W	SAR1	25830	33448	0.77	-0.37	-0.49	-1.76
YPL156C		13420	17382	0.77	-0.37	-0.49	-1.77
YOR097C		5208	6746	0.77	-0.37	-0.49	-1.77
YMR173W-A		6552	8488	0.77	-0.37	-0.49	-1.77
YMR237W		6221	8060	0.77	-0.37	-0.49	-1.77
YML075C	HMG1	16137	20923	0.77	-0.37	-0.49	-1.77
YBR221C	PDB1	30194	39174	0.77	-0.38	-0.49	-1.77
YDR071C		28876	37498	0.77	-0.38	-0.49	-1.78
YHL048W	COS8	16931	21988	0.77	-0.38	-0.49	-1.78
YLR229C	CDC42	39718	51598	0.77	-0.38	-0.49	-1.78
YAL049C		20361	26455	0.77	-0.38	-0.49	-1.78
YLR188W	MDL1	12083	15709	0.77	-0.38	-0.49	-1.79
YJR023C		6827	8877	0.77	-0.38	-0.49	-1.79
YPR065W	ROX1	5143	6691	0.77	-0.38	-0.49	-1.79
YMR035W	IMP2	2668	3472	0.77	-0.38	-0.49	-1.79
YBR173C	UMP1	20678	26921	0.77	-0.38	-0.49	-1.79
YER012W	PRE1	16526	21524	0.77	-0.38	-0.49	-1.79
YOR099W	KTR1	11278	14695	0.77	-0.38	-0.49	-1.80
YMR186W	HSC82	7009	9134	0.77	-0.38	-0.49	-1.80
YGL073W	HSF1	7798	10163	0.77	-0.38	-0.49	-1.80
YMR039C	SUB1	6134	7998	0.77	-0.38	-0.50	-1.80
YNR059W	MNT4	1907	2490	0.77	-0.38	-0.50	-1.81
YIL013C	PDR11	1621	2117	0.77	-0.38	-0.50	-1.81
YLR186W		2193	2867	0.76	-0.39	-0.50	-1.82
YOR185C	GSP2	12398	16210	0.76	-0.39	-0.50	-1.82
YPL094C	SEC62	28943	37843	0.76	-0.39	-0.50	-1.82
YKL117W	SBA1	14108	18450	0.76	-0.39	-0.50	-1.82
YPR063C		9594	12548	0.76	-0.39	-0.50	-1.82
YNL281W	HCH1	30791	40341	0.76	-0.39	-0.50	-1.83
YOR248W		11297	14804	0.76	-0.39	-0.50	-1.83
YOR209C	NPT1	24368	31962	0.76	-0.39	-0.50	-1.83
YIL016W	SNL1	16001	21023	0.76	-0.39	-0.51	-1.84
YBR066C	NRG2	2998	3941	0.76	-0.39	-0.51	-1.84

YOR069W	VPS5	8220	10811	0.76	-0.40	-0.51	-1.85
YGL047W		14208	18689	0.76	-0.40	-0.51	-1.85
YBL050W	SEC17	19847	26107	0.76	-0.40	-0.51	-1.85
YGR132C	PHB1	20902	27501	0.76	-0.40	-0.51	-1.85
YBR241C		2250	2961	0.76	-0.40	-0.51	-1.85
YGR284C		17433	22959	0.76	-0.40	-0.51	-1.85
YNL155W		9308	12261	0.76	-0.40	-0.51	-1.85
YBR004C		1191	1570	0.76	-0.40	-0.51	-1.86
YPL196W		19982	26358	0.76	-0.40	-0.51	-1.86
YPL234C	TFP3	18411	24304	0.76	-0.40	-0.51	-1.87
YJL079C	PRY1	1440	1902	0.76	-0.40	-0.51	-1.87
YOL032W		15502	20513	0.76	-0.40	-0.52	-1.88
YGR008C	STF2	6841	9052	0.76	-0.40	-0.52	-1.88
YHR138C		4355	5767	0.76	-0.41	-0.52	-1.88
YHR191C	CTF8	1930	2556	0.76	-0.41	-0.52	-1.88
YGL087C	MMS2	8874	11751	0.76	-0.41	-0.52	-1.88
YDR533C		12964	17185	0.75	-0.41	-0.52	-1.89
YDL100C		16659	22089	0.75	-0.41	-0.52	-1.89
YAL012W	CYS3	28272	37500	0.75	-0.41	-0.52	-1.89
YFR050C	PRE4	11325	15024	0.75	-0.41	-0.52	-1.89
YBR253W	SRB6	18530	24588	0.75	-0.41	-0.52	-1.89
YJR017C	ESS1	6115	8120	0.75	-0.41	-0.52	-1.90
YAL030W	SNC1	11433	15182	0.75	-0.41	-0.52	-1.90
YML092C	PRE8	18119	24069	0.75	-0.41	-0.52	-1.90
YEL076C-A		10384	13802	0.75	-0.41	-0.52	-1.90
YIL108W		6504	8660	0.75	-0.41	-0.53	-1.91
YGR121C	MEP1	3532	4708	0.75	-0.41	-0.53	-1.92
YPL206C		16379	21834	0.75	-0.41	-0.53	-1.92
YDR258C	HSP78	3546	4732	0.75	-0.42	-0.53	-1.92
YLR259C	HSP60	25591	34165	0.75	-0.42	-0.53	-1.92
YNL064C	YDJ1	26726	35693	0.75	-0.42	-0.53	-1.93
YOR289W		1855	2478	0.75	-0.42	-0.53	-1.93
YDR518W	EUG1	3322	4439	0.75	-0.42	-0.53	-1.93
YDR476C		5557	7427	0.75	-0.42	-0.53	-1.93
YDR233C		27256	36430	0.75	-0.42	-0.53	-1.93
YOL122C	SMF1	3982	5324	0.75	-0.42	-0.53	-1.93

YOR085W	OST3	14446	19318	0.75	-0.42	-0.53	-1.93
YJL035C	TAD2	2768	3706	0.75	-0.42	-0.53	-1.94
YPL088W		11486	15392	0.75	-0.42	-0.53	-1.94
YER094C	PUP3	23686	31749	0.75	-0.42	-0.54	-1.95
YOL013C	HRD1	12272	16450	0.75	-0.42	-0.54	-1.95
YAL004W		15508	20790	0.75	-0.42	-0.54	-1.95
YOR007C	SGT2	11988	16089	0.75	-0.42	-0.54	-1.95
YOR117W	RPT5	20328	27297	0.74	-0.43	-0.54	-1.95
YER062C	HOR2	3653	4917	0.74	-0.43	-0.54	-1.97
YCL038C		5076	6855	0.74	-0.43	-0.55	-1.98
YMR315W		8349	11300	0.74	-0.44	-0.55	-2.00
YER072W	NRF1	38435	52090	0.74	-0.44	-0.55	-2.00
YPL250C		10700	14509	0.74	-0.44	-0.55	-2.01
YLR251W		1702	2310	0.74	-0.44	-0.55	-2.01
YBR099C		3427	4652	0.74	-0.44	-0.55	-2.01
YPL154C	PEP4	27813	37777	0.74	-0.44	-0.55	-2.01
YBL058W	SHP1	21408	29121	0.74	-0.44	-0.56	-2.02
YDR242W	AMD2	1077	1466	0.73	-0.44	-0.56	-2.03
YDL169C	UGX2	3960	5390	0.73	-0.44	-0.56	-2.03
YOR052C		10592	14416	0.73	-0.44	-0.56	-2.03
YGL090W	LIF1	1295	1765	0.73	-0.45	-0.56	-2.03
YKL058W	TOA2	29260	39913	0.73	-0.45	-0.56	-2.04
YOR157C	PUP1	36827	50299	0.73	-0.45	-0.56	-2.04
YNL277W	MET2	12951	17693	0.73	-0.45	-0.56	-2.05
YPL078C	ATP4	35520	48525	0.73	-0.45	-0.56	-2.05
YDR055W	PST1	969	1325	0.73	-0.45	-0.56	-2.05
YOR089C	VPS21	16945	23169	0.73	-0.45	-0.56	-2.05
YCL009C	ILV6	27315	37351	0.73	-0.45	-0.56	-2.05
YOL109W	ZEO1	15055	20604	0.73	-0.45	-0.57	-2.05
YGL127C	SOH1	12925	17714	0.73	-0.45	-0.57	-2.06
YFL056C	AAD6	6227	8539	0.73	-0.46	-0.57	-2.07
YHR146W		29405	40587	0.72	-0.46	-0.58	-2.10
YDR222W		10729	14822	0.72	-0.47	-0.58	-2.10
YDL193W		14288	19782	0.72	-0.47	-0.58	-2.12
YPL149W	APG5	6297	8722	0.72	-0.47	-0.58	-2.12
YLR065C		21910	30362	0.72	-0.47	-0.58	-2.12

YNL208W		17690	24516	0.72	-0.47	-0.58	-2.12
YKL065C	YET1	8597	11917	0.72	-0.47	-0.58	-2.12
YCLX08C		879	1219	0.72	-0.47	-0.58	-2.12
YMR173W	DDR48	6826	9490	0.72	-0.48	-0.59	-2.14
YKL192C	ACP1	12437	17303	0.72	-0.48	-0.59	-2.14
YNL241C	ZWF1	21215	29533	0.72	-0.48	-0.59	-2.14
YPR154W		4053	5649	0.72	-0.48	-0.59	-2.15
YGL225W	GOG5	38068	53130	0.72	-0.48	-0.59	-2.16
YBR100W		1012	1413	0.72	-0.48	-0.59	-2.16
YGL105W	ARC1	36072	50394	0.72	-0.48	-0.59	-2.16
YOR385W		11371	15888	0.72	-0.48	-0.59	-2.16
YOR362C	PRE10	21590	30169	0.72	-0.48	-0.60	-2.16
YDR210W		13512	18920	0.71	-0.49	-0.60	-2.17
YBR169C	SSE2	3762	5268	0.71	-0.49	-0.60	-2.17
YCL040W	GLK1	23017	32267	0.71	-0.49	-0.60	-2.18
YDR043C	NRG1	2072	2907	0.71	-0.49	-0.60	-2.18
YIL040W		25928	36400	0.71	-0.49	-0.60	-2.19
YDL070W	BDF2	9528	13388	0.71	-0.49	-0.60	-2.19
YER103W	SSA4	7755	10913	0.71	-0.49	-0.61	-2.20
YCL026C		2643	3723	0.71	-0.49	-0.61	-2.21
YIL153W	RRD1	2246	3165	0.71	-0.49	-0.61	-2.21
YML011C		8506	11988	0.71	-0.50	-0.61	-2.21
YIL034C	CAP2	25314	35680	0.71	-0.50	-0.61	-2.21
YOR239W		4118	5804	0.71	-0.50	-0.61	-2.21
YGR048W	UFD1	6958	9814	0.71	-0.50	-0.61	-2.21
YGR014W	MSB2	3941	5567	0.71	-0.50	-0.61	-2.22
YKR042W	UTH1	32369	45784	0.71	-0.50	-0.61	-2.23
YLR303W	MET17	28696	40611	0.71	-0.50	-0.61	-2.23
YLR350W		12638	17943	0.70	-0.51	-0.62	-2.25
YPL028W	ERG10	32853	46734	0.70	-0.51	-0.62	-2.26
YJL118W		6288	8960	0.70	-0.51	-0.62	-2.27
YDR059C	UBC5	2130	3037	0.70	-0.51	-0.62	-2.27
YHR150W		8480	12100	0.70	-0.51	-0.63	-2.27
YOL129W		13475	19264	0.70	-0.52	-0.63	-2.28
YKL213C	DOA1	2916	4176	0.70	-0.52	-0.63	-2.29
YER042W	MXR1	25055	35905	0.70	-0.52	-0.63	-2.30

YFR004W	RPN11	21858	31383	0.70	-0.52	-0.63	-2.31
YOL005C	RPB11	21424	30793	0.70	-0.52	-0.64	-2.31
YMR178W		13549	19482	0.70	-0.52	-0.64	-2.31
YLR216C	CPR6	21200	30506	0.69	-0.53	-0.64	-2.32
YOL019W		8823	12696	0.69	-0.53	-0.64	-2.32
YLR378C	SEC61	6890	9926	0.69	-0.53	-0.64	-2.32
YEL060C	PRB1	1262	1822	0.69	-0.53	-0.64	-2.34
YCL018W	LEU2	36250	52589	0.69	-0.54	-0.65	-2.36
YKR012C		13871	20147	0.69	-0.54	-0.65	-2.37
YJL060W		15131	22034	0.69	-0.54	-0.65	-2.38
YBR078W	ECM33	28361	41455	0.68	-0.55	-0.66	-2.40
YLR213C	CRR1	19132	27980	0.68	-0.55	-0.66	-2.40
YCR013C		36783	53814	0.68	-0.55	-0.66	-2.40
YHR181W		1105	1617	0.68	-0.55	-0.66	-2.41
YGL038C	OCH1	3938	5770	0.68	-0.55	-0.66	-2.41
YCR061W		9237	13534	0.68	-0.55	-0.66	-2.41
YBL078C	AUT7	10139	14876	0.68	-0.55	-0.67	-2.42
YDR155C	CPH1	14900	21905	0.68	-0.56	-0.67	-2.43
YBR162C		17232	25343	0.68	-0.56	-0.67	-2.43
YGR110W		2740	4037	0.68	-0.56	-0.67	-2.44
YKL163W	PIR3	4774	7052	0.68	-0.56	-0.68	-2.46
YOL053C-A		2674	3952	0.68	-0.56	-0.68	-2.46
YGR282C	BGL2	43572	64452	0.68	-0.56	-0.68	-2.46
YGR080W	TWF1	7362	10890	0.68	-0.56	-0.68	-2.46
YKL096W	CWP1	22890	33894	0.68	-0.57	-0.68	-2.47
YDR202C		1203	1782	0.68	-0.57	-0.68	-2.47
YGR232W		14674	21775	0.67	-0.57	-0.68	-2.48
YKL219W	COS9	2641	3924	0.67	-0.57	-0.68	-2.49
YNR010W	CSE2	11575	17219	0.67	-0.57	-0.69	-2.49
YOL163W		1033	1541	0.67	-0.58	-0.69	-2.50
YNR069C		1848	2759	0.67	-0.58	-0.69	-2.51
YPR159W	KRE6	25442	38112	0.67	-0.58	-0.70	-2.53
YPR052C	NHP6A	19018	28490	0.67	-0.58	-0.70	-2.53
YDL237W		761	1141	0.67	-0.58	-0.70	-2.53
YLR120C	YPS1	4209	6316	0.67	-0.59	-0.70	-2.54
YMR235C	RNA1	16145	24299	0.66	-0.59	-0.70	-2.55

YKL103C	LAP4	8294	12497	0.66	-0.59	-0.70	-2.56
YNL044W	YIP3	32551	49070	0.66	-0.59	-0.70	-2.56
YCL034W		20252	30600	0.66	-0.60	-0.71	-2.57
YLR370C	ARC18	14858	22475	0.66	-0.60	-0.71	-2.58
YDR309C	GIC2	570	865	0.66	-0.60	-0.71	-2.60
YGL037C		24608	37346	0.66	-0.60	-0.71	-2.60
YOR247W	SRL1	10283	15616	0.66	-0.60	-0.72	-2.60
YLR121C	YPS3	5393	8229	0.66	-0.61	-0.72	-2.63
YAL008W	FUN14	12123	18532	0.65	-0.61	-0.72	-2.63
YJL158C	CIS3	8493	13033	0.65	-0.62	-0.73	-2.65
YBR244W	AMI1	2786	4279	0.65	-0.62	-0.73	-2.66
YLR432W		14712	22660	0.65	-0.62	-0.74	-2.67
YOL016C	CMK2	2643	4075	0.65	-0.62	-0.74	-2.68
YGL166W	CUP2	7111	10982	0.65	-0.63	-0.74	-2.69
YGR161C		6878	10621	0.65	-0.63	-0.74	-2.69
YOR288C	MPD1	4969	7737	0.64	-0.64	-0.75	-2.73
YPR183W	DPM1	21349	33263	0.64	-0.64	-0.75	-2.73
YNL020C	ARK1	7784	12164	0.64	-0.64	-0.76	-2.75
YLR356W		4920	7692	0.64	-0.64	-0.76	-2.75
YMR040W		2254	3536	0.64	-0.65	-0.76	-2.77
YFR034C	PHO4	2098	3305	0.63	-0.66	-0.77	-2.79
YNL305C		11152	17728	0.63	-0.67	-0.78	-2.84
YCL043C	PDI1	18544	29612	0.63	-0.68	-0.79	-2.86
YJL108C		4838	7734	0.63	-0.68	-0.79	-2.87
YLR225C		25423	40882	0.62	-0.69	-0.80	-2.90
YHR198C		1516	2439	0.62	-0.69	-0.80	-2.90
YBR005W		4946	7966	0.62	-0.69	-0.80	-2.91
YDL022W	GPD1	9623	15530	0.62	-0.69	-0.80	-2.92
YPR158W		8163	13176	0.62	-0.69	-0.80	-2.92
YPL240C	HSP82	8025	12966	0.62	-0.69	-0.80	-2.93
YIR017C	MET28	3922	6339	0.62	-0.69	-0.81	-2.93
YAR073W		32660	53071	0.62	-0.70	-0.81	-2.96
YIL074C	SER33	39047	63646	0.61	-0.70	-0.82	-2.97
YHR044C	DOG1	5152	8410	0.61	-0.71	-0.82	-2.98
YOL031C		5979	9857	0.61	-0.72	-0.83	-3.03
YDL173W		19427	32058	0.61	-0.72	-0.84	-3.04

YPL106C	SSE1	30633	50588	0.61	-0.72	-0.84	-3.04
YGL184C		5651	9367	0.60	-0.73	-0.84	-3.06
YML028W	TSA1	20858	34774	0.60	-0.74	-0.85	-3.09
YHR216W	PUR5	37483	62556	0.60	-0.74	-0.85	-3.10
YMR304C-A		6986	11715	0.60	-0.75	-0.86	-3.12
YBR287W		38513	64623	0.60	-0.75	-0.86	-3.12
YOL007C	CSI2	15917	26778	0.59	-0.75	-0.86	-3.14
YOR027W	STI1	8401	14195	0.59	-0.76	-0.87	-3.16
YMR184W		11549	19602	0.59	-0.76	-0.88	-3.18
YNL007C	SIS1	18316	31112	0.59	-0.76	-0.88	-3.19
YOR036W	PEP12	7266	12388	0.59	-0.77	-0.88	-3.21
YAL005C	SSA1	28308	48268	0.59	-0.77	-0.88	-3.21
YDL072C		31329	53486	0.59	-0.77	-0.88	-3.21
YHR148W	IMP3	35219	60454	0.58	-0.78	-0.89	-3.24
YPL056C		1675	2886	0.58	-0.79	-0.90	-3.26
YAR075W		34152	58902	0.58	-0.79	-0.90	-3.27
YJL094C		6137	10606	0.58	-0.79	-0.90	-3.28
YNR068C		3787	6569	0.58	-0.79	-0.91	-3.30
YBL075C	SSA3	2504	4481	0.56	-0.84	-0.95	-3.46
YLR099C	ICT1	9508	17050	0.56	-0.84	-0.95	-3.47
YKR011C		1649	2963	0.56	-0.85	-0.96	-3.48
YOR220W		7001	12641	0.55	-0.85	-0.96	-3.51
YBR072W	HSP26	1385	2541	0.54	-0.88	-0.99	-3.59
YNL289W	PCL1	15296	28238	0.54	-0.88	-1.00	-3.62
YJL116C	NCA3	2726	5037	0.54	-0.89	-1.00	-3.63
YLL024C	SSA2	29740	55993	0.53	-0.91	-1.03	-3.73
YLL026W	HSP104	10838	20521	0.53	-0.92	-1.03	-3.76
YNL036W	NCE103	27558	53368	0.52	-0.95	-1.07	-3.88
YKL097W-A		17352	34160	0.51	-0.98	-1.09	-3.96
YKL001C	MET14	18044	36410	0.50	-1.01	-1.13	-4.09
YMR316C-A		6545	13208	0.50	-1.01	-1.13	-4.09
YLR109W	AHP1	24161	48847	0.49	-1.02	-1.13	-4.10
YBR016W		8440	17090	0.49	-1.02	-1.13	-4.11
YJL034W	KAR2	18889	38588	0.49	-1.03	-1.14	-4.16
YDL124W		26770	54961	0.49	-1.04	-1.15	-4.18
YMR305C	SCW10	16521	34499	0.48	-1.06	-1.17	-4.27

YLR202C		7841	16478	0.48	-1.07	-1.18	-4.30
YJL144W		7475	15771	0.47	-1.08	-1.19	-4.33
YDR171W	HSP42	4570	11196	0.41	-1.29	-1.41	-5.11
YER035W		759	1875	0.40	-1.31	-1.42	-5.15
YGR142W	BTN2	13911	35547	0.39	-1.35	-1.47	-5.33
YMR316W		7979	21029	0.38	-1.40	-1.51	-5.49
YMR251W-A	HOR7	23064	62743	0.37	-1.44	-1.56	-5.66
YBR296C	PHO89	1311	3924	0.33	-1.58	-1.69	-6.16
