# Supplementary material for: Genome-scale approaches for discovering novel nonconventional splicing substrates of the Ire1 nuclease
Source: Genome Biol. 2004 Dec 22;6(1):R3. doi: 10.1186/gb-2004-6-1-r3 (PMC549064; doi:10.1186/gb-2004-6-1-r3)
Supplement: Additional data file 4 — The captions and keys to the supplementary tables [file gb-2004-6-1-r3-s4.doc]

Captions to supplementary tables in additional data files

**Supplementary Table 1**

This table contains all results from the Ire1*-cleaved vs. mock-cleaved microarray experiment, displayed in Figure 3.

Key:

ORF ID — *Saccharomyces* Genome Database ORF designation

Gene — gene name, if any

X=Cy3 — Normalized fluorescence intensity of Cy3 (green) signal

Y=Cy5 — Normalized fluorescence intensity of Cy5 (red) signal

(X/Y) — ratio of Cy3: Cy5 normalized fluorescence intensity

log2(X/Y) — logarithm base 2 of (X/Y)

mean(log2(X/Y)) — average over all log2(X/Y)

sigma = standard deviation (Microsoft Excel STDEV) over all log2(X/Y))

N sigma — [log2(X/Y)-mean(log2(X/Y))]/sigma

**Supplementary Table 2**

All hits from the computational screen for the *HAC1* consensus cleavage site are listed here.

ORFs were considered to include the sequences 1000 nucleotides upstream of the initiation codon and 1000 nucleotides downstream of the STOP codon. Coordinates used in the table consider the first base of the initiation codon to be “+1”; negative values are therefore represent bases in the presumptive 5' UTR.

Key:(Each line represents a unique pattern match.)

ORF — *Saccharomyces* Genome Database ORF designation

5' — first nucleotide of the pattern match

3' — last nucleotide of the pattern match

5' stem — sequence of the four bases comprising the 5' leg of the stem

loop — sequence of the final nucleotide of the 5' leg of the stem; the seven nucleotides of the loop; and the first nucleotide of the 3' leg of the stem (invariably a G by assumption)

3' stem — sequence of the final four bases comprising the 3' leg of the stem

**Supplementary Table 3**

This table contains all results from the *rlg1-100* vs. wildtype microarray experiment, displayed in Figure 5.

Key:

ORF ID — *Saccharomyces* Genome Database ORF designation

Gene — gene name, if any

X=Cy3 — Normalized fluorescence intensity of Cy3 (green) signal

Y=Cy5 — Normalized fluorescence intensity of Cy5 (red) signal

(X/Y) — ratio of Cy3: Cy5 normalized fluorescence intensity

log2(X/Y) — logarithm base 2 of (X/Y)

mean(log2(X/Y)) — average over all log2(X/Y)

sigma = standard deviation (Microsoft Excel STDEV) over all log2(X/Y))

N sigma — [log2(X/Y)-mean(log2(X/Y))]/sigma
